# Identification of a potent palladium-aryldiphosphine catalytic system for high-performance carbonylation of alkenes

**DOI:** 10.1038/s41467-024-46286-9

**Published:** 2024-03-05

**Authors:** Kang Zhao, Hongli Wang, Teng Li, Shujuan Liu, Enrico Benassi, Xiao Li, Yao Yao, Xiaojun Wang, Xinjiang Cui, Feng Shi

**Affiliations:** 1 State Key Laboratory for Oxo Synthesis and Selective Oxidation, Lanzhou Institute of Chemical Physics, Chinese Academy of Sciences, No. 18 Lanzhou, PR China; 2 University of Chinese Academy of Sciences, No. 19A Beijing, PR China; 3 Novosibirsk State University, No. 2 Pigorova ul, Novosibirsk, Russian Federation; 4 Nanjing Chengzhi Clean Energy Co., LTD., Nanjing, PR China

**Keywords:** Catalyst synthesis, Homogeneous catalysis, Synthetic chemistry methodology

## Abstract

The development of stable and efficient ligands is of vital significance to enhance the catalytic performance of carbonylation reactions of alkenes. Herein, an aryldiphosphine ligand (**L11**) bearing the [Ph_2_P(ortho-C_6_H_4_)]_2_CH_2_ skeleton is reported for palladium-catalyzed regioselective carbonylation of alkenes. Compared with the industrially successful Pd/1,2-bis(di-tert-butylphosphinomethyl)benzene catalyst, catalytic efficiency catalyzed by Pd/**L11** on methoxycarbonylation of ethylene is obtained, exhibiting better catalytic performance (TON: >2,390,000; TOF: 100,000 h^−1^; selectivity: >99%) and stronger oxygen-resistance stability. Moreover, a substrate compatibility (122 examples) including chiral and bioactive alkenes or alcohols is achieved with up to 99% yield and 99% regioselectivity. Experimental and computational investigations show that the appropriate bite angle of aryldiphosphine ligand and the favorable interaction of 1,4-dioxane with Pd/**L11** synergistically contribute to high activity and selectivity while the electron deficient phosphines originated from electron delocalization endow **L11** with excellent oxygen-resistance stability.

## Abstract (web summary)

The development of stable and efficient ligands is of vital significance to enhance the catalytic performance of carbonylation reactions of alkenes. Here, the authors develop an aryldiphosphine ligand, used in palladium-catalyzed regioselective carbonylation of alkenes, exhibiting high catalytic performance and strong oxygen-resistance stability.

## Introduction

Carbonylation of alkenes via transition metal catalysis provides a straightforward and atom-efficient approach to synthesis carboxylate and its derivatives^1–5^, which represent important structural scaffolds in the pharmaceutical, polymer, cosmetics, food industries^6–9^. Various catalytic systems based on palladium, rhodium, nickel, copper and cobalt have been developed, and Pd/phosphine complexes exhibit outstanding catalytic performance under more mild reaction conditions^5,10–14^. Due to the critical role of phosphine ligands for controlling catalytic activity and selectivity on alkoxycarbonylation of alkenes, it is highly significant to design efficient phosphine ligands and elucidate how they can modulate the catalytic performance.

**Fig. 1:**
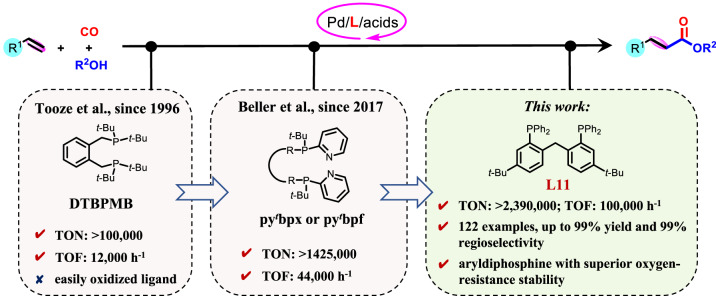
Ligand advances in palladium-catalyzed carbonylation of alkenes. TON turnover number, TOF turnover frequency, DTBPMB 1,2-bis(di-tert-butylphosphinomethyl)benzene, py^*t*^bpx 1,2-bis((tert-butyl(pyridin-2-yl)phosphanyl)methyl)benzene, py^*t*^bpf 1,1’-bis(tert-butyl(pyridin-2-yl)phosphanyl)ferrocene.

In the past decades, several phosphine ligands have been developed, exhibiting good catalytic activity and selectivity on alkoxycarbonylation of alkenes. In the 1990s, 1,2-bis(di-tert-butylphosphinomethyl)benzene (DTBPMB) was developed for palladium-catalyzed methoxycarbonylation of ethylene, producing methyl propionate (MP) with 100,000 of TON and 12,000 h^−1^ of TOF, which provides the basis to synthesize the fundamental methyl methacrylate via the Lucite’s Alpha process (Fig. 1)^15–17^. This versatile DTBPMB ligand was also applied to alkoxycarbonylation of aliphatic alkenes with high selectivity for linear esters and acids^18,19^. Moreover, great efforts on designing alkyl phosphines with bulky steric substituents have been devoted for controlling the activity and selectivity of alkoxycarbonylation of alkenes, such as, bis(di-tert-butylphosphanyl)benzene^20,21^ and bis(diphosphaadmantyl)alkanes^22^. Nevertheless, these catalytic systems are still limited with the unsatisfactory reaction performance and inferior ligand stability. Recently, Beller’s group designed two ligands containing both tertiary butyl and pyridyl groups on the P-atom, 1,2-bis((tert-butyl(pyridin-2-yl)phosphanyl)methyl)benzene (py^t^bpx) and 1,1’-bis(tert-butyl(pyridin-2-yl)phosphanyl)ferrocene (py^t^bpf), achieving better catalytic efficiency with 1,425,000 of TON and 44,000 h^−1^ of TOF on methoxycarbonylation of ethylene (Fig. 1)^23–25^. Despite these state-of-the-art systems, the above-mentioned alkyl phosphines still suffer from drawbacks such as expensive cost and easy oxidation. From the point of view of both academia and industry, it is highly desirable to develop a stable and potent phosphine ligand that is efficient, inexpensive, practically operated, and resistant to air.

As known, aryl phosphines are more stable toward oxidation due to better electron delocalization compared to the aliphatic counterparts. However, aryl phosphines normally afford lower activity than alkyl ones in alkoxycarbonylation of alkenes^17,23,26–28^. On the other hand, the bite angle (P-M-P) of diphosphine ligands coordinated with metal centers has been elucidated to be vital for the modulation of activity and selectivity because the energy barriers of key transition state and intermediates during the catalytic cycle can be regulated by changing the electronic structure and steric hindrance^29,30^. In alkoxycarbonylation, the electron density of metal center decreases with the bite angle of P-M-P increasing, thus facilitating nucleophilic attack of the alcohols and methanolysis of acyl palladium species^19,31^. However, the chelation ability is weakened with excessive large bite angle, leading to low catalytic performance^26,29^. Therefore, the appropriate P-Pd-P bite angle is beneficial for acquiring high reactivity on alkoxycarbonylation of alkenes.

[Ph_2_P(ortho-C_6_H_4_)]_2_CH_2_ has been developed as one of the most attractive skeletons of aryldiphosphine ligands where the aryl phosphine can rotate within limits owing to the presence of [CH_2_], leading to inherent but limited flexibility and optimal coordination mode with the metal center^32,33^. It can be speculated that the [Ph_2_P(ortho-C_6_H_4_)]_2_CH_2_ skeleton incorporating appropriate substituents might provide the sweet P-Pd-P bite angle for alkoxycarbonylation of alkenes with high catalytic performance. As such, it has resulted in a call for developing [Ph_2_P(ortho-C_6_H_4_)]_2_CH_2_ based aryldiphosphine ligands with appropriate bite angle and electron delocalization enabling high catalytic performance. However, no efficient aryl phosphine ligand has ever been reported up to now. Herein, we demonstrate an example of highly stable and efficient aryldiphosphine ligand based on [Ph_2_P(ortho-C_6_H_4_)]_2_CH_2_ skeleton for palladium-catalyzed carbonylation of alkenes. Compared with the reported py^t^bpx and industrially applied DTBPMB ligand, our aryldiphosphine ligand not only exhibits better catalytic performance (TON: >2,390,000; TOF: 100,000 h^−1^; selectivity: >99%), but also presents stronger oxygen-resistance stability (over 100 days of exposure under air), thus providing a potential application (1000 ppm of oxygen tolerance) for industrial production (Fig. 1).

## Results

### Catalytic performance study

**Fig. 2:**
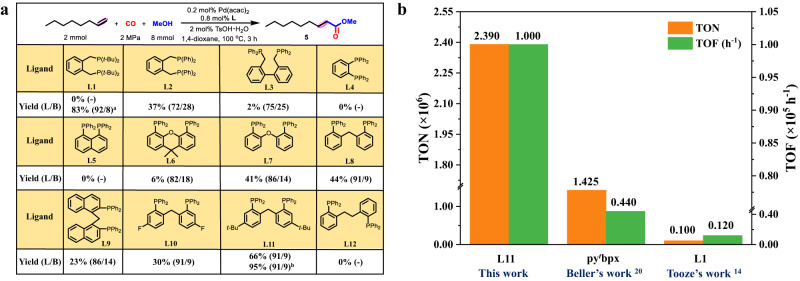
Ligand optimization and its application in methoxycarbonylation. **a** Ligand optimization for methoxycarbonylation of 1-octene; Reaction condtions: 1-octene (2 mmol), methanol (8 mmol), Pd(acac)_2_ (0.2 mol%), L (0.8 mol%), TsOH·H_2_O (2 mol%), CO (2 MPa), 1,4-dioxane (4 ml), 100 °C, 3 h, GC yields of the mixture of linear and branched esters are based on 1-octene using n-decane as the internal standard, the ratio of linear and branched esters is determined by GC analysis and is shown in parentheses; ^a^ Methanol (4 ml) instead of 1,4-dioxane as the solvent; ^b^ 1-octene (8 mmol), methanol (2 mmol), 20 h. **b** Methoxycarbonylation of ethylene at mole-scale; Reaction conditions: ethylene (2.0 mol), methanol (1200 ml), Pd(acac)_2_ (0.00069 mmol), **L11** (0.5 mmol), TsOH·H_2_O (4.0 mmol), CO (8.0 MPa), 100 °C, 48 h for TON, or 18 h for TOF. TON, turnover number. TOF, turnover frequency. py^*t*^bpx, 1,2-bis((tert-butyl(pyridin-2-yl)phosphanyl)methyl)benzene.

Initially, a variety of representative diphosphine ligands were screened when using methoxycarbonylation of 1-octene and methanol as the benchmark reaction (Fig. 2a and Supplementary Table 1). In the presence of state-of-the-art industrial ligand DTBPMB (**L1**), 83% yield and 92% regioselectivity were obtained under methanol as previous literature reported^18,19^, but difficult to actuate the transformation at all when using 1,4-dioxane. This peculiar phenomenon demonstrated that 1,4-dioxane inhibited the Pd/**L1**-catalyzed methoxycarbonylation. Other DTBPMB-type ligands (**L2** and **L3**) replacing the alkyl tert-butyl groups by aryl rings exhibited low yields and regioselectivities. Besides, aryldiphosphine ligands DPPB (**L4**) and DPPN (**L5**) with rigid skeletons showed no activity at all. However, Xantphos (**L6**) showed 6% yield and 82% regioselectivity. Interestingly, a semi-rigid aryldiphosphine ligand (**L7**) with inherent but limited flexibility promoted methoxycarbonylation of 1-octene with 41% yield and 86% regioselectivity. The conversion and selectivity were further improved by replacing the [O] linkage with [CH_2_] group (**L8**) with 44% yield and 91% regioselectivity, thus inspiring us to modify this [Ph_2_P(ortho-C_6_H_4_)]_2_CH_2_ scaffold. Ligands bearing various aryl substitutes (**L9**-**L11, L13 and L14**) were tested and tertiary butyl group modified aryldiphosphine ligand **L11** offered the optimal activity (66%) and regioselectivity (91%). However, with more flexible aryldiphosphine ligand **L12**, no activity was observed on the methoxycarbonylation of 1-octene. With the optimal aryldiphosphine ligand **L11**, influences of the solvents and other reaction parameters were investigated (Supplementary Tables 2–6, Supplementary Fig. 12, and Fig. 2a). Finally, the yield and regioselectivity were further improved up to 95% and 91%, respectively, under the optimal reaction condition: 0.2 mol% Pd(acac)_2_, 0.8 mol% **L11**, 2 mol% TsOH·H_2_O, in 4 ml 1,4-dioxane, 2 MPa CO, 100 °C for 20 h.

Methyl propionate commercially produced by Pd_2_(dba)_3_/**L1**-catalyzed methoxycarbonylation of ethylene is the starting material to synthesize methyl methacrylate (MMA), which is the important unit of polymethylmethacrylate (PMMA) generally applied in biomedicine, sensors, battery electrolytes, molecular separations, polymer conductivity, and ect^34,35^. Herein, the methoxycarbonylation of ethylene at mole-scale was successfully performed in presence of **L11** even when the Pd dosage decreased to 0.33 ppm (Fig. 2b, Supplementary Figs. 13 and 14), affording prominent activity (TON: >2,390,000; TOF: 100,000 h^−1^) and selectivity (>99%). Remarkably, this Pd(acac)_2_/**L11** catalytic system demonstrated higher catalytic efficiency than the optimal Pd(acac)_2_/py^t^bpx (TON: >1,425,000; TOF: 44,000 h^−1^; selectivity: >99%) and Pd_2_(dba)_3_/**L1** (TON: >100,000; TOF: 12,000 h^−1^; selectivity: >99%) catalytic systems reported so far^17,24^, thus providing more promising industrial application of this catalytic system.

### Investigation of the oxygen-resistance stability

**Fig. 3:**
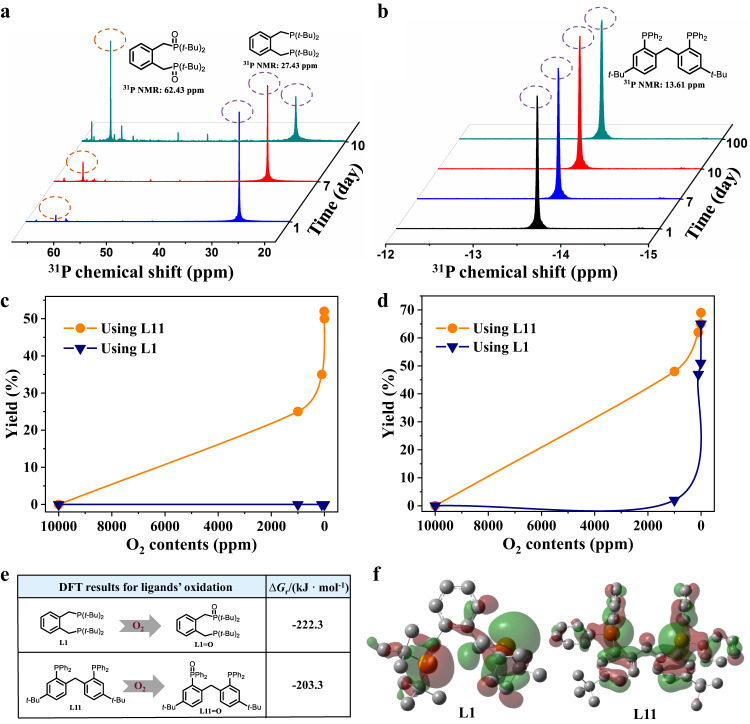
Comparison tests of oxygen-resistance stability between L1 and L11. **a**, **b** Stability tests of **L1** and **L11** under air conditions at room temperature, respectively. **c**, **d** Stability tests of **L1** and **L11** during the reaction process in 1,4-dioxane or methanol, respectively. **e** Change in Gibbs’ free energy (∆G_r_) for **L1** and **L11** oxidation, computed at DFT level. **f** HOMO orbitals of **L1** and **L11**.

The oxygen-resistance stability comparison between **L11** and **L1** was examined (Fig. 3). After being exposed under air at room temperature, the phosphine atom on **L1** was quickly oxidized after several days, whereas the pristine phosphine valence state of **L11** was still unchanged even after 100 days of exposure (Fig. 3a, b, and Supplementary Figs. 15 and 16). Afterwards, the methoxycarbonylation of 1-octene was conducted under different oxygen contents in presence of Pd/**L11** and Pd/**L1**, respectively. Notably, the activities were increased gradually as the oxygen content decreased from 10000 to 1 ppm when using Pd/**L11** system in dioxane, and 25% yield was achieved even at 1000 ppm of oxygen content while no yield was observed for Pd/**L1** system even at 1 ppm (Fig. 3c, and Supplementary Fig. 17). Because methanol was a common-used solvent in the state-of-the-art Pd/**L1** system, thus the oxygen-resistance stability was also tested in methanol. 48% yield was observed using Pd/**L11** in presence of 1000 ppm of oxygen content whereas only trace amount of the desired product was obtained using Pd/**L1** (Fig. 3d and Supplementary Fig. 18). Importantly, the reaction solution displayed transparent yellow after methoxycarbonylation catalyzed by Pd/**L11** in the case of 1,4-dioxane or methanol as a solvent, whereas palladium black particles were observed obviously using Pd/**L1** under identical reaction conditions (Supplementary Figs. 19 and 20).

Density functional theory (DFT) calculations revealed that the Gibbs’ free energy (∆G_r_) upon oxidation for **L11** was less negative than that for **L1** (−203.3 vs −222.3 kJ/mol, Fig. 3e), and the energy difference between HOMO orbital of **L11** and LUMO orbital of triplet oxygen was larger than that of **L1** (235.85 kJ/mol vs 222.48 kJ/mol), indicating **L11** was more resistant to oxidation than **L1**. Furthermore, the electron orbital in **L11** spanned larger range than that in **L1**, showing that the stronger electron delocalization of **L11** was the intrinsic factor resistant to the oxidation (Fig. 3f). These comparison results demonstrated that aryldiphosphine ligand **L11** possessed stronger oxygen-resistance stability for both storage and reaction, which was crucial for its practicability due to the possible reduction of industrial operating costs.

### Mechanism study

**Fig. 4:**
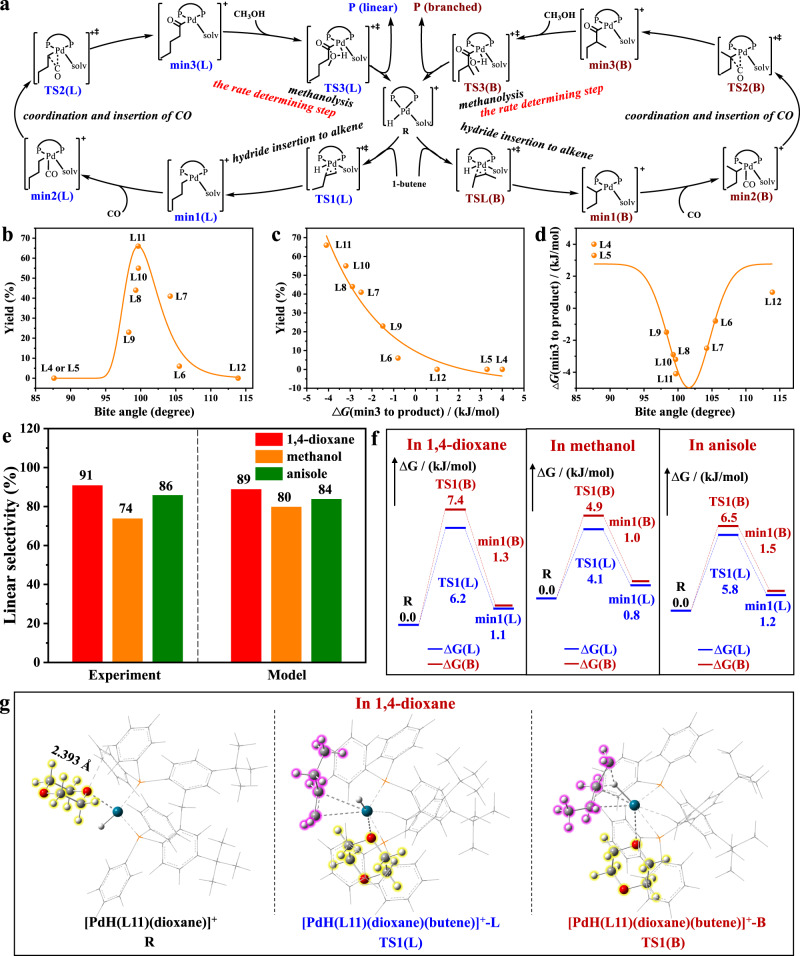
Investigation of the ligand bite angel and solvent effect. **a** Computed structure of R, TS, min, and P, and plausible mechanism. **b** Dependence between bite angles of P-Pd-P and yield of methoxycarbonylation of 1-octene. **c** Dependence between △G(min3 to product) and yield of methoxycarbonylation of 1-octene via various aryldiphosphine ligands. **d** Dependence between bite angles of P-Pd-P and △G(min3 to product). **e** Experimental tests and Boltzmann populations of the selected solvents on solvent effect. **f** Free energy profiles for the formation of linear (L; blue) and branched esters (B; red); See Fig. 4a for the computed structure of R, TS, min, and P. **g** DFT optimized structures of the initial complex with 1,4-dioxane (highlighted in yellow), [PdH(**L11**)(dioxane)]^+^, and upon hydride insertion to 1-butene (highlighted in pink), [PdH(**L11**)(dioxane)(butene)]^+^-L, [PdH(**L11**)(dioxane)(butene)]^+^-B; Legend of colors: White (H), Gray (C), Red (O), Orange (P), and Petrol Green (Pd).

The relationship between the bite angle of P-Pd-P and the catalytic activity was investigated (Fig. 4b). The yield of methoxycarbonylation of 1-octene improved rapidly as increasing the bite angle of P-Pd-P from 87.62° (**L4**) to 99.68° (**L11**), and then declined sharply with the ligand bite angle further increased from 99.68° (**L11**) to 113.90° (**L12**). The highest activity was obtained in presence of **L11**, indicating that appropriate bite angle of P-Pd-P around 100° was benificial for achieving the highest catalytic activity. Owing to the rate-limitation role of methanolysis of acyl palladium species (min3 to product) (Fig. 4a), the correlation between the activation energy of this elementary step and activity was examined (Fig. 4c)^25^. The yield of methoxycarbonylation of 1-octene slipped from 66% to 0% as the values of ∆G(min3 to product) increased from −4.1 to 4 kJ/mol. Subsequently, the dependence between bite angle of P-Pd-P and ∆G was explored where the minimal value of ∆G (−4.1 kJ/mol) was obtained in presence of **L11** (Fig. 4d). So, it could be concluded that aryldiphosphine ligand **L11** with appropriate bite angle of P-Pd-P was responsible for the optimal catalytic activity because of the lowest energy barrier of min3 to product.

When using 1,4-dioxane, methanol, and anisole in methoxycarbonylation of 1-octene, 91%, 74%, and 86% regioselectivities were obtained, respectively, while no activity was observed at all when reacting in acetonitrile and 1,2-dimethoxyethane (Fig. 4e and Supplementary Table 2). Then, DFT calculations were conducted to reveal the origin of regioselectivity. The overall pathway of alkoxycarbonylation of alkenes can be formally divided into three consecutive steps: (1) hydride insertion to alkene (R → TS1 → min1), (2) coordination and insertion of CO (min1 → min2 → TS2 → min3), and (3) methanolysis (min3 → TS3 → P) (Fig. 4a), where the hydride insertion to alkene was the key step in determining the regioselectivity^1,36^. The energy files for the reaction pathways of linear and branched products in different solvents were illustrated (Fig. 4f and Supplementary Fig. 21). Clearly, the differences of ∆G_a_ between TS1(B) and TS1(L) during the hydride insertion to alkene in 1,4-dioxane was 1.2 kJ/mol, higher than that in MeOH (0.8 kJ/mol) and anisole (0.7 kJ/mol), confirming that the linear reaction pathway in 1,4-dioxane was more kinetically favored. The relative ratios for the L/B in 1,4-dioxane methanol, and anisole were also calculated by Boltzmann population to be 89%, 80% and 84%, respectively, which matched well with the experimental data, further proving the hydride insertion to alkene determined the regioselectivity (Fig. 4e).

Under the optimal catalytic system, the coordination chemistry of 1,4-dioxane with Pd/**L11** was crucial for determining the regioselectivity. Compared with other solvents, a specific interaction between the oxygen lone-pair electrons of 1,4-dioxane and a hydrogen atom of the phenyl rings of **L11** was verified with a distance of 2.393 Å, leading to an in situ formed configuration of [PdH(**L11**)(1,4-dioxane)]^+^(R) (Fig. 4g). This interaction was the reason leading to the highest ∆G_a_ difference (1.2 kJ/mol) between TS1(B) and TS1(L) during the hydride insertion to 1-butene, indicating the preferable formation of the TS1(L). The highest ∆G_a_ difference probably arisen from the steric hindrance of TS1(B) configuration, leading to the high regioselectivity towards linear product. However, ∆G_a_ of the hydride insertion to alkene for acetonitrile or 1,2-dimethoxyethane was too high to initiate the catalytic cycle (Supplementary Fig. 21), suggesting their reaction paths were kinetically hindered. Consequently, the specific 1,4-dioxane effect benefited the linear reaction pathway, thus enhancing the selectivity towards the linear esters^37–39^.

Subsequently, various control experiments were conducted to gain mechanistic insight into the reaction. The crude solution after 0.5 h of methoxycarbonylation of 1-octene was investigated and subjected to run the high-resolution mass spectrometry analysis. An assignable peak located at m/z = 853.2495 was observed, which was attributed to Intermediate A (Supplementary Fig. 22; chemical formula: C_50_H_53_O_2_P_2_Pd^+^, exact mass: 853.2550). This result indicated that the formed Intermediate A might be involved in the proposed catalytic cycle. Furthermore, in situ generated Pd-P intermediates were also detected in methoxycarbonylation of 1-octene by ^31^P NMR measurements (Supplementary Figs. 23 and 24). Remarkably, chemical shifts of the Pd-P intermediates before and after reaction changed from 24.05 to 20.90 ppm, attributing to in situ generated [PdH(**L11**)(1,4-dioxane)]^+^(R)^40^.

In situ high-pressure FTIR analysis was conducted to monitor the dynamic catalytic behavior of the Pd/**L11** and other intermediates during the reaction process (Supplementary Fig. 25). With the reaction temperature increasing from 30 to 100 °C, characteristic peaks of the Pd-H (palladium–hydride) and Pd-P (palladium–phosphine) species, both of which were identified as the key active intermediates that made the catalytic reaction proceeding smoothly, appeared immediately at 1929 and 970 cm^−1^, respectively, and then reached its maximum intensities at about 100 °C (Supplementary Fig. 25b, c)^41^. The peaks at 2017, 1988, and 1971 cm^−1^ were assigned to vibrations of Pd-CO that were the complexation results of various Pd/**L11** intermediates with CO (Supplementary Fig. 25b)^42,43^. Furthermore, two peaks at 3078 and 1640 cm^−1^ were ascribed to the stretching vibration of =C-H and -C=C- on 1-octene, and these signal intensities became weaken with the reaction temperature rising (Supplementary Fig. 25a, b); meanwhile, the signal intensity of stretching vibration of -C=O- of the target ester located at 1739 cm^−1^ was intensified, which demonstrated the consumption of 1-octene and the generation of methyl nonanoate as the reaction process goes on. These mechanism studies confirmed that the Pd-P and Pd-H intermediates were active species during the catalytic process and formation of which were facilitated in the presence of **L11**.

### Scope of the methodology

**Fig. 5:**
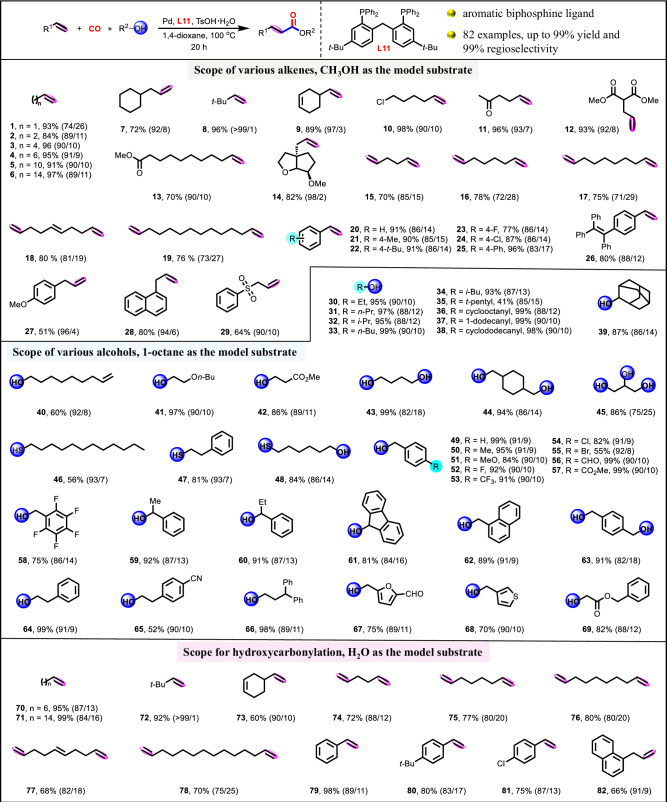
Substrate scope of common alkenes and alcohols. Detailed experimental procedures and reaction parameters for the various substrates were included in Supplementary Information.

Substrate compatibility of common alkenes and alcohols for alkoxycarbonylation or hydroxycarbonylation was tested (Fig. 5). Both short- (**1**–**3**) and long-chain (**4**–**6**) aliphatic alkenes were converted to the desired linear esters smoothly, affording excellent yields (84–97%) and regioselectivities (74–91%). Aliphatic alkenes with bulky steric hindrance groups (**7** and **8**) were well tolerated with excellent regioselectivities (92-99%) because the large steric hindrance groups inhibited the branched step of hydride insertion to alkene. Notably, alkene (**9**) bearing both internal and terminal C=C bonds reacted selectively at the position of terminal C=C bond, obtaining 89% yield and 97% regioselectivity. Moreover, aliphatic alkenes with chlorine (**10**), ketone (**11**), and ester (**12** and **13**), and chiral aliphatic alkene (**14**) were equally applicable. Importantly, diesters **15** to **19**, which has potential applications in polymer chemistry, were also directly synthesized with up to 80% yield and 85% regioselectivity through methoxycarbonylation of terminal dienes. Furthermore, various aryl alkenes (**20**–**26**), allylbenzenes (**27**–**28**), and allyl phenyl sulfone (**29**) were also employed and afforded the desired esters in good to excellent yields (51–96%) and regioselectivities (83-96%).

Primary (**30,** **31,** **33**, and **37**) and secondary (**32,** **34,** **36,** **38** and **39**) aliphatic alcohols were converted to the corresponding esters in nearly quantitative yields and excellent regioselectivities. Comparatively, tertiary aliphatic alcohol afforded the target ester (**35**) with lower yield (41%) and regioselectivity (85%) because the hydroxycarbonylation of 1-octene was occurred due to the formation of H_2_O caused by the dehydration of tertiary amyl alcohol. Besides, aliphatic alcohols (**40**–**42**) bearing terminal alkene, ether, or ester group were converted smoothly to give linear esters with up to 97% yield and 92% regioselectivity. Gratifyingly, this catalytic system also allowed efficient multi-alkoxycarbonylations of polyhydric alcohols for direct synthesis of plasticizers and polyol ester base oil^44–46^, producing the corresponding polyesters (**43**–**45**) with excellent yields (86–99%) and regioselectivities (75–86%). Thiocarbonylations of thiols were also realized to yield the corresponding thioesters (**46**–**48**) with up to 84% yield and 93% regioselectivity. Subsequently, versatile benzyl alcohol derivatives (**49**–**63**) were investigated to the afford desired esters with up to 99% yield and 92% regioselectivity. Other aryl-substituted and heteroaromatic alcohols were also well tolerated, affording up to 99% yield and 91% regioselectivity (**64**–**69**).

Carboxylic acids which are widely applied in the synthesis of polymers, cosmetics, pharmaceuticals^47–51^, were also produced by hydroxycarbonylation of alkenes. Both aliphatic (**70** to **78**) and aromatic (**79** to **82**) carboxylic acids were generated with up to 99% yield and 99% linear regioselectivity. Notably, linear dicarboxylic acids (**73** to **77**), as desirable synthon in the manufacture of polymers (such as Nylon PACM-12), fragrances, lubricants^52,53^, were directly synthesized with good yields (68–80%) and regioselectivities (75–88%) via hydroxycarbonylation of terminal dienes, thus proving the wide practicality of this catalytic system.

**Fig. 6:**
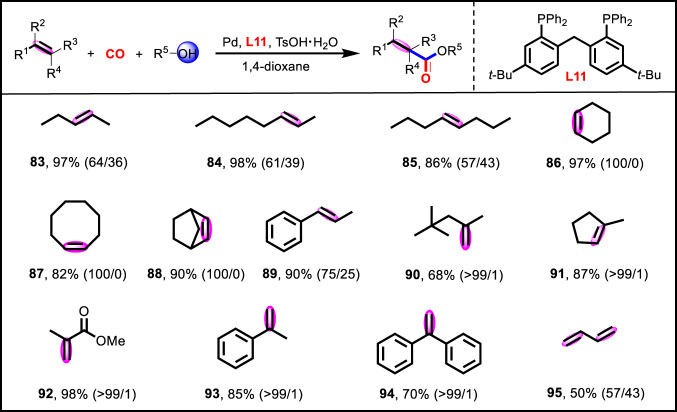
Substrate scope of internal and branched alkenes, as well as 1, 3-butadiene. Detailed experimental procedures and reaction parameters for the various substrates were included in Supplementary Information.

Next, methoxycarbonylation of internal alkenes were also conducted catalyzed by the presented Pd/**L11** catalytic system (Fig. 6). For aliphatic internal alkenes (**83**–**85**), remarkable activity (86–98%) and moderate linear regioselectivity (57-64%) of the desired products were achieved. Meanwhile, cyclic internal alkenes, such as cyclohexene (**86**), cycloctene (**87**), and norbornene (**88**), reacted smoothly to yield the corresponding esters with up to 97% yield and 100% selectivity. Aromatic internal alkene of β-methylstyrene (**89**) could also afford the target ester with 90% yield and 75% linear regioselectivity. Furthermore, aliphatic and aromatic branched alkenes (**90**–**94**) were also employed and afforded the corresponding linear esters in good to excellent yields (68–98%) and outstanding regioselectivities (>99%). Notably, methoxycarbonylation of industrially important 1,3-butadiene (**95**) could also proceed well, giving moderate reactivity and regioselecyivity toward the dimethyl adipate.

**Fig. 7:**
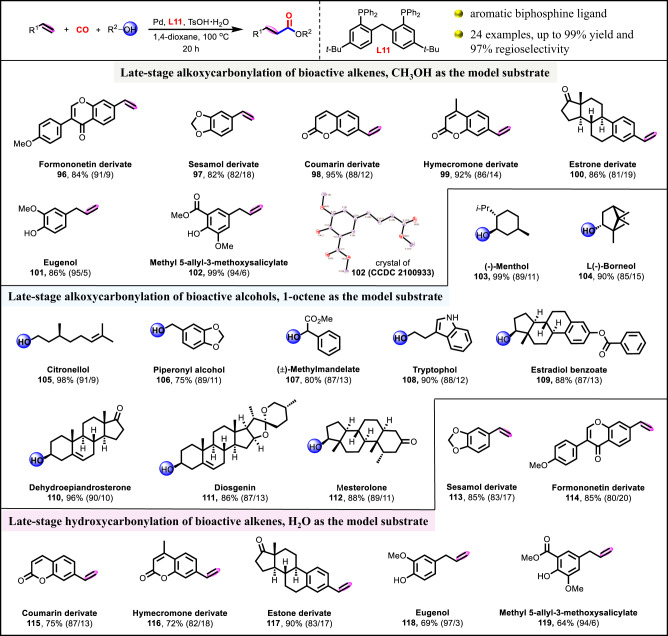
Late-stage functionalization of bioactive alkenes and alcohols. Detailed experimental procedures and reaction parameters for the various substrates were included in Supplementary Information.

The late-stage modification of lead compounds or even actual drugs is of current interest for the discovery of new bioactive agents^54,55^. To validate the practicality of this methodology, the late-stage functionalized bioactive or chiral alkenes and alcohols for alkoxycarbonylation or hydroxycarbonylation were investigated (Fig. 7). Alkenes derived from drugs or natural products, including formononetin (**96**), sesamol (**97**), coumarin (**98**), hymecromone (**99**) and estrone (**100**) functionalities were successfully converted to the corresponding esters in high efficiency (82–95% yields and 81–91% regioselectivities). The biomass-based alkenes, eugenol (**101**), and methyl 5-allyl-3-methoxysalicylate (**102**) were also eligible candidates as well. Moreover, bioactive alcohols, including (-)-menthol (**103**), L(-)-borneol (**104**), citronellol (**105**), piperonyl alcohol (**106**), (±)-methylmandelate (**107**), tryptophol (**108**), estradiol benzoate (**109**), dehydroepiandrosterone (**110**), diosgenin (**111**), and mesterolone (**112**), were employed to furnish the desired esters with 75–99% yields and 85–91% regioselectivities. Notably, hydroxycarbonylation of these alkenes from bioactive or chiral derivatives (**113**–**119**) generated the desire acids with up to 90% yield and 97% regioselectivity, further highlighting the potential synthetic utility of this protocol^56^.

**Fig. 8:**
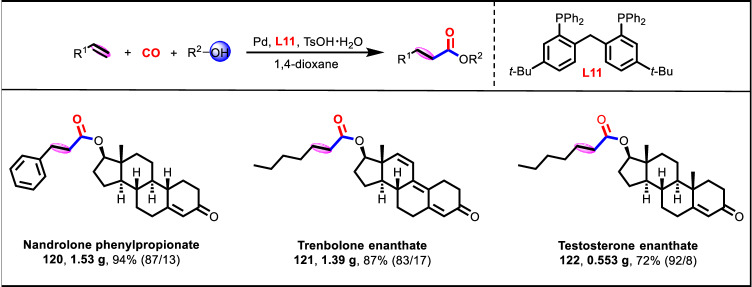
Gram-scale synthesis of clinically important pharmaceutical molecules. Detailed experimental procedures and reaction parameters for the various substrates were included in Supplementary Information.

Nandrolone phenylpropionate (**120**), trenbolone enanthate (**121**), and testosterone enanthate (**122**) were a class of anabolic hormone approved by FDA (Food and Drug Administration), which were commonly-used in drug therapy for the palliative treatment of women with advanced breast cancer, the ideal contraceptive of male, and the increase of muscle strength and circumference, respectively. Gratifyingly, gram-scale synthesis of these clinically important pharmaceutical molecules were realized directly by the alkoxycarbonylation of the corresponding alkenes and alcohols under this potent Pd/**L11** catalytic system, exhibiting excellent reaction performance (**120**: 1.53 g, 94% yield, 87% linear selectivity; **121**: 1.39 g, 87% yield, 83% linear selectivity; **122**: 0.553 g, 72% yield, 92% linear selectivity) (Fig. 8). This application further validated the potential practicability of this protocol for the synthesis of pharmaceutical molecules.

## Discussion

In summary, a stable and efficient aryldiphosphine ligand with [Ph_2_P(ortho-C_6_H_4_)]_2_CH_2_ skeleton for palladium-catalyzed regioselective carbonylation of alkenes was developed. With the optimal ligand **L11**, higher catalytic efficiency was obtained on methoxycarbonylation of ethylene (TON: >2,390,000; TOF: 100,000 h^−1^; selectivity: >99%), which represented excellent catalytic performance so far and possessed stronger oxygen-resistance stability compared with the industrially successful Pd/DTBPMB catalyst. Various alkenes and alcohols were well tolerated and selectively transformed to the desired linear esters or acids with up to 99% yield and 99% regioselectivity. Importantly, the late-stage carbonylation of bioactive and chiral alkenes or alcohols were successfully achieved with high activity and regioselectivity; gram-scale synthesis of clinically important pharmaceutical molecules, nandrolone phenylpropionate, trenbolone enanthate, and testosterone enanthate were also realized via this protocol. Experimental and computational investigations indicated that the appropriate P-Pd-P bite angle of the Pd/**L11** and the favorable 1,4-dioxane effect with the Pd/**L11** synergistically improved the catalytic activity and regioselectivity. Moreover, electron deficient phosphines on aryldiphosphine ligand caused by the electron delocalization were responsible for better oxygen-resistance stability.

## Methods

### General information

All reactions involving air and water-sensitive materials were performed under argon atmosphere. All chemicals were purchased from Innochem, Energy Chemical, J&K Scientific, Sigma-Aldrich, Acros Organics and used without further purification unless otherwise noted. Analytical thin layer chromatography (TLC) was performed using pre-coated Jiangyou silica gel HSGF254 (0.2 mm ± 0.03 mm). Visualization of the developed chromatogram was performed by UV absorbance (254 nm) or TLC stains (KMnO_4_ solution). Flash chromatography was performed using silica gel 60, 0.063–0.2 mm, 200–300 mesh (Jiangyou, Yantai) with the indicated solvent system. GC-MS analyses were performed with on an Agilent 5977 A MSD GC-MS. Gas chromatography analyses were performed on an Agilent 7890 A GC equipped with a HP-5 capillary column and FID detector. Nuclear magnetic resonance spectra were recorded on a Bruker AvanceTM III 400 MHz in deuterated chloroform unless otherwise noted. Data are reported in parts per million (ppm) as follows: chemical shift, multiplicity (s = singlet, d = doublet, t = triplet, q = quartet, quint = quintet, m = multiplet, dd = doublet of doublet and br = broad signal), coupling constant in Hz and integration. The high-resolution mass spectrometry was recorded on a Bruker Q-TOF II MSD. X-ray crystallographic analyses were performed on a Bruker Smart APEX II, all the crystal structure diagrams were drawn by the Ortep-3 program^57^. HPLC analysis for measuring values of linear/branched products was performed on a Waters-Breeze system (2487 Dual λ Absorbance Detector and 1525 Binary HPLC Pump). Chiralpak AD-H columns were purchased from Daicel Chemical Industries, LTD. All measurements were carried out at room temperature unless otherwise stated. In situ high-pressure FT-IR spectra were obtained using a Nicolet NEXUS 670 spectrometer, the spectral resolution was about 4 cm^−1^.

### General procedure for alkoxycarbonylation or hydroxycarbonylation

An 80 ml steel autoclave was charged with Pd(acac)_2_ (1.2 mg, 3.94 μmol, 0.2 mol%), **L11** (10.4 mg, 16.05 μmol, 0.8 mol%), TsOH·H_2_O (7.6 mg, 40.00 μmol, 2 mol%), and 1,4-dioxane (4 ml). Then alkenes (8 mmol) and alcohols or H_2_O (36 mg, 2 mmol) were introduced into the autoclave. After the autoclave was purged with CO (1 MPa) for four times at room temperature and then pressurized with CO to 2 MPa, the autoclave was sealed and put into a preheated reactor, stirring at 100 °C for 20 h. Afterwards, the autoclave was cooled to room temperature and depressurized slowly. Subsequently, the reaction mixture was diluted with EtOAc (6.0 ml). Finally, the total yields of linear and branched esters or acids were obtained by gas chromatography analysis, flash column chromatography on silica gel, or acid-base extraction, and the regioselectivities of linear and branched esters or acids were obtained by gas chromatography analysis, liquid chromatography analysis, or ^1^H NMR analysis. Detailed experimental procedures and reaction parameters for the various substrates were included in Supplementary Information.

### Synthetic procedure of L11

A solution of *n*-butyllithium in hexane (2.5 M, 8.8 ml, 22 mmol) was added dropwise over a period of 10 min to a solution of bis(2-bromo-4-(tert-butyl)phenyl)methane (4.38 g, 10 mmol) in anhydrous tetrahydrofuran (60 ml) at −78 °C under argon atmosphere. The solution was stirred for 1 h and then chlorodiphenylphosphine (4.84 g, 22 mmol) dissolved in anhydrous tetrahydrofuran (5 ml) was added dropwise. The mixture was continued to stir at −78 °C for 1 h and the system was heated to room temperature, and allowed to react overnight. The reaction was quenched with 2 N HCl solution. The mixture was extracted with ethyl acetate and water for 3 times, the combined organic phases were dried over anhydrous Na_2_SO_4_, and concentrated under reduced pressure. The residue was purified by flash column chromatography on silica gel using EtOAc–petroleum ether mixture (1:50) as an eluent to afford the desired compound **L11** as a white solid (4.2 g, 65% yield). Caution: The usage of *n*-butyllithium in hexane should be careful and protected by inert atmosphere owing to its easy flammability under air or moisture condition. Detailed synthetic procedures and reaction parameters for the other ligands were included in Supplementary Information.

### Supplementary information

Supplementary Information

Peer Review File

## Data Availability

All data generated and analyzed during this study are included in this Article and its Supplementary Information. All data are available from the corresponding author upon request.

## References

[1] Li H (2016). The scope and mechanism of palladium-catalysed markovnikov alkoxycarbonylation of alkenes. Nat. Chem..

[2] An J (2018). Acid-promoter-free ethylene methoxycarbonylation over ru-clusters/ceria: The catalysis of interfacial lewis acid-base pair. J. Am. Chem. Soc..

[3] Yang J (2019). Direct synthesis of adipic acid esters via palladium-catalyzed carbonylation of 1,3-dienes. Science.

[4] Hood DM (2020). Highly active cationic cobalt(ii) hydroformylation catalysts. Science.

[5] Folster CP (2021). Development and applications of selective hydroesterification reactions. Trends Chem..

[6] Morrill LC, Smith AD (2014). Organocatalytic lewis base functionalisation of carboxylic acids, esters and anhydrides via c1-ammonium or azolium enolates. Chem. Soc. Rev..

[7] Ashfaq M (2014). Synthetic thioamide, benzimidazole, quinolone and derivatives with carboxylic acid and ester moieties: A strategy in the design of antituberculosis agents. Curr. Med. Chem..

[8] Wang X (2018). Single-site ruthenium pincer complex knitted into porous organic polymers for dehydrogenation of formic acid. Chemsuschem.

[9] Liu M (2018). Transformation of alcohols to esters promoted by hydrogen bonds using oxygen as the oxidant under metal-free conditions. Sci. Adv..

[10] Kalck P, Urrutigoity M (2015). Recent improvements in the alkoxycarbonylation reaction catalyzed by transition metal complexes. Inorg. Chim. Acta.

[11] Sang R (2020). State-of-the-art palladium-catalyzed alkoxycarbonylations. Org. Chem. Front..

[12] Ai H-J, Lu W, Wu X-F (2021). Ligand-controlled regiodivergent thiocarbonylation of alkynes toward linear and branched alpha,beta-unsaturated thioesters. Angew. Chem. Int. Ed..

[13] Ai H-J (2021). Palladium-catalyzed thiocarbonylation of alkenes toward linear thioesters. ACS Catal..

[14] Ren X (2021). Asymmetric alkoxy- and hydroxy-carbonylations of functionalized alkenes assisted by beta-carbonyl groups. Angew. Chem. Int. Ed..

[15] Tooze, R. P., et al., inventors; Imperial Chemical Industries Plc, assignee. Carbonylation of ethylene and stable catalyst system containing bidentate phosphine compounds for patent WO9619434. (1996).

[16] Pearson, J. M. & Hadden, R. A., inventors; Imperial Chemical Industries PLC, Pearson, Jean Margaret, Hadden, Raymond Anthony, assignee. Process and palladium-bidentate phosphine ligand catalysts for the carbonylation of ethylene into propionic acid and its esters patent WO9841495. (1998).

[17] Clegg W (1999). Highly active and selective catalysts for the production of methyl propanoate via the methoxycarbonylation of ethene. Chem. Commun..

[18] Jimenez Rodriguez C (2004). Highly selective formation of linear esters from terminal and internal alkenes catalysed by palladium complexes of bis-(di-tert-butylphosphinomethyl)benzene. Chem. Commun..

[19] Vondran J (2021). Magic of alpha: The chemistry of a remarkable bidentate phosphine, 1,2-bis(di-tert-butylphosphinomethyl)benzene. Chem. Rev..

[20] Knight JG (2000). Remarkable differences in catalyst activity and selectivity for the production of methyl propanoate versus co-ethylene copolymer by a series of palladium complexes of related c_4_-bridged diphosphines. Organometallics.

[21] Pugh RI, Drent E (2002). Methoxycarbonylation versus hydroacylation of ethene; dramatic influence of the ligand in cationic palladium catalysis. Adv. Synth. Catal..

[22] Pugh, R. I., Drent, E. & Pringle, P. G. Tandem isomerisation-carbonylation catalysis: Highly active palladium(ii) catalysts for the selective methoxycarbonylation of internal alkenes to linear esters. *Chem. Commun.* **37**, 1476–1477 (2001).

[23] Dong K (2017). Efficient palladium-catalyzed alkoxycarbonylation of bulk industrial olefins using ferrocenyl phosphine ligands. Angew. Chem. Int. Ed..

[24] Dong K (2017). Highly active and efficient catalysts for alkoxycarbonylation of alkenes. Nat. Commun..

[25] Dong K (2018). Cooperative catalytic methoxycarbonylation of alkenes: Uncovering the role of palladium complexes with hemilabile ligands. Chem. Sci..

[26] van Leeuwen P (2003). Alcoholysis of acylpalladium(ii) complexes relevant to the alternating copolymerization of ethene and carbon monoxide and the alkoxycarbonylation of alkenes: The importance of cis-coordinating phosphines. J. Am. Chem. Soc..

[27] Labrue F (2005). Synthesis and x-ray crystal structure of 2-(phosphinomethyl)ferrocenyl diphenylphosphine. J. Organomet. Chem..

[28] Lindahl SE (2013). Utilizing redox-mediated bergman cyclization toward the development of dual-action metalloenediyne therapeutics. J. Am. Chem. Soc..

[29] Kamer PCJ, van Leeuwen PWN, Reek JNH (2001). Wide bite angle diphosphines: Xantphos ligands in transition metal complexes and catalysis. Acc. Chem. Res..

[30] Birkholz MN, Freixa Z, van Leeuwen PWNM (2009). Bite angle effects of diphosphines in c-c and c-x bond forming cross coupling reactions. Chem. Soc. Rev..

[31] Fanjul, T., et al. Interplay of bite angle and cone angle effects. A comparison between o-c6h4(ch2pr2)(pr‘2) and o-c6h4(ch2pr2)(ch2pr‘2) as ligands for pd-catalysed ethene hydromethoxycarbonylation. *Dalton Trans.* **42**, 100–115 (2013). 10.1039/c2dt31913f 23080322

[32] Burrows AD (2010). Solid state interconversion of cages and coordination networks via conformational change of a semi-rigid ligand. Chem. Commun..

[33] Xu Z-X (2015). Integration of a semi-rigid proline ligand and 4,4 ‘-bipyridine in the synthesis of homochiral metal-organic frameworks with helices. Dalton Trans..

[34] Ali U, Abd Karim KJB, Buang NA (2015). A review of the properties and applications of poly (methyl methacrylate) (pmma). Polym. Rev..

[35] Mahboub MJD (2018). Catalysis for the synthesis of methacrylic acid and methyl methacrylate. Chem. Soc. Rev..

[36] Gao P (2021). Phosphorus coordinated rh single-atom sites on nanodiamond as highly regioselective catalyst for hydroformylation of olefins. Nat. Commun..

[37] Davies DL (2015). Experimental and dft studies explain solvent control of c–h activation and product selectivity in the rh(iii)-catalyzed formation of neutral and cationic heterocycles. J. Am. Chem. Soc..

[38] Cheng G (2021). Critical role of solvent-modulated hydrogen-binding strength in the catalytic hydrogenation of benzaldehyde on palladium. Nat. Catal..

[39] Yang J (2021). Efficient palladium-catalyzed carbonylation of 1,3-dienes: Selective synthesis of adipates and other aliphatic diesters. Angew. Chem. Int. Ed..

[40] Lesueur W (1997). A bidentate bisphosphine functioning in intramolecular aliphatic metalation and as an nmr spectroscopic probe for the metal coordination environment. Inorg. Chem..

[41] Yang D (2019). Novel multi-dentate phosphines for pd-catalyzed alkoxycarbonylation of alkynes promoted by h2o additive. J. Catal..

[42] Diebolt O, van Leeuwen PWNM, Kamer PCJ (2012). Operando spectroscopy in catalytic carbonylation reactions. ACS Catal..

[43] Yang D (2018). Co-catalysis over a bi-functional ligand-based pd-catalyst for tandem bis-alkoxycarbonylation of terminal alkynes. Green. Chem..

[44] Hu Y (2022). Efficient synthesis of novel plasticizers by direct palladium-catalyzed di- or multi-carbonylations. Angew. Chem. Int. Ed..

[45] Gao X (2015). A quantitative structure tribo-ability relationship model for ester lubricant base oils. J. Tribol..

[46] Sang R (2018). Palladium-catalyzed selective generation of co from formic acid for carbonylation of alkenes. J. Am. Chem. Soc..

[47] Moret S, Dyson PJ, Laurenczy G (2014). Direct synthesis of formic acid from carbon dioxide by hydrogenation in acidic media. Nat. Commun..

[48] Korstanje TJ (2015). Hydrogenation of carboxylic acids with a homogeneous cobalt catalyst. Science.

[49] Hu Y (2017). Sustainable production of pyromellitic acid with pinacol and diethyl maleate. Green. Chem..

[50] Song S (2020). Visible-light-driven amino acids production from biomass-based feedstocks over ultrathin cds nanosheets. Nat. Commun..

[51] Li Z, Wen X, Liu H (2022). Efficient conversion of bio-renewable citric acid to high-value carboxylic acids on stable solid catalysts. Green. Chem..

[52] McKeen, L. W. 8 - polyamides (nylons). In: *Film properties of plastics and elastomers*, 3rd ed. (ed. McKeen, L. W.) 157–188 (William Andrew Publishing, 2012).

[53] Abraham, T. W.& Höfer, R. 10.03 - lipid-based polymer building blocks and polymers. In: *Polymer science: A comprehensive reference* (eds. Matyjaszewski, K. & Möller, M.) 15–58 (Elsevier, 2012).

[54] Sharma A, Hartwig JF (2015). Metal-catalysed azidation of tertiary c–h bonds suitable for late-stage functionalization. Nature.

[55] Cernak T (2016). The medicinal chemist’s toolbox for late stage functionalization of drug-like molecules. Chem. Soc. Rev..

[56] Sang R (2019). Synthesis of carboxylic acids by palladium-catalyzed hydroxycarbonylation. Angew. Chem. Int. Ed..

[57] Farrugia LJ (2012). Wingx and ortep for windows: An update. J. Appl. Crystallogr..

