# Supplementary material for: Identification of a potent palladium-aryldiphosphine catalytic system for high-performance carbonylation of alkenes
Source: Nat Commun. 2024 Mar 5;15:2016. doi: 10.1038/s41467-024-46286-9 (PMC10914764; doi:10.1038/s41467-024-46286-9)
Supplement: Supplementary file 1 — Supplementary Information [file 41467_2024_46286_MOESM1_ESM.pdf]

Supplementary Information for

Identification of a potent palladium-aryldiphosphine catalytic system for high-performance carbonylation of alkenes

Kang Zhao[1,2,†], Hongli Wang[1,†], Teng Li[1], Shujuan Liu[1], Enrico Benassi[3]*, Xiao Li[4], Yao Yao[4], Xiaojun Wang[4], Xinjiang Cui[1]*, and Feng Shi[1]*

[1] State Key Laboratory for Oxo Synthesis and Selective Oxidation, Lanzhou Institute of Chemical Physics, Chinese Academy of Sciences; No. 18, Lanzhou, People's Republic of China.

[2] University of Chinese Academy of Sciences, No. 19A, Beijing, People's Republic of China.

[3] Novosibirsk State University, No. 2, Pigorova ul., Novosibirsk, Russian Federation.

[4] Nanjing Chengzhi Clean Energy Co., LTD., Nanjing, People's Republic of China.

[†] These authors contributed equally: Kang Zhao, Hongli Wang.

*e-mail: enrico.benassi@unimore.it, xinjiangcui@licp.cas.cn, fshi@licp.cas.cn

This PDF file includes:

Supplementary Note 1: Synthetic procedure of alkenes and ligands................................. 2
Supplementary Note 2: Optimization of the reaction conditions..................................... 24
Supplementary Note 3: Detailed procedure for alkoxycarbonylation or hydroxycarbonylation 30
Supplementary Note 4: NMR data of products.. 34
Supplementary Note 5: Upscaling reaction for TON and TOF experiments.................... 55
Supplementary Note 6: Comparison of oxygen-resistance stability between L1 and L11. 57
Supplementary Note 7: Computational investigations 62
Supplementary Note 8: Mechanistic studies... 65
Supplementary Note 9: Copies of GC, LC and NMR spectra 69
Supplementary Note 10: References.. 207

Supplementary Note 1: Synthetic procedure of alkenes and ligands

Synthetic procedure of alkenes 96-100

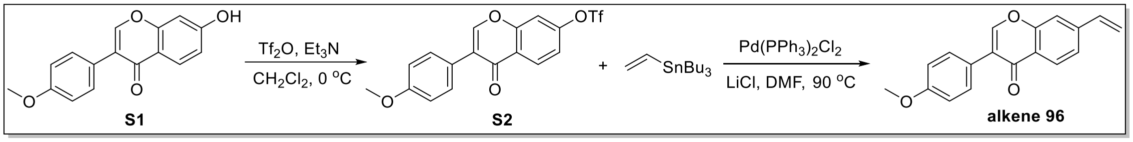

To a solution of **S1** (2.68 g, 10 mmol) and Et_3N (2.02 g, 20 mmol) in DCM (50 mL) was added Tf_2O (3.10 g, 11 mmol) at 0 °C under Argon, then the mixture was warmed up to room temperature and stirred for another 2 h. The reaction was quenched with saturated NH_4Cl and extracted with DCM. The combined organic layers were dried over Na_2SO_4, filtered and concentrated in vacuum. The residue was purified by flash column chromatography on silica gel using dichloromethane–petroleum ether mixture (2:1) as an eluent to afford the desired compound **S2** as a white solid (2.76 g, 69% yield).[1]

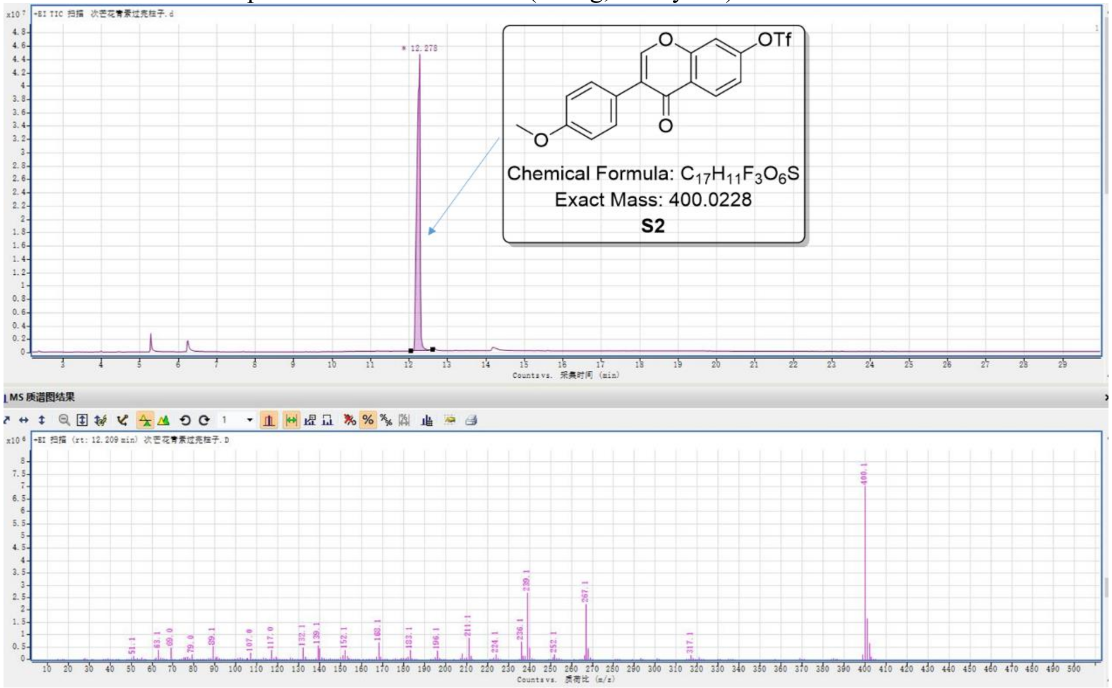

Supplementary Fig. 1 GC-MS spectrum of S2.

To a solution of **S2** (1.20 g, 3 mmol), $Pd(PPh_3)_2Cl_2$ (210.6 mg, 0.3 mmol) and LiCl (127.2 mg, 3 mol) in DMF (18 mL) was added tributyl(vinyl)stannane (1.05 g, 3.3 mmol) dropwise under Argon. The mixture was heated at 90 °C for 4 h. After completion the reaction mixture was cooled down to room temperature and diluted with DCM and washed with 1 M HCI (aq.), then washed with saturated $NaHCO_3$(aq.) and brine. The combined aqueous layers were extracted once with DCM. The combined organic layers were dried over Na_2SO_4, filtered and concentrated in vacuum. The residue was purified by flash column chromatography on silica gel using petroleum ether-dichloromethane-ethyl acetate mixture (6:1:1) as an eluent to afford the desired **alkene 96** as a white solid (637.7 mg, 76% yield).[1]

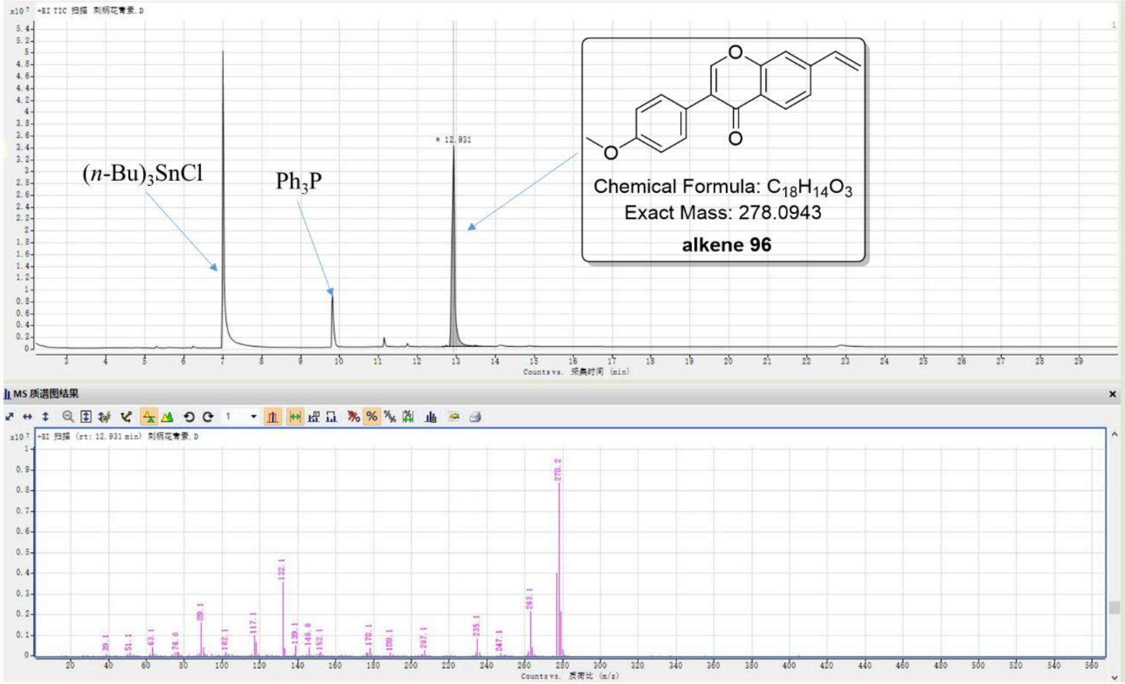

Supplementary Fig. 2 GC-MS spectrum of alkene 96.

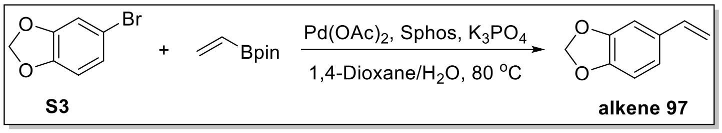

To an oven-dried microwave vial was added **S3** (1.0 g, 5 mmol), vinyl boronic acid, pinacol ester (846 mg, 5.5 mmol), $Pd(OAc)_2$ (460 mg, 0.2 mmol), SPhos (164 mg, 0.4 mmol), and K_3PO_4 (3.2 g, 15 mmol). The vial was capped and purged with nitrogen before the addition of 1,4-dioxane (20 mL) and H_2O (0.5 mL). The reaction mixture was heated at 80 °C for 1 h. The vial was then decapped, diluted with EtOAc and passed through a layer of Celite. The resultant solution was concentrated under vacuum and diluted with EtOAc. The organics were washed with H_2O followed by brine and the organic phases were collected. The organic phase was dried over Na_2SO_4, filtered, and concentrated under vacuum. The residue was purified by flash column chromatography on silica gel using petroleum ether as an eluent to afford the desired **alkene 97** as a colorless oil (673.4 mg, 91% yield).[2]

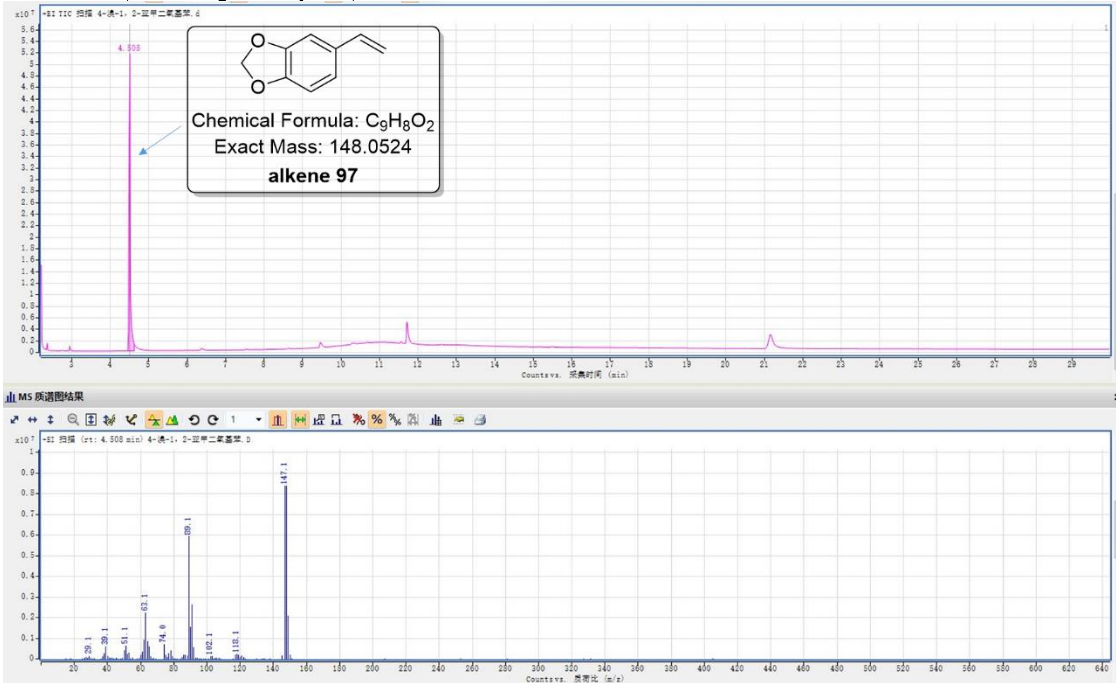

Supplementary Fig. 3 GC-MS spectrum of alkene 97.

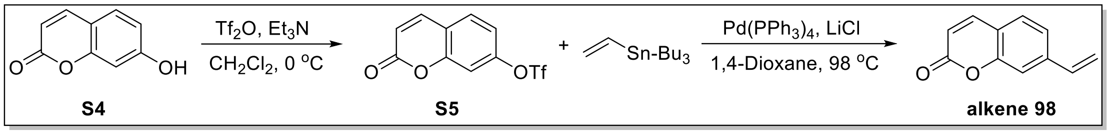

To a solution of **S4** (1.62 g, 10 mmol) and Et_3N (2.02 g, 20 mmol) in DCM (50 mL) was added Tf_2O (3.10 g, 11 mmol) at 0 °C under Argon, then the mixture was warmed up to room temperature and stirred for another 2 h. The reaction was quenched with saturated NH_4Cl and extracted with DCM. The combined organic layers were dried over Na_2SO_4, filtered and concentrated in vacuum. The residue was purified by flash column chromatography on silica gel using dichloromethane–petroleum ether mixture (2:1) as an eluent to afford the desired compound **S5** as a white solid (1.76 g, 60% yield).[1]

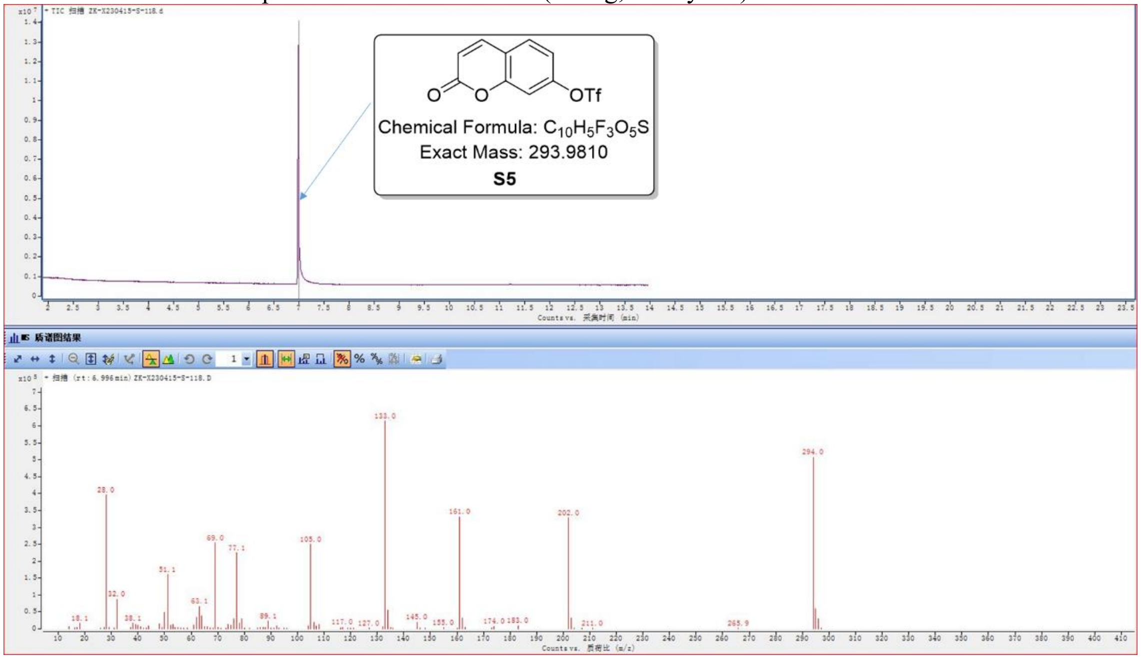

Supplementary Fig. 4 GC-MS spectrum of S5.

To a solution of **S5** (1.47 g, 5 mmol) in 14 mL of 1,4-dioxane were added tri-n-butylethenylstannane (1.46 mL, 5 mmol), LiCl (0.59 g, 14 mmol), $Pd(PPh_3)_4$ (139 mg, 0.1 mmol), and a few crystals of 2,6-di-tert-butyl-4-methyl-phenol. The resulting suspension was heated to 98 ^{o}C for 4 h, then cooled to room temperature. The mixture was diluted with EtOAc, filtered through a small pad of Celite, and washed with water, 1M HCl solution, saturated $NaHCO_3$ aqueous solution, and a concentrated sodium chloride solution. The solution was dried over anhydrous Na_2SO_4 and concentrated in vacuum. The residue was purified by flash column chromatography on silica gel using EtOAc–petroleum ether mixture (1:4) as an eluent to afford the desired **alkene 98** as a white solid (584 mg, 68% yield).[3]

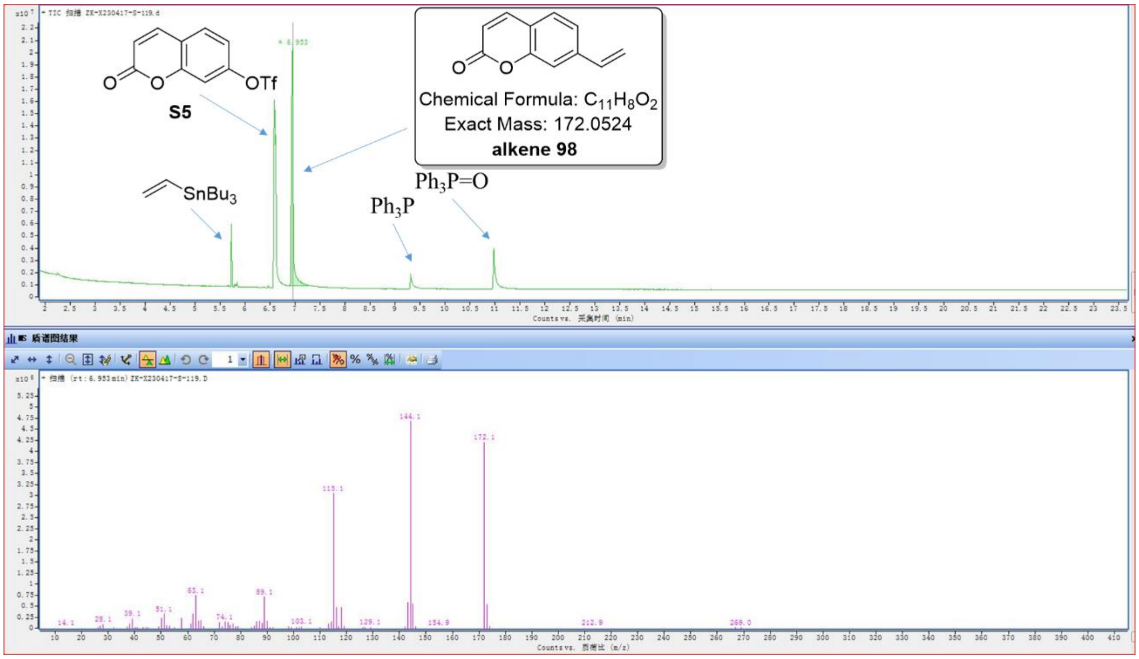

Supplementary Fig. 5 GC-MS spectrum of alkene 98.

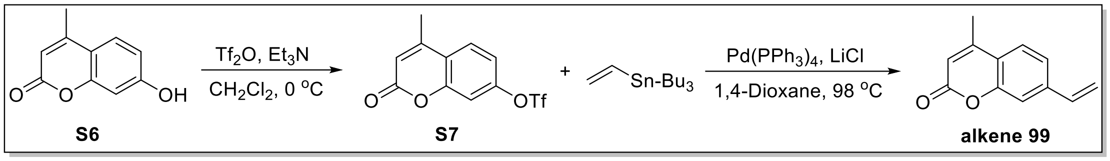

To a solution of **S6** (1.76 g, 10 mmol) and Et_3N (2.02 g, 20 mmol) in DCM (50 mL) was added Tf_2O (3.10 g, 11 mmol) at 0 °C under Argon, then the mixture was warmed up to room temperature and stirred for another 2 h. The reaction was quenched with saturated NH_4Cl and extracted with DCM. The combined organic layers were dried over Na_2SO_4, filtered and concentrated in vacuum. The residue was purified by flash column chromatography on silica gel using dichloromethane–petroleum ether mixture (2:1) as an eluent to afford the desired compound **S7** as a white solid (1.85 g, 60% yield).[1]

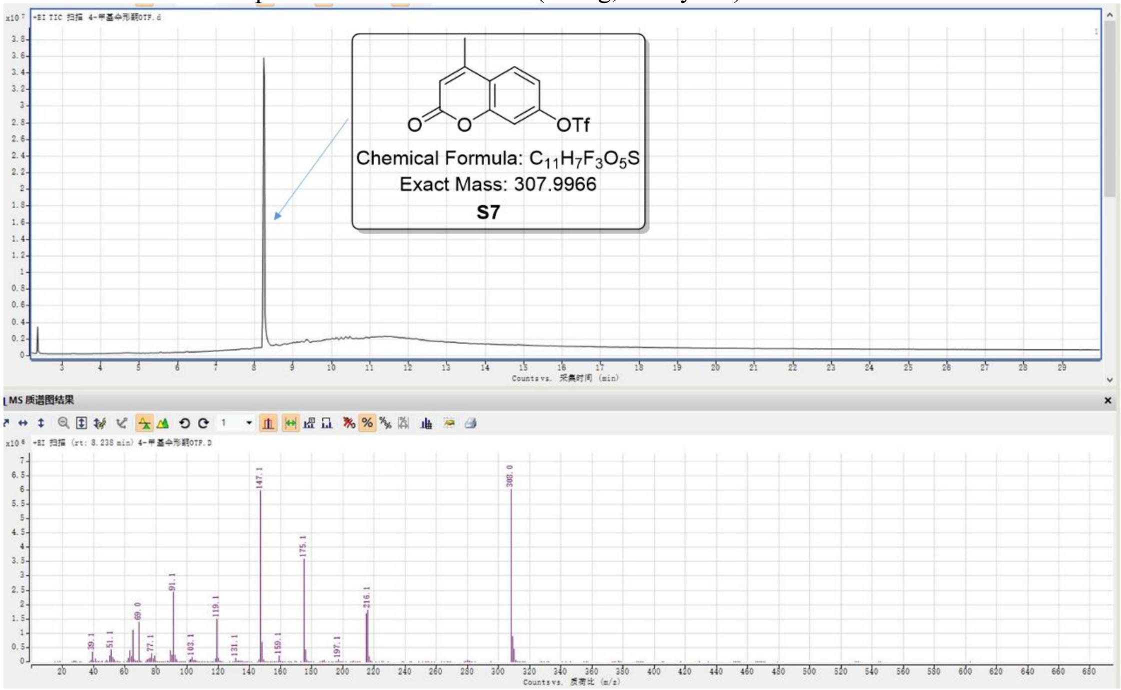

Supplementary Fig. 6 GC-MS spectrum of S7.

To a solution of **S6** (1.54 g, 5 mmol) in 14 mL of 1,4-dioxane were added tri-n-butylethenylstannane (1.46 mL, 5 mmol), LiCl (0.59 g, 14 mmol), $Pd(PPh_3)_4$ (139 mg, 0.1 mmol), and a few crystals of 2,6-di-tert-butyl-4-methyl-phenol. The resulting suspension was heated to 98 °C for 4 h, then cooled to room temperature. The mixture was diluted with EtOAc, filtered through a small pad of Celite, and washed with water, 1M HCl solution, saturated $NaHCO_3$ aqueous solution, and a concentrated sodium chloride solution. The solution was dried over anhydrous Na_2SO_4 and concentrated in vacuum. The residue was purified by flash column chromatography on silica gel using EtOAc–petroleum ether mixture (1:4) as an eluent to afford the desired **alkene 99** as a white solid (700 mg, 72% yield).[3]

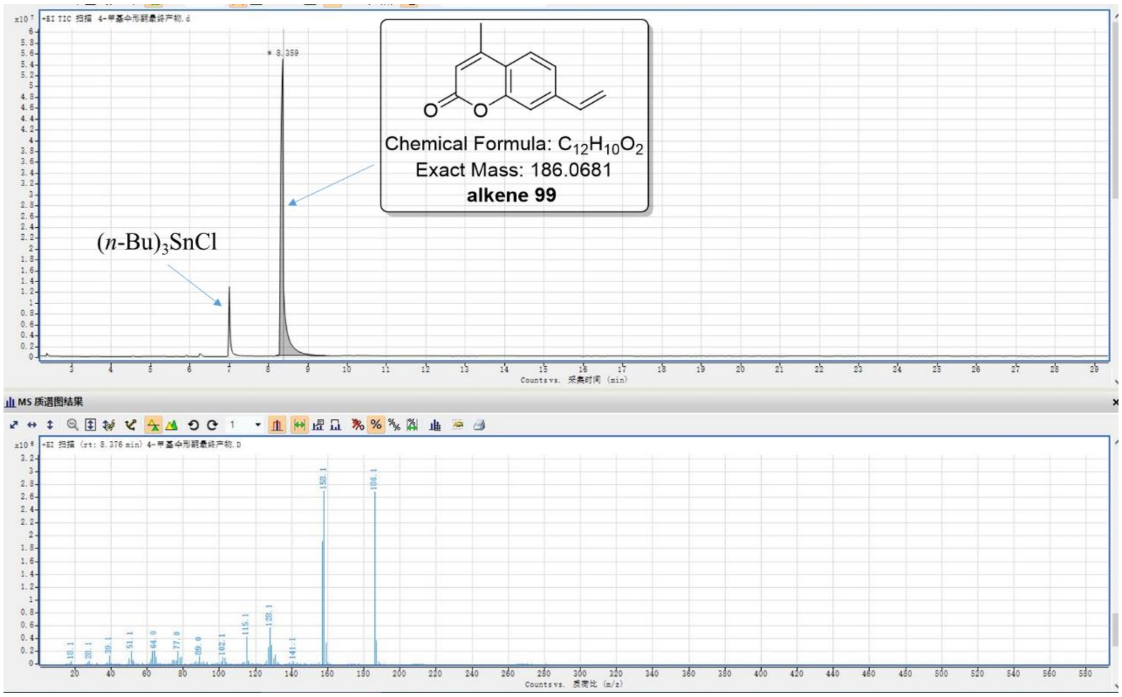

Supplementary Fig. 7 GC-MS spectrum of alkene 99.

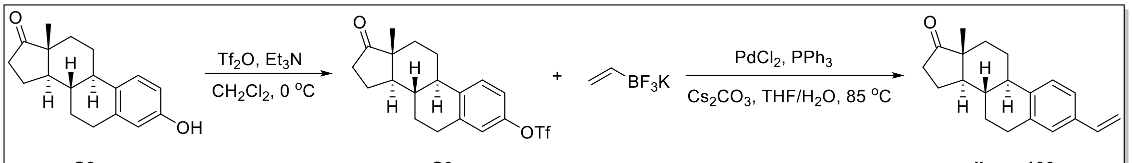

S8 **S9** **alkene 100**

To a solution of **S8** (3.08 g, 10 mmol) and Et_3N (2.02 g, 20 mmol) in DCM (50 mL) was added Tf_2O (3.10 g, 11 mmol) at 0 °C under Argon, then the mixture was warmed up to room temperature and stirred for another 2 h. The reaction was quenched with saturated NH_4Cl and extracted with DCM. The combined organic layers were dried over Na_2SO_4, filtered and concentrated in vacuum. The residue was purified by flash column chromatography on silica gel using dichloromethane–petroleum ether mixture (1:2) as an eluent to afford the desired compound **S9** as a pale yellow solid (3.78 g, 94% yield).[4]

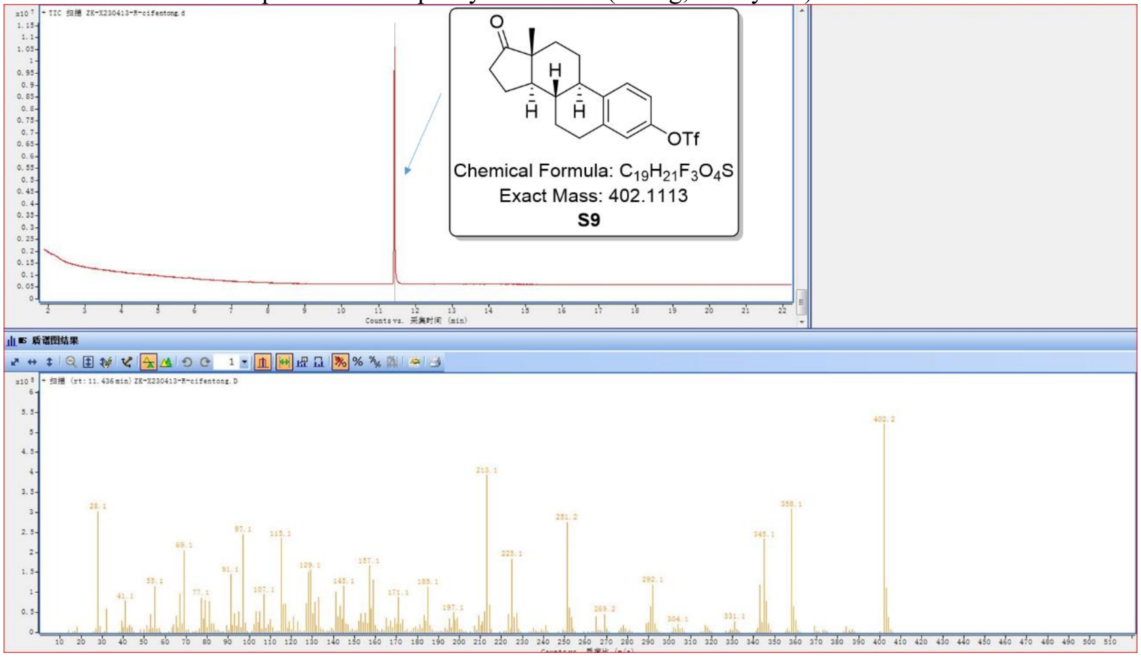

Supplementary Fig. 8 GC-MS spectrum of S9.

A microwave vial with a magnetic stirring bar was charged with **S9** (2.01 mg, 5 mmol), potassium vinyltrifluoroborate (1.34 g, 10 mmol), $PdCl_2$ (89 mg, 0.5 mmol), triphenylphosphine (156 mg, 0.6 mmol) and cesium carbonate (4.89 g, 15 mmol) and then it was evacuated and refilled with argon three times. THF (10 mL) and distilled water (1.3 mL) were added, the vial was sealed with the corresponding cap and the resulting dark brown mixture was stirred at 85 °C for 15 h. More water was added and the mixture was extracted three times with dichloromethane. The combined organic fractions were dried over anhydrous Na_2SO_4, concentrated in vacuum. The residue was purified by flash column chromatography on silica gel using EtOAc–petroleum ether mixture (1:10 to 1:4) as an eluent to afford the desired **alkene 100** as a white solid (1.26 g, 90% yield).[4]

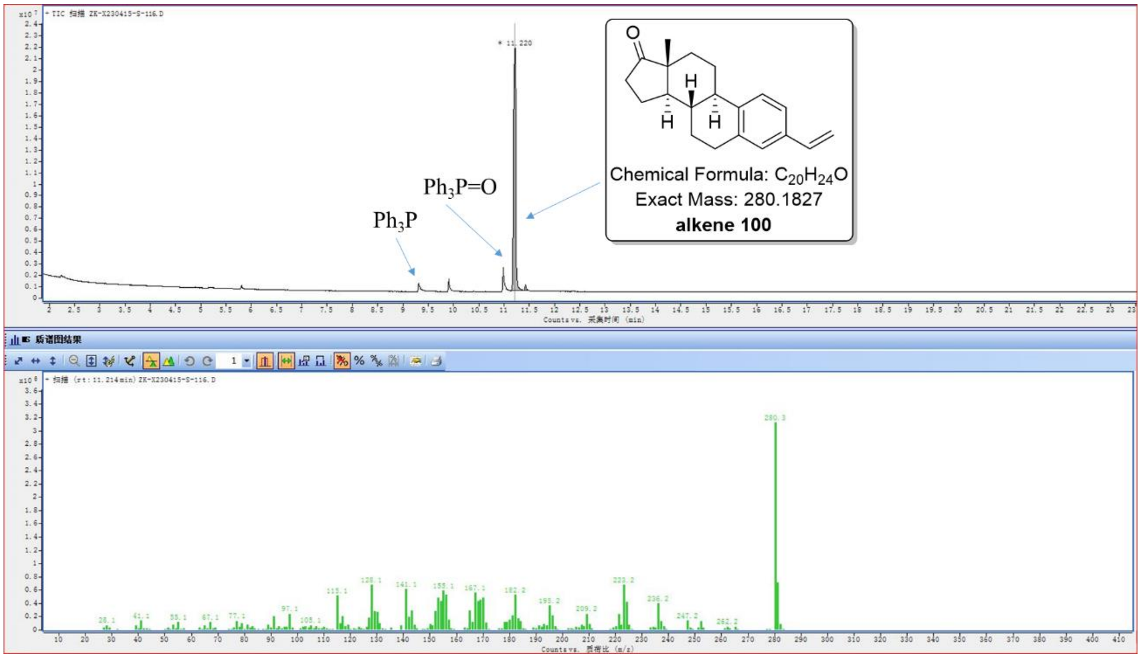

Supplementary Fig. 9 GC-MS spectrum of alkene 100.

Synthetic procedure of L8, and L10 to L14

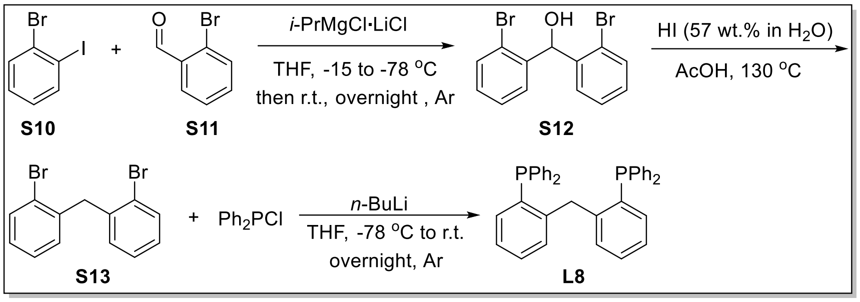

To a -15 ℃ stirring solution of **S10** (5.6 g, 20 mmol) in THF (100 mL) was added *i*-PrMgCl·LiCl (1.3 M, 16.8 mL, 22 mmol). When the exchange was deemed complete (monitored by observing the formation of bromobenzene in quenched aliquots, GCMS), the reaction was cooled to -78 ℃ and **S11** (3.68 g, 20 mmol) was added dropwise with a syringe. The cold bath was left in place and the reaction was allowed to warm to room temperature overnight. The reaction mixture was diluted with EtOAc and quenched with HCl (aq, 6M) until the aqueous layer showed litmus red at which time an additional portion of water was added. The organic phases were separated and the aqueous layer was extracted with EtOAc. The combined organics were washed with brine, dried with Na_2SO_4 and concentrated in vacuum. The residue was purified by flash column chromatography on silica gel using EtOAc–petroleum ether mixture (1:20) as an eluent to afford the desired compound **S12** as a yellow oil liquid (6.12 g, 90% yield).

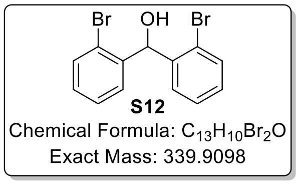

Bis(2-bromophenyl)methanol (S12): ^{1}H NMR (400 MHz, $CDCl_3$) δ 7.63 – 7.42 (m, 2H), 7.30 – 7.18 (m, 4H), 7.10 (ddd, *J* = 8.0, 6.5, 2.6 Hz, 2H), 6.32 (d, *J* = 3.9 Hz, 1H), 2.57 (d, *J* = 4.0 Hz, 1H). **^{13}C NMR** (101 MHz, $CDCl_3$) δ 141.01, 133.07, 129.52, 128.78, 127.72, 123.98, 74.32.[5]

To a solution of bis(2-bromophenyl)methanol (6.12 g, 18 mmol) in acetic acid (120 mL) was added HI (16.4 g, 72 mmol). The reaction mixture was refluxed for 2 hours at which time TLC showed no starting material. The reaction mixture was allowed to cool and stand for 1 day. The reaction mixture was quenched by adding a saturated solution of sodium sulfite until no further color change from dark to yellow was observed. The mixture was diluted with water and the aqueous phase was extracted with EtOAc. The combined organics were washed with NaOH (aq, 1 M) until the washings were litmus blue. The now acid free organics were dried over Na_2SO_4 and concentrated in vacuum. The residue was purified by flash column chromatography on silica gel using petroleum ether as an eluent to afford the desired compound **S13** as a colorless oil liquid (4.66 g, 80% yield).

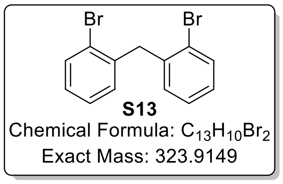

Bis(2-bromophenyl)methane (S13): ^{1}H NMR (400 MHz, $CDCl_3$) δ 7.65 (dd, *J* = 7.9, 1.2 Hz, 2H), 7.27 (td, *J* = 7.5, 1.2 Hz, 2H), 7.16 (td, *J* = 7.8, 1.7 Hz, 2H), 7.03 (dd, *J* = 7.6, 1.5 Hz, 2H), 4.25 (s, 2H). **^{13}C NMR** (101 MHz, $CDCl_3$) δ 138.97, 132.95, 130.81, 128.22, 127.65, 125.20, 42.20.[5]

A solution of *n*-butyllithium in hexane (2.5 M, 2.2 mL, 5.5 mmol) was added dropwise over a period of 5 min to a solution of **S13** (0.82 g, 2.5 mmol) in anhydrous tetrahydrofuran (20 mL) at -78 °C under argon atmosphere. The solution was stirred for 1 h and then chlorodiphenylphosphine (1.21 g, 5.5 mmol) dissolved in anhydrous tetrahydrofuran (5 mL) was added dropwise. The mixture was continued to stir at -78 °C for 1 h and the system was heated to room temperature, and allowed to react overnight. The reaction was quenched with 2N HCl solution. The mixture was extracted with ethyl acetate and water for 3 times, the combined organic phases were dried over anhydrous Na_2SO_4, and concentrated under reduced pressure. The residue

was purified by flash column chromatography on silica gel using EtOAc–petroleum ether mixture (1:50) as an eluent to afford the desired compound **L8** as a white solid (1.12 g, 83% yield).

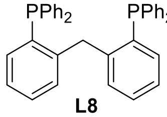

L8
Chemical Formula: $C_{37}H_{30}P_2$
Exact Mass: 536.1823

Bis(2-(diphenylphosphaneyl)phenyl)methane (L8): ^{1}**H NMR** (400 MHz, $CDCl_3$) δ 7.36 – 7.27 (m, 12H), 7.22 (td, *J* = 7.5, 3.4 Hz, 8H), 7.16 (td, *J* = 7.4, 1.4 Hz, 2H), 7.08 (td, *J* = 7.4, 1.1 Hz, 2H), 6.97 – 6.78 (m, 4H), 4.45 (s, 2H). ^{13}**C NMR** (101 MHz, $CDCl_3$) δ 145.47, 145.20, 136.89, 136.87, 136.84, 136.79, 136.76, 136.73, 136.59, 136.56, 136.53, 136.47, 136.44, 136.42, 134.17, 134.07, 133.97, 133.52, 130.20, 130.17, 130.14, 128.95, 128.65, 128.61, 128.58, 128.54, 128.51, 128.48, 126.46, 38.67, 38.43, 38.20. ^{31}**P NMR** (162 MHz, $CDCl_3$) δ -14.78. **HRMS** (ESI)**:** Calcd. for $C_{37}H_{31}P_2$ $[M+H]^+$: 537.1891, found: 537.1896.

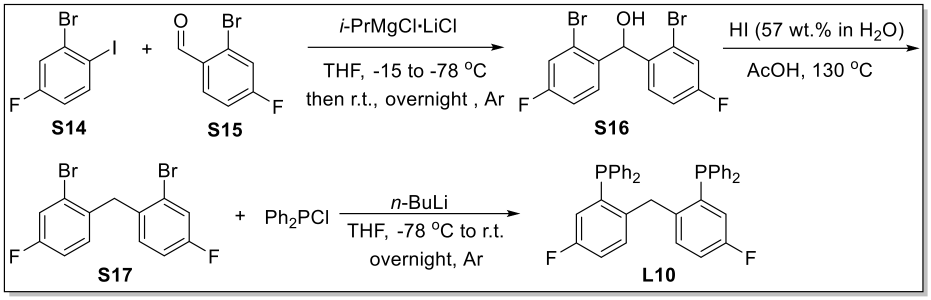

To a -15 ℃ stirring solution of **S14** (6.0 g, 20 mmol) in THF (100 mL) was added *i*-PrMgCl ·LiCl (1.3 M, 16.8 mL, 22 mmol). When the exchange was deemed complete (monitored by observing the formation of bromobenzene in quenched aliquots, GCMS), the reaction was cooled to -78 ℃ and **S15** (4.04 g, 20 mmol) was added dropwise with a syringe. The cold bath was left in place and the reaction was allowed to warm to room temperature overnight. The reaction mixture was diluted with EtOAc and quenched with HCl (aq, 6M) until the aqueous layer showed litmus red at which time an additional portion of water was added. The organic phases were separated and the aqueous layer was extracted with EtOAc. The combined organics were washed with brine, dried with Na_2SO_4 and concentrated in vacuum. The residue was purified by flash column chromatography on silica gel using EtOAc–petroleum ether mixture (1:19) as an eluent to afford the desired compound **S16** as a white solid (4.14 g, 55% yield).

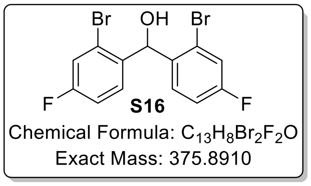

Bis(2-bromo-4-fluorophenyl)methanol (S16): ^{1}H NMR (400 MHz, $CDCl_3$) δ 7.42 – 7.14 (m, 4H), 7.04 (td, *J* = 8.3, 2.6 Hz, 2H), 6.31 (s, 1H), 2.60 (d, *J* = 2.6 Hz, 1H). **^{13}C NMR** (101 MHz, $CDCl_3$) δ 162.02 (d, *J* = 251.5 Hz), 136.94 (d, *J* = 3.5 Hz), 129.72 (d, *J* = 8.6 Hz), 123.89 (d, *J* = 9.6 Hz), 120.42 (d, *J* = 24.6 Hz), 114.88 (d, *J* = 20.9 Hz), 73.23. **^{19}F NMR** (376 MHz, $CDCl_3$) δ -111.92.[6]

To a solution of **S16** (4.14 g, 11 mmol) in acetic acid (80 mL) was added HI (10.9 g, 48 mmol). The reaction mixture was refluxed for 2 hours at which time TLC showed no starting material. The reaction mixture was allowed to cool and stand for 1 day. The reaction mixture was quenched by adding a saturated solution of sodium sulfite until no further color change from dark to yellow was observed. The mixture was diluted with water and the aqueous phase was extracted with EtOAc. The combined organics were washed with NaOH (aq, 1 M) until the washings were litmus blue. The now acid free organics were dried over Na_2SO_4 and concentrated in vacuum. The residue was purified by flash column chromatography on silica gel using petroleum ether as an eluent to afford the desired compound **S17** as a white solid (3.17 g, 80% yield).

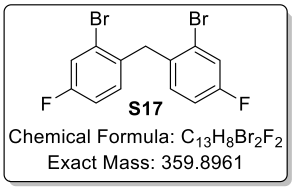

Bis(2-bromo-4-fluorophenyl)methane (S17): ^{1}H NMR (400 MHz, $CDCl_3$) δ 7.35 (dd, *J* = 8.1, 2.0 Hz, 2H), 7.10 – 6.80 (m, 4H), 4.11 (s, 2H). **^{13}C NMR** (101 MHz, $CDCl_3$) δ 161.28 (d, *J* = 249.7 Hz), 134.69 (d, *J* = 3.5 Hz), 131.38 (d, *J* = 8.3 Hz), 124.88 (d, *J* = 9.5 Hz), 120.24 (d, *J* = 24.3 Hz), 114.82 (d, *J* = 20.8 Hz), 40.61. **^{19}F NMR** (376 MHz, $CDCl_3$) δ -114.02.[6]

A solution of *n*-butyllithium in hexane (2.5 M, 2.2 mL, 5.5 mmol) was added dropwise over a period of 5 min to a solution of **S17** (0.905 g, 2.5 mmol) in anhydrous tetrahydrofuran (20 mL) at -78 °C under argon atmosphere. The solution was stirred for 1 h and then chlorodiphenylphosphine (1.21 g, 5.5 mmol) dissolved in anhydrous tetrahydrofuran (5 mL) was added dropwise. The mixture was continued to stir at -78 °C for 1 h and the system was heated to room temperature, and allowed to react overnight. The reaction was quenched with 2N HCl solution. The mixture was extracted with ethyl acetate and water for 3 times, the combined organic phases were dried over anhydrous Na_2SO_4, and concentrated under reduced pressure. The residue

was purified by flash column chromatography on silica gel using EtOAc–petroleum ether mixture (1:25) as an eluent to afford the desired compound **L10** as a white solid (0.5 g, 35% yield).

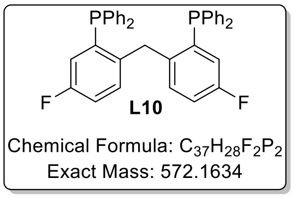

Bis(2-(diphenylphosphanyl)-4-fluorophenyl)methane (L10): ^{1}**H NMR** (400 MHz, $CDCl_3$) δ 7.35 – 7.27 (m, 12H), 7.23 – 7.11 (m, 8H), 6.81 (d, *J* = 6.7 Hz, 4H), 6.56 (d, *J* = 9.3 Hz, 2H), 4.28 (s, 2H). ^{13}**C NMR** (101 MHz, $CDCl_3$) δ 162.70, 160.25, 140.61, 140.58, 140.34, 140.31, 139.52, 139.47, 139.36, 139.32, 135.87, 135.84, 135.80, 135.77, 134.20, 134.18, 134.08, 133.98, 133.96, 131.60, 131.57, 131.53, 131.49, 131.46, 129.07, 128.78, 128.75, 128.71, 119.88, 119.67, 115.86, 115.65, 37.13, 36.90, 36.68. ^{19}**F NMR** (376 MHz, $CDCl_3$) δ -116.16. ^{31}**P NMR** (162 MHz, $CDCl_3$) δ -14.28. **HRMS** (ESI)**:** Calcd. for $C_{37}H_{29}F_2P_2$ $[M+H]^+$: 573.1713, found: 573.1697.

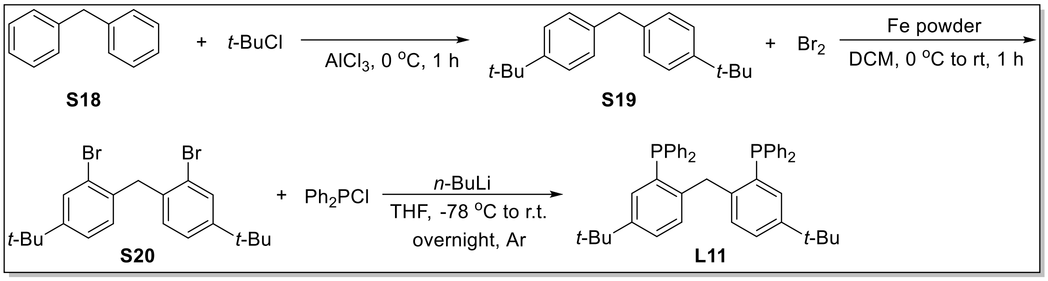

In a 500 mL round bottom flask equipped with a $CaCl_2$ drying tube and a magnetic stirring bar, anhydrous $AlCl_3$ (0.14 g, 1.02 mmol) were added to the mixture of diphenylmethane (20.0g, 119 mmol) and tert-butyl chloride (33 mL, 303 mmol) at 0 ℃ under vigorous stirring. After 5 min, a second portion of anhydrous $AlCl_3$ (0.16 g, 1.23 mmol) were added. After vigorous stirring for 1 h, the hard mass obtained was recrystallized from hot isopropanol. After cooling in a refrigerator, the precipitated products were collected and washed with cold isopropanol to give a white solid **S19** (24.0 g, 72%).

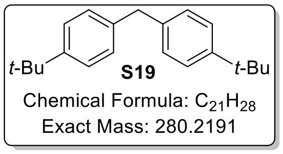

Bis(4-(tert-butyl)phenyl)methane (S19): ^{1}H NMR (400 MHz, $CDCl_3$) δ 7.29 (d, *J* = 8.3 Hz, 4H), 7.12 (d, *J* = 8.3 Hz, 4H), 3.91 (s, 2H), 1.29 (s, 18H). **^{13}C NMR** (101 MHz, $CDCl_3$) δ 148.85, 138.39, 128.69, 125.45, 41.08, 34.50, 31.57.[7]

In a three-necked round-bottom 200 mL flask equipped with a dropping funnel with a pressure-equalizing bypass, a $CaCl_2$ drying tube, and a magnetic stirring bar, a solution of bromine (7.7 mL, 150 mmol) in CH_2Cl_2 (36 mL) was added dropwise to a mixture of **S19** (20.0 g, 72 mmol) and iron powder (0.36 g, 6.4 mmol) in CH_2Cl_2 (72 mL) under vigorous stirring for about 30 min at 0 ºC. The reaction mixture was allowed to warm to room temperature, and additionally was stirred for 1h. Then, the mixture was washed with aqueous $NaHSO_3$. The organic layer was separated, and the aqueous layer was washed twice with CH_2Cl_2. The combined organic extract was dried over anhydrous $NaSO_4$ and evaporated. The residue was purified by flash column chromatography on silica gel using EtOAc–petroleum ether mixture (1:4) as an eluent to afford the desired compound **S20** as a yellowish solid (30.9 g, 99 %).[7]

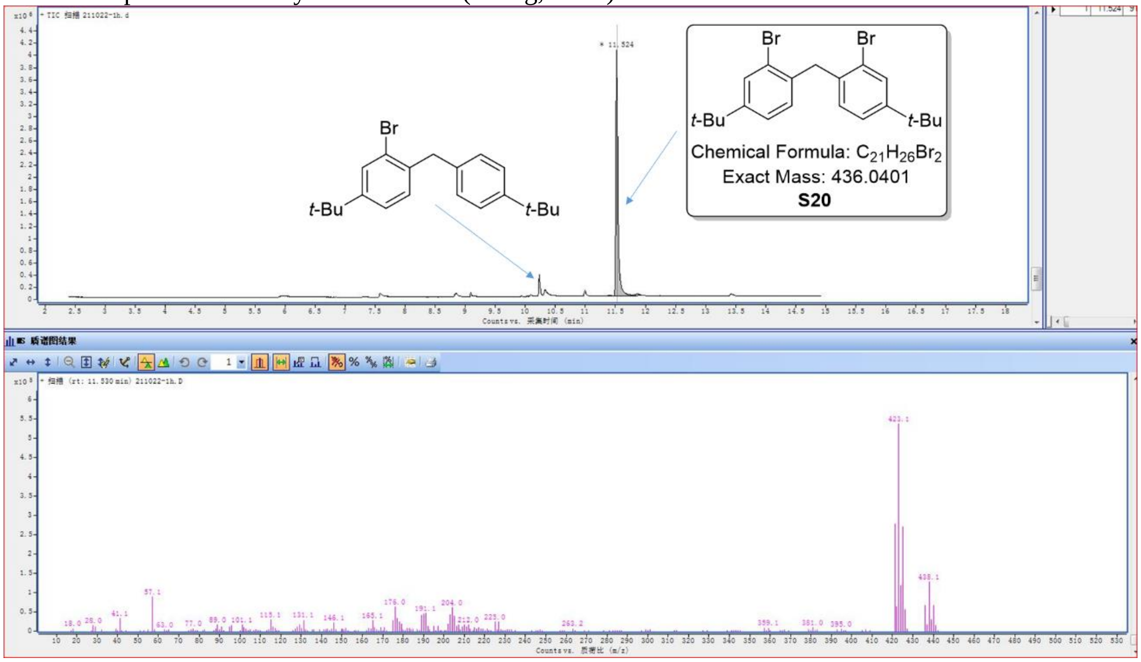

Supplementary Fig. 10 GC-MS spectrum of S20.

A solution of *n*-butyllithium in hexane (2.5 M, 8.8 mL, 22 mmol) was added dropwise over a period of 10 min to a solution of **S20** (4.38 g, 10 mmol) in anhydrous tetrahydrofuran (60 mL) at -78 ºC under argon atmosphere. The solution was stirred for 1 h and then chlorodiphenylphosphine (4.84 g, 22 mmol) dissolved in anhydrous tetrahydrofuran (5 mL) was added dropwise. The mixture was continued to stir at -78 ºC for 1 h and the system was heated to room temperature, and allowed to react overnight. The reaction was quenched with 2N HCl solution. The mixture was extracted with ethyl acetate and water for 3 times, the combined organic phases were dried over anhydrous Na_2SO_4, and concentrated under reduced pressure. The residue was purified by flash column chromatography on silica gel using EtOAc–petroleum ether mixture (1:50) as an eluent to afford the desired compound **L11** as a white solid (4.2 g, 65% yield).

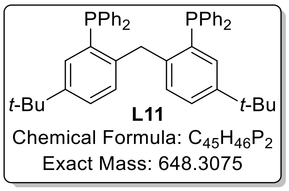

L11
Chemical Formula: $C_{45}H_{46}P_2$
Exact Mass: 648.3075

Bis(4-(*tert*-butyl)-2-(diphenylphosphaneyl)phenyl)methane (L11): ^{1}**H NMR** (400 MHz, $CDCl_3$) δ 7.30 (dd, *J* = 4.0, 2.3 Hz, 12H), 7.26 – 7.19 (m, 8H), 7.13 (dd, *J* = 8.0, 2.1 Hz, 2H), 6.88 (dd, *J* = 4.5, 2.1 Hz, 2H), 6.84 (dd, *J* = 8.0, 4.6 Hz, 2H), 4.37 (s, 2H), 1.08 (s, 18H). ^{13}**C NMR** (101 MHz, $CDCl_3$) δ 148.59, 142.63, 142.36, 135.55, 135.43, 134.14, 134.04, 133.94, 130.92, 129.72, 128.54, 128.44, 128.41, 128.38, 125.73, 37.71, 37.48, 37.26, 34.53, 31.25. ^{31}**P NMR** (162 MHz, $CDCl_3$) δ -13.65. **HRMS** (ESI)**:** Calcd. for $C_{45}H_{47}P_2$ $[M+H]^+$: 649.3135, found: 649.3148.

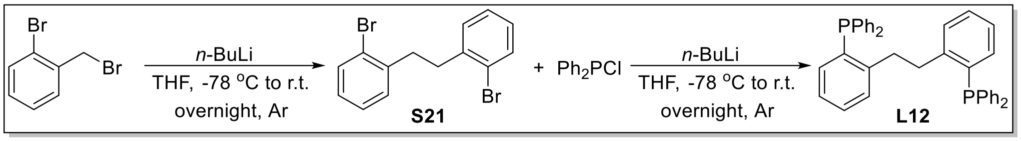

2-Bromobenzyl bromide (3.6 g, 14.4 mmol) was dissolved in dry THF (20 mL) under an argon atmosphere and cooled to -78 ℃. Then *n*-BuLi (2.5 M, 2.88 mL, 7.2 mmol) was added dropwise to the stirred solution. After stirring at this temperature for a further 1 h, water (10 mL) was added and the reaction was allowed to warm to room temperature. The organic layers were separated and the aqueous layer were extracted with EtOAc. The combined organic phases were washed with a saturated solution of ammonium chloride, dried over anhydrous Na_2SO_4 and concentrated under reduced pressure. The residue was purified by flash column chromatography on silica gel using petroleum ether as an eluent and recrystallized from petroleum ether to afford the desired compound **S21** as a white solid (2.23 g, 91% yield).

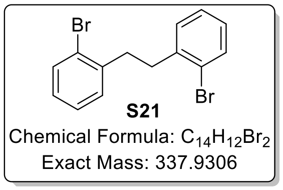

1,2-Bis(2-bromophenyl)ethanee (S21): **^{1}H NMR** (400 MHz, $CDCl_3$) δ 7.53 (d, *J* = 8.1 Hz, 2H), 7.29 – 7.11 (m, 4H), 7.10 – 6.93 (m, 2H), 3.03 (s, 4H). **^{13}C NMR** (101 MHz, $CDCl_3$) δ 140.68, 132.91, 130.75, 127.95, 127.57, 124.60, 77.16, 36.56.[8]

A solution of *n*-butyllithium in hexane (2.5 M, 2.2 mL, 5.5 mmol) was added dropwise over a period of 5 min to a solution of **S21** (0.845 g, 2.5 mmol) in anhydrous tetrahydrofuran (20 mL) at -78 °C under argon atmosphere. The solution was stirred for 1 h and then chlorodiphenylphosphine (1.21 g, 5.5 mmol) dissolved in anhydrous tetrahydrofuran (5 mL) was added dropwise. The mixture was continued to stir at -78 °C for 1 h and the system was heated to room temperature, and allowed to react overnight. The reaction was quenched with 2N HCl solution. The mixture was extracted with ethyl acetate and water for 3 times, the combined organic phases were dried over anhydrous Na_2SO_4, and concentrated under reduced pressure. The residue was purified by flash column chromatography on silica gel using EtOAc–petroleum ether mixture (1:5) as an eluent to afford the desired compound **L12** as a white solid (0.825 g, 60% yield).

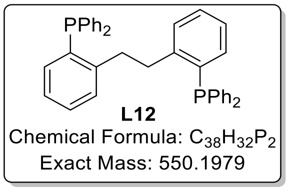

1,2-Bis(2-(diphenylphosphaneyl)phenyl)ethane (L12): **^{1}H NMR** (400 MHz, $CDCl_3$) δ 7.46 – 7.15 (m, 22H), 7.07 (ddd, *J* = 7.3, 4.3, 3.0 Hz, 4H), 6.90 – 6.77 (m, 2H), 3.07 (s, 4H). **^{13}C NMR** (101 MHz, $CDCl_3$) δ 146.80, 146.54, 137.28, 137.17, 135.49, 135.37, 134.16, 133.96, 133.73, 129.66, 129.63, 129.60, 129.14, 128.74, 128.67, 128.63, 128.60, 126.40, 36.65, 36.63, 36.44, 36.42. **^{31}P NMR** (162 MHz, $CDCl_3$) δ -15.80. **HRMS** (ESI)**:** Calcd. for $C_{38}H_{33}P_2$ $[M+H]^+$: 551.2054, found: 551.2052.

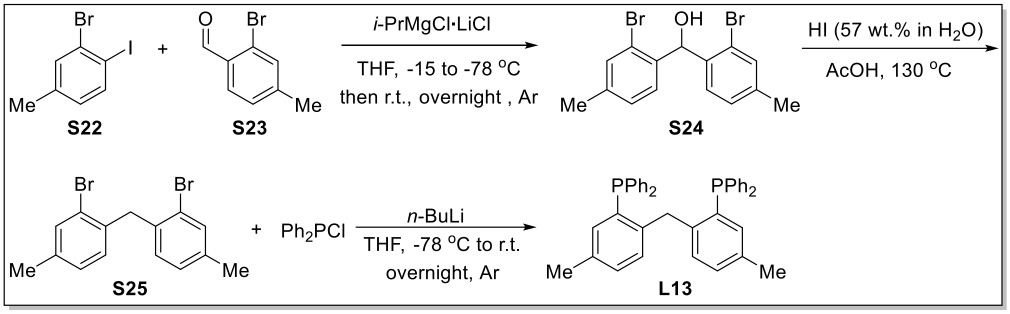

To a -15 ℃ stirring solution of **S22** (5.92 g, 20 mmol) in THF (100 mL) was added *i*-PrMgCl·LiCl (1.3 M, 16.8 mL, 22 mmol). When the exchange was deemed complete (monitored by observing the formation of bromobenzene in quenched aliquots, GCMS), the reaction was cooled to -78 ℃ and **S23** (3.96 g, 20 mmol) was added dropwise with a syringe. The cold bath was left in place and the reaction was allowed to warm to room temperature overnight. The reaction mixture was diluted with EtOAc and quenched with HCl (aq, 6M) until the aqueous layer showed litmus red at which time an additional portion of water was added. The organic phases were separated and the aqueous layer was extracted with EtOAc. The combined organics were washed with brine, dried with Na_2SO_4 and concentrated in vacuum. The residue was purified by flash column chromatography on silica gel using EtOAc–petroleum ether mixture (1:20) as an eluent to afford the desired compound **S24** as a white solid (6.62 g, 90% yield).[6]

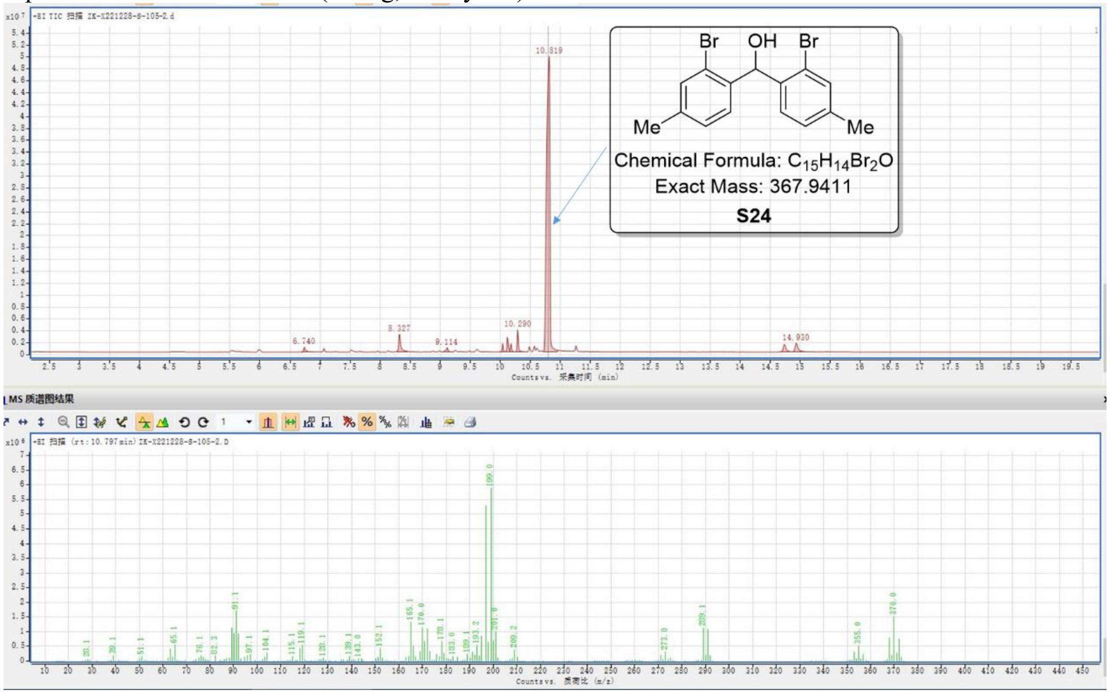

Supplementary Fig. 11 GC-MS spectrum of S24.

To a solution of **S24** (6.62 g, 18 mmol) in acetic acid (120 mL) was added HI (16.4 g, 72 mmol). The reaction mixture was refluxed for 2 hours at which time TLC showed no starting material. The reaction mixture was allowed to cool and stand for 1 day. The reaction mixture was quenched by adding a saturated solution of sodium sulfite until no further color change from dark to yellow was observed. The mixture was diluted with water and the aqueous phase was extracted with EtOAc. The combined organics were washed with NaOH (aq, 1 M) until the washings were litmus blue. The now acid free organics were dried over Na_2SO_4 and concentrated in vacuum. The residue was purified by flash column chromatography on silica gel using petroleum ether as an eluent to afford the desired compound **S25** as a yellow oil liquid (5.70 g, 90% yield).

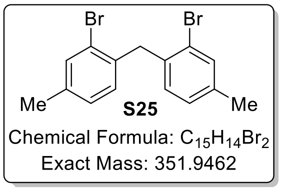

Bis(2-bromo-4-methylphenyl)methane (S25): ^{1}H NMR (400 MHz, $CDCl_3$) δ 7.40 (s, 2H), 6.99 (d, *J* = 7.8 Hz, 2H), 6.84 (d, *J* = 7.8 Hz, 2H), 4.09 (s, 2H), 2.28 (s, 6H). **^{13}C NMR** (101 MHz, $CDCl_3$) δ 138.09, 136.04, 133.32, 130.46, 128.40, 124.85, 41.29, 20.75.[6]

A solution of *n*-butyllithium in hexane (2.5 M, 2.2 mL, 5.5 mmol) was added dropwise over a period of 5 min to a solution of **S25** (0.885 g, 2.5 mmol) in anhydrous tetrahydrofuran (20 mL) at -78 ^{o}C under argon atmosphere. The solution was stirred for 1 h and then chlorodiphenylphosphine (1.21 g, 5.5 mmol) dissolved in anhydrous tetrahydrofuran (5 mL) was added dropwise. The mixture was continued to stir at -78 ^{o}C for 1 h and the system was heated to room temperature, and allowed to react overnight. The reaction was quenched with 2N HCl solution. The mixture was extracted with ethyl acetate and water for 3 times, the combined organic phases were dried over anhydrous Na_2SO_4, and concentrated under reduced pressure. The residue was purified by flash column chromatography on silica gel using EtOAc–petroleum ether mixture (1:50) as an eluent to afford the desired compound **L13** as a white solid (0.59 g, 42% yield).

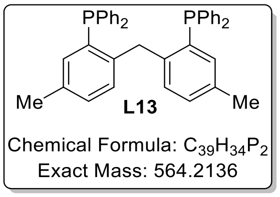

Bis(2-(diphenylphosphanyl)-4-methylphenyl)methane (L13): ^{1}H NMR (400 MHz, $CDCl_3$) δ 7.42 – 7.11 (m, 20H), 6.94 (d, *J* = 7.4 Hz, 2H), 6.84 – 6.72 (m, 2H), 6.68 (s, 2H), 4.35 (s, 2H), 2.13 (s, 6H). **^{13}C NMR** (101 MHz, $CDCl_3$) δ 142.78, 142.51, 137.06, 136.96, 136.07, 135.95, 135.66, 134.14, 134.04, 133.93, 130.09, 130.06, 130.03, 129.82, 128.52, 128.48, 128.44, 37.83, 37.61, 37.38, 21.24. **^{31}P NMR** (162 MHz, $CDCl_3$) δ -14.48. **HRMS** (ESI)**:** Calcd. for $C_{39}H_{35}P_2$ $[M+H]^+$: 565.2214, found: 565.2209.

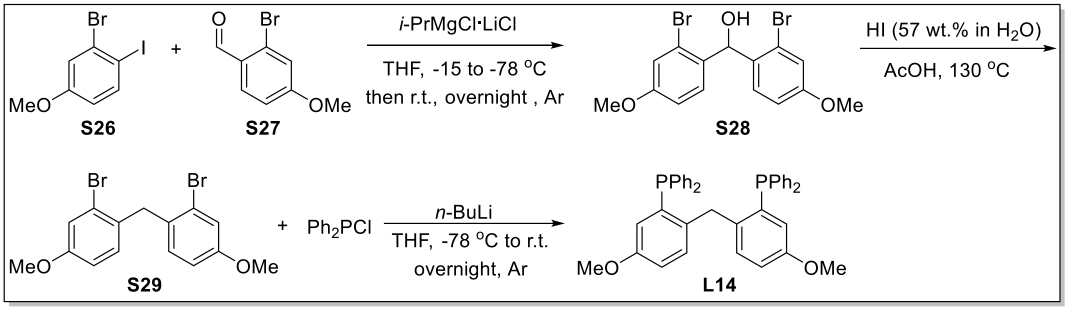

To a -15 ℃ stirring solution of **S26** (6.24 g, 20 mmol) in THF (100 mL) was added *i*-PrMgCl·LiCl (1.3 M, 16.8 mL, 22 mmol). When the exchange was deemed complete (monitored by observing the formation of bromobenzene in quenched aliquots, GCMS), the reaction was cooled to -78 ℃ and **S27** (4.26 g, 20 mmol) was added dropwise with a syringe. The cold bath was left in place and the reaction was allowed to warm to room temperature overnight. The reaction mixture was diluted with EtOAc and quenched with HCl (aq, 6M) until the aqueous layer showed litmus red at which time an additional portion of water was added. The organic phases were separated and the aqueous layer was extracted with EtOAc. The combined organics were washed with brine, dried with Na_2SO_4 and concentrated in vacuum. The residue was purified by flash column chromatography on silica gel using EtOAc–petroleum ether mixture (1:20 to 1:4) as an eluent to afford the desired compound **S28** as a white solid (4.8 g, 60% yield).

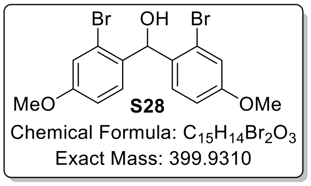

Bis(2-bromo-4-methoxyphenyl)methanol (S28): ^{1}**H NMR** (400 MHz, $CDCl_3$) δ 7.23 (d, *J* = 8.7 Hz, 2H), 7.12 (d, *J* = 2.6 Hz, 2H), 6.84 (dd, *J* = 8.7, 2.6 Hz, 2H), 6.28 (d, *J* = 2.4 Hz, 1H), 3.79 (s, 6H), 2.48 (d, *J* = 3.2 Hz, 1H). ^{13}**C NMR** (101 MHz, $CDCl_3$) δ 159.66, 133.52, 129.26, 124.12, 118.29, 113.52, 73.51.[9]

To a solution of **S28** (4.8 g, 12 mmol) in acetic acid (80 mL) was added HI (10.9 g, 48 mmol). The reaction mixture was refluxed for 2 hours at which time TLC showed no starting material. The reaction mixture was allowed to cool and stand for 1 day. The reaction mixture was quenched by adding a saturated solution of sodium sulfite until no further color change from dark to yellow was observed. The mixture was diluted with water and the aqueous phase was extracted with EtOAc. The combined organics were washed with NaOH (aq, 1 M) until the washings were litmus blue. The now acid free organics were dried over Na_2SO_4 and concentrated in vacuum. The residue was purified by flash column chromatography on silica gel using petroleum ether as an eluent to afford the desired compound **S29** as a white solid (2.3 g, 50% yield).

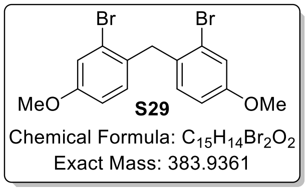

Bis(2-bromo-4-methoxyphenyl)methane (S29): ^{1}**H NMR** (400 MHz, $CDCl_3$) δ 7.14 (d, *J* = 2.6 Hz, 2H), 6.88 (d, *J* = 8.5 Hz, 2H), 6.76 (dd, *J* = 8.5, 2.6 Hz, 2H), 4.05 (s, 2H), 3.77 (s, 6H). ^{13}**C NMR** (101 MHz, $CDCl_3$) δ 158.74, 131.33, 131.06, 125.12, 118.10, 113.68, 77.16, 55.62, 40.33.[9]

A solution of *n*-butyllithium in hexane (2.5 M, 2.2 mL, 5.5 mmol) was added dropwise over a period of 5 min to a solution of **S29** (0.965 g, 2.5 mmol) in anhydrous tetrahydrofuran (20 mL) at -78 ºC under argon atmosphere. The solution was stirred for 1 h and then chlorodiphenylphosphine (1.21 g, 5.5 mmol) dissolved in anhydrous tetrahydrofuran (5 mL) was added dropwise. The mixture was continued to stir at -78 ºC for 1 h and the system was heated to room temperature, and allowed to react overnight. The reaction was quenched with 2N HCl solution. The mixture was extracted with ethyl acetate and water for 3 times, the combined organic phases were dried over anhydrous Na_2SO_4, and concentrated under reduced pressure. The residue was purified by flash column chromatography on silica gel using EtOAc–petroleum ether mixture (1:50 to 1:20) as an eluent to afford the desired compound **L14** as a white solid (0.745 g, 50% yield).

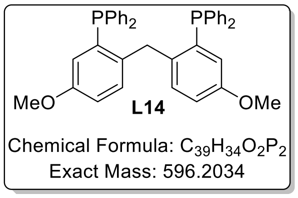

Bis(2-(diphenylphosphanyl)-4-methoxyphenyl)methane (L14): ^{1}H NMR (400 MHz, $CDCl_3$) δ 7.16 (d, *J* = 22.5 Hz, 20H), 6.73 (s, 2H), 6.62 – 6.47 (m, 2H), 6.33 (s, 2H), 4.17 (s, 2H), 3.46 (s, 6H). **^{13}C NMR** (101 MHz, $CDCl_3$) δ 157.75, 137.81, 137.74, 137.61, 137.55, 136.70, 136.60, 134.15, 134.05, 133.95, 131.05, 128.68, 128.54, 128.51, 128.47, 119.07, 113.93, 55.02, 36.84, 36.61, 36.39. **^{31}P NMR** (162 MHz, $CDCl_3$) δ -13.67. **HRMS** (ESI)**:** Calcd. for $C_{39}H_{35}O_2P_2$ $[M+H]^+$: 597.2112, found: 597.2108.

Synthetic procedure of L9

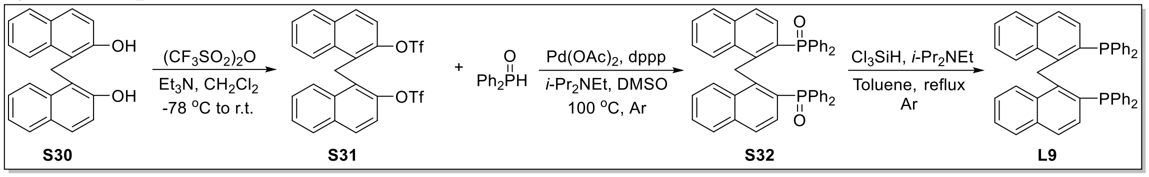

Ligand **L9** was prepared according to literature precedent.[10]

To a stirred solution of **S30** (3.0 g, 10 mmol) and Et_3N (4.7 ml, 33 mmol) in anhydrous dichloromethane (20 ml) was added dropwise trifluoromethanesulfonic anhydride (4.5 ml, 26 mmol) at -78 °C over a 20 min period. The reaction mixture was stirred at -78 °C for 4 h, then allowed to warm to room temperature. Following removal of the solvent in vacuum, the residue was diluted with EtOAc (20 ml), washed with 5% aqueous HCl, saturated $NaHCO_3$ and brine (once for each). The organic layer was dried over Na_2SO_4, concentrated under reduced pressure, and purified by flash column chromatography on silica gel using EtOAc–petroleum ether mixture (1:10 to 1:3) as an eluent to afford the desired compound **S31** as a white solid (5.0 g, yield 89%).

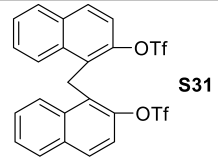

Chemical Formula: $C_{23}H_{14}F_6O_6S_2$
Exact Mass: 564.0136

Bis(2-trifluoromethanesulfonyloxynaphthyl)methane (S31): **^{1}H NMR** (400 MHz, $CDCl_3$) δ 7.85 (t, *J* = 7.9 Hz, 6H), 7.55 – 7.43 (m, 4H), 7.39 (t, *J* = 7.6 Hz, 2H), 5.09 (s, 2H). **^{13}C NMR** (101 MHz, $CDCl_3$) δ 145.70, 132.94, 132.64, 130.09, 129.10, 127.76, 127.68, 126.90, 124.41, 120.38, 119.59, 117.20, 24.73. **^{19}F NMR** (376 MHz, $CDCl_3$) δ -73.26. **HRMS** (ESI)**:** Calcd. for $C_{23}H_{14}F_6O_6S_2Na$ $[M+Na]^+$: 587.0025, found: 587.0028.

Under argon atmosphere, to a mixure of **S31** (1.13 g, 2 mmol), diphenylphosphine oxide (1.05, 5.2 mmol), palladium acetate (45 mg, 0.2 mmol) and 1,3-bis(diphenylphospino)-propane (dppp, 82 mg, 0.2 mmol) were added degassed DMSO (15 ml) and diisopropylethylamine (2.0 ml), and the resulting mixture was heated with stirring at 100 °C for 6 h. After completion of the reaction (monitored by TLC), the mixture was cooled to room temperature and diluted with ethyl acetate (30 ml), and then washed sequentially with 5% aqueous HCl (20 ml), saturated aqueous $NaHCO_3$ (20 ml) and brine (20 ml). The organic phase was dried over anhydrous Na_2SO_4 and concentrated under reduced pressure. The residue was purified by flash column chromatography on silica gel using CH_2Cl_2–MeOH mixture (100:1) as an eluent to afford the desired compound **S32** as a white solid (1.04 g, yield 78%).

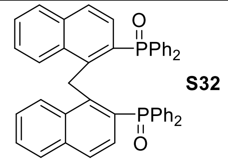

Chemical Formula: $C_{45}H_{34}O_2P_2$
Exact Mass: 668.2034

(Methylenebis(naphthalene-1,2-diyl))bis(diphenylphosphine oxide) (S32): ^{1}H NMR (400 MHz, $CDCl_3$) δ 8.08 (d, *J* = 8.5 Hz, 2H), 7.80 – 7.54 (m, 4H), 7.56 – 7.20 (m, 16H), 7.11 (dd, *J* = 18.3, 9.8 Hz, 8H), 6.95 (t, *J* = 7.5 Hz, 2H), 5.72 (s, 2H). **^{13}C NMR** (101 MHz, $CDCl_3$) δ 146.78, 146.70, 135.29, 135.27, 134.17, 133.75, 133.15, 133.11, 133.00, 132.71, 132.40, 132.31, 131.71, 131.69, 131.43, 131.42, 131.22, 131.12, 128.60, 128.48, 128.39, 127.86, 127.51, 127.37, 127.19, 126.75, 126.62, 126.36, 126.07, 37.97, 37.91, 37.85. **^{31}P NMR** (162 MHz, $CDCl_3$) δ 32.17. **HRMS** (ESI)**:** Calcd. for Calcd. for $C_{45}H_{34}O_2P_2Na$ $[M+Na]^+$: 691.1930, found: 691.1926.

Under argon atmosphere, to a toluene solution (15 ml) of compound **S32** (1.0 g, 1.5 mmol) at 0 °C were added diisopropylethylamine (9.6 ml, 55 mmol) and $HSiCl_3$ (4.5 ml, 44 mmol), and the resulting mixture refluxed for 20 h. After cooling to room temperature, the mixture was diluted with Et_2O and the reaction

quenched with small amount of saturated aqueous $NaHCO_3$ solution (about 0.5~1.0 ml). The resulting suspension was filtered through celite and the solid washed with Et_2O. The combined organic layer was washed with brine, dried over anhydrous Na_2SO_4, and concentrated under reduced pressure. The residue was purified by flash column chromatography on silica gel using EtOAc–petroleum ether mixture (1:50) as an eluent to afford the desired compound **L9** as a white solid (770 mg, yield 81%).

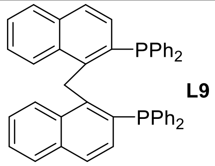

Chemical Formula: $C_{45}H_{34}P_2$
Exact Mass: 636.2136

Bis(2-(diphenylphosphaneyl)naphthalen-1-yl)methane (L9): ^{1}H NMR (400 MHz, $CDCl_3$) δ 8.01 (d, *J* = 8.6 Hz, 2H), 7.54 (d, *J* = 8.1 Hz, 2H), 7.46 (d, *J* = 8.5 Hz, 2H), 7.37 – 7.02 (m, 24H), 6.97 (t, *J* = 7.6 Hz, 2H), 5.67 (t, *J* = 7.1 Hz, 2H). **^{13}C NMR** (101 MHz, $CDCl_3$) δ 143.75, 143.61, 143.48, 135.88, 133.15, 132.49, 132.41, 131.90, 129.20, 127.48, 127.29, 125.97, 125.03, 124.56, 35.31, 35.00, 34.68. **^{31}P NMR** (162 MHz, $CDCl_3$) δ -13.25. **HRMS** (ESI)**:** Calcd. for Calcd. for $C_{45}H_{34}P_2Na$ $[M+Na]^+$: 637.2211, found: 637.2209.

Supplementary Note 2: Optimization of the reaction conditions

Supplementary Table 1. The effect of ligand.[a]

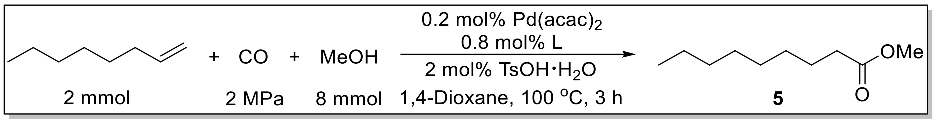

Entry	Ligand	Yield (%)	L/B[b]
1	L1	0	-
2	L2	37	72/28
3	L3	2	75/25
4	L4	0	-
5	L5	0	-
6	L6	6	82/18
7	L7	41	86/14
8	L8	44	91/9
9	L9	23	86/14
10	L10	30	91/9
11	L11	66	91/9
12	L12	0	-
13	L13	50	90/10
14	L14	42	90/10
15	L15	0	-
16	L16	0	-
17	L17	0	-

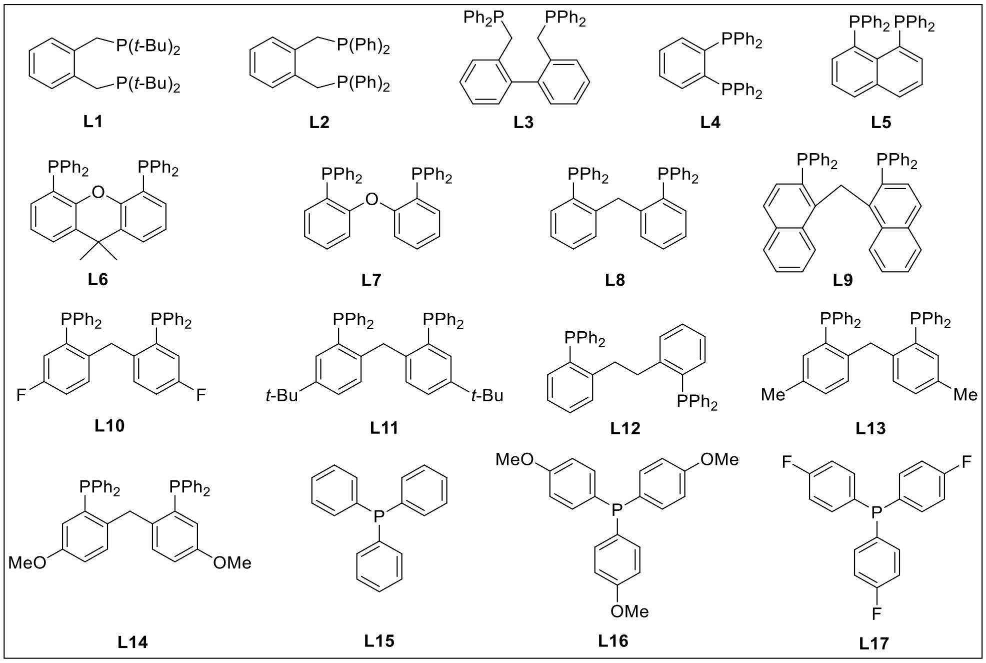

[a] Reaction conditions: 1-octene (2 mmol), MeOH (8 mmol), Pd(acac)$_2$ (0.2 mol %), L (0.8 mol%), TsOH·H_2O (2 mol%), CO (2 MPa), 1,4-Dioxane (4 ml), 100 °C, 3 h; 1.6 mol% of ligand were used for **L15-L17**. GC yields of linear and branched esters are determined based on 1-octene using *n*-decane as the internal standard.
[b] L/B (the ratio of linear/branched esters) is determined by GC analysis.

Supplementary Table 2. The effect of solvent.[a]

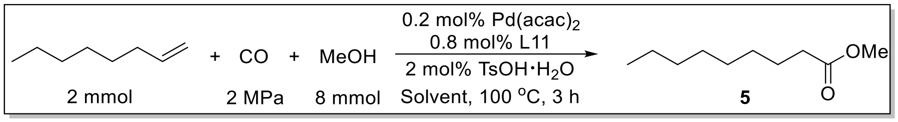

Entry	Solvent	Yield (%)	L/B[b]
1	MeOH	60	74/26
2	THF	48	89/11
3	1,4-Dioxane	66	91/9
4	Tetrahydropyrane	50	90/10
5	18-Crown ether-6	60	82/18
6	1, 3-Dioxy-pentacycline	N.R.	-
7	*n*-Butyl ether	N.R.	-
8	*n*-Propyl ether	N.R.	-
9	DME	N.R.	-
10	Anisole	62	86/14
11	MTBE	46	86/14
12	Thiophene	66	87/13
13	Thiophane	3	50/50
14	Dibutyl sulfide	48	88/12
15	Toluene	71	81/19
16	Mesitylene	65	84/16
17	Xylenes	60	83/17
18	Hexane	74	81/19
19	Cyclohexane	54	84/16
20	Pyrrolidine	N.R.	-
21	CH_3CN	N.R.	-
22[c]	Pyridine	N.R.	-
23	DMF	N.R.	-
24	DCE	63	74/26

[a] Reaction conditions: 1-octene (2 mmol), MeOH (8 mmol), Pd(acac)$_2$ (0.2 mol %), L11 (0.8 mol%), TsOH H_2O (2 mol%), CO (2 MPa), Solvent (4 ml), 100 °C, 3 h. GC yields of linear and branched esters are determined based on 1-octene using *n*-decane as the internal standard. [b] L/B (the ratio of linear/branched esters) is determined by GC analysis.

Supplementary Table 3. The effect of CO pressure.[a]

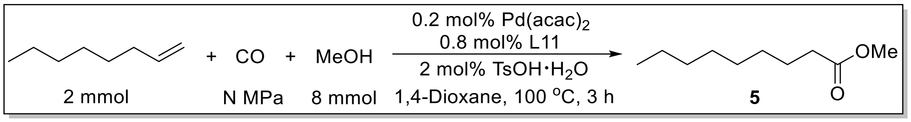

Entry	CO (MPa)	Yield (%)	L/B[b]
1	0.5	56	90/10
2	1	59	91/9
3	2	66	91/9
4	4	49	89/11
5	6	38	87/13

[a] Reaction conditions: 1-octene (2 mmol), MeOH (8 mmol), Pd(acac)$_2$ (0.2 mol %), L11 (0.8 mol%), TsOH·H_2O (2 mol%), CO (N MPa), 1,4-Dioxane (4 ml), 100 ºC, 3 h. GC yields of linear and branched esters are determined based on 1-octene using *n*-decane as the internal standard. [b] L/B (the ratio of linear/branched esters) is determined by GC analysis.

Supplementary Table 4. The effect of temperature.[a]

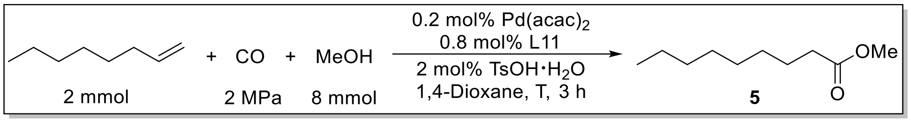

Entry	T (ºC)	Conv. (%)	Selectivity (%)	Selectivity of iso-alkenes (%)	Selectivity of esters (%)	Yield of esters (%)	L/B[b]
1	60	25	>99	12	88	22	84/16
2	80	73	>99	33	67	49	89/11
3	100	92	>99	28	72	66	91/9
4	120	95	>99	47	53	50	90/10
5	140	96	>99	60	40	38	88/12

[a] Reaction conditions: 1-octene (2 mmol), MeOH (8 mmol), $Pd(acac)_2$ (0.2 mol %), L11 (0.8 mol%), $TsOH·H_2O$ (2 mol%), CO (2 MPa), 1,4-Dioxane (4 ml), T, 3 h. Conversion of 1-octene are determined by GC analysis, and GC yields of linear and branched esters are based on 1-octene using *n*-decane as the internal standard. [b] L/B (the ratio of linear/branched esters) is determined by GC analysis.

Supplementary Table 5. The ratio of 1-octene and MeOH.[a]

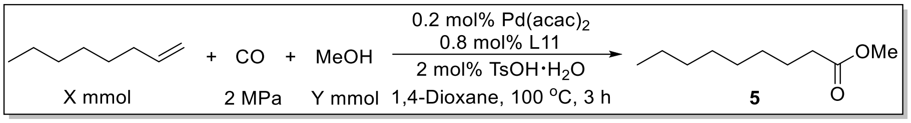

Entry	X/Y	Yield (%)	L/B[b]
1	2/8	66	91/9
2	2/4	46	91/9
3	4/2	44	91/9
4	8/2	70	91/9

[a] Reaction conditions: 1-octene (X mmol), MeOH (Y mmol), $Pd(acac)_2$ (0.2 mol %), L11 (0.8 mol%), $TsOH{\cdot}H_2O$ (2 mol%), CO (2 MPa), 1,4-Dioxane (4 ml), 100 °C, 3 h. GC yields of the mixture of linear and branched esters are determined based on the reagent that was used in low doses (1-octene or MeOH) using *n*-decane as the internal standard. [b] L/B (the ratio of linear/branched esters) is determined by GC analysis.

Supplementary Table 6. The effect of time and kinetic profile.[a]

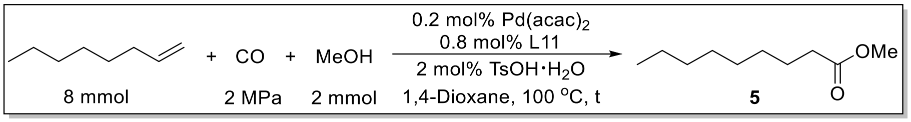

Entry	t (h)	Yield (%)	L/B[b]
1	0.25	4	90/10
2	0.5	11	90/10
3	0.75	26	91/9
4	1	41	90/10
5	1.5	52	91/9
6	2	58	91/9
7	3	70	90/10
8	6	89	91/9
9	12	95	91/9
10	20	95	91/9

[a] Reaction conditions: 1-octene (8 mmol), MeOH (2 mmol), $Pd(acac)_2$ (0.2 mol %), L11 (0.8 mol%), $TsOH{\cdot}H_2O$ (2 mol%), CO (2 MPa), 1,4-Dioxane (4 ml), 100 °C, t. GC yields of the mixture of linear and branched esters are determined based on MeOH using *n*-decane as the internal standard. [b] L/B (the ratio of linear/branched esters) is determined by GC analysis.

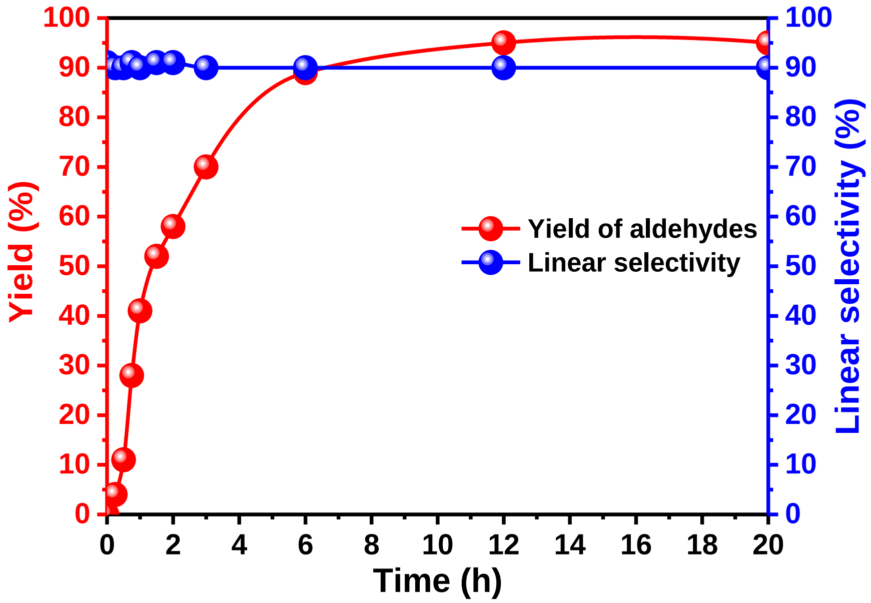

Supplementary Fig. 12 Kinetic profile for Pd-catalyzed methoxycarbonylation of 1-octene.

Supplementary Note 3: Detailed procedure for alkoxycarbonylation or hydroxycarbonylation

For alkoxycarbonylation

For substrates **1**:

An 80 ml steel autoclave was charged with Pd(acac)$_2$ (1.2 mg, 0.2 mol%), **L11** (10.4 mg, 0.8 mol%), PTSA·H$_2$O (7.6 mg, 2 mol%), and THF (4 ml). Then alkene **1** (8 mmol) and methanol (2 mmol) were introduced into the autoclave. After the autoclave was purged with CO (1 MPa) for four times at room temperature and then pressurized with CO to 2 MPa, the autoclave was sealed and put into a preheated reactor, stirring at 100 ^{o}C for 20 h. Afterwards, the autoclave was cooled to room temperature and depressurized slowly. Subsequently, the reaction mixture was diluted with EtOAc (6.0 ml). Finally, the total yield of linear and branched esters was obtained by gas chromatography analysis using *n*-decane as the internal standard, and the regioselectivitiy of linear and branched esters was obtained by gas chromatography analysis.

For substrates **2-4, 7-8, 11, 20, 30-35**:

An 80 ml steel autoclave was charged with Pd(acac)$_2$ (1.2 mg, 0.2 mol%), **L11** (10.4 mg, 0.8 mol%), PTSA·H$_2$O (7.6 mg, 2 mol%), and 1,4-dioxane (4 ml). Then alkenes (8 mmol) and alcohols (2 mmol) were introduced into the autoclave. After the autoclave was purged with CO (1 MPa) for four times at room temperature and then pressurized with CO to 2 MPa, the autoclave was sealed and put into a preheated reactor, stirring at 100 ^{o}C for 20 h. Afterwards, the autoclave was cooled to room temperature and depressurized slowly. Subsequently, the reaction mixture was diluted with EtOAc (6.0 ml). Finally, the total yields of linear and branched esters were obtained by gas chromatography analysis using *n*-decane as the internal standard, and the regioselectivitiies of linear and branched esters were obtained by gas chromatography analysis.

For substrates **5, 6, 9, 10, 13, 21-25, 27, 28, 36-42, 46, 47, 49-62, 64-69, 101-108, 110, 112**:

An 80 ml steel autoclave was charged with Pd(acac)$_2$ (1.2 mg, 0.2 mol%), **L11** (10.4 mg, 0.8 mol%), PTSA·H$_2$O (7.6 mg, 2 mol%), and 1,4-dioxane (4 ml). Then alkenes (8 mmol) and alcohols (2 mmol) were introduced into the autoclave. After the autoclave was purged with CO (1 MPa) for four times at room temperature and then pressurized with CO to 2 MPa, the autoclave was sealed and put into a preheated reactor, stirring at 100 ^{o}C for 20 h. Afterwards, the autoclave was cooled to room temperature and depressurized slowly. Subsequently, the reaction mixture was diluted with EtOAc (6.0 ml). Finally, the total yields of linear and branched esters were obtained by flash column chromatography using EtOAc–petroleum ether mixture (1:100 to 1:3) as an eluent on silica gel, and the regioselectivitiies of linear and branched esters were obtained by gas chromatography analysis.

For substrates **12, 14, 26, 29, 96-100**:

An 80 ml steel autoclave was charged with Pd(acac)$_2$ (1.2 mg, 0.4 mol%), **L11** (10.4 mg, 1.6 mol%), PTSA·H$_2$O (7.6 mg, 4 mol%), and 1,4-dioxane (4 ml). Then alkenes (1 mmol) and methanol (4 mmol) were introduced into the autoclave. After the autoclave was purged with CO (1 MPa) for four times at room temperature and then pressurized with CO to 2 MPa, the autoclave was sealed and put into a preheated reactor, stirring at 100 ^{o}C for 20 h. Afterwards, the autoclave was cooled to room temperature and depressurized slowly. Subsequently, the reaction mixture was diluted with EtOAc (6.0 ml). Finally, the total yields of linear and branched esters were obtained by flash column chromatography using EtOAc–petroleum ether mixture (1:100 to 1:3) as an eluent on silica gel, and the regioselectivitiies of linear and branched esters were obtained by gas chromatography analysis.

For substrates **15-19**:

An 80 ml steel autoclave was charged with Pd(acac)$_2$ (1.2 mg, 0.4 mol%), **L11** (10.4 mg, 1.6 mol%), PTSA·H$_2$O (7.6 mg, 4 mol%), and 1,4-dioxane (4 ml). Then alkenes (1 mmol) and methanol (8 mmol) were introduced into the autoclave. After the autoclave was purged with CO (1 MPa) for four times at room temperature and then pressurized with CO to 2 MPa, the autoclave was sealed and put into a preheated reactor, stirring at 100 ^{o}C for 20 h. Afterwards, the autoclave was cooled to room temperature and depressurized slowly. Subsequently, the reaction mixture was diluted with EtOAc (6.0 ml). Finally, the total yields of linear and branched esters were obtained by flash column chromatography using EtOAc–petroleum ether mixture (1:100 to 1:3) as an eluent on silica gel, and the regioselectivitiies of linear and branched esters were obtained by gas chromatography analysis.

For substrates **43, 48, 63**:

An 80 ml steel autoclave was charged with Pd(acac)$_2$ (1.2 mg, 0.4 mol%), **L11** (10.4 mg, 1.6 mol%), PTSA·H$_2$O (7.6 mg, 4 mol%), and 1,4-dioxane (4 ml). Then 1-octene (8 mmol) and alcohols (1 mmol) were introduced into the autoclave. After the autoclave was purged with CO (1 MPa) for four times at room

temperature and then pressurized with CO to 2 MPa, the autoclave was sealed and put into a preheated reactor, stirring at 100 °C for 20 h. Afterwards, the autoclave was cooled to room temperature and depressurized slowly. Subsequently, the reaction mixture was diluted with EtOAc (6.0 ml). Finally, the total yields of linear and branched esters were obtained by flash column chromatography using EtOAc–petroleum ether mixture (1:100 to 1:3) as an eluent on silica gel, and the regioselectivitiies of linear and branched esters were obtained by gas chromatography analysis.

For substrates **44**:

An 80 ml steel autoclave was charged with $Pd(acac)_2$ (1.2 mg, 0.4 mol%), **L11** (10.4 mg, 1.6 mol%), PTSA·H_2O (7.6 mg, 4 mol%), and 1,4-dioxane (4 ml). Then 1-octene (8 mmol) and alcohols (1 mmol) were introduced into the autoclave. After the autoclave was purged with CO (1 MPa) for four times at room temperature and then pressurized with CO to 2 MPa, the autoclave was sealed and put into a preheated reactor, stirring at 100 °C for 20 h. Afterwards, the autoclave was cooled to room temperature and depressurized slowly. Subsequently, the reaction mixture was diluted with EtOAc (6.0 ml). Finally, the total yield of linear and branched esters was obtained by flash column chromatography using EtOAc–petroleum ether mixture (1:100 to 1:3) as an eluent on silica gel, and the regioselectivitiy of linear and branched esters was obtained by ^{1}H NMR analysis.

For substrates **45**:

An 80 ml steel autoclave was charged with $Pd(acac)_2$ (1.2 mg, 0.4 mol%), **L11** (10.4 mg, 1.6 mol%), PTSA·H_2O (7.6 mg, 4 mol%), and 1,4-dioxane (4 ml). Then 1-octene (12 mmol) and alcohols (1 mmol) were introduced into the autoclave. After the autoclave was purged with CO (1 MPa) for four times at room temperature and then pressurized with CO to 2 MPa, the autoclave was sealed and put into a preheated reactor, stirring at 100 °C for 20 h. Afterwards, the autoclave was cooled to room temperature and depressurized slowly. Subsequently, the reaction mixture was diluted with EtOAc (6.0 ml). Finally, the total yield of linear and branched esters was obtained by flash column chromatography using EtOAc–petroleum ether mixture (1:100 to 1:3) as an eluent on silica gel, and the regioselectivitiy of linear and branched esters was obtained by gas chromatography analysis.

For substrates **83, 84, 86, 88, 91**:

An 80 ml steel autoclave was charged with $Pd(acac)_2$ (6.1 mg, 1 mol%), **L11** (52 mg, 4 mol%), PTSA·H_2O (38 mg, 10 mol%), and 1,4-dioxane (4 ml). Then alkenes (8 mmol) and MeOH (2 mmol) were introduced into the autoclave. After the autoclave was purged with CO (1 MPa) for four times at room temperature and then pressurized with CO to 2 MPa, the autoclave was sealed and put into a preheated reactor, stirring at 110 °C for 16 h. Afterwards, the autoclave was cooled to room temperature and depressurized slowly. Subsequently, the reaction mixture was diluted with EtOAc (6.0 ml). Finally, the total yield of linear and branched esters was obtained by gas chromatography analysis using *n*-decane as the internal standard, and the regioselectivitiy of linear and branched esters was obtained by gas chromatography analysis.

For substrates **85, 87, 89, 93, 94**:

An 80 ml steel autoclave was charged with $Pd(acac)_2$ (6.1 mg, 2 mol%), **L11** (52 mg, 8 mol%), PTSA·H_2O (38 mg, 20 mol%), and 1,4-dioxane (4 ml). Then alkenes (4 mmol) and MeOH (1 mmol) were introduced into the autoclave. After the autoclave was purged with CO (1 MPa) for four times at room temperature and then pressurized with CO to 2 MPa, the autoclave was sealed and put into a preheated reactor, stirring at 120 °C for 16 h. Afterwards, the autoclave was cooled to room temperature and depressurized slowly. Subsequently, t he reaction mixture was diluted with EtOAc (6.0 ml). Finally, the total yield of linear and branched esters was obtained by gas chromatography analysis using *n*-decane as the internal standard for **85** and **87**, and by flash column chromatography using EtOAc–petroleum ether mixture (1:100 to 1:3) as an eluent on silica gel for **90**, **93**, and **94**, and the regioselectivitiy of linear d branched esters was obtained by gas chromatography analysis.

For substrates **90, 92**:

An 80 ml steel autoclave was charged with $Pd(acac)_2$ (6.1 mg, 1 mol%), **L11** (52 mg, 4 mol%), PTSA·H_2O (38 mg, 10 mol%), and 1,4-dioxane (4 ml). Then alkenes (8 mmol) and MeOH (2 mmol) were introduced into the autoclave. After the autoclave was purged with CO (1 MPa) for four times at room temperature and then pressurized with CO to 2 MPa, the autoclave was sealed and put into a preheated reactor, stirring at 120 °C for 24 h. Afterwards, the autoclave was cooled to room temperature and depressurized slowly. Subsequently, the reaction mixture was diluted with EtOAc (6.0 ml). Finally, the total yield of linear and branched esters was obtained by gas chromatography analysis using *n*-decane as the internal standard for **91**, and by ^{1}H NMR analysis using triphenylmethane as the internal standard for **92**, and the regioselectivitiy of linear and branched esters was obtained by gas chromatography analysis.

For substrates **95**:

An 80 ml steel autoclave was charged with $Pd(acac)_2$ (12.2 mg, 4 mol%), **L11** (104 mg, 16 mol%), PTSA·H_2O (76 mg, 40 mol%), and THF (4 ml). Then 1, 3-butadiene (1 mmol, 2 M in THF, 0.5 ml) and MeOH (8 mmol) were introduced into the autoclave. After the autoclave was purged with CO (1 MPa) for four times at room temperature and then pressurized with CO to 4 MPa, the autoclave was sealed and put into a preheated reactor, stirring at 130 °C for 36 h. Afterwards, the autoclave was cooled to room temperature and depressurized slowly. Subsequently, the reaction mixture was diluted with EtOAc (6.0 ml). Finally, the total yield of linear and branched esters was obtained by ^{1}H NMR analysis using triphenylmethane as the internal standard, and the regioselectivitiy of linear and branched esters was obtained by gas chromatography analysis.

For substrates **109, 111**:

An 80 ml steel autoclave was charged with $Pd(acac)_2$ (1.2 mg, 0.4 mol%), **L11** (10.4 mg, 1.6 mol%), PTSA·H_2O (7.6 mg, 4 mol%), and 1,4-dioxane (4 ml). Then 1-octene (4 mmol) and alcohols (1 mmol) were introduced into the autoclave. After the autoclave was purged with CO (1 MPa) for four times at room temperature and then pressurized with CO to 2 MPa, the autoclave was sealed and put into a preheated reactor, stirring at 100 °C for 20 h. Afterwards, the autoclave was cooled to room temperature and depressurized slowly. Subsequently, the reaction mixture was diluted with EtOAc (6.0 ml). Finally, the total yields of linear and branched esters were obtained by flash column chromatography using EtOAc–petroleum ether mixture (1:100 to 1:3) as an eluent on silica gel, and the regioselectivitiies of linear and branched esters were obtained by liquid chromatography analysis.

For substrates **120, 121**:

An 80 ml steel autoclave was charged with $Pd(acac)_2$ (4.8 mg, 0.4 mol%), **L11** (42 mg, 1.6 mol%), PTSA·H_2O (15.2 mg, 4 mol%), and 1,4-dioxane (8 ml). Then alkenes (16 mmol) and alcohols (4 mmol) were introduced into the autoclave. After the autoclave was purged with CO (1 MPa) for four times at room temperature and then pressurized with CO to 2 MPa, the autoclave was sealed and put into a preheated reactor, stirring at 110 °C for 36 h. Afterwards, the autoclave was cooled to room temperature and depressurized slowly. Subsequently, the reaction mixture was diluted with EtOAc (6.0 ml). Finally, the total yield of linear and branched esters was obtained by flash column chromatography using EtOAc–petroleum ether mixture (1:100 to 1:3) as an eluent on silica gel, and the regioselectivitiy of linear and branched esters was obtained by gas chromatography analysis.

For substrates **122**:

An 80 ml steel autoclave was charged with $Pd(acac)_2$ (1.2 mg, 0.2 mol%), **L11** (10.4 mg, 0.8 mol%), PTSA·H_2O (7.6 mg, 2 mol%), and 1,4-dioxane (4 ml). Then 1-hexene (8 mmol) and Trenbolone (2 mmol) were introduced into the autoclave. After the autoclave was purged with CO (1 MPa) for four times at room temperature and then pressurized with CO to 2 MPa, the autoclave was sealed and put into a preheated reactor, stirring at 100 °C for 20 h. Afterwards, the autoclave was cooled to room temperature and depressurized slowly. Subsequently, the reaction mixture was diluted with EtOAc (6.0 ml). Finally, the total yield of linear and branched esters was obtained by flash column chromatography using EtOAc–petroleum ether mixture (1:100 to 1:3) as an eluent on silica gel, and the regioselectivitiy of linear and branched esters was obtained by gas chromatography analysis.

For hydroxycarbonylation

For substrates **70-73, 79-82**:

An 80 ml steel autoclave was charged with $Pd(acac)_2$ (1.2 mg, 0.2 mol%), **L11** (10.4 mg, 0.8 mol%), PTSA·H_2O (7.6 mg, 2 mol%), and 1,4-dioxane (4 ml). Then alkenes (8 mmol) and H_2O (2 mmol) were introduced into the autoclave. After the autoclave was purged with CO (1 MPa) for four times at room temperature and then pressurized with CO to 2 MPa, the autoclave was sealed and put into a preheated reactor, stirring at 100 °C for 20 h. Afterwards, the autoclave was cooled to room temperature and depressurized slowly. Subsequently, the reaction mixture was diluted with EtOAc (6.0 ml). Finally, the total yields of linear and branched acids were obtained by acid-base extraction, and the regioselectivities of linear and branched acids were obtained by gas chromatography analysis.

For substrates **74-78**:

An 80 ml steel autoclave was charged with $Pd(acac)_2$ (1.2 mg, 0.4 mol%), **L11** (10.4 mg, 1.6 mol%), PTSA·H_2O (7.6 mg, 4 mol%), and 1,4-dioxane (4 ml). Then alkenes (1 mmol) and H_2O (8 mmol) were introduced into the autoclave. After the autoclave was purged with CO (1 MPa) for four times at room temperature and then pressurized with CO to 2 MPa, the autoclave was sealed and put into a preheated reactor, stirring at 100 °C for 20 h. Afterwards, the autoclave was cooled to room temperature and depressurized slowly. Subsequently, the reaction mixture was diluted with EtOAc (6.0 ml). Finally, the total yields of linear

and branched acids were obtained by acid-base extraction, and the regioselectivities of linear and branched acids were obtained by ^{1}H NMR analysis.

For substrates **113-117**:

An 80 ml steel autoclave was charged with $Pd(acac)_2$ (1.2 mg, 0.4 mol%), **L11** (10.4 mg, 1.6 mol%), PTSA·H_2O (7.6 mg, 4 mol%), and 1,4-dioxane (4 ml). Then alkenes (1 mmol) and H_2O (4 mmol) were introduced into the autoclave. After the autoclave was purged with CO (1 MPa) for four times at room temperature and then pressurized with CO to 2 MPa, the autoclave was sealed and put into a preheated reactor, stirring at 100 °C for 20 h. Afterwards, the autoclave was cooled to room temperature and depressurized slowly. Subsequently, the reaction mixture was diluted with EtOAc (6.0 ml). Finally, the total yields of linear and branched acids were obtained by acid-base extraction, and the regioselectivities of linear and branched were obtained by ^{1}H NMR analysis.

For substrates **118-119**:

An 80 ml steel autoclave was charged with $Pd(acac)_2$ (1.2 mg, 0.4 mol%), **L11** (10.4 mg, 1.6 mol%), PTSA·H_2O (7.6 mg, 4 mol%), and 1,4-dioxane (4 ml). Then alkenes (1 mmol) and H_2O (4 mmol) were introduced into the autoclave. After the autoclave was purged with CO (1 MPa) for four times at room temperature and then pressurized with CO to 2 MPa, the autoclave was sealed and put into a preheated reactor, stirring at 100 °C for 20 h. Afterwards, the autoclave was cooled to room temperature and depressurized slowly. Subsequently, the reaction mixture was diluted with EtOAc (6.0 ml). Finally, the total yields of linear and branched acids were obtained by acid-base extraction, and the regioselectivities of linear and branched acids were obtained by gas chromatography analysis.

Supplementary Note 4: NMR data of products

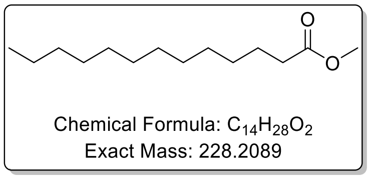

Methyl tridecanoate (5): yellow oil liquid, 416 mg, 91% yield, 90% linear selectivity. **^{1}H NMR** (400 MHz, $CDCl_3$) δ 3.66 (s, 3H), 2.30 (t, *J* = 7.5 Hz, 2H), 1.61 (dd, *J* = 14.2, 7.0 Hz, 2H), 1.27 (d, *J* = 10.0 Hz, 18H), 0.88 (t, *J* = 6.6 Hz, 3H). **^{13}C NMR** (101 MHz, $CDCl_3$) δ 174.33, 51.43, 34.18, 32.02, 29.75, 29.73, 29.70, 29.56, 29.45, 29.37, 29.26, 25.05, 22.78, 14.17.[11]

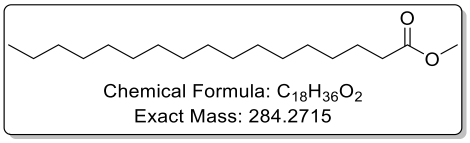

Methyl heptadecanoate (6): colorless oil liquid, 550 mg, 97% yield, 89% linear selectivity. **^{1}H NMR** (400 MHz, $CDCl_3$) δ 3.58 (s, 3H), 2.22 (t, *J* = 7.5 Hz, 2H), 1.53 (dd, *J* = 14.2, 7.1 Hz, 2H), 1.19 (s, 26H), 0.80 (t, *J* = 6.7 Hz, 3H). **^{13}C NMR** (101 MHz, $CDCl_3$) δ 174.18, 51.33, 34.12, 32.02, 29.79, 29.77, 29.76, 29.75, 29.69, 29.61, 29.59, 29.56, 29.47, 29.36, 29.25, 25.03, 22.77, 14.12. **HRMS** (ESI)**:** Calcd. for $C_{18}H_{36}O_2Na$ $[M+Na]^+$: 307.2609, found: 307.2608.

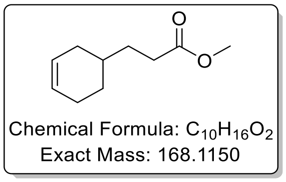

Methyl 3-(cyclohex-3-en-1-yl)propanoate (9): colorless oil liquid, 300 mg, 89% yield, 97% linear selectivity. **^{1}H NMR** (400 MHz, $CDCl_3$) δ 5.57 (s, 2H), 3.60 (s, 3H), 2.28 (t, *J* = 7.7 Hz, 2H), 2.14 – 1.82 (m, 3H), 1.77 – 1.38 (m, 5H), 1.33 – 1.00 (m, 1H). **^{13}C NMR** (101 MHz, $CDCl_3$) δ 174.44, 127.08, 126.21, 51.55, 33.17, 31.76, 31.62, 31.53, 28.61, 25.14. **HRMS** (ESI)**:** Calcd. for $C_{10}H_{16}O_2Na$ $[M+Na]^+$: 191.1048, found: 191.1043.

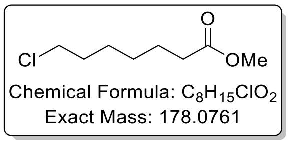

Methyl 7-chloroheptanoate (10): colorless oil liquid, 348 mg, 98% yield, 90% linear selectivity. **^{1}H NMR** (400 MHz, $CDCl_3$) δ 3.67 (s, 3H), 3.53 (t, *J* = 6.7 Hz, 2H), 2.32 (t, *J* = 7.5 Hz, 2H), 1.86 – 1.70 (m, 2H), 1.71 – 1.54 (m, 2H), 1.54 – 1.41 (m, 2H), 1.40 – 1.29 (m, 2H). **^{13}C NMR** (101 MHz, $CDCl_3$) δ 174.13, 51.53, 45.01, 33.97, 32.44, 28.43, 26.57, 24.80.[12]

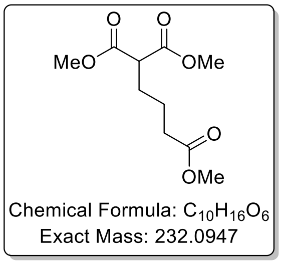

Trimethyl butane-1,1,4-tricarboxylate (12): colorless oil liquid, 216 mg, 93% yield, 92% linear selectivity. **^{1}H NMR** (400 MHz, $CDCl_3$) δ 3.75 (s, 6H), 3.67 (s, 3H), 3.39 (t, *J* = 7.5 Hz, 1H), 2.36 (t, *J* = 7.4 Hz, 2H), 1.98 – 1.88 (m, 2H), 1.73 – 1.59 (m, 2H). **^{13}C NMR** (101 MHz, $CDCl_3$) δ 173.34, 169.54, 52.52, 51.56, 51.34, 33.49, 28.17, 22.62. **HRMS** (ESI)**:** Calcd. for $C_{10}H_{16}O_6Na$ $[M+Na]^+$: 255.0839, found: 255.0838.

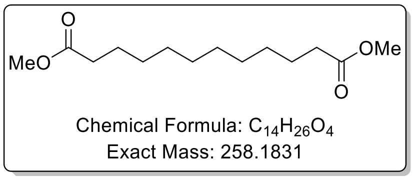

Dimethyl dodecanedioate (13): colorless oil liquid, 362 mg, 70% yield, 90% linear selectivity. **^{1}H NMR** (400 MHz, $CDCl_3$) δ 3.66 (s, 6H), 2.30 (t, *J* = 7.5 Hz, 4H), 1.72 – 1.44 (m, 4H), 1.28 (s, 12H). **^{13}C NMR** (101 MHz, $CDCl_3$) δ 174.33, 51.45, 34.14, 29.40, 29.26, 29.17, 24.99.[12]

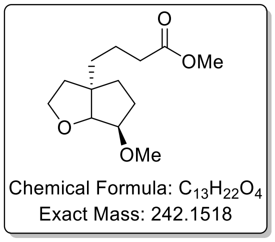

Methyl 4-((*3aR,6R*)-6-methoxyhexahydro-*3aH*-cyclopenta[*b*]furan-3a-yl)butanoate (14): colorless oil liquid, 200 mg, 82% yield, 98% linear selectivity. **^{1}H NMR** (400 MHz, $CDCl_3$) δ 3.84 (td, *J* = 8.1, 4.6 Hz, 1H), 3.77 (dt, *J* = 15.6, 7.8 Hz, 1H), 3.67 (s, 3H), 3.29 (s, 3H), 2.34 (t, *J* = 7.5 Hz, 2H), 2.12 – 2.04 (m, 1H), 1.96 – 1.83 (m, 1H), 1.78 – 1.41 (m, 9H), 1.29 (td, *J* = 12.8, 4.6 Hz, 1H). **^{13}C NMR** (101 MHz, $CDCl_3$) δ 174.26, 118.06, 66.12, 54.36, 51.56, 50.81, 38.00, 36.76, 34.92, 34.81, 33.76, 21.60, 21.44. **HRMS** (ESI)**:** Calcd. for $C_{13}H_{22}O_4Na$ $[M+Na]^+$: 265.1416, found: 265.1481.

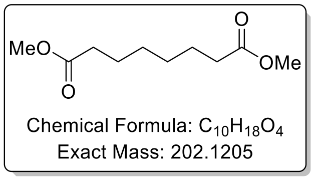

Dimethyl octanedioate (15): colorless oil liquid, 141 mg, 70% yield, 85% linear selectivity. **^{1}H NMR** (400 MHz, $CDCl_3$) δ 3.67 (s, 6H), 2.31 (t, *J* = 7.5 Hz, 4H), 1.77 – 1.53 (m, 4H), 1.43 – 1.27 (m, 4H). **^{13}C NMR** (101 MHz, $CDCl_3$) δ 174.18, 51.48, 34.02, 28.80, 24.79.[13]

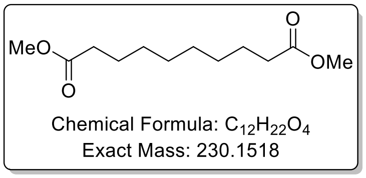

Dimethyl decanedioate (16): colorless oil liquid, 180 mg, 78% yield, 72% linear selectivity. **^{1}H NMR** (400 MHz, $CDCl_3$) δ 3.67 (s, 6H), 2.30 (t, *J* = 7.5 Hz, 4H), 1.76 – 1.44 (m, 4H), 1.30 (s, 8H). **^{13}C NMR** (101 MHz, $CDCl_3$) δ 174.31, 51.48, 34.11, 29.10, 24.96.[14]

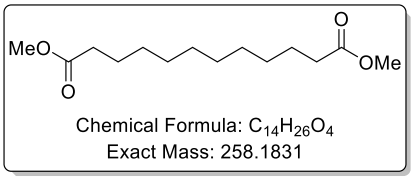

Dimethyl dodecanedioate (17): colorless oil liquid, 194 mg, 75% yield, 71% linear selectivity. **^{1}H NMR** (400 MHz, $CDCl_3$) δ 3.67 (s, 6H), 2.30 (t, *J* = 7.5 Hz, 4H), 1.73 – 1.48 (m, 4H), 1.28 (s, 12H). **^{13}C NMR** (101 MHz, $CDCl_3$) δ 174.26, 51.39, 34.07, 29.35, 29.22, 29.12, 24.94.[15]

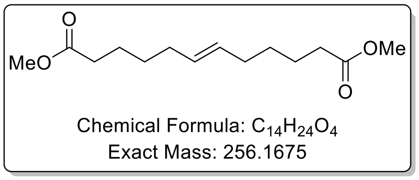

Dimethyl (E)-dodec-6-enedioate (18): colorless oil liquid, 205 mg, 80% yield, 81% linear selectivity. **^{1}H NMR** (400 MHz, $CDCl_3$) δ 5.57 – 4.94 (m, 2H), 3.67 (s, 6H), 2.59 – 2.15 (m, 4H), 2.17 – 1.85 (m, 4H), 1.80 – 1.51 (m, 4H), 1.47 – 1.25 (m, 4H). **^{13}C NMR** (101 MHz, $CDCl_3$) δ 174.17, 130.17, 51.44, 33.94, 32.16, 29.02, 24.44. **HRMS** (ESI)**:** Calcd. for $C_{14}H_{24}O_4Na$ $[M+Na]^+$: 279.1567, found: 279.1571.

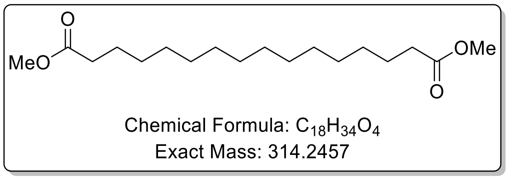

Dimethyl hexadecanedioate (19): white solid, 238 mg, 76% yield, 73% linear selectivity. **^{1}H NMR** (400 MHz, $CDCl_3$) δ 3.66 (s, 6H), 2.30 (t, *J* = 7.6 Hz, 4H), 1.70 – 1.50 (m, 4H), 1.27 (d, *J* = 12.3 Hz, 20H). **^{13}C NMR** (101 MHz, $CDCl_3$) δ 174.25, 51.38, 34.09, 29.64, 29.60, 29.47, 29.28, 29.17, 24.97.[16]

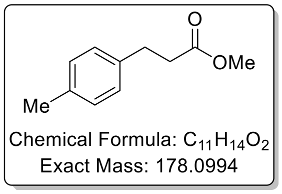

Methyl 3-(*p*-tolyl)propanoate (21): colorless oil liquid, 320 mg, 90% yield, 85% linear selectivity. **^{1}H NMR** (400 MHz, $CDCl_3$) δ 7.07 (s, 4H), 3.64 (s, 3H), 2.90 (t, *J* = 7.8 Hz, 2H), 2.59 (t, *J* = 7.8 Hz, 2H), 2.30 (s, 3H). **^{13}C NMR** (101 MHz, $CDCl_3$) δ 173.38, 137.48, 135.73, 129.21, 128.17, 51.54, 35.85, 30.55, 21.00.[17]

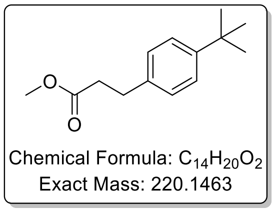

Methyl 3-(4-(*tert*-butyl)phenyl)propanoate (22): colorless oil liquid, 400 mg, 91% yield, 86% linear selectivity. **^{1}H NMR** (400 MHz, $CDCl_3$) δ 7.37 – 7.26 (m, 2H), 7.12 (d, *J* = 8.2 Hz, 2H), 3.64 (d, *J* = 8.3 Hz, 3H), 3.03 – 2.74 (m, 2H), 2.69 – 2.41 (m, 2H), 1.30 (s, 9H). **^{13}C NMR** (101 MHz, $CDCl_3$) δ 175.26 (iso), 173.52 (n), 150.00 (iso), 149.15 (n), 137.53 (n and iso), 128.01 (n), 127.17 (iso), 125.63 (iso), 125.49 (n), 52.02 (iso), 51.65 (n,), 45.00 (iso), 35.76 (n), 34.52 (iso), 34.46 (n), 31.47 (n), 31.43 (iso), 30.48 (n), 18.69 (iso, $CHCH_3$).[18]

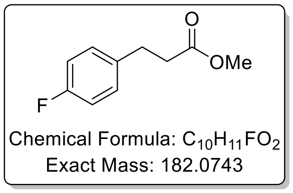

Methyl 3-(4-fluorophenyl)propanoate (23): colorless oil liquid, 280 mg, 77% yield, 86% linear selectivity. **^{1}H NMR** (400 MHz, $CDCl_3$) δ 7.21 – 7.05 (m, 2H), 7.05 – 6.81 (m, 2H), 3.65 (s, 3H), 2.91 (t, *J* = 7.7 Hz, 2H), 2.60 (t, *J* = 7.7 Hz, 2H). **^{13}C NMR** (101 MHz, $CDCl_3$) δ 173.19, 161.56 (d, *J* = 244.0 Hz), 136.22 (d, *J* = 3.2 Hz), 129.77 (d, *J* = 7.9 Hz), 115.31 (d, *J* = 21.2 Hz), 51.65, 35.81, 30.17. **^{19}F NMR** (376 MHz, $CDCl_3$) δ -117.04.[18]

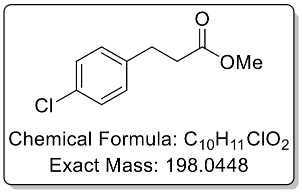

Methyl 3-(4-chlorophenyl)propanoatemethyl (24): yellowish oil liquid, 345 mg, 87% yield, 86% linear selectivity. **^{1}H NMR** (400 MHz, $CDCl_3$) δ 7.27 – 7.21 (m, 2H), 7.12 (d, *J* = 8.4 Hz, 2H), 3.66 (s, 3H), 2.91

(t, *J* = 7.7 Hz, 2H), 2.60 (t, *J* = 7.7 Hz, 2H). ^{13}**C NMR** (101 MHz, $CDCl_3$) δ 173.12, 139.05, 132.14, 129.77, 128.70, 51.75, 35.58, 30.34.[18]

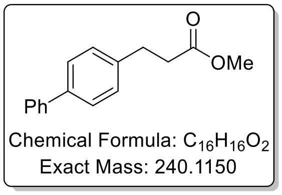

Methyl 3-([1,1'-biphenyl]-4-yl)propanoate (25): colorless oil liquid, 460 mg, 96% yield, 83% linear selectivity. ^{1}**H NMR** (400 MHz, $CDCl_3$) δ 7.53 (dd, *J* = 20.2, 7.9 Hz, 4H), 7.41 (t, *J* = 7.5 Hz, 2H), 7.36 – 7.20 (m, 3H), 3.67 (s, 3H), 2.98 (t, *J* = 7.8 Hz, 2H), 2.66 (t, *J* = 7.8 Hz, 2H). ^{13}C NMR (101 MHz, $CDCl_3$) δ 173.40, 141.02, 139.71, 139.34, 128.83, 128.81, 127.34, 127.22, 127.09, 51.73, 35.71, 30.65.[19]

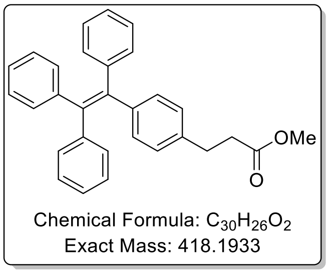

Methyl 3-(4-(1,2,2-triphenylvinyl)phenyl)propanoate (26): colorless viscous liquid, 334 mg, 80% yield, 88% linear selectivity. ^{1}**H NMR** (400 MHz, $CDCl_3$) δ 7.09 – 6.98 (m, 15H), 6.91 (q, *J* = 8.2 Hz, 4H), 3.60 (s, 3H), 2.84 (t, *J* = 7.8 Hz, 2H), 2.65 – 2.45 (m, 2H). ^{13}**C NMR** (101 MHz, $CDCl_3$) δ 173.31, 143.83, 143.81, 141.72, 140.82, 140.75, 138.65, 131.49, 131.37, 127.68, 127.58, 126.44, 126.40, 51.60, 35.58, 30.67. **HRMS** (ESI)**:** Calcd. for $C_{30}H_{26}O_2Na$ $[M+Na]^+$: 441.1830, found: 441.1793.

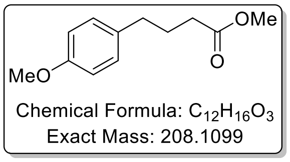

Methyl 4-(4-methoxyphenyl)butanoate (27): colorless oil liquid, 210 mg, 51% yield, 96% linear selectivity. ^{1}**H NMR** (400 MHz, $CDCl_3$) δ 7.09 (d, *J* = 8.5 Hz, 2H), 6.82 (d, *J* = 8.6 Hz, 2H), 3.77 (s, 3H), 3.65 (s, 3H), 2.58 (t, *J* = 7.5 Hz, 2H), 2.31 (t, *J* = 7.5 Hz, 2H), 1.92 (p, *J* = 7.5 Hz, 2H). ^{13}**C NMR** (101 MHz, $CDCl_3$) δ 174.07, 157.99, 133.51, 129.46, 113.88, 55.31, 51.55, 34.29, 33.41, 26.81.[20]

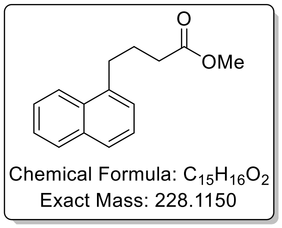

Methyl 4-(naphthalen-1-yl)butanoate (28): yellow oil liquid, 365 mg, 80% yield, 94% linear selectivity. ^{1}**H NMR** (400 MHz, $CDCl_3$) δ 8.03 (d, *J* = 8.3 Hz, 1H), 7.80 (d, *J* = 7.8 Hz, 1H), 7.67 (d, *J* = 8.1 Hz, 1H), 7.56 – 7.38 (m, 2H), 7.34 (t, *J* = 7.6 Hz, 1H), 7.26 (d, *J* = 6.8 Hz, 1H), 3.62 (s, 3H), 3.24 – 2.84 (m, 2H), 2.36 (t, *J* = 7.3 Hz, 2H), 2.21 – 1.82 (m, 2H). ^{13}**C NMR** (101 MHz, $CDCl_3$) δ 173.86, 137.52, 133.94, 131.86, 128.78, 126.85, 126.17, 125.88, 125.50, 125.49, 123.78, 51.47, 33.64, 32.30, 25.78.[21]

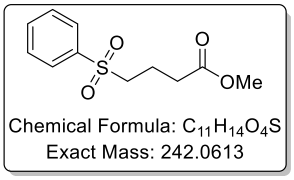

Methyl 3-(phenylsulfonyl)propanoate (29): colorless oil liquid, 155 mg, 64% yield, 90% linear selectivity. ^{1}**H NMR** (400 MHz, $CDCl_3$) δ 7.92 (d, *J* = 8.0 Hz, 2H), 7.68 (t, *J* = 7.4 Hz, 1H), 7.61 – 7.54 (m, 2H), 3.65

(s, 3H), 3.19 (t, *J* = 8.7, 6.8 Hz, 2H), 2.47 (t, *J* = 7.1 Hz, 2H), 2.20 – 1.89 (m, 2H). **^{13}C NMR** (101 MHz, $CDCl_3$) δ 172.56, 138.92, 133.87, 129.41, 128.08, 55.12, 51.83, 31.97, 18.30. **HRMS** (ESI)**:** Calcd. for $C_{11}H_{14}O_4SNa$ $[M+Na]^+$: 265.0505, found: 265.0504.

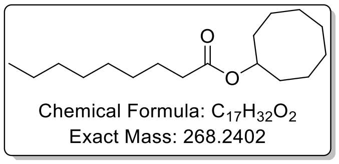

Cyclooctyl nonanoate (36): yellowish oil liquid, 530 mg, 99% yield, 88% linear selectivity. **^{1}H NMR** (400 MHz, $CDCl_3$) δ 4.94 (tt, *J* = 7.9, 3.8 Hz, 1H), 2.25 (t, *J* = 7.5 Hz, 2H), 1.85 – 1.65 (m, 6H), 1.59 (dd, *J* = 20.1, 12.8 Hz, 10H), 1.28 (d, *J* = 5.4 Hz, 10H), 0.88 (t, *J* = 6.2 Hz, 3H). **^{13}C NMR** (101 MHz, $CDCl_3$) δ 173.10, 74.63, 39.77, 34.78, 31.84, 31.57, 29.27, 29.15, 27.12, 25.41, 25.11, 22.96, 22.65, 14.05. **HRMS** (ESI)**:** Calcd. for $C_{17}H_{32}O_2Na$ $[M+Na]^+$: 291.2304, found: 291.2295.

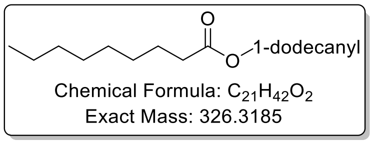

Dodecyl nonanoate (37): colorless oil liquid, 648 mg, 99% yield, 90% linear selectivity. **^{1}H NMR** (400 MHz, $CDCl_3$) δ 4.05 (t, *J* = 6.7 Hz, 2H), 2.28 (t, *J* = 7.5 Hz, 2H), 1.61 (dd, *J* = 13.3, 6.5 Hz, 4H), 1.26 (s, 28H), 0.97 – 0.77 (m, 6H). **^{13}C NMR** (101 MHz, $CDCl_3$) δ 173.93, 64.41, 34.46, 32.03, 31.93, 29.76, 29.75, 29.69, 29.65, 29.47, 29.37, 29.35, 29.27, 29.25, 28.78, 26.06, 25.13, 22.78, 22.74, 14.16, 14.13.[22]

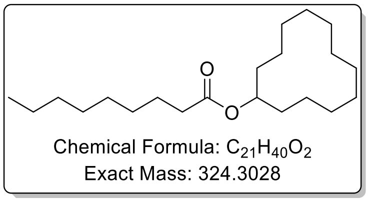

Cyclododecyl nonanoate (38): yellowish oil liquid, 638 mg, 98% yield, 90% linear selectivity. **^{1}H NMR** (400 MHz, $CDCl_3$) δ 5.10 – 4.89 (m, 1H), 2.25 (td, *J* = 7.5, 2.6 Hz, 2H), 1.77 – 1.54 (m, 4H), 1.40 (ddd, *J* = 41.1, 22.1, 6.0 Hz, 30H), 0.88 (t, *J* = 5.5 Hz, 3H). **^{13}C NMR** (101 MHz, $CDCl_3$) δ 173.31, 71.66, 39.76, 34.62, 31.82, 29.25, 29.15, 25.08, 24.05, 23.82, 23.39, 23.21, 22.63, 20.93, 14.02. **HRMS** (ESI)**:** Calcd. for $C_{21}H_{40}O_2Na$ $[M+Na]^+$: 347.2919, found: 347.2921.

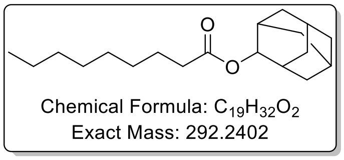

Adamantan-2-yl nonanoate (39): yellowish oil liquid, 628 mg, 87% yield, 86% linear selectivity. **^{1}H NMR** (400 MHz, $CDCl_3$) δ 4.93 (s, 1H), 2.32 (td, *J* = 7.6, 1.4 Hz, 2H), 2.10 – 1.92 (m, 4H), 1.90 – 1.70 (m, 8H), 1.64 (dd, *J* = 13.8, 6.8 Hz, 2H), 1.56 (d, *J* = 12.2 Hz, 2H), 1.29 (d, *J* = 11.7 Hz, 10H), 0.88 (t, *J* = 6.2 Hz, 3H). **^{13}C NMR** (101 MHz, $CDCl_3$) δ 173.18, 76.63, 37.46, 36.38, 34.89, 31.94, 31.83, 29.28, 29.20, 29.19, 27.33, 27.09, 25.24, 22.67, 14.07. **HRMS** (ESI)**:** Calcd. for $C_{19}H_{32}O_2Na$ $[M+Na]^+$: 315.2295, found: 315.2295.

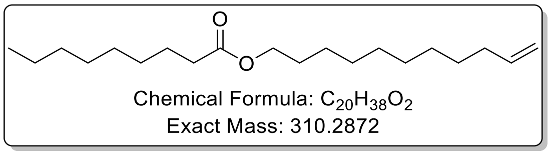

Undec-10-en-1-yl nonanoate (40): colorless oil liquid, 372 mg, 60% yield, 92% linear selectivity. **^{1}H NMR** (400 MHz, $CDCl_3$) δ 5.93 – 5.59 (m, 1H), 4.96 (dd, *J* = 24.3, 13.6 Hz, 2H), 4.05 (t, *J* = 6.7 Hz, 2H), 2.29 (t, *J* = 7.5 Hz, 2H), 2.13 – 1.94 (m, 4H), 1.76 – 1.48 (m, 4H), 1.32 (s, 20H), 0.88 (t, *J* = 6.6 Hz, 3H). **^{13}C NMR** (101 MHz, $CDCl_3$) δ 174.16, 139.33, 114.26, 64.52, 34.56, 33.94, 31.95, 29.60, 29.53, 29.37, 29.30, 29.27, 29.24, 29.05, 28.79, 26.07, 25.17, 22.78, 21.15, 14.22. **HRMS** (ESI)**:** Calcd. for $C_{20}H_{38}O_2Na$ $[M+Na]^+$: 333.2754, found: 333.2764.

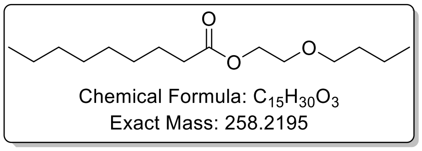

2-Butoxyethyl nonanoate (41): colorless oil liquid, 500 mg, 97% yield, 90% linear selectivity. **^{1}H NMR** (400 MHz, $CDCl_3$) δ 4.31 – 4.13 (m, 2H), 3.68 – 3.57 (m, 2H), 3.47 (t, *J* = 6.6 Hz, 2H), 2.33 (t, *J* = 7.5 Hz, 2H), 1.71 – 1.50 (m, 4H), 1.45 – 1.21 (m, 12H), 0.97 – 0.83 (m, 6H). **^{13}C NMR** (101 MHz, $CDCl_3$) δ 173.86, 71.16, 68.66, 63.47, 34.29, 31.88, 31.73, 29.30, 29.19, 25.00, 22.70, 19.30, 14.11, 13.91.[23]

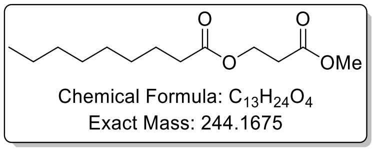

3-Methoxy-3-oxopropyl nonanoate (42): colorless oil liquid, 420 mg, 86% yield, 89% linear selectivity. **^{1}H NMR** (400 MHz, $CDCl_3$) δ 4.34 (t, *J* = 6.3 Hz, 2H), 3.71 (s, 3H), 2.65 (t, *J* = 6.3 Hz, 2H), 2.29 (t, *J* = 7.5 Hz, 2H), 1.72 – 1.50 (m, 2H), 1.28 (s, 10H), 0.88 (t, *J* = 6.6 Hz, 3H). **^{13}C NMR** (101 MHz, $CDCl_3$) δ 173.64, 171.21, 59.65, 51.88, 34.22, 33.80, 31.88, 29.28, 29.18, 29.16, 24.97, 22.71, 14.13. **HRMS** (ESI)**:** Calcd. for $C_{13}H_{24}O_4Na$ $[M+Na]^+$: 267.1568, found: 267.1567.

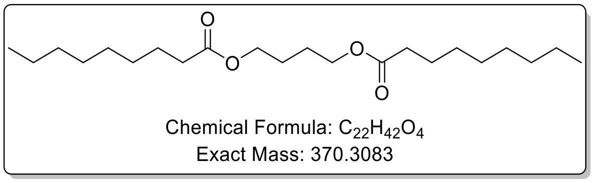

Butane-1,4-diyl dinonanoate (43): yellowish oil liquid, 368 mg, 99% yield, 82% linear selectivity. **^{1}H NMR** (400 MHz, $CDCl_3$) δ 4.02 (d, *J* = 1.8 Hz, 4H), 2.23 (ddd, *J* = 15.0, 7.3, 2.0 Hz, 4H), 1.63 (d, *J* = 1.9 Hz, 4H), 1.54 (s, 4H), 1.18 (dd, *J* = 20.2, 4.2 Hz, 20H), 0.80 (dd, *J* = 6.8, 4.9 Hz, 6H). **^{13}C NMR** (101 MHz, $CDCl_3$) δ 173.82, 63.69, 34.27, 31.80, 29.22, 29.15, 29.12, 25.36, 24.96, 22.62, 14.03. **HRMS** (ESI)**:** $C_{22}H_{42}O_4Na$ $[M+Na]^+$: 393.2979, found: 393.2975.

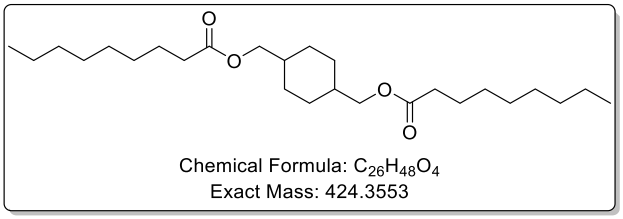

Cyclohexane-1,4-diylbis(methylene) dinonanoate (44): yellowish oil liquid, 800 mg, 94% yield, 86% linear selectivity. **^{1}H NMR** (400 MHz, $CDCl_3$) δ 3.90 (d, *J* = 6.5 Hz, 4H), 2.29 (t, *J* = 7.5 Hz, 4H), 1.78 (t, *J* = 20.8 Hz, 3H), 1.72 – 1.49 (m, 6H), 1.43 (dd, *J* = 17.1, 11.0 Hz, 2H), 1.28 (d, *J* = 6.9 Hz, 20H), 1.10 – 0.95 (m, 3H), 0.88 (t, *J* = 6.5 Hz, 6H). **^{13}C NMR** (101 MHz, $CDCl_3$) δ 173.87, 69.13, 66.94, 37.15, 34.37, 31.84, 29.25, 29.20, 29.17, 28.91, 25.36, 25.07, 22.67, 14.10. **HRMS** (ESI)**:** Calcd. for $C_{26}H_{48}O_4Na$ $[M+Na]^+$: 447.3440, found: 447.3445.

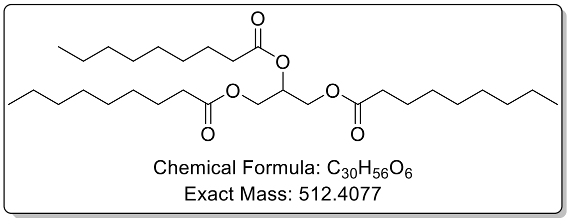

Propane-1,2,3-triyl trinonanoate (45): colorless oil liquid, 442 mg, 86% yield, 75% linear selectivity. **^{1}H NMR** (400 MHz, $CDCl_3$) δ 5.46 – 5.02 (m, 1H), 4.30 (dd, *J* = 11.9, 4.3 Hz, 2H), 4.15 (dd, *J* = 11.9, 6.0 Hz, 2H), 2.51 – 2.08 (m, 6H), 1.73 – 1.49 (m, 6H), 1.28 (d, *J* = 5.0 Hz, 30H), 0.88 (t, *J* = 6.7 Hz, 9H). **^{13}C NMR** (101 MHz, $CDCl_3$) δ 173.36, 172.95, 68.98, 62.20, 34.15, 31.91, 29.32, 29.22, 29.21, 24.96, 22.74, 14.17. **HRMS** (ESI)**:** Calcd. for $C_{30}H_{56}O_6Na$ $[M+Na]^+$: 535.3967, found: 535.3969.

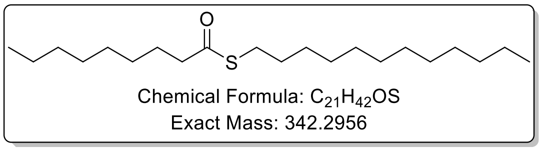

***S*-Dodecyl nonanethioate (46):** colorless oil liquid, 384 mg, 56% yield, 93% linear selectivity. **^{1}H NMR** (400 MHz, $CDCl_3$) δ 2.86 (t, *J* = 7.3 Hz, 2H), 2.53 (t, *J* = 7.5 Hz, 2H), 1.71 – 1.60 (m, 2H), 1.60 – 1.50 (m, 2H), 1.26 (s, 28H), 0.88 (t, *J* = 6.5 Hz, 6H). **^{13}C NMR** (101 MHz, $CDCl_3$) δ 199.96, 44.30, 32.06, 31.94, 29.79, 29.78, 29.75, 29.72, 29.64, 29.50, 29.36, 29.28, 29.23, 29.11, 28.98, 28.95, 25.86, 22.83, 22.78, 14.25, 14.22. **HRMS** (ESI)**:** Calcd. for $C_{21}H_{42}OSNa$ $[M+Na]^+$: 365.2846, found: 365.2849.

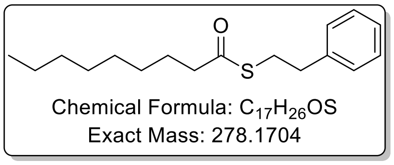

5-(Nonanoylthio)pentyl nonanoate (47): colorless oil liquid, 450 mg, 81% yield, 93% linear selectivity. **^{1}H NMR** (400 MHz, $CDCl_3$) δ 7.33 – 7.24 (m, 2H), 7.25 – 7.16 (m, 3H), 3.11 (t, 2H), 2.85 (t, *J* = 7.7 Hz, 2H), 2.53 (t, *J* = 7.5 Hz, 2H), 1.74 – 1.51 (m, 2H), 1.36 – 1.17 (m, 10H), 0.88 (t, *J* = 6.6 Hz, 3H). **^{13}C NMR** (101 MHz, $CDCl_3$) δ 199.56, 140.15, 128.70, 128.57, 126.58, 44.29, 36.06, 31.91, 30.29, 29.33, 29.20, 29.05, 25.78, 22.75, 14.21. **HRMS** (ESI)**:** Calcd. for $C_{17}H_{26}OSNa$ $[M+Na]^+$: 301.1591, found: 301.1597.

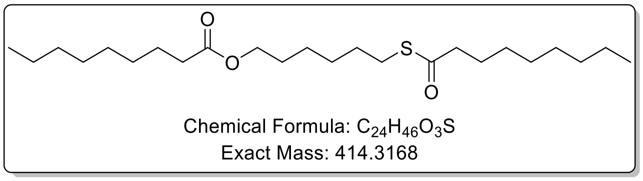

5-(Nonanoylthio)pentyl nonanoate (48): colorless oil liquid, 349 mg, 84% yield, 86% linear selectivity. **^{1}H NMR** (400 MHz, $CDCl_3$) δ 4.05 (t, *J* = 5.9 Hz, 2H), 2.86 (t, *J* = 6.4 Hz, 2H), 2.53 (t, *J* = 6.7 Hz, 2H), 2.28 (t, *J* = 6.7 Hz, 2H), 1.83 – 1.48 (m, 8H), 1.38 (s, 4H), 1.28 (d, *J* = 6.2 Hz, 18H), 0.87 (dd, *J* = 6.3, 5.3 Hz, 6H). **^{13}C NMR** (101 MHz, $CDCl_3$) δ 199.64, 173.93, 64.18, 44.19, 34.42, 31.87, 31.86, 29.59, 29.29, 29.28, 29.23, 29.20, 29.15, 29.02, 28.67, 28.58, 28.46, 25.75, 25.56, 25.07, 22.70, 14.14. **HRMS** (ESI)**:** Calcd. for $C_{24}H_{46}O_3SNa$ $[M+Na]^+$: 437.3071, found: 437.3060.

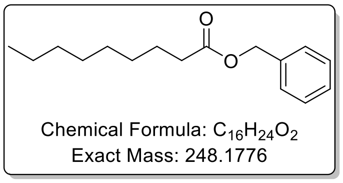

Benzyl nonanoate (49): colorless oil liquid, 490 mg, 99% yield, 91% linear selectivity. **^{1}H NMR** (400 MHz, $CDCl_3$) δ 7.42 – 7.17 (m, 5H), 5.10 (s, 2H), 2.34 (t, *J* = 7.5 Hz, 2H), 1.64 (dt, *J* = 14.7, 7.4 Hz, 2H), 1.36 – 1.17 (m, 10H), 0.87 (t, *J* = 6.9 Hz, 3H). **^{13}C NMR** (101 MHz, $CDCl_3$) δ 173.69, 136.26, 128.59, 128.23, 128.20, 66.10, 34.39, 31.88, 29.29, 29.21, 25.04, 22.72, 14.16.[24]

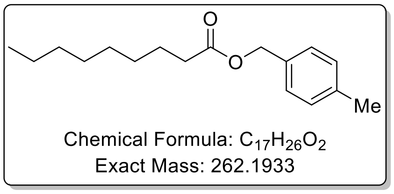

4-Methylbenzyl nonanoate (50): colorless oil liquid, 500 mg, 95% yield, 91% linear selectivity. **^{1}H NMR** (400 MHz, $CDCl_3$) δ 7.24 (d, *J* = 8.0 Hz, 2H), 7.15 (d, *J* = 7.8 Hz, 2H), 5.06 (s, 2H), 2.36 – 2.28 (m, 5H), 1.72 – 1.52 (m, 2H), 1.36 – 1.19 (m, 10H), 0.87 (t, *J* = 6.9 Hz, 3H). **^{13}C NMR** (101 MHz, $CDCl_3$) δ 173.74, 138.01, 133.27, 129.27, 128.41, 66.05, 34.43, 31.88, 29.30, 29.21, 25.05, 22.73, 21.23, 14.15. **HRMS** (ESI)**:** Calcd. for $C_{17}H_{26}O_2Na$ $[M+Na]^+$: 285.1824, found: 285.1825.

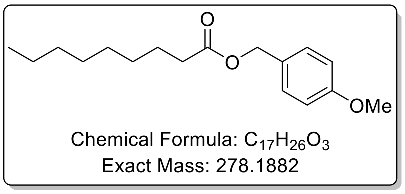

4-Methoxybenzyl nonanoate (51): yellowish oil liquid, 468 mg, 84% yield, 91% linear selectivity. **^{1}H NMR** (400 MHz, $CDCl_3$) δ 7.29 (d, *J* = 8.7 Hz, 2H), 6.88 (d, *J* = 8.7 Hz, 2H), 5.04 (s, 2H), 3.80 (s, 3H), 2.32 (t, *J* = 7.6 Hz, 2H), 1.74 – 1.54 (m, 2H), 1.33 – 1.16 (m, 10H), 0.87 (t, *J* = 6.9 Hz, 3H). **^{13}C NMR** (101 MHz, $CDCl_3$) δ 173.89, 159.70, 130.14, 128.42, 114.03, 65.98, 55.37, 34.50, 31.91, 29.32, 29.24, 25.08, 22.75, 14.20.[25]

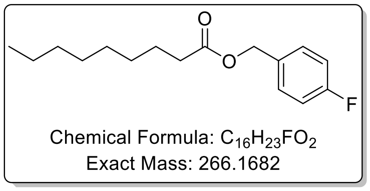

4-Fluorobenzyl nonanoate (52): yellowish oil liquid, 488 mg, 92% yield, 90% linear selectivity. **^{1}H NMR** (400 MHz, $CDCl_3$) δ 7.38 – 7.29 (m, 2H), 7.09 – 6.96 (m, 2H), 5.07 (s, 2H), 2.33 (t, *J* = 7.5 Hz, 2H), 1.71 – 1.56 (m, 2H), 1.26 (d, *J* = 7.9 Hz, 10H), 0.87 (t, *J* = 6.9 Hz, 3H). **^{13}C NMR** (101 MHz, $CDCl_3$) δ 173.69, 162.71 (d, *J* = 246.8 Hz), 132.14 (d, *J* = 3.2 Hz), 130.26 (d, *J* = 8.3 Hz), 115.53 (d, *J* = 21.5 Hz), 65.40, 34.39, 31.89, 29.30, 29.21, 25.04, 22.74, 14.16. **^{19}F NMR** (376 MHz, $CDCl_3$) δ -113.79. **HRMS** (ESI)**:** Calcd. for $C_{16}H_{23}FO_2Na$ $[M+Na]^+$: 289.1571, found: 289.1574.

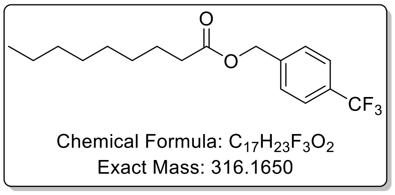

4-(Trifluoromethyl)benzyl nonanoate (53): colorless oil liquid, 572 mg, 91% yield, 90% linear selectivity. **^{1}H NMR** (400 MHz, $CDCl_3$) δ 7.62 (d, *J* = 8.1 Hz, 2H), 7.46 (d, *J* = 8.0 Hz, 2H), 5.16 (s, 2H), 2.38 (t, *J* = 7.5 Hz, 2H), 1.87 – 1.50 (m, 2H), 1.40 – 1.19 (m, 10H), 0.87 (t, *J* = 6.9 Hz, 3H). **^{13}C NMR** (101 MHz, $CDCl_3$) δ 173.59, 140.32, 130.44 (q, *J* = 32.5 Hz), 128.21, 125.61 (q, *J* = 3.7 Hz), 122.81, 65.17, 34.34, 31.91, 29.32, 29.24, 25.05, 22.75, 14.16. **^{19}F NMR** (376 MHz, $CDCl_3$) δ -62.68. **HRMS** (ESI)**:** Calcd. for $C_{17}H_{23}F_3O_2Na$ $[M+Na]^+$: 339.1543, found: 339.1542.

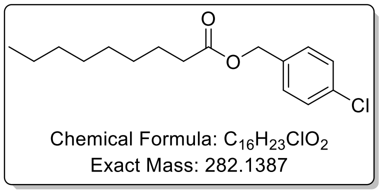

4-Chlorobenzyl nonanoate (54): colorless oil liquid, 462 mg, 82% yield, 91% linear selectivity. **^{1}H NMR** (400 MHz, $CDCl_3$) δ 7.55 – 7.08 (m, 4H), 5.07 (s, 2H), 2.34 (t, *J* = 7.5 Hz, 2H), 1.72 – 1.53 (m, 2H), 1.26 (d, *J* = 8.1 Hz, 10H), 0.87 (t, *J* = 6.9 Hz, 3H). **^{13}C NMR** (101 MHz, $CDCl_3$) δ 173.70, 134.80, 134.17, 129.69, 128.85, 65.33, 34.41, 31.91, 29.32, 29.24, 25.06, 22.76, 14.21. **HRMS** (ESI)**:** Calcd. for $C_{16}H_{23}ClO_2Na$ $[M+Na]^+$: 305.1278, found: 305.1279.

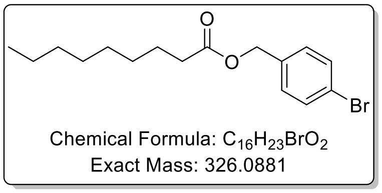

4-Bromobenzyl nonanoate (55): colorless oil liquid, 360 mg, 55% yield, 92% linear selectivity. **^{1}H NMR** (400 MHz, $CDCl_3$) δ 7.51 – 7.43 (m, 2H), 7.22 (d, *J* = 8.4 Hz, 2H), 5.05 (s, 2H), 2.34 (t, *J* = 7.5 Hz, 2H),

1.72 – 1.54 (m, 2H), 1.36 – 1.18 (m, 10H), 0.87 (t, *J* = 7.0 Hz, 3H). ^{13}**C NMR** (101 MHz, $CDCl_3$) δ 173.67, 135.31, 131.81, 129.98, 122.31, 65.35, 34.39, 31.91, 29.32, 29.24, 25.05, 22.76, 14.21. **HRMS** (ESI)**:** Calcd. for $C_{16}H_{23}BrO_2Na$ $[M+Na]^+$: 349.0768, found: 349.0774.

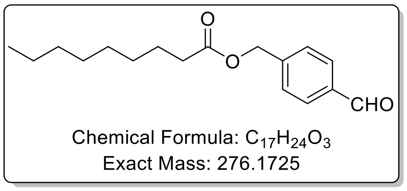

4-Formylbenzyl nonanoate (56): colorless oil liquid, 550 mg, 99% yield, 90% linear selectivity. ^{1}**H NMR** (400 MHz, $CDCl_3$) δ 10.02 (s, 1H), 7.88 (d, *J* = 8.1 Hz, 2H), 7.51 (d, *J* = 8.0 Hz, 2H), 5.19 (s, 2H), 2.39 (t, *J* = 7.5 Hz, 2H), 1.72 – 1.60 (m, 2H), 1.28 (d, *J* = 12.6 Hz, 10H), 0.87 (t, *J* = 6.5 Hz, 3H). ^{13}**C NMR** (101 MHz, $CDCl_3$) δ 191.91, 173.60, 143.06, 136.14, 130.06, 128.26, 65.25, 34.32, 31.87, 29.28, 29.21, 29.19, 25.02, 22.72, 14.17. **HRMS** (ESI)**:** Calcd. for $C_{17}H_{24}O_3Na$ $[M+Na]^+$: 299.1616, found: 299.1618.

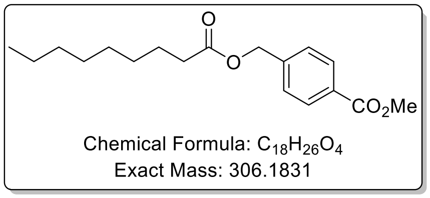

Methyl 4-((nonanoyloxy)methyl)benzoate (57): colorless oil liquid, 609 mg, 99% yield, 90% linear selectivity. ^{1}**H NMR** (400 MHz, $CDCl_3$) δ 8.03 (d, *J* = 8.0 Hz, 2H), 7.41 (d, *J* = 8.0 Hz, 2H), 5.16 (s, 2H), 3.91 (s, 3H), 2.38 (t, *J* = 7.5 Hz, 2H), 1.64 (dd, *J* = 14.2, 7.1 Hz, 2H), 1.27 (dd, *J* = 6.9, 4.8 Hz, 10H), 0.87 (t, *J* = 6.7 Hz, 3H). ^{13}**C NMR** (101 MHz, $CDCl_3$) δ 173.51, 166.74, 141.29, 129.86, 127.67, 127.63, 65.28, 52.14, 34.27, 31.83, 29.24, 29.15, 24.97, 22.67, 14.10. **HRMS** (ESI)**:** Calcd. for $C_{18}H_{26}O_4Na$ $[M+Na]^+$: 329.1723, found: 329.1723.

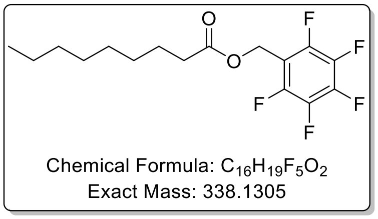

(Perfluorophenyl)methyl nonanoate (58): colorless oil liquid, 508 mg, 75% yield, 86% linear selectivity. ^{1}**H NMR** (400 MHz, $CDCl_3$) δ 5.20 (s, 2H), 2.33 (t, *J* = 7.5 Hz, 2H), 1.78 – 1.45 (m, 2H), 1.44 – 1.09 (m, 10H), 0.87 (t, *J* = 6.9 Hz, 3H). ^{13}**C NMR** (101 MHz, $CDCl_3$) δ 173.21, 147.10, 144.60, 143.13, 140.59, 138.91, 136.39, 109.80, 53.23, 34.04, 31.91, 29.29, 29.23, 29.18, 24.95, 22.76, 14.14. ^{19}**F NMR** (376 MHz, $CDCl_3$) δ -142.05 (dd, *J* = 21.7, 8.1 Hz), -152.86 (t, *J* = 21.2 Hz), -161.76 (td, *J* = 21.1, 7.8 Hz). **HRMS** (ESI)**:** Calcd. for $C_{16}H_{19}F_5O_2Na$ $[M+Na]^+$: 361.1200, found: 321.1197.

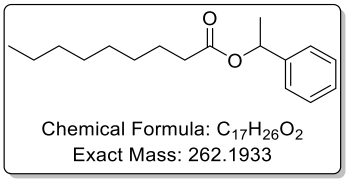

1-Phenylethyl nonanoate (59): colorless oil liquid, 480 mg, 92% yield, 87% linear selectivity. ^{1}**H NMR** (400 MHz, $CDCl_3$) δ 7.36 – 7.31 (m, 4H), 7.30 – 7.22 (m, 1H), 5.89 (q, *J* = 6.6 Hz, 1H), 2.38 – 2.19 (m, 2H), 1.61 (dd, *J* = 14.4, 7.2 Hz, 2H), 1.52 (d, *J* = 6.6 Hz, 3H), 1.37 – 1.20 (m, 10H), 0.87 (t, *J* = 6.9 Hz, 3H). ^{13}**C NMR** (101 MHz, $CDCl_3$) δ 173.13, 141.97, 128.54, 127.85, 126.14, 72.07, 34.71, 31.89, 29.31, 29.22, 29.20, 25.07, 22.73, 22.34, 14.17. **HRMS** (ESI)**:** Calcd. for $C_{17}H_{26}O_2Na$ $[M+Na]^+$: 285.1827, found: 285.1825.

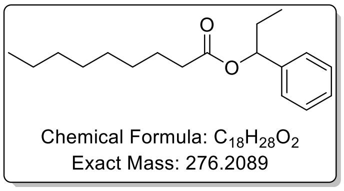

1-Phenylpropyl nonanoate (60): yellowish oil liquid, 500 mg, 91% yield, 87% linear selectivity. **^{1}H NMR** (400 MHz, $CDCl_3$) δ 7.37 – 7.28 (m, 4H), 7.29 – 7.20 (m, 1H), 5.67 (t, *J* = 6.9 Hz, 1H), 2.40 – 2.23 (m, 2H), 1.99 – 1.72 (m, 2H), 1.68 – 1.55 (m, 2H), 1.34 – 1.20 (m, 10H), 0.93 – 0.82 (m, 6H). **^{13}C NMR** (101 MHz, $CDCl_3$) δ 173.26, 140.86, 128.43, 127.82, 126.62, 77.14, 34.71, 31.91, 29.49, 29.33, 29.23, 25.13, 22.75, 14.18, 10.02. **HRMS** (ESI)**:** Calcd. for $C_{18}H_{28}O_2Na$ $[M+Na]^+$: 299.1977, found: 299.1982.

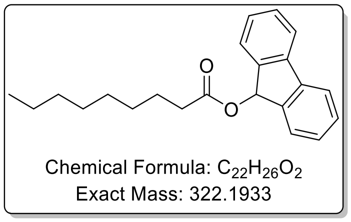

9*H*-Fluoren-9-yl nonanoate (61): yellow oil liquid, 524 mg, 81% yield, 84% linear selectivity. **^{1}H NMR** (400 MHz, $CDCl_3$) δ 7.62 (d, *J* = 7.5 Hz, 2H), 7.52 (d, *J* = 7.5 Hz, 2H), 7.37 (t, *J* = 7.3 Hz, 2H), 7.26 (td, *J* = 7.5, 0.9 Hz, 2H), 6.81 (d, *J* = 3.9 Hz, 1H), 2.41 (t, *J* = 7.5 Hz, 2H), 1.68 (dt, *J* = 15.1, 7.4 Hz, 2H), 1.42 – 1.13 (m, 10H), 0.87 (t, *J* = 6.8 Hz, 3H). **^{13}C NMR** (101 MHz, $CDCl_3$) δ 174.64, 142.29, 141.09, 129.47, 127.88, 125.89, 120.09, 74.99, 34.62, 31.87, 29.28, 29.21, 25.22, 22.73, 14.18. **HRMS** (ESI)**:** Calcd. for $C_{22}H_{26}O_2Na$ $[M+Na]^+$: 345.1824, found: 345.1825.

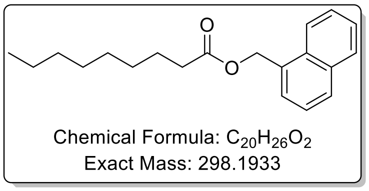

Naphthalen-1-ylmethyl nonanoate (62): yellowish oil liquid, 530 mg, 89% yield, 91% linear selectivity. **^{1}H NMR** (400 MHz, $CDCl_3$) δ 8.00 (d, *J* = 8.2 Hz, 1H), 7.90 – 7.78 (m, 2H), 7.59 – 7.47 (m, 3H), 7.47 – 7.34 (m, 1H), 5.56 (s, 2H), 2.34 (t, *J* = 7.5 Hz, 2H), 1.71 – 1.54 (m, 2H), 1.35 – 1.13 (m, 10H), 0.86 (t, *J* = 6.9 Hz, 3H). **^{13}C NMR** (101 MHz, $CDCl_3$) δ 173.89, 133.85, 131.75, 129.33, 128.82, 127.54, 126.61, 126.03, 125.38, 123.70, 64.50, 34.48, 31.89, 29.31, 29.24, 29.22, 25.11, 22.75, 14.20. **HRMS** (ESI)**:** Calcd. for $C_{20}H_{26}O_2Na$ $[M+Na]^+$: 321.1815, found: 321.1825.

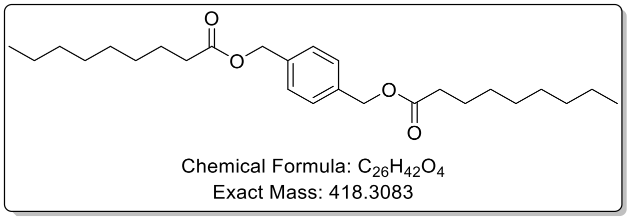

1,4-Phenylenebis(methylene) dinonanoate (63): colorless oil liquid, 382 mg, 91% yield, 82% linear selectivity. **^{1}H NMR** (400 MHz, $CDCl_3$) δ 7.35 (s, 4H), 5.11 (s, 4H), 2.35 (t, *J* = 7.6 Hz, 4H), 1.79 – 1.47 (m, 4H), 1.27 (d, *J* = 8.9 Hz, 20H), 0.87 (t, *J* = 6.9 Hz, 6H). **^{13}C NMR** (101 MHz, $CDCl_3$) δ 173.74, 136.27, 128.45, 65.78, 34.43, 31.91, 29.32, 29.25, 29.22, 25.06, 22.75, 14.19. **HRMS** (ESI)**:** Calcd. for $C_{26}H_{42}O_4Na$ $[M+Na]^+$: 441.2972, found: 441.2975.

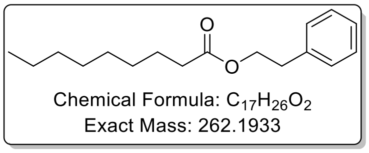

Phenethyl nonanoate (64): colorless oil liquid, 620 mg, 99% yield, 91% linear selectivity. **^{1}H NMR** (400 MHz, $CDCl_3$) δ 7.33 – 7.24 (m, 2H), 7.21 (dd, *J* = 6.8, 4.6 Hz, 3H), 4.28 (t, *J* = 7.1 Hz, 2H), 2.92 (t, *J* = 7.1

Hz, 2H), 2.27 (t, *J* = 7.5 Hz, 2H), 1.64 – 1.51 (m, 2H), 1.37 – 1.18 (m, 10H), 0.88 (t, *J* = 6.8 Hz, 3H). ^{13}**C NMR** (101 MHz, $CDCl_3$) δ 173.78, 137.96, 128.95, 128.51, 126.57, 64.73, 35.23, 34.38, 31.89, 29.30, 29.19, 25.01, 22.72, 14.15. **HRMS** (ESI)**:** Calcd. for $C_{17}H_{26}O_2Na$ $[M+Na]^+$: 285.1819, found: 285.1825.

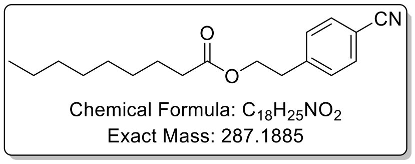

4-Cyanophenethyl nonanoate (65): yellow oil liquid, 300 mg, 52% yield, 90% linear selectivity. ^{1}**H NMR** (400 MHz, $CDCl_3$) δ 7.60 (d, *J* = 8.2 Hz, 2H), 7.34 (d, *J* = 8.2 Hz, 2H), 4.31 (t, *J* = 6.7 Hz, 2H), 3.00 (t, *J* = 6.7 Hz, 2H), 2.27 (t, *J* = 7.5 Hz, 2H), 1.67 – 1.50 (m, 2H), 1.22 (d, *J* = 28.6 Hz, 10H), 0.88 (t, *J* = 6.7 Hz, 3H). ^{13}**C NMR** (101 MHz, $CDCl_3$) δ 173.68, 143.67, 132.27, 129.71, 118.82, 110.59, 63.67, 35.22, 34.22, 31.79, 29.19, 29.10, 29.08, 24.89, 22.63, 14.08. **HRMS** (ESI)**:** Calcd. for $C_{18}H_{25}NO_2Na$ $[M+Na]^+$: 310.1772, found: 310.1777.

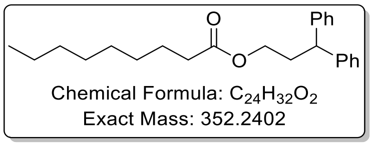

3,3-Diphenylpropyl nonanoate (66): yellow oil liquid, 692 mg, 98% yield, 89% linear selectivity. ^{1}**H NMR** (400 MHz, $CDCl_3$) δ 7.34 – 7.19 (m, 8H), 7.20 – 7.10 (m, 2H), 4.30 – 3.75 (m, 3H), 2.50 – 2.32 (m, 2H), 2.25 (t, *J* = 7.5 Hz, 2H), 1.70 – 1.47 (m, 2H), 1.40 – 1.20 (m, 10H), 0.88 (t, *J* = 6.8 Hz, 3H). ^{13}**C NMR** (101 MHz, $CDCl_3$) δ 173.85, 144.13, 128.66, 127.88, 126.49, 62.74, 47.77, 34.41, 31.92, 29.35, 29.27, 29.25, 25.10, 22.75, 14.20. **HRMS** (ESI)**:** Calcd. for $C_{24}H_{32}O_2Na$ $[M+Na]^+$: 375.2298, found: 35.2295.

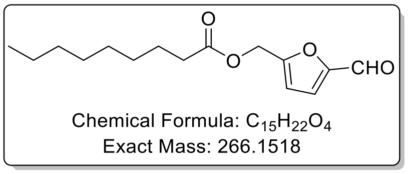

(5-Formylfuran-2-yl)methyl nonanoate (67): colorless oil liquid, 400 mg, 75% yield, 89% linear selectivity. ^{1}**H NMR** (400 MHz, $CDCl_3$) δ 9.64 (s, 1H), 7.23 (d, *J* = 3.5 Hz, 1H), 6.60 (d, *J* = 3.4 Hz, 1H), 5.14 (s, 2H), 2.36 (t, *J* = 7.5 Hz, 2H), 1.87 – 1.53 (m, 2H), 1.55 – 1.10 (m, 10H), 0.87 (t, *J* = 6.4 Hz, 3H). ^{13}**C NMR** (101 MHz, $CDCl_3$) δ 177.86, 173.21, 155.75, 152.86, 121.76, 112.51, 57.67, 34.00, 31.81, 29.20, 29.12, 29.09, 24.85, 22.66, 14.10. **HRMS** (ESI)**:** Calcd. for $C_{15}H_{22}O_4Na$ $[M+Na]^+$: 289.1411, found: 289.1410.

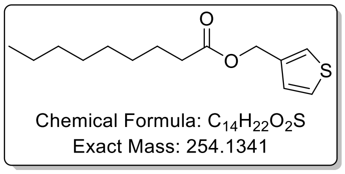

Thiophen-3-ylmethyl nonanoate (68): colorless oil liquid, 400 mg, 75% yield, 90% linear selectivity. ^{1}**H NMR** (400 MHz, $CDCl_3$) δ 7.42 – 7.12 (m, 2H), 7.16 – 6.92 (m, 1H), 5.11 (s, 2H), 2.33 (t, *J* = 7.5 Hz, 2H), 1.74 – 1.51 (m, 2H), 1.26 (d, *J* = 6.8 Hz, 10H), 0.87 (t, *J* = 6.8 Hz, 3H).[26]

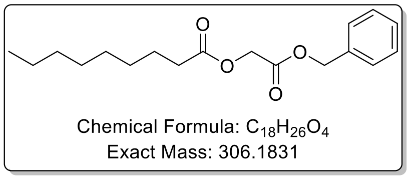

2-(Benzyloxy)-2-oxoethyl nonanoate (69): yellowish oil liquid, 530 mg, 82% yield, 88% linear selectivity. ^{1}**H NMR** (400 MHz, $CDCl_3$) δ 7.42 – 7.30 (m, 5H), 5.19 (s, 2H), 4.65 (s, 2H), 2.41 (t, *J* = 7.6 Hz, 2H), 1.77 – 1.54 (m, 2H), 1.39 – 1.15 (m, 10H), 0.88 (t, *J* = 6.8 Hz, 3H). ^{13}**C NMR** (101 MHz, $CDCl_3$) δ 173.31, 167.98, 135.25, 128.78, 128.67, 128.52, 67.20, 60.70, 33.96, 31.95, 29.33, 29.23, 29.19, 24.95, 22.78, 14.23. **HRMS** (ESI)**:** Calcd. for $C_{18}H_{26}O_4Na$ $[M+Na]^+$: 329.1727, found: 329.1723.

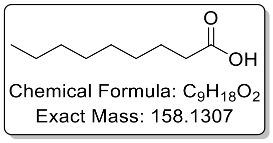

Nonanoic acid (70): colorless oil liquid, 300 mg, 95% yield, 87% linear selectivity. **^{1}H NMR** (400 MHz, $CDCl_3$) δ 11.20 (s, 1H), 2.35 (t, *J* = 7.5 Hz, 2H), 1.70 – 1.57 (m, 2H), 1.40 – 1.21 (m, 10H), 0.88 (t, *J* = 6.8 Hz, 3H). **^{13}C NMR** (101 MHz, $CDCl_3$) δ 180.66, 34.28, 31.95, 29.35, 29.24, 29.21, 24.82, 22.78, 14.18.[27]

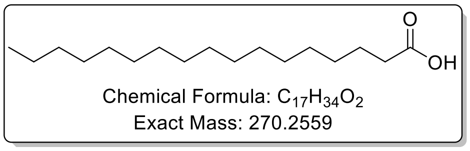

Heptadecanoic acid (71): white solid, 535 mg, 99% yield, 84% linear selectivity. **^{1}H NMR** (400 MHz, $CDCl_3$) δ 11.33 – 10.74 (m, 1H), 2.35 (t, *J* = 7.5 Hz, 2H), 1.79 – 1.49 (m, 2H), 1.26 (s, 26H), 0.88 (t, *J* = 6.8 Hz, 3H). **^{13}C NMR** (101 MHz, $CDCl_3$) δ 180.42, 34.24, 32.09, 29.85, 29.82, 29.80, 29.75, 29.59, 29.52, 29.40, 29.22, 24.83, 22.85, 14.26.[27]

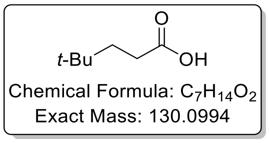

4,4-Dimethylpentanoic acid (72): colorless oil liquid, 239 mg, 92% yield, >99% linear selectivity. **^{1}H NMR** (400 MHz, $CDCl_3$) δ 10.67 (s, 1H), 2.77 – 2.19 (m, 2H), 1.73 – 1.33 (m, 2H), 0.91 (s, 9H). **^{13}C NMR** (101 MHz, $CDCl_3$) δ 181.23, 38.47, 30.19, 30.08, 29.11.[28]

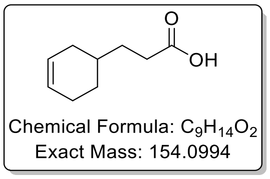

3-(Cyclohex-3-en-1-yl)propanoic acid (73): colorless oil liquid, 186 mg, 60% yield, 90% linear selectivity. **^{1}H NMR** (400 MHz, $CDCl_3$) δ 11.56 (s, 1H), 6.00 – 5.29 (m, 2H), 2.67 – 2.29 (m, 2H), 2.35 – 1.91 (m, 3H), 1.88 – 1.45 (m, 5H), 1.36 – 1.02 (m, 1H). **^{13}C NMR** (101 MHz, $CDCl_3$) δ 180.91, 127.15, 126.19, 33.08, 31.84, 31.53, 31.34, 28.61, 25.15.[29]

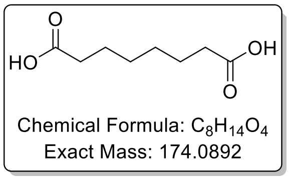

Octanedioic acid (74): white solid, 125 mg, 72% yield, 88% linear selectivity. **^{1}H NMR** (400 MHz, DMSO) δ 12.03 (s, 2H), 2.19 (t, *J* = 7.3 Hz, 4H), 1.58 – 1.39 (m, 4H), 1.26 (s, 4H). **^{13}C NMR** (101 MHz, DMSO) δ 174.54, 39.52, 33.64, 28.34, 24.43.[29]

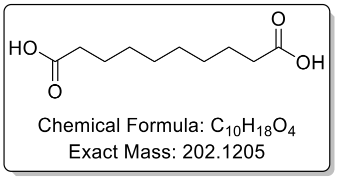

Decanedioic acid (75): white solid, 156 mg, 77% yield, 80% linear selectivity. **^{1}H NMR** (400 MHz, DMSO) δ 11.99 (s, 2H), 2.18 (t, *J* = 7.4 Hz, 4H), 1.49 (dd, *J* = 9.5, 4.4 Hz, 4H), 1.25 (s, 8H). **^{13}C NMR** (101 MHz, DMSO) δ 174.57, 33.75, 28.74, 28.63, 24.59.[29]

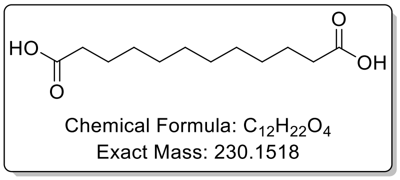

Dodecanedioic acid (76): white solid, 184 mg, 80% yield, 80% linear selectivity. **^{1}H NMR** (400 MHz, DMSO) δ 12.02 (s, 2H), 2.45 – 2.05 (m, 4H), 1.79 – 1.46 (m, 4H), 1.29 (s, 12H). **^{13}C NMR** (101 MHz, DMSO) δ 174.57, 33.76, 29.02, 28.89, 28.70, 24.62.[30]

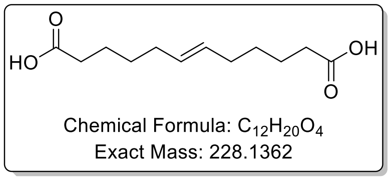

(E)-dodec-6-enedioic acid (77): colorless oil liquid, 155 mg, 68% yield, 82% linear selectivity. **^{1}H NMR** (400 MHz, DMSO) δ 12.00 (s, 2H), 5.37 (d, *J* = 3.6 Hz, 2H), 2.26 – 2.13 (m, 4H), 2.06 – 1.85 (m, 4H), 1.59 – 1.42 (m, 4H), 1.38 – 1.15 (m, 4H). **^{13}C NMR** (101 MHz, DMSO) δ 174.59, 130.06, 33.66, 31.88, 28.67, 24.19. **HRMS** (ESI)**:** Calcd. for $C_{12}H_{20}O_4Na$ $[M+Na]^+$: 251.1254, found: 251.1255.

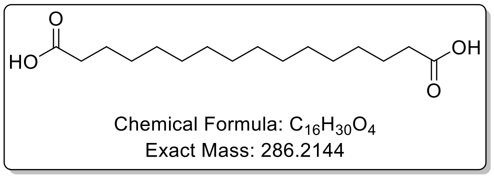

Hexadecanedioic acid (78): white solid, 200 mg, 70% yield, 75% linear selectivity. **^{1}H NMR** (400 MHz, DMSO) δ 11.93 (s, 2H), 2.35 – 2.01 (m, 4H), 1.64 – 1.37 (m, 4H), 1.23 (s, 20H). **^{13}C NMR** (101 MHz, DMSO) δ 174.51, 33.78, 29.28, 29.25, 29.16, 29.00, 28.78, 24.66.[31]

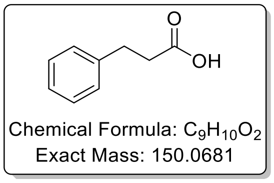

3-Phenylpropanoic acid (79): colorless oil liquid, 294 mg, 98% yield, 89% linear selectivity. **^{1}H NMR** (400 MHz, $CDCl_3$) δ 10.90 (s, 1H), 7.38 – 7.23 (m, 2H), 7.19 (dd, *J* = 7.2, 5.3 Hz, 3H), 2.94 (t, *J* = 7.8 Hz, 2H), 2.66 (t, *J* = 7.8 Hz, 2H). **^{13}C NMR** (101 MHz, $CDCl_3$) δ 179.59, 140.24, 128.65, 128.35, 126.46, 35.73, 30.64.[27]

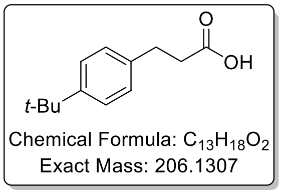

3-(4-(*tert*-Butyl)phenyl)propanoic acid (80): white solid, 330 mg, 80% yield, 83% linear selectivity. **^{1}H NMR** (400 MHz, $CDCl_3$) δ 11.10 (s, 1H), 7.48 – 7.23 (m, 2H), 7.14 (d, *J* = 8.3 Hz, 2H), 2.93 (t, *J* = 7.8 Hz, 2H), 2.68 (t, *J* = 7.8 Hz, 2H), 1.30 (s, 9H). **^{13}C NMR** (101 MHz, $CDCl_3$) δ 179.66, 149.33, 137.22, 128.05, 125.59, 35.75, 34.52, 31.51, 30.15.[32]

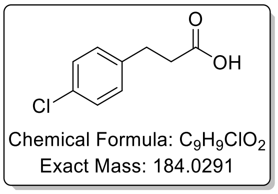

3-(4-Chlorophenyl)propanoic acid (81): white solid, 276 mg, 75% yield, 87% linear selectivity. **^{1}H NMR** (400 MHz, $CDCl_3$) δ 11.04 (s, 1H), 7.32 – 7.20 (m, 2H), 7.13 (d, *J* = 8.5 Hz, 2H), 2.91 (t, *J* = 7.6 Hz, 2H),

2.66 (dd, J = 9.0, 6.3 Hz, 2H). **^{13}C NMR** (101 MHz, $CDCl_3$) δ 179.08, 138.66, 132.32, 129.79, 128.80, 35.54, 29.99.[33]

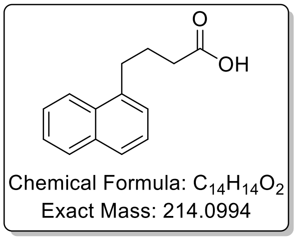

4-(Naphthalen-1-yl)butanoic acid (82): white solid, 280 mg, 66% yield, 91% linear selectivity. **^{1}H NMR** (400 MHz, $CDCl_3$) δ 11.30 (s, 1H), 8.05 (d, J = 8.3 Hz, 1H), 7.85 (d, J = 7.6 Hz, 1H), 7.72 (d, J = 8.2 Hz, 1H), 7.57 – 7.43 (m, 2H), 7.43 – 7.35 (m, 1H), 7.31 (d, J = 6.8 Hz, 1H), 3.45 – 2.91 (m, 2H), 2.46 (t, J = 7.3 Hz, 2H), 2.10 (p, J = 7.4 Hz, 2H). **^{13}C NMR** (101 MHz, $CDCl_3$) δ 149.47, 129.25, 115.87, 112.16, 52.95, 38.39, 32.02, 29.77, 29.71, 29.43, 27.34, 26.76, 22.82, 14.26.[34]

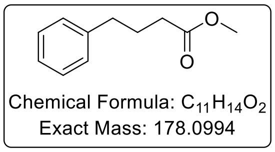

Methyl 4-phenylbutanoate (89): colorless oil liquid, 160 mg, 90% yield, 75% linear selectivity. **^{1}H NMR** (400 MHz, $CDCl_3$) δ 7.28 – 7.21 (m, 2H), 7.20 – 7.11 (m, 3H), 3.62 (s, 3H), 2.62 (t, 2H), 2.29 (t, J = 7.5 Hz, 2H), 2.05 – 1.82 (m, 2H). **^{13}C NMR** (101 MHz, $CDCl_3$) δ 173.74, 141.30, 128.41, 128.32, 125.92, 51.34, 35.05, 33.25, 26.44. **HRMS** (ESI)**:** Calcd. for $C_{11}H_{14}O_2Na$ $[M+Na]^+$: 201.0895, found: 201.0886.

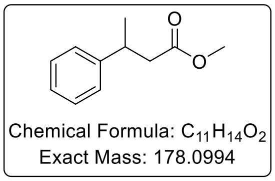

Methyl 3-phenylbutanoate (93): colorless oil liquid, 152 mg, 85% yield, >99% linear selectivity. **^{1}H NMR** (400 MHz, $CDCl_3$) δ 7.25 – 6.91 (m, 5H), 3.45 (s, 3H), 3.28 – 3.05 (m, 1H), 2.55 – 2.31 (m, 2H), 1.16 (d, J = 7.0 Hz, 3H). **^{13}C NMR** (101 MHz, $CDCl_3$) δ 172.54, 145.60, 128.40, 126.59, 126.30, 51.22, 42.56, 36.32, 21.65. **HRMS** (ESI)**:** Calcd. for $C_{11}H_{14}O_2Na$ $[M+Na]^+$:201.0895, found: 201.0886.

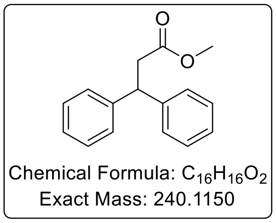

Methyl 3,3-diphenylpropanoate (94): yellow oil liquid, 168 mg, 70% yield, >99% linear selectivity. **^{1}H NMR** (400 MHz, $CDCl_3$) δ 7.19 – 7.09 (m, 8H), 7.08 – 7.01 (m, 2H), 4.46 (t, J = 8.0 Hz, 1H), 3.42 (s, 3H), 2.94 (d, J = 8.0 Hz, 2H). **^{13}C NMR** (101 MHz, $CDCl_3$) δ 172.22, 143.51, 128.59, 127.67, 126.57, 51.63, 46.99, 40.57. **HRMS** (ESI)**:** Calcd. for $C_{16}H_{16}O_2Na$ $[M+Na]^+$: 263.1042, found: 263.1043.

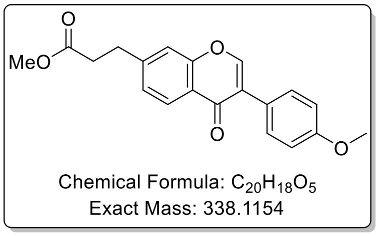

Methyl 3-(3-(4-methoxyphenyl)-4-oxo-4H-chromen-7-yl)propanoate (96): white solid, 284 mg, 84% yield, 91% linear selectivity. **^{1}H NMR** (400 MHz, $CDCl_3$) δ 8.22 (d, J = 8.2 Hz, 1H), 7.96 (s, 1H), 7.50 (d, J = 8.7 Hz, 2H), 7.39 – 7.16 (m, 2H), 6.97 (d, J = 8.7 Hz, 2H), 3.84 (s, 3H), 3.69 (s, 3H), 3.09 (t, J = 7.5 Hz,

2H), 2.72 (t, *J* = 7.6 Hz, 2H). **^{13}C NMR** (101 MHz, $CDCl_3$) δ 176.33, 172.81, 159.69, 156.43, 152.47, 147.25, 130.19, 126.65, 125.79, 125.05, 124.23, 123.01, 117.37, 114.07, 55.43, 51.91, 34.97, 30.92. **HRMS** (E SI)**:** Calcd. for $C_{20}H_{18}O_5Na$ $[M+Na]^+$: 265.0505, found: 265.0503.

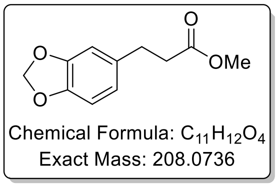

Methyl 3-(benzo[d][1,3]dioxol-5-yl)propanoate (97): colorless oil liquid, 170 mg, 82% yield, 82% linear selectivity. **^{1}H NMR** (400 MHz, $CDCl_3$) δ 6.82 – 6.41 (m, 3H), 5.81 (s, 2H), 3.57 (s, 3H), 2.76 (t, *J* = 7.8 Hz, 2H), 2.48 (dd, *J* = 9.1, 6.4 Hz, 2H). **^{13}C NMR** (101 MHz, $CDCl_3$) δ 173.22, 147.67, 145.97, 134.32, 121.07, 108.76, 108.23, 100.85, 51.57, 35.98, 30.69. **HRMS** (ESI)**:** Calcd. for $C_{11}H_{12}O_4Na$ $[M+Na]^+$: 231.0628, found: 231.0631.

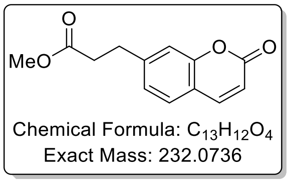

Methyl 3-(2-oxo-2H-chromen-7-yl)propanoate (98): white solid, 220 mg, 95% yield, 88% linear selectivity. **^{1}H NMR** (400 MHz, $CDCl_3$) δ 7.69 (d, *J* = 9.5 Hz, 1H), 7.42 (d, *J* = 7.9 Hz, 1H), 7.24 – 6.97 (m, 2H), 6.38 (d, *J* = 9.6 Hz, 1H), 3.69 (d, *J* = 2.1 Hz, 3H), 3.05 (t, *J* = 7.6 Hz, 2H), 2.69 (t, *J* = 7.6 Hz, 2H). **^{13}C NMR** (101 MHz, $CDCl_3$) δ 172.78, 160.94, 154.29, 145.55, 143.32, 127.97, 124.87, 117.25, 116.44, 116.00, 51.86, 35.03, 30.87. **HRMS** (ESI)**:** Calcd. for $C_{13}H_{12}O_4Na$ $[M+Na]^+$: 255.0628, found: 255.0615.

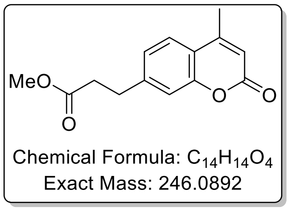

Methyl 3-(4-methyl-2-oxo-2H-chromen-7-yl)propanoate (99): white solid, 226 mg, 92% yield, 86% linear selectivity. **^{1}H NMR** (400 MHz, $CDCl_3$) δ 7.53 (d, *J* = 8.5 Hz, 1H), 7.16 (dd, *J* = 4.2, 2.5 Hz, 2H), 6.25 (d, *J* = 1.1 Hz, 1H), 3.69 (d, *J* = 1.7 Hz, 3H), 3.05 (t, *J* = 7.6 Hz, 2H), 2.69 (t, *J* = 7.6 Hz, 2H), 2.43 (t, *J* = 1.9 Hz, 3H). **^{13}C NMR** (101 MHz, $CDCl_3$) δ 172.81, 160.97, 153.72, 152.37, 145.38, 124.74, 124.61, 118.37, 116.56, 114.47, 77.16, 51.85, 35.03, 30.72, 18.67. **HRMS** (ESI)**:** Calcd. for $C_{14}H_{14}O_4Na$ $[M+Na]^+$: 269.0784, found: 269.0782.

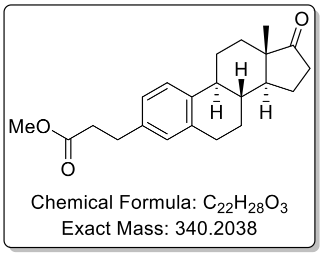

Methyl 3-((8R,9S,13S,14S)-13-methyl-17-oxo-7,8,9,11,12,13,14,15,16,17-decahydro-6H-cyclopenta[a]phenanthren-3-yl)propanoate (100): white solid, 292 mg, 86% yield, 81% linear selectivity. **^{1}H NMR** (400 MHz, $CDCl_3$) δ 7.36 – 7.12 (m, 1H), 7.12 – 6.79 (m, 2H), 3.66 (s, 3H), 3.06 – 2.73 (m, 4H), 2.60 (t, *J* = 9.4, 6.3 Hz, 2H), 2.53 – 2.34 (m, 2H), 2.31 – 2.17 (m, 1H), 2.17 – 1.86 (m, 4H), 1.64 – 1.35 (m, 6H), 0.89 (s, 3H). **^{13}C NMR** (101 MHz, $CDCl_3$) δ 173.15, 137.81, 137.51, 136.37, 128.75, 125.52, 125.35, 51.41, 50.31, 47.78, 44.12, 38.03, 35.66, 35.49, 31.47, 30.23, 29.22, 26.39, 25.58, 21.44, 13.68. **HRMS** (ESI)**:** Calcd. for $C_{22}H_{28}O_3Na$ $[M+Na]^+$: 363.1931, found: 363.1926.

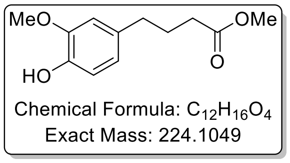

Methyl 4-(4-hydroxy-3-methoxyphenyl)butanoate (101): colorless oil liquid, 385 mg, 86% yield, 95% linear selectivity. **^{1}H NMR** (400 MHz, $CDCl_3$) δ 6.82 (d, *J* = 7.9 Hz, 1H), 6.74 – 6.60 (m, 2H), 5.71 (d, *J* = 1.0 Hz, 1H), 3.85 (s, 3H), 3.66 (s, 3H), 2.67 – 2.47 (m, 2H), 2.32 (t, *J* = 7.5 Hz, 2H), 1.99 – 1.80 (m, 2H). **^{13}C NMR** (101 MHz, $CDCl_3$) δ 174.14, 146.52, 143.89, 133.31, 121.06, 114.33, 111.12, 55.87, 51.54, 34.82, 33.35, 26.80.[35]

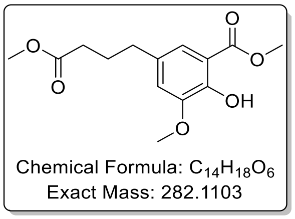

Methyl 2-hydroxy-3-methoxy-5-(4-methoxy-4-oxobutyl)benzoate (102): white solid, 560 mg, 99% yield, 94% linear selectivity. **^{1}H NMR** (400 MHz, $CDCl_3$) δ 10.86 (s, 1H), 7.24 (d, *J* = 1.9 Hz, 1H), 6.88 (d, *J* = 1.7 Hz, 1H), 3.94 (s, 3H), 3.90 (s, 3H), 3.67 (s, 3H), 2.80 – 2.47 (m, 2H), 2.34 (t, *J* = 7.4 Hz, 2H), 2.15 – 1.70 (m, 2H). **^{13}C NMR** (101 MHz, $CDCl_3$) δ 148.77, 137.76, 136.62, 136.54, 131.11, 127.56, 126.97, 126.69, 125.81, 116.22, 113.78, 112.01, 54.03, 39.91. **HRMS** (ESI)**:** Calcd. for $C_{14}H_{18}O_6Na$ $[M+Na]^+$: 305.0993, found: 305.0996.

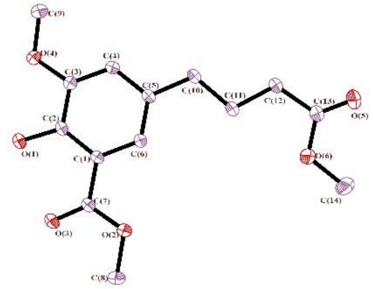

Single crystals of the product methyl 2-hydroxy-3-methoxy-5-(4-methoxy-4-oxobutyl)benzoate (**102**) were obtained from recrystallization in dichloromethane/*n*-hexane at room temperature. Hydrogen atoms have been omitted for clarity. Displacement ellipsoids correspond to 30% probability.

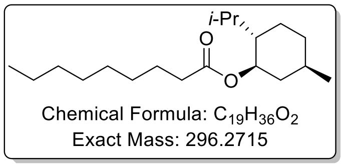

(1*R*,2*S*,5*R*)-2-Isopropyl-5-methylcyclohexyl nonanoate (103): colorless oil liquid, 586 mg, 99% yield, 89% linear selectivity. **^{1}H NMR** (400 MHz, $CDCl_3$) δ 4.68 (td, *J* = 10.9, 4.4 Hz, 1H), 2.27 (t, *J* = 7.4 Hz, 2H), 1.99 (dd, *J* = 7.2, 4.5 Hz, 1H), 1.88 (dtd, *J* = 13.9, 6.8, 2.4 Hz, 1H), 1.73 – 1.56 (m, 4H), 1.57 – 1.43 (m, 1H), 1.42 – 1.18 (m, 11H), 1.03 – 0.81 (m, 12H), 0.76 (d, *J* = 7.0 Hz, 3H). **^{13}C NMR** (101 MHz, $CDCl_3$) δ 173.44, 73.90, 47.15, 41.08, 34.83, 34.42, 31.91, 31.48, 29.33, 29.26, 29.24, 26.35, 25.24, 23.53, 22.74, 22.11, 20.84, 16.37, 14.16. **HRMS** (ESI)**:** Calcd. for $C_{19}H_{36}O_2Na$ $[M+Na]^+$: 319.2607, found: 319.2608.

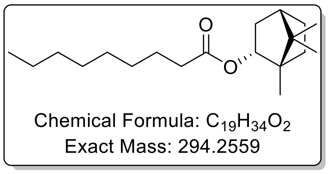

(1*S*,2*R*,4*S*)-1,7,7-Trimethylbicyclo[2.2.1]heptan-2-yl nonanoate (104): yellow oil liquid, 530 mg, 90% yield, 85% linear selectivity. **^{1}H NMR** (400 MHz, $CDCl_3$) δ 4.95 – 4.84 (m, 1H), 2.41 – 2.26 (m, 3H), 2.03 – 1.90 (m, 1H), 1.75 (dd, *J* = 13.7, 9.7 Hz, 1H), 1.70 – 1.58 (m, 3H), 1.28 (dd, *J* = 16.0, 12.2 Hz, 13H), 0.90 (ddd, *J* = 26.4, 17.7, 7.8 Hz, 12H). **^{13}C NMR** (101 MHz, $CDCl_3$) δ 173.91, 79.43, 48.69, 47.74, 44.92, 36.84, 34.64, 31.82, 29.25, 29.16, 28.05, 27.12, 25.14, 22.63, 19.67, 18.81, 14.05, 13.46. **HRMS** (ESI)**:** Calcd. for $C_{19}H_{34}O_2Na$ $[M+Na]^+$: 317.2445, found: 317.2451.

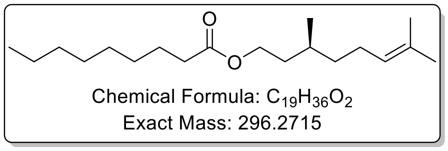

3,7-Dimethyloct-6-en-1-yl nonanoate (105): colorless oil liquid, 580 mg, 98% yield, 91% linear selectivity. **^{1}H NMR** (400 MHz, $CDCl_3$) δ 5.08 (t, *J* = 6.5 Hz, 1H), 4.23 – 3.99 (m, 2H), 2.28 (t, *J* = 7.5 Hz, 2H), 2.07 – 1.86 (m, 2H), 1.76 – 1.49 (m, 10H), 1.49 – 1.08 (m, 13H), 1.00 – 0.73 (m, 6H). **^{13}C NMR** (101 MHz, $CDCl_3$) δ 173.95, 131.29, 124.67, 62.78, 37.07, 35.59, 34.48, 31.91, 29.57, 29.33, 29.26, 29.23, 25.75, 25.48, 25.10, 22.73, 19.48, 17.68, 14.14. **HRMS** (ESI)**:** Calcd. for $C_{19}H_{36}O_2Na$ $[M+Na]^+$:319.2603, found: 319.2608.

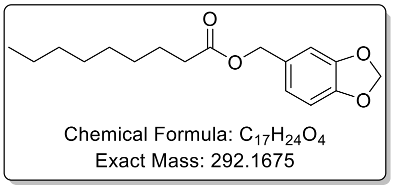

Benzo[*d*][1,3]dioxol-5-ylmethyl nonanoate (106): colorless oil liquid, 440 mg, 75% yield, 89% linear selectivity. **^{1}H NMR** (400 MHz, $CDCl_3$) δ 6.87 – 6.79 (m, 2H), 6.77 (d, *J* = 7.8 Hz, 1H), 5.94 (s, 2H), 5.00 (s, 2H), 2.32 (t, *J* = 7.6 Hz, 2H), 1.61 (dd, *J* = 14.5, 7.2 Hz, 2H), 1.25 (dd, *J* = 8.5, 5.7 Hz, 10H), 0.87 (t, *J* = 7.0 Hz, 3H). **^{13}C NMR** (101 MHz, $CDCl_3$) δ 173.71, 147.87, 147.65, 130.01, 122.24, 109.05, 108.27, 101.20, 66.08, 34.41, 31.87, 29.28, 29.20, 25.02, 22.71, 14.14. **HRMS** (ESI)**:** Calcd. for $C_{17}H_{24}O_4Na$ $[M+Na]^+$: 315.1565, found: 315.1567.

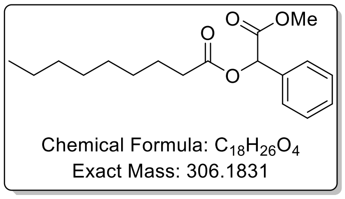

2-Methoxy-2-oxo-1-phenylethyl nonanoate (107): yellowish oil liquid, 490 mg, 80% yield, 7% linear selectivity. **^{1}H NMR** (400 MHz, $CDCl_3$) δ 7.52 – 7.43 (m, 2H), 7.43 – 7.33 (m, 3H), 5.93 (s, 1H), 3.71 (s, 3H), 2.68 – 2.26 (m, 2H), 1.80 – 1.56 (m, 2H), 1.48 – 1.13 (m, 10H), 0.87 (t, *J* = 6.8 Hz, 3H). **^{13}C NMR** (101 MHz, $CDCl_3$) δ 173.24, 169.48, 134.03, 129.28, 128.86, 127.70, 74.34, 52.63, 34.06, 31.89, 29.28, 29.19, 29.12, 24.88, 22.72, 14.17. **HRMS** (ESI)**:** Calcd. for $C_{18}H_{26}O_4Na$ $[M+Na]^+$: 329.1723, found: 329.1723.

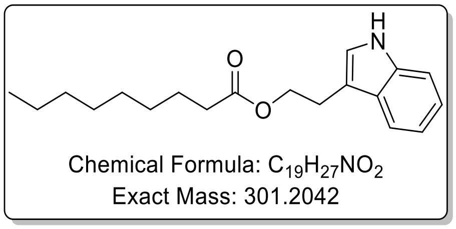

2-(1*H*-Indol-3-yl)ethyl nonanoate (108): yellow oil liquid, 542 mg, 90% yield, 88% linear selectivity. **^{1}H NMR** (400 MHz, $CDCl_3$) δ 8.14 (s, 1H), 7.61 (d, *J* = 7.8 Hz, 1H), 7.27 (d, *J* = 8.0 Hz, 1H), 7.16 (t, *J* = 7.1 Hz, 1H), 7.13 – 7.06 (m, 1H), 6.92 (d, *J* = 1.6 Hz, 1H), 4.34 (t, *J* = 7.2 Hz, 2H), 3.07 (t, *J* = 7.2 Hz, 2H), 2.29 (t, *J* = 7.5 Hz, 3H), 1.75 – 1.49 (m, 3H), 1.25 (s, 14H), 0.87 (t, *J* = 6.8 Hz, 4H). **^{13}C NMR** (101 MHz, $CDCl_3$) δ 174.28, 136.28, 127.50, 122.17, 122.03, 119.38, 118.80, 111.87, 111.27, 64.59, 34.49, 31.88, 29.30, 29.21, 29.19, 25.04, 24.88, 22.72, 14.17. **HRMS** (ESI)**:** Calcd. for $C_{19}H_{27}NO_2Na$ $[M+Na]^+$: 324.1924, found: 324.1934.

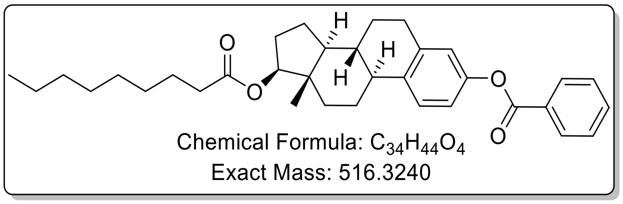

(8*R*,9*S*,13*S*,14*S*,17*S*)-13-Methyl-17-(nonanoyloxy)-7,8,9,11,12,13,14,15,16,17-decahydro-6*H*-cyclopenta[*a*]phenanthren-3-yl benzoate (109): white solid, 457 mg, 88% yield, 87% linear selectivity. **^{1}H NMR** (400 MHz, $CDCl_3$) δ 8.24 – 8.09 (m, 2H), 7.61 (t, *J* = 7.4 Hz, 1H), 7.49 (t, *J* = 7.6 Hz, 2H), 7.32 (d, *J* = 8.5 Hz, 1H), 6.97 (dd, *J* = 8.4, 2.3 Hz, 1H), 6.92 (d, *J* = 1.8 Hz, 1H), 4.71 (t, *J* = 8.4 Hz, 1H), 2.98 – 2.73 (m, 2H), 2.47 – 2.11 (m, 6H), 1.98 – 1.82 (m, 2H), 1.79 – 1.70 (m, 1H), 1.68 – 1.58 (m, 2H), 1.58 – 1.47 (m, 3H), 1.46 – 1.37 (m, 3H), 1.36 – 1.23 (m, 10H), 0.93 – 0.79 (m, 6H). **^{13}C NMR** (101 MHz, $CDCl_3$) δ 174.02, 165.51, 148.80, 138.32, 138.01, 133.56, 130.23, 129.82, 128.61, 126.57, 121.71, 118.79, 82.46, 49.94, 44.12, 43.03, 38.34, 37.01, 34.71, 31.92, 29.64, 29.34, 29.25, 27.71, 27.15, 26.18, 25.24, 23.39, 22.75, 14.21, 12.20. **HRMS** (ESI)**:** Calcd. for $C_{34}H_{44}O_4Na$ $[M+Na]^+$: 539.3128, found: 539.3132.

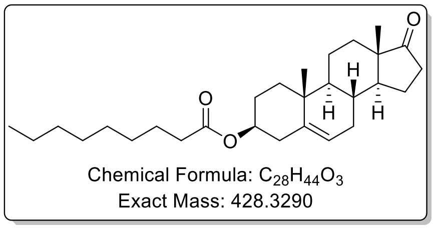

(3*S*,8*R*,9*S*,10*R*,13*S*,14*S*)-10,13-Dimethyl-17-oxo-2,3,4,7,8,9,10,11,12,13,14,15,16,17-tetradecahydro-1*H*-cyclopenta[*a*]phenanthren-3-yl nonanoate (110): yellowish oil liquid, 818 mg, 96% yield, 90% linear selectivity. **^{1}H NMR** (400 MHz, CDCl3) δ 5.41 (d, *J* = 4.9 Hz, 1H), 4.61 (dq, *J* = 5.8, 4.8 Hz, 1H), 2.46 (dd, *J* = 19.1, 8.7 Hz, 1H), 2.38 – 2.21 (m, 4H), 2.09 (ddd, *J* = 20.7, 8.2, 2.2 Hz, 2H), 2.02 – 1.91 (m, 1H), 1.92 – 1.79 (m, 3H), 1.76 – 1.44 (m, 8H), 1.28 (d, *J* = 6.3 Hz, 13H), 1.05 (s, 4H), 0.93 – 0.82 (m, 6H). **^{13}C NMR** (101 MHz, $CDCl_3$) δ 173.14, 139.93, 121.75, 77.36, 73.34, 51.66, 50.12, 47.44, 38.08, 36.92, 36.69, 35.75, 34.60, 31.77, 31.43, 31.38, 30.74, 29.18, 29.08, 29.07, 27.70, 25.00, 22.60, 21.84, 20.29, 19.30, 14.06, 13.48. **HRMS** (ESI)**:** Calcd. for $C_{28}H_{44}O_3Na$ $[M+Na]^+$: 451.3179, found: 451.3183.

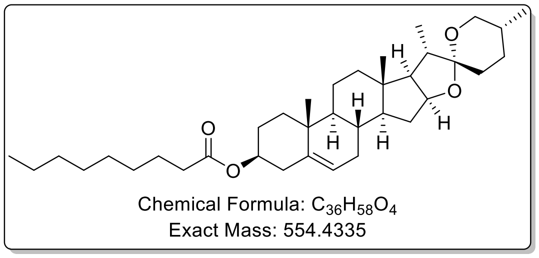

(4S,5'R,6aR,6bS,8aS,8bR,9S,10R,11aS,12aS,12bS)-5',6a,8a,9-tetramethyl-1,3,3',4,4',5,5',6,6a,6b,6',7,8,8a,8b,9,11a,12,12a,12b-icosahydrospiro[naphtho[2',1':4,5]indeno[2,1-b]furan-10,2'-pyran]-4-yl nonanoate (111): white solid, 480 mg, 86% yield, 87% linear selectivity. **^{1}H NMR** (400 MHz, $CDCl_3$) δ 5.37 (d, *J* = 3.9 Hz, 1H), 4.79 – 4.48 (m, 1H), 4.41 (dd, *J* = 14.8, 7.4 Hz, 1H), 3.52 – 3.43 (m, 1H), 3.37 (t, *J* = 10.9 Hz, 1H), 2.41 – 2.22 (m, 4H), 2.11 – 1.93 (m, 2H), 1.91 – 1.81 (m, 3H), 1.76 (dd, *J* = 18.6, 11.4 Hz, 2H), 1.68 – 1.41 (m, 12H), 1.25 (d, *J* = 24.6 Hz, 13H), 1.14 (ddd, *J* = 16.3, 10.6, 4.9 Hz, 2H), 1.03 (d, *J* = 9.5 Hz, 2H), 0.95 (dd, *J* = 14.0, 6.9 Hz, 4H), 0.88 (t, *J* = 6.6 Hz, 3H), 0.79 (s, 6H). **^{13}C NMR** (101 MHz, $CDCl_3$) δ 173.42, 139.89, 122.42, 109.38, 80.93, 73.69, 66.96, 62.22, 56.57, 50.07, 41.74, 40.38, 39.86, 38.26, 37.10, 36.87, 34.83, 32.17, 31.97, 31.93, 31.54, 30.43, 29.35, 29.25, 28.94, 27.91, 25.19, 22.77, 20.94, 19.47, 17.27, 16.41, 14.65, 14.23. **HRMS** (ESI)**:** Calcd. for $C_{36}H_{58}O_4Na$ $[M+Na]^+$: 577.4227, found: 577.4231.

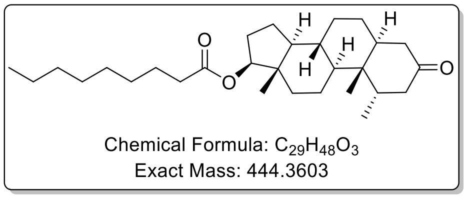

(1*S*,5*S*,8*R*,9*S*,10*S*,13*S*,14*S*,17*S*)-1,10,13-Trimethyl-3-oxohexadecahydro-1*H*-cyclopenta[*a*]phenanthren-17-yl nonanoate (112): white solid, 782 mg, 88% yield, 89% linear selectivity. **^{1}H NMR** (400 MHz, $CDCl_3$) δ 4.62 (t, *J* = 8.5 Hz, 1H), 2.70 (dd, *J* = 14.5, 5.9 Hz, 1H), 2.47 – 2.25 (m, 2H), 2.21 (d, *J* = 14.0 Hz, 1H), 2.18 – 2.01 (m, 2H), 1.88 – 1.56 (m, 6H), 1.50 (dd, *J* = 14.0, 7.3 Hz, 3H), 1.43 – 1.19 (m, 15H), 1.19 – 0.94 (m, 7H), 0.94 – 0.66 (m, 10H). **^{13}C NMR** (101 MHz, $CDCl_3$) δ 212.11, 173.92, 82.37, 50.69, 48.51, 45.89, 44.92, 42.79, 39.85, 39.49, 37.82, 36.84, 35.28, 34.58, 31.80, 31.05, 29.22, 29.13, 28.69, 27.51, 25.12, 23.55, 22.63, 20.04, 14.68, 14.40, 14.09, 12.18. **HRMS** (ESI)**:** Calcd. for $C_{29}H_{48}O_3Na$ $[M+Na]^+$: 467.3498, found: 467.3496.

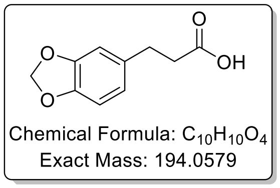

3-(benzo[d][1,3]dioxol-5-yl)propanoic acid (113): white solid, 165 mg, 85% yield, 83% linear selectivity. **^{1}H NMR** (400 MHz, $CDCl_3$) δ 11.20 (s, 1H), 6.86 – 6.54 (m, 3H), 5.91 (s, 2H), 2.86 (t, *J* = 7.7 Hz, 2H), 2.62 (t, *J* = 7.7 Hz, 2H). **^{13}C NMR** (101 MHz, $CDCl_3$) δ 179.46, 147.78, 146.13, 134.04, 121.21, 108.87, 108.40, 100.97, 36.06, 30.42. **HRMS** (ESI)**:** Calcd. for $C_{10}H_{10}O_4Na$ $[M+Na]^+$: 217.0471, found: 217.0470.

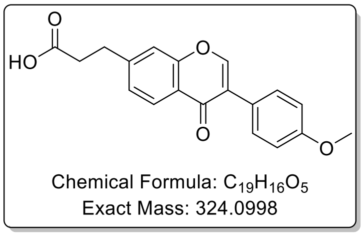

3-(3-(4-methoxyphenyl)-4-oxo-4H-chromen-7-yl)propanoic acid (114): white solid, 277 mg, 85% yield, 89% linear selectivity. **^{1}H NMR** (400 MHz, DMSO) δ 12.42 (s, 1H), 8.42 (s, 1H), 8.03 (d, *J* = 8.2 Hz, 1H), 7.74 – 7.47 (m, 3H), 7.38 (dd, *J* = 8.2, 1.2 Hz, 1H), 6.99 (d, *J* = 8.8 Hz, 2H), 3.79 (s, 3H), 2.99 (t, *J* = 7.5 Hz, 2H), 2.66 (t, *J* = 7.6 Hz, 2H). **^{13}C NMR** (101 MHz, DMSO) δ 175.15, 173.58, 159.07, 155.70, 153.73, 148.14, 130.10, 126.09, 125.44, 124.09, 123.50, 122.05, 117.39, 113.66, 55.16, 34.47, 30.29. **HRMS** (ESI)**:** Calcd. for $C_{19}H_{16}O_5Na$ $[M+Na]^+$: 347.0890, found: 347.0887.

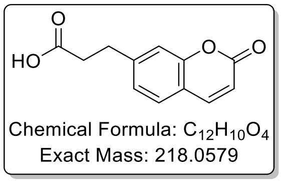

3-(2-oxo-2H-chromen-7-yl)propanoic acid (115): white solid, 164 mg, 75% yield, 87% linear selectivity. **^{1}H NMR** (400 MHz, DMSO) δ 12.28 (s, 1H), 8.01 (d, *J* = 9.5 Hz, 1H), 7.61 (d, *J* = 7.9 Hz, 1H), 7.40 – 7.05 (m, 2H), 6.42 (d, *J* = 9.5 Hz, 1H), 2.92 (t, *J* = 7.5 Hz, 2H), 2.61 (t, *J* = 7.6 Hz, 2H). **^{13}C NMR** (101 MHz, DMSO) δ 173.58, 160.13, 153.62, 146.04, 144.14, 128.26, 124.90, 116.90, 115.85, 115.32, 34.61, 30.29. **HRMS** (ESI)**:** Calcd. for $C_{12}H_{10}O_4Na$ $[M+Na]^+$: 241.0471, found: 241.0469.

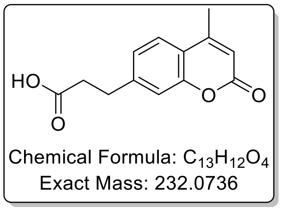

3-(4-methyl-2-oxo-2H-chromen-7-yl)propanoic acid (116): white solid, 167 mg, 72% yield, 82% linear selectivity. **^{1}H NMR** (400 MHz, DMSO) δ 12.28 (s, 1H), 7.64 (d, *J* = 8.5 Hz, 1H), 7.45 – 7.04 (m, 2H), 6.31 (s, 1H), 2.92 (t, *J* = 7.5 Hz, 2H), 2.61 (t, *J* = 7.5 Hz, 2H), 2.39 (s, 3H). **^{13}C NMR** (101 MHz, DMSO) δ 173.58, 159.90, 153.13, 152.99, 145.98, 125.17, 124.67, 117.70, 115.92, 113.55, 34.61, 30.14, 18.04. **HRMS** (ESI)**:** Calcd. for $C_{13}H_{12}O_4Na$ [M+Na]$^+$: 255.0628, found: 255.0639.

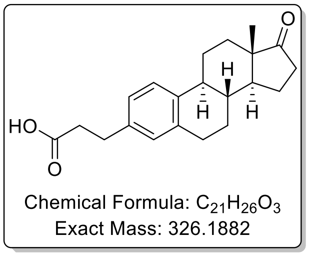

3-((8R,9S,13S,14S)-13-methyl-17-oxo-7,8,9,11,12,13,14,15,16,17-decahydro-6H-cyclopenta[a]phenanthren-3-yl)propanoic acid (117): white solid, 293 mg, 90% yield, 83% linear selectivity. **^{1}H NMR** (400 MHz, DMSO) δ 12.17 (s, 1H), 7.16 (d, *J* = 7.9 Hz, 1H), 6.96 (d, *J* = 8.4 Hz, 1H), 6.91 (s, 1H), 2.92 – 2.64 (m, 4H), 2.57 – 2.27 (m, 4H), 2.18 (s, 1H), 2.01 (ddd, *J* = 34.9, 23.1, 9.3 Hz, 3H), 1.77 (d, *J* = 8.1 Hz, 1H), 1.65 – 1.23 (m, 6H), 0.82 (s, 3H). **^{13}C NMR** (101 MHz, DMSO) δ 173.87, 138.01, 137.23, 135.99, 128.61, 125.56, 125.20, 49.73, 47.35, 43.78, 37.73, 35.39, 35.34, 31.44, 29.95, 28.97, 26.12, 25.38, 21.19, 13.52. **HRMS** (ESI)**:** Calcd. for $C_{21}H_{26}O_3Na$ [M+Na]$^+$: 349.1774, found: 349.1772.

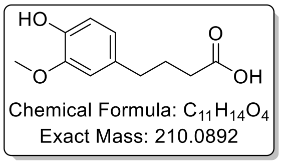

4-(4-hydroxy-3-methoxyphenyl)butanoic acid (118): white solid, 290 mg, 69% yield, 97% linear selectivity. **^{1}H NMR** (400 MHz, DMSO) δ 12.07 (s, 1H), 8.69 (s, 1H), 6.72 (dd, *J* = 13.8, 4.8 Hz, 2H), 6.57 (dd, *J* = 8.0, 1.7 Hz, 1H), 3.75 (s, 3H), 2.64 – 2.35 (m, 2H), 2.21 (t, *J* = 7.4 Hz, 2H), 1.88 – 1.62 (m, 2H). **^{13}C NMR** (101 MHz, DMSO) δ 174.67, 147.61, 144.74, 132.60, 120.61, 115.51, 112.61, 55.65, 34.27, 33.29, 26.81. **HRMS** (ESI)**:** Calcd. for $C_{11}H_{14}O_4Na$ [M+Na]$^+$: 233.0784, found: 233.0785.

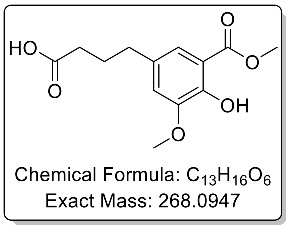

4-(4-hydroxy-3-methoxy-5-(methoxycarbonyl)phenyl)butanoic acid (119): white solid, 344 mg, 64% yield, 94% linear selectivity. **^{1}H NMR** (400 MHz, DMSO) δ 12.09 (s, 1H), 10.43 (s, 1H), 7.14 (d, *J* = 1.9 Hz, 1H), 7.05 (d, *J* = 1.8 Hz, 1H), 3.88 (s, 3H), 3.80 (s, 3H), 2.52 (t, *J* = 10.0, 5.2 Hz, 2H), 2.22 (t, *J* = 7.4 Hz, 2H), 1.89 – 1.47 (m, 2H). **^{13}C NMR** (101 MHz, DMSO) δ 174.42, 169.93, 149.14, 148.20, 132.12, 119.70, 117.60, 112.36, 55.90, 52.50, 33.99, 33.11, 26.35. **HRMS** (ESI)**:** Calcd. for $C_{13}H_{16}O_6Na$ [M+Na]$^+$: 291.0839, found: 291.0841.

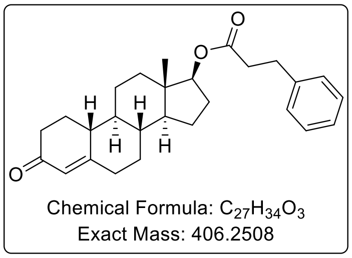

(8R,9S,10R,13S,14S,17S)-13-methyl-3-oxo-2,3,6,7,8,9,10,11,12,13,14,15,16,17-tetradecahydro-1H-cyclopenta[a]phenanthren-17-yl 3-phenylpropanoate (120): white solid, 1.53 g, 94% yield, 87% linear selectivity. **^{1}H NMR** (400 MHz, $CDCl_3$) δ 7.33 – 7.24 (m, 2H), 7.19 (d, *J* = 7.3 Hz, 3H), 5.82 (s, 1H), 4.89 –

4.31 (m, 1H), 2.94 (t, *J* = 7.7 Hz, 2H), 2.63 (t, *J* = 7.8 Hz, 2H), 2.51 – 2.34 (m, 2H), 2.30 – 2.19 (m, 3H), 2.18 – 2.11 (m, 1H), 2.10 – 1.98 (m, 1H), 1.86 – 1.77 (m, 2H), 1.72 – 1.59 (m, 2H), 1.56 – 1.41 (m, 3H), 1.39 – 1.29 (m, 2H), 1.27 – 1.14 (m, 2H), 1.13 – 0.98 (m, 2H), 0.89 – 0.81 (m, H), 0.79 (s, 2H). **^{13}C NMR** (101 MHz, $CDCl_3$) δ 199.80, 172.85, 166.40, 140.48, 128.45, 128.28, 126.22, 124.63, 82.51, 49.47, 49.34, 42.69, 42.50, 40.15, 36.54, 36.51, 36.01, 35.41, 31.08, 30.61, 27.41, 26.58, 25.97, 23.32, 12.03. **HRMS** (ESI)**:** Calcd. for $C_{27}H_{34}O_3Na$ $[M+Na]^+$: 429.2391, found: 429.2400.

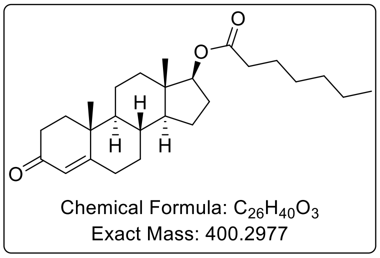

(8R,9S,10R,13S,14S,17S)-10,13-dimethyl-3-oxo-2,3,6,7,8,9,10,11,12,13,14,15,16,17-tetradecahydro-1H-cyclopenta[a]phenanthren-17-yl heptanoate (121): yellow oil liquid, 1.39 g, 87% yield, 83% linear selectivity. **^{1}H NMR** (400 MHz, $CDCl_3$) δ 5.73 (s, 1H), 4.77 – 4.45 (m, 1H), 2.49 – 2.34 (m, 3H), 2.33 – 2.25 (m, 3H), 2.24 – 2.11 (m, 1H), 2.07 – 1.99 (m, 1H), 1.90 – 1.82 (m, 1H), 1.81 – 1.74 (m, 1H), 1.74 – 1.54 (m, 6H), 1.54 – 1.45 (m, 1H), 1.44 – 1.25 (m, 8H), 1.20 (s, 3H), 1.17 – 1.12 (m, 1H), 1.11 – 1.01 (m, 2H), 1.00 – 0.94 (m, 1H), 0.93 – 0.86 (m, 3H), 0.85 (d, *J* = 3.5 Hz, 3H). **^{13}C NMR** (101 MHz, $CDCl_3$) δ 199.30, 173.74, 170.91, 123.86, 82.08, 53.66, 50.20, 42.44, 38.55, 36.59, 35.64, 35.34, 34.48, 33.86, 32.69, 31.44, 31.40, 28.73, 27.45, 25.00, 23.43, 22.44, 20.48, 17.33, 13.99, 12.00. **HRMS** (ESI)**:** Calcd. for $C_{26}H_{40}O_3Na$ $[M+Na]^+$: 423.2879, found: 423.2870.

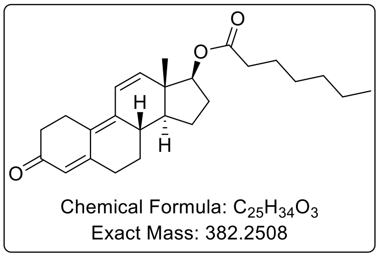

(8S,13S,14S,17S)-13-methyl-3-oxo-2,3,6,7,8,13,14,15,16,17-decahydro-1H-cyclopenta[a]phenanthren-17-yl heptanoate (122): yellow oil liquid, 553 mg, 72% yield, 93% linear selectivity. **^{1}H NMR** (400 MHz, $CDCl_3$) δ 6.33 (dd, *J* = 29.5, 9.9 Hz, 2H), 5.70 (s, 1H), 4.81 – 4.65 (m, 1H), 2.82 – 2.64 (m, 2H), 2.58 – 2.43 (m, 2H), 2.38 (t, *J* = 7.3 Hz, 3H), 2.26 (t, *J* = 7.4 Hz, 3H), 1.88 – 1.77 (m, 1H), 1.74 – 1.62 (m, 1H), 1.61 – 1.47 (m, 4H), 1.32 – 1.15 (m, 8H), 0.87 (s, 3H), 0.81 (t, *J* = 8.6, 4.8 Hz, 3H). **^{13}C NMR** (101 MHz, $CDCl_3$) δ 198.90, 173.66, 156.08, 141.71, 141.33, 127.15, 123.66, 123.50, 78.04, 47.54, 44.90, 37.38, 36.55, 34.36, 31.35, 31.30, 28.70, 27.48, 26.81, 24.92, 24.25, 23.07, 22.39, 14.35, 13.96. **HRMS** (ESI)**:** Calcd. for $C_{25}H_{34}O_3Na$ $[M+Na]^+$: 405.2398, found: 405.2400.

Supplementary Note 5: Upscaling reaction for TON and TOF experiments

TON experiment

Under argon atmosphere, $Pd(acac)_2$ (0.21 mg, 0.00069 mmol), L11 (324 mg, 0.5 mmol), PTSA·H_2O (760 mg, 4 mmol), and MeOH (1200 ml, about 30 mol) were introduced into a 1.7 L of steel autoclave. Then ethene (56 g, 2 mol) and CO were successively introduced into the autoclave. The reaction mixture was carried out at 100 ºC for 48 h, during which the total pressure was retained at 8 MPa. Afterwards, the autoclave was cooled to room temperature and depressurized slowly. Finally, the quantity and chemoselectivity of methyl propionate were determined by gas chromatography (Agilent 7890A GC equipped with a HP-5 capillary column with 5 wt.% phenyl groups and the FID detector) based on ethene and using *n*-decane as the internal standard. Weight: 145.3 g; Yield: 82.5%; TON (turnover number): 2,390,000; TOF (turnover frequency): 49,792 h^{-1}; chemoselectivity: >99%.

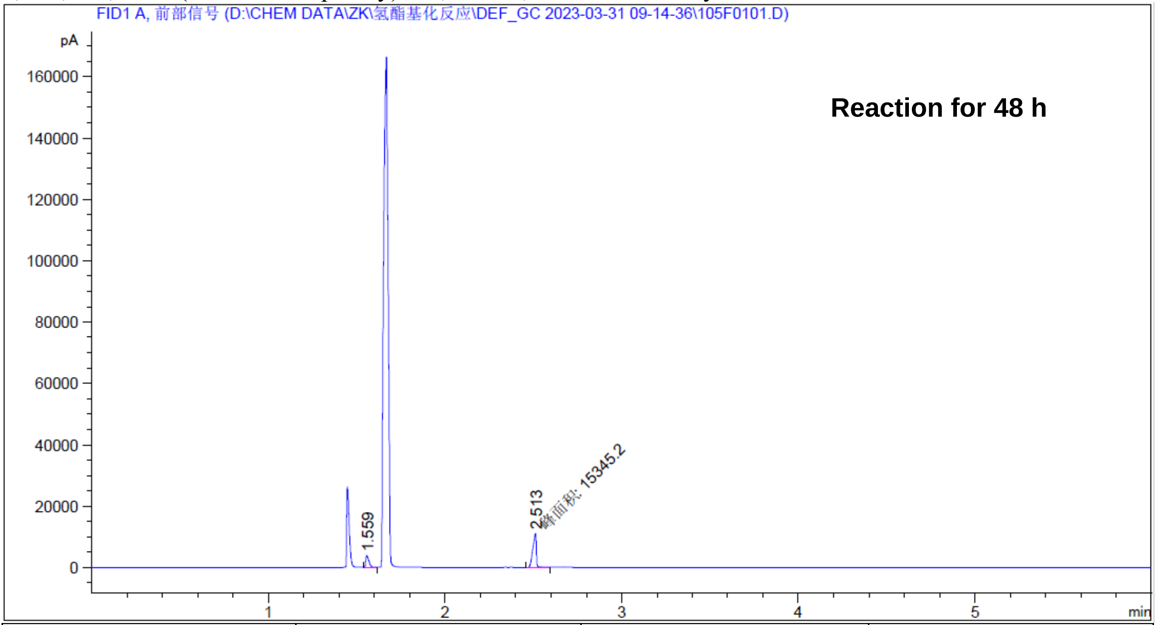

Peak	Ret. Time (min)	Area	Area (%)
Methyl propionate	1.559	5014.9	24.6
n-decane	2.513	15345.2	75.4
Total		20360.1	100

Supplementary Fig. 13 GC spectrum after 48 h reaction.

TOF experiment

Under argon atmosphere, Pd(acac)$_2$ (0.21 mg, 0.00069 mmol), L11 (324 mg, 0.5 mmol), PTSA·H_2O (760 mg, 4 mmol), and MeOH (1200 ml, about 30 mol) were introduced into a 1.7 L of steel autoclave. Then ethene (56 g, 2 mol) and CO were successively introduced into the autoclave. The reaction mixture was carried out at 100 ºC for 18 h, during which the total pressure was retained at 8 MPa. Afterwards, the autoclave was cooled to room temperature and depressurized slowly. Finally, the quantity and chemoselectivity of methyl propionate were determined by gas chromatography (Agilent 7890A GC equipped with a HP-5 capillary column with 5 wt.% phenyl groups and the FID detector) based on ethene and using *n*-decane as the internal standard. Weight: 109.4 g; Yield: 62%; TON (turnover number): 1,800,000; TOF (turnover frequency): 100,000 h^{-1}; chemoselectivity: >99%.

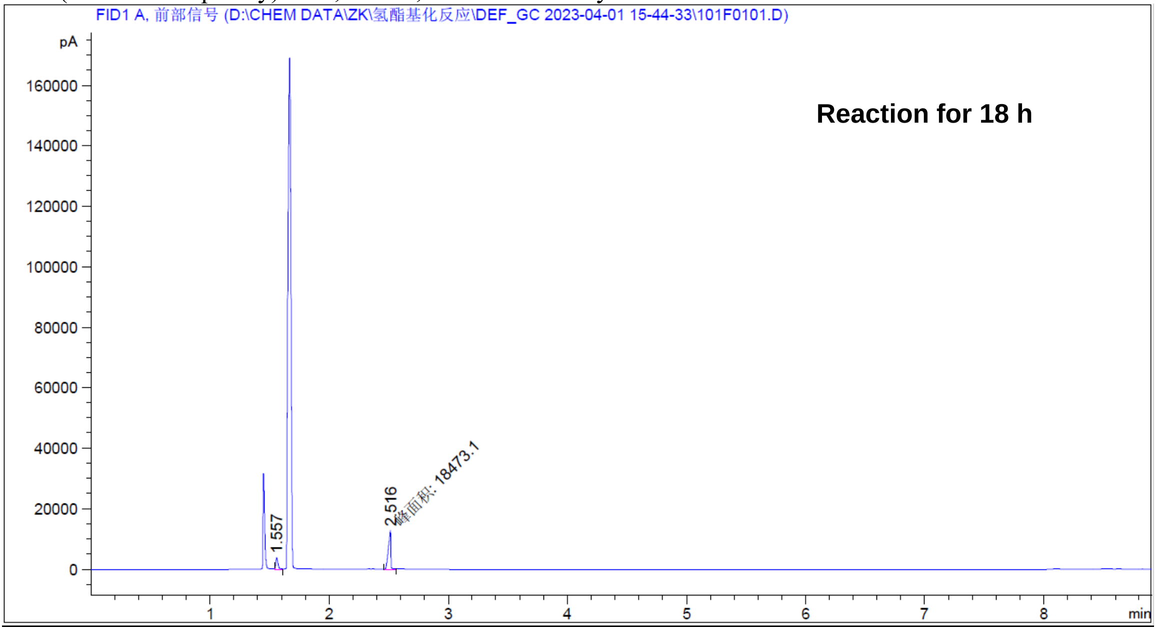

Peak	Ret. Time (min)	Area	Area (%)
Methyl propionate	1.557	4608.9	19.97
n-decane	2.516	18473.1	80.03
Total		193893	100

Supplementary Fig. 14 GC spectrum after 18 h reaction.

Supplementary Note 6: Comparison of oxygen-resistance stability between L1 and L11

Stability comparison of L1 and L11 at air condition and room temperature

Two 10 ml tube was charged with a certain amount of L1 and L11, separately, keeping them under air condition for the required days at room temperature. After treated with the required time, L1 and L11 was subjected to ^{31}P NMR analysis.

For L1

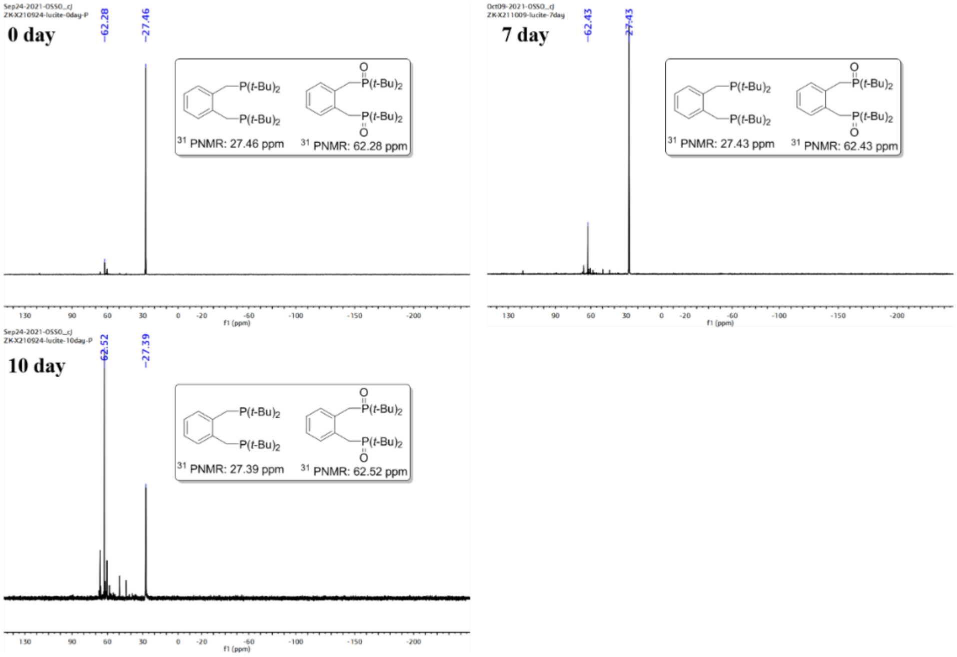

Supplementary Fig. 15 Stability tests of L1 at air condition and room temperature.

For L11

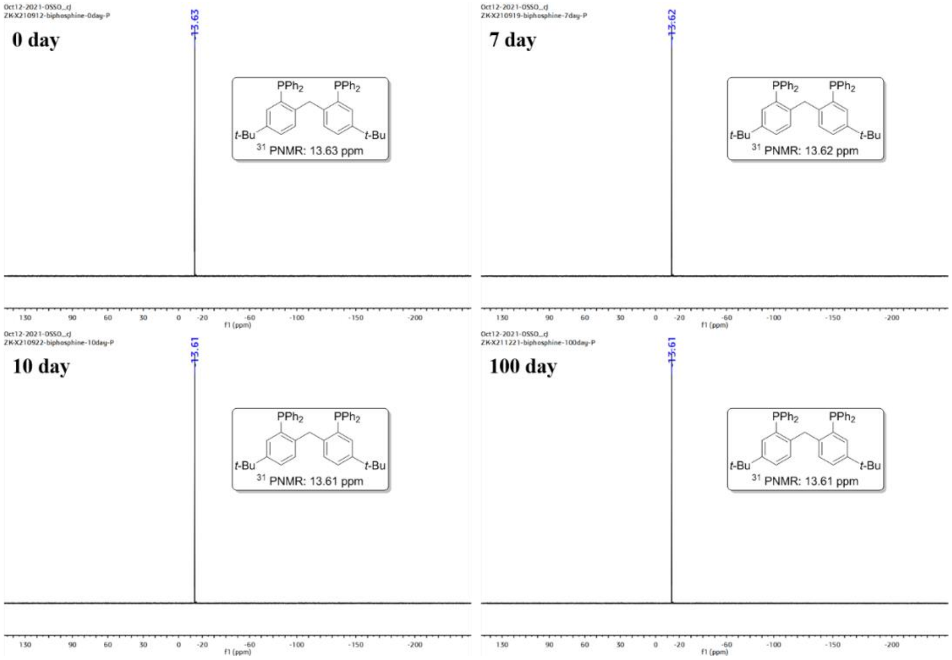

Supplementary Fig. 16 Stability tests of L11 at air condition and room temperature.

Stability comparison of L1 and L11 under reaction condition

1,4-dioxane as the solvent

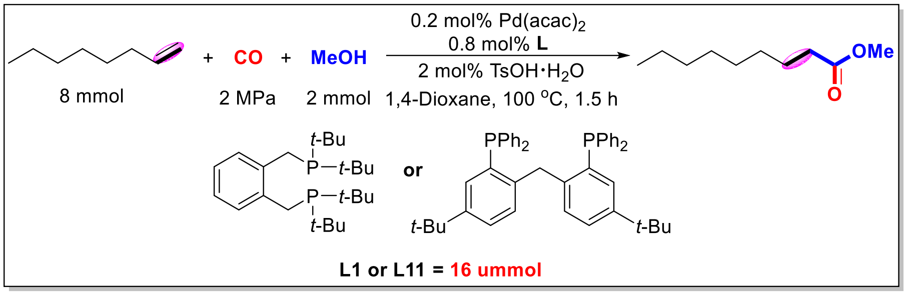

An 80 ml steel autoclave was charged with Pd(acac)$_2$ (1.2 mg, 0.2 mol%), **L1** or **L11** (0.8 mol%), PTSA·H_2O (7.6 mg, 2 mol%), and 1,4-Dioxane (4 ml). 1-octene (8.0 mmol) and MeOH (2.0 mmol) were introduced into the autoclave. After the autoclave was purged with CO (1 MPa) for the required times at room temperature and then pressurized with CO to 2 MPa, the autoclave was sealed and put into a preheated reactor, stirring at 100 °C for 1.5 h. Afterwards, the autoclave was cooled to room temperature and depressurized slowly. Subsequently, the reaction mixture was analyzed by gas chromatography (Agilent 7890A GC equipped with a HP-5 capillary column with 5 wt.% phenyl groups and the FID detector). The yield and regioselectivity was obtained by GC analysis using *n*-decane as the internal standard. O_2 content in the autoclave was estimated on the basis of the ideal gas models.

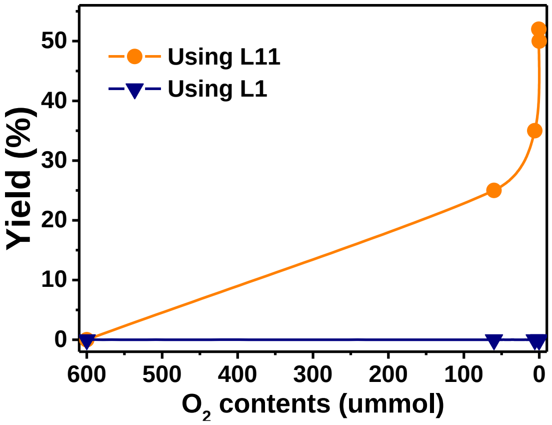

Supplementary Fig. 17 Stability comparison of L1 and L11 when using 1,4-dioxane as the solvent.

MeOH as the solvent

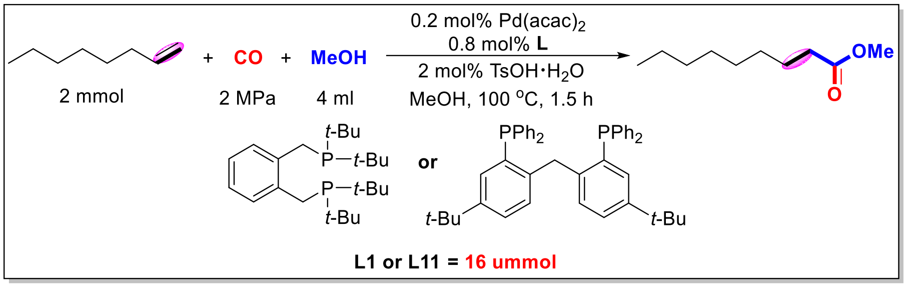

An 80 ml steel autoclave was charged with Pd(acac)$_2$ (1.2 mg, 0.2 mol%), **L1** or **L11** (0.8 mol%), PTSA·H_2O (7.6 mg, 2 mol%), and MeOH (4 ml). 1-octene (2.0 mmol) were introduced into the autoclave. After the autoclave was purged with CO (1 MPa) for the required times at room temperature and then pressurized with CO to 2 MPa, the autoclave was sealed and put into a preheated reactor, stirring at 100 °C for 1.5 h. Afterwards, the autoclave was cooled to room temperature and depressurized slowly. Subsequently, the reaction mixture was analyzed by gas chromatography (Agilent 7890A GC equipped with a HP-5 capillary column with 5 wt.% phenyl groups and the FID detector). The yield and regioselectivity was obtained by GC analysis using *n*-decane as the internal standard. O_2 content in the autoclave was estimated on the basis of the ideal gas models.

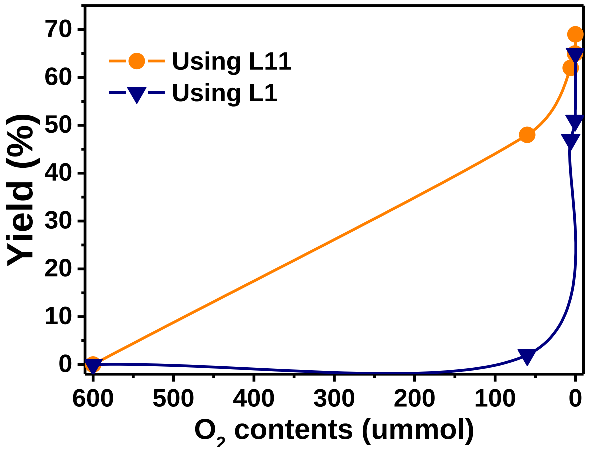

Supplementary Fig. 18 Stability comparison of L1 and L11 when using MeOH as the solvent.

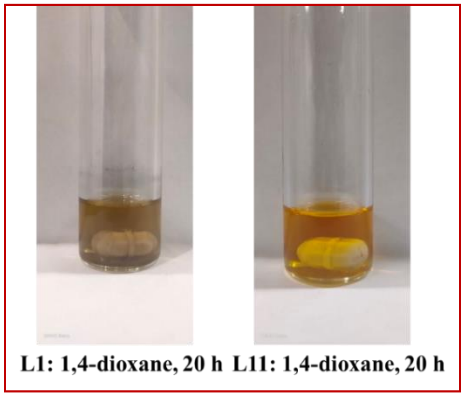

Supplementary Fig. 19 Stability comparison of L1 and L11 when using 1,4-dioxane as the solvent. Reaction condition: After the autoclave was purged with CO (1 MPa) for three times at room temperature and then pressurized with CO to 2 MPa, the autoclave was sealed and put into a preheated reactor, stirring at 100 ^{o}C for 20 h.

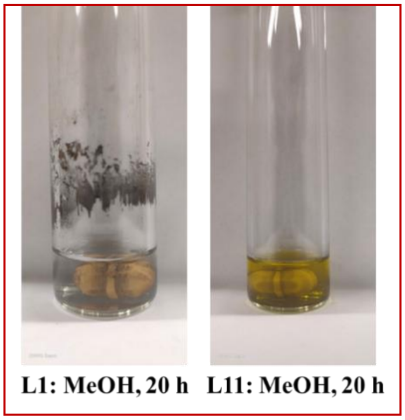

Supplementary Fig. 20 Stability comparison of L1 and L11 when using MeOH as the solvent. Reaction condition: After the autoclave was purged with CO (1 MPa) for three times at room temperature and then pressurized with CO to 2 MPa, the autoclave was sealed and put into a preheated reactor, stirring at 100 ºC for 20 h.

Supplementary Note 7: Computational investigations

The geometries of the involved L1 and L11 oxidation in gas phase, molecules and aggregates for 1-butene methoxycarbonylation in solution phase were fully optimised, at Density Functional Theory (DFT) level, using B3PW91[36-39] hybrid functional, coupled with a mixed basis set, consisting of a combination of Pople's triple-ζ 6-311+G** basis set for light atoms (H, C, N, O, and P) and the Stuttgart/Dresden Electron Core Potential SDD for the metal atom (Pd). For reactions in solutions, the D3 version of Grimme's empirical dispersion with Becke-Johnson damping[40] was included. This method had been successfully used in the Literature for similar investigations by Bühl and co-workers.[41-44] Thermochemical quantities were computed at $T = 373.15$ K and $p = 1.00$ atm.

The optimised geometries were submitted to vibrational frequencies calculation (in harmonic approximation) to check the absence of negative frequencies for minima and the presence of one negative frequency for transition states.

Solvent effects were accounted by means of the Polarizable Continuum Model (PCM) using the integral equation formalism variant (IEFPCM).[45] The values of static dielectric constant (ε) and refraction index (n^2) were taken from the Literature.[46] The cavitation radii were the standard UFF radii, scaled by a factor $\alpha = 1.1$; the scale factor for the metal atom and other atoms involved in the reaction process were modified to check the consistency of the results.

Integration grid for the electronic density was set to 250 radial shells and 974 angular points. Accuracy for the two-electron integrals was set to 10^{-14} a.u.. The convergence criteria for Self-Consistent Field were set to 10^{-12} for root mean square (RMS) change in density matrix and 10^{-10} for maximum change in density matrix. Convergence criteria for geometry optimisations were set to 2×10^{-6} a.u. for maximum force, 1×10^{-6} a.u. for RMS force, 6×10^{-6} a.u. for maximum displacement and 4×10^{-6} a.u. for RMS displacement.

All calculations were performed using GAUSSIAN G16.C01 package.[47]

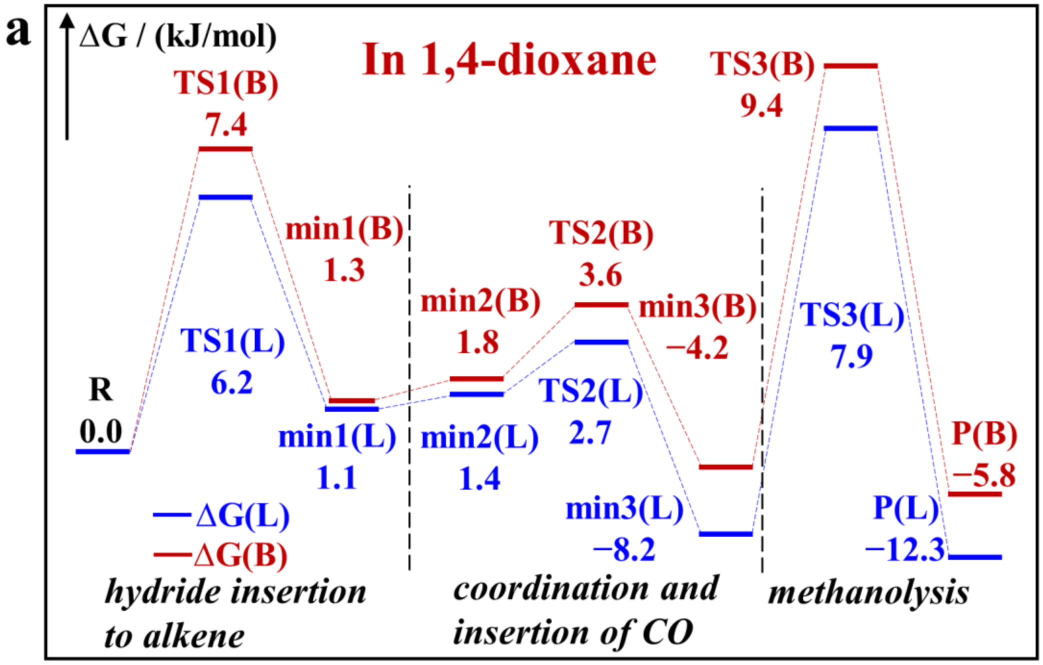

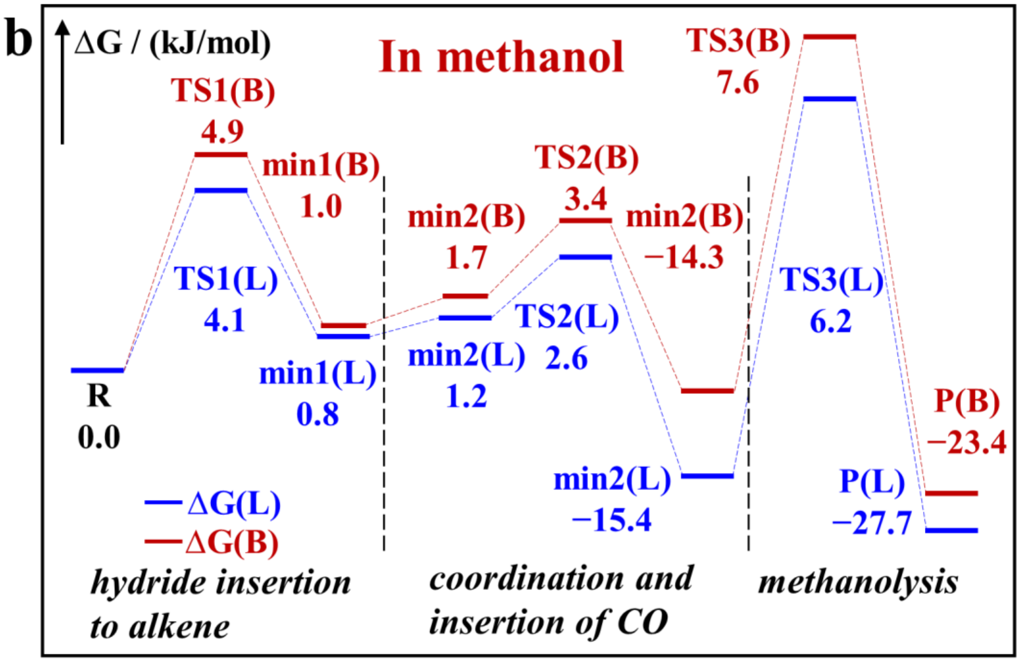
b
ΔG / (kJ/mol)
In methanol
TS1(B)
4.9
min1(B)
1.0
TS1(L)
4.1
min1(L)
0.8
R
0.0
min2(B)
1.7
min2(L)
1.2
TS2(B)
3.4
TS2(L)
2.6
min2(B)
−14.3
min2(L)
−15.4
TS3(B)
7.6
TS3(L)
6.2
P(B)
−23.4
P(L)
−27.7
ΔG(L)
ΔG(B)
hydride insertion to alkene
coordination and insertion of CO
methanolysis

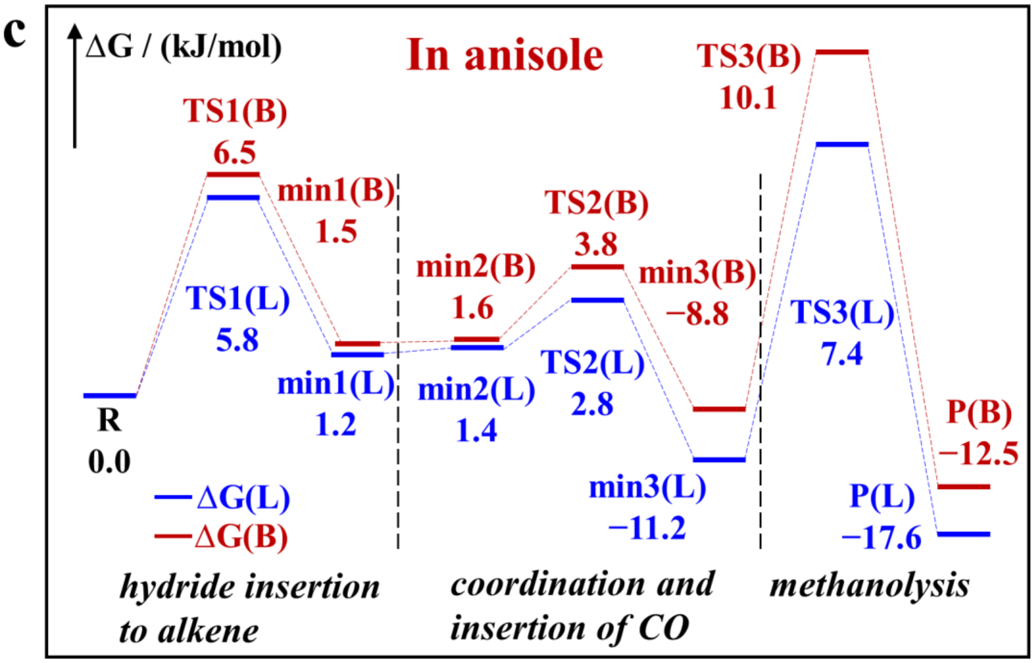
c
ΔG / (kJ/mol)
In anisole
TS1(B)
6.5
min1(B)
1.5
TS1(L)
5.8
min1(L)
1.2
R
0.0
min2(B)
1.6
min2(L)
1.4
TS2(B)
3.8
TS2(L)
2.8
min3(B)
−8.8
min3(L)
−11.2
TS3(B)
10.1
TS3(L)
7.4
P(B)
−12.5
P(L)
−17.6
ΔG(L)
ΔG(B)
hydride insertion to alkene
coordination and insertion of CO
methanolysis

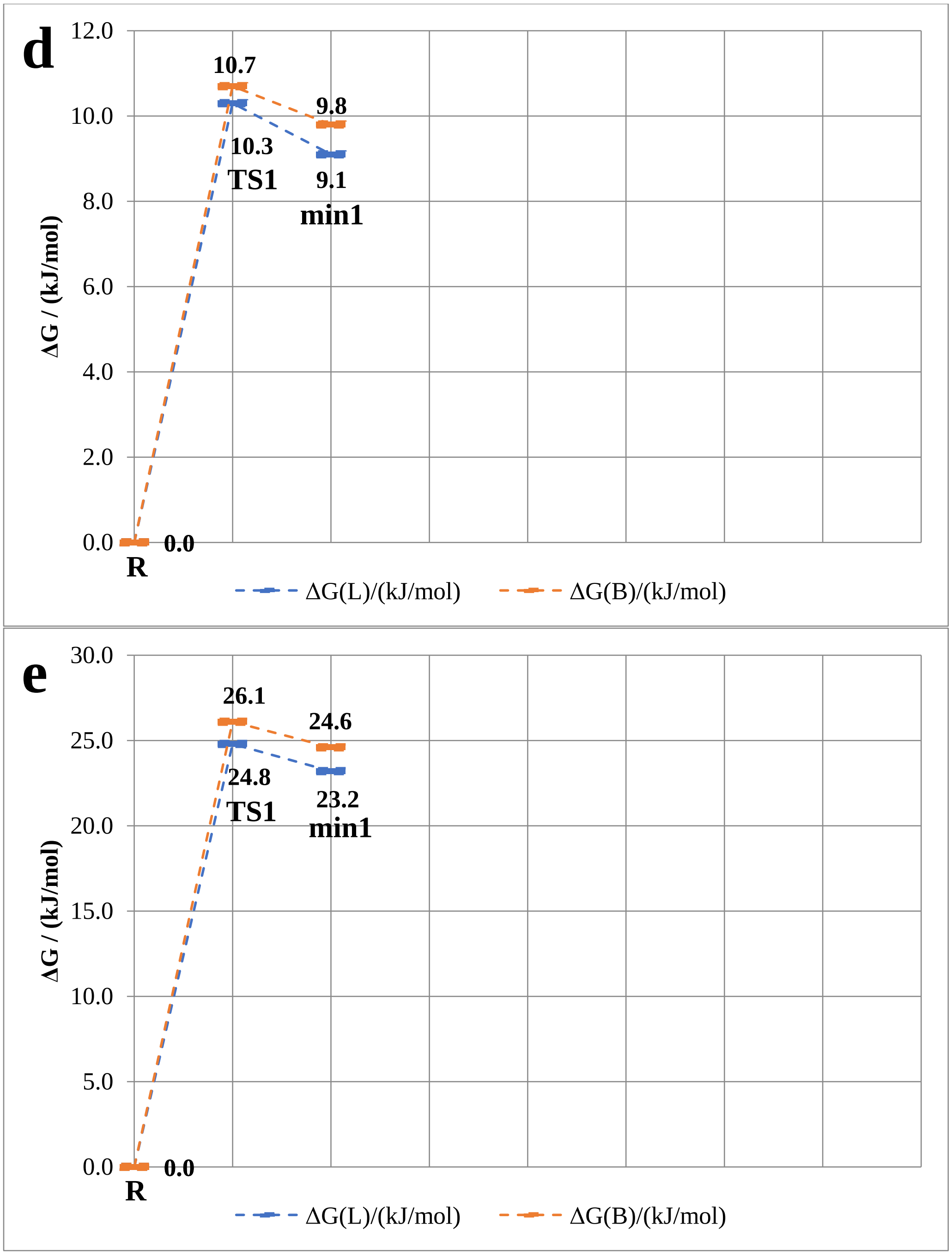

Supplementary Fig. 21 Partial reaction profiles computed for 1,4-dioxane (a), methanol (b), anisole (c), MeCN (d) and DME (e) at 373.15 K.

Supplementary Note 8: Mechanistic studies

HRMS detection for methoxycarbonylation of 1-octene using Pd(acac)$_2$/L11 as the catalyst.

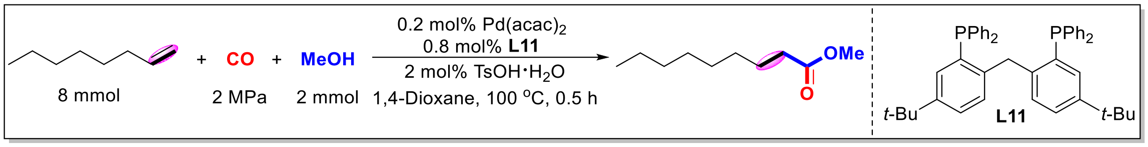

An 80 ml steel autoclave was charged with Pd(acac)$_2$ (1.2 mg, 0.2 mol%), **L11** (10.4 mg, 0.8 mol%), PTSA·H_2O (7.6 mg, 2 mol%), and 1,4-Dioxane (4 ml). 1-octene (8.0 mmol) and MeOH (2.0 mmol) were introduced into the autoclave. After the autoclave was purged with CO (1 MPa) for three times at room temperature and then pressurized with CO to 2 MPa, the autoclave was sealed and put into a preheated reactor, stirring at 100 °C for 0.5 h. Afterwards, the autoclave was cooled to room temperature and depressurized slowly. Subsequently, the reaction mixture was analyzed by gas chromatography (Agilent 7890A GC equipped with a HP-5 capillary column with 5 wt.% phenyl groups and the FID detector). 12% yield and 91% linear selectivity was obtained by GC analysis using *n*-decane as the internal standard. Meanwhile, the crude solution after reaction was subjected to run HRMS analysis.

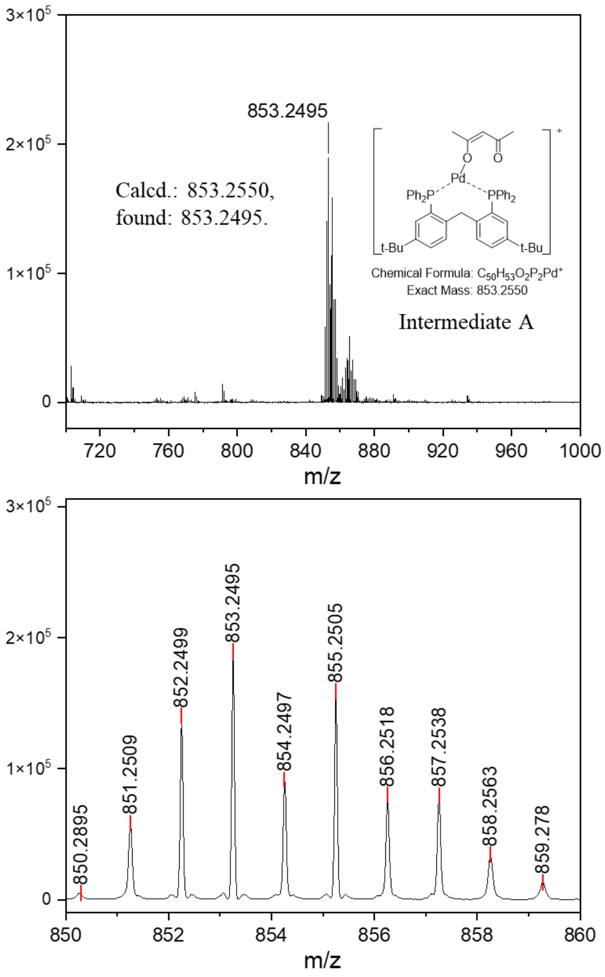

Supplementary Fig. 22 HRMS detection of the reaction mixture after reaction.

^{31}P NMR characterization for methoxycarbonylation of 1-octene using Pd(acac)$_2$/L11 as the catalyst.

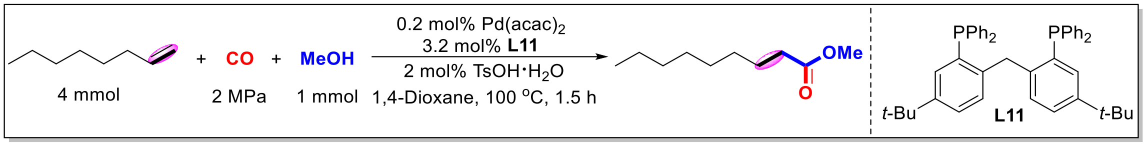

A 36 ml steel autoclave was charged with Pd(acac)$_2$ (0.6 mg, 0.2 mol%), **L11** (20.8 mg, 3.2 mol%), PTSA·H_2O (3.8 mg, 2 mol%), and 1,4-Dioxane (2 ml). 1-octene (4.0 mmol) and MeOH (1.0 mmol) were introduced into the autoclave. After the autoclave was purged with CO (1 MPa) for three times at room temperature and then pressurized with CO to 2 MPa, the autoclave was sealed and put into a preheated reactor, stirring at 100 °C for 1.5 h. Afterwards, the autoclave was cooled to room temperature and depressurized slowly. Subsequently, the reaction mixture was analyzed by gas chromatography (Agilent 7890A GC equipped with a HP-5 capillary column with 5 wt.% phenyl groups and the FID detector). 51% yield and 91% linear selectivity was obtained by GC analysis using *n*-decane as the internal standard. Finally, the crude solution before and after reaction were subjected to run ^{31}P NMR analysis.

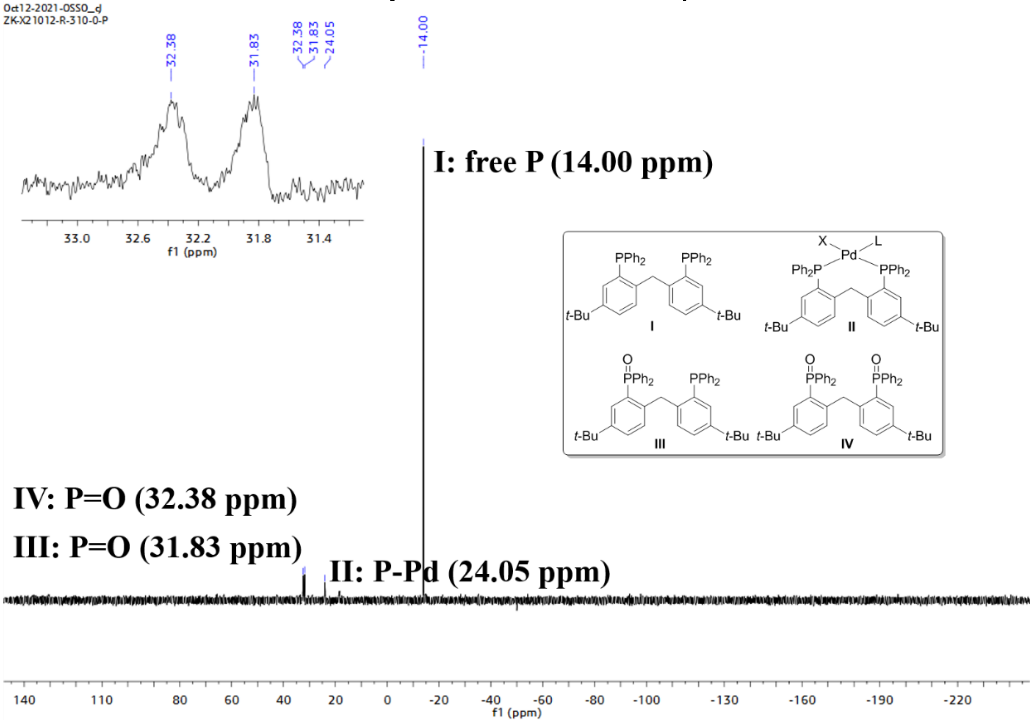

Supplementary Fig. 23 ^{31}P NMR characterization of the reaction mixture before reaction.

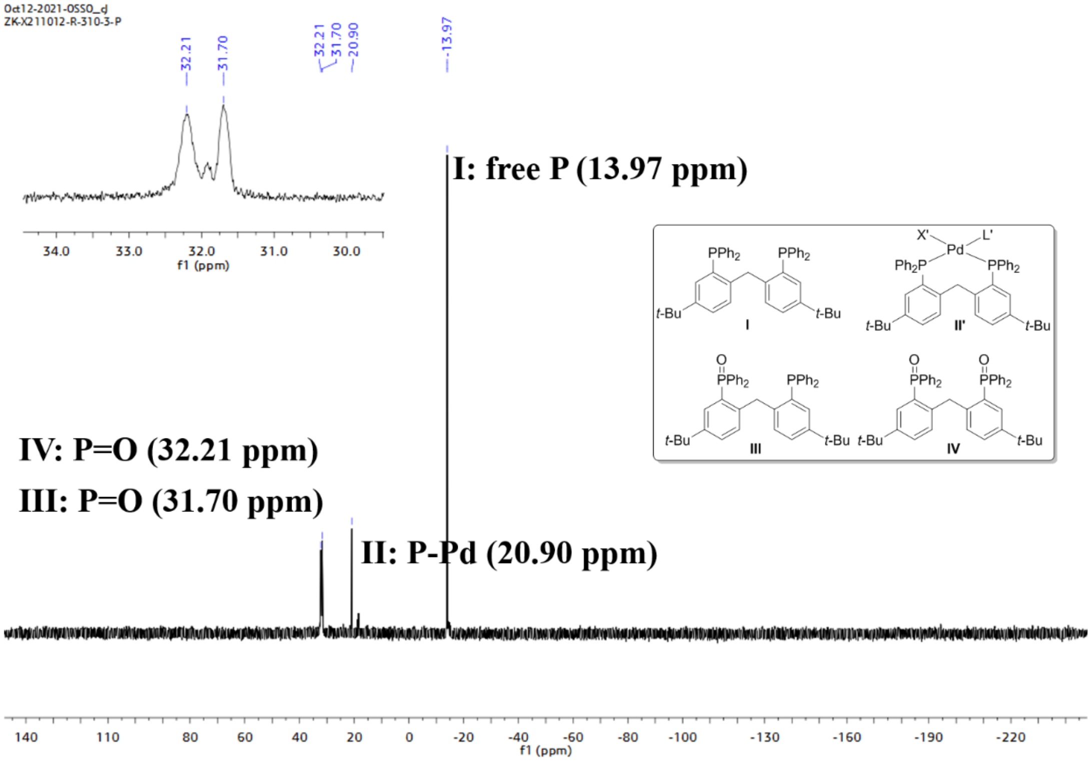

Supplementary Fig. 24 ^{31}P NMR characterization of the reaction mixture after reaction.

In situ high-pressure FTIR characterization of 1-octene methoxycarbonylation

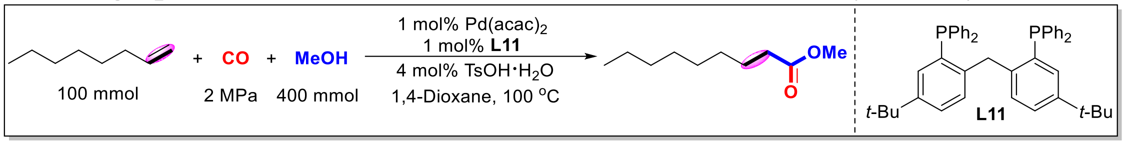

A 300 ml steel autoclave was charged with $Pd(acac)_2$ (304 mg, 1 mol%), **L11** (648 mg, 1 mol%), PTSA·H_2O (760 mg, 4 mol%), and 1,4-Dioxane (100 ml). 1-octene (11.2 g, 100 mmol) and MeOH (12.8 g, 400 mmol) were introduced into the autoclave. After the autoclave was purged with CO (1 MPa) for four times at room temperature and then pressurized with CO to 2 MPa, the autoclave was linked with in-situ IR spectroscopy system. Finally, real-time monitoring of this catalytic system was performed at different reaction temperatures and times.

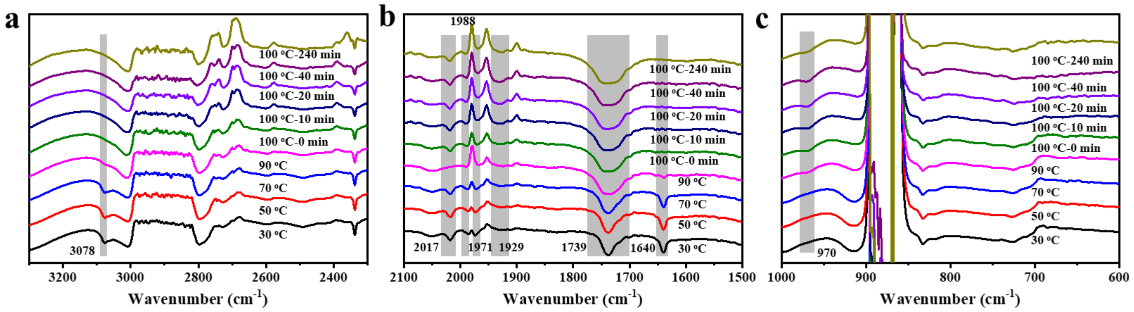

Supplementary Fig. 25 In situ high-pressure FTIR characterization of 1-octene methoxycarbonylation. a, 3300-2400 cm^{-1}. b, 2100-1500 cm^{-1}. c, 1000-600 cm^{-1}.

Supplementary Note 9: Copies of GC and NMR spectra

NMR spectra of the ligands

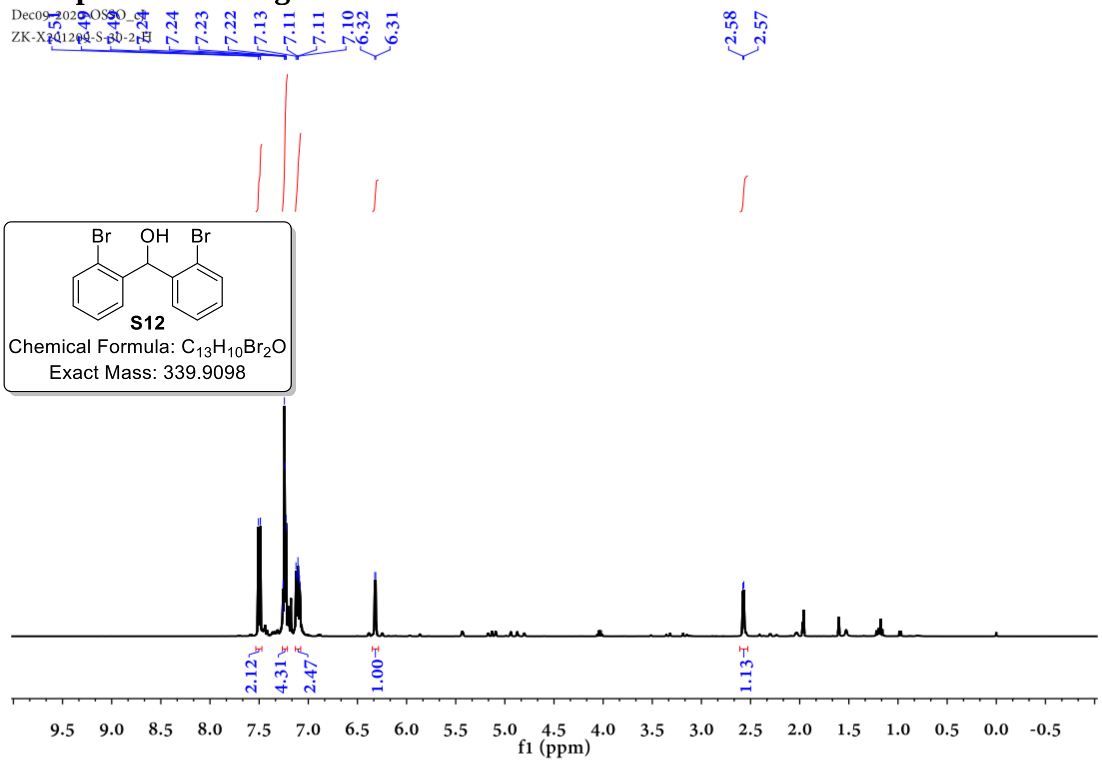

Supplementary Fig. 26 ^{1}H NMR (400 MHz, 20 ºC) spectrum of S12 in $CDCl_3$.

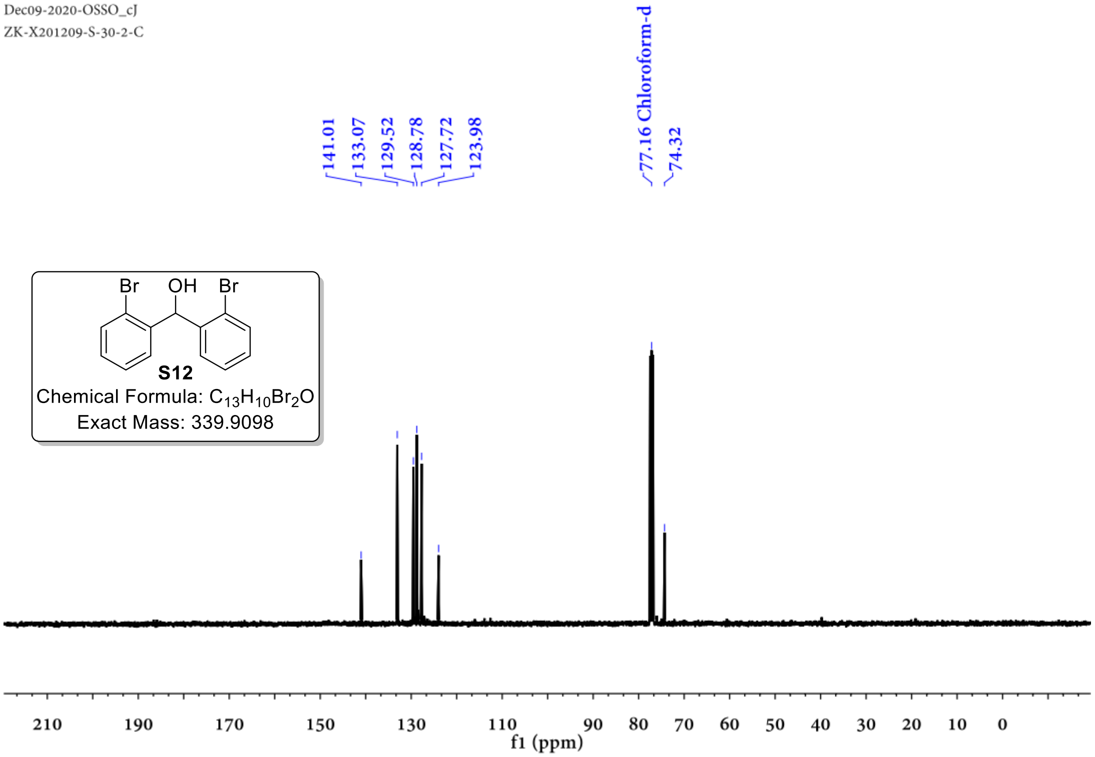

Supplementary Fig. 27 ^{13}C NMR (101 MHz, 20 ºC) spectrum of S12 in $CDCl_3$.

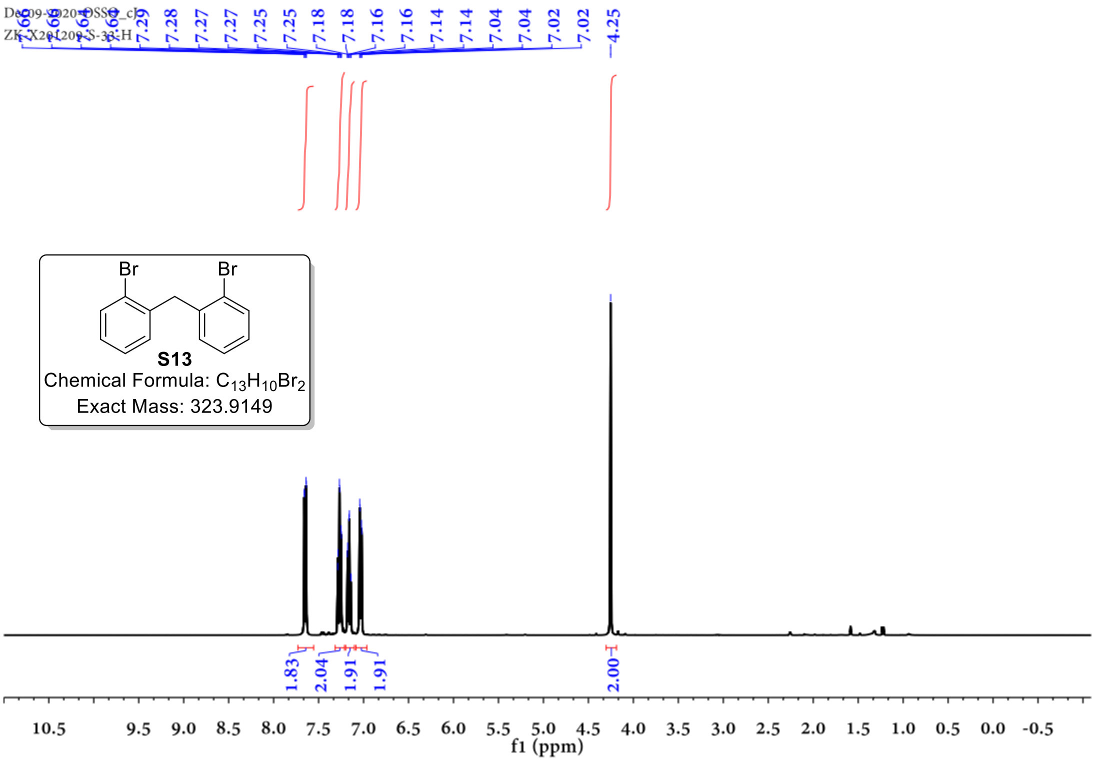

Supplementary Fig. 28 ^{1}H NMR (400 MHz, 20 °C) spectrum of S13 in $CDCl_3$.

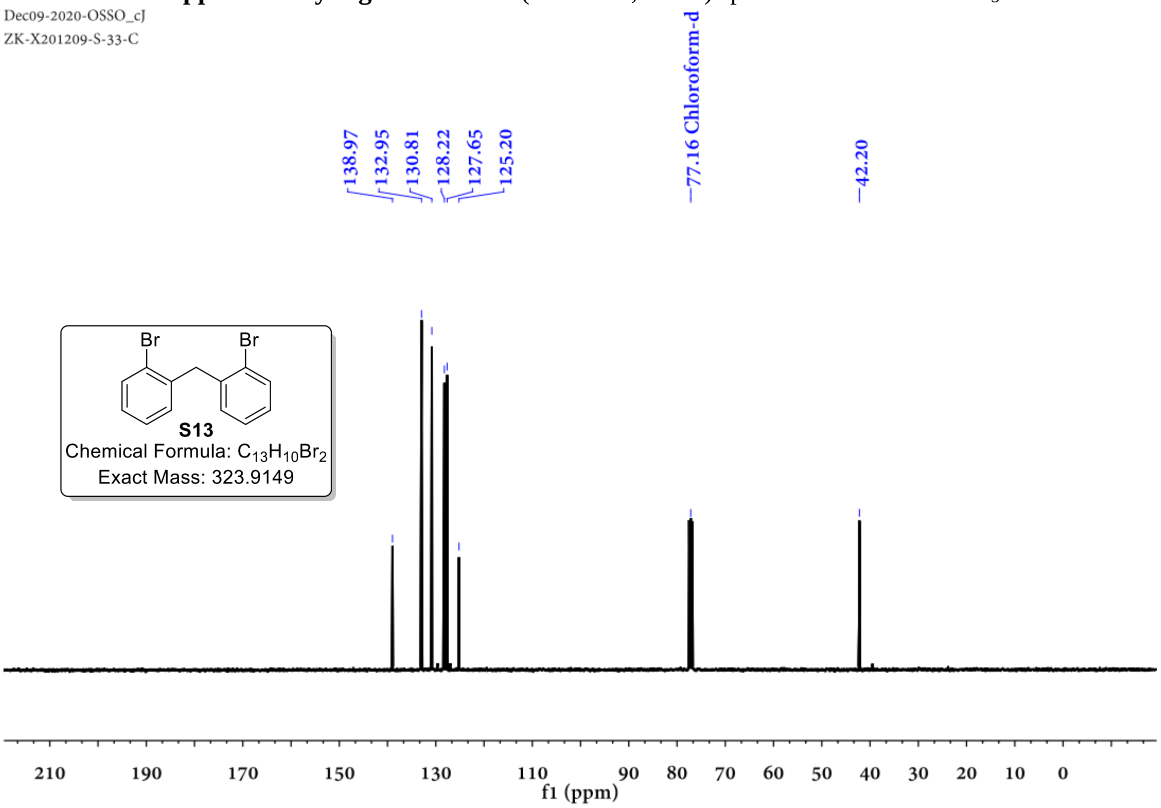

Supplementary Fig. 29 ^{13}C NMR (101 MHz, 20 °C) spectrum of S13 in $CDCl_3$.

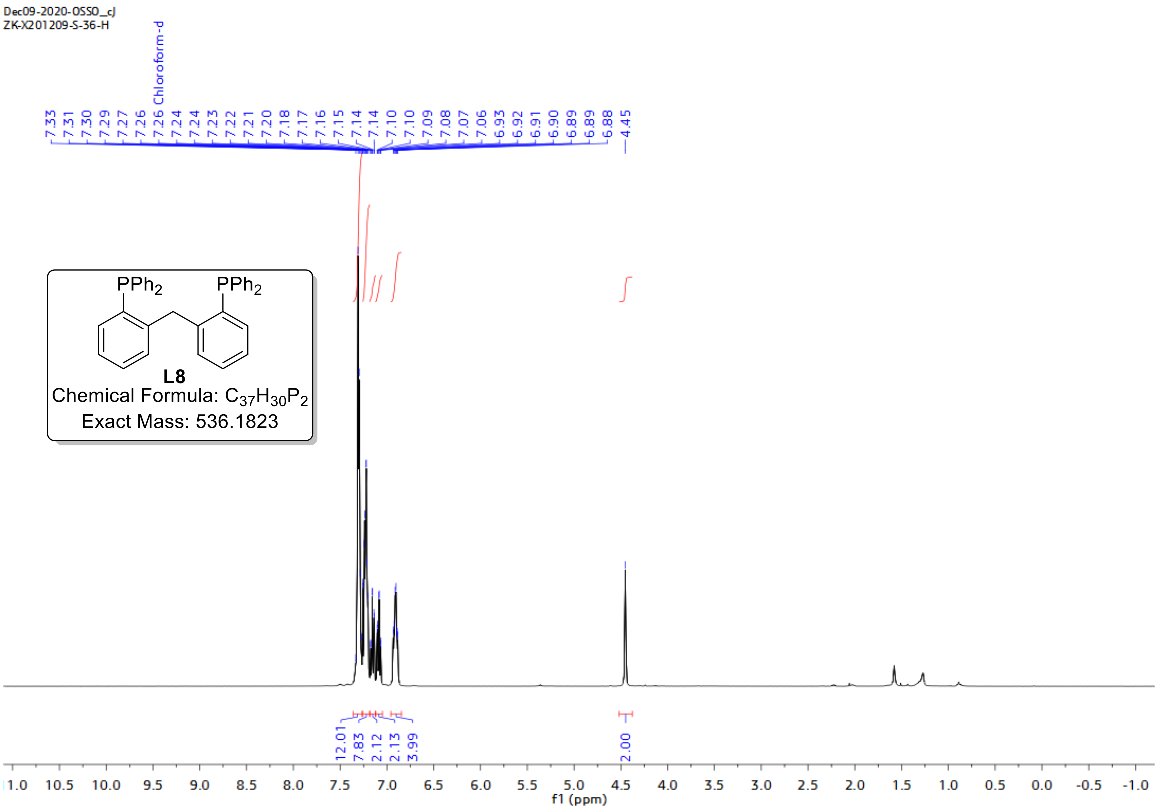

Supplementary Fig. 30 ^{1}H NMR (400 MHz, 20 °C) spectrum of L8 in $CDCl_3$.

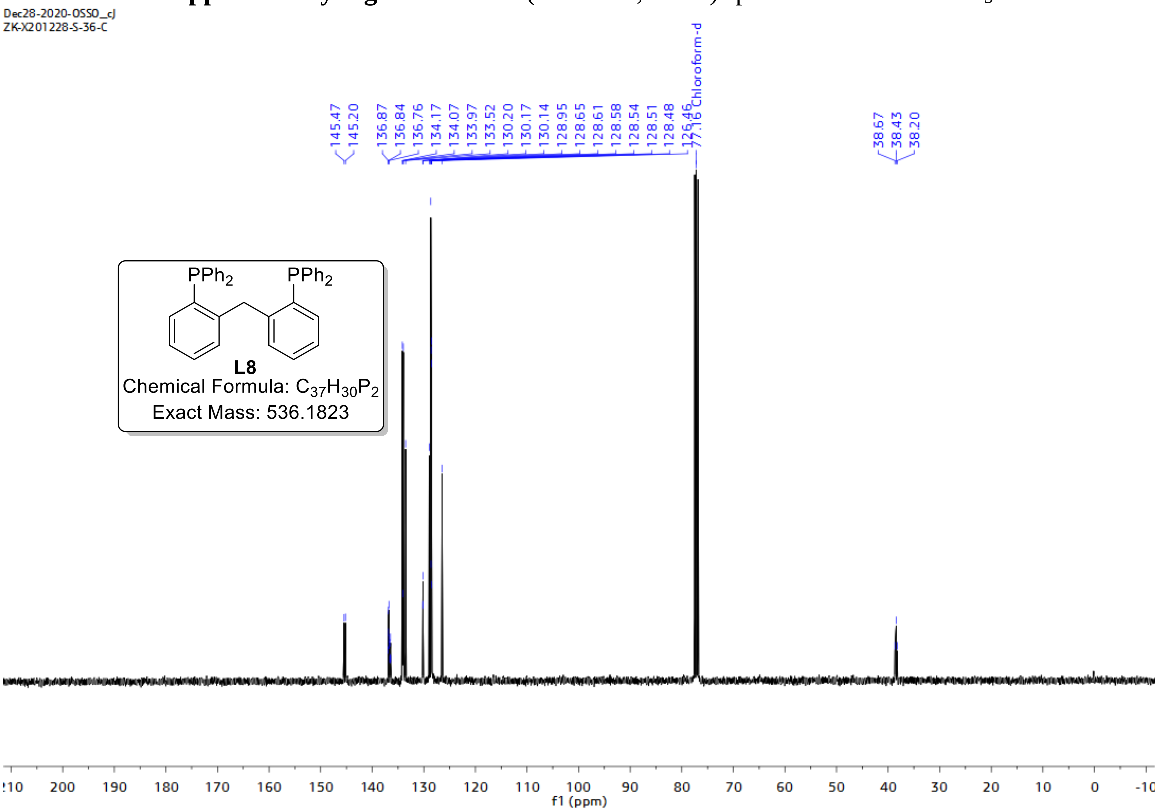

Supplementary Fig. 31 ^{13}C NMR (101 MHz, 20 °C) spectrum of L8 in $CDCl_3$.

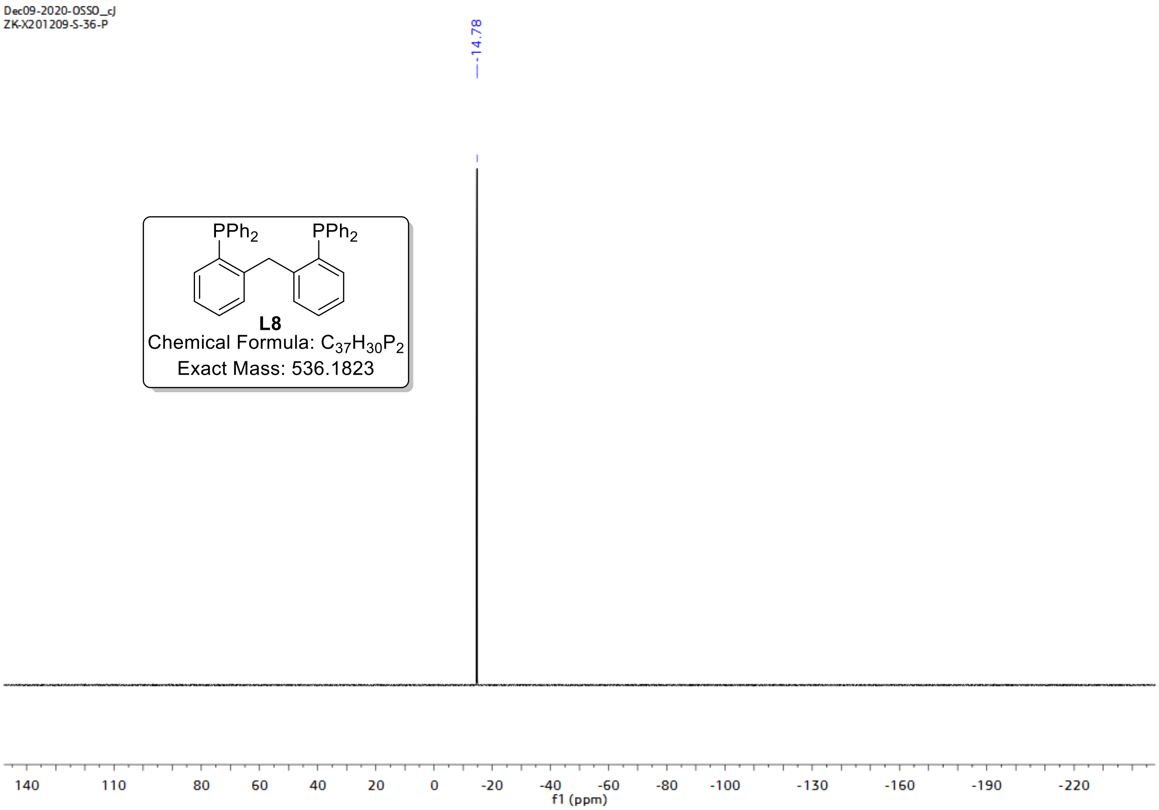

Supplementary Fig. 32 ^{31}P NMR (162 MHz, 20 ºC) spectrum of L8 in $CDCl_3$.

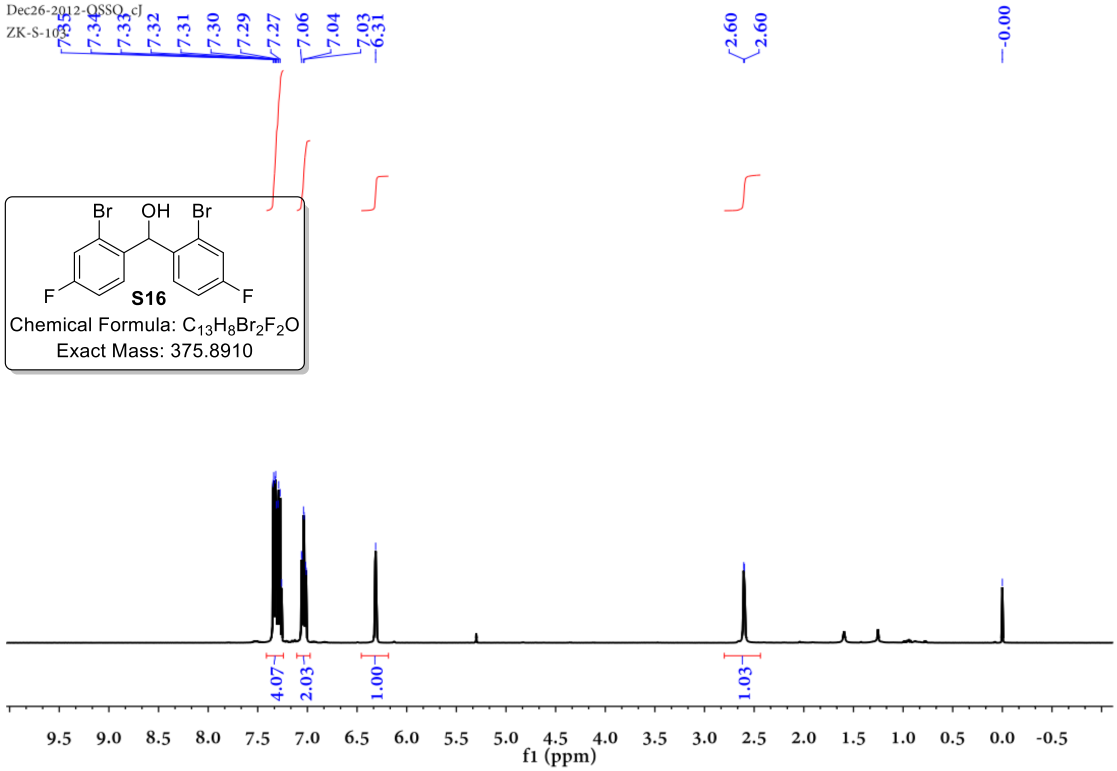

Supplementary Fig. 33 ^{1}H NMR (400 MHz, 20 ºC) spectrum of S16 in $CDCl_3$.

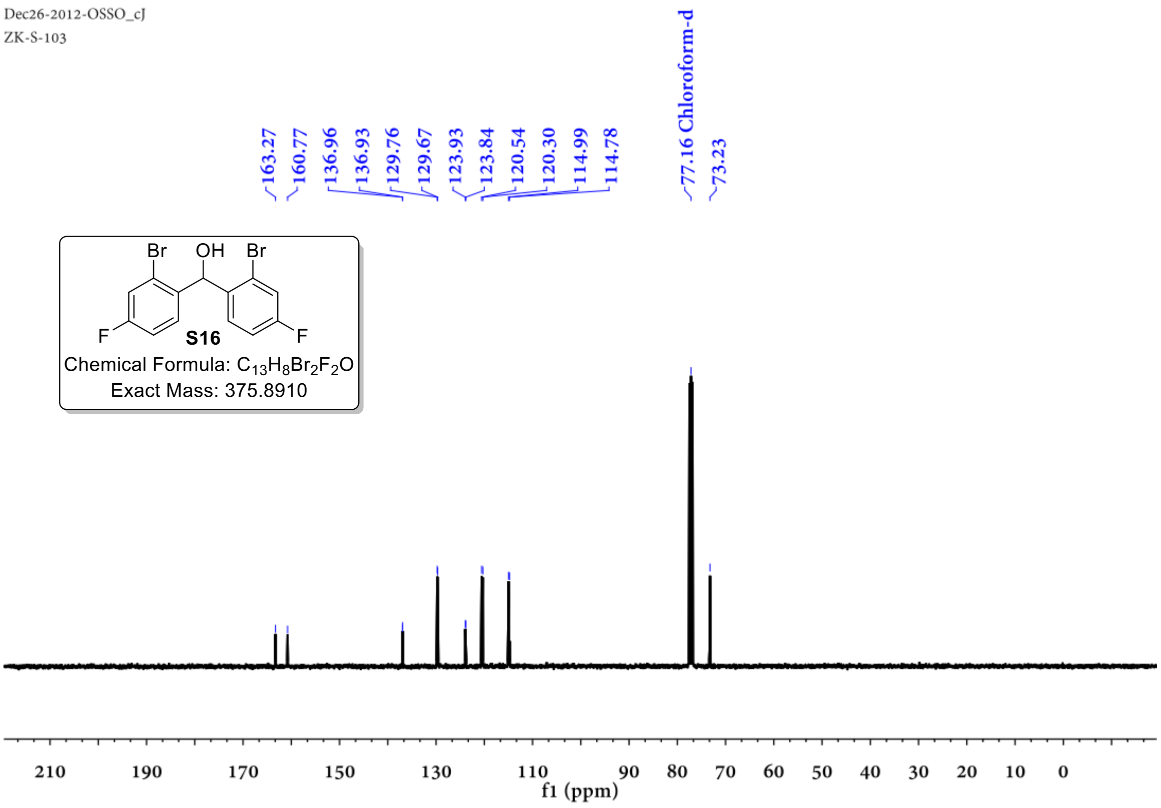

Supplementary Fig. 34 ^{13}C NMR (101 MHz, 20 °C) spectrum of S16 in $CDCl_3$.

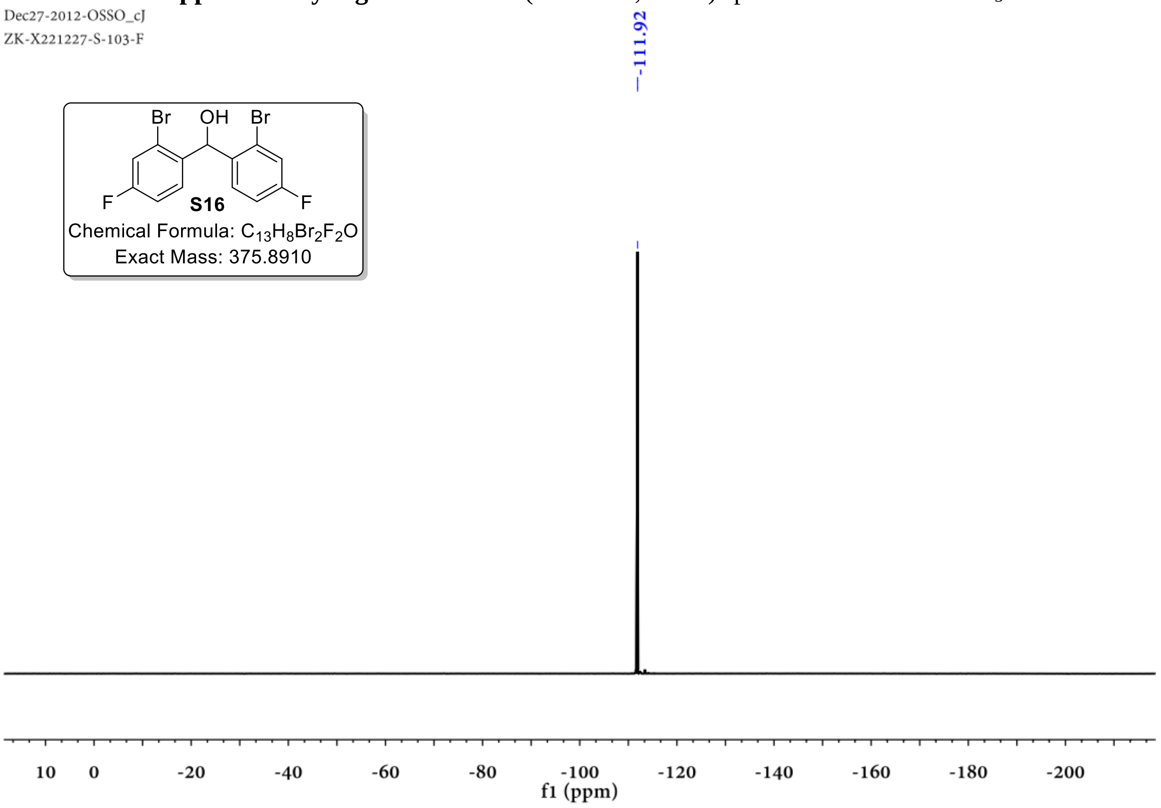

Supplementary Fig. 35 ^{19}F NMR (376 MHz, 20 °C) spectrum of S16 in $CDCl_3$.

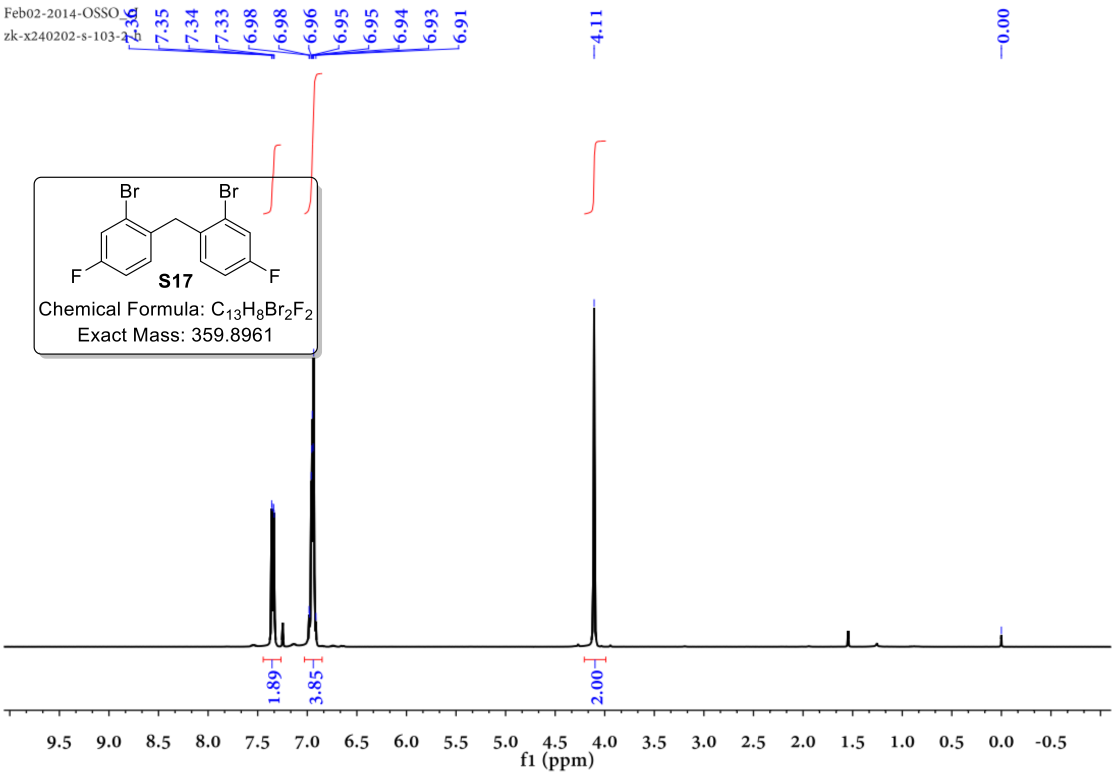

Supplementary Fig. 36 ^{1}H NMR (400 MHz, 20 °C) spectrum of S17 in $CDCl_3$.

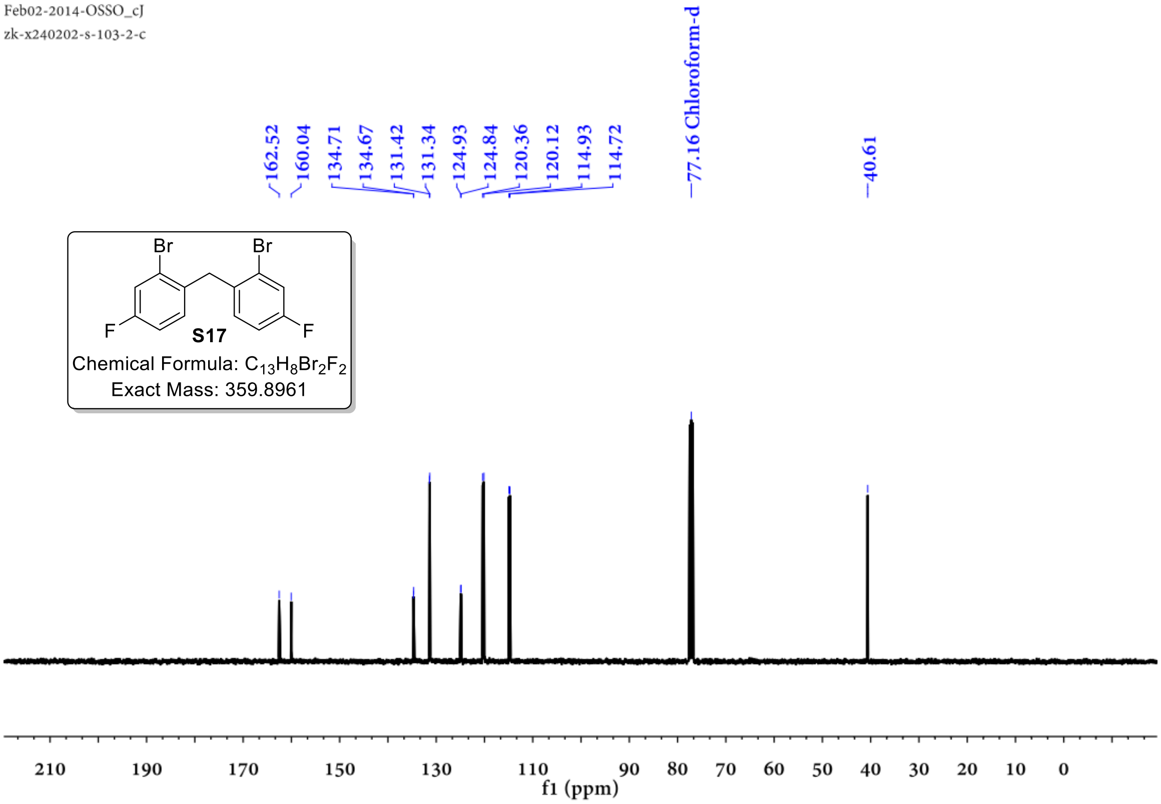

Supplementary Fig. 37 ^{13}C NMR (101 MHz, 20 °C) spectrum of S17 in $CDCl_3$.

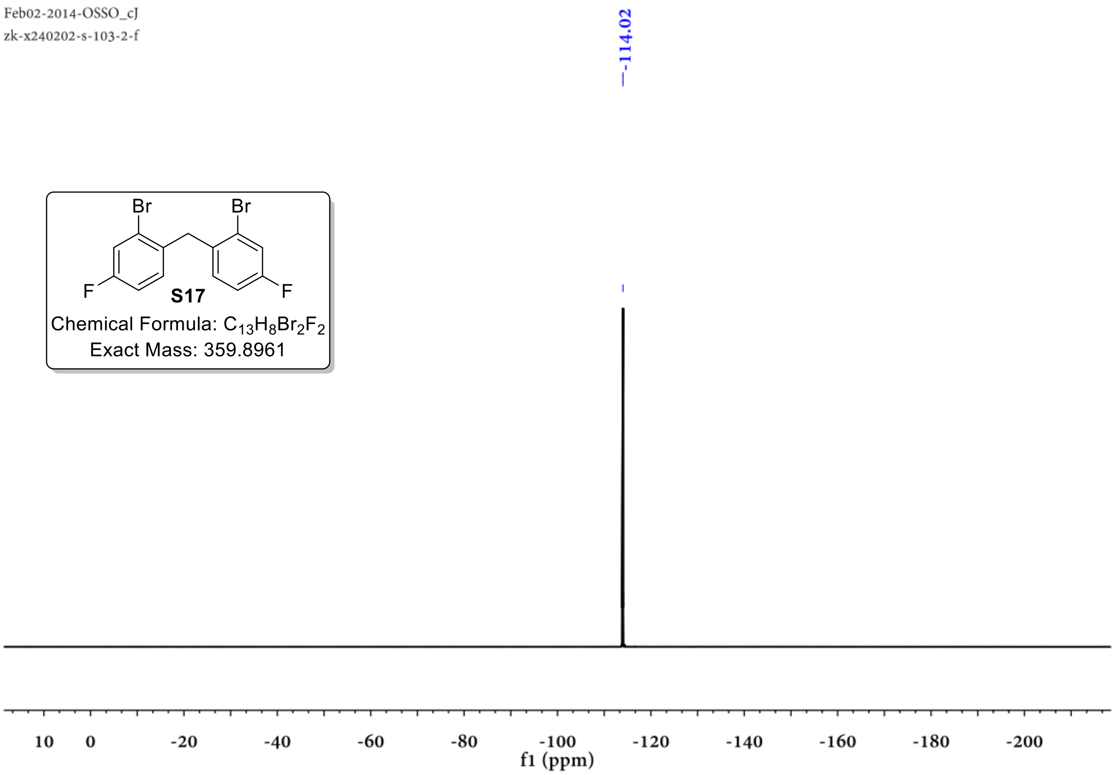

Supplementary Fig. 38 ^{19}F NMR (376 MHz, 20 ºC) spectrum of S17 in $CDCl_3$.

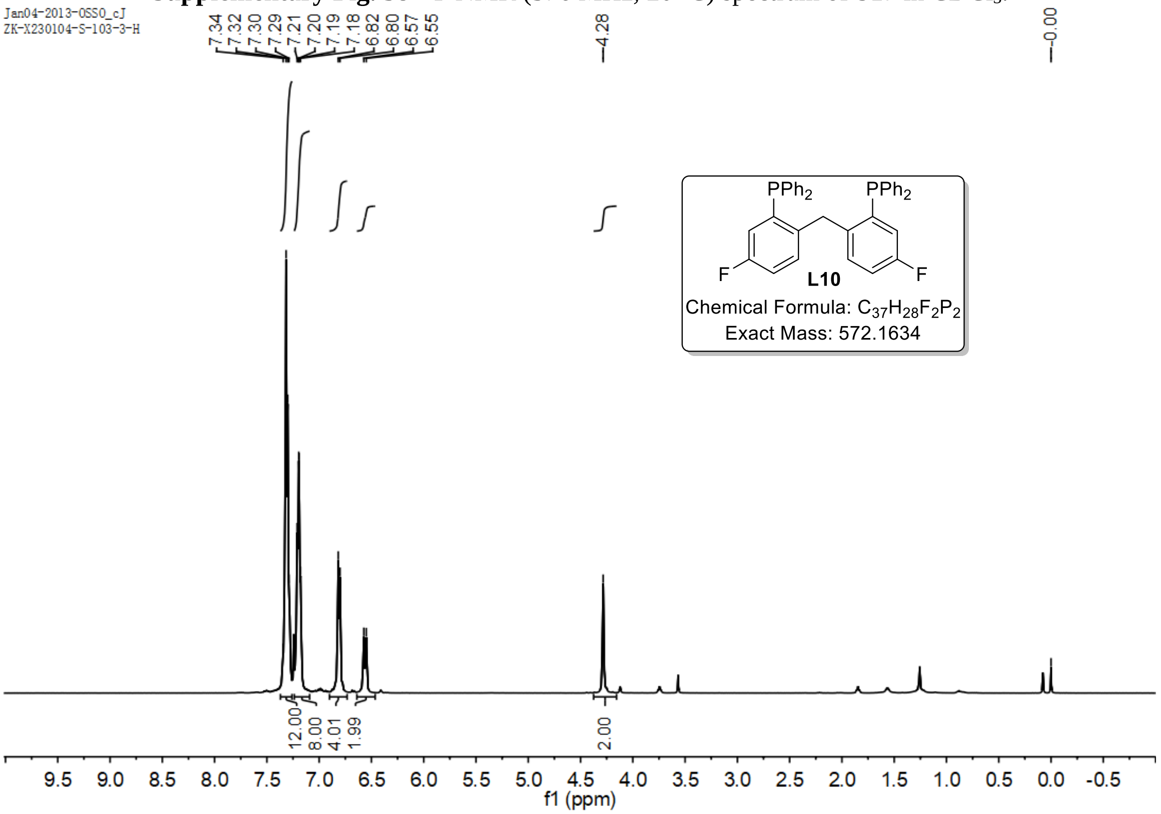

Supplementary Fig. 39 ^{11}H NMR (400 MHz, 20 ºC) spectrum of L10 in $CDCl_3$.

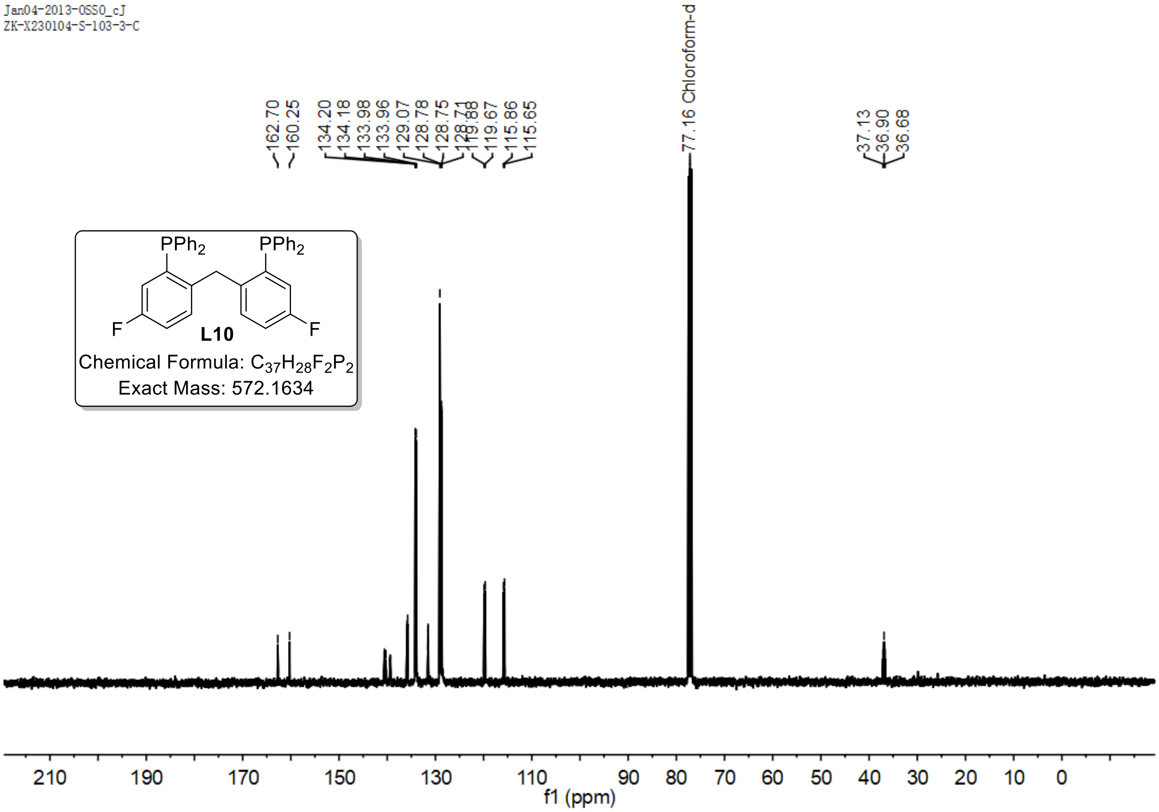

Supplementary Fig. 40 ^{13}C NMR (101 MHz, 20 ºC) spectrum of L10 in $CDCl_3$.

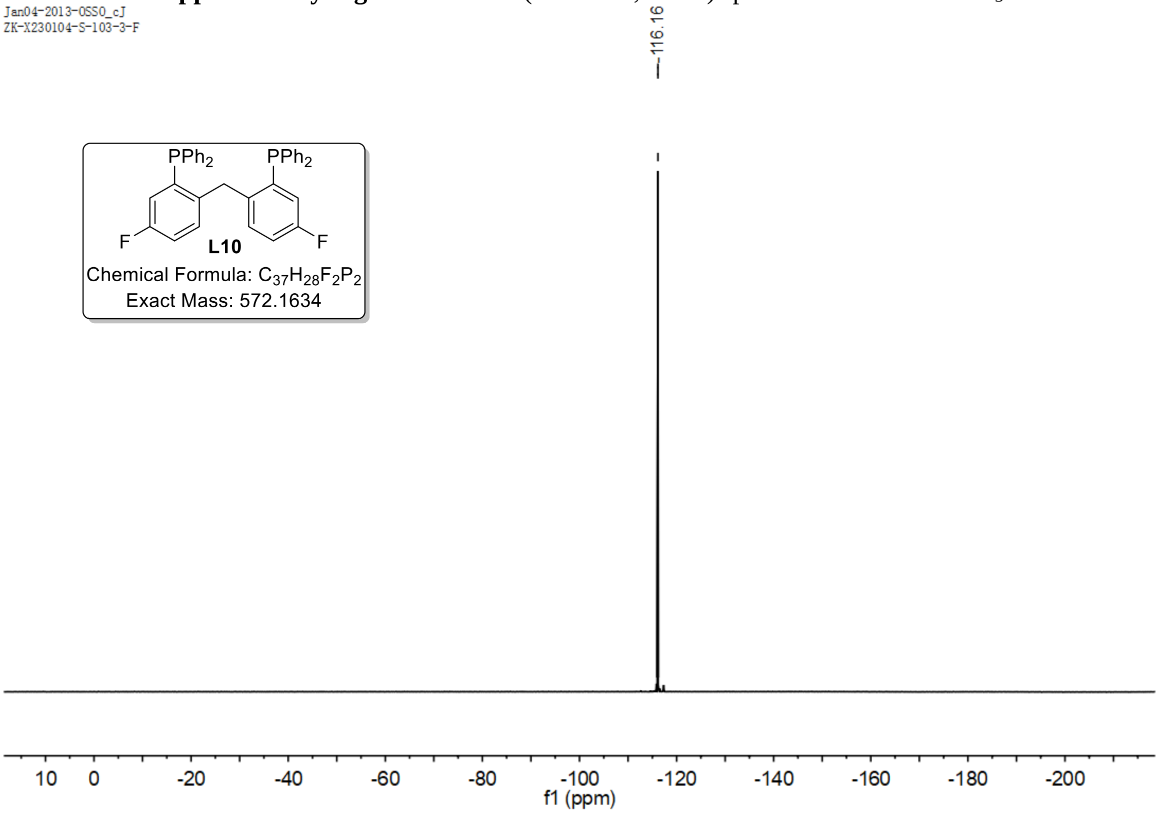

Supplementary Fig. 41 ^{19}F NMR (376 MHz, 20 ºC) spectrum of L10 in $CDCl_3$.

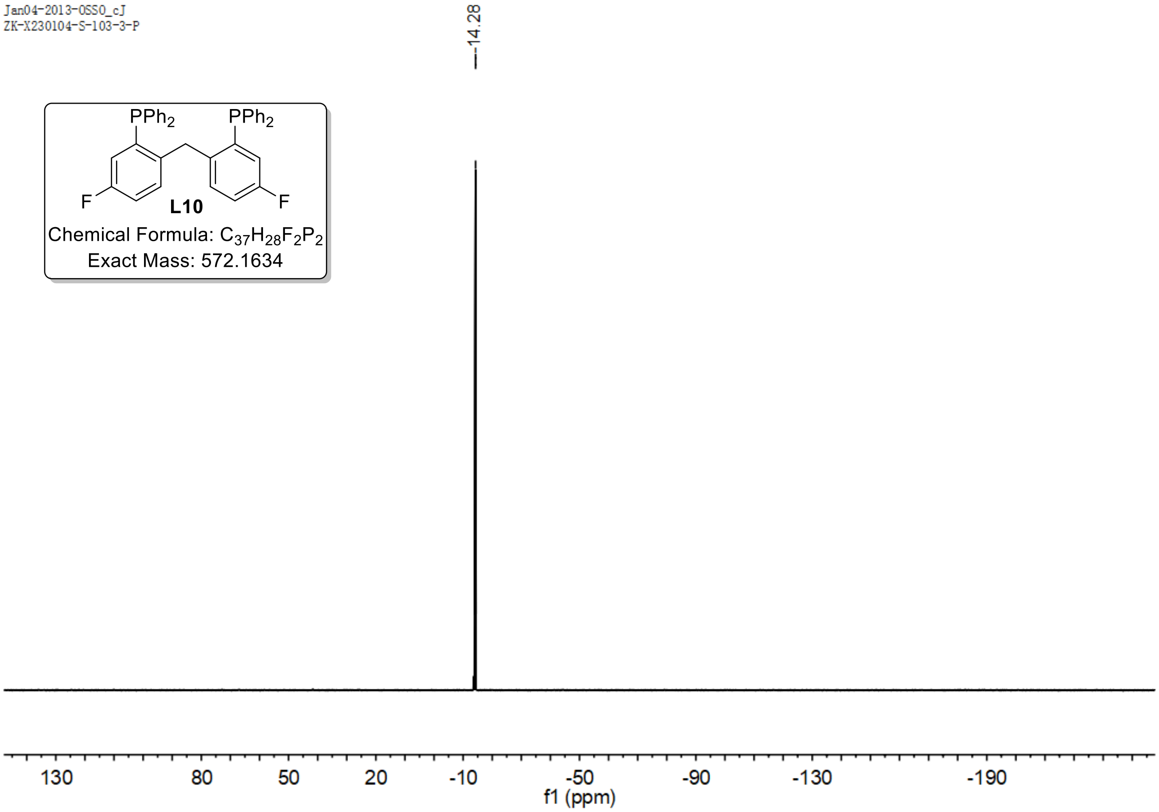

Supplementary Fig. 42 ^{31}P NMR (162 MHz, 20 °C) spectrum of L10 in $CDCl_3$.

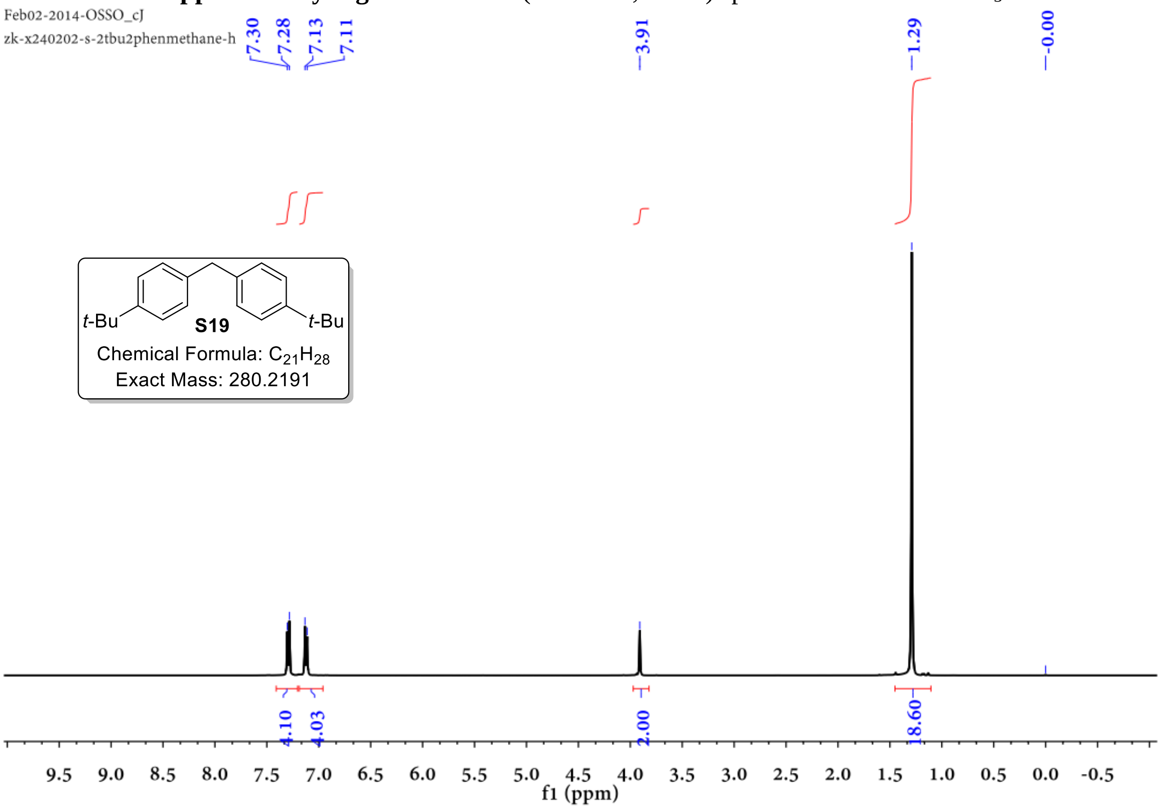

Supplementary Fig. 43 ^{1}H NMR (400 MHz, 20 °C) spectrum of S19 in $CDCl_3$.

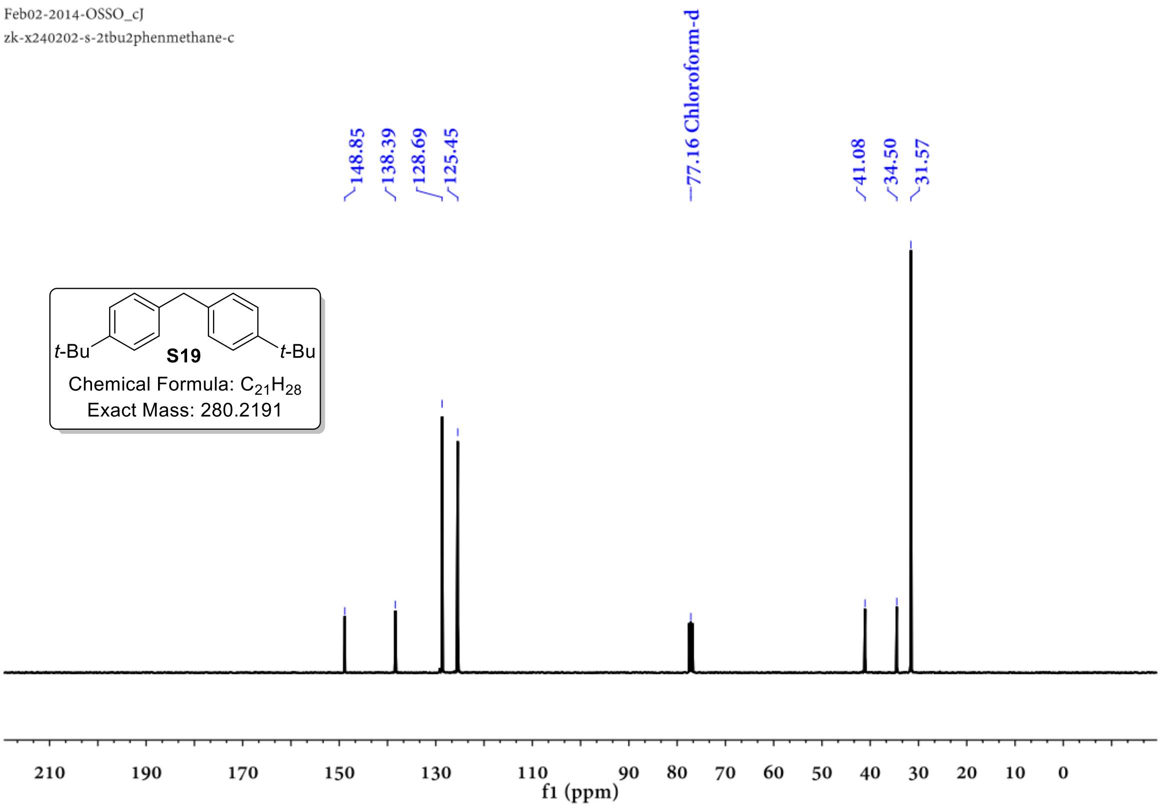

Supplementary Fig. 44 ^{13}C NMR (101 MHz, 20 °C) spectrum of S19 in $CDCl_3$.

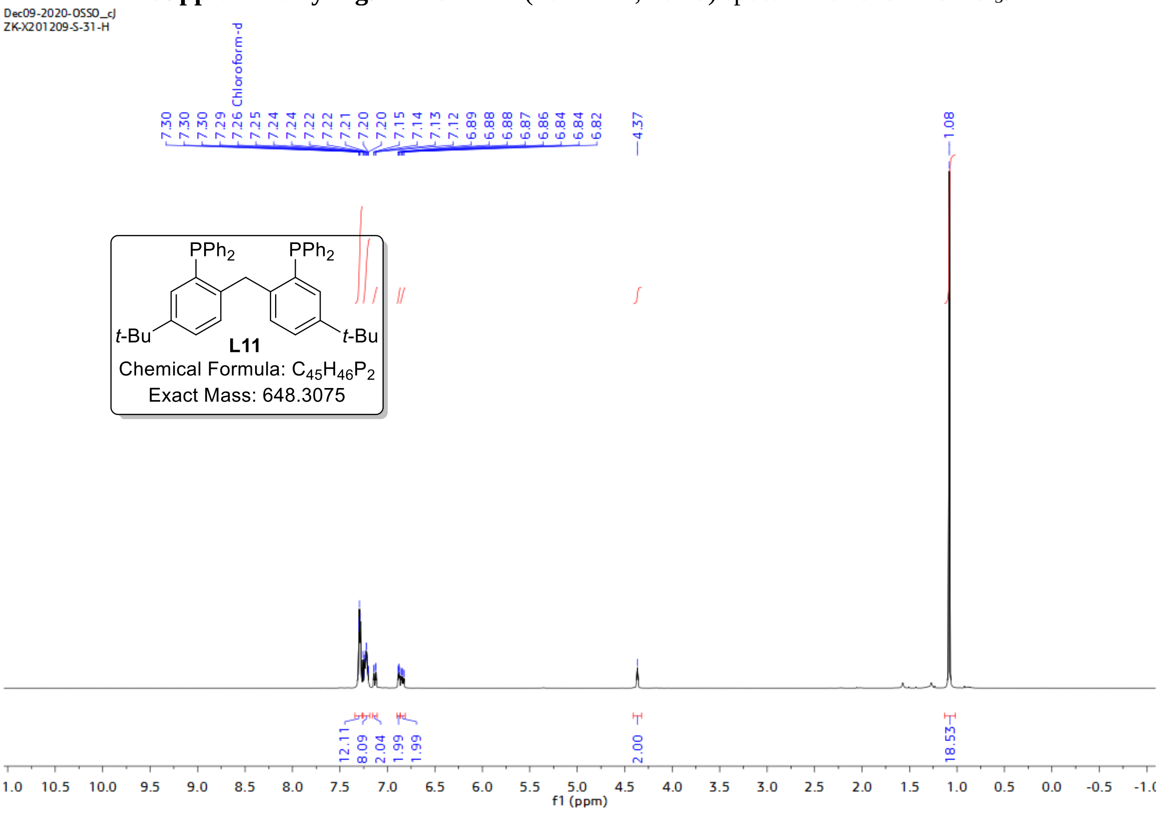

Supplementary Fig. 45 ^{1}H NMR (400 MHz, 20 °C) spectrum of L11 in $CDCl_3$.

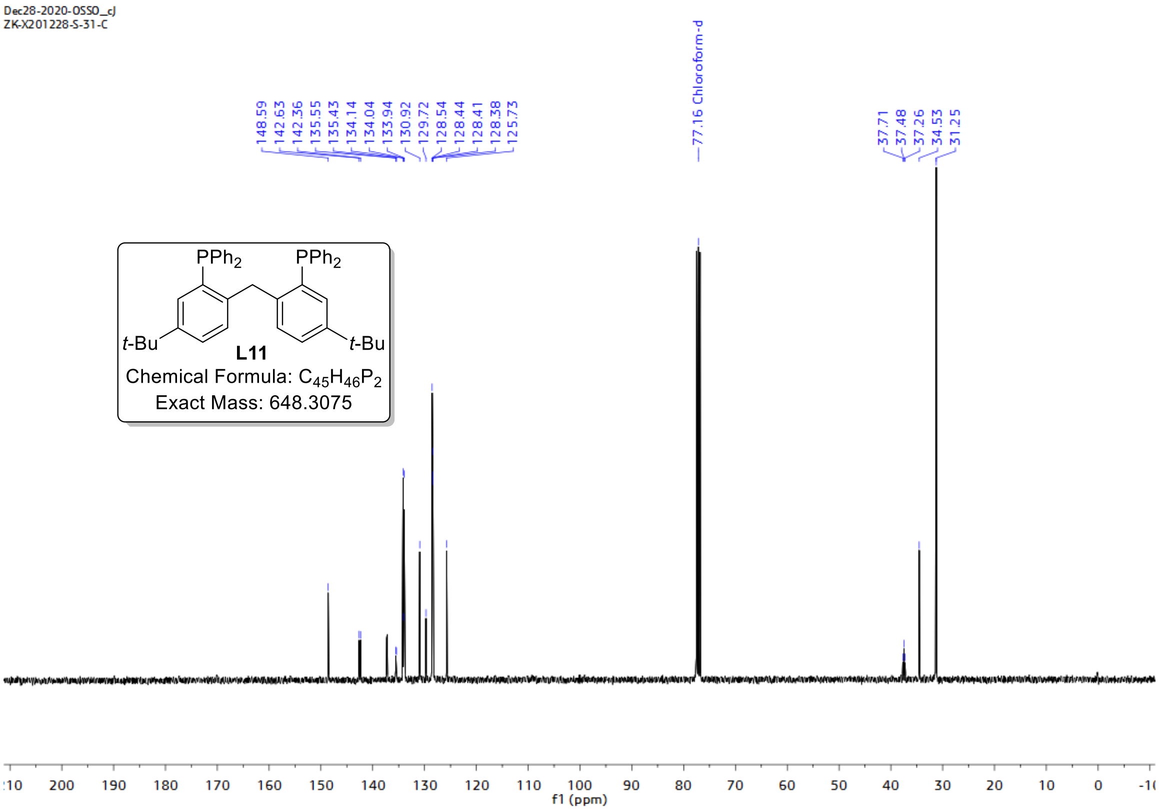

Supplementary Fig. 46 ^{13}C NMR (101 MHz, 20 ºC) spectrum of L11 in $CDCl_3$.

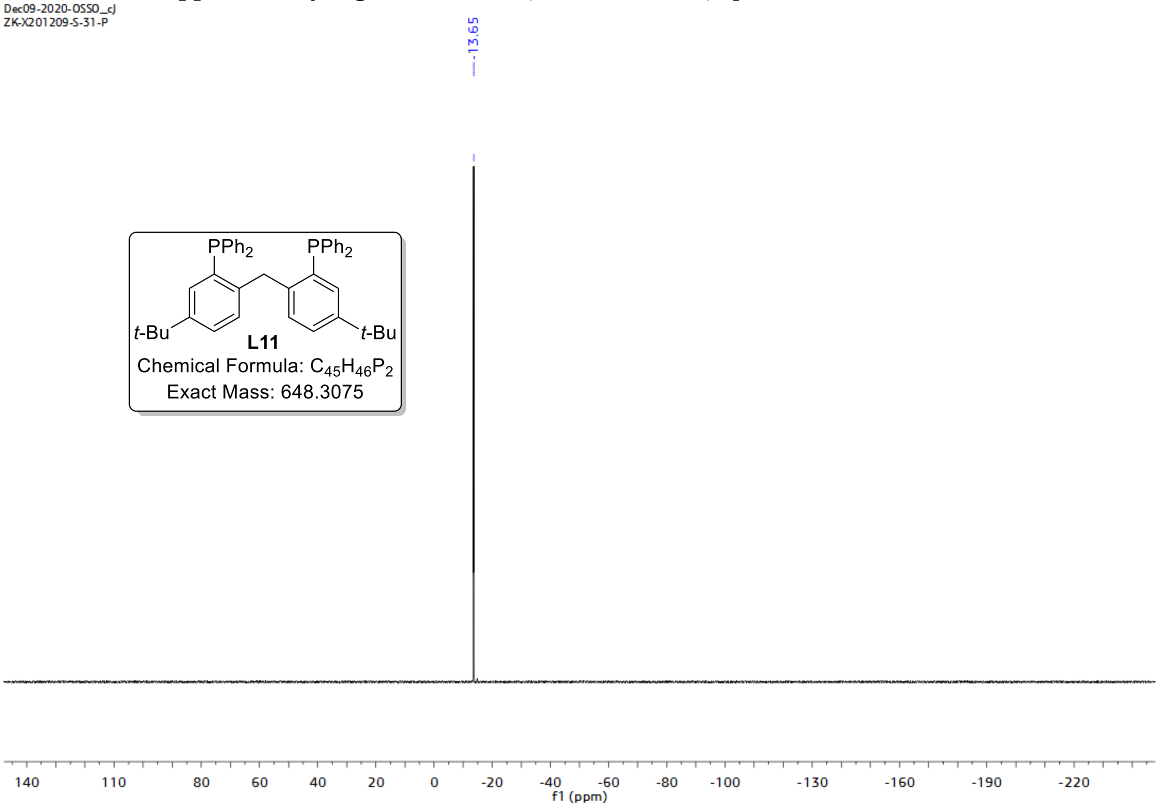

Supplementary Fig. 47 ^{31}P NMR (162 MHz, 20 ºC) spectrum of L11 in $CDCl_3$.

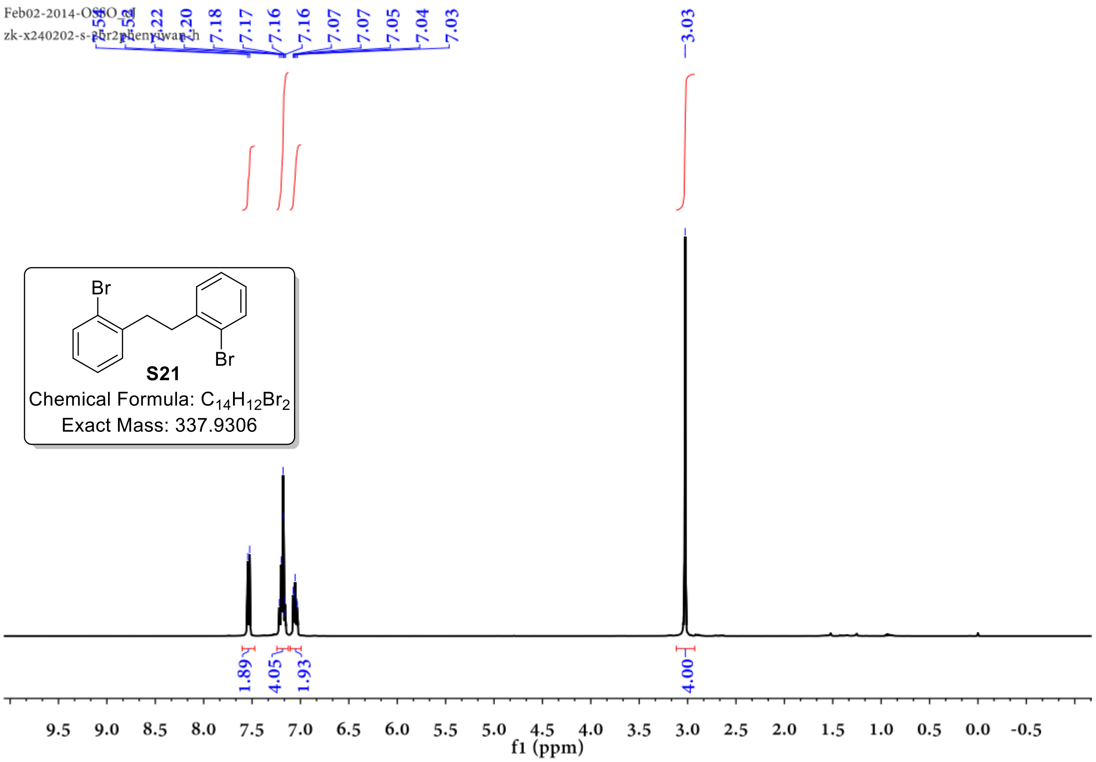

Supplementary Fig. 48 ^{1}H NMR (400 MHz, 20 °C) spectrum of S21 in $CDCl_3$.

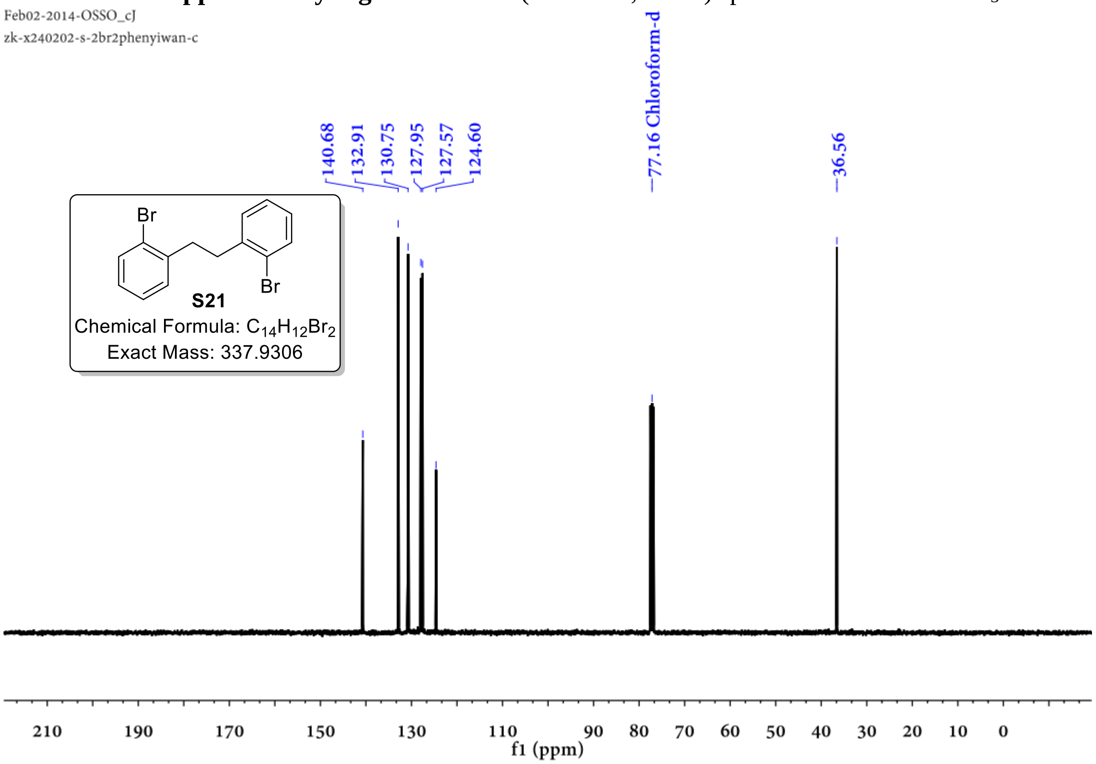

Supplementary Fig. 49 ^{13}C NMR (101 MHz, 20 °C) spectrum of S21 in $CDCl_3$.

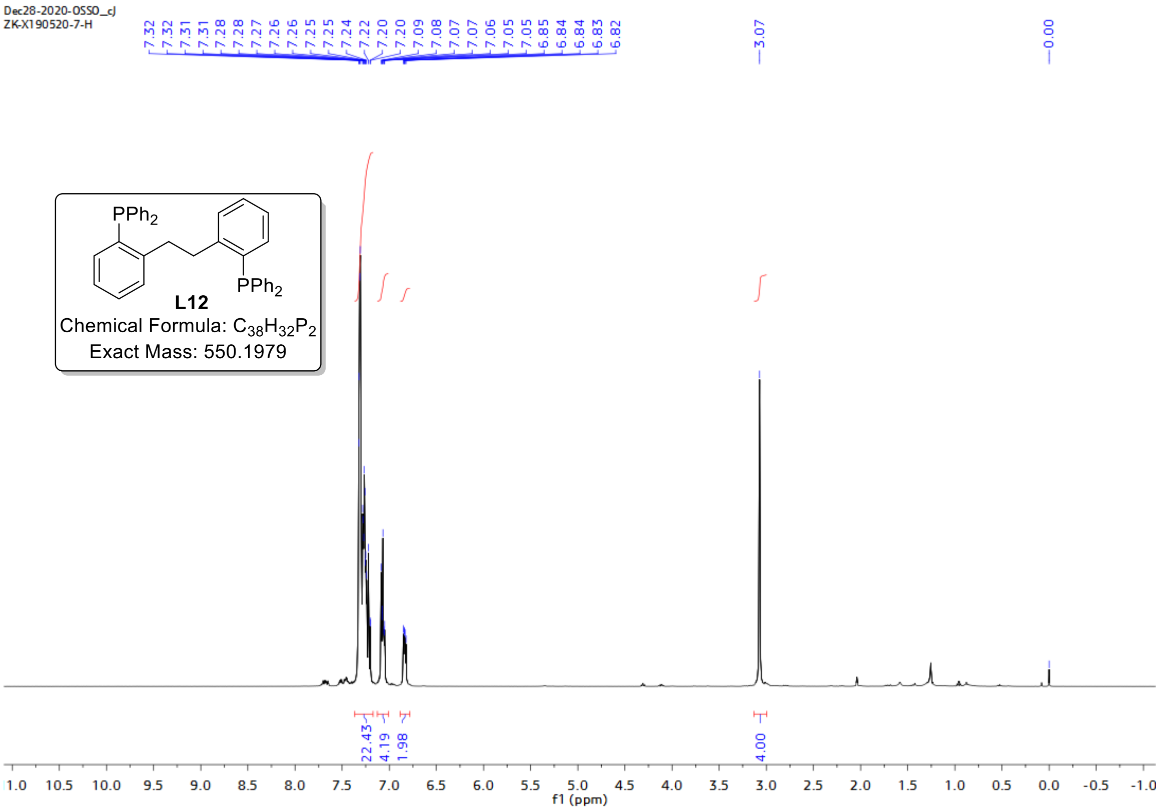

Supplementary Fig. 50 ^{1}H NMR (400 MHz, 20 ºC) spectrum of L12 in $CDCl_3$.

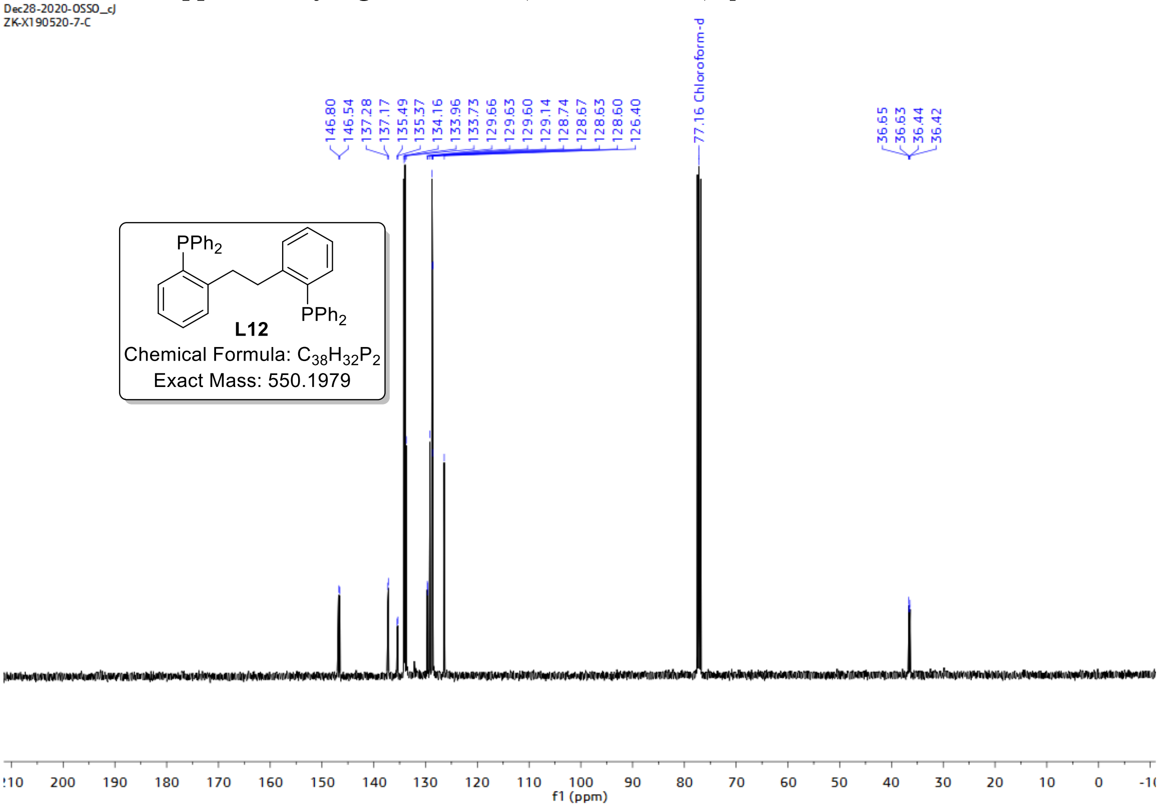

Supplementary Fig. 51 ^{13}C NMR (101 MHz, 20 ºC) spectrum of L12 in $CDCl_3$.

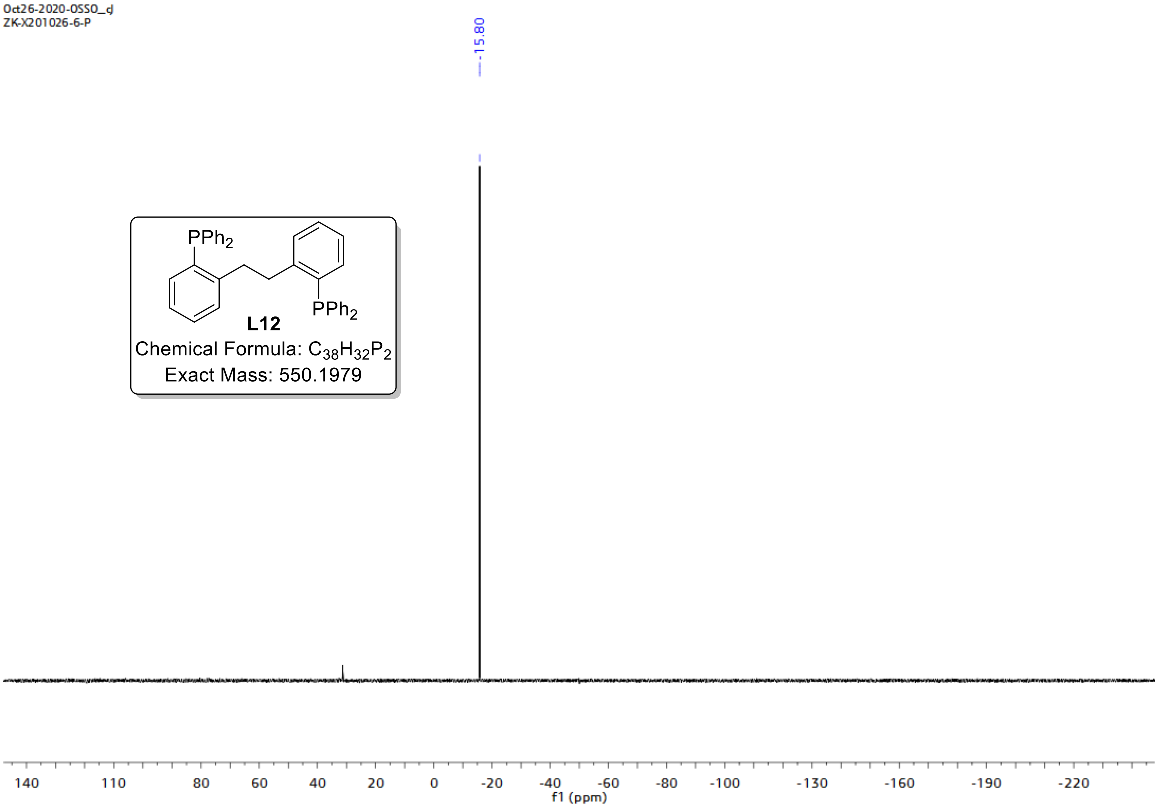

Supplementary Fig. 52 ^{31}P NMR (162 MHz, 20 ºC) spectrum of L12 in $CDCl_3$.

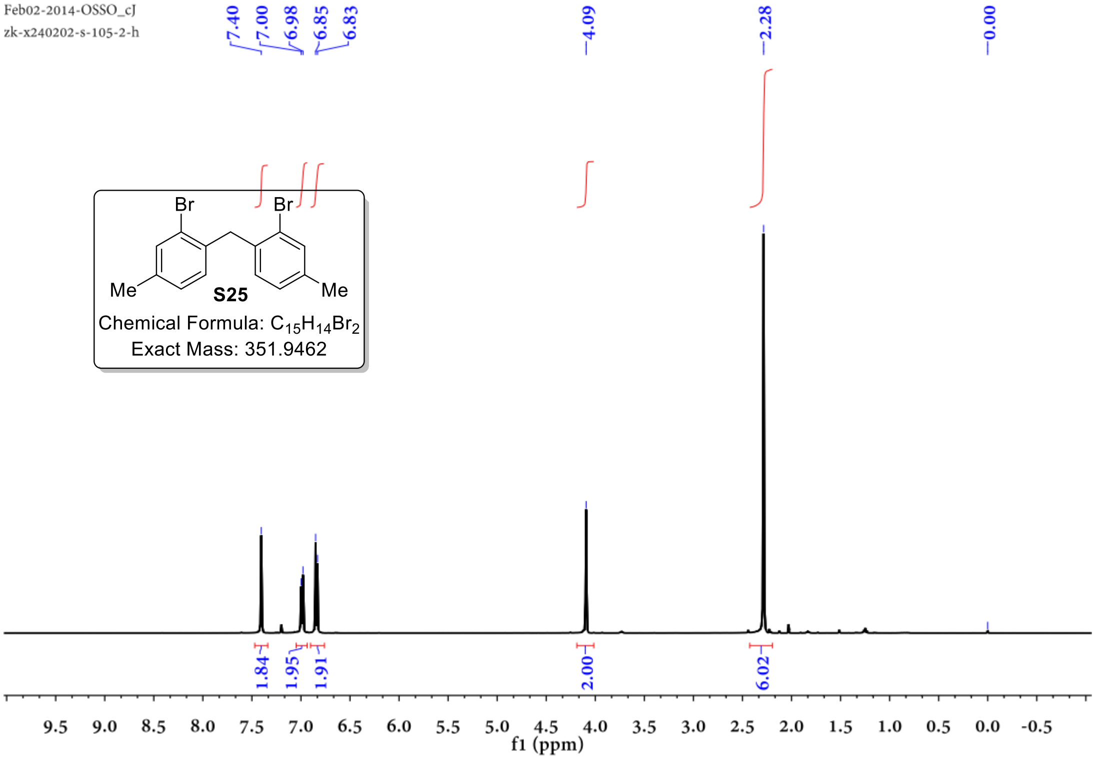

Supplementary Fig. 53 ^{1}H NMR (400 MHz, 20 ºC) spectrum of S25 in $CDCl_3$.

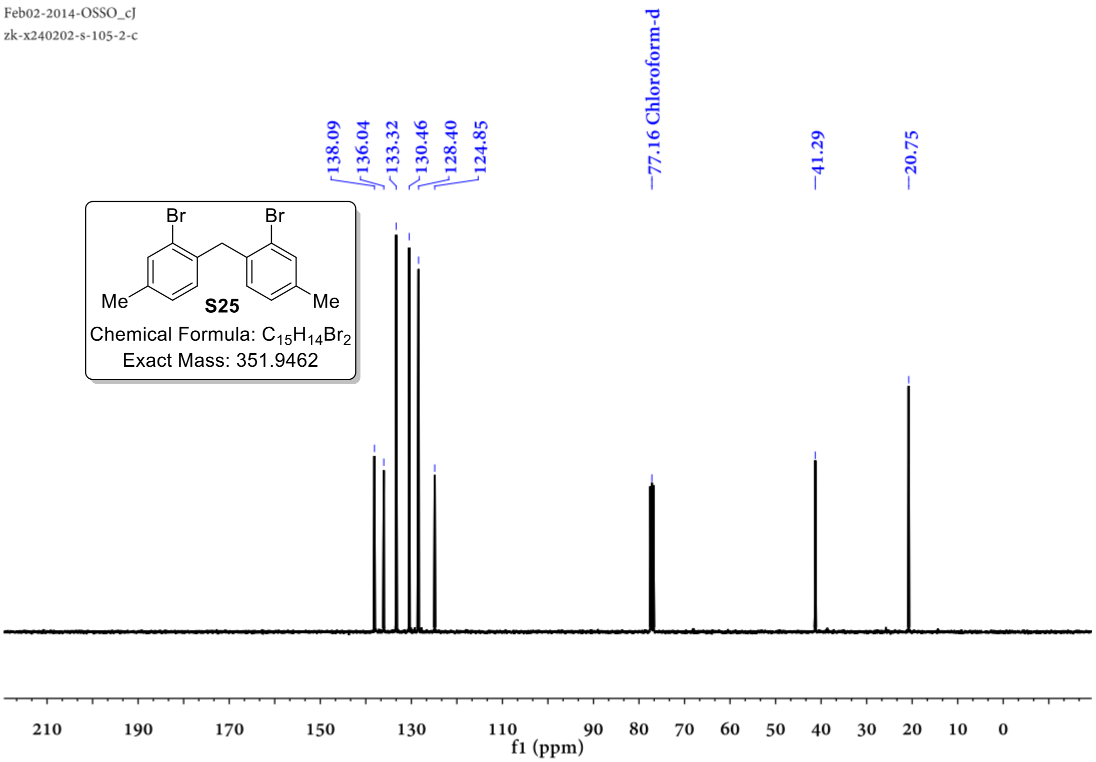

Supplementary Fig. 54 ^{13}C NMR (101 MHz, 20 °C) spectrum of S25 in $CDCl_3$.

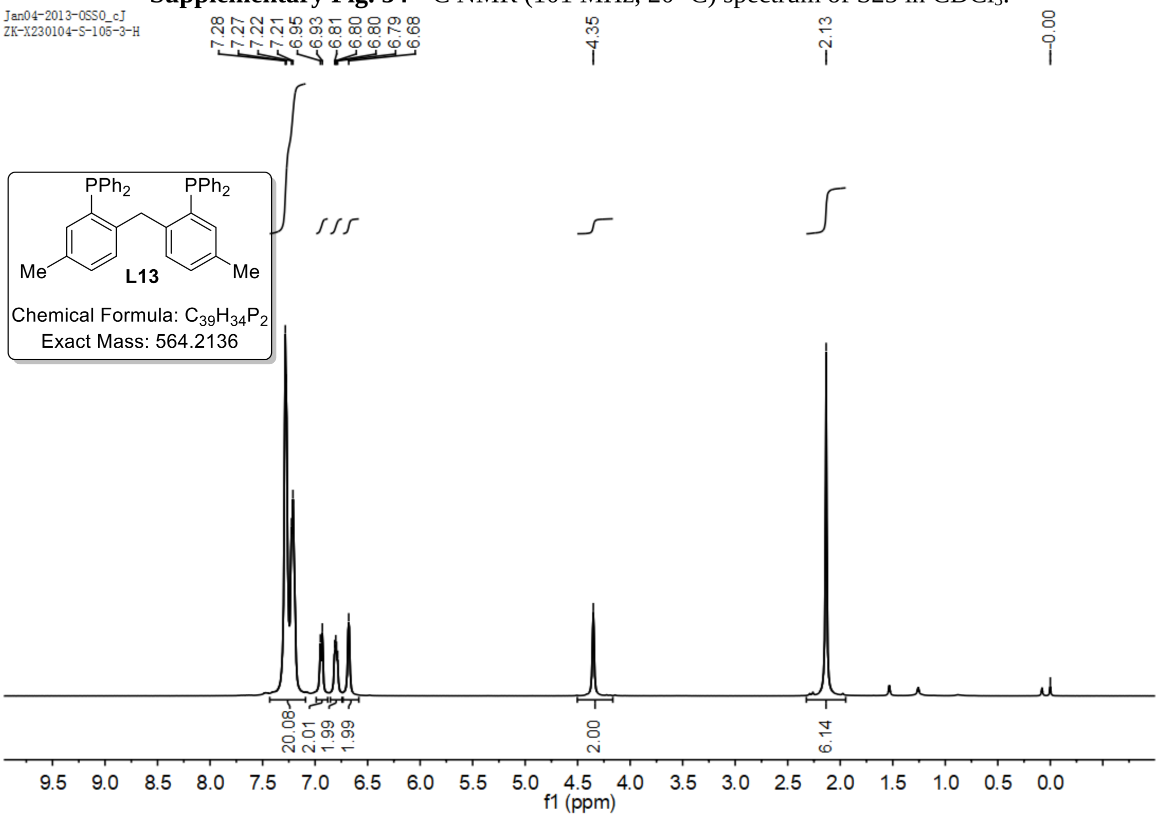

Supplementary Fig. 55 ^{1}H NMR (400 MHz, 20 °C) spectrum of L13 in $CDCl_3$.

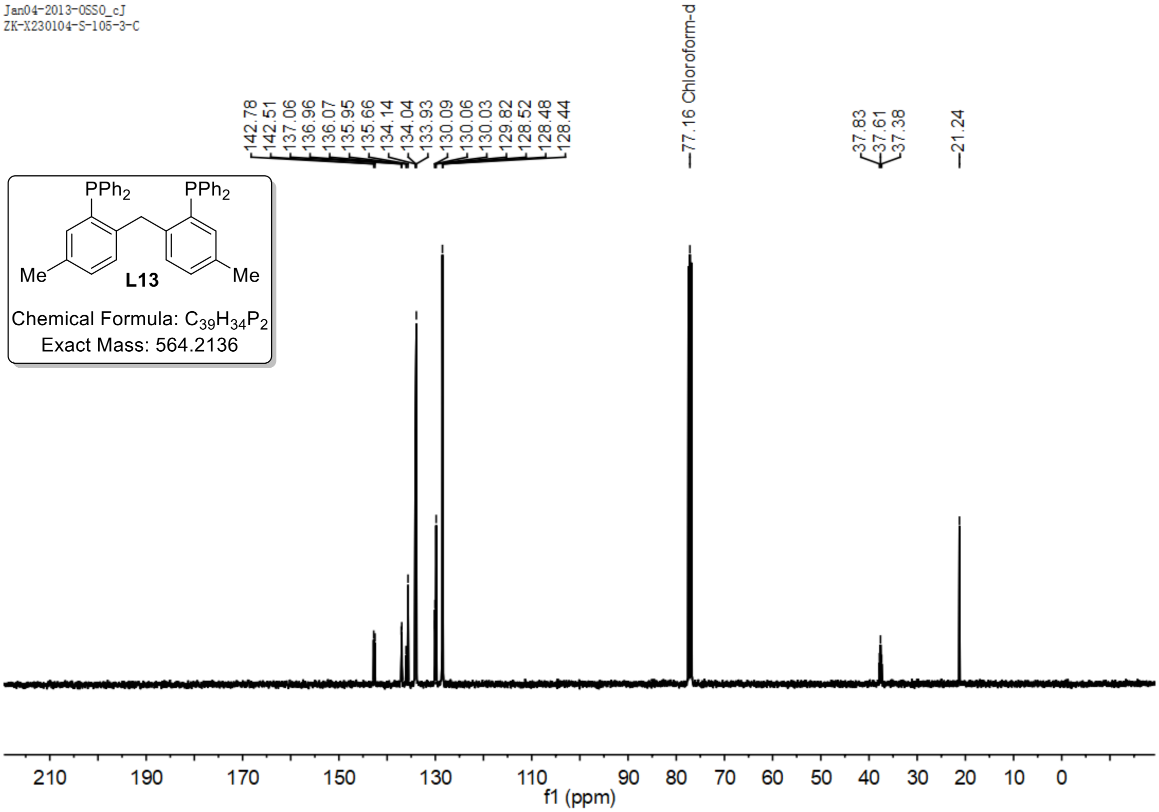

Supplementary Fig. 56 ^{13}C NMR (101 MHz, 20 ºC) spectrum of L13 in $CDCl_3$.

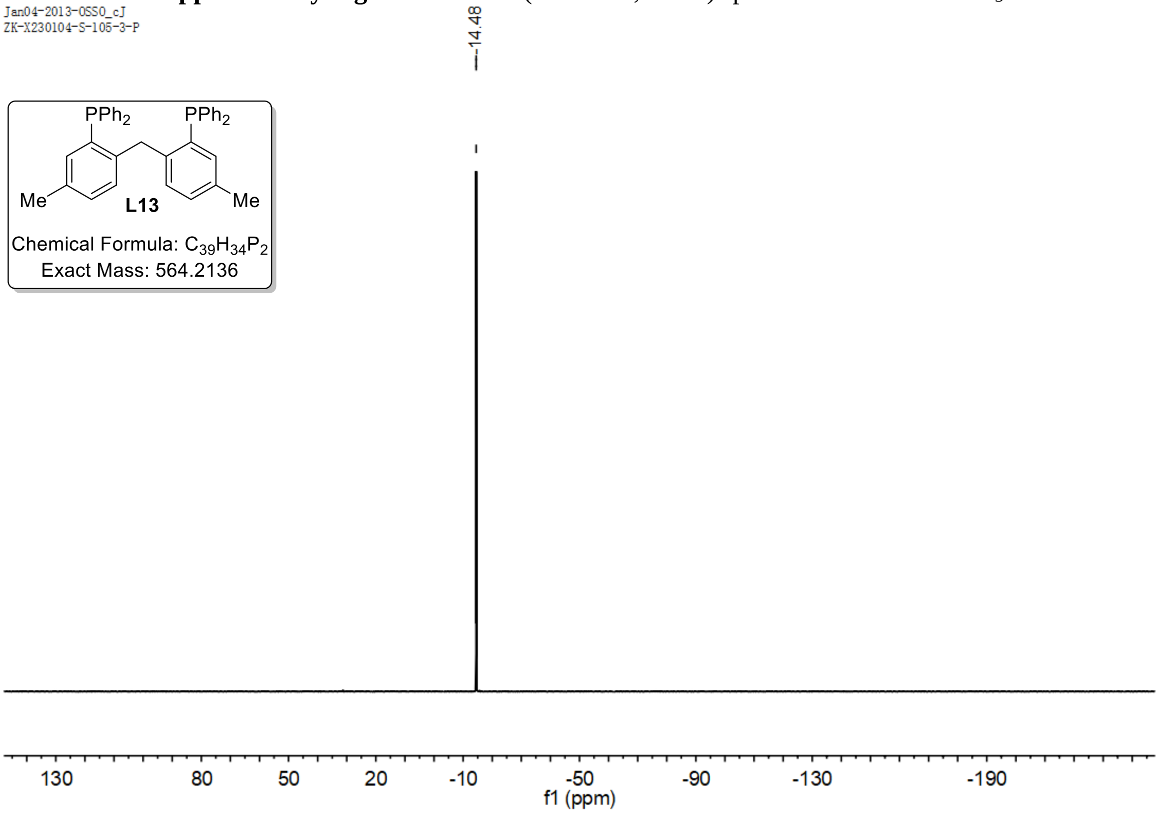

Supplementary Fig. 57 ^{31}P NMR (162 MHz, 20 ºC) spectrum of L13 in $CDCl_3$.

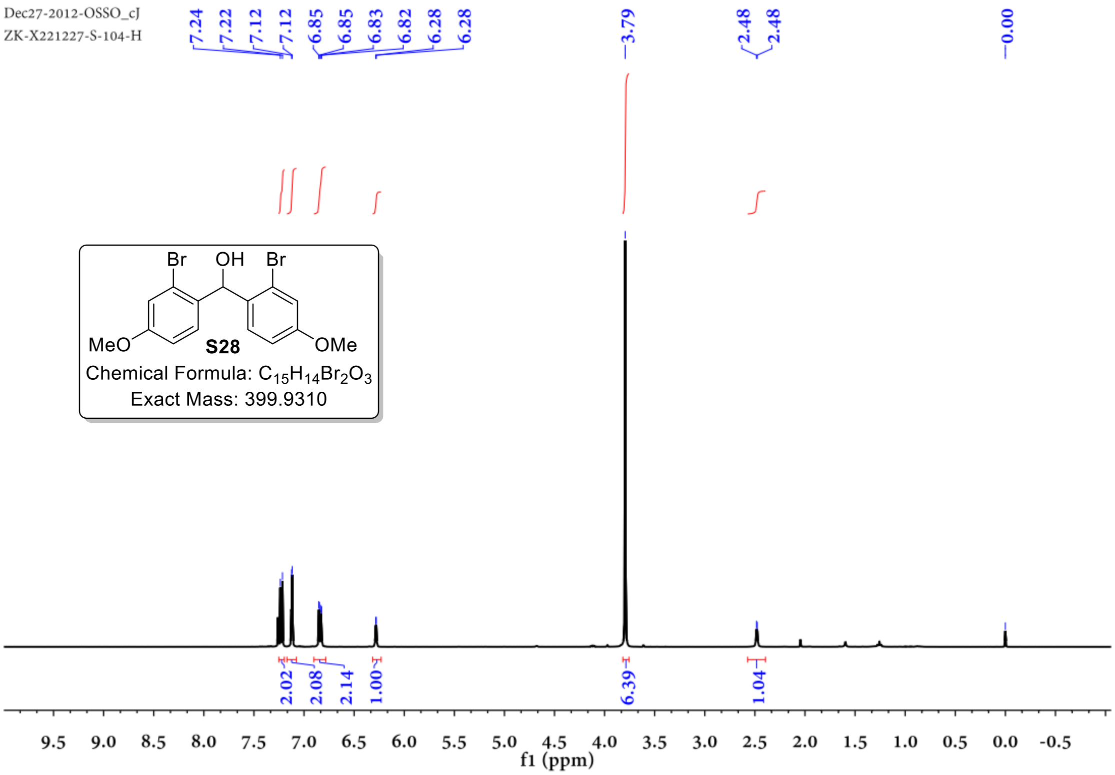

Supplementary Fig. 58 ^{1}H NMR (400 MHz, 20 ºC) spectrum of S28 in $CDCl_3$.

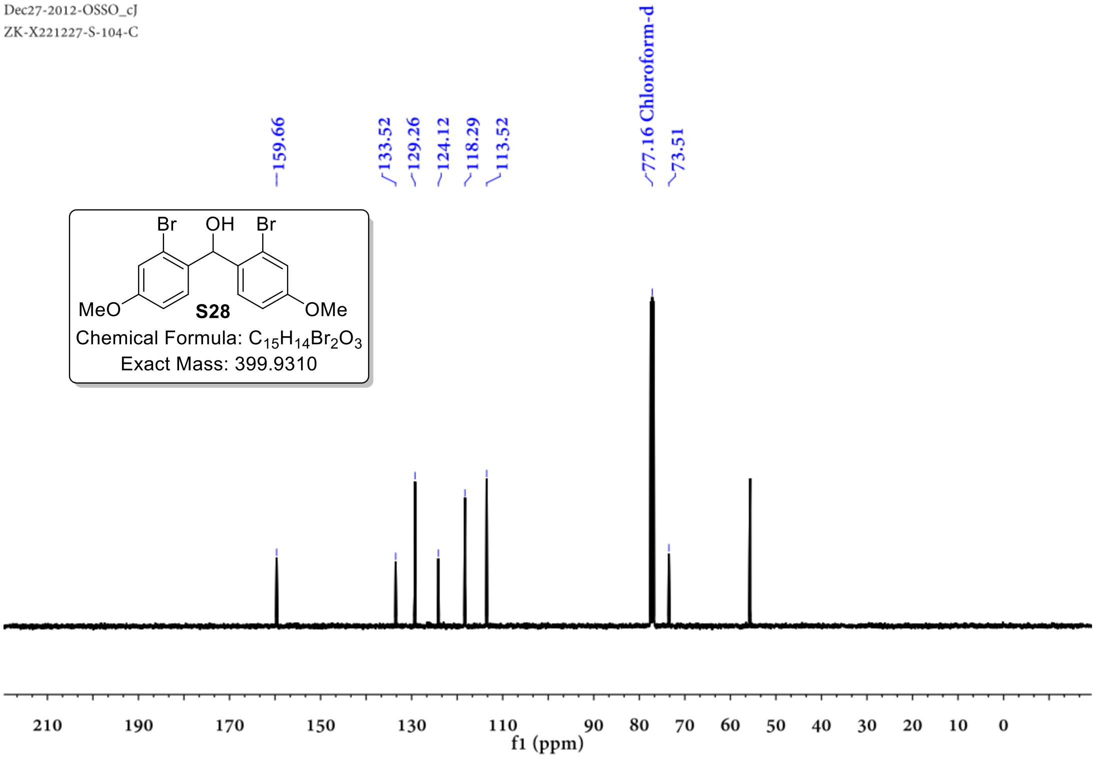

Supplementary Fig. 59 ^{13}C NMR (101 MHz, 20 ºC) spectrum of S28 in $CDCl_3$.

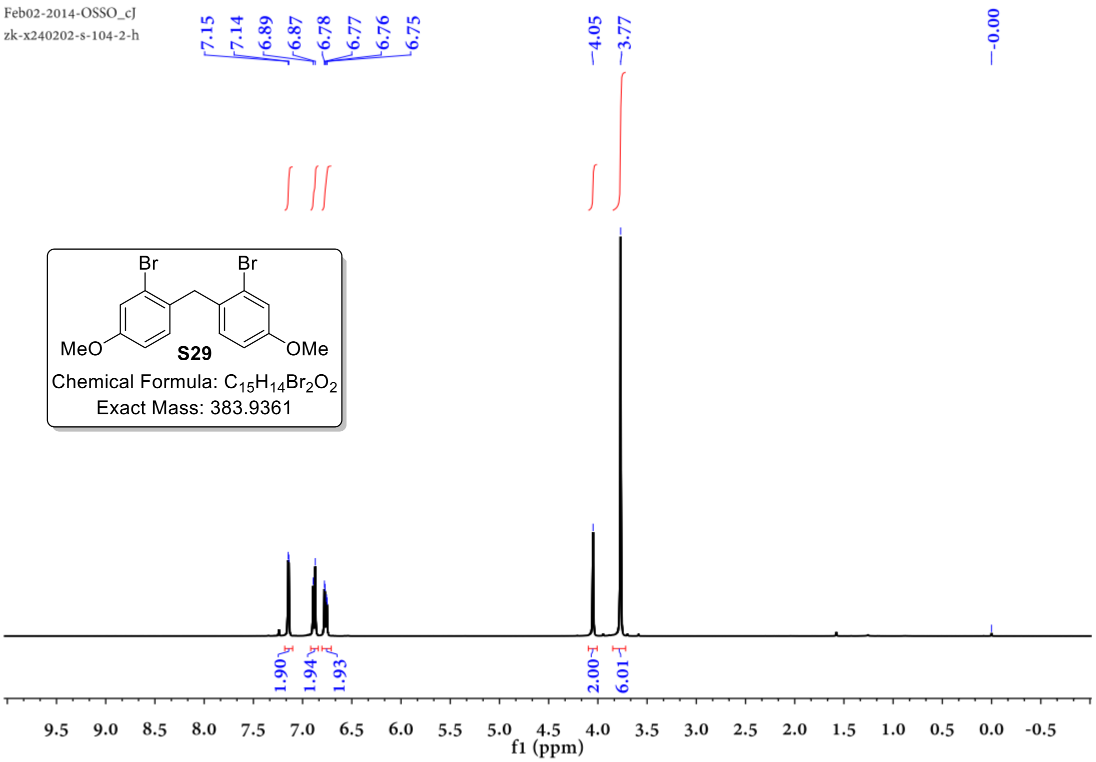

Supplementary Fig. 60 ^{1}H NMR (400 MHz, 20 °C) spectrum of S29 in $CDCl_3$.

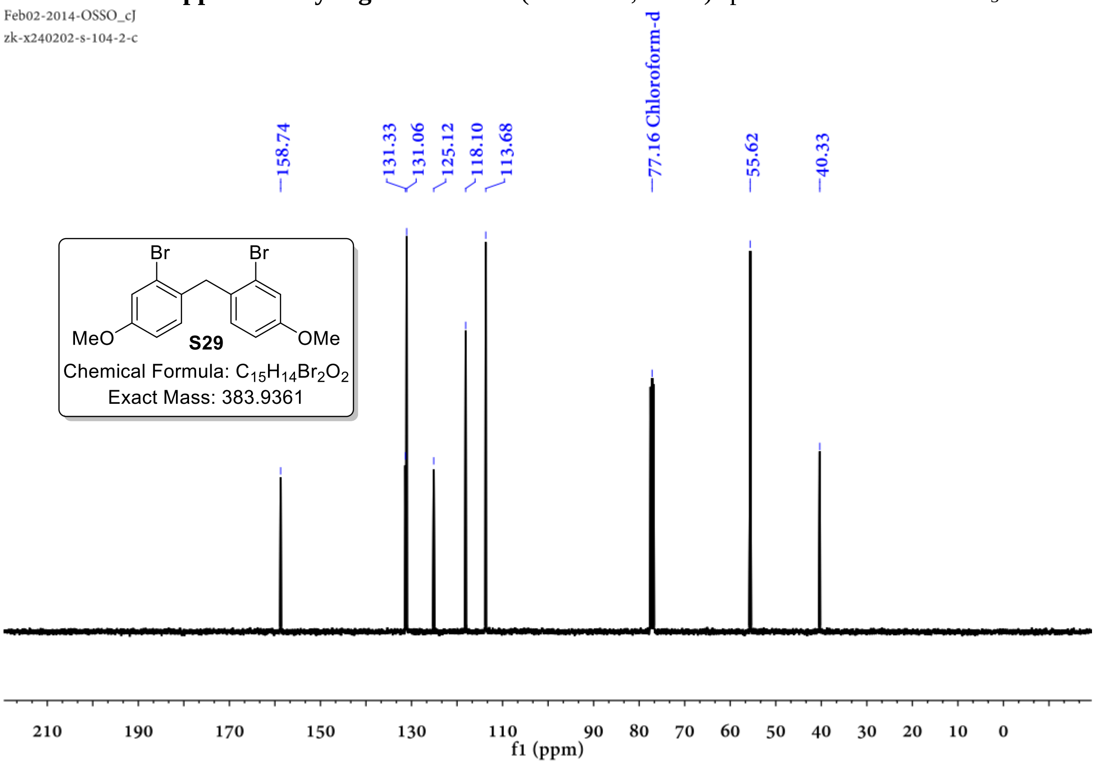

Supplementary Fig. 61 ^{13}C NMR (101 MHz, 20 °C) spectrum of S29 in $CDCl_3$.

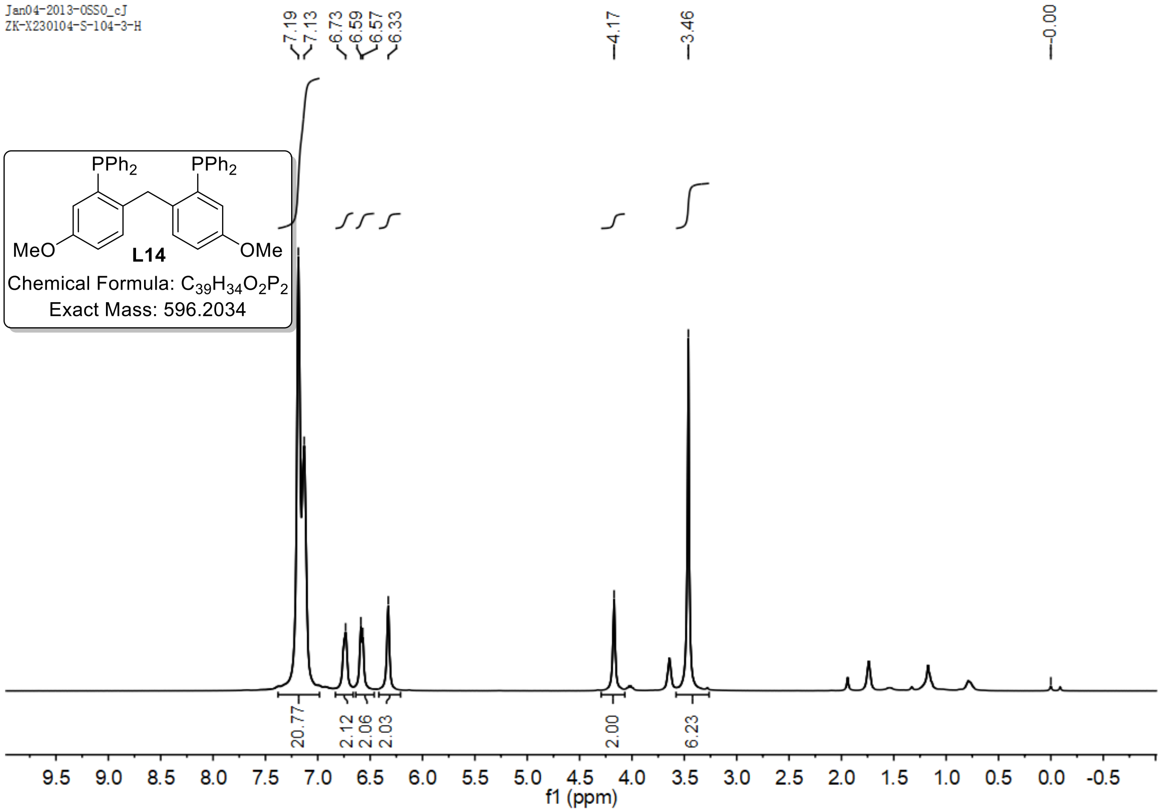

Supplementary Fig. 62 ^{1}H NMR (400 MHz, 20 °C) spectrum of L14 in $CDCl_3$.

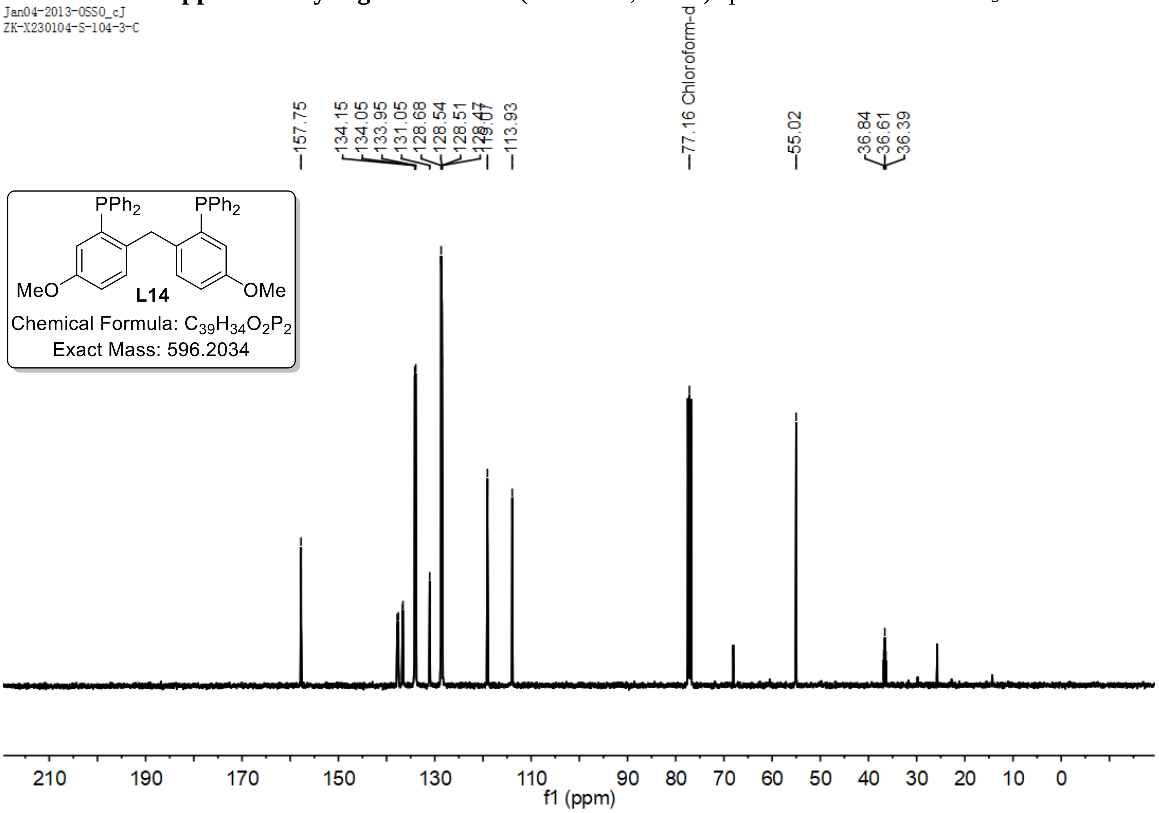

Supplementary Fig. 63 ^{13}C NMR (101 MHz, 20 °C) spectrum of L14 in $CDCl_3$.

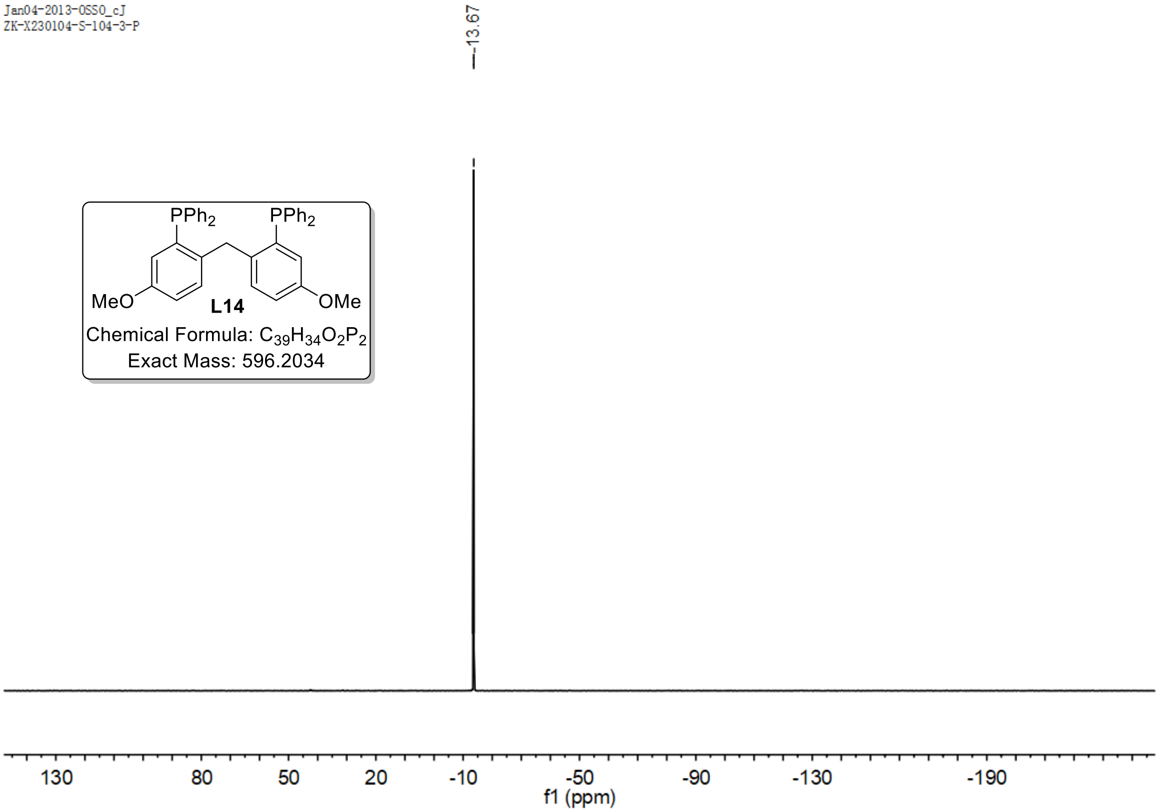

Supplementary Fig. 64 ^{31}P NMR (162 MHz, 20 ºC) spectrum of L14 in $CDCl_3$.

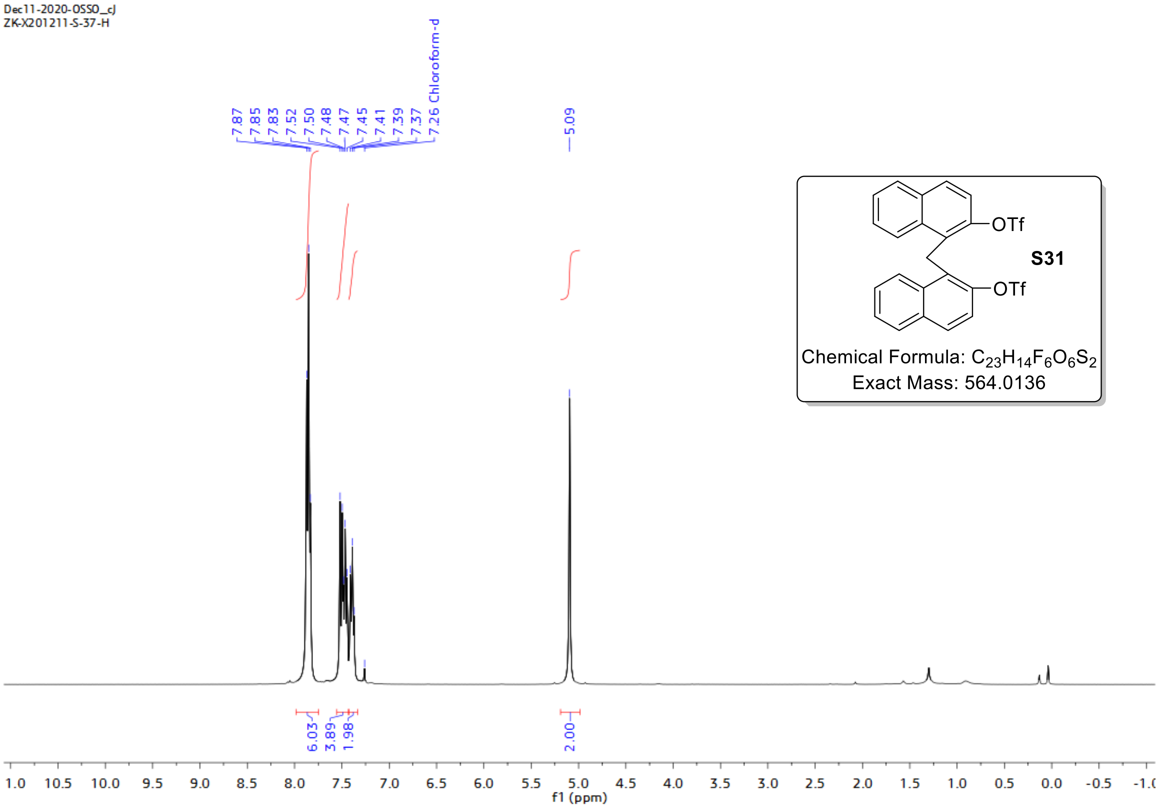

Supplementary Fig. 65 ^{1}H NMR (101 MHz, 20 ºC) spectrum of S31 in $CDCl_3$.

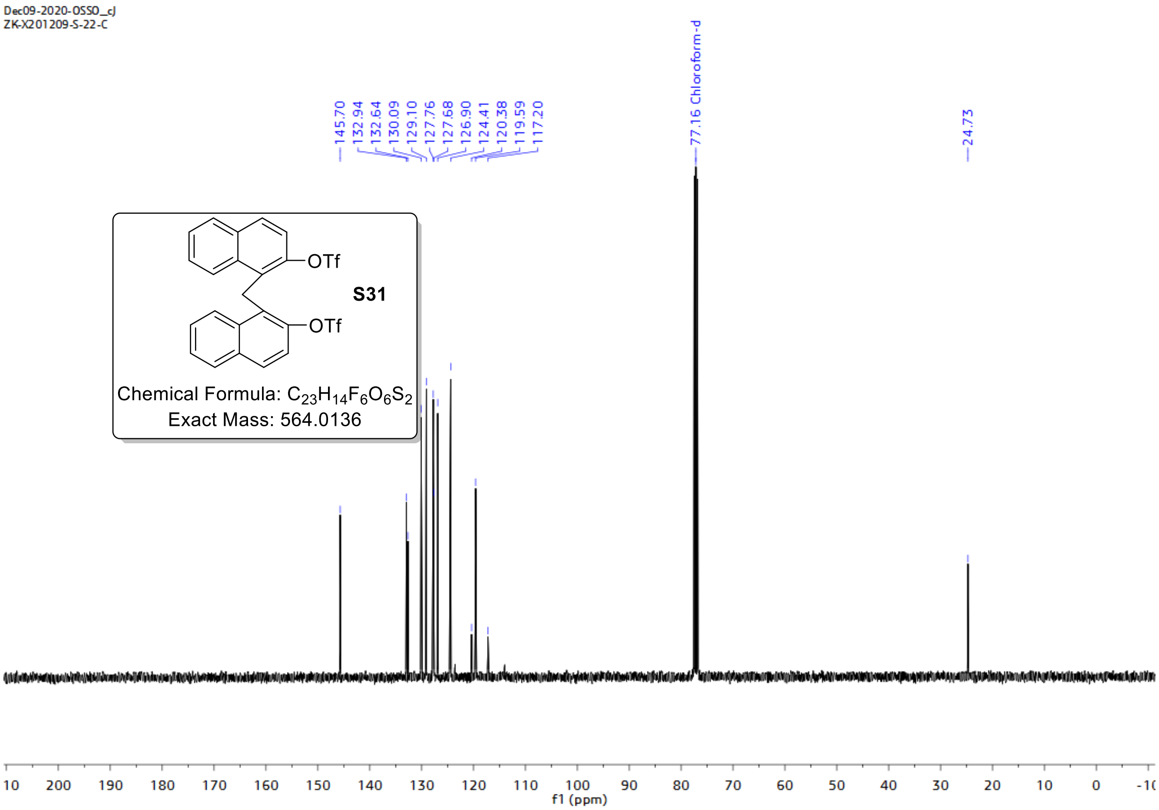

Supplementary Fig. 66 ^{13}C NMR (101 MHz, 20 °C) spectrum of S31 in $CDCl_3$.

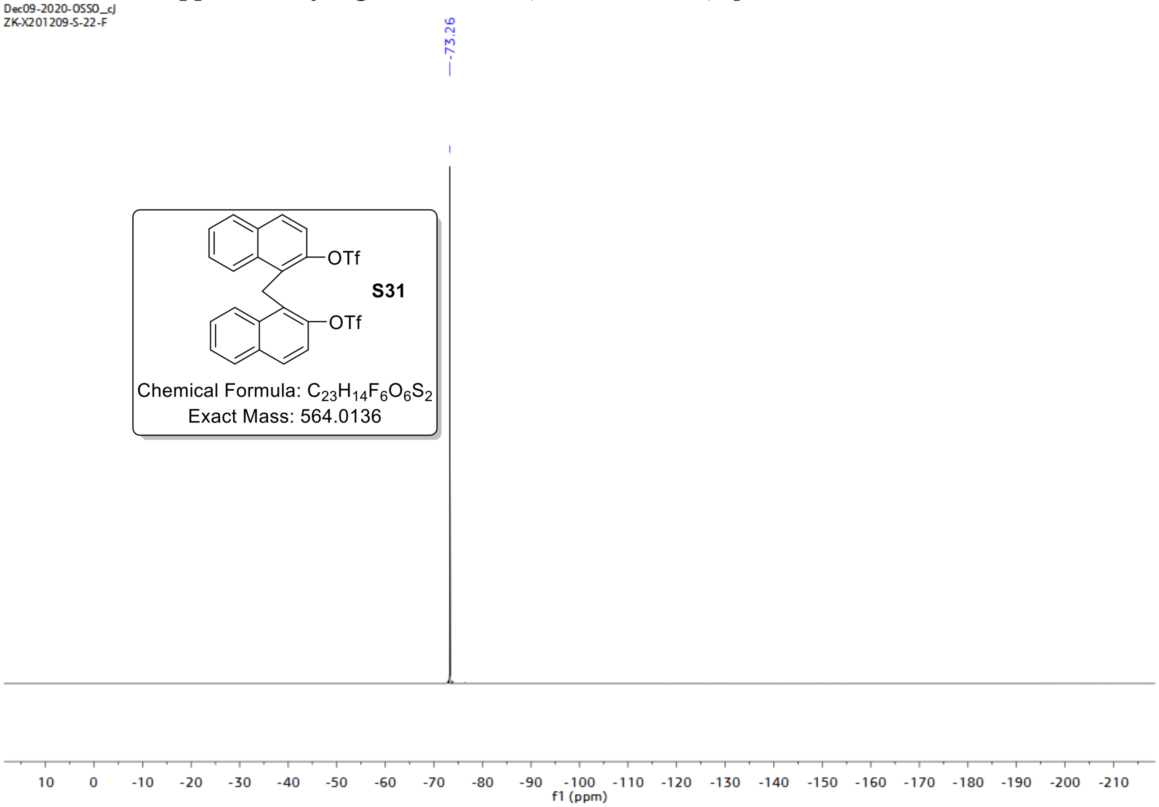

Supplementary Fig. 67 ^{19}F NMR (376 MHz, 20 °C) spectrum of S31 in $CDCl_3$.

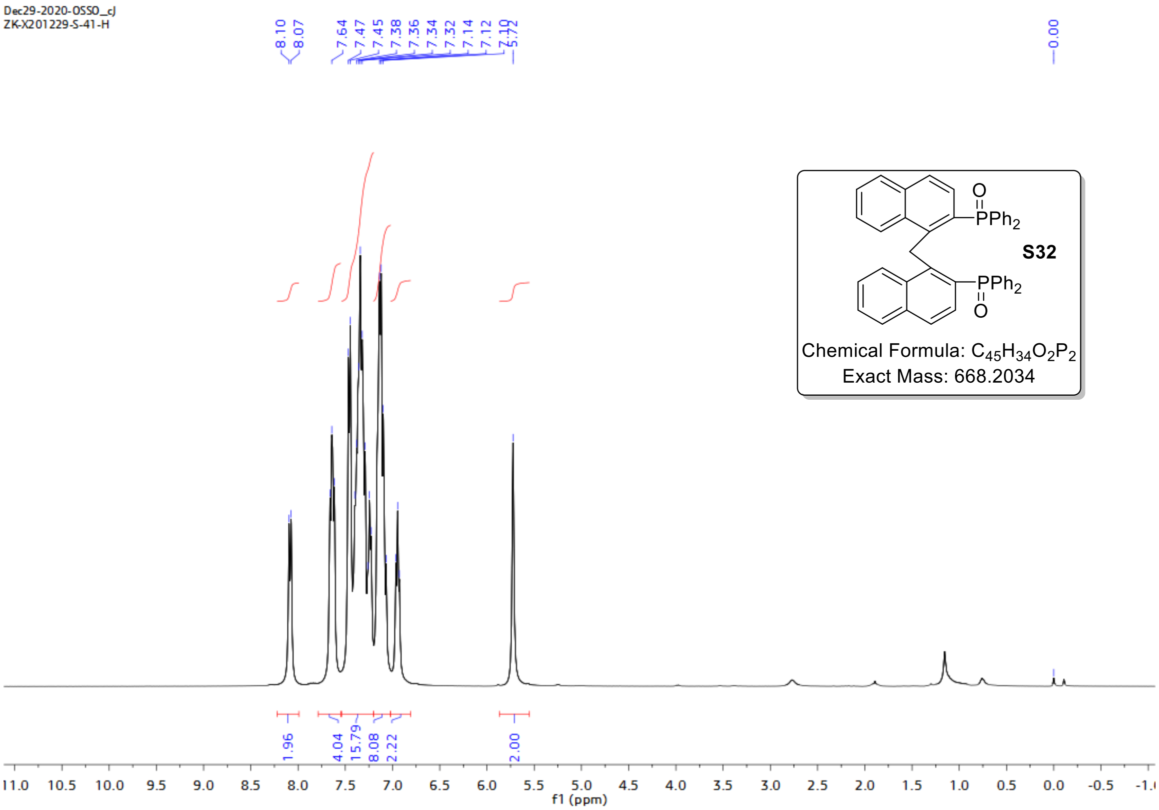

Supplementary Fig. 68 ^{1}H NMR (400 MHz, 20 °C) spectrum of S32 in $CDCl_3$.

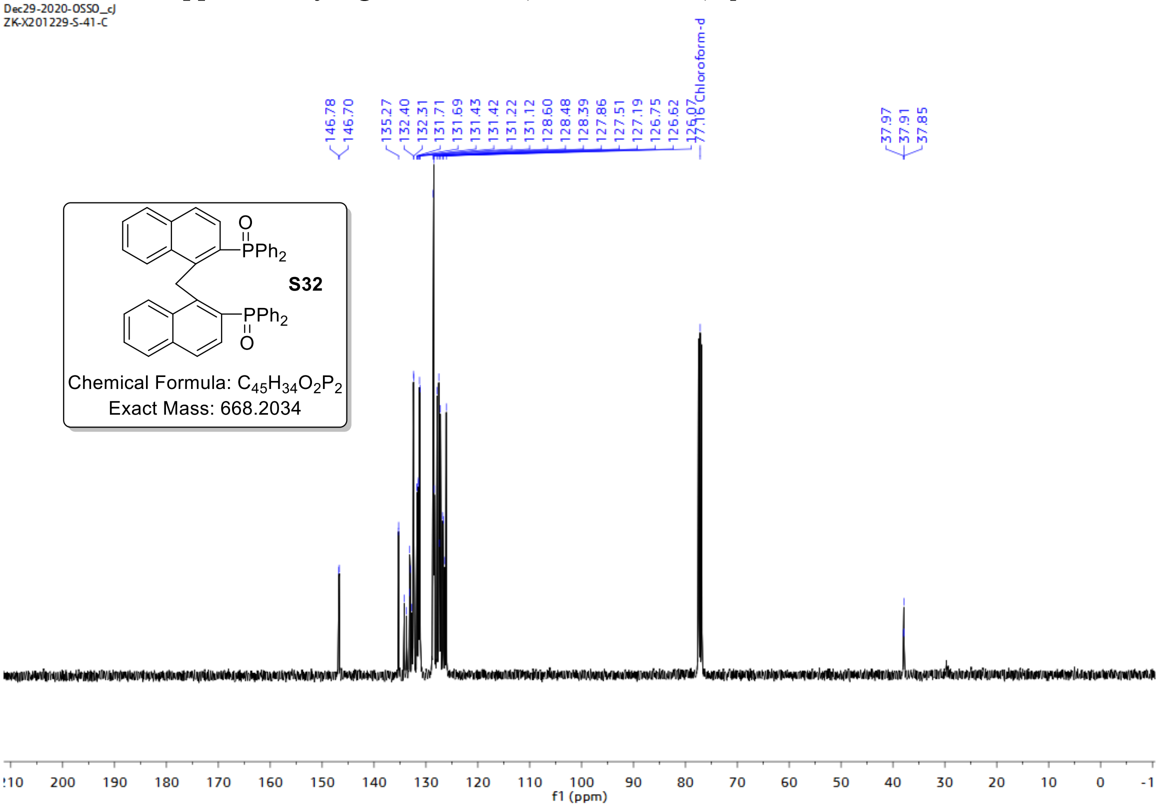

Supplementary Fig. 69 ^{13}C NMR (101 MHz, 20 °C) spectrum of S32 in $CDCl_3$.

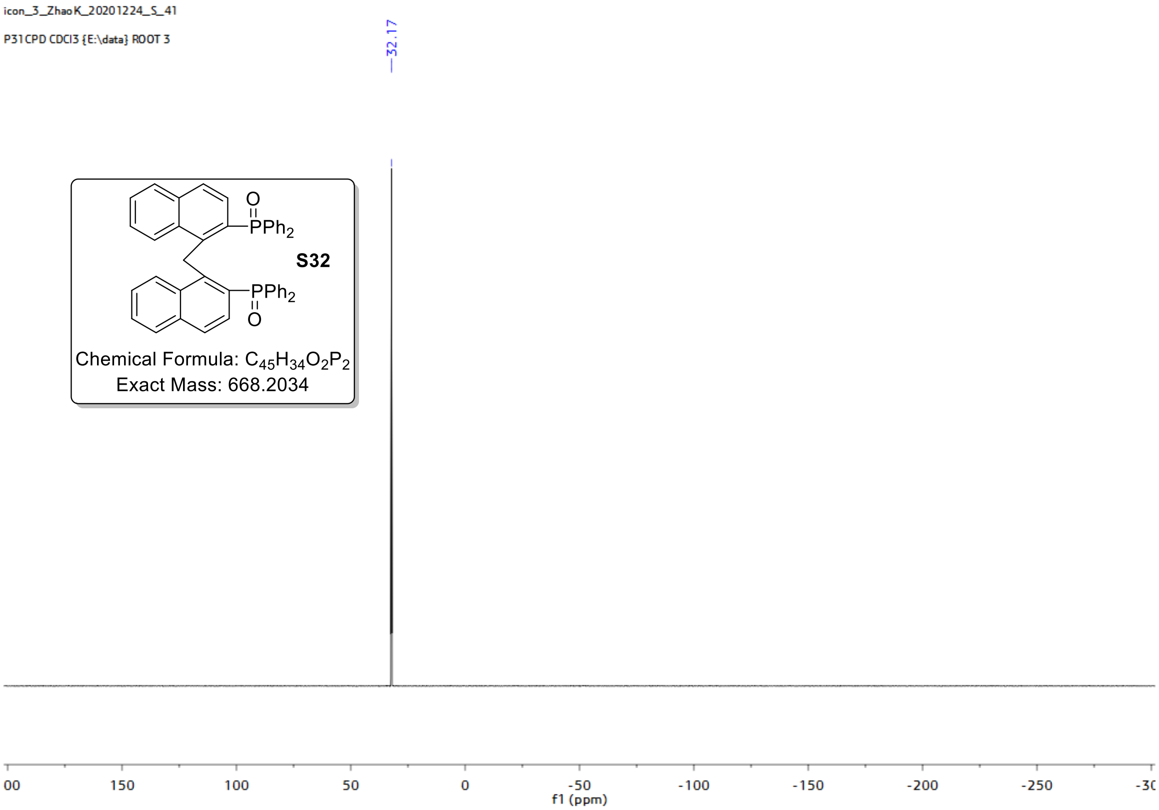

Supplementary Fig. 70 ^{19}F NMR (372 MHz, 20 ºC) spectrum of S32 in $CDCl_3$.

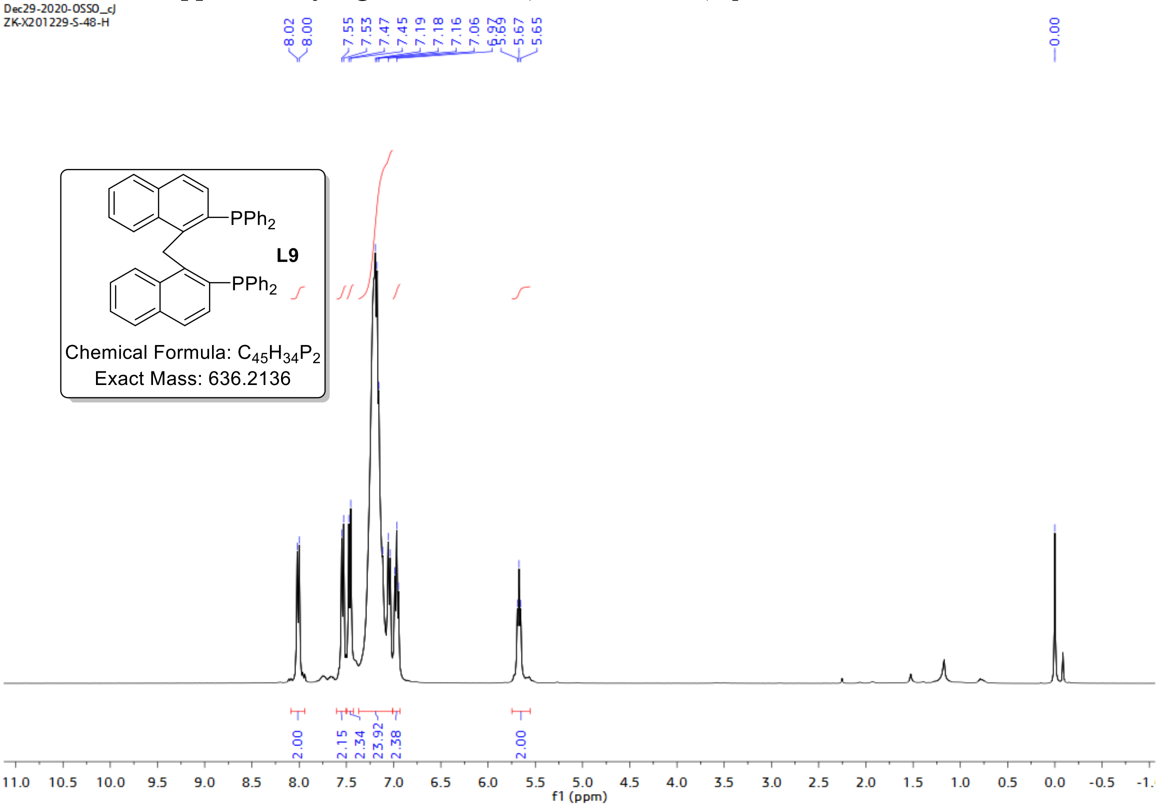

Supplementary Fig. 71 ^{1}H NMR (400 MHz, 20 ºC) spectrum of L19 in $CDCl_3$.

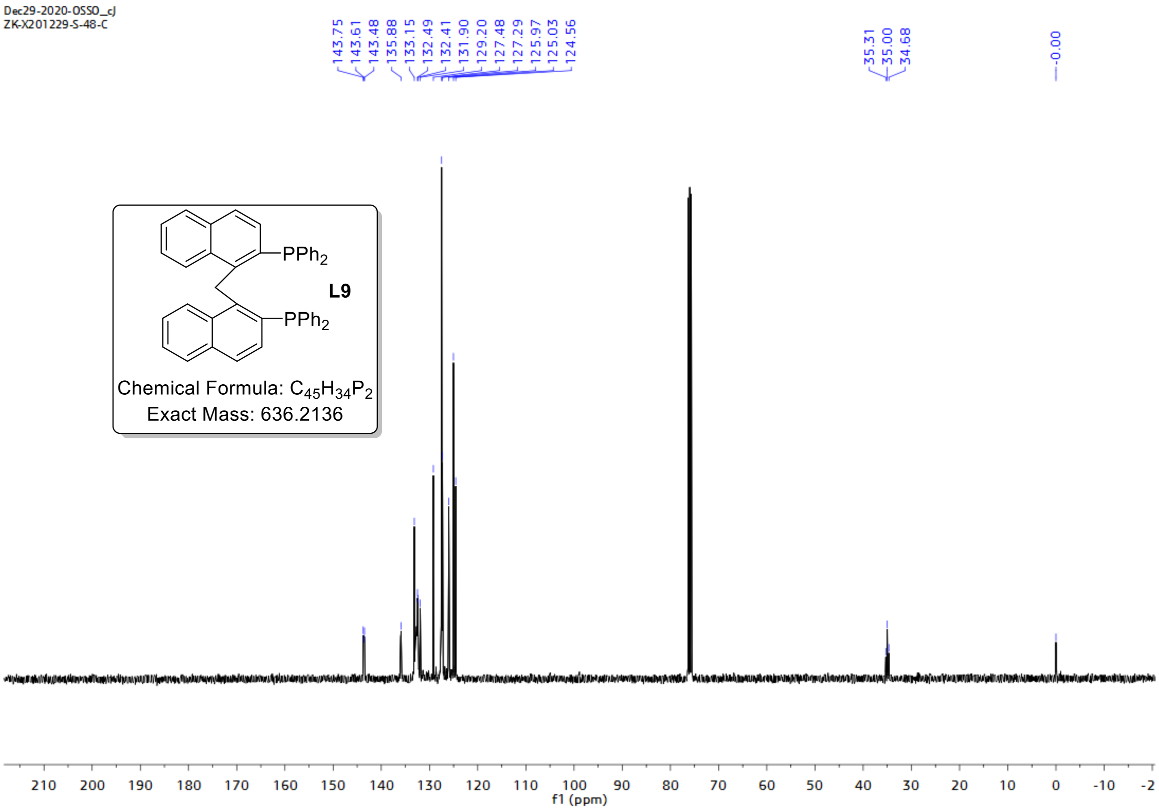

Supplementary Fig. 72 ^{13}C NMR (101 MHz, 20 ºC) spectrum of L9 in $CDCl_3$.

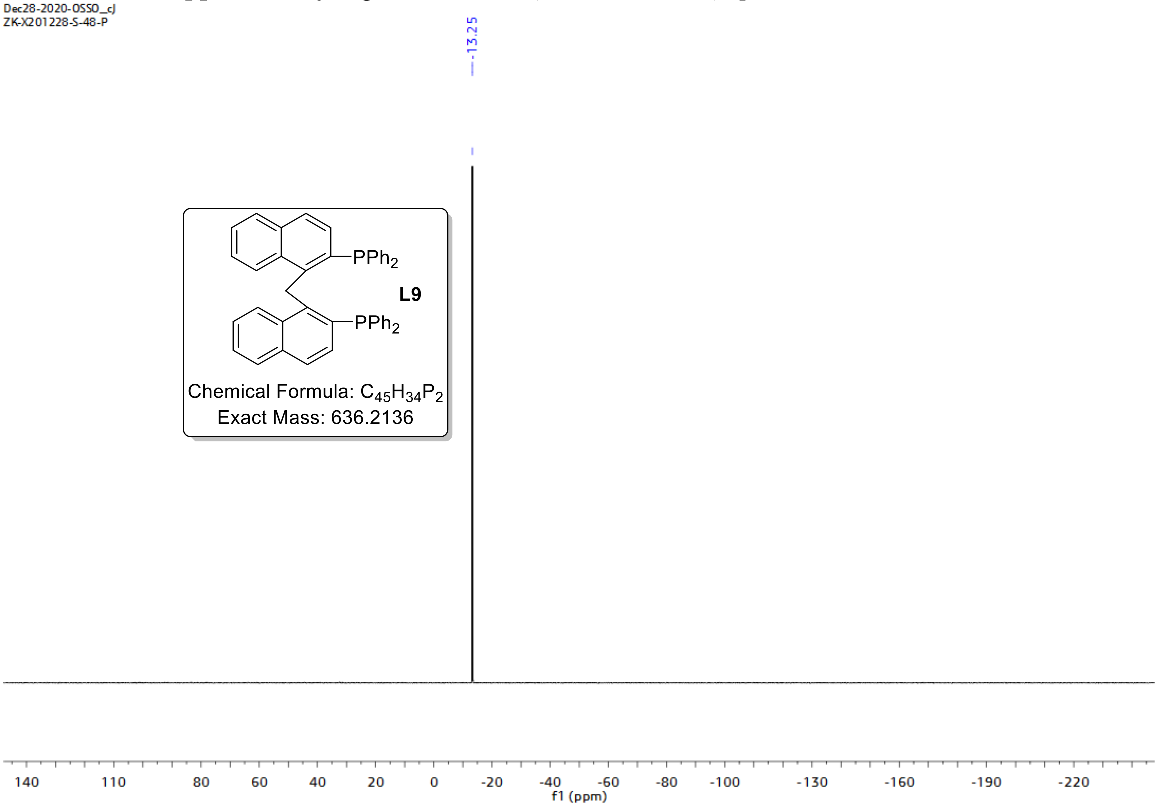

Supplementary Fig. 73 ^{31}P NMR (162 MHz, 20 ºC) spectrum of L9 in $CDCl_3$.

Quantitative GC spectra of the products

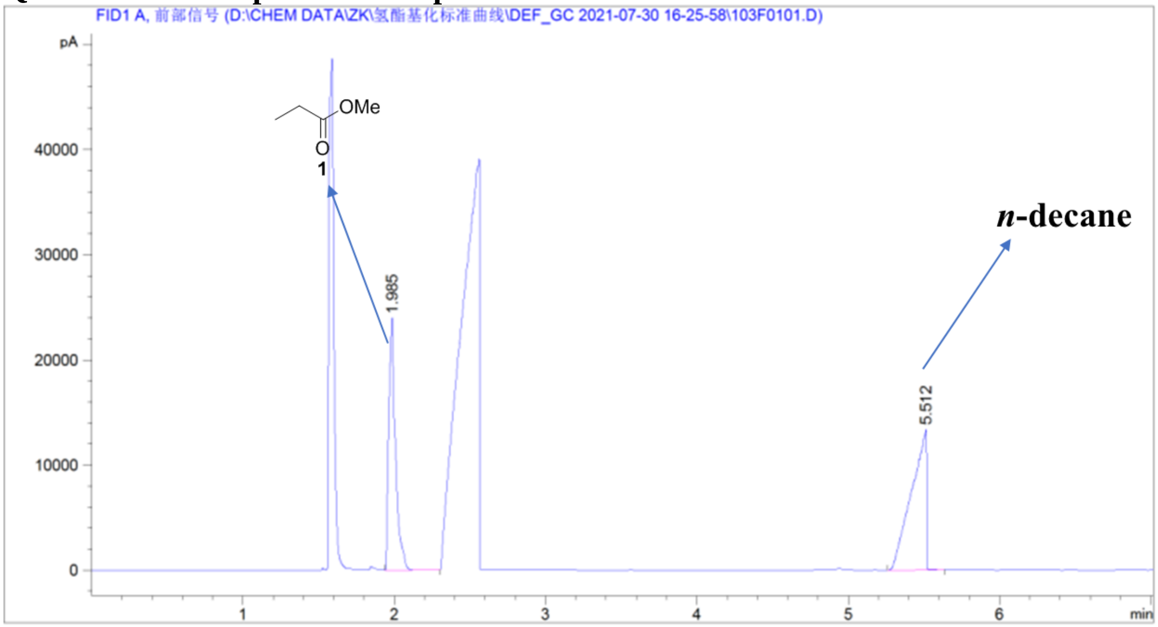

Supplementary Fig. 74 GC spectrum of methyl propionate.

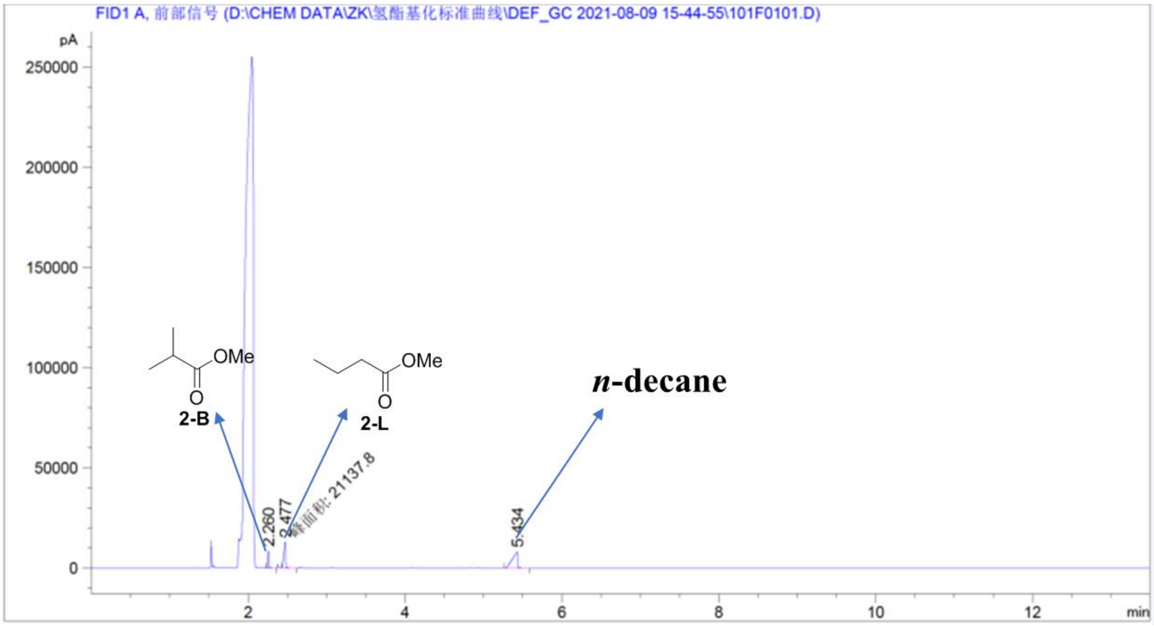

Supplementary Fig. 75 GC spectrum of product 2.

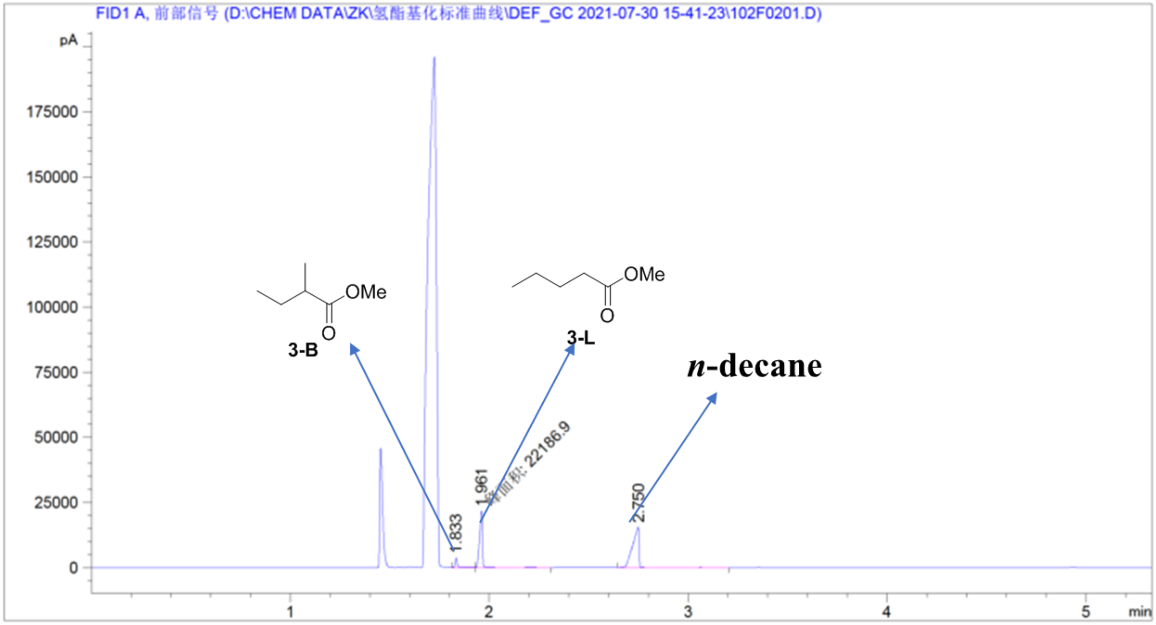

Supplementary Fig. 76 GC spectrum of product 3.

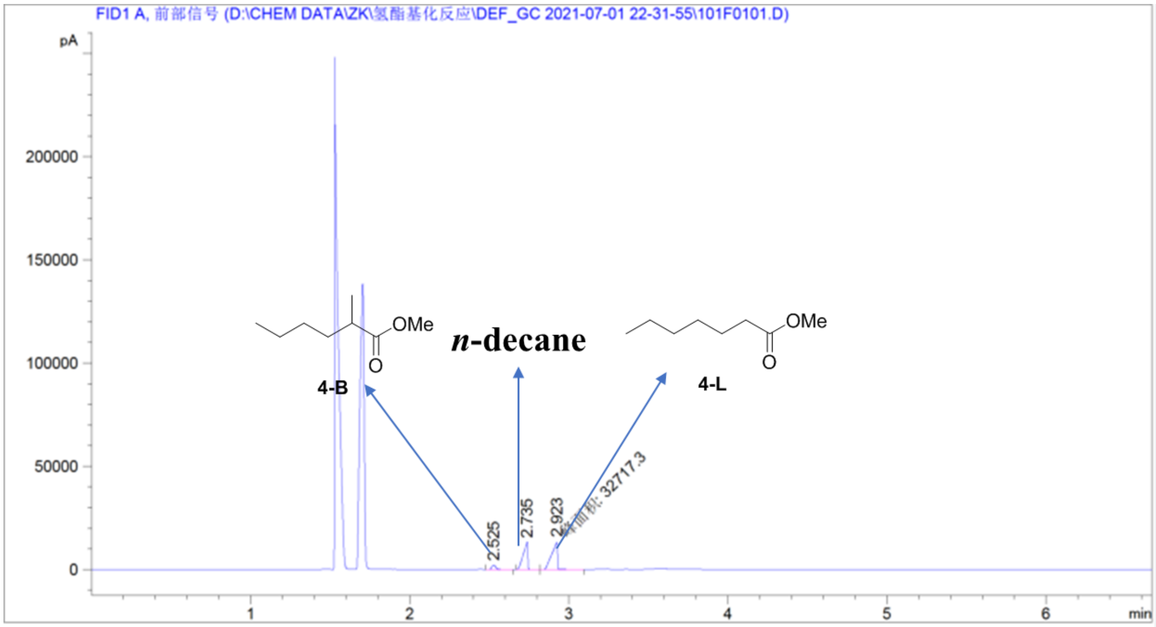

Supplementary Fig. 77 GC spectrum of product 4.

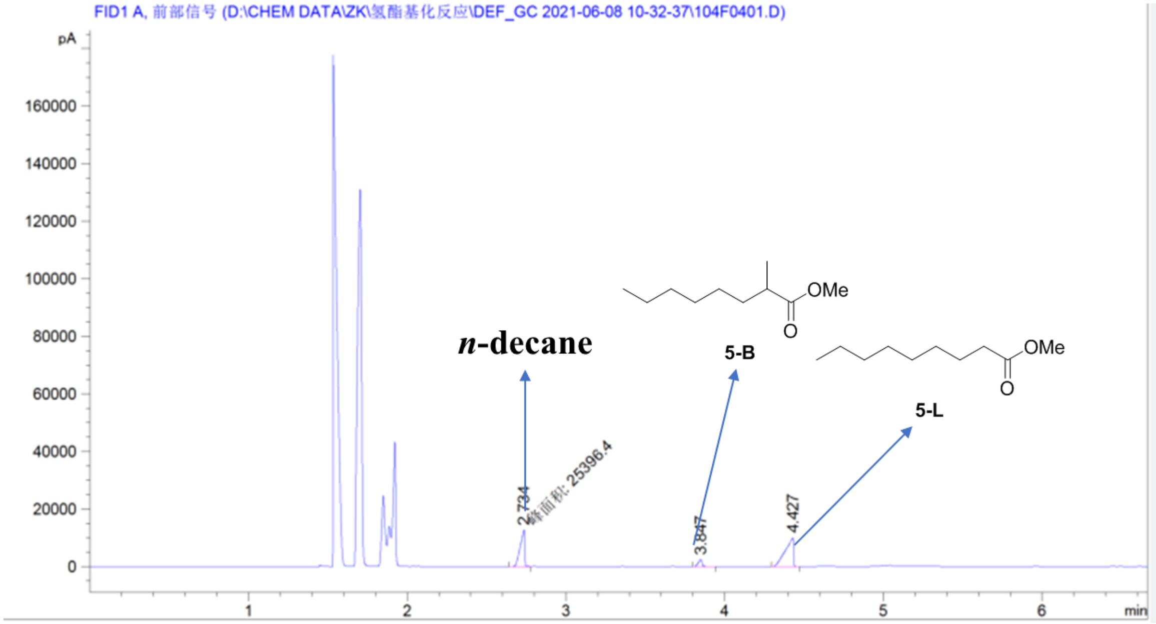

Supplementary Fig. 78 GC spectrum of product 5.

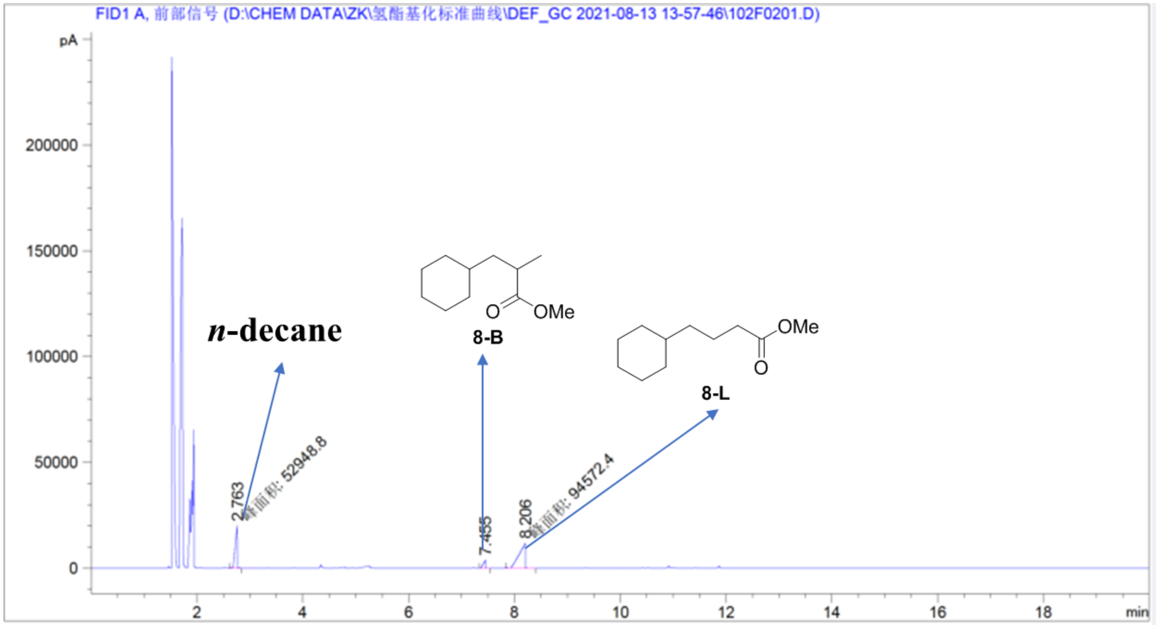

Supplementary Fig. 79 GC spectrum of product 8.

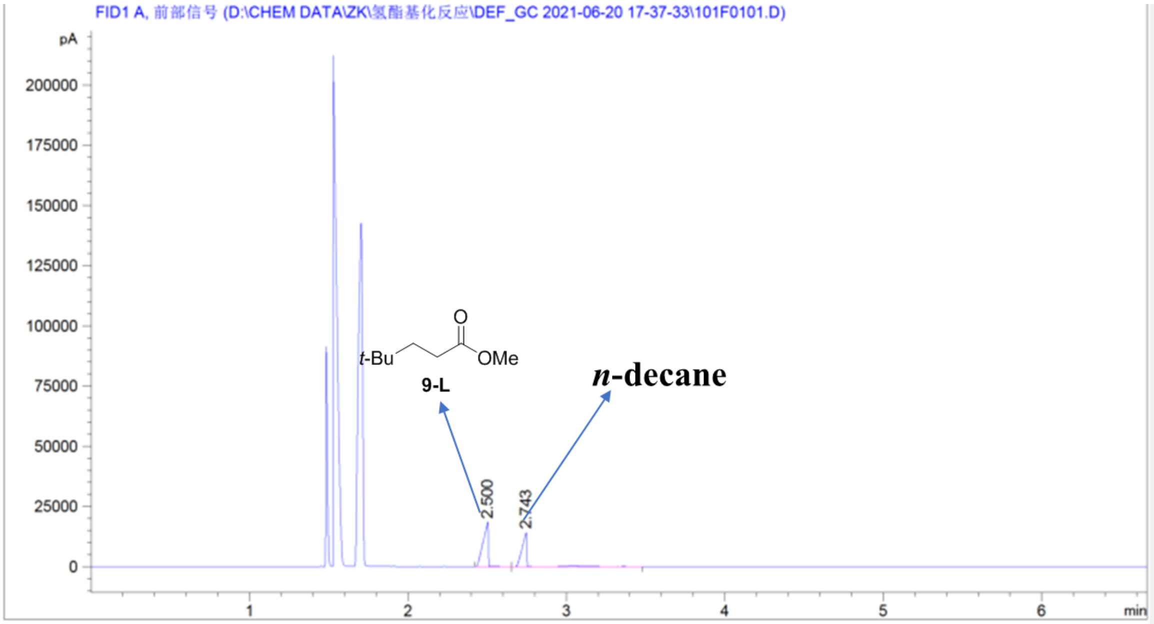

Supplementary Fig. 80 GC spectrum of product 9.

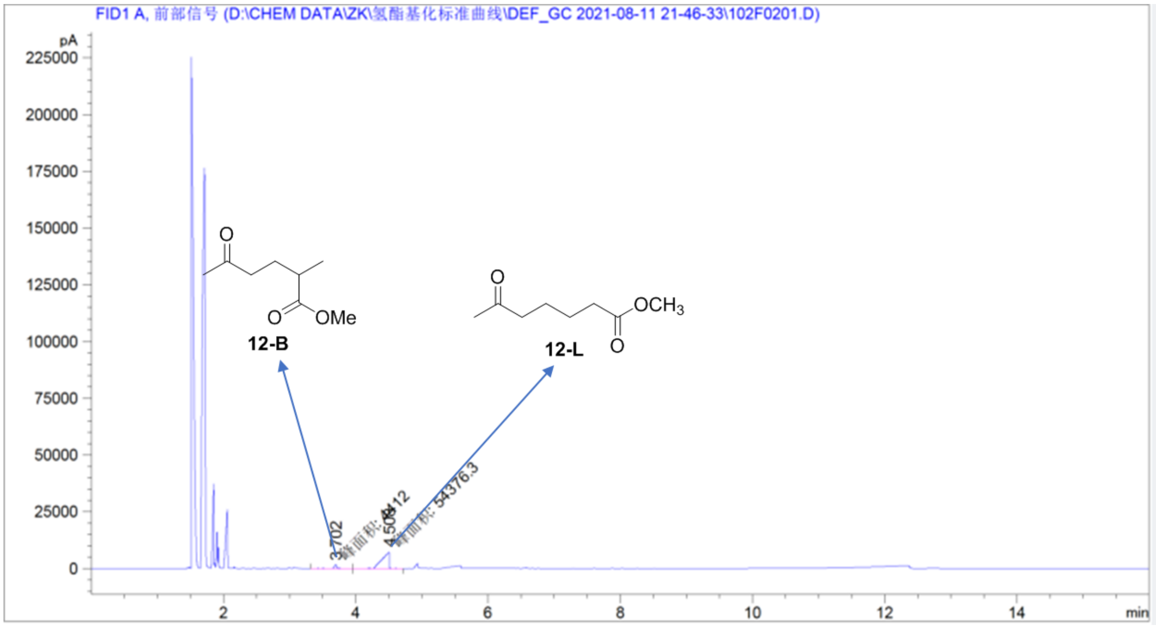

Supplementary Fig. 81 GC spectrum of product 12.

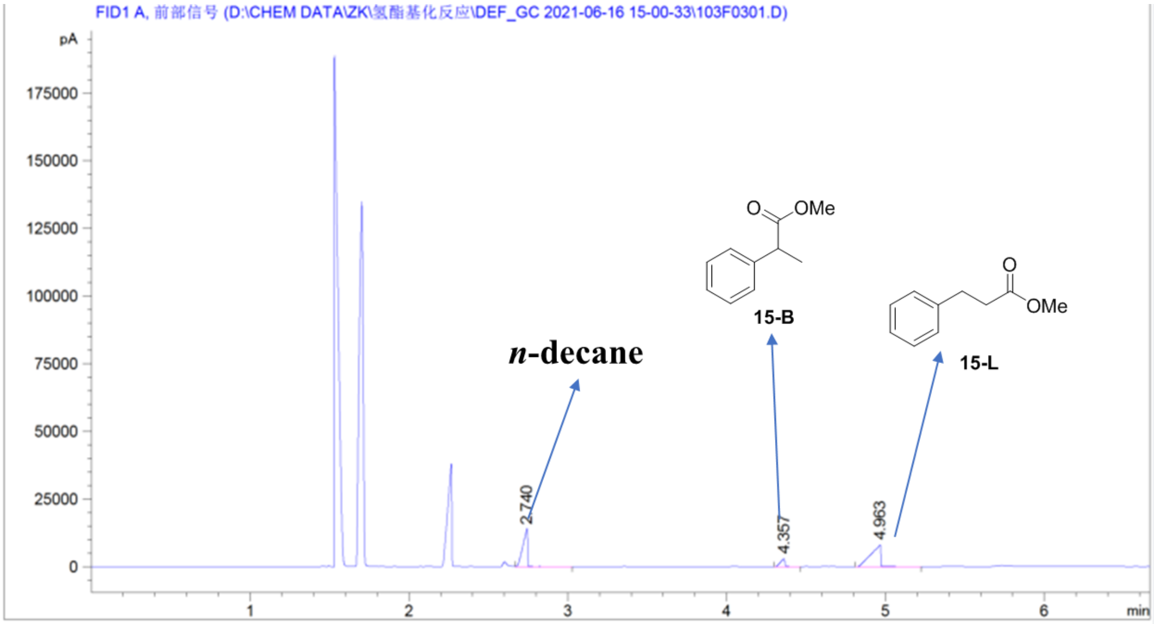

Supplementary Fig. 82 GC spectrum of product 15.

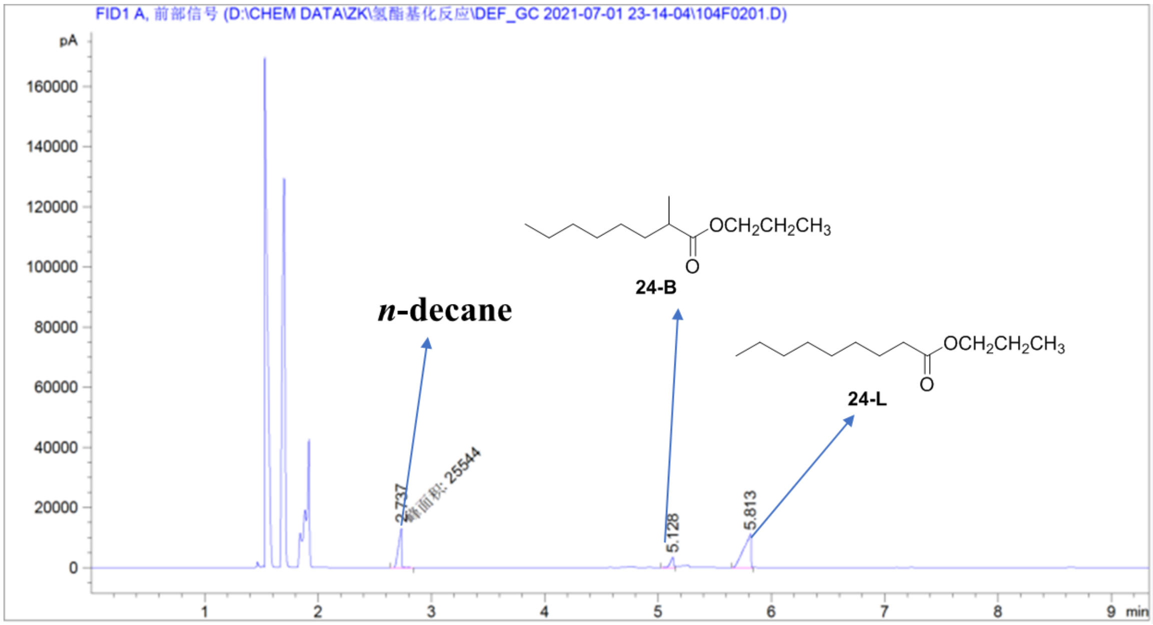

Supplementary Fig. 83 GC spectrum of product 24.

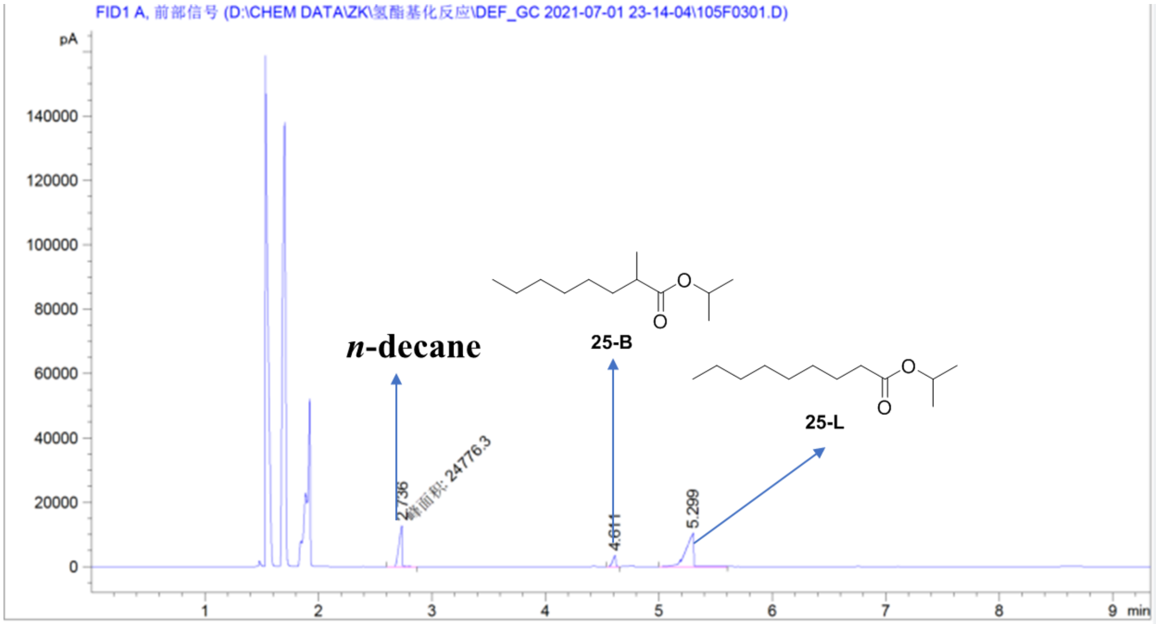

Supplementary Fig. 84 GC spectrum of product 25.

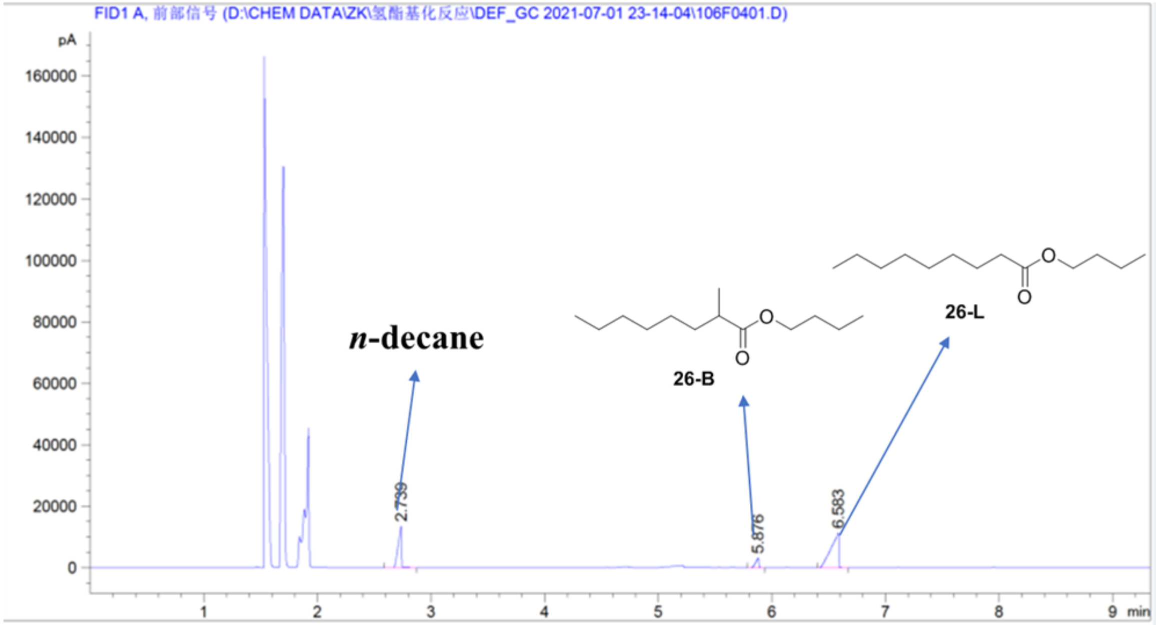

Supplementary Fig. 85 GC spectrum of product 26.

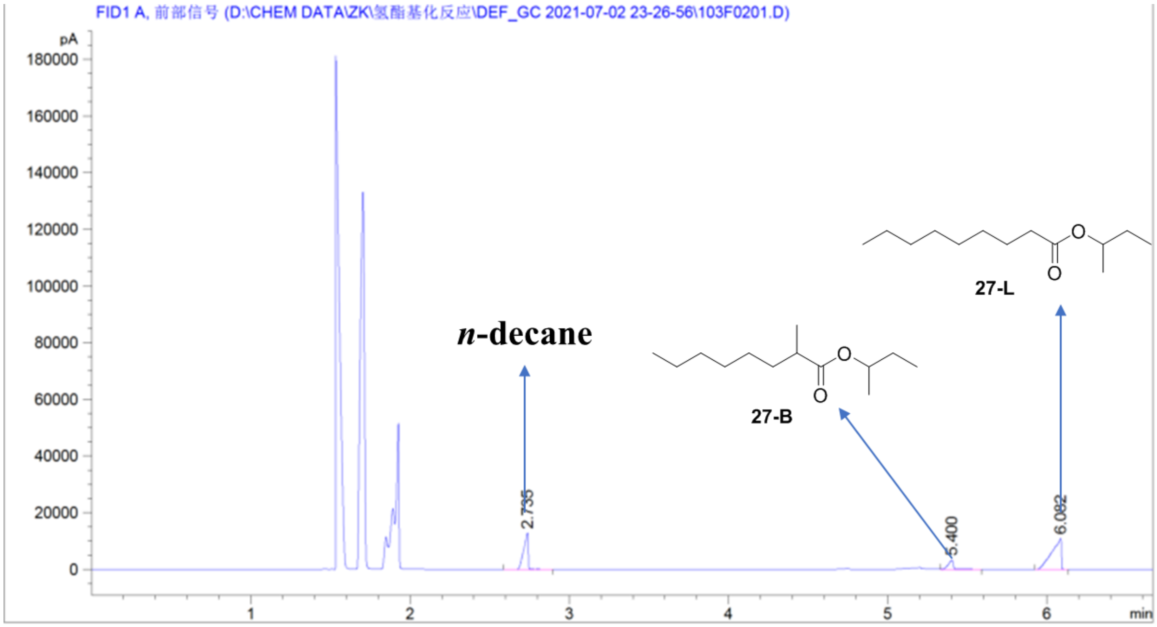

Supplementary Fig. 86 GC spectrum of product 27.

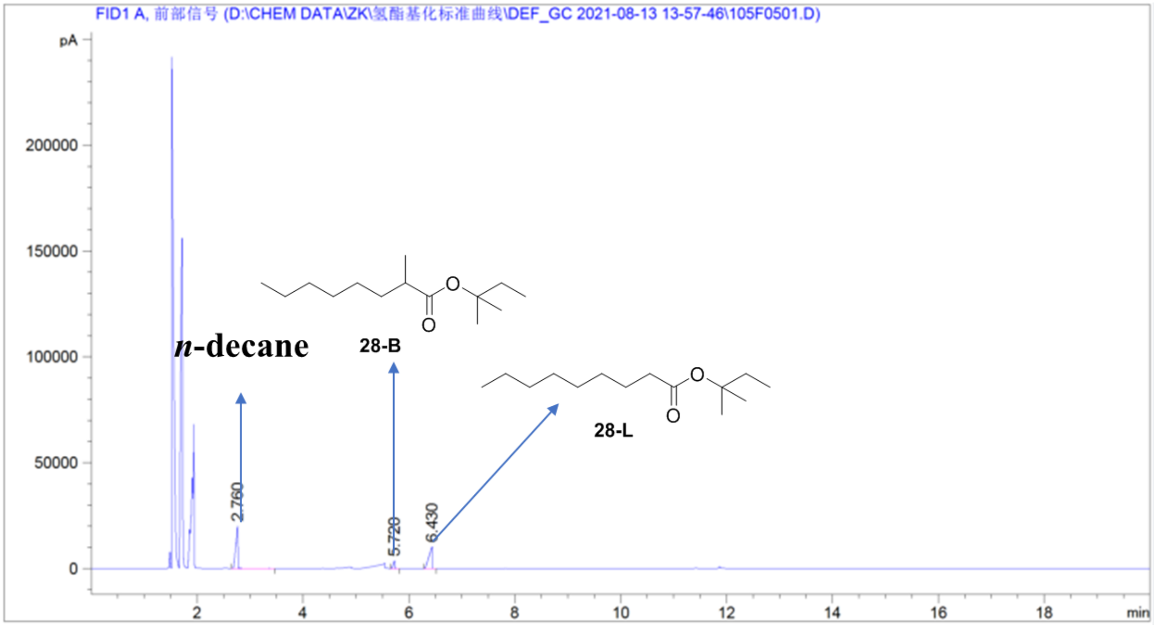

Supplementary Fig. 87 GC spectrum of product 28.

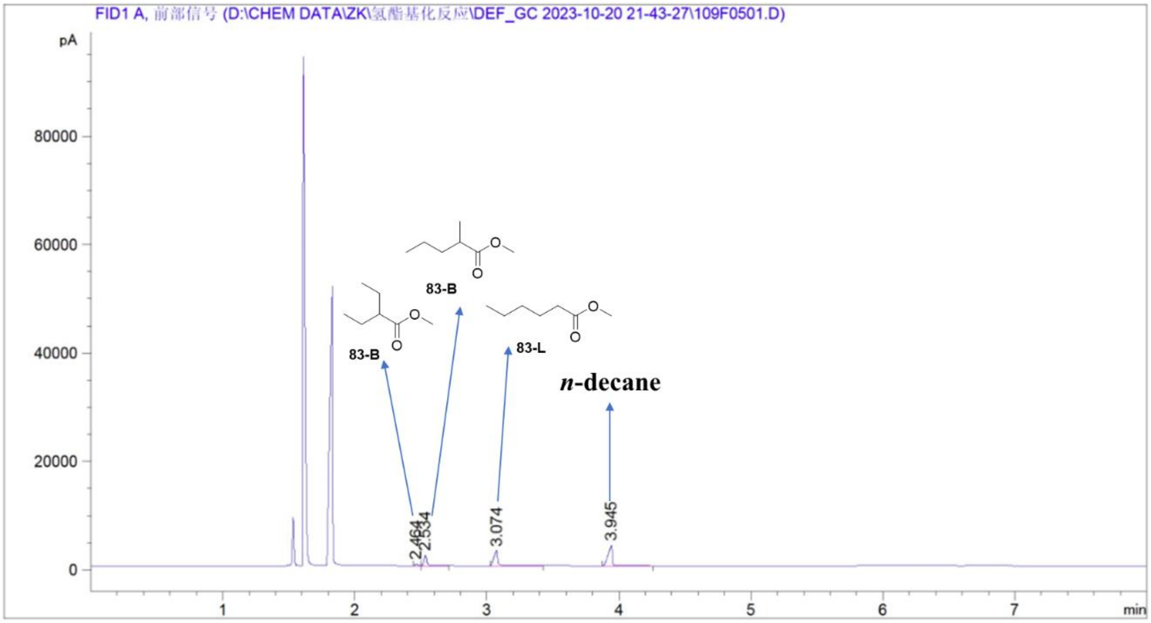

Supplementary Fig. 88 GC spectrum of product 83.

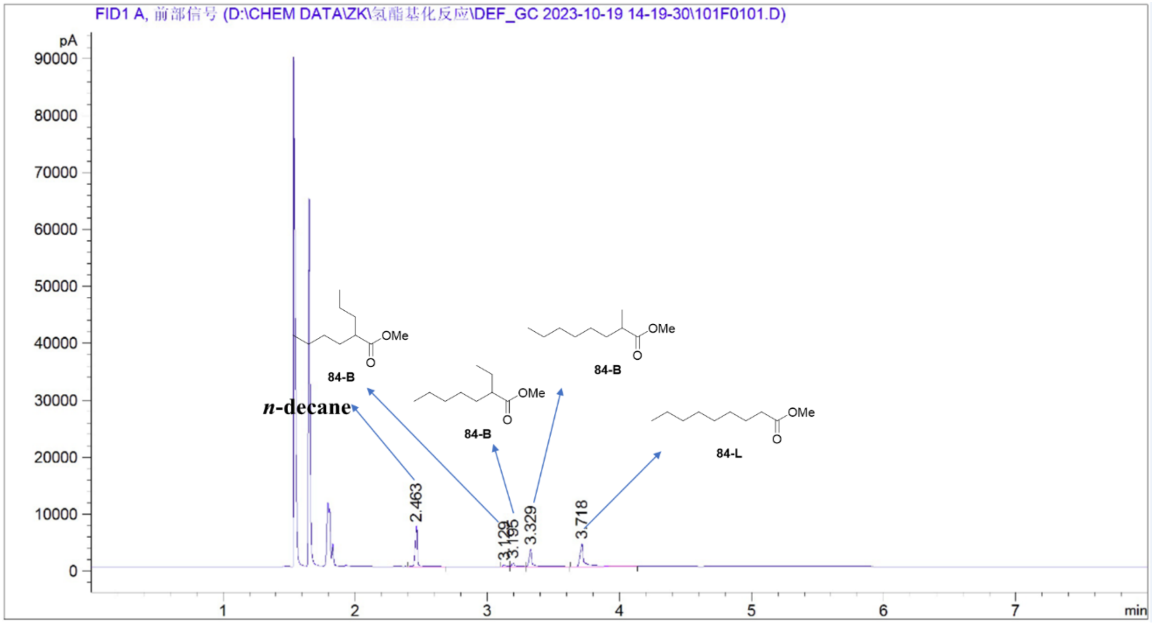

Supplementary Fig. 89 GC spectrum of product 84.

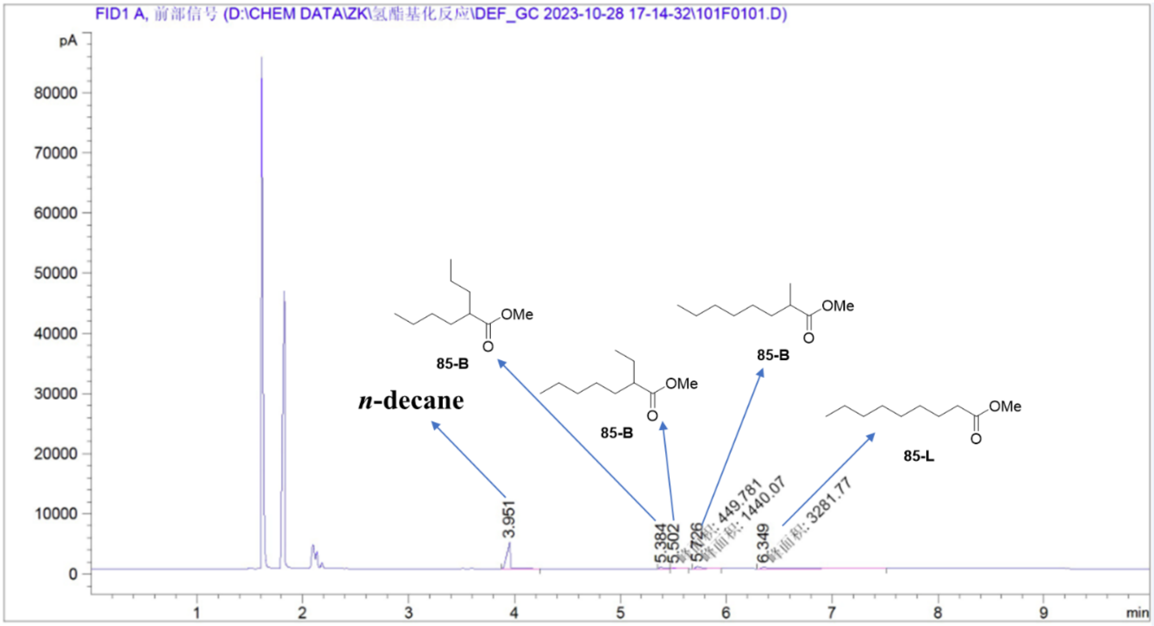

Supplementary Fig. 90 GC spectrum of product 85.

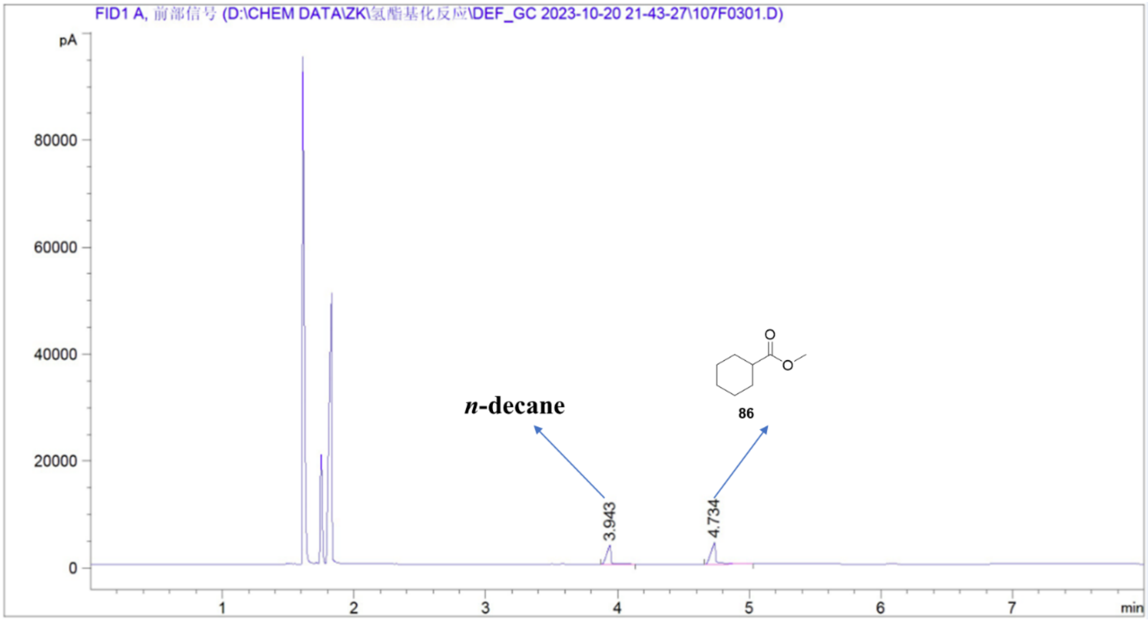

Supplementary Fig. 91 GC spectrum of product 86.

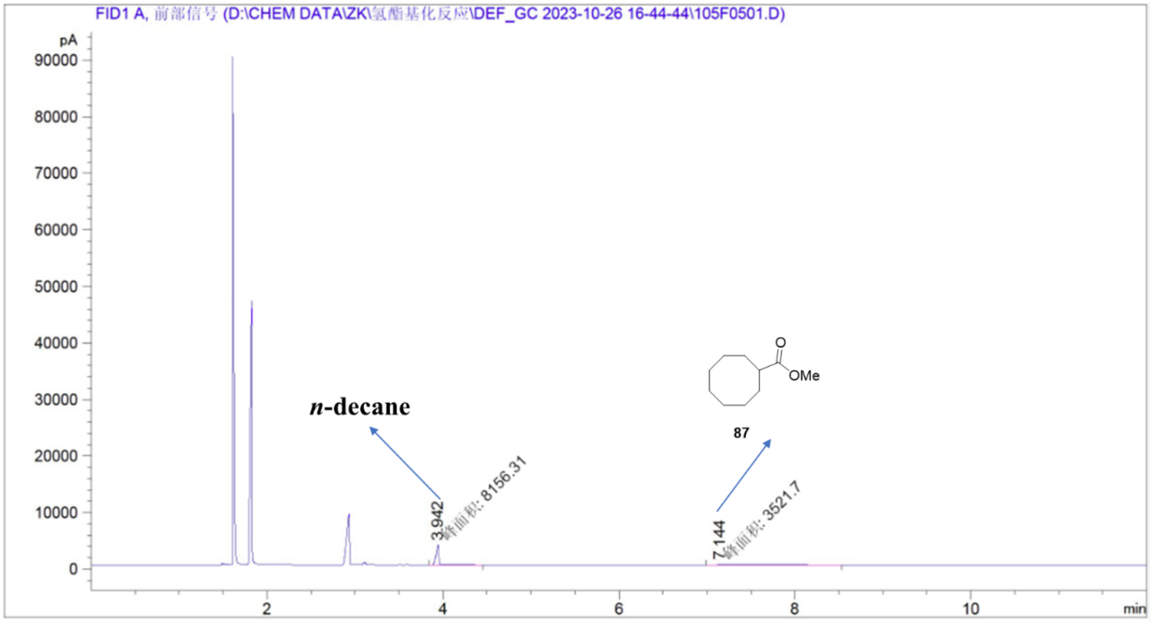

Supplementary Fig. 92 GC spectrum of product 87.

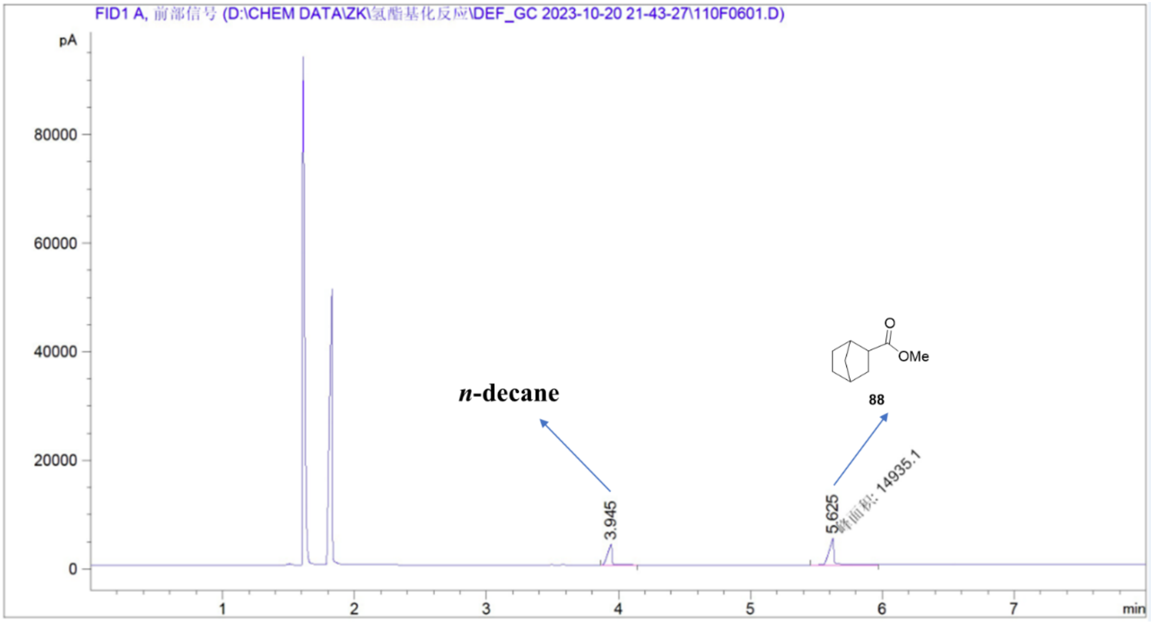

Supplementary Fig. 93 GC spectrum of product 88.

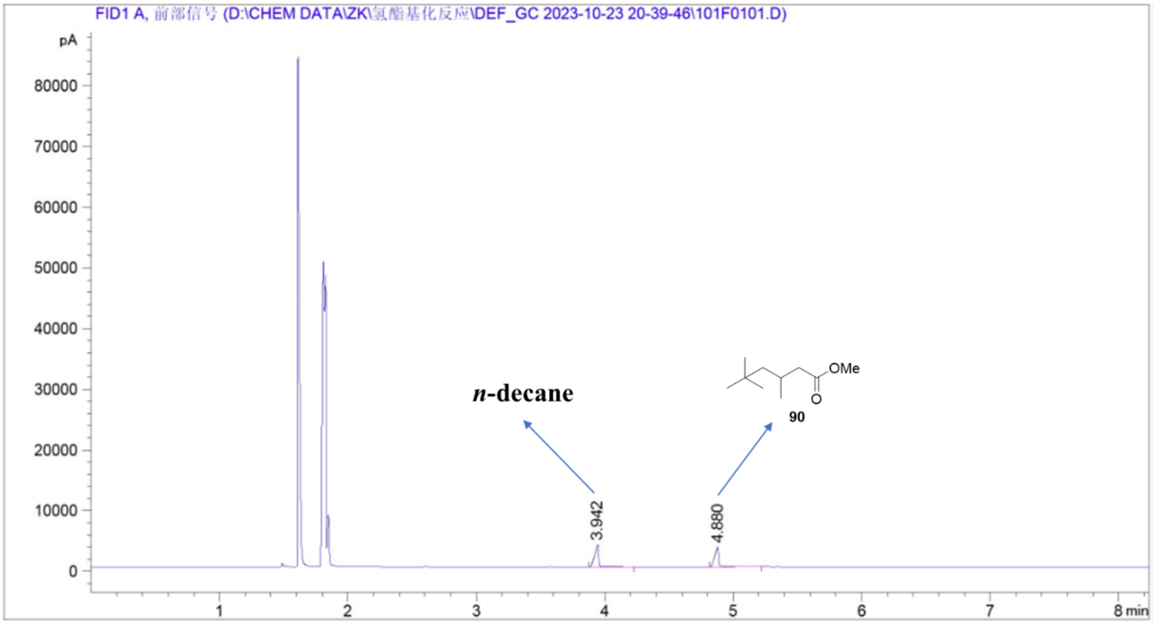

Supplementary Fig. 94 GC spectrum of product 90.

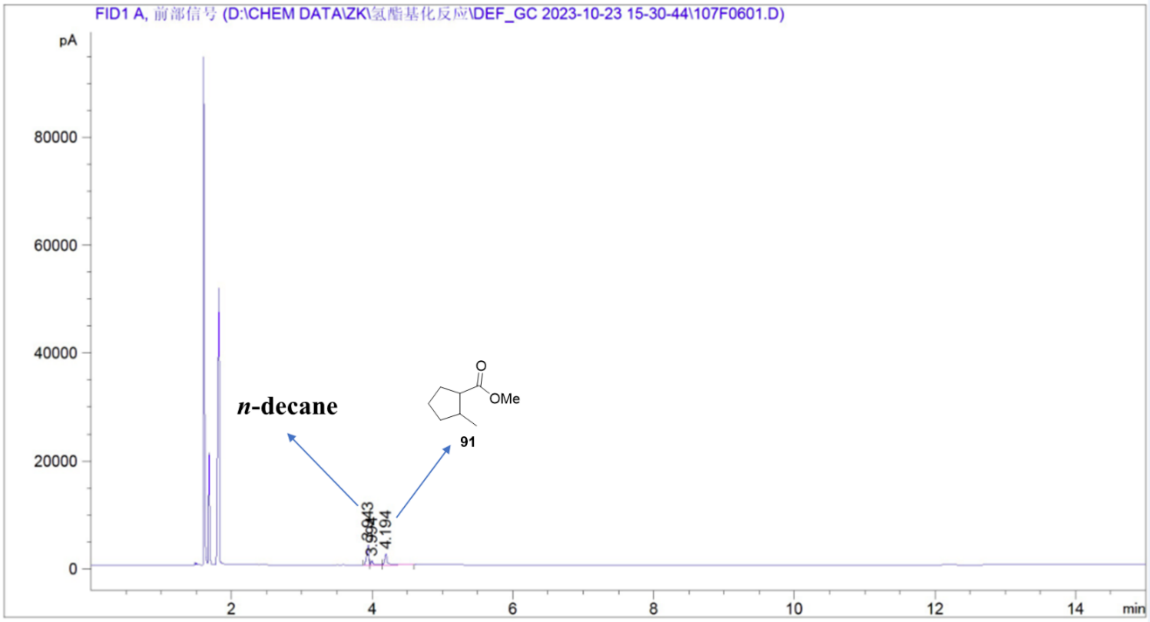

Supplementary Fig. 95 GC spectrum of product 91.

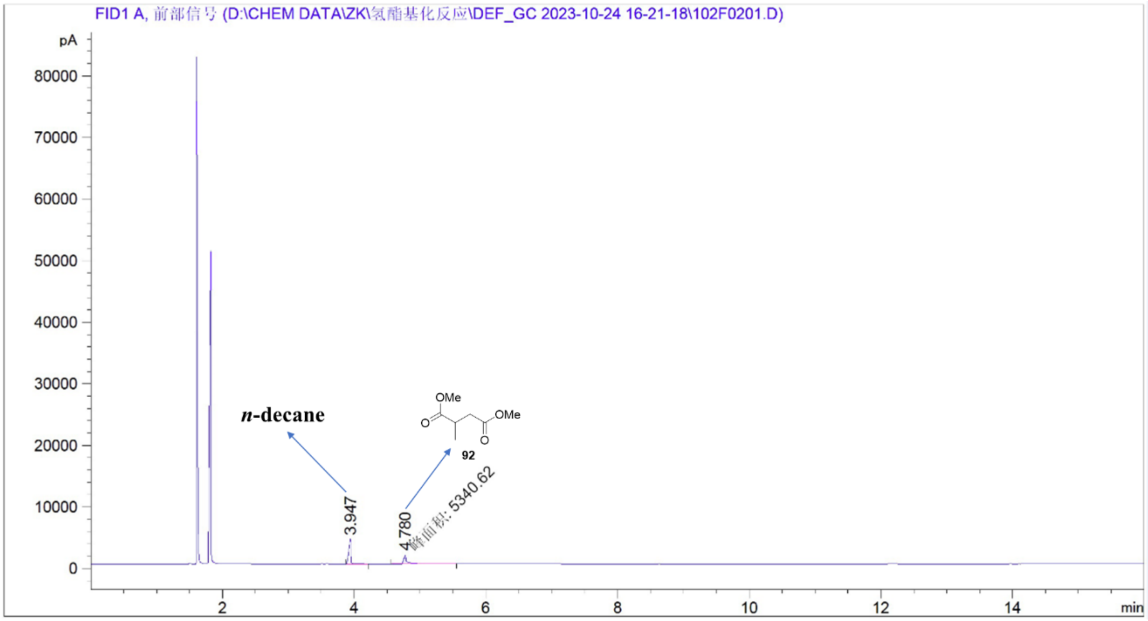

Supplementary Fig. 96 GC spectrum of product 92.

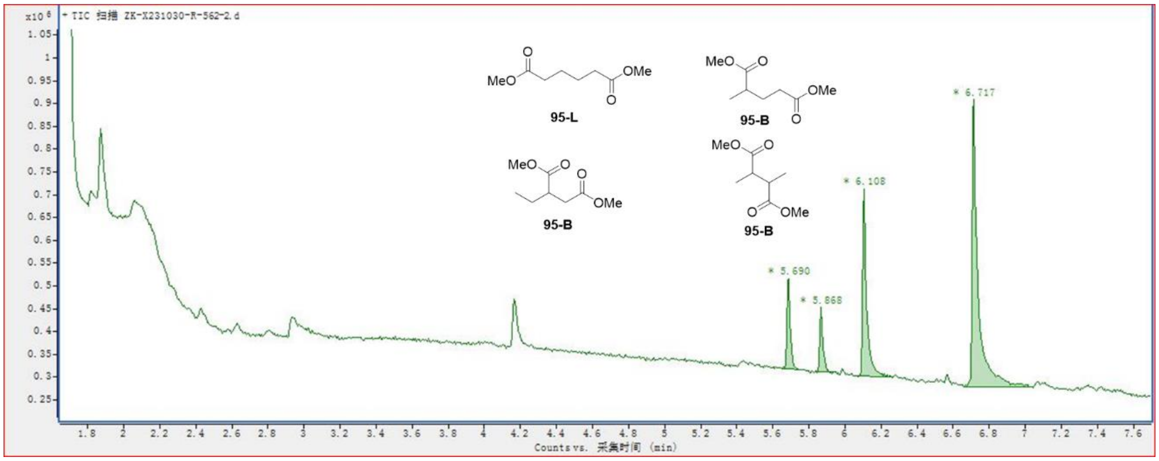

Supplementary Fig. 97 GC spectrum of product 95.

Quantitative NMR spectra of the products

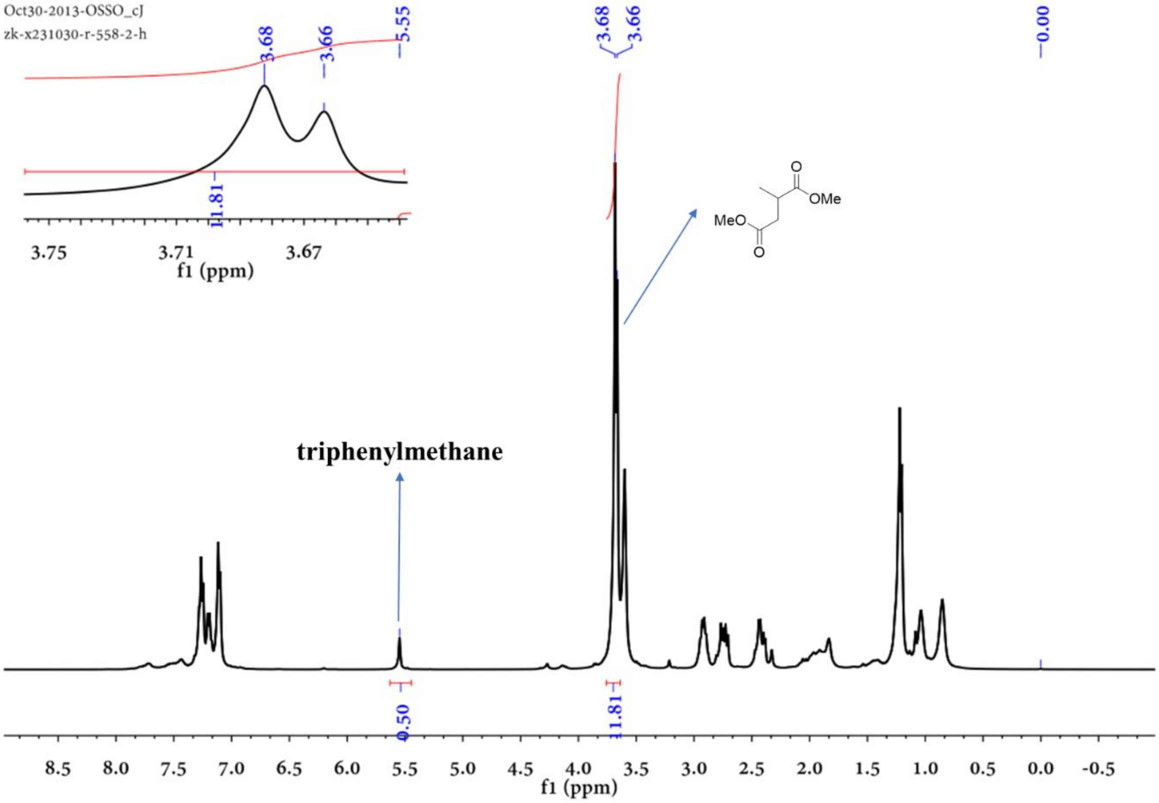

Supplementary Fig. 98 ^{1}H NMR (400 MHz, 20 ºC) spectrum of product 92 in $CDCl_3$.

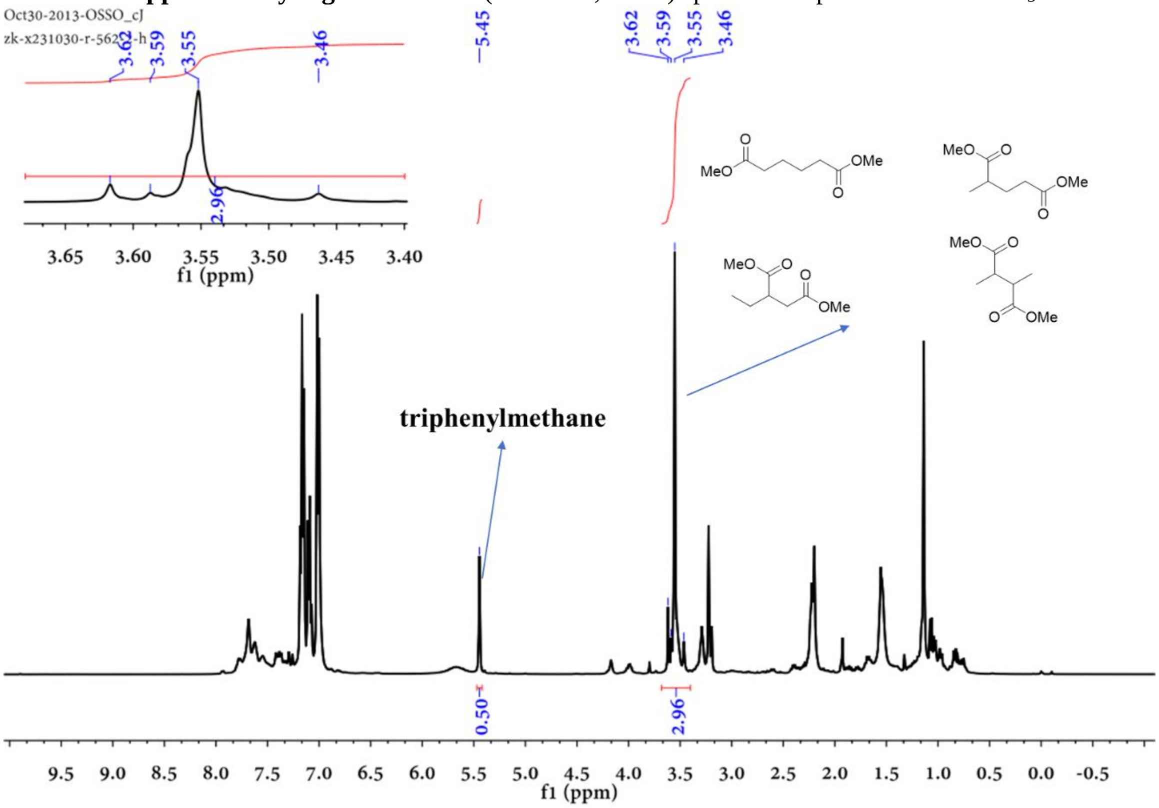

Supplementary Fig. 99 ^{1}H NMR (400 MHz, 20 ºC) spectrum of product 95 in $CDCl_3$.

LC spectra of the products

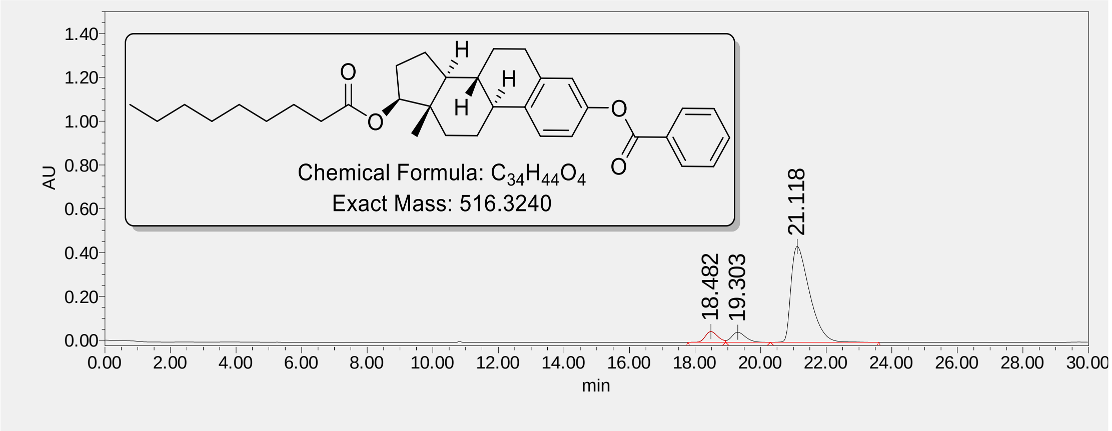

	Time	Area	Height	Area%
1	18.482	1276879	48969	6.45
2	19.303	1350900	45894	6.82
3	21.118	17166830	437929	86.72

Supplementary Fig. 100 LC spectrum of product 109.

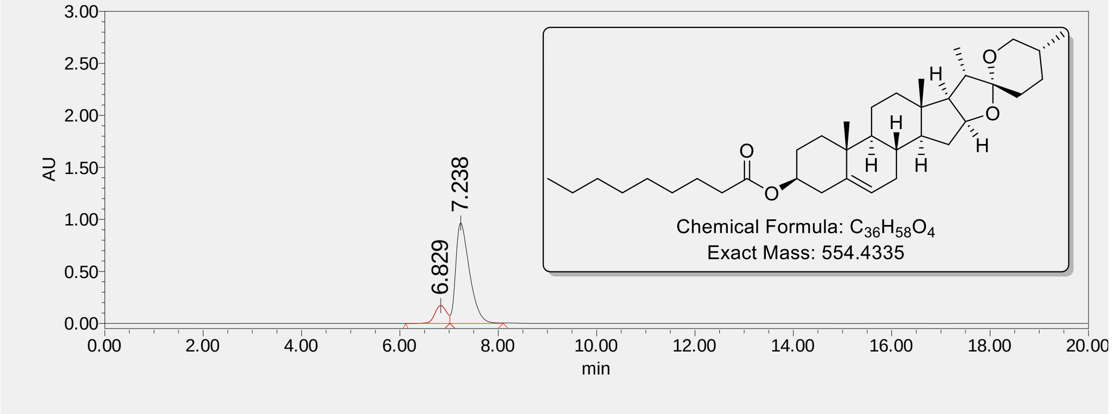

	Time	Area	Height	Area%
1	6.829	2823380	171697	13.01
2	7.238	18870064	968375	86.99

Supplementary Fig. 101 LC spectrum of product 111.

NMR spectra of the products

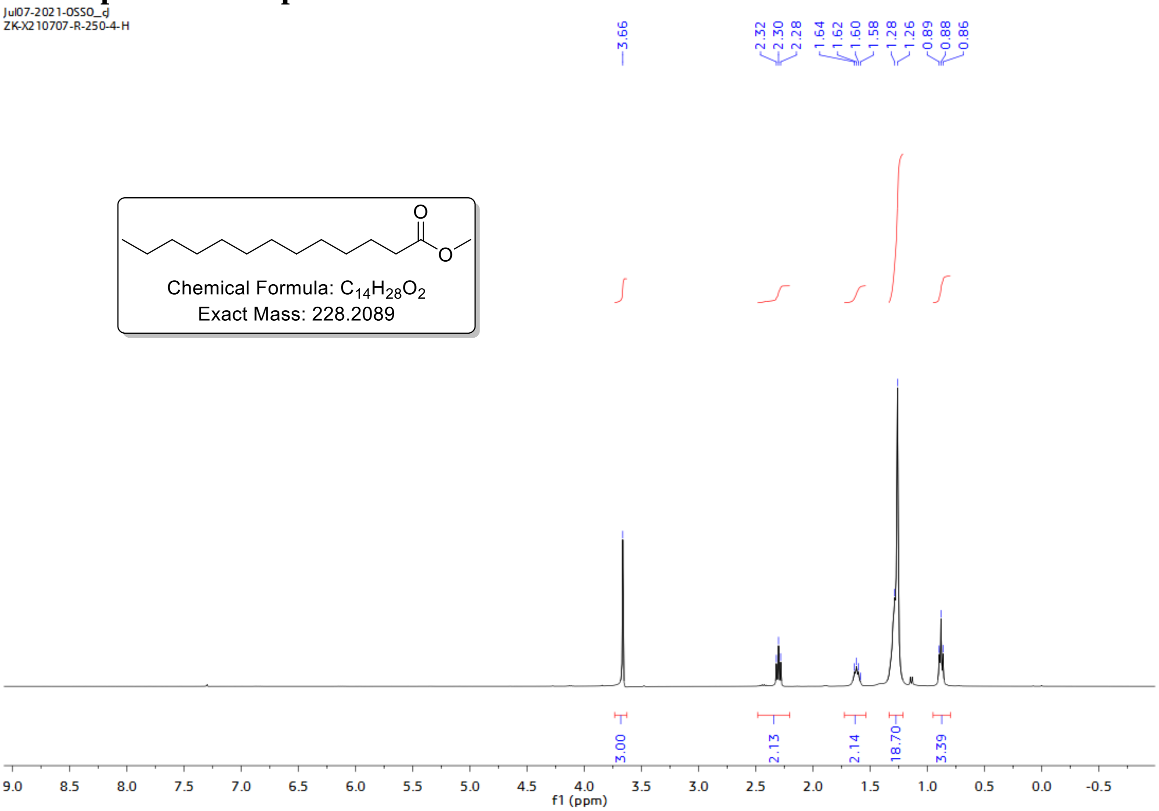

Supplementary Fig. 102 ^{1}H NMR (400 MHz, 20 °C) spectrum of product 5 in $CDCl_3$.

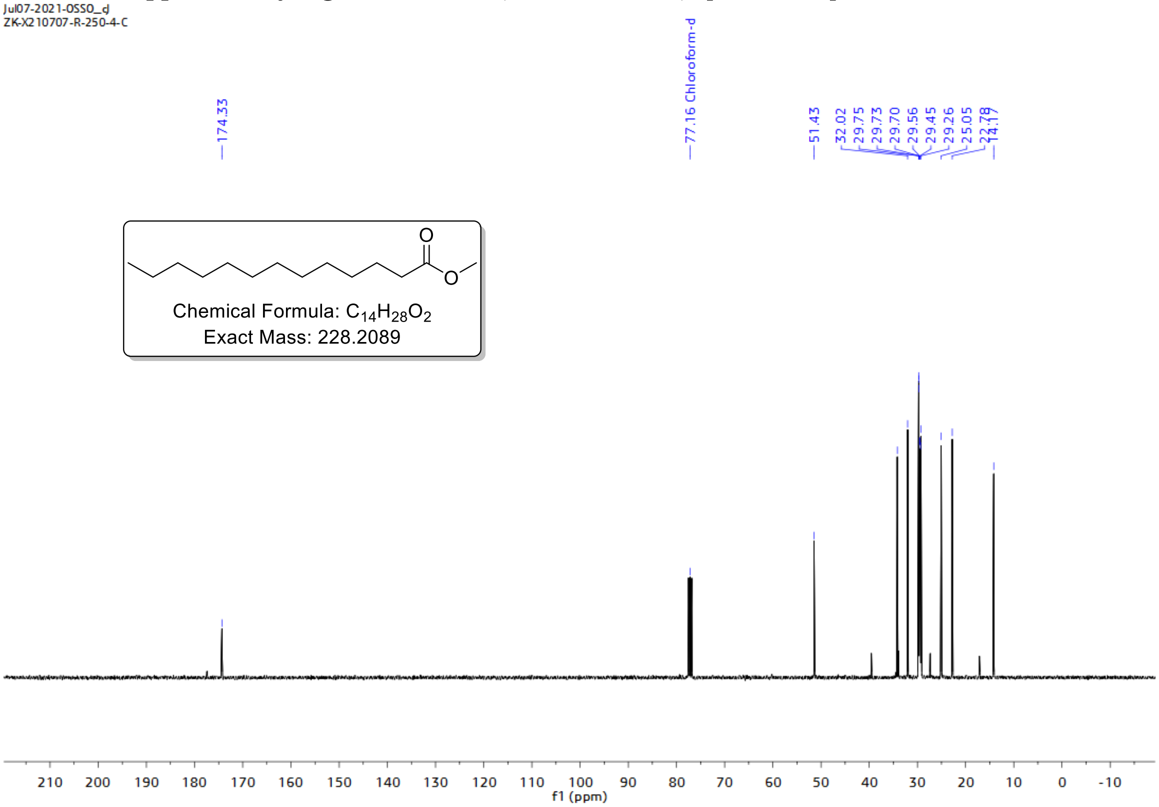

Supplementary Fig. 103 ^{13}C NMR (101 MHz, 20 °C) spectrum of product 5 in $CDCl_3$.

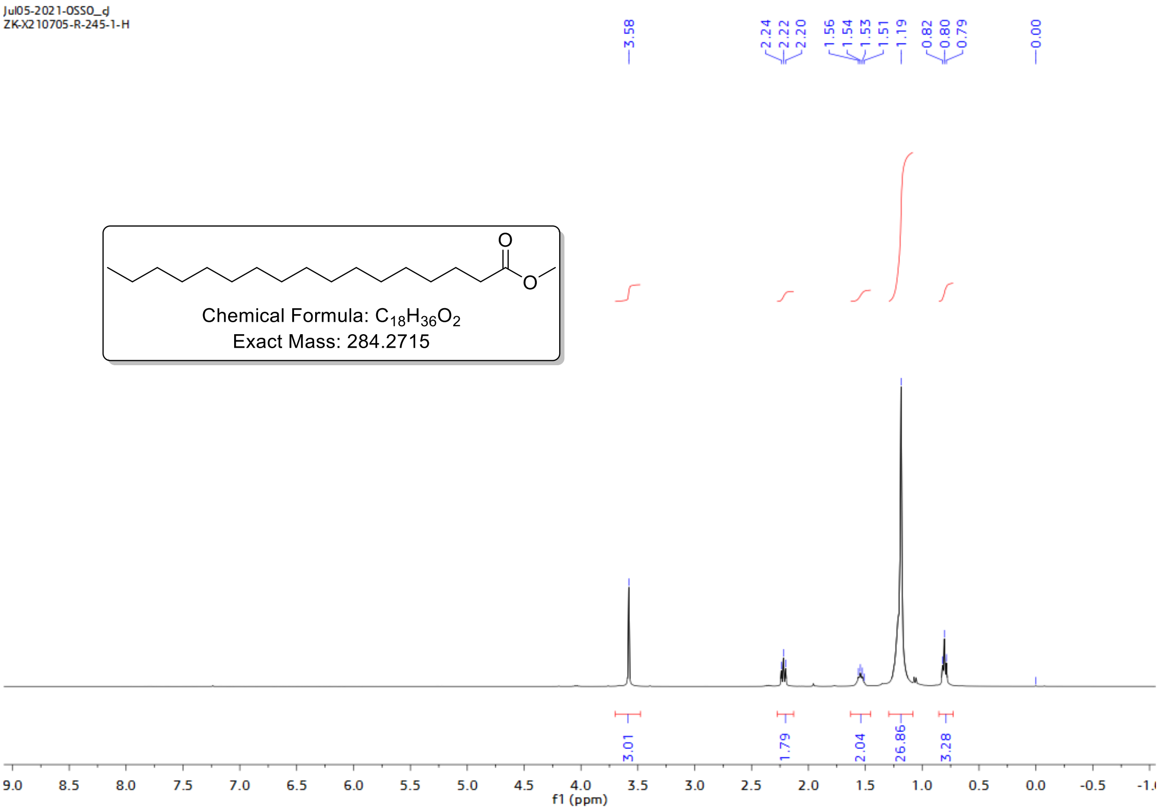

Supplementary Fig. 104 ^{1}H NMR (400 MHz, 20 °C) spectrum of product 6 in $CDCl_3$.

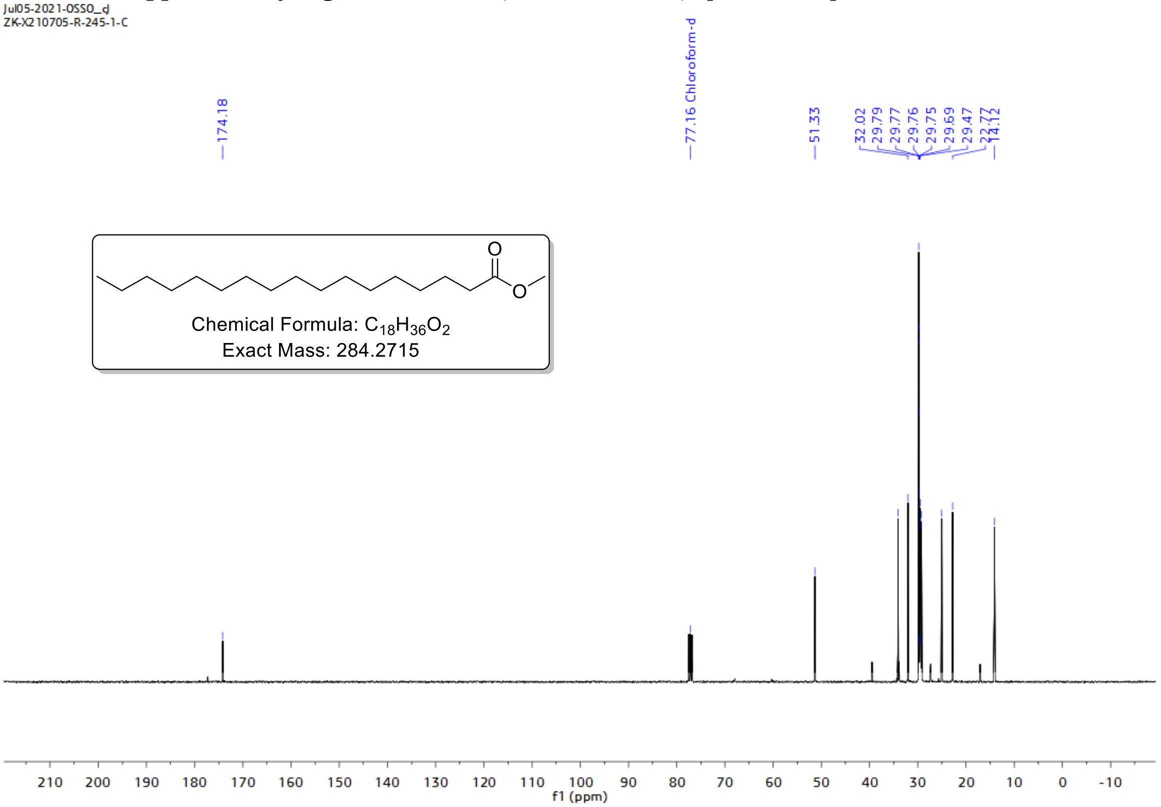

Supplementary Fig. 105 ^{13}C NMR (101 MHz, 20 °C) spectrum of product 6 in $CDCl_3$.

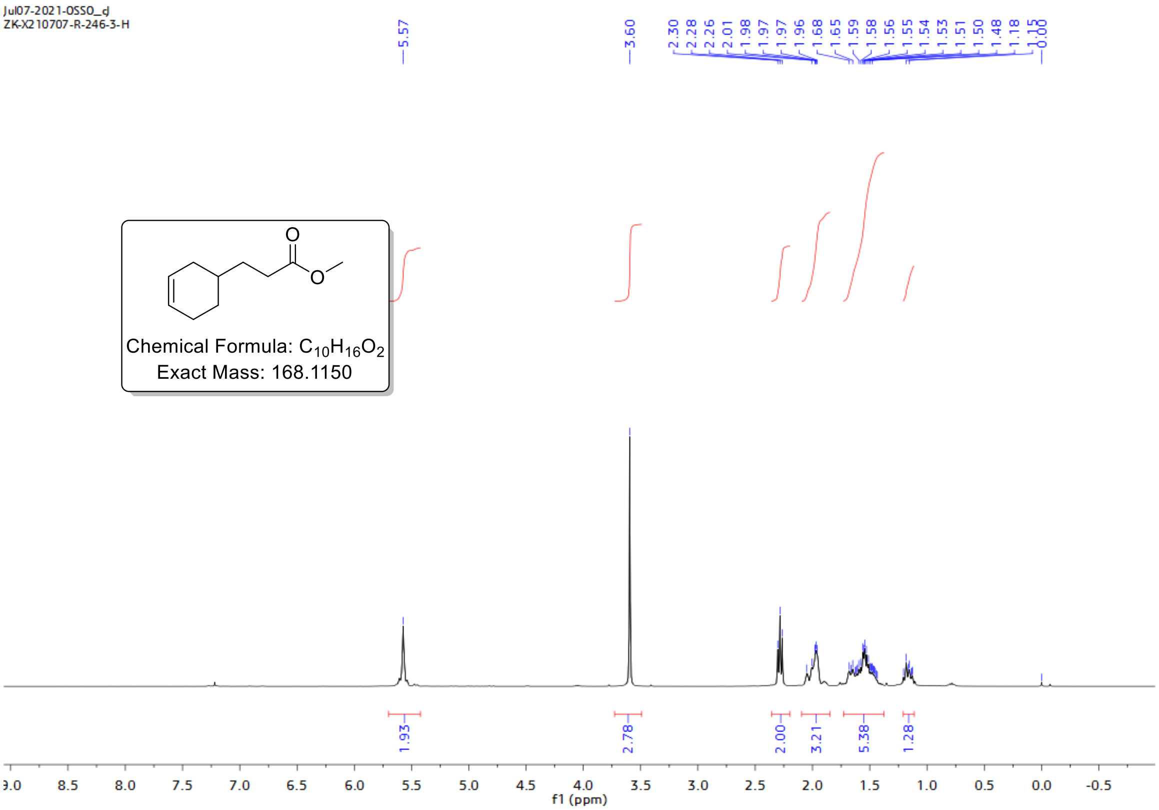

Supplementary Fig. 106 ^{1}H NMR (400 MHz, 20 °C) spectrum of product 9 in $CDCl_3$.

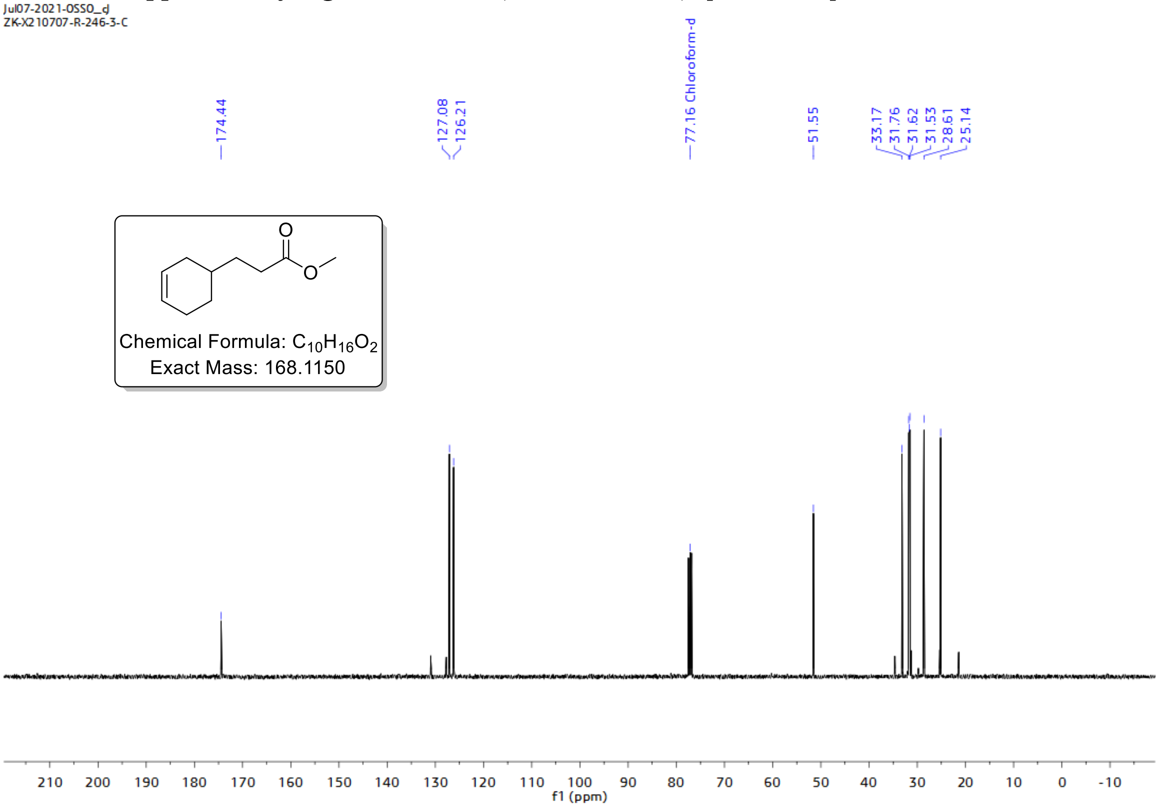

Supplementary Fig. 107 ^{13}C NMR (101 MHz, 20 °C) spectrum of product 9 in $CDCl_3$.

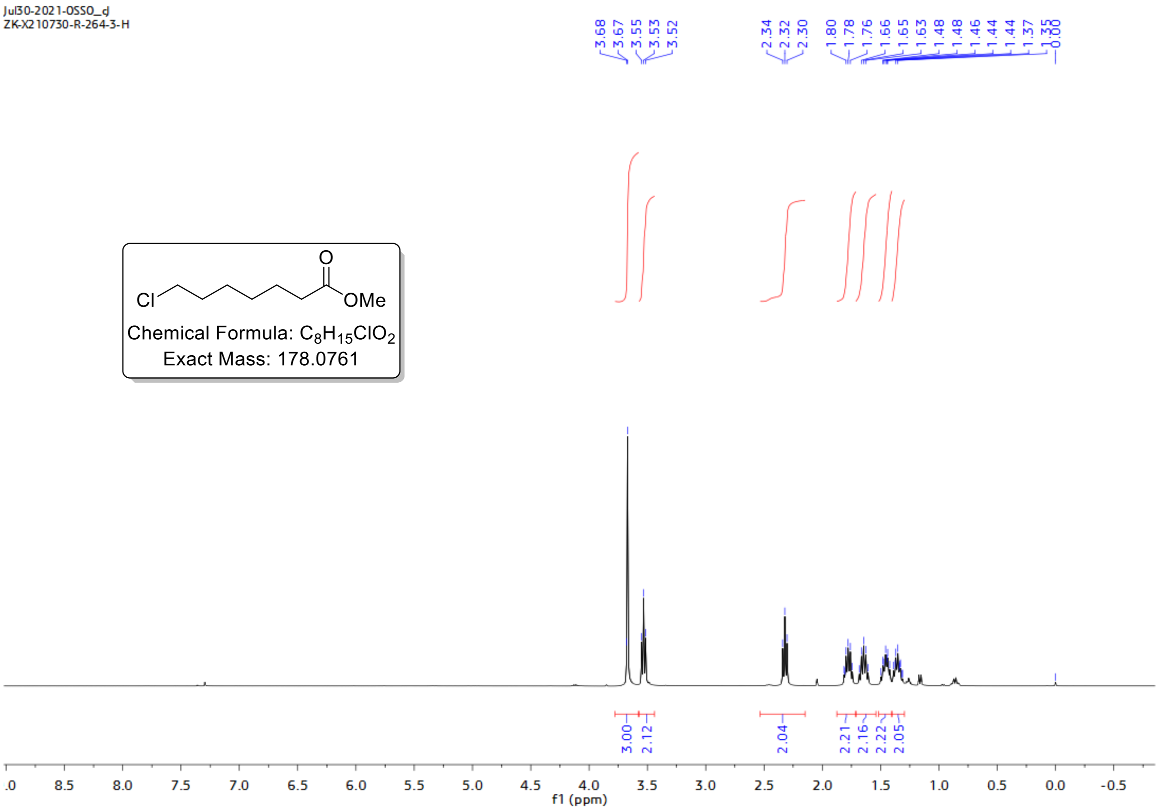

Supplementary Fig. 108 ^{1}H NMR (400 MHz, 20 ºC) spectrum of product 10 in $CDCl_3$.

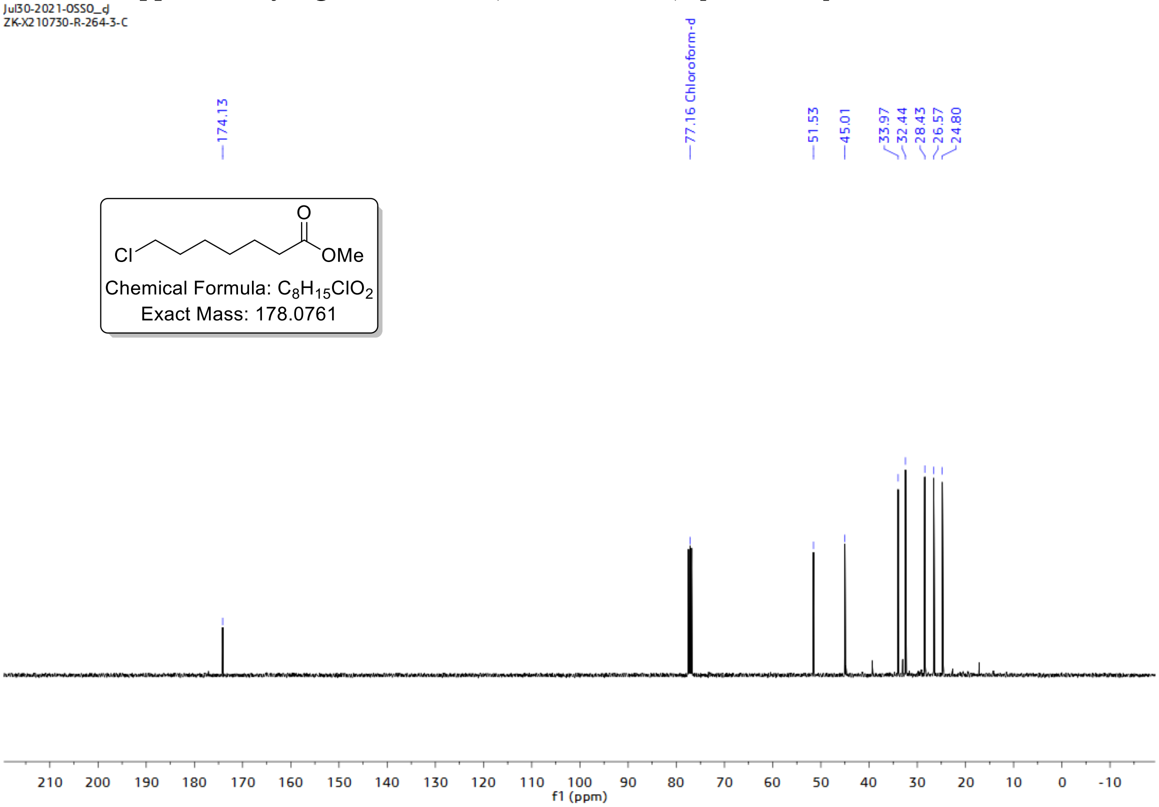

Supplementary Fig. 109 ^{13}C NMR (101 MHz, 20 ºC) spectrum of product 10 in $CDCl_3$.

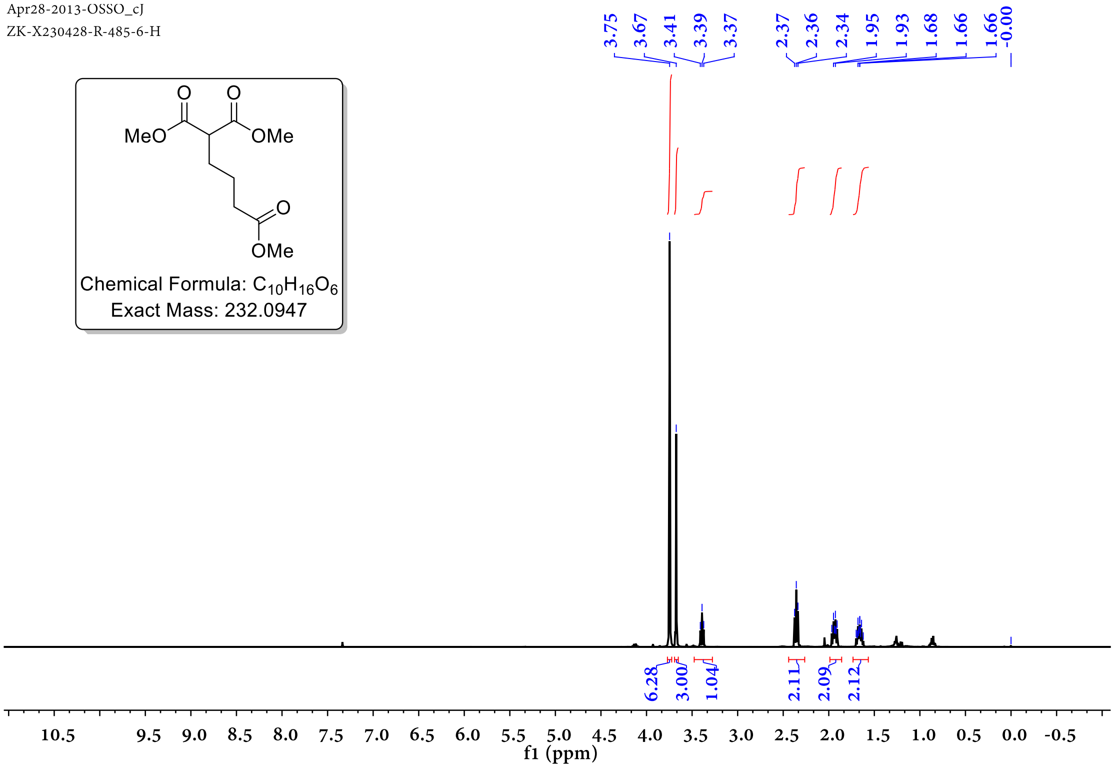

Supplementary Fig. 110 ^{1}H NMR (400 MHz, 20 ºC) spectrum of product 12 in $CDCl_3$.

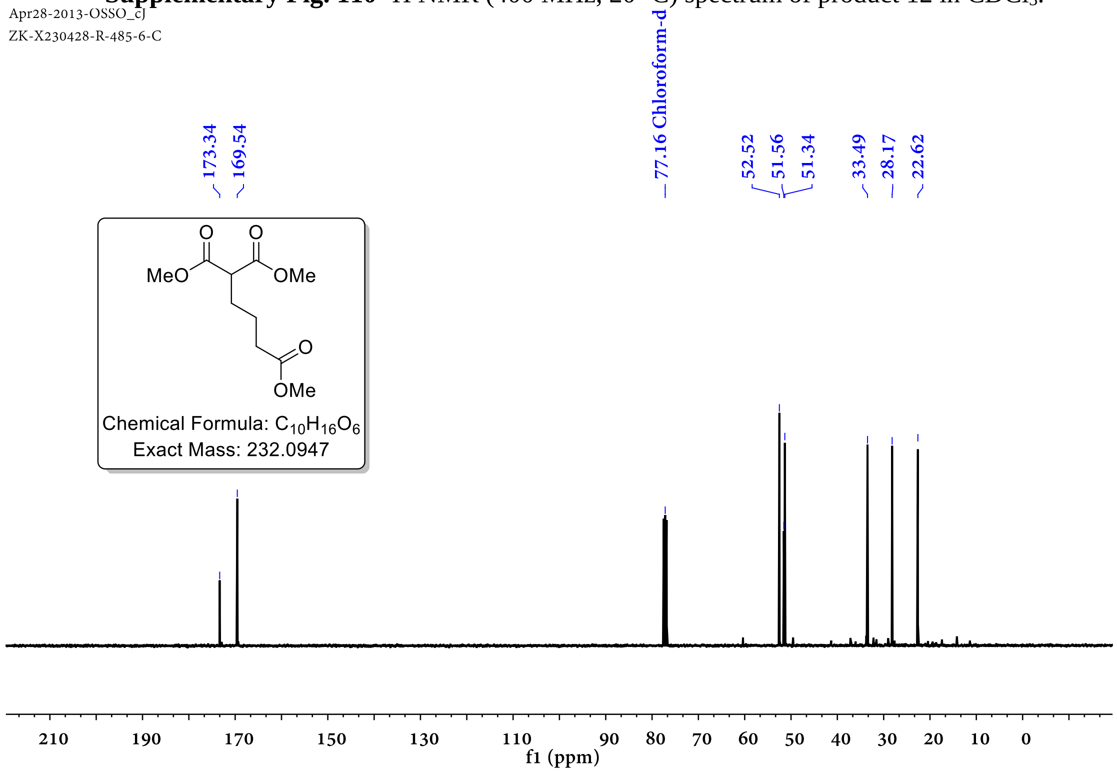

Supplementary Fig. 111 ^{13}C NMR (101 MHz, 20 ºC) spectrum of product 12 in $CDCl_3$.

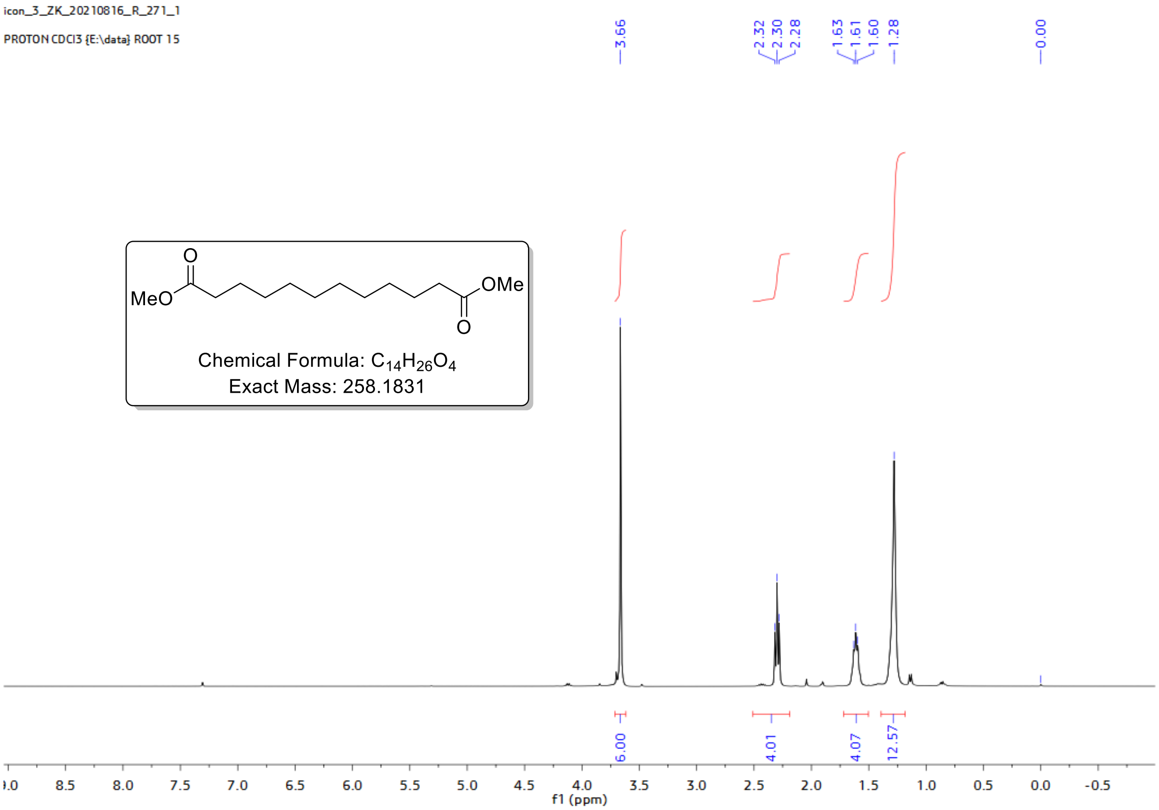

Supplementary Fig. 112 ^{1}H NMR (400 MHz, 20 ºC) spectrum of product 13 in $CDCl_3$.

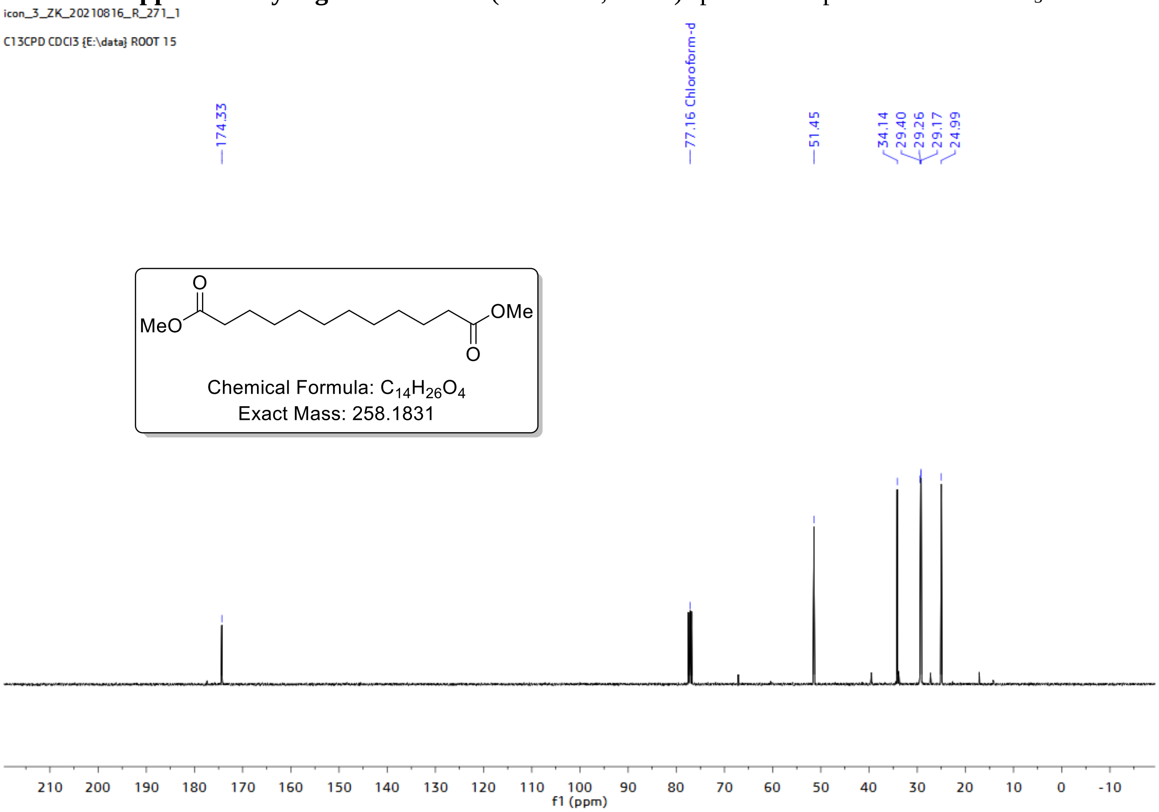

Supplementary Fig. 113 ^{13}C NMR (101 MHz, 20 ºC) spectrum of product 13 in $CDCl_3$.

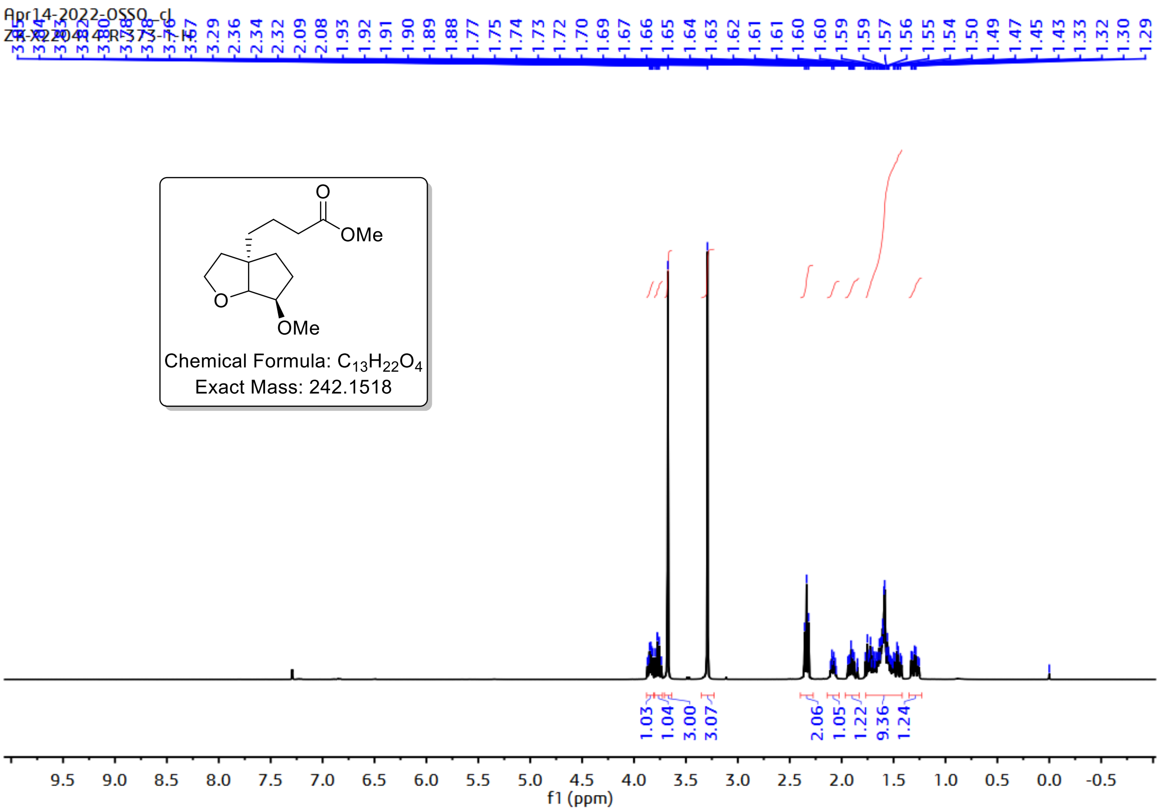

Supplementary Fig. 114 ^{1}H NMR (400 MHz, 20 ºC) spectrum of product 14 in $CDCl_3$.

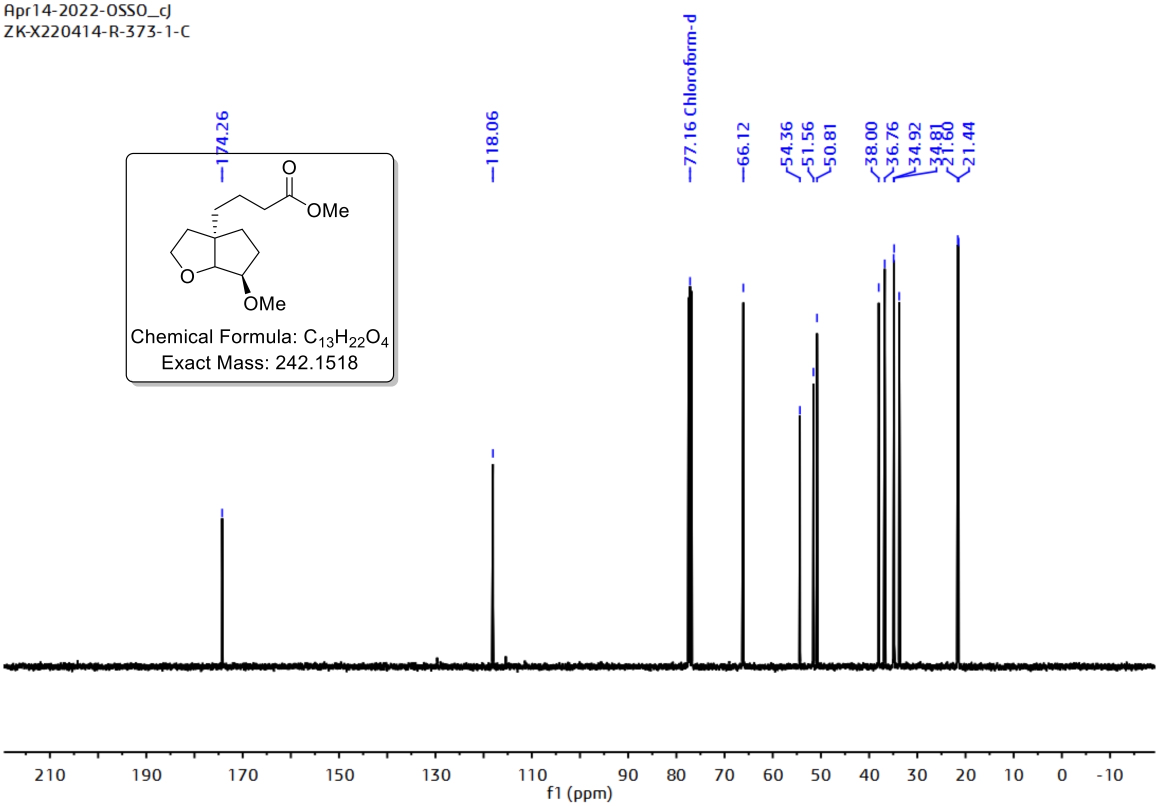

Supplementary Fig. 115 ^{13}C NMR (101 MHz, 20 ºC) spectrum of product 14 in $CDCl_3$.

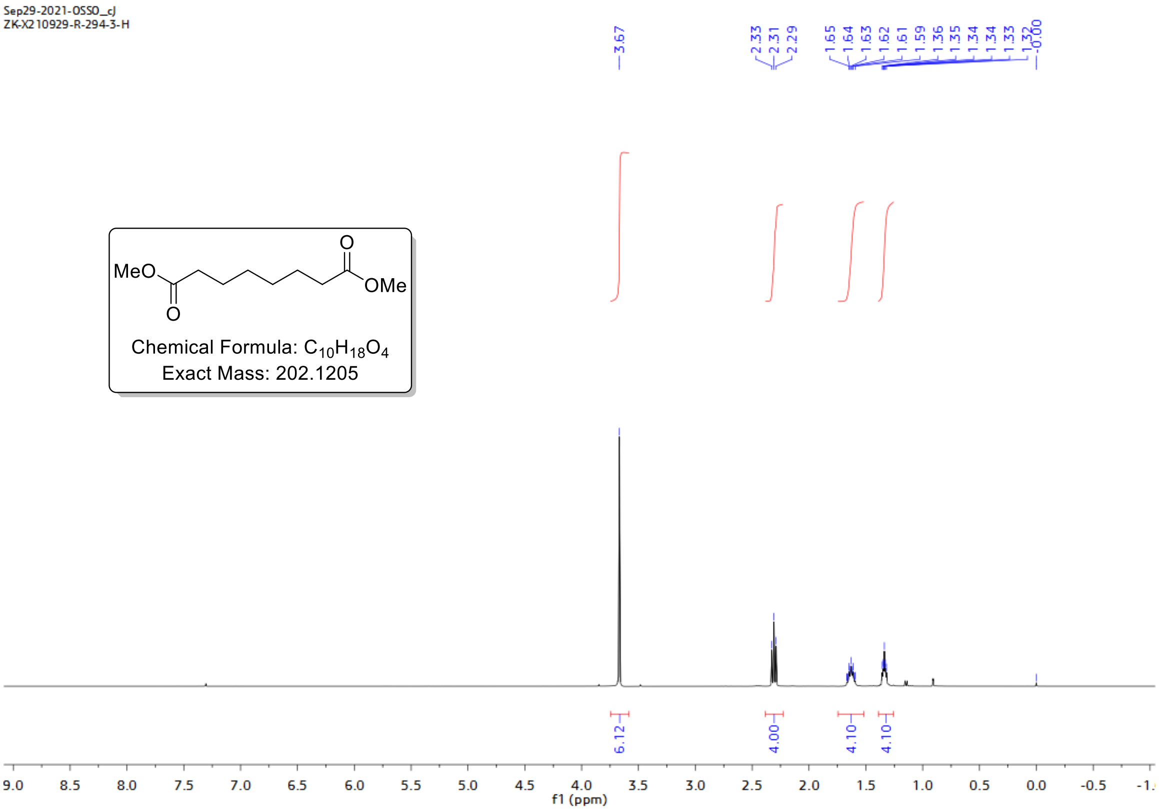

Supplementary Fig. 116 ^{1}H NMR (400 MHz, 20 ºC) spectrum of product 15 in $CDCl_3$.

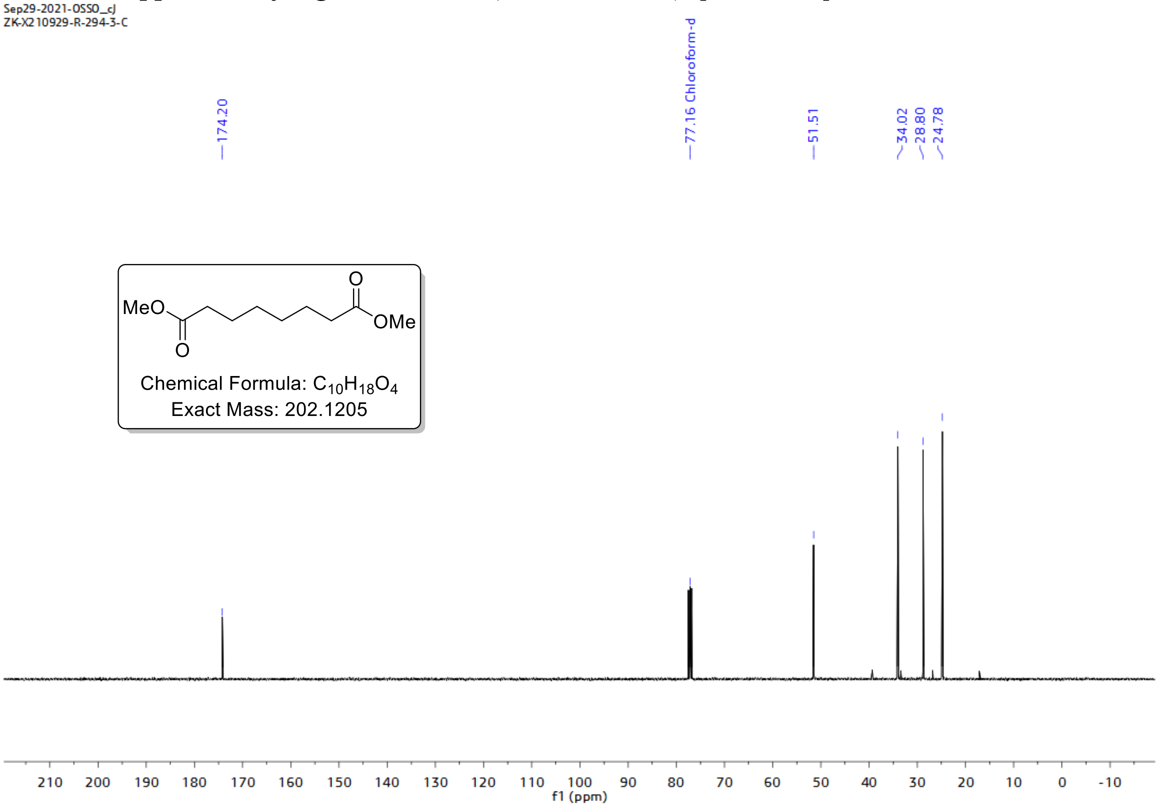

Supplementary Fig. 117 ^{13}C NMR (101 MHz, 20 ºC) spectrum of product 15 in $CDCl_3$.

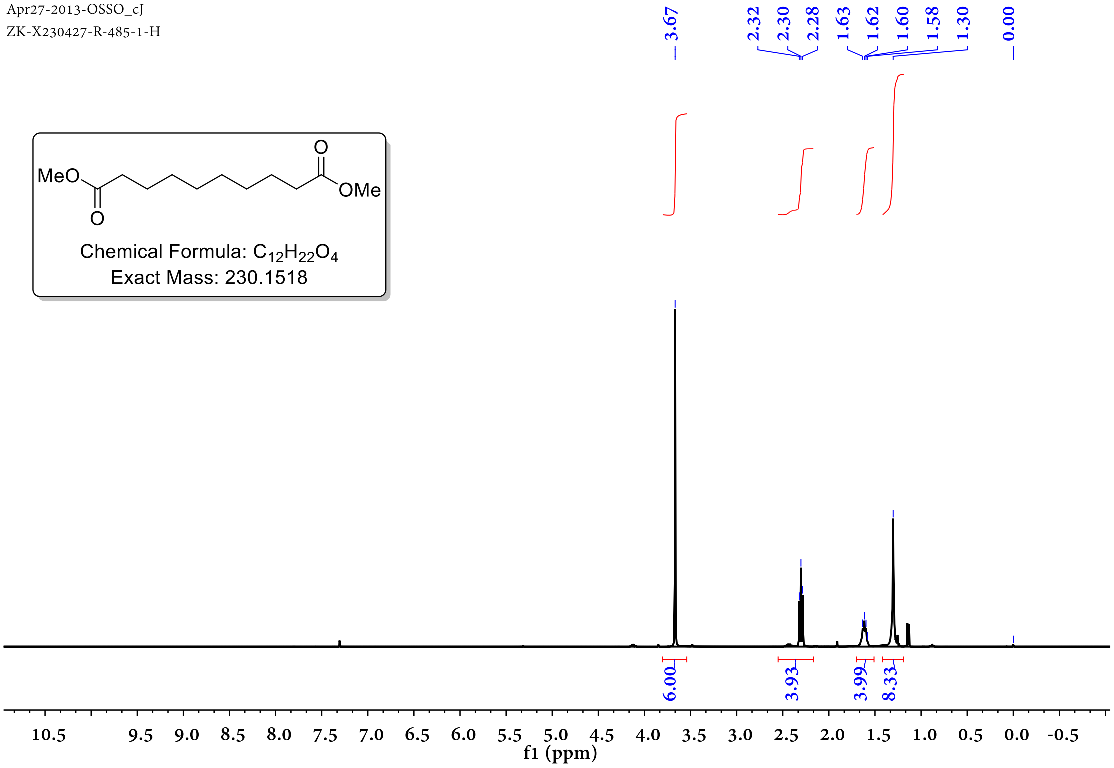

Supplementary Fig. 118 ^{1}H NMR (400 MHz, 20 °C) spectrum of product 16 in $CDCl_3$.

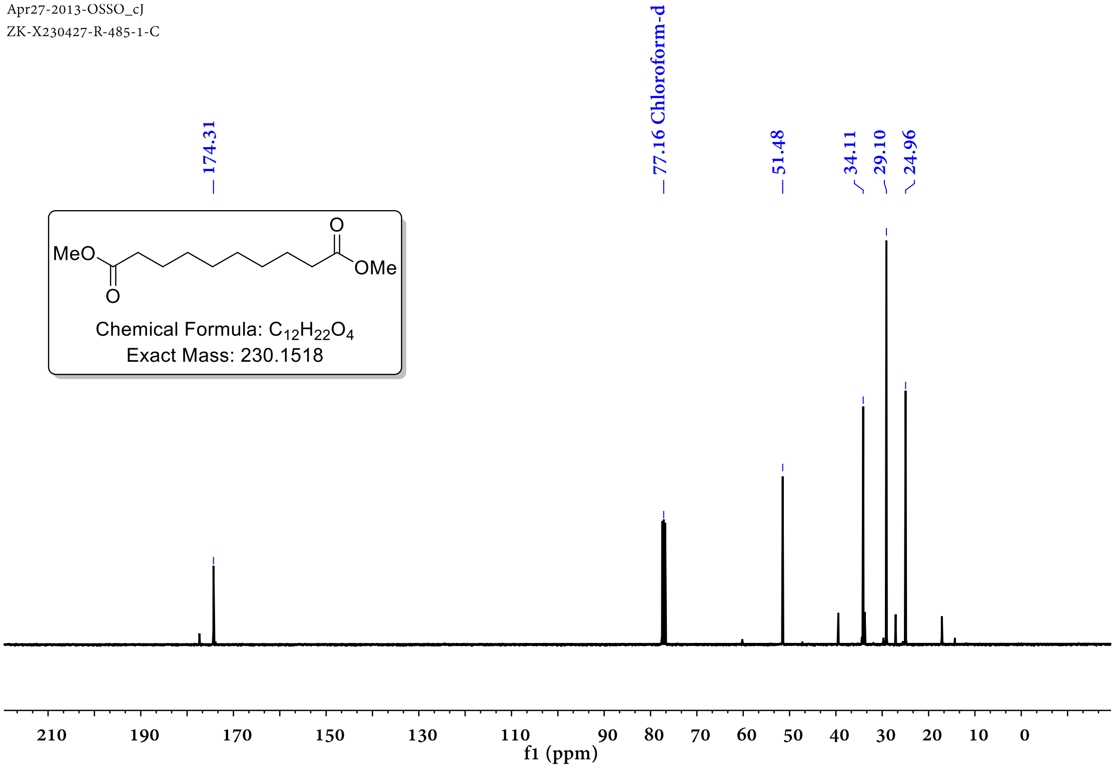

Supplementary Fig. 119 ^{13}C NMR (101 MHz, 20 °C) spectrum of product 16 in $CDCl_3$.

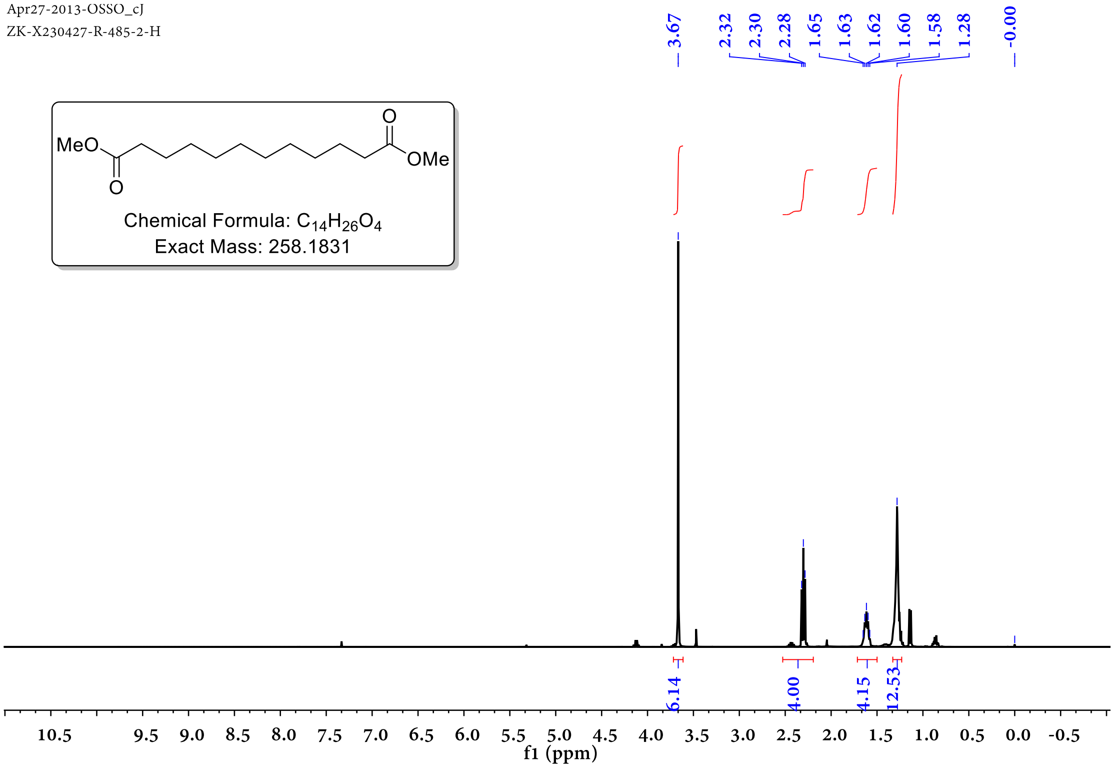

Supplementary Fig. 120 ^{1}H NMR (400 MHz, 20 ºC) spectrum of product 17 in $CDCl_3$.

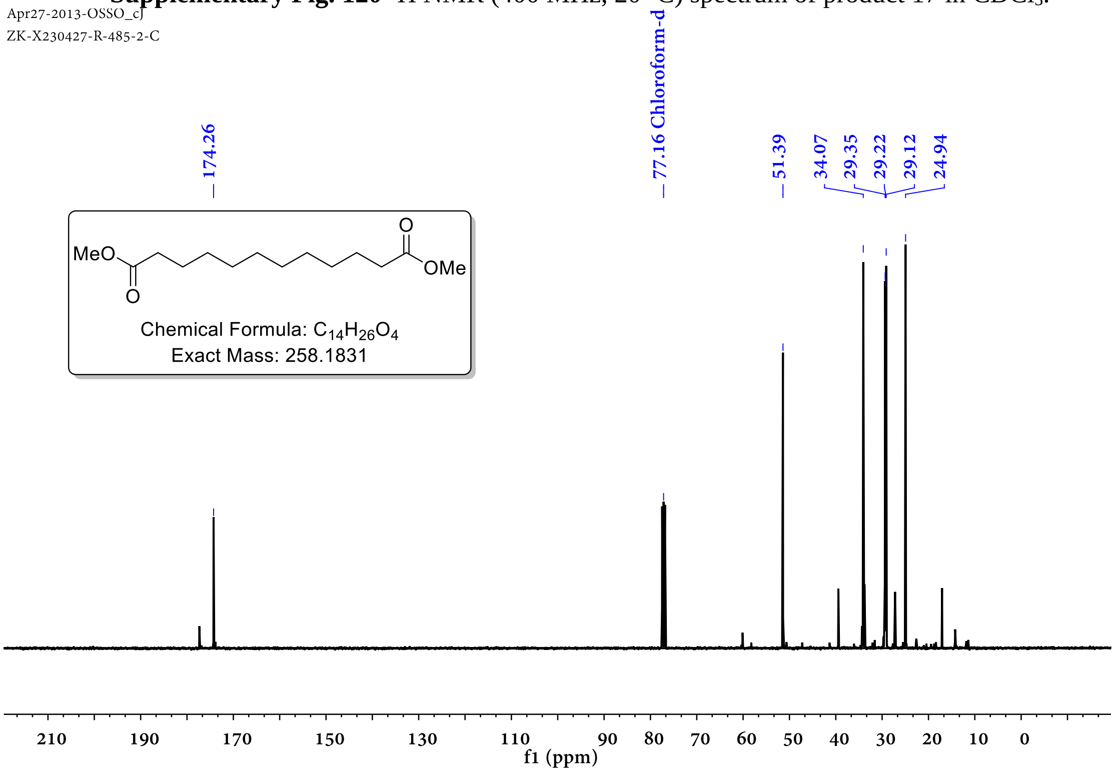

Supplementary Fig. 121 ^{13}C NMR (101 MHz, 20 ºC) spectrum of product 17 in $CDCl_3$.

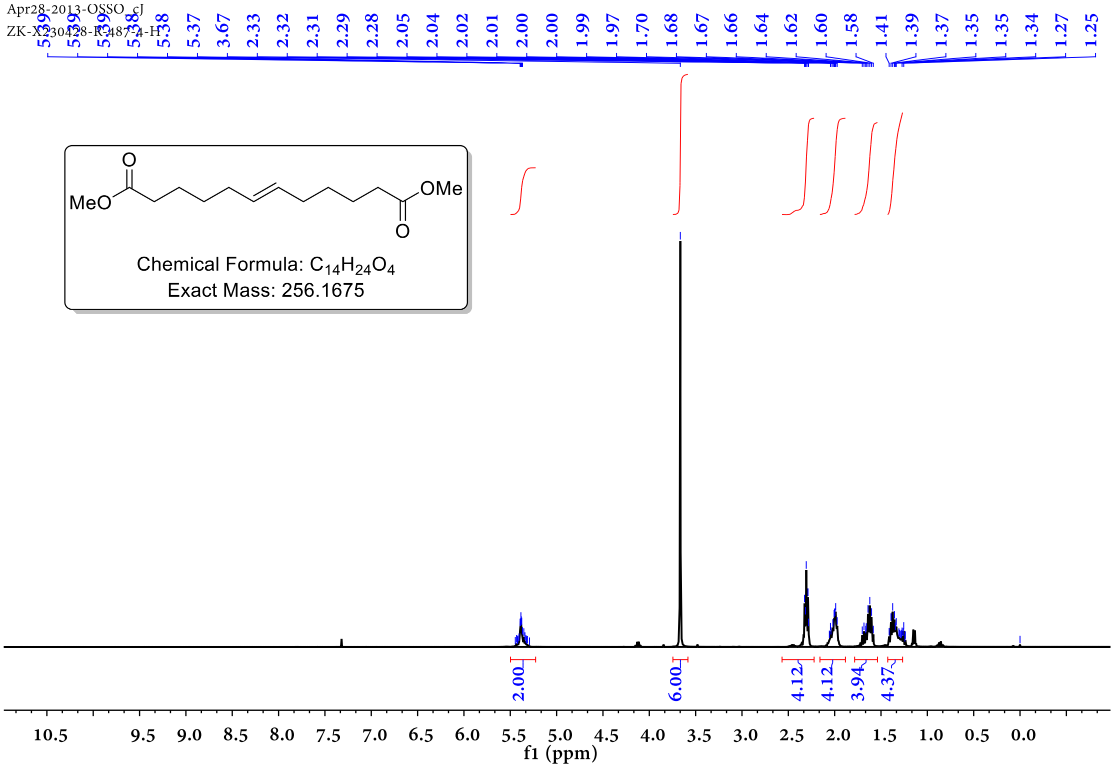

Supplementary Fig. 122 ^{1}H NMR (400 MHz, 20 ºC) spectrum of product 18 in $CDCl_3$.

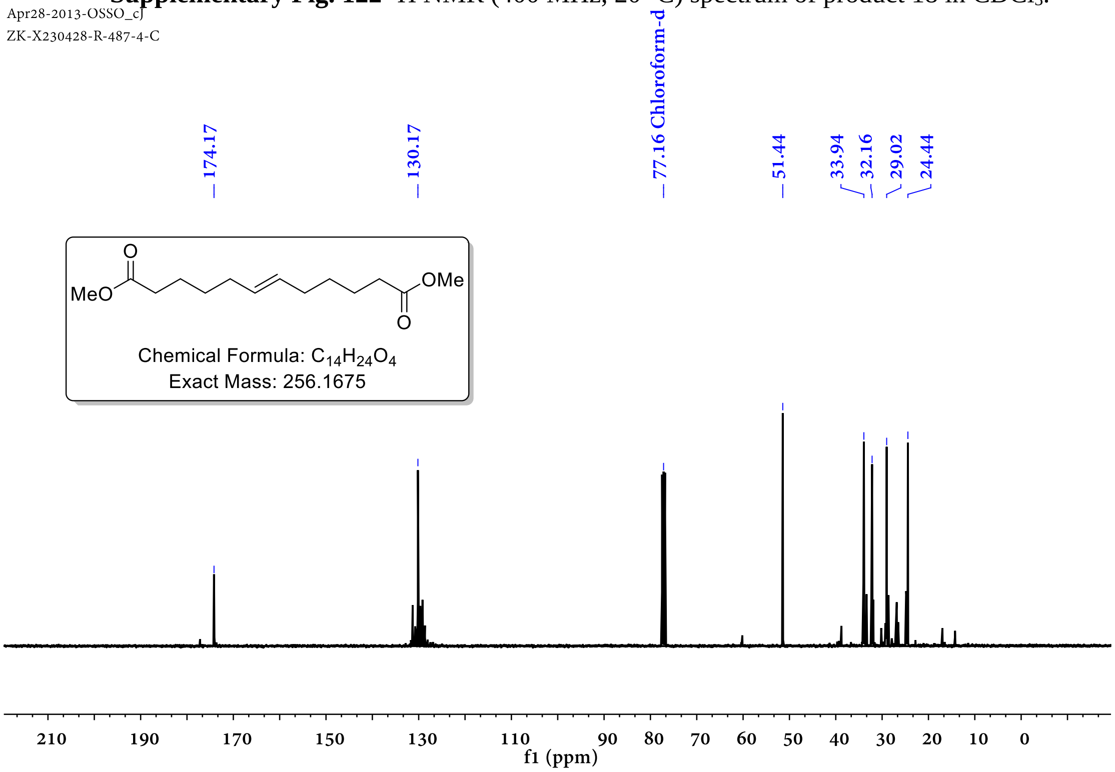

Supplementary Fig. 123 ^{13}C NMR (101 MHz, 20 ºC) spectrum of product 18 in $CDCl_3$.

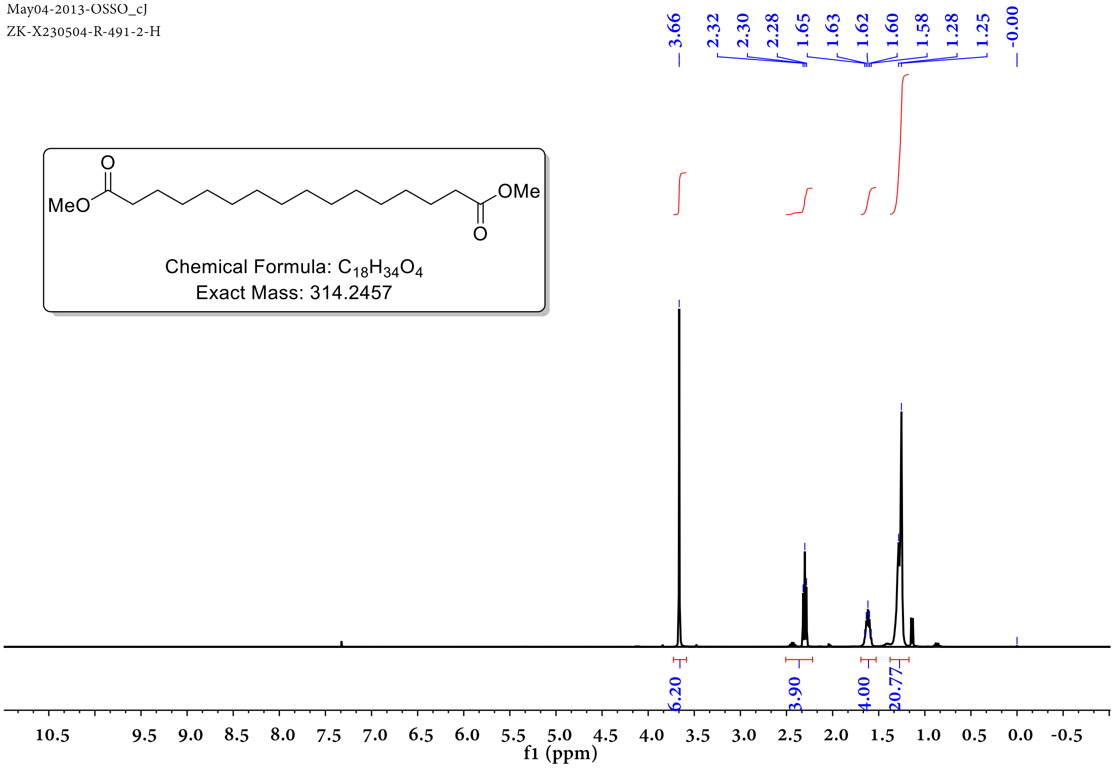

Supplementary Fig. 124 ^{1}H NMR (400 MHz, 20 ºC) spectrum of product 19 in $CDCl_3$.

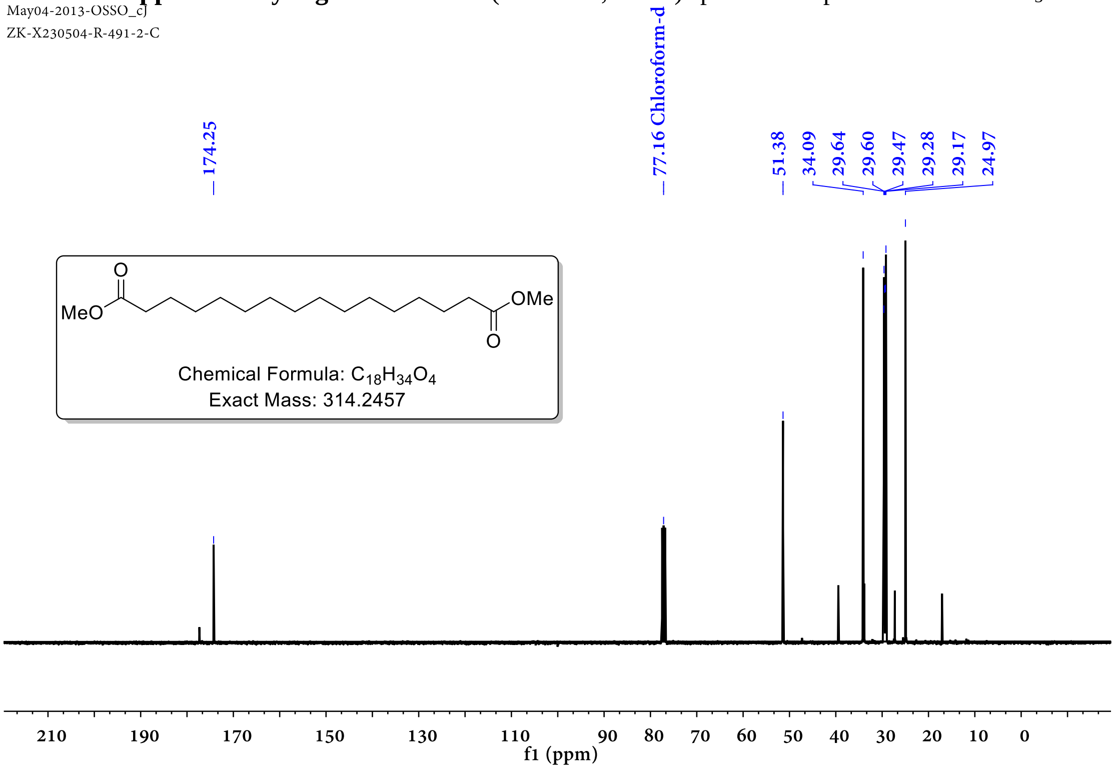

Supplementary Fig. 125 ^{13}C NMR (101 MHz, 20 ºC) spectrum of product 19 in $CDCl_3$.

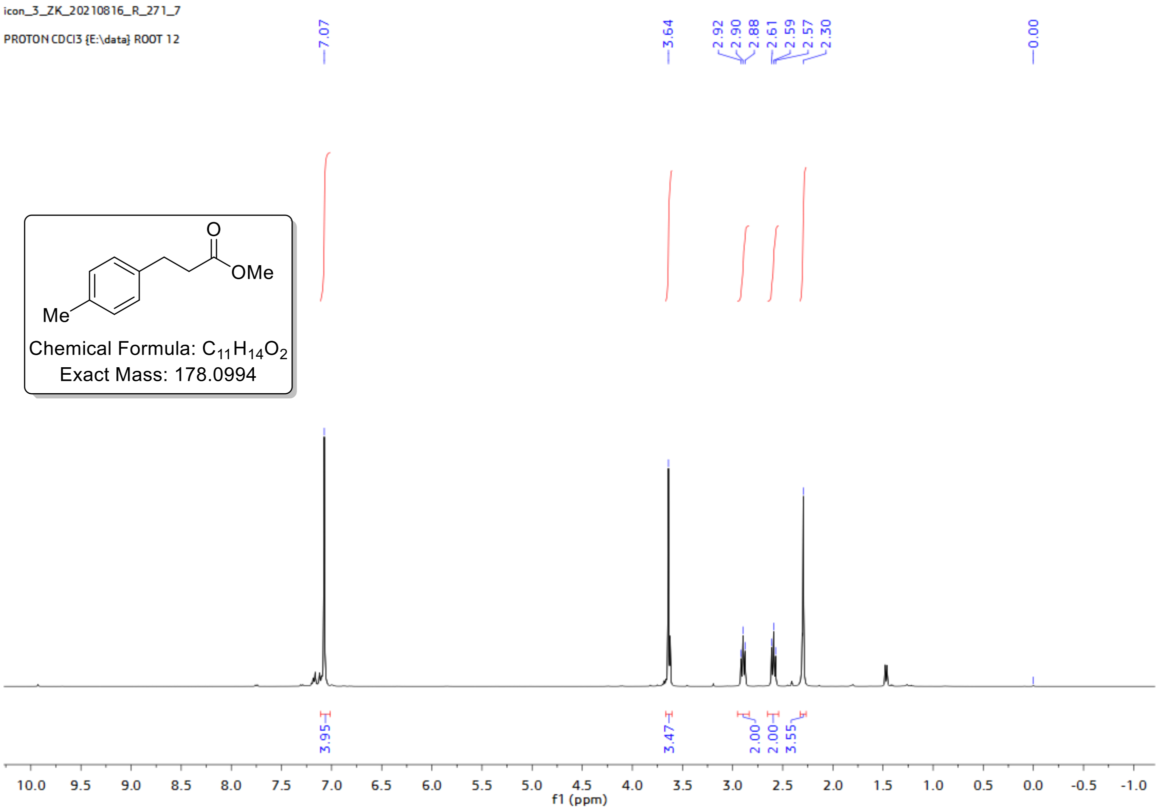

Supplementary Fig. 126 ^{1}H NMR (400 MHz, 20 ºC) spectrum of product 21 in $CDCl_3$.

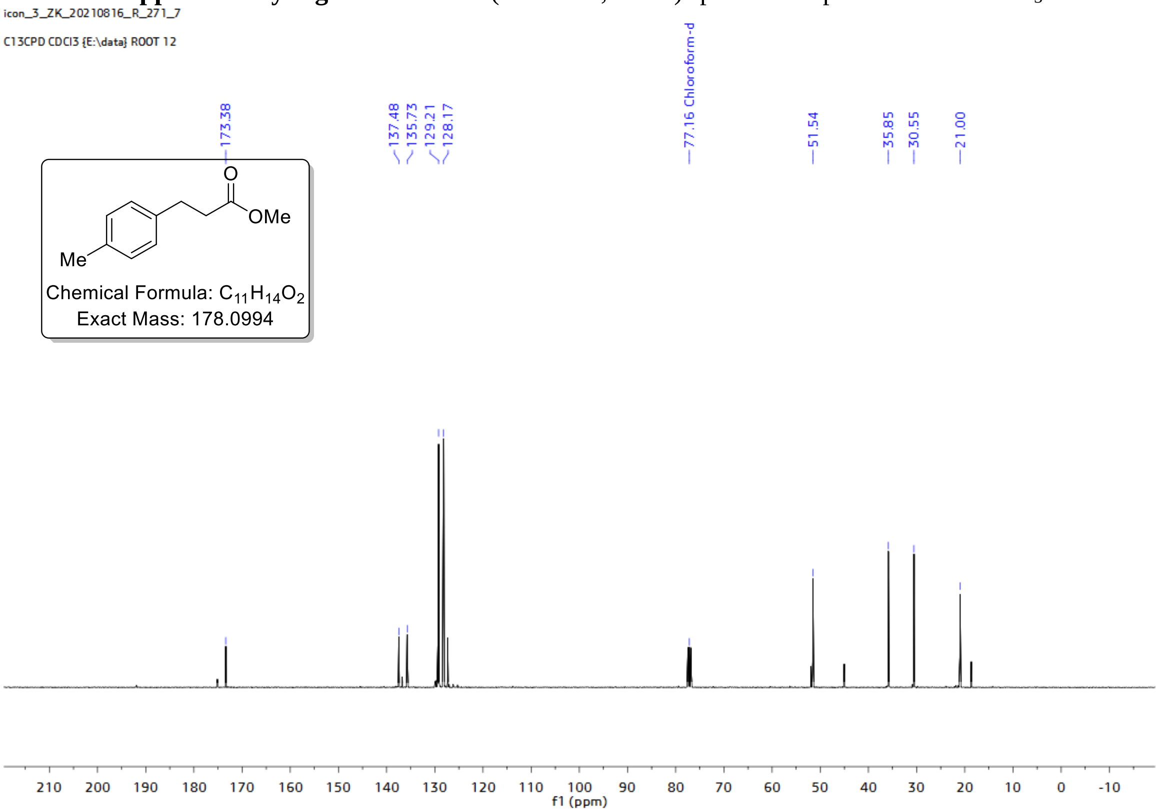

Supplementary Fig. 127 ^{13}C NMR (101 MHz, 20 ºC) spectrum of product 21 in $CDCl_3$.

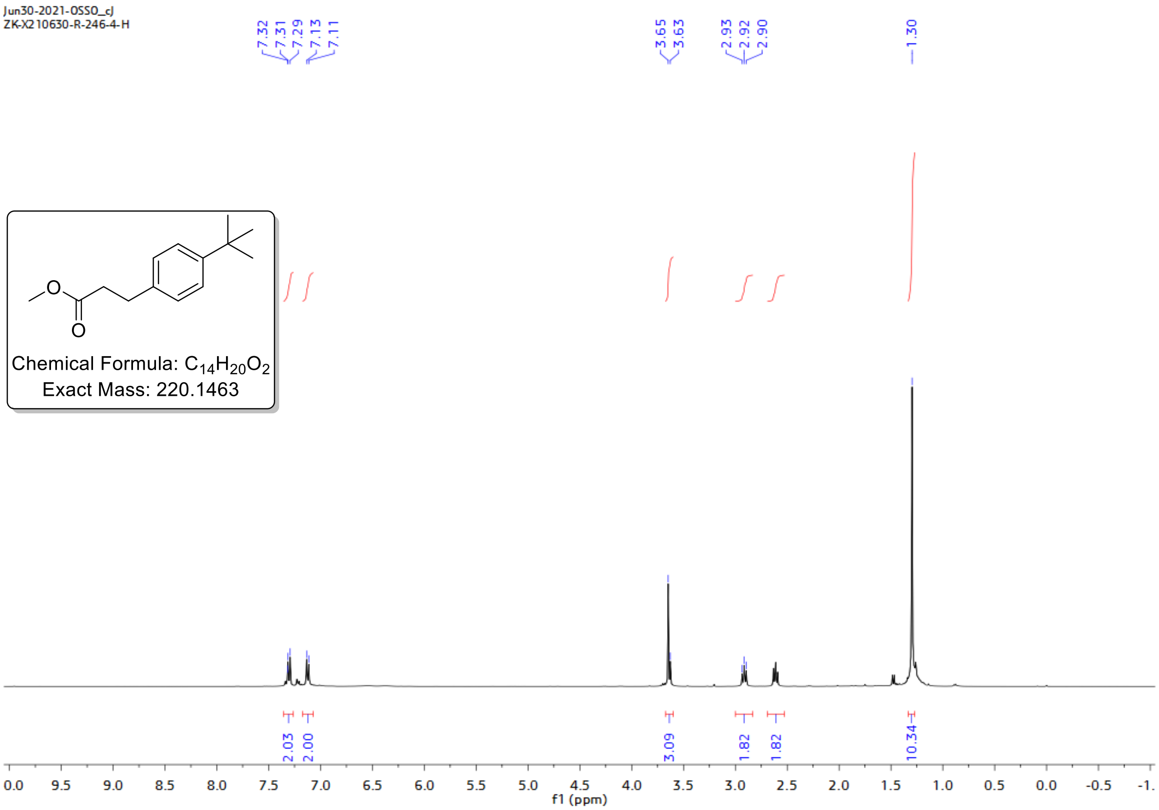

Supplementary Fig. 128 ^{1}H NMR (400 MHz, 20 ºC) spectrum of product 22 in $CDCl_3$.

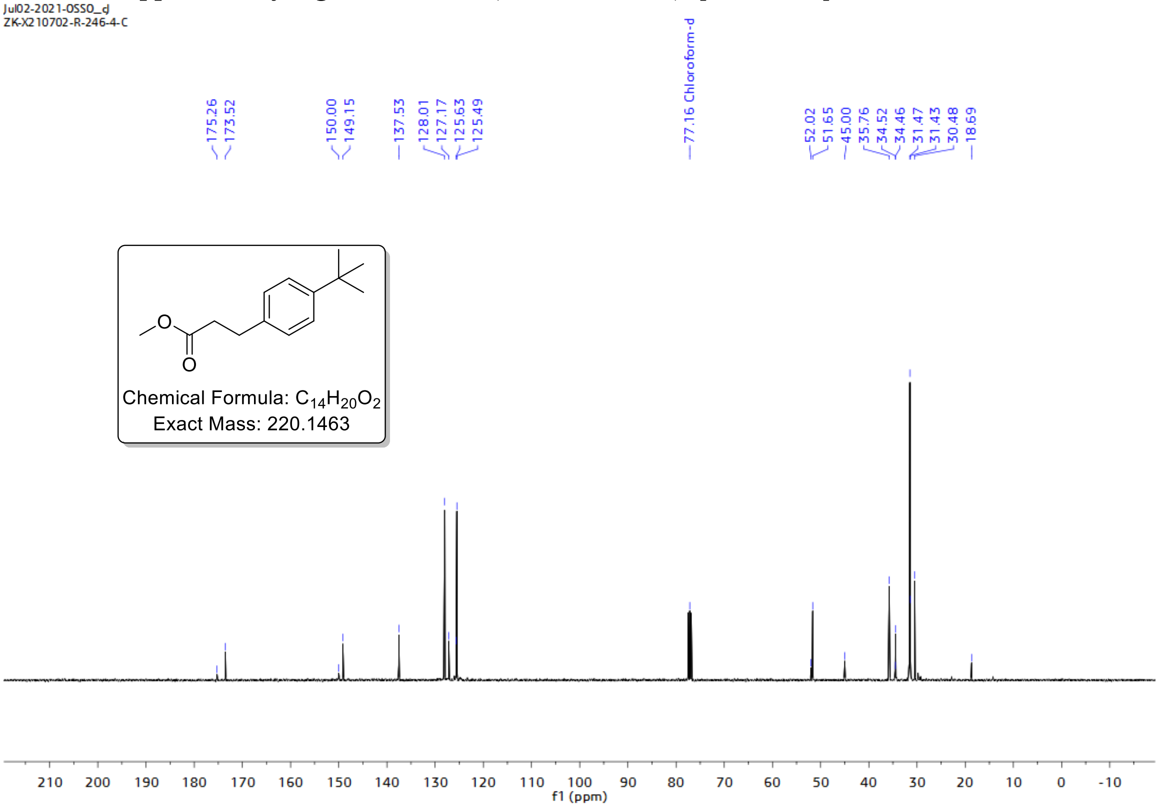

Supplementary Fig. 129 ^{13}C NMR (101 MHz, 20 ºC) spectrum of product 22 in $CDCl_3$.

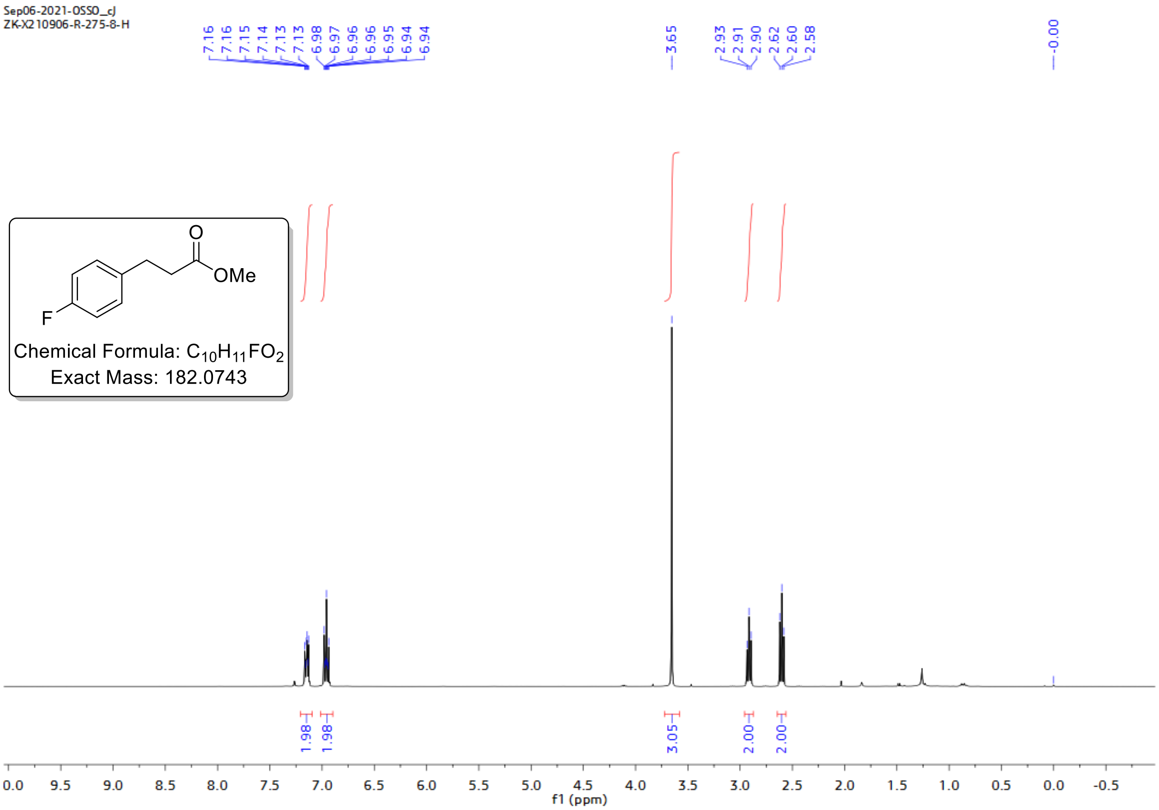

Supplementary Fig. 130 ^{1}H NMR (400 MHz, 20 ºC) spectrum of product 23 in $CDCl_3$.

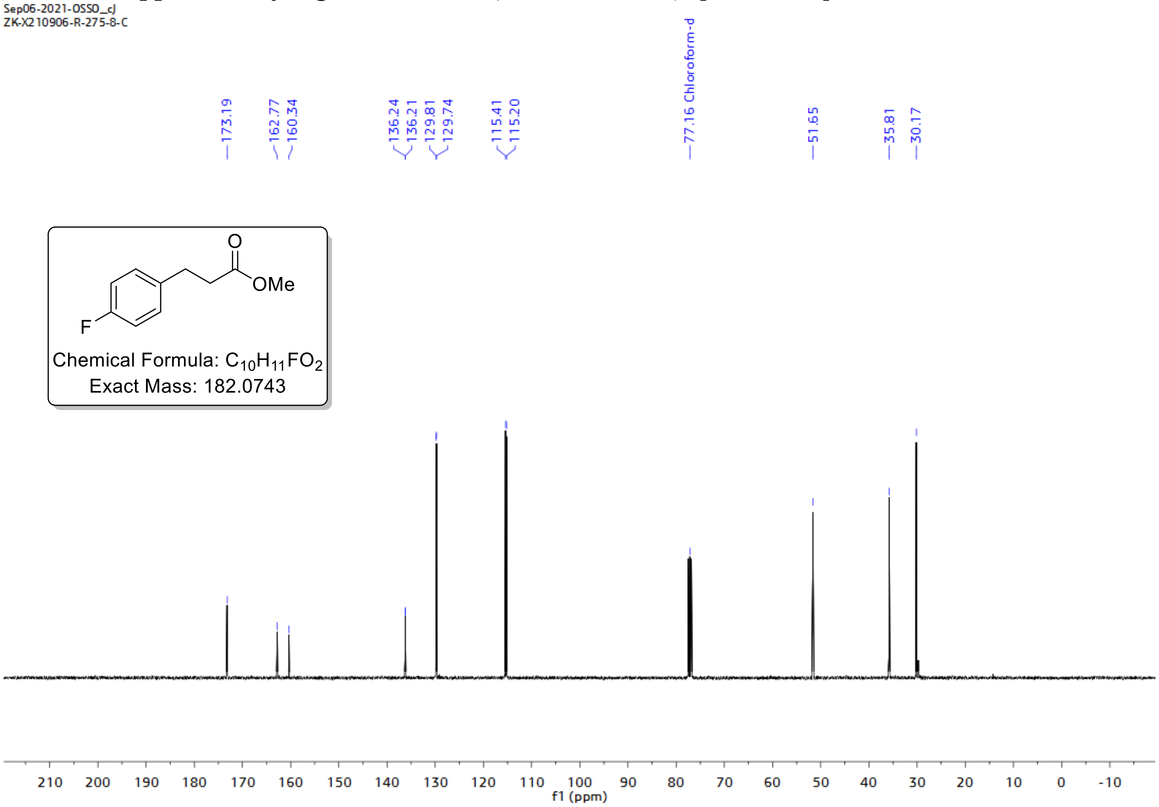

Supplementary Fig. 131 ^{13}C NMR (101 MHz, 20 ºC) spectrum of product 23 in $CDCl_3$.

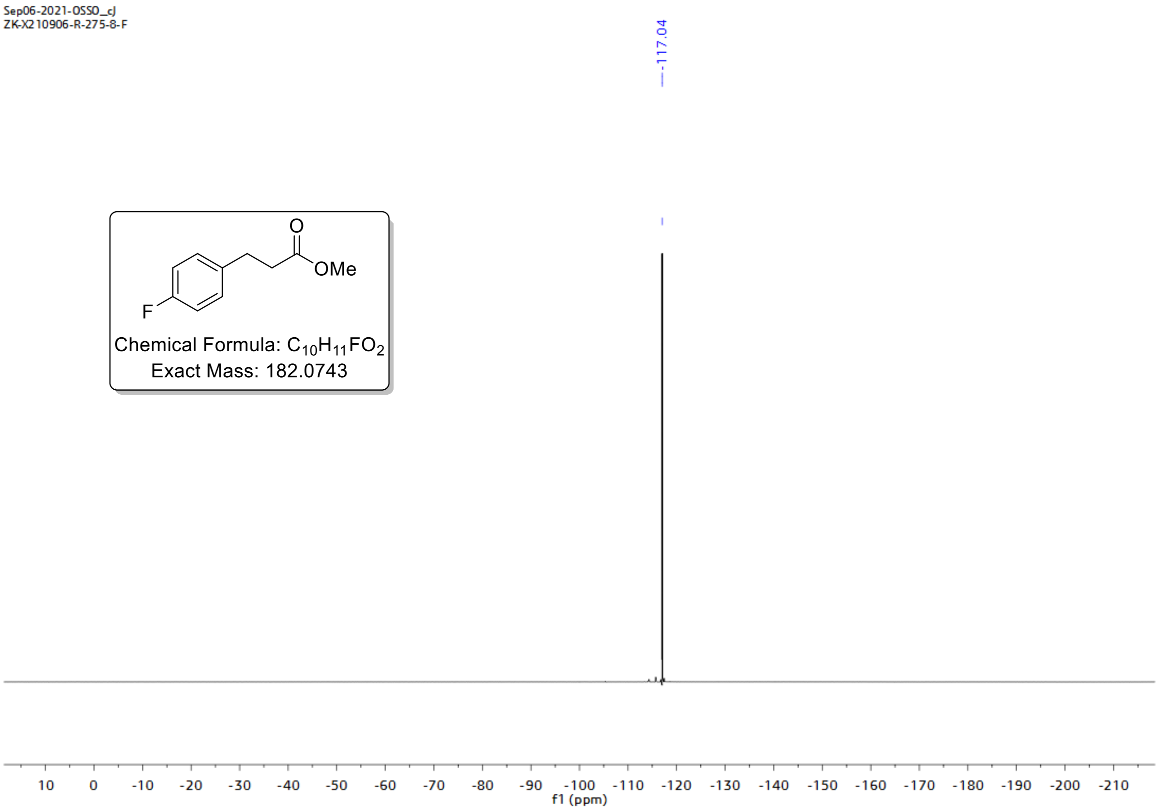

Supplementary Fig. 132 ^{19}F NMR (376 MHz, 20 ºC) spectrum of product 23 in $CDCl_3$.

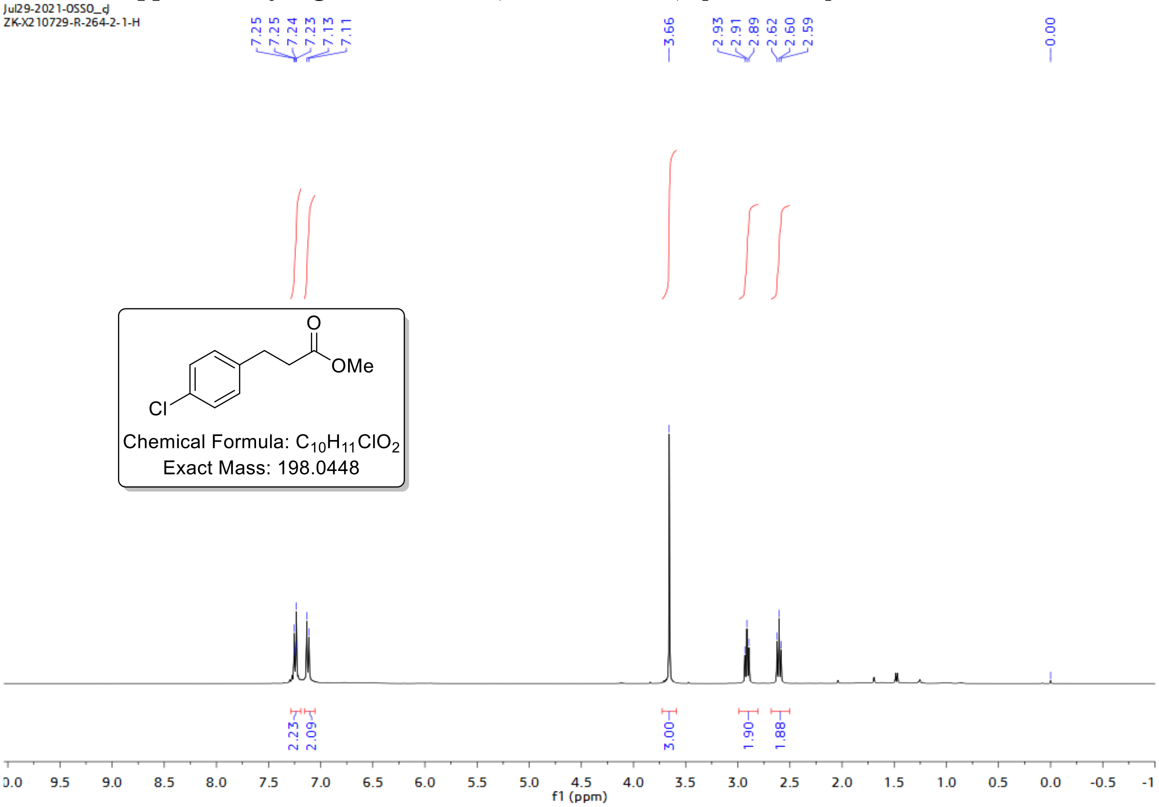

Supplementary Fig. 133 ^{1}H NMR (400 MHz, 20 ºC) spectrum of product 24 in $CDCl_3$.

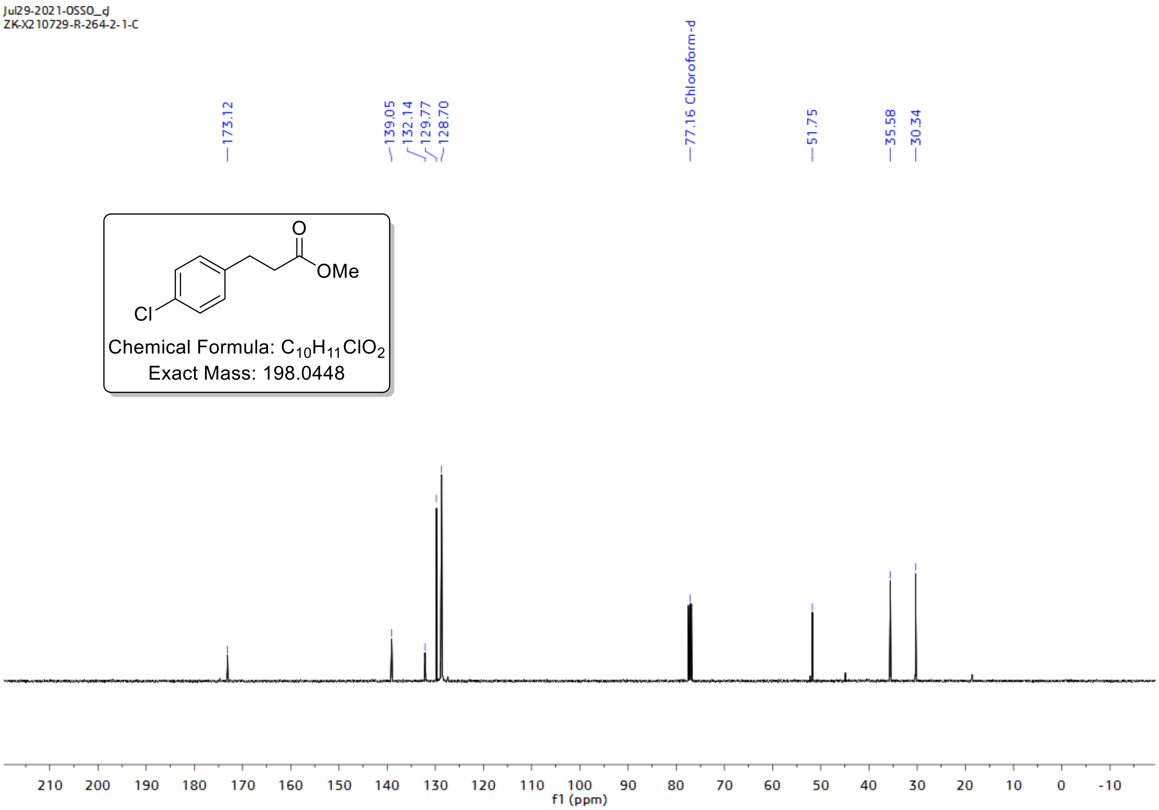

Supplementary Fig. 134 ^{13}C NMR (101 MHz, 20 ºC) spectrum of product 24 in $CDCl_3$.

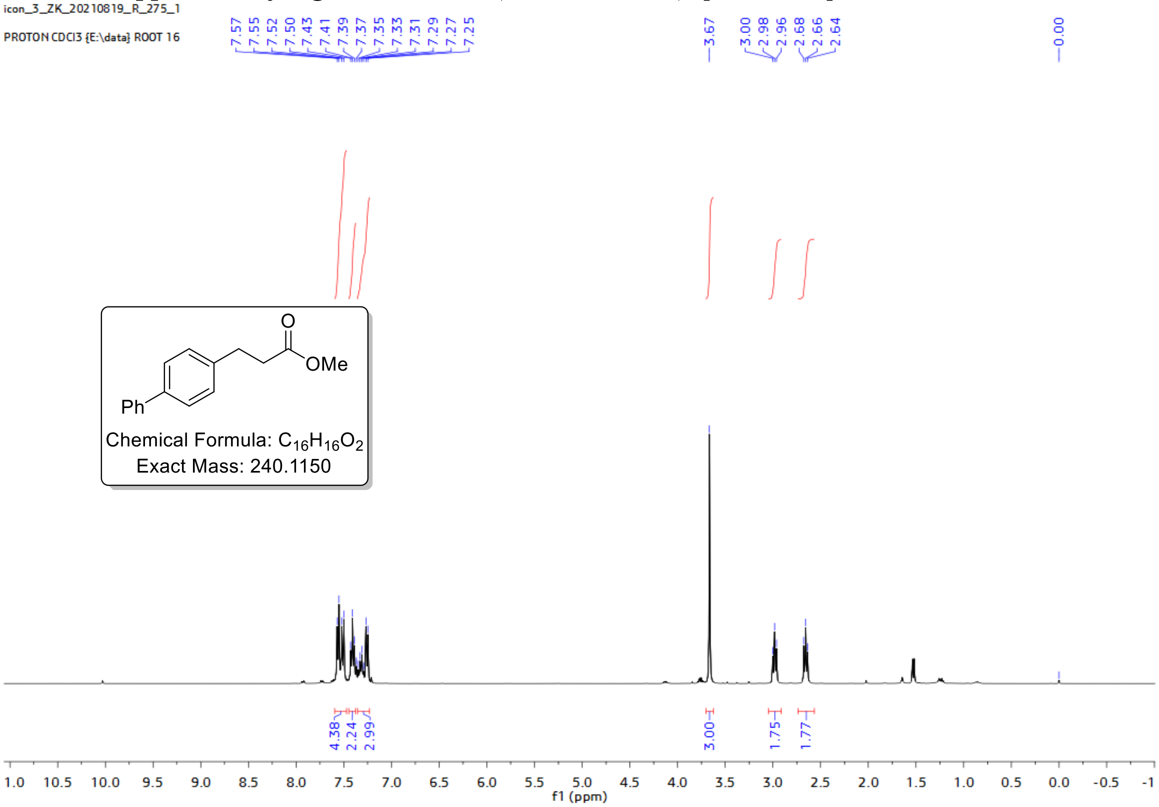

Supplementary Fig. 135 ^{1}H NMR (400 MHz, 20 ºC) spectrum of product 25 in $CDCl_3$.

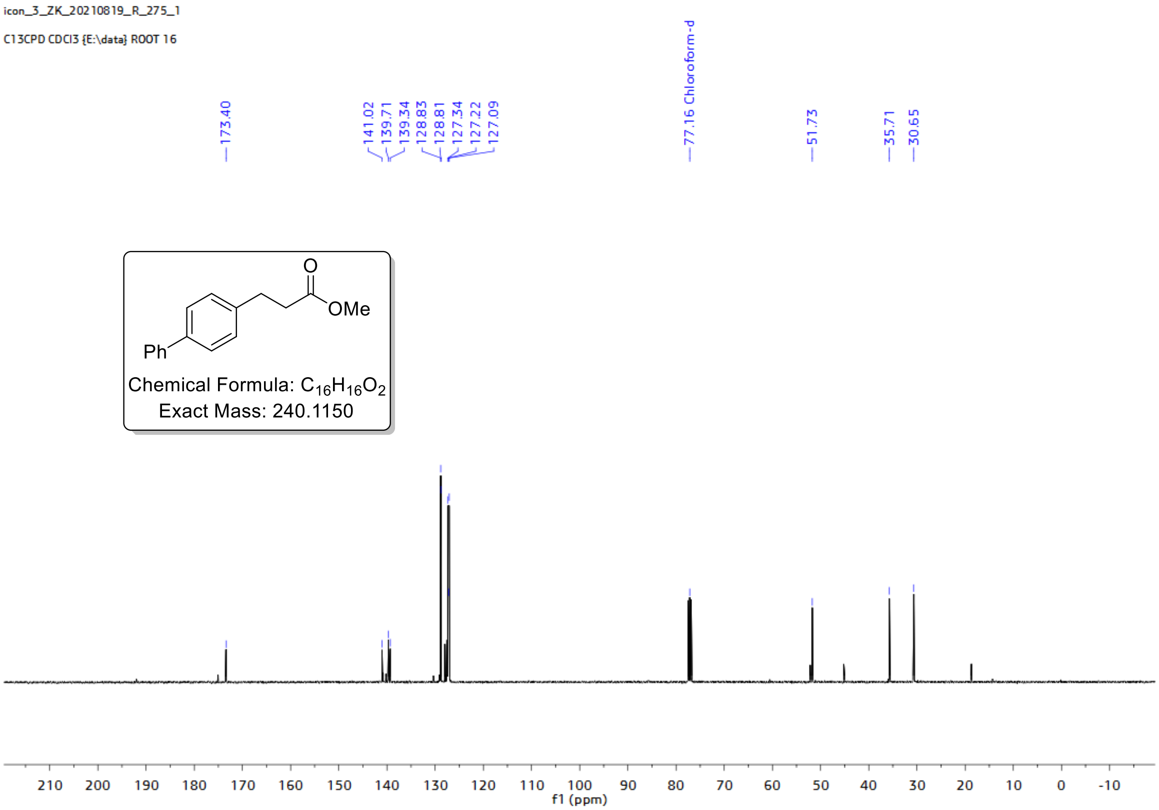

Supplementary Fig. 136 ^{13}C NMR (101 MHz, 20 ºC) spectrum of product 25 in $CDCl_3$.

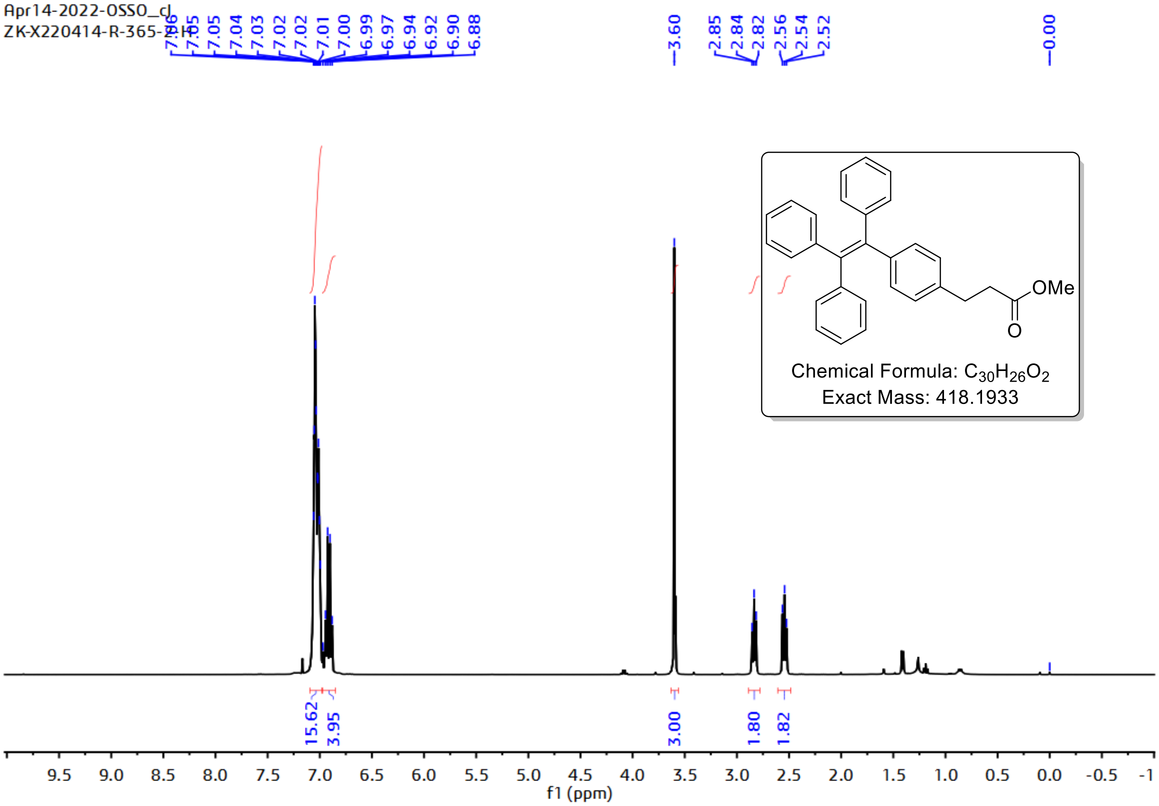

Supplementary Fig. 137 ^{1}H NMR (400 MHz, 20 ºC) spectrum of product 26 in $CDCl_3$.

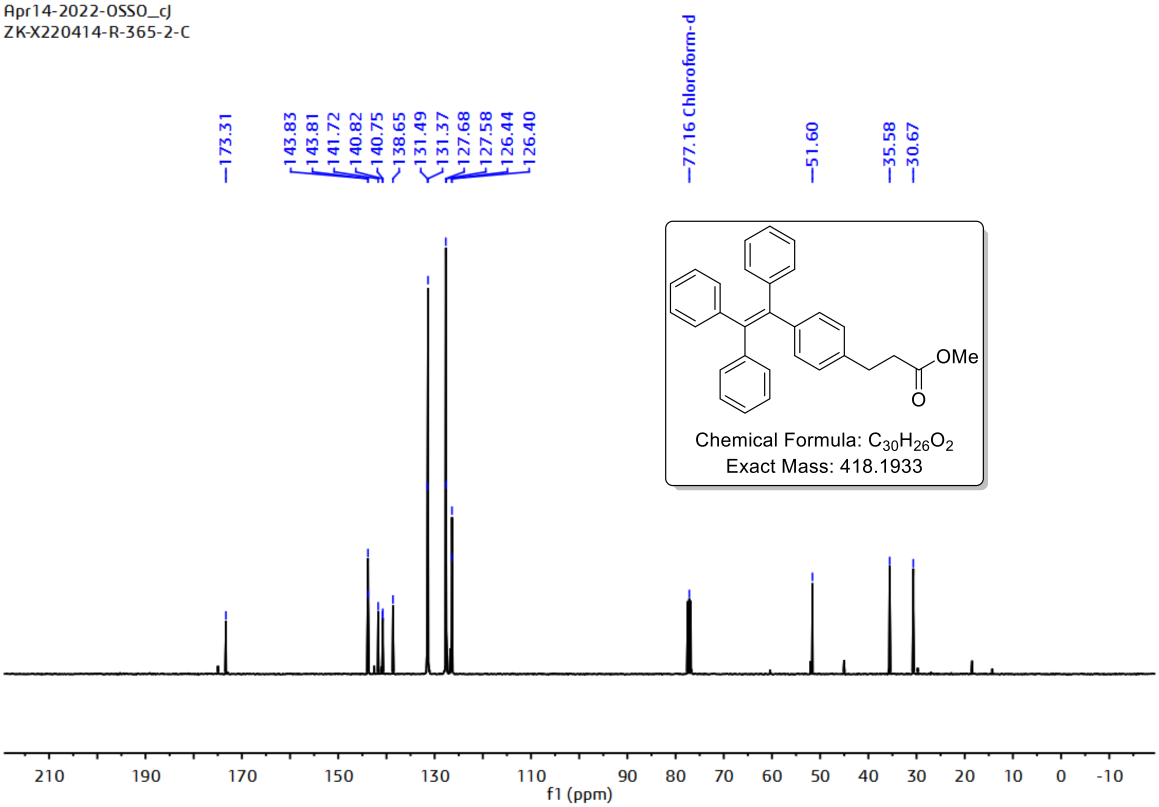

Supplementary Fig. 138 ^{13}C NMR (101 MHz, 20 ºC) spectrum of product 26 in $CDCl_3$.

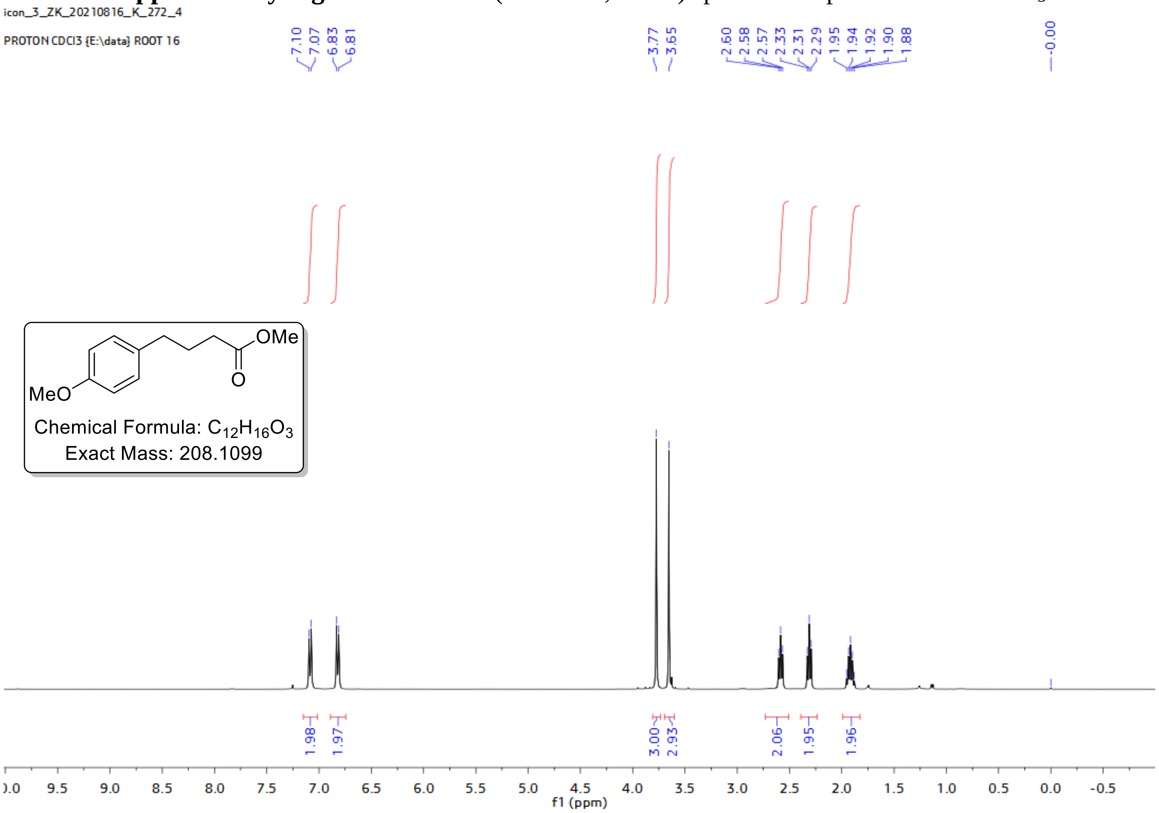

Supplementary Fig. 139 ^{1}H NMR (400 MHz, 20 ºC) spectrum of product 27 in $CDCl_3$.

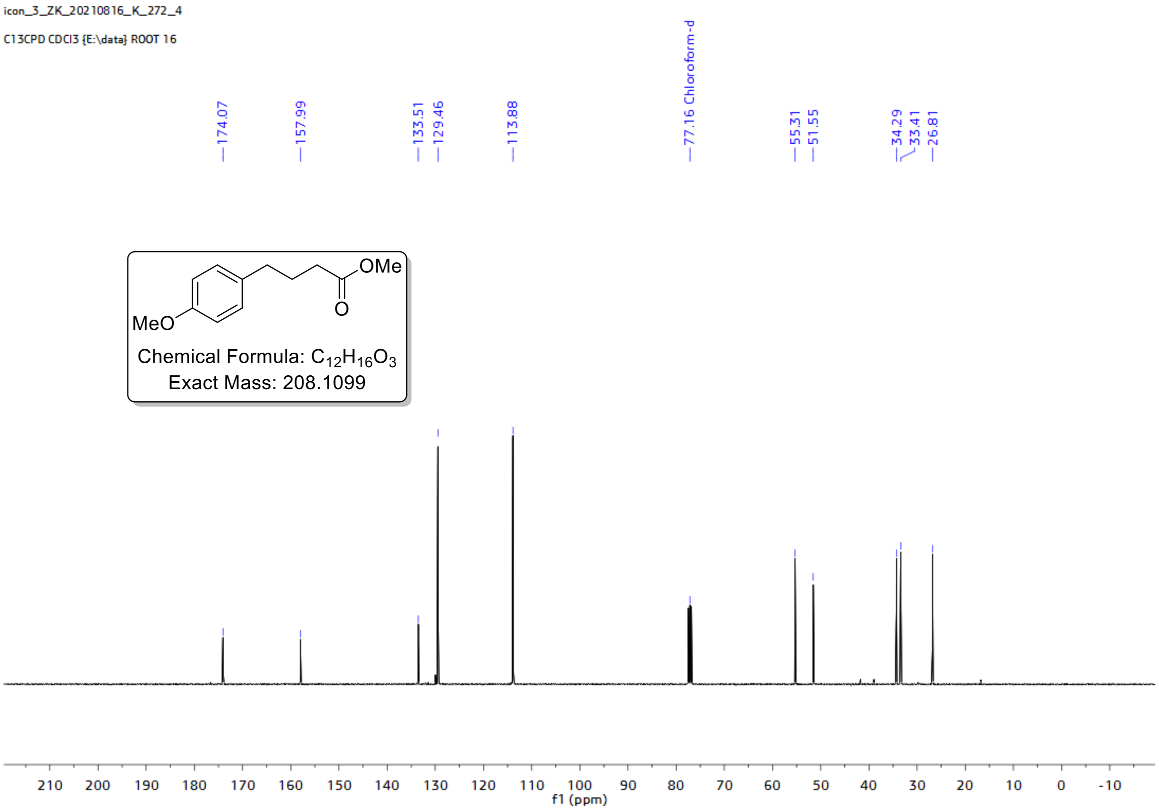

Supplementary Fig. 140 ^{13}C NMR (101 MHz, 20 ºC) spectrum of product 27 in $CDCl_3$.

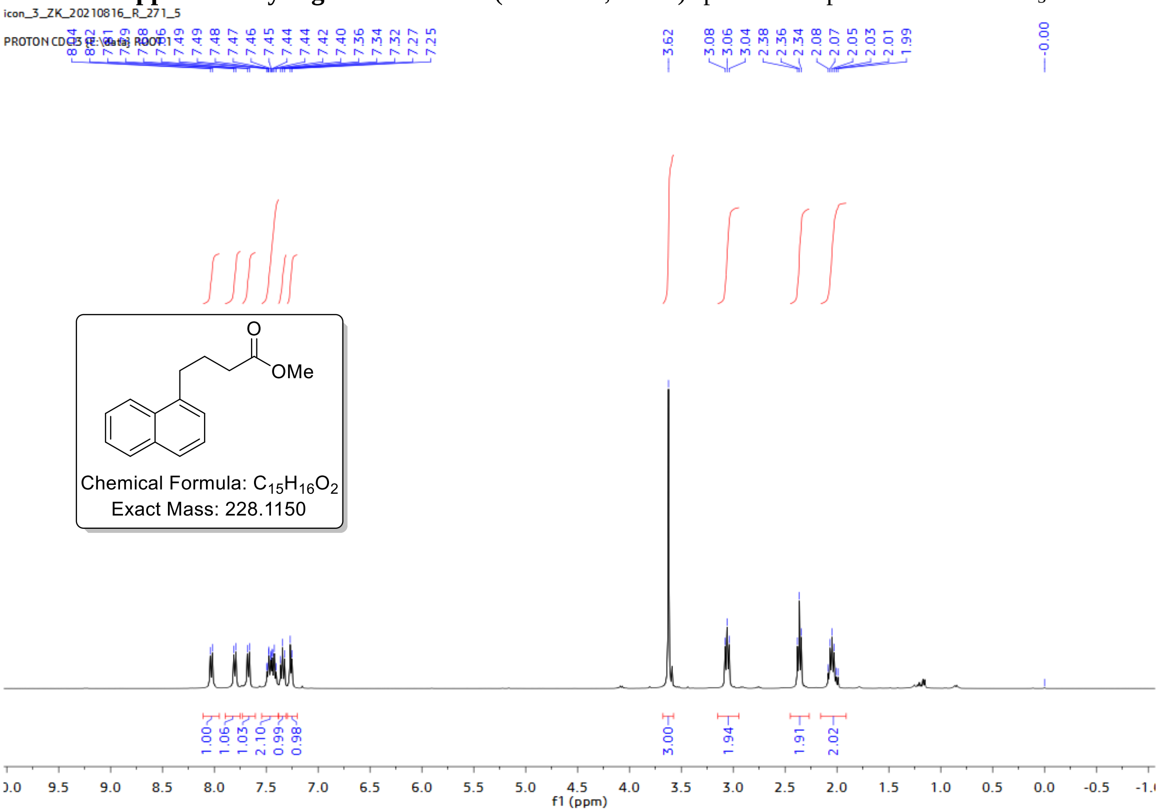

Supplementary Fig. 141 ^{1}H NMR (400 MHz, 20 ºC) spectrum of product 28 in $CDCl_3$.

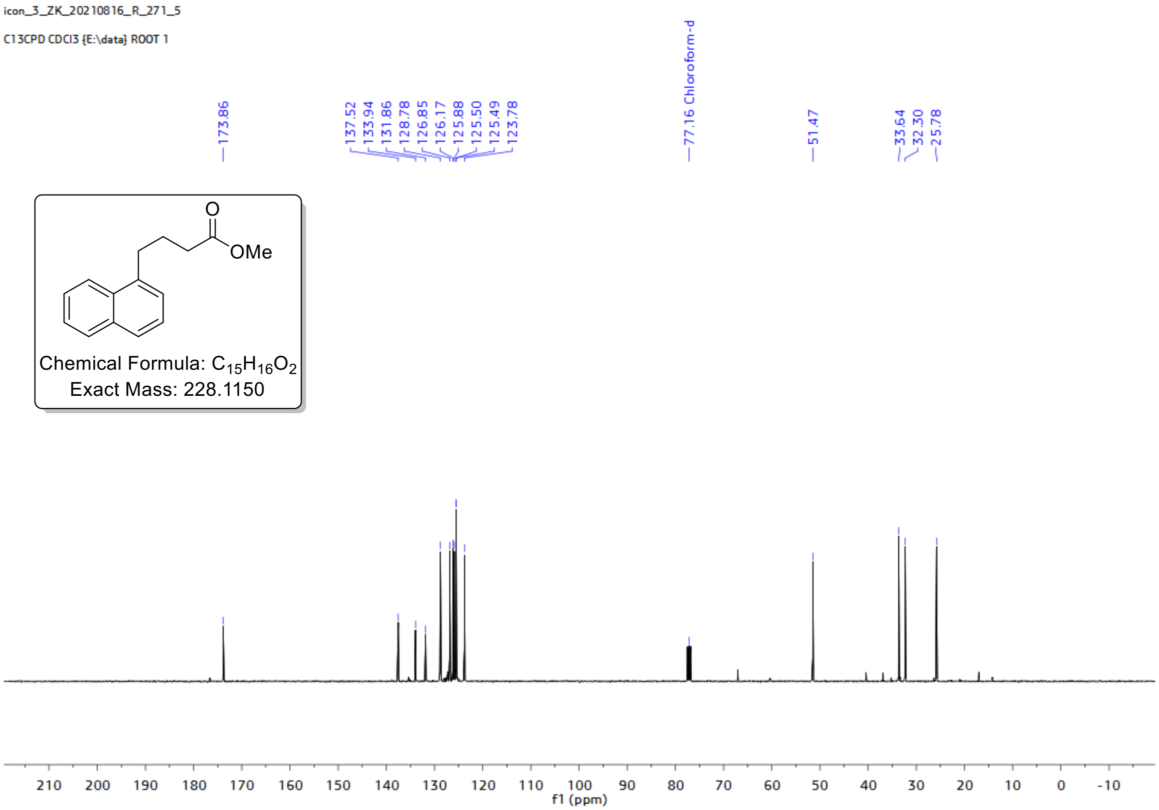

Supplementary Fig. 142 ^{13}C NMR (101 MHz, 20 °C) spectrum of product 28 in $CDCl_3$.

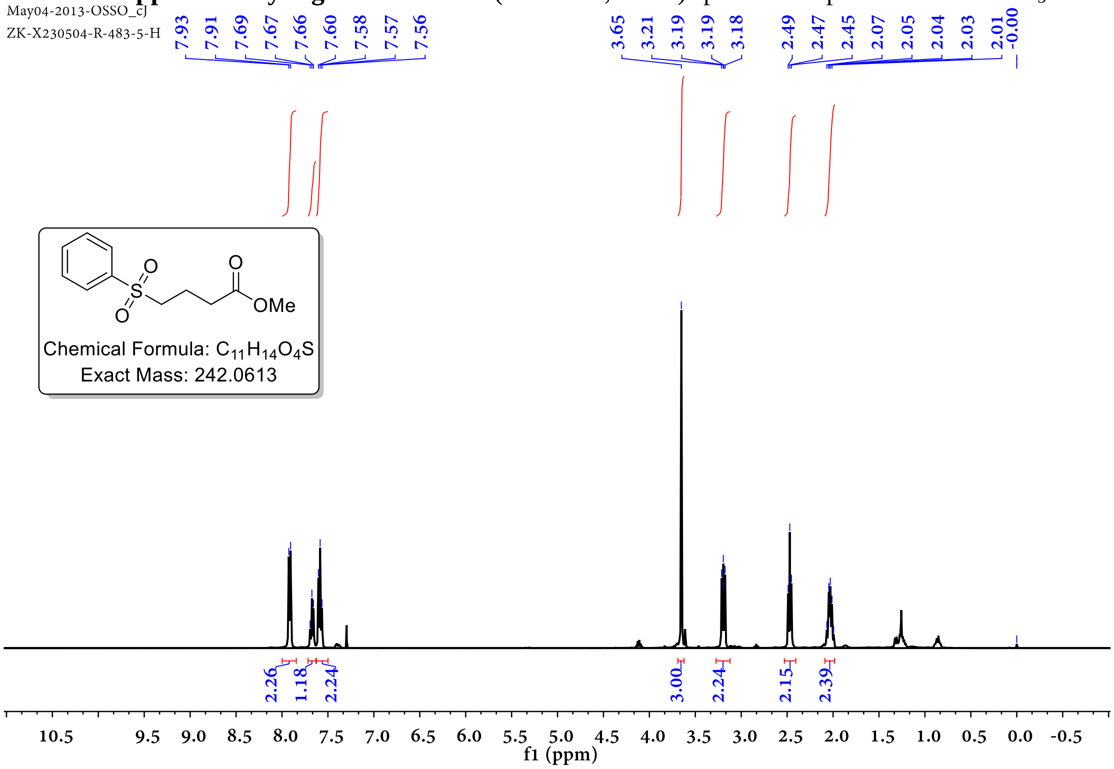

Supplementary Fig. 143 ^{1}H NMR (400 MHz, 20 °C) spectrum of product 29 in $CDCl_3$.

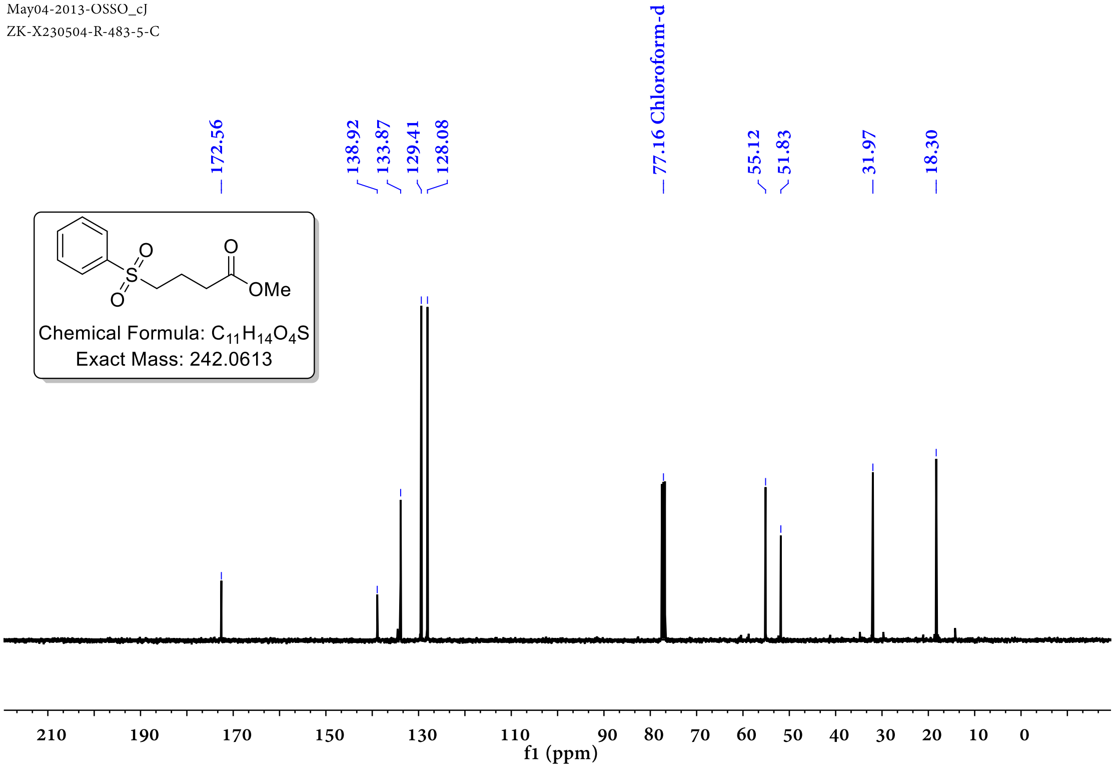

Supplementary Fig. 144 ^{13}C NMR (101 MHz, 20 °C) spectrum of product 29 in $CDCl_3$.

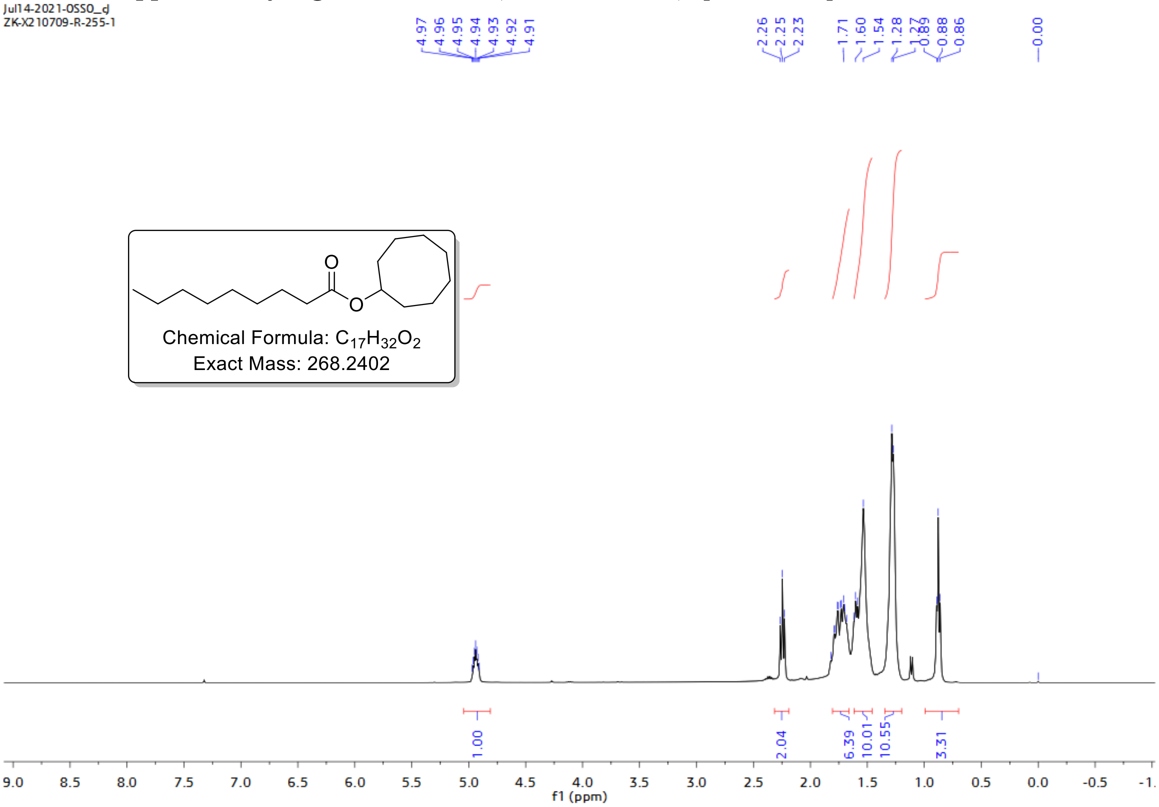

Supplementary Fig. 145 ^{1}H NMR (400 MHz, 20 °C) spectrum of product 36 in $CDCl_3$.

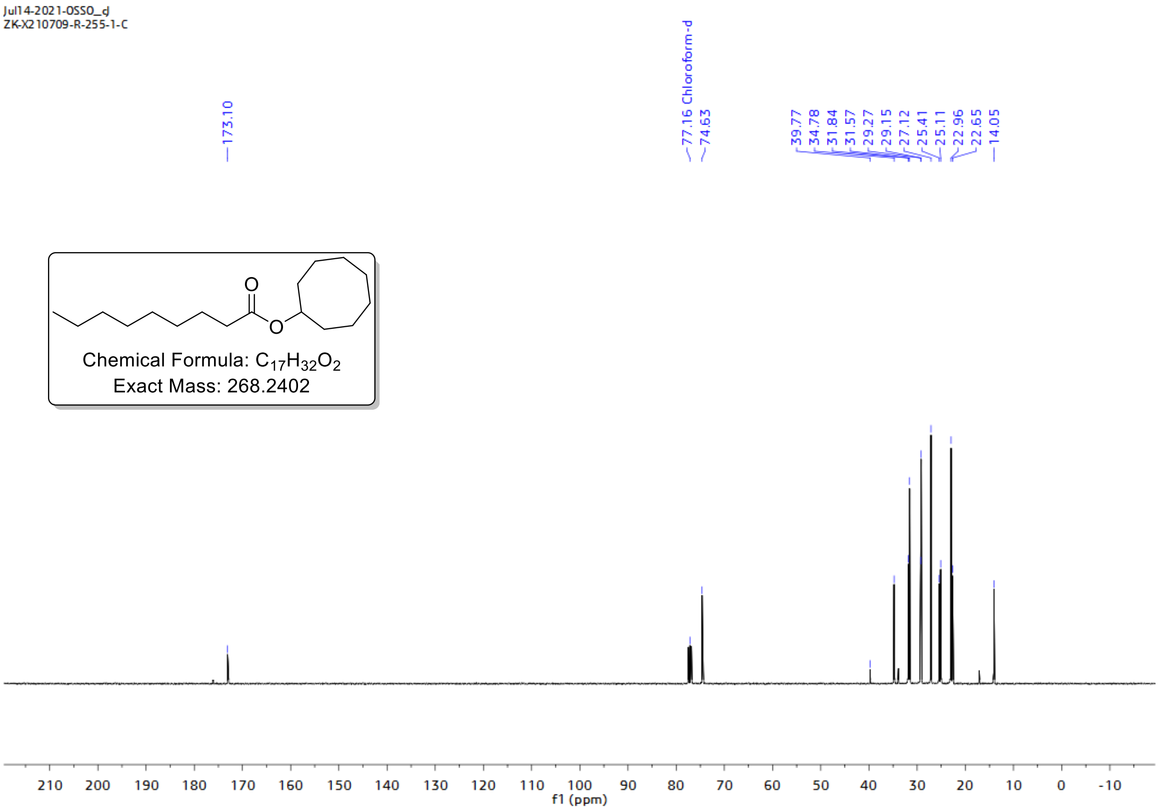

Supplementary Fig. 146 ^{13}C NMR (101 MHz, 20 ºC) spectrum of product 36 in $CDCl_3$.

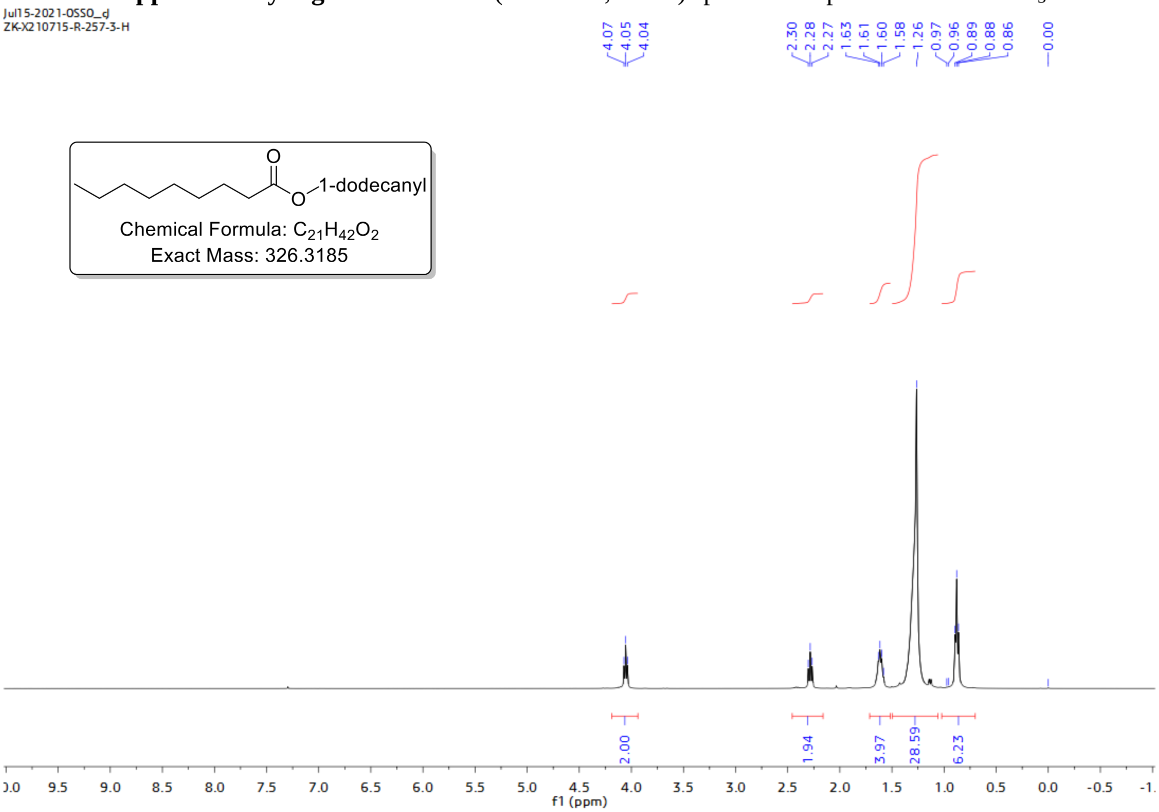

Supplementary Fig. 147 ^{1}H NMR (400 MHz, 20 ºC) spectrum of product 37 in $CDCl_3$.

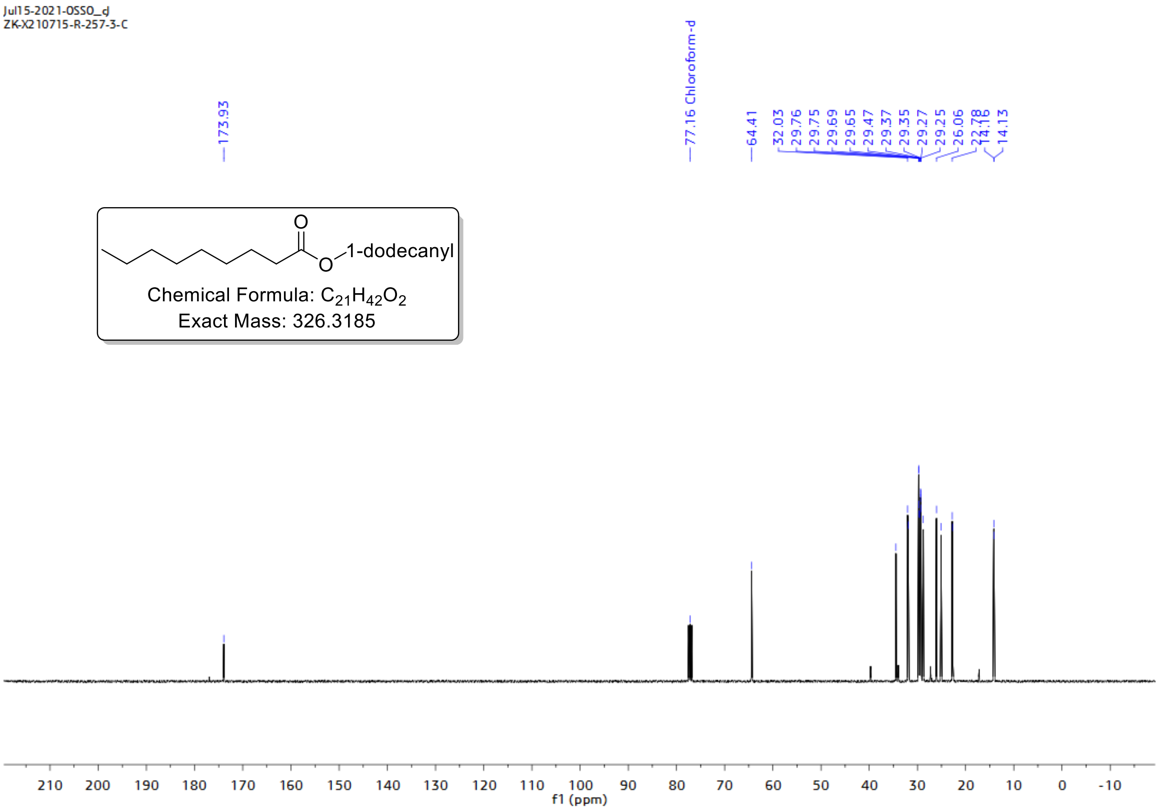

Supplementary Fig. 148 ^{13}C NMR (101 MHz, 20 ºC) spectrum of product 37 in $CDCl_3$.

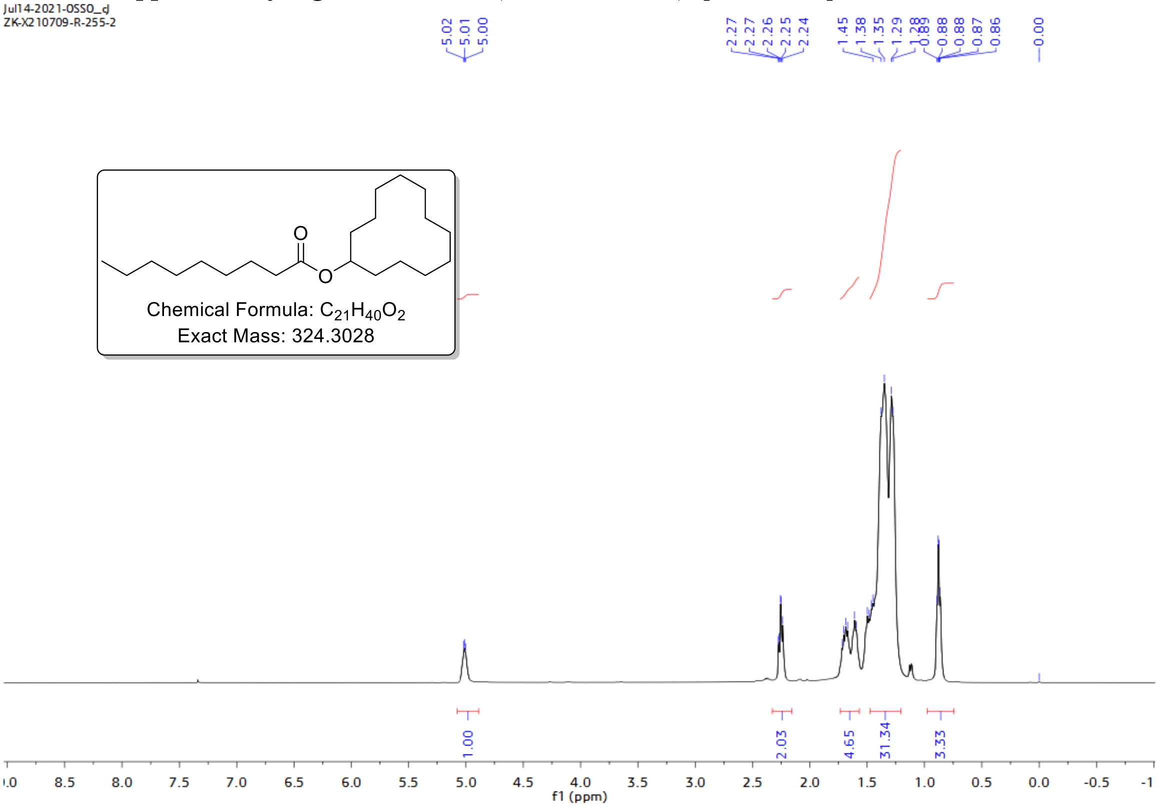

Supplementary Fig. 149 ^{1}H NMR (400 MHz, 20 ºC) spectrum of product 38 in $CDCl_3$.

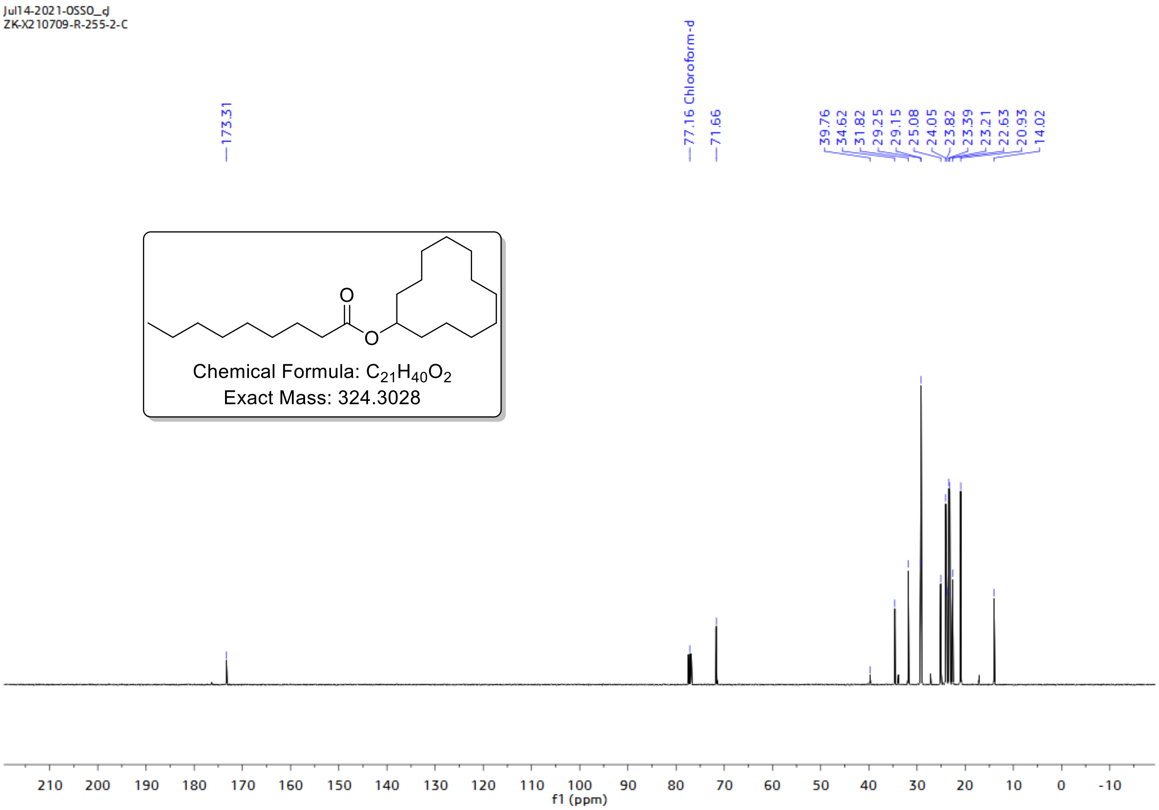

Supplementary Fig. 150 ^{13}C NMR (101 MHz, 20 °C) spectrum of product 38 in $CDCl_3$.

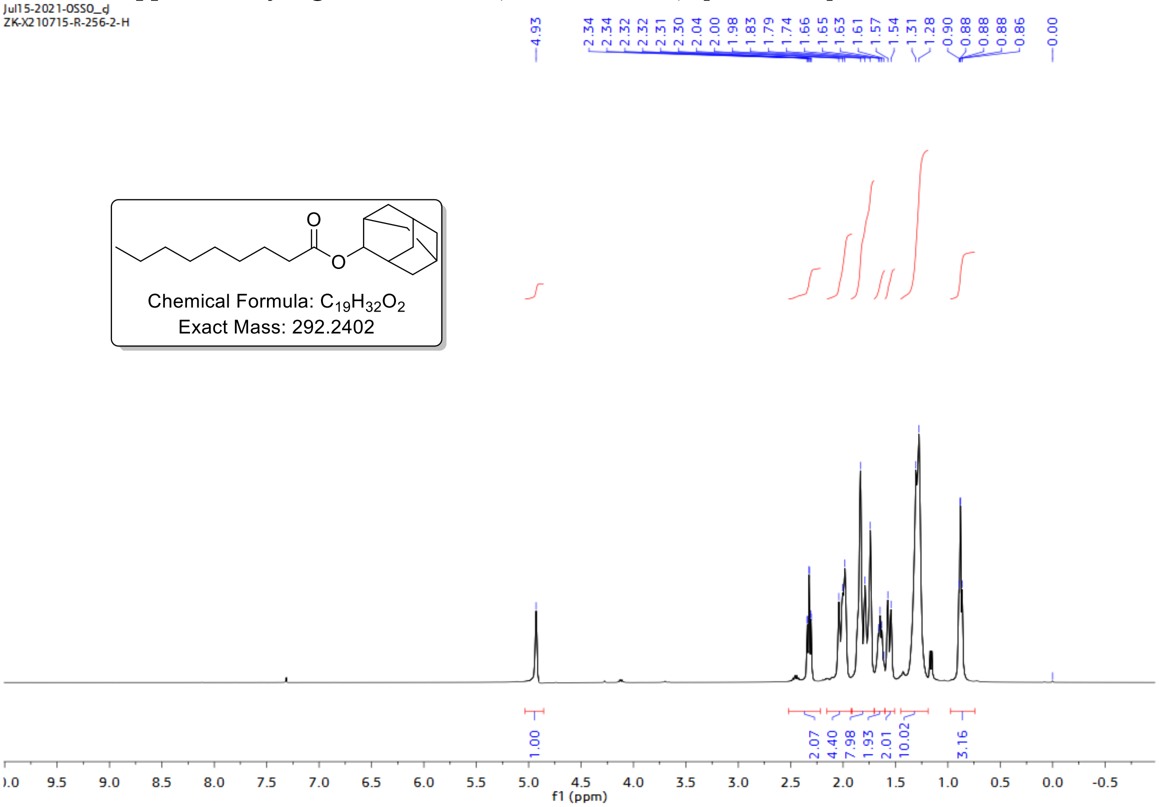

Supplementary Fig. 151 ^{1}H NMR (400 MHz, 20 °C) spectrum of product 39 in $CDCl_3$.

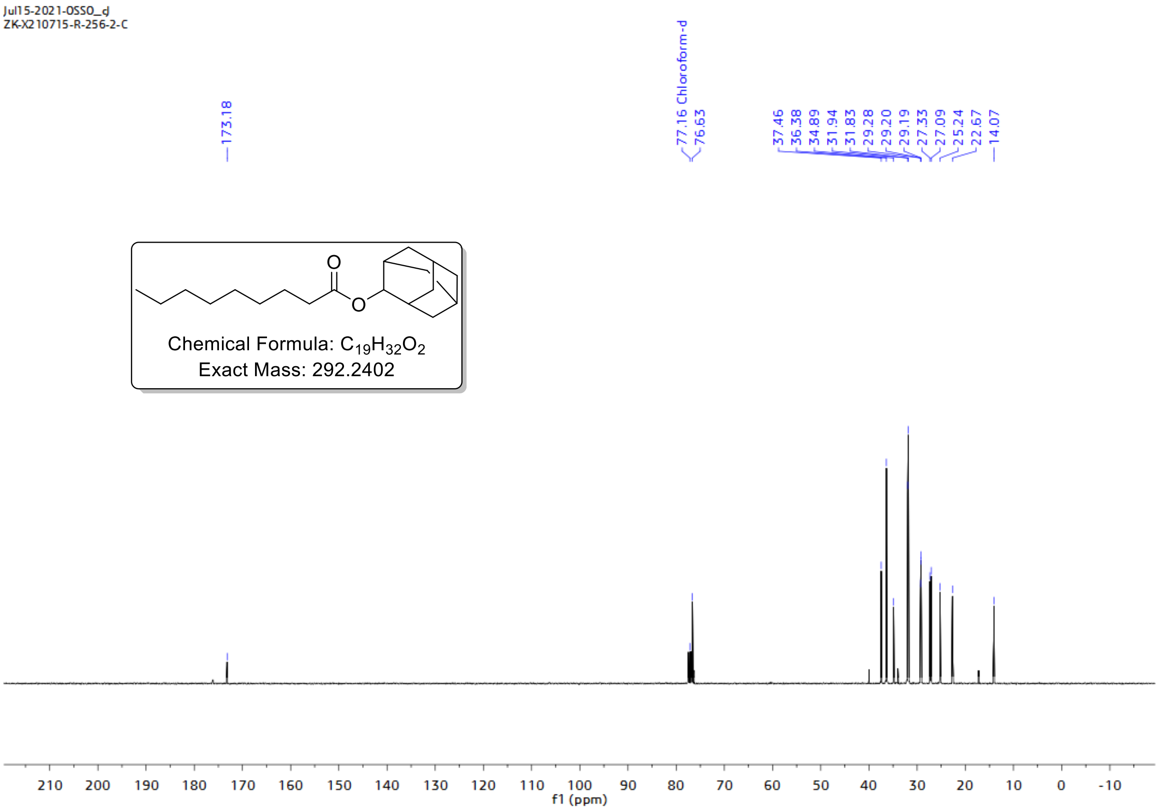

Supplementary Fig. 152 ^{13}C NMR (101 MHz, 20 ºC) spectrum of product 39 in $CDCl_3$.

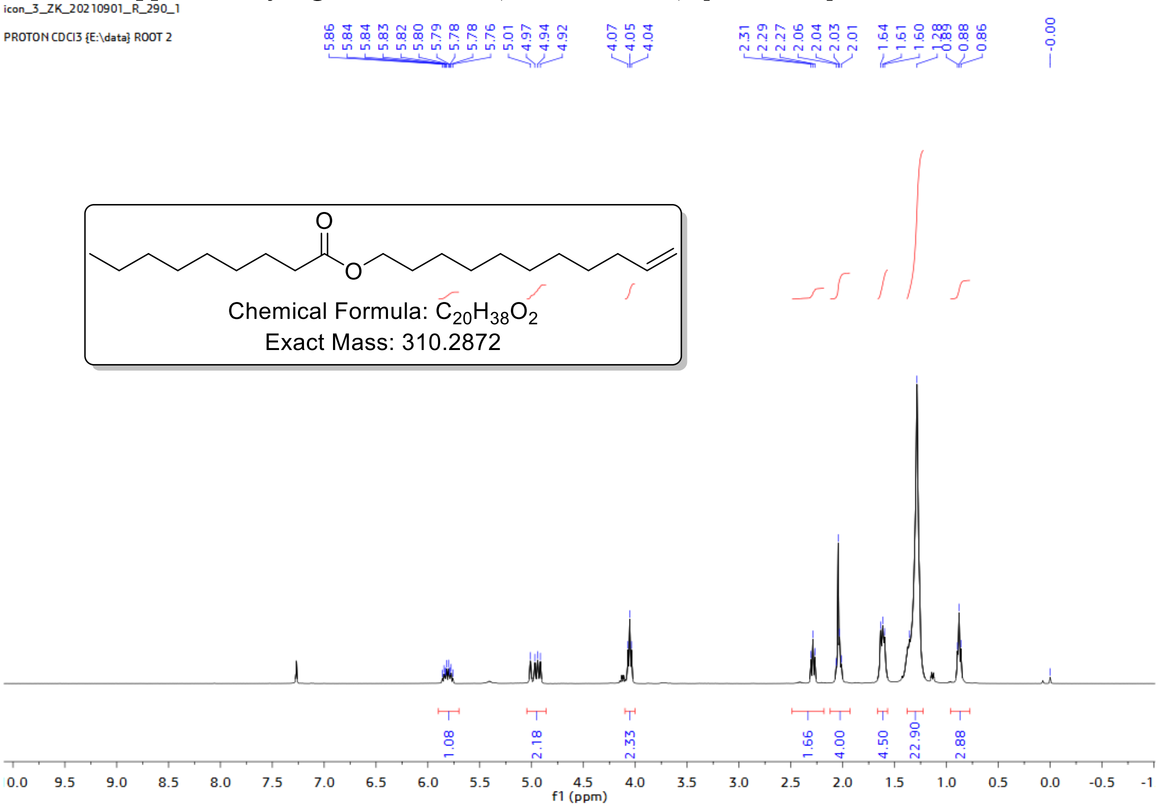

Supplementary Fig. 153 ^{1}H NMR (400 MHz, 20 ºC) spectrum of product 40 in $CDCl_3$.

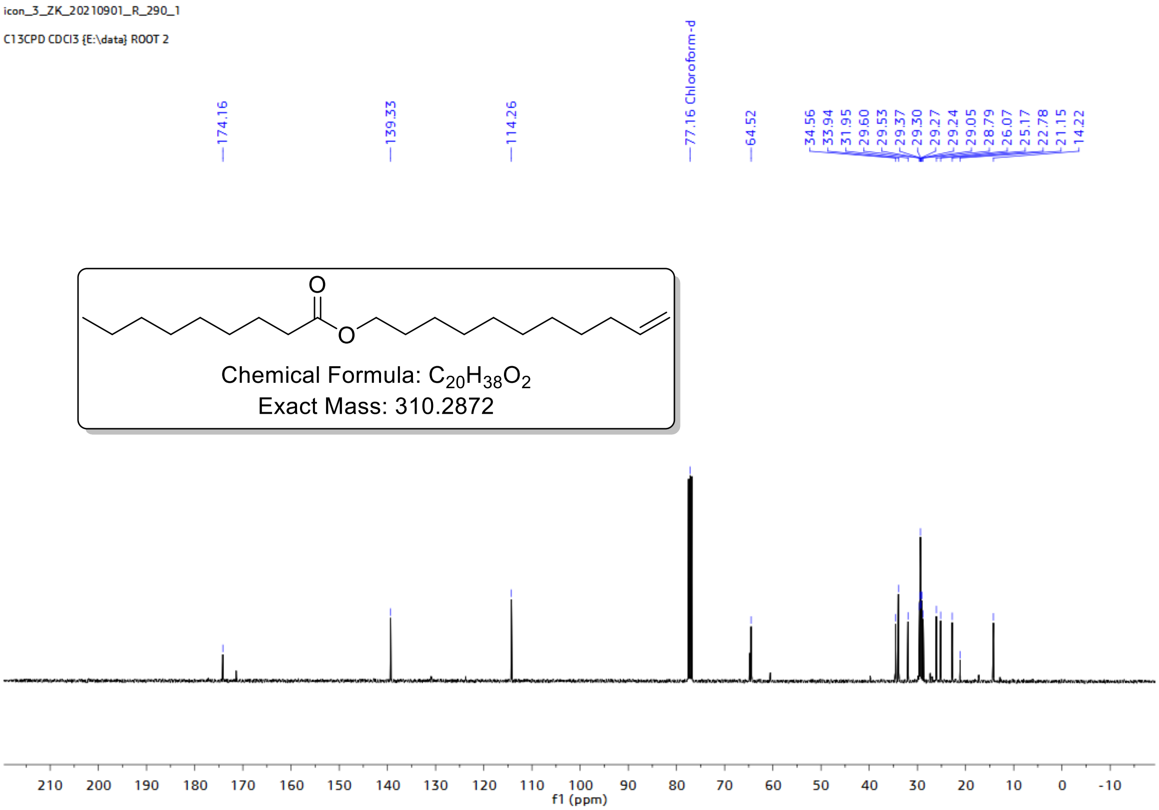

Supplementary Fig. 154 ^{13}C NMR (101 MHz, 20 ºC) spectrum of product 40 in $CDCl_3$.

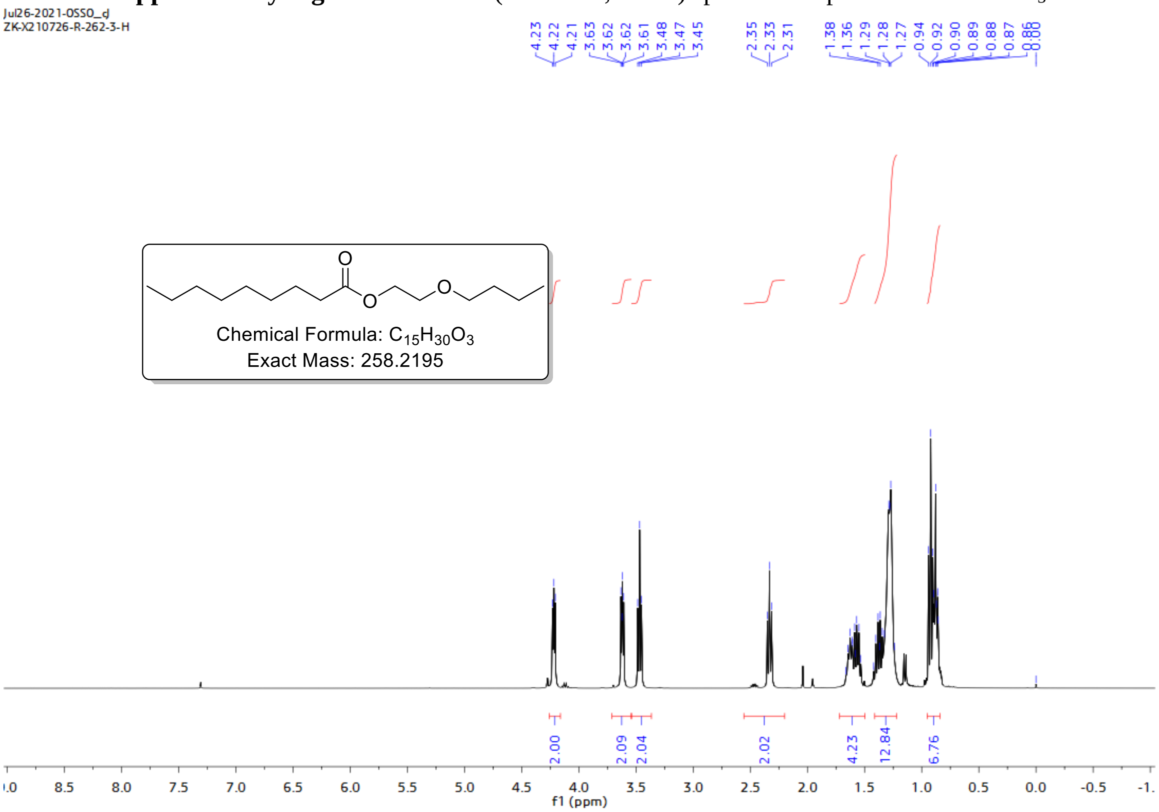

Supplementary Fig. 155 ^{1}H NMR (400 MHz, 20 ºC) spectrum of product 41 in $CDCl_3$.

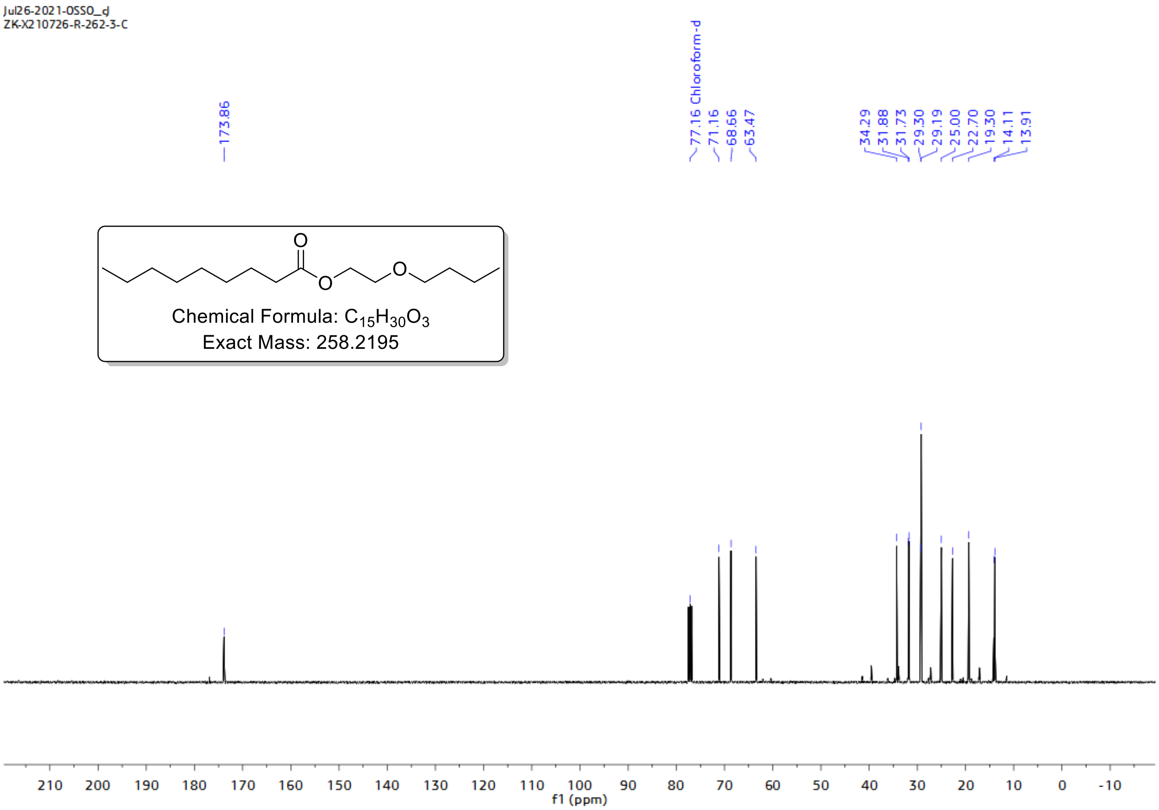

Supplementary Fig. 156 ^{13}C NMR (101 MHz, 20 ºC) spectrum of product 41 in $CDCl_3$.

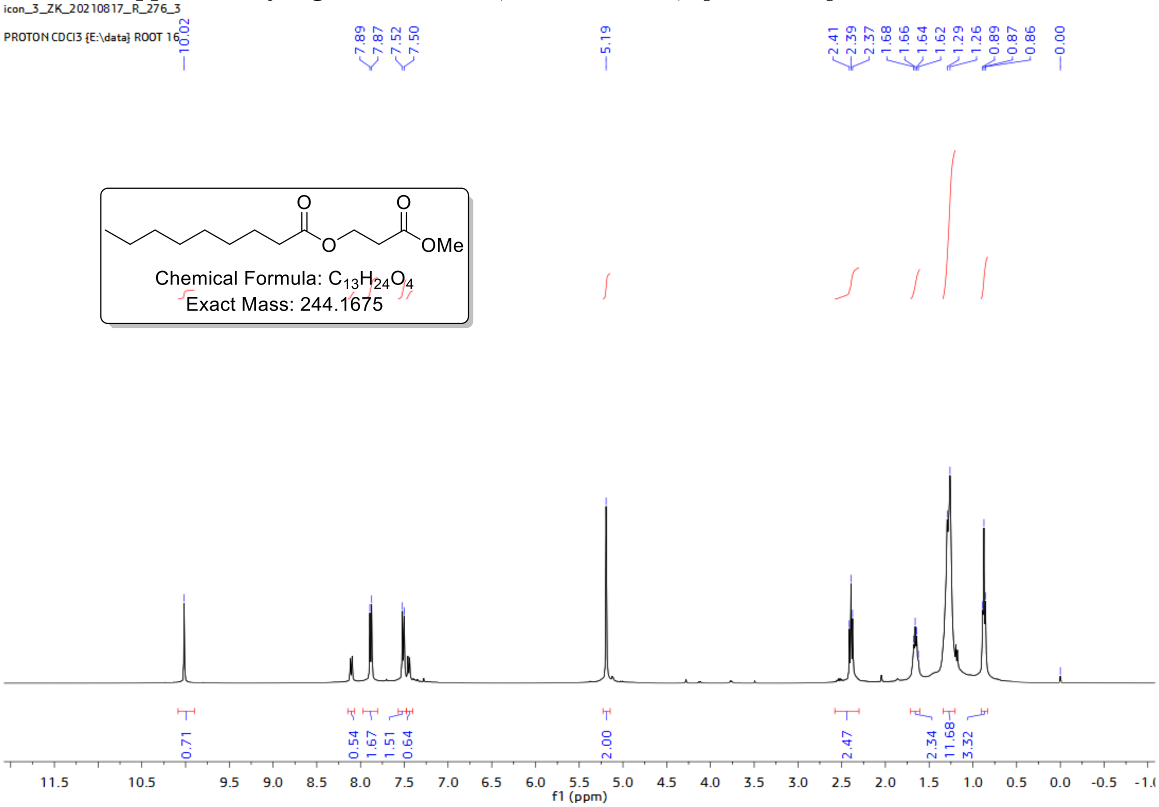

Supplementary Fig. 157 ^{1}H NMR (400 MHz, 20 ºC) spectrum of product 42 in $CDCl_3$.

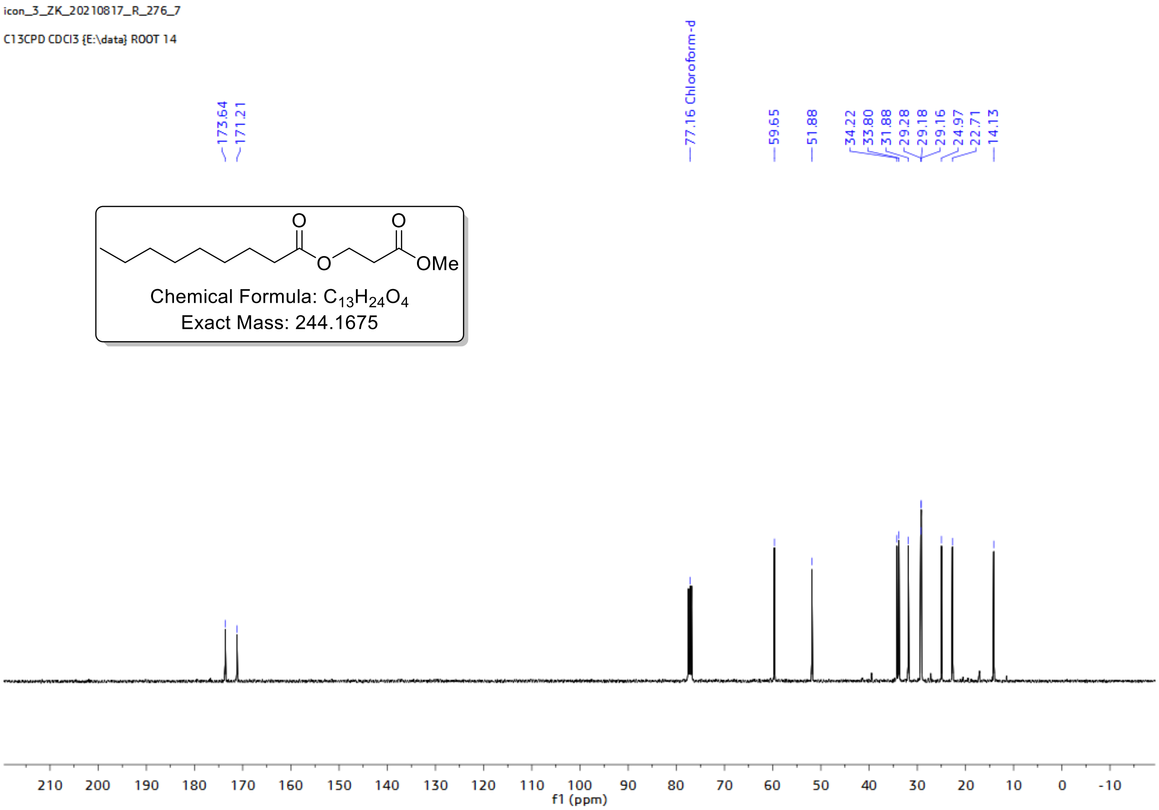

Supplementary Fig. 158 ^{13}C NMR (101 MHz, 20 ºC) spectrum of product 42 in $CDCl_3$.

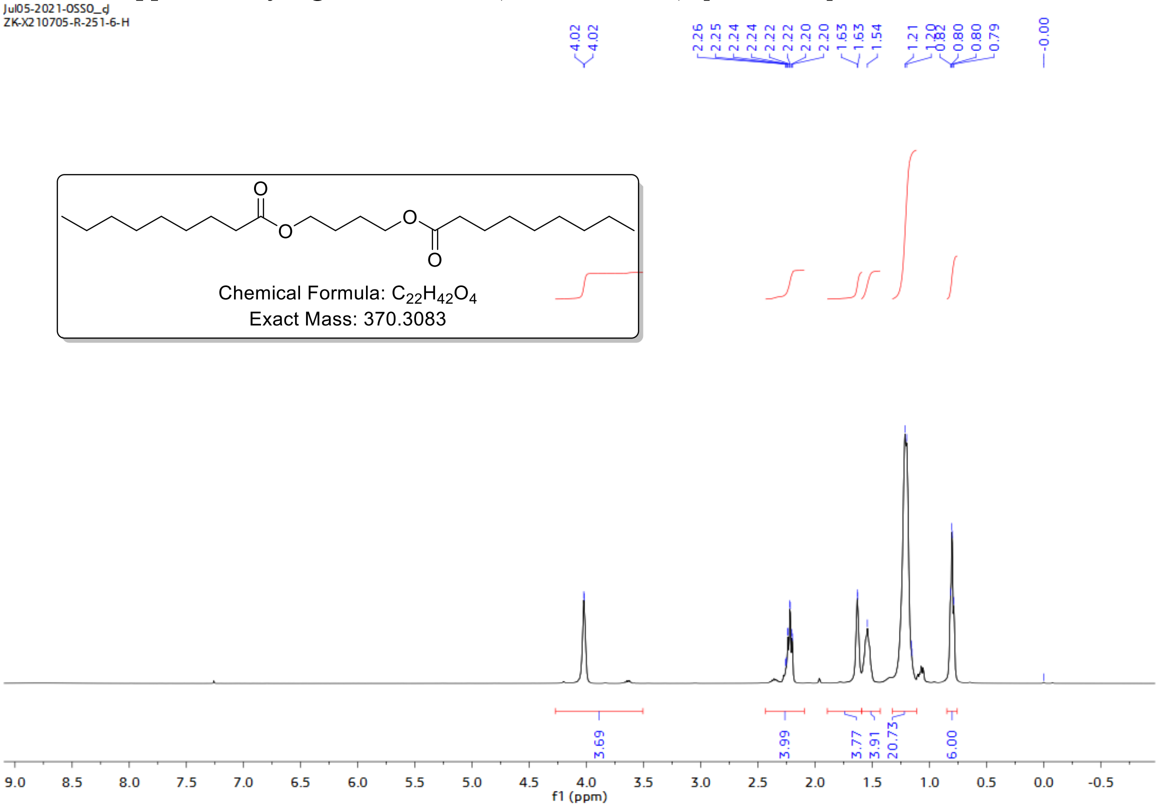

Supplementary Fig. 159 ^{1}H NMR (400 MHz, 20 ºC) spectrum of product 43 in $CDCl_3$.

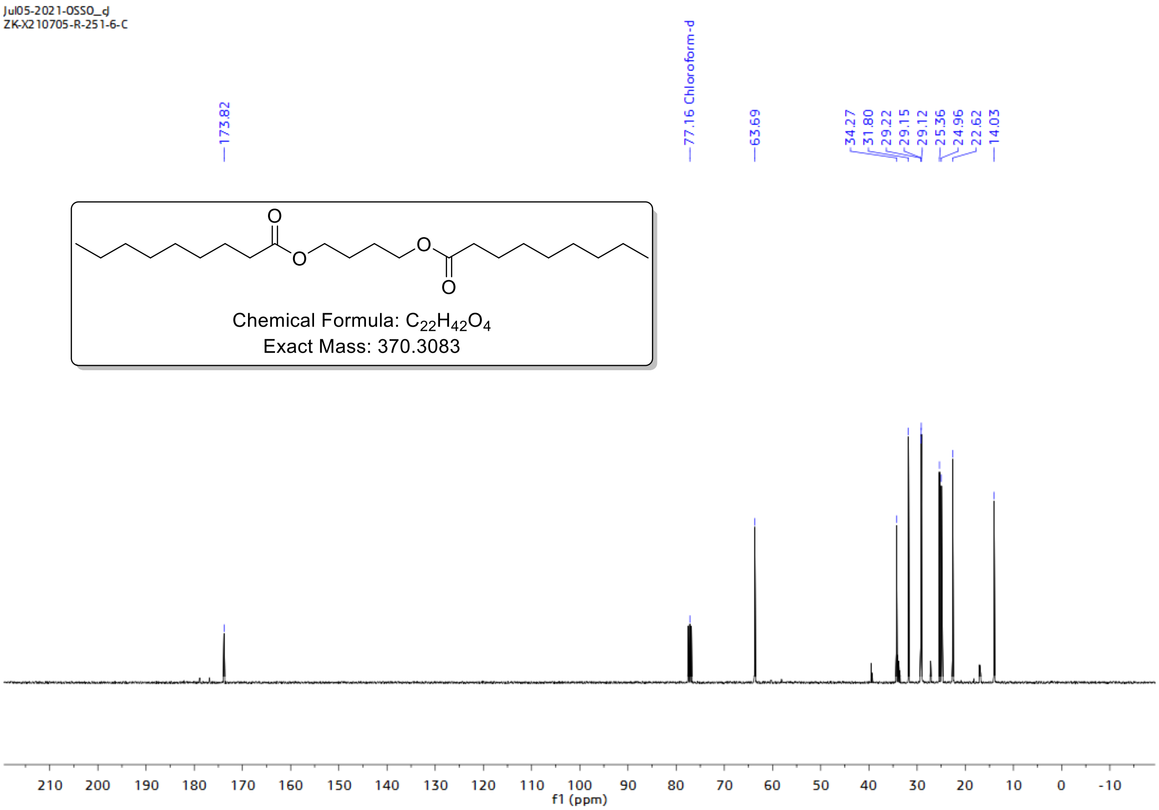

Supplementary Fig. 160 ^{13}C NMR (101 MHz, 20 ºC) spectrum of product 43 in $CDCl_3$.

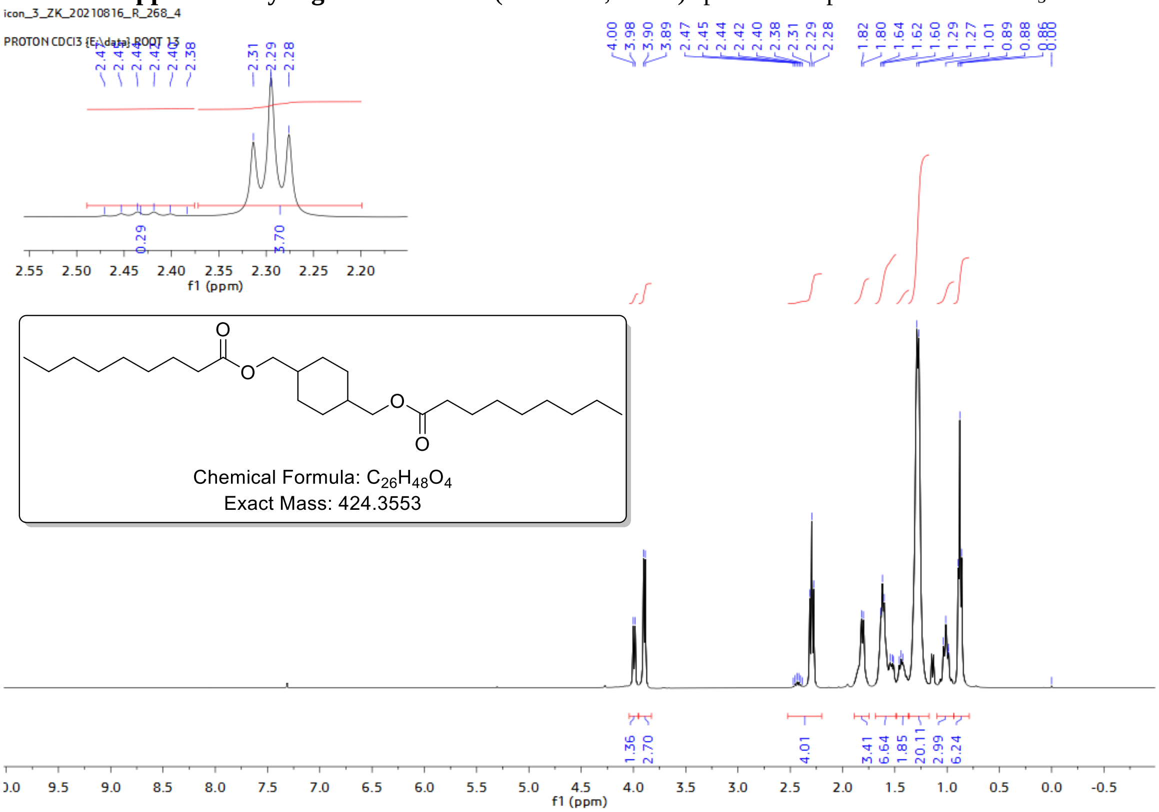

Supplementary Fig. 161 ^{1}H NMR (400 MHz, 20 ºC) spectrum of product 44 in $CDCl_3$.

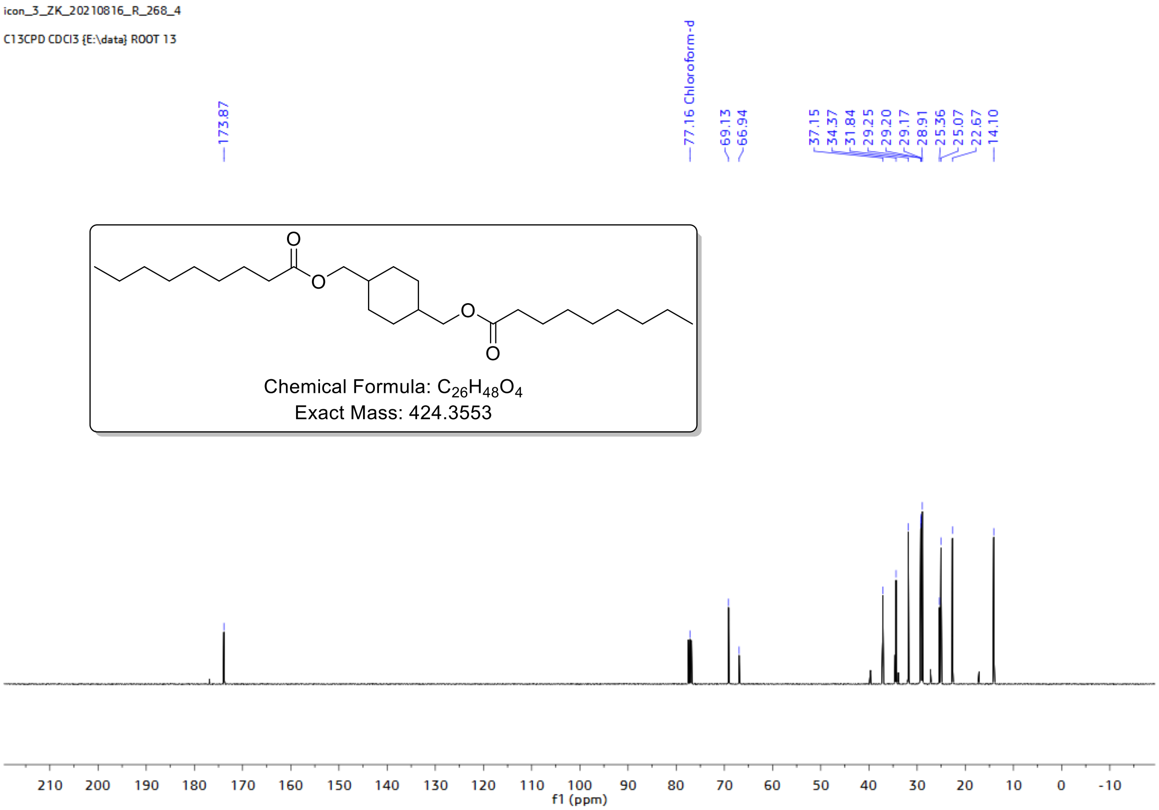

Supplementary Fig. 162 ^{13}C NMR (101 MHz, 20 ºC) spectrum of product 44 in $CDCl_3$.

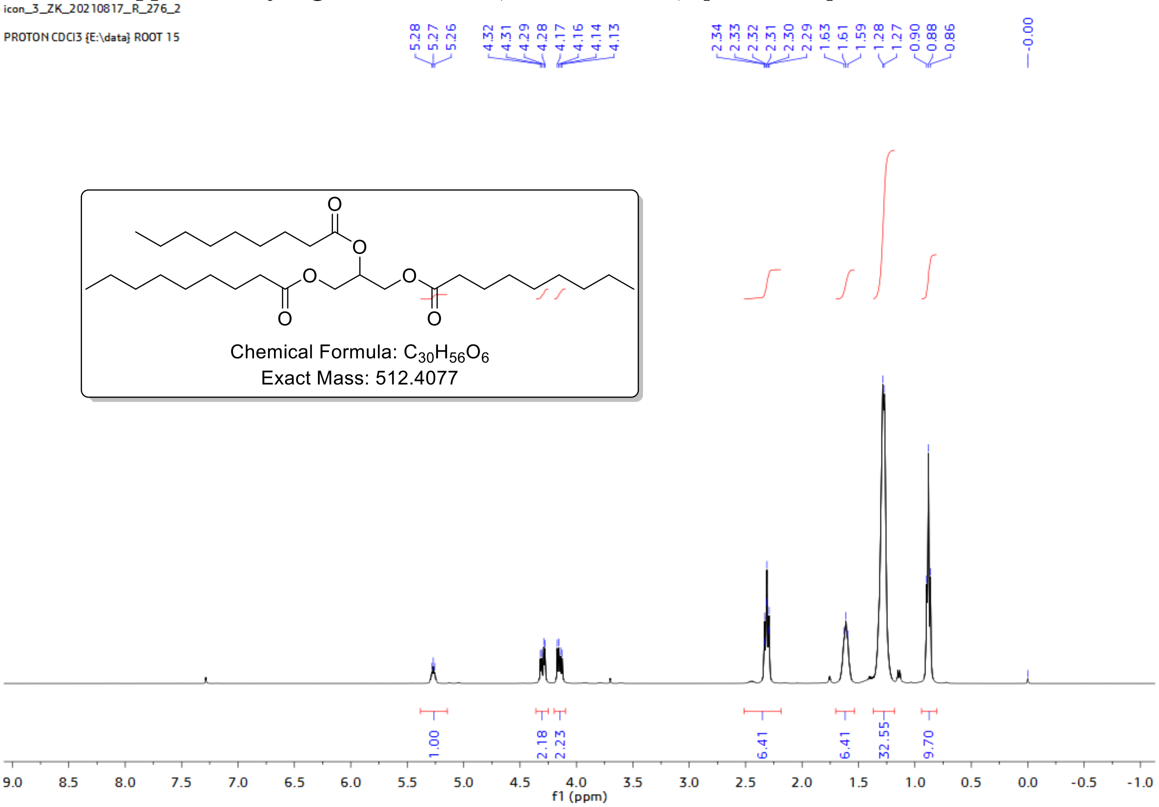

Supplementary Fig. 163 ^{1}H NMR (400 MHz, 20 ºC) spectrum of product 45 in $CDCl_3$.

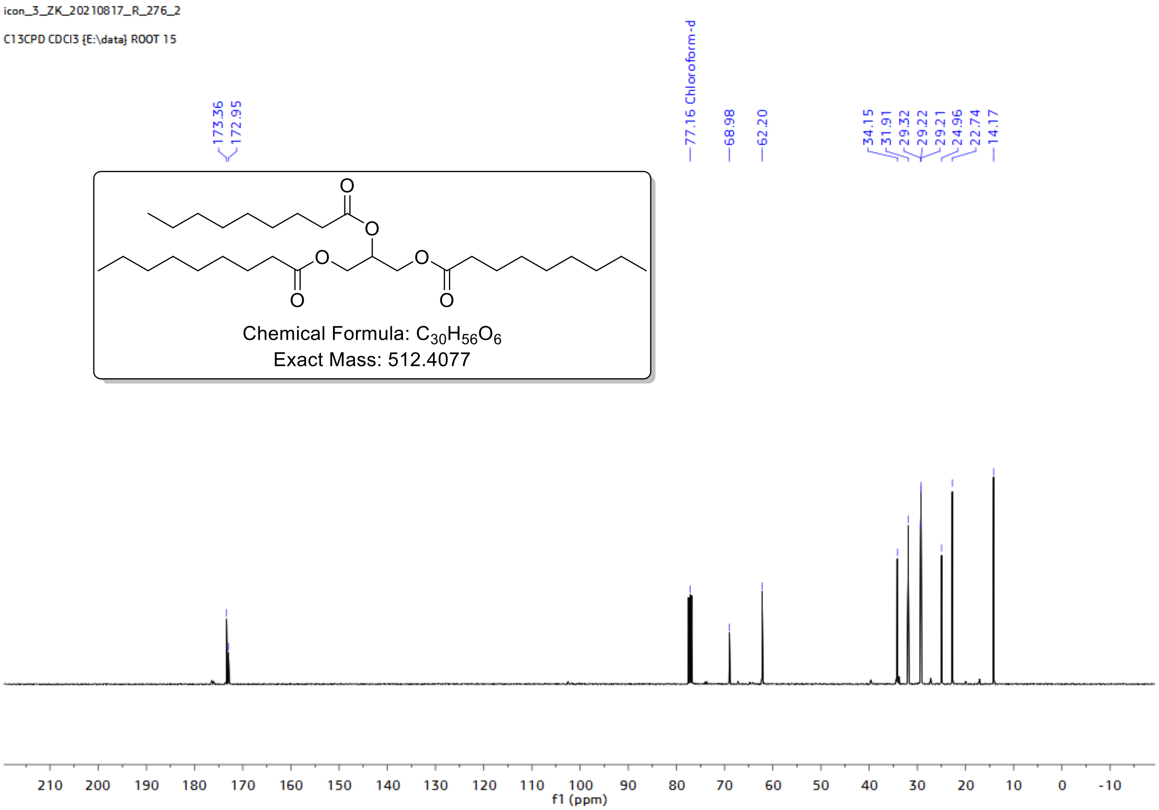

Supplementary Fig. 164 ^{13}C NMR (101 MHz, 20 °C) spectrum of product 45 in $CDCl_3$.

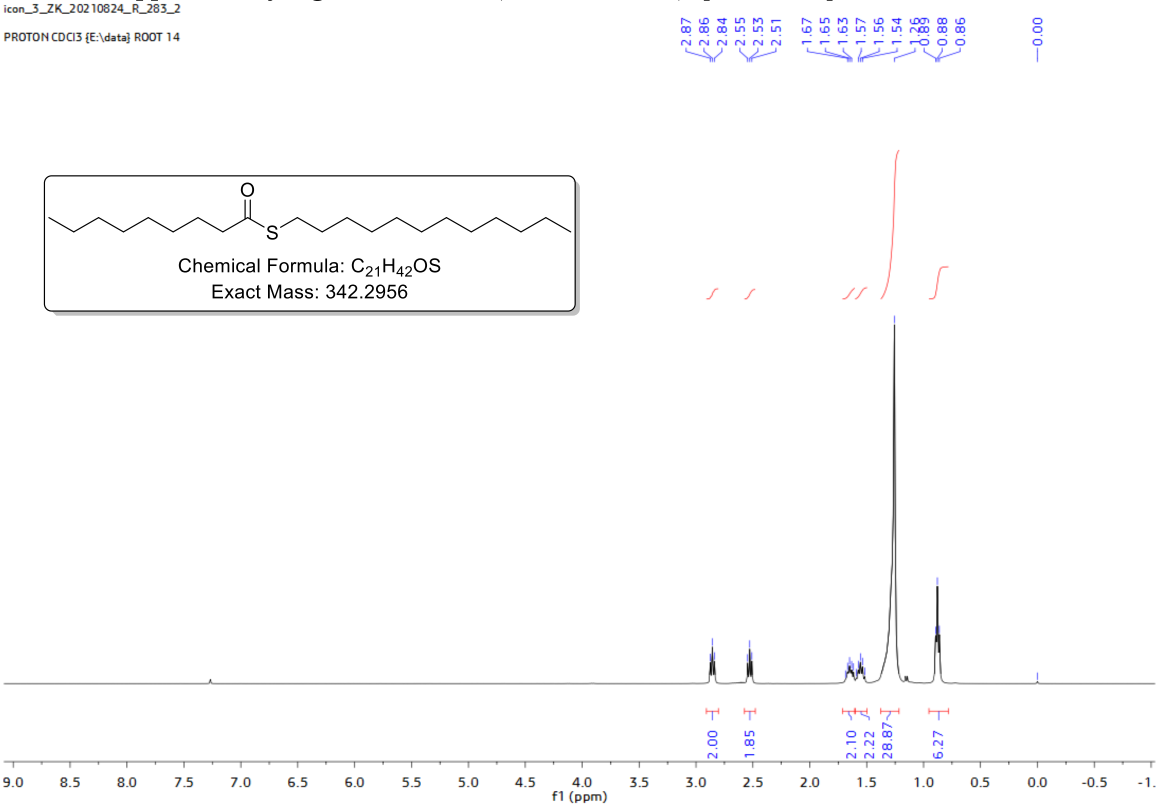

Supplementary Fig. 165 ^{1}H NMR (400 MHz, 20 °C) spectrum of product 46 in $CDCl_3$.

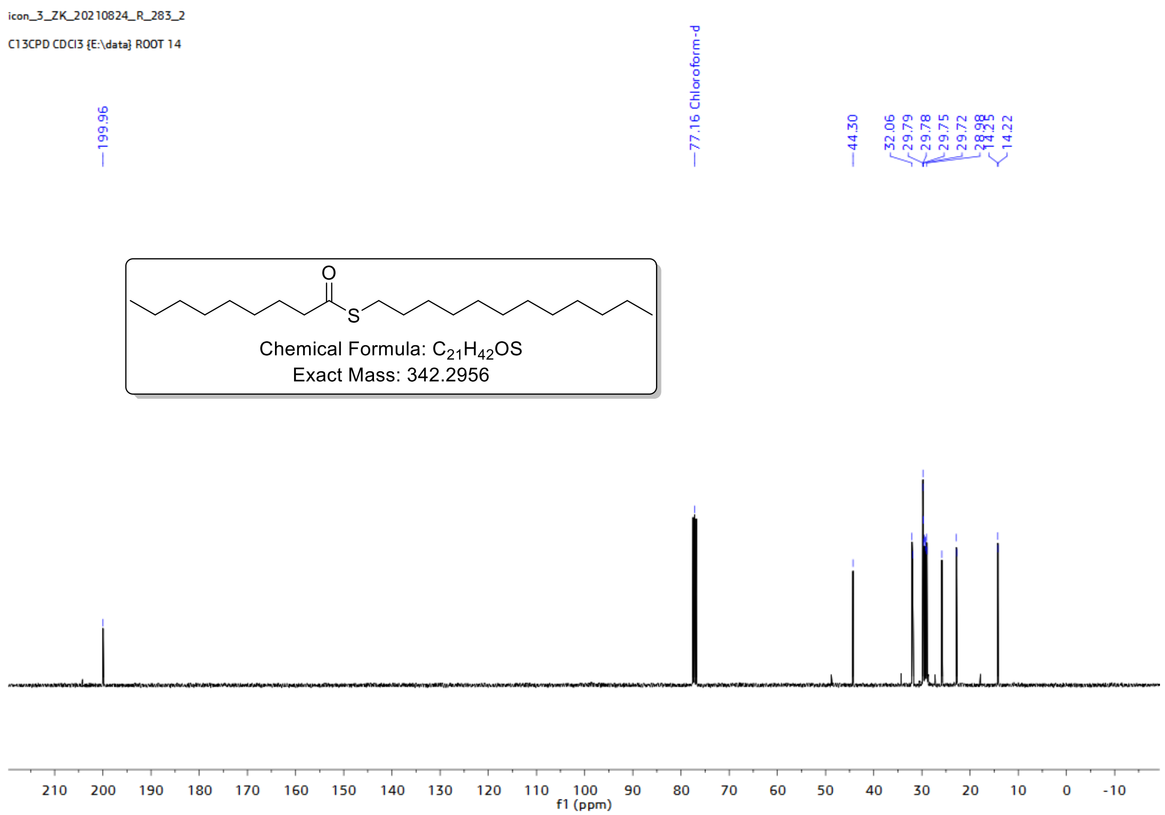

Supplementary Fig. 166 ^{13}C NMR (101 MHz, 20 ºC) spectrum of product 46 in $CDCl_3$.

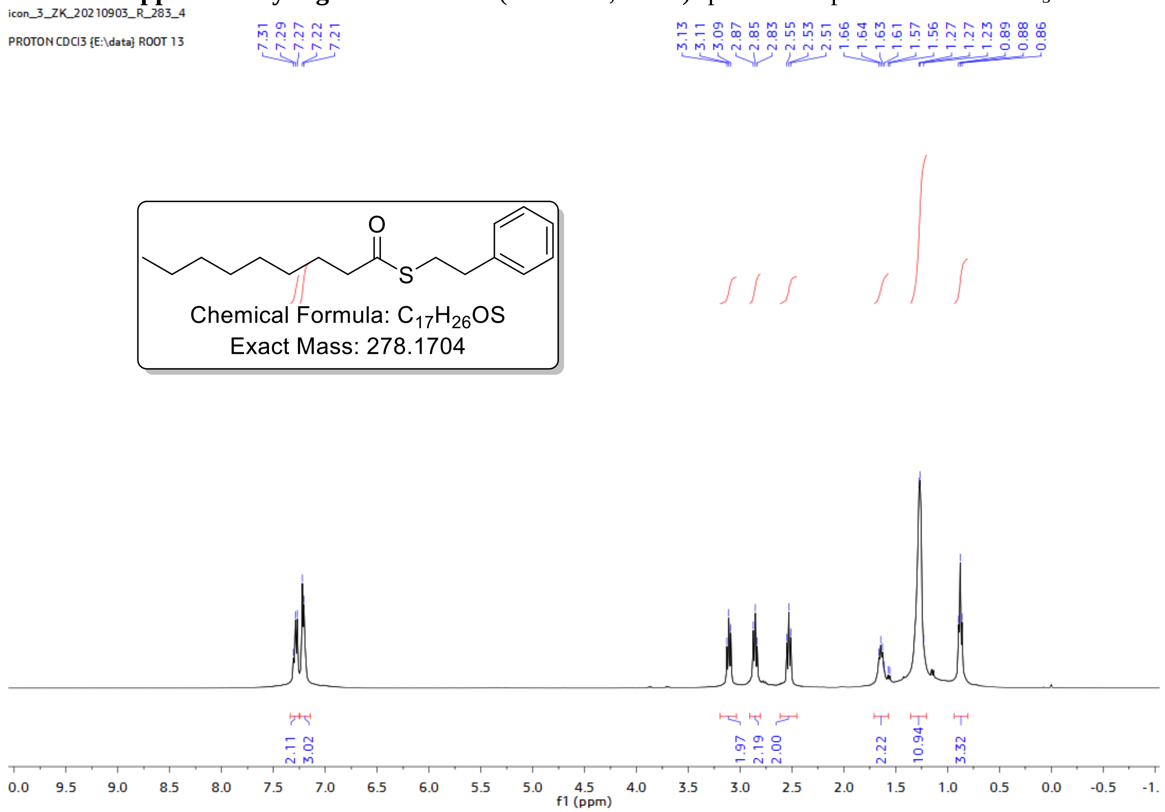

Supplementary Fig. 167 ^{1}H NMR (400 MHz, 20 ºC) spectrum of product 47 in $CDCl_3$.

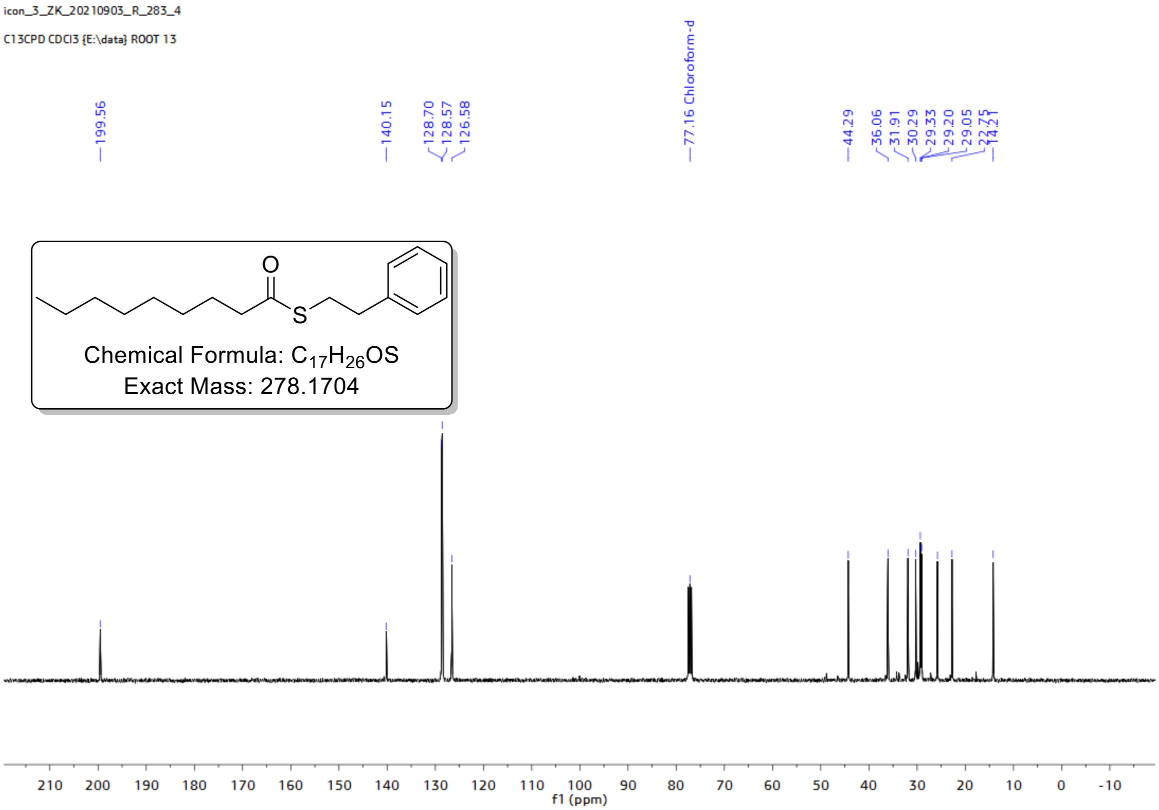

Supplementary Fig. 168 ^{13}C NMR (101 MHz, 20 °C) spectrum of product 47 in $CDCl_3$.

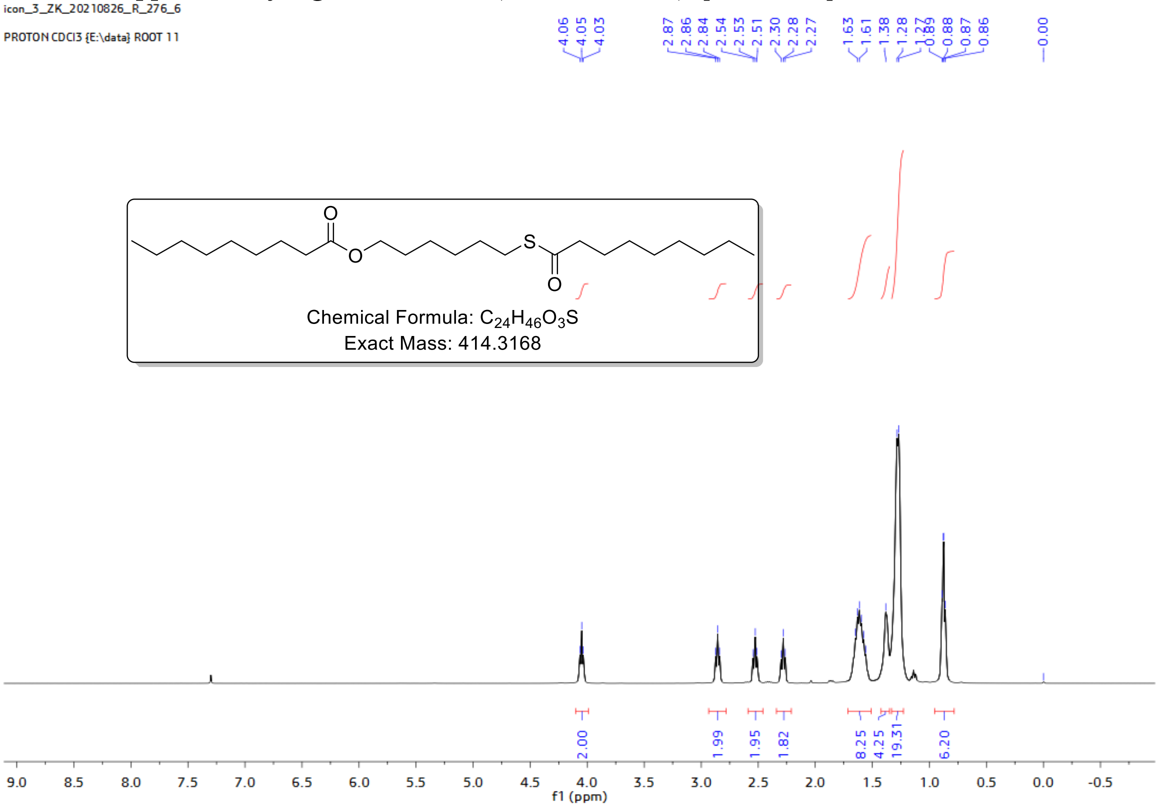

Supplementary Fig. 169^{1}H NMR (400 MHz, 20 °C) spectrum of product 48 in $CDCl_3$.

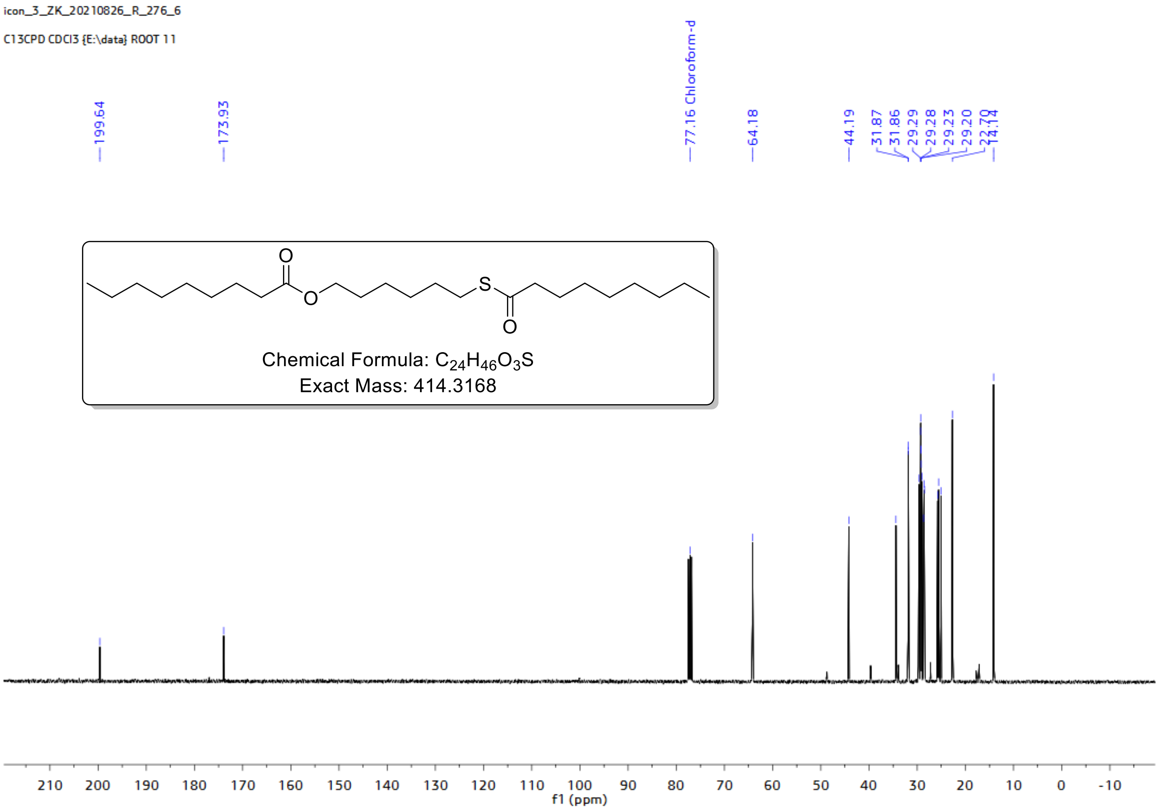

Supplementary Fig. 170 ^{13}C NMR (101 MHz, 20 ºC) spectrum of product 48 in $CDCl_3$.

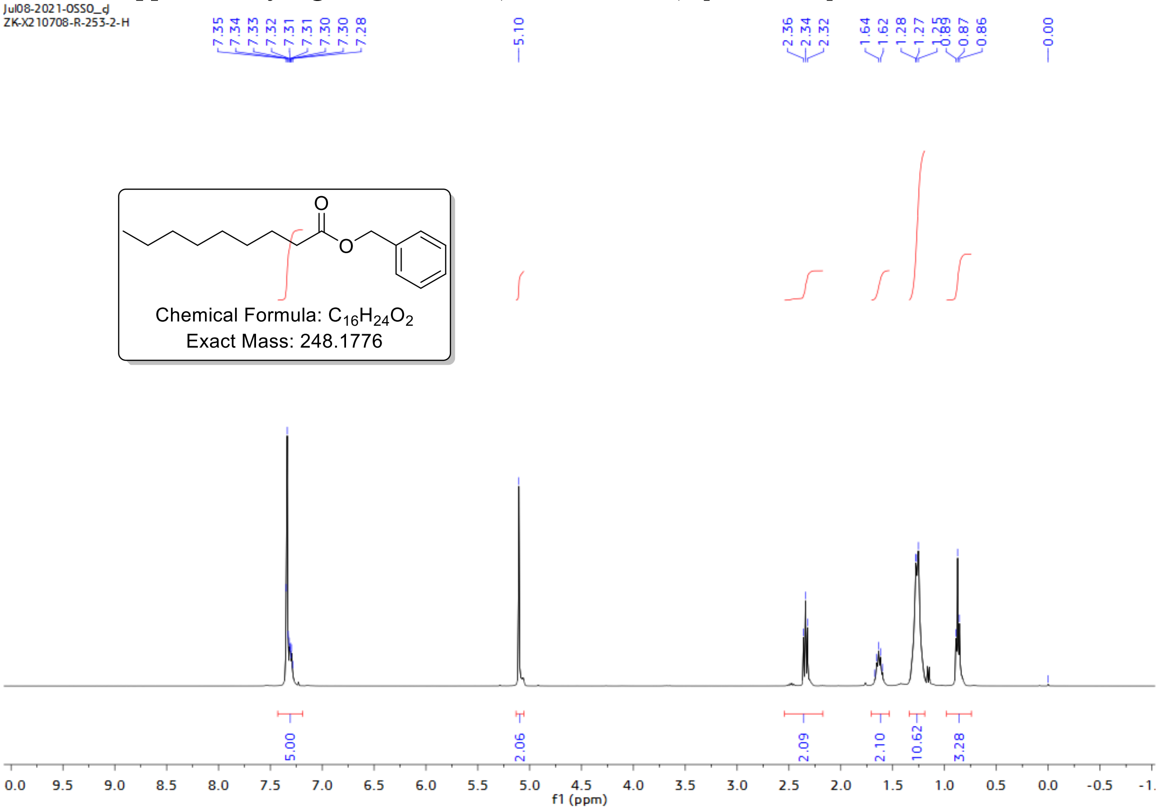

Supplementary Fig. 171 ^{1}H NMR (400 MHz, 20 ºC) spectrum of product 49 in $CDCl_3$.

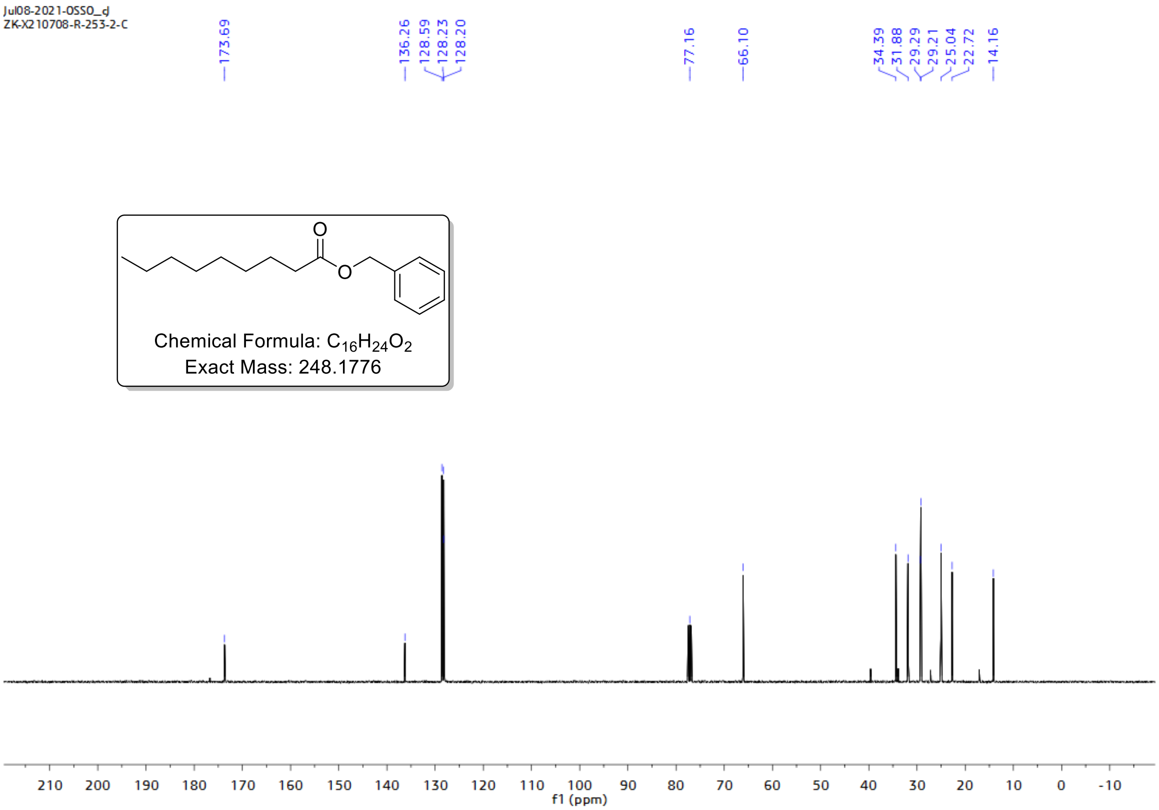

Supplementary Fig. 172 ^{13}C NMR (101 MHz, 20 ºC) spectrum of product 49 in $CDCl_3$.

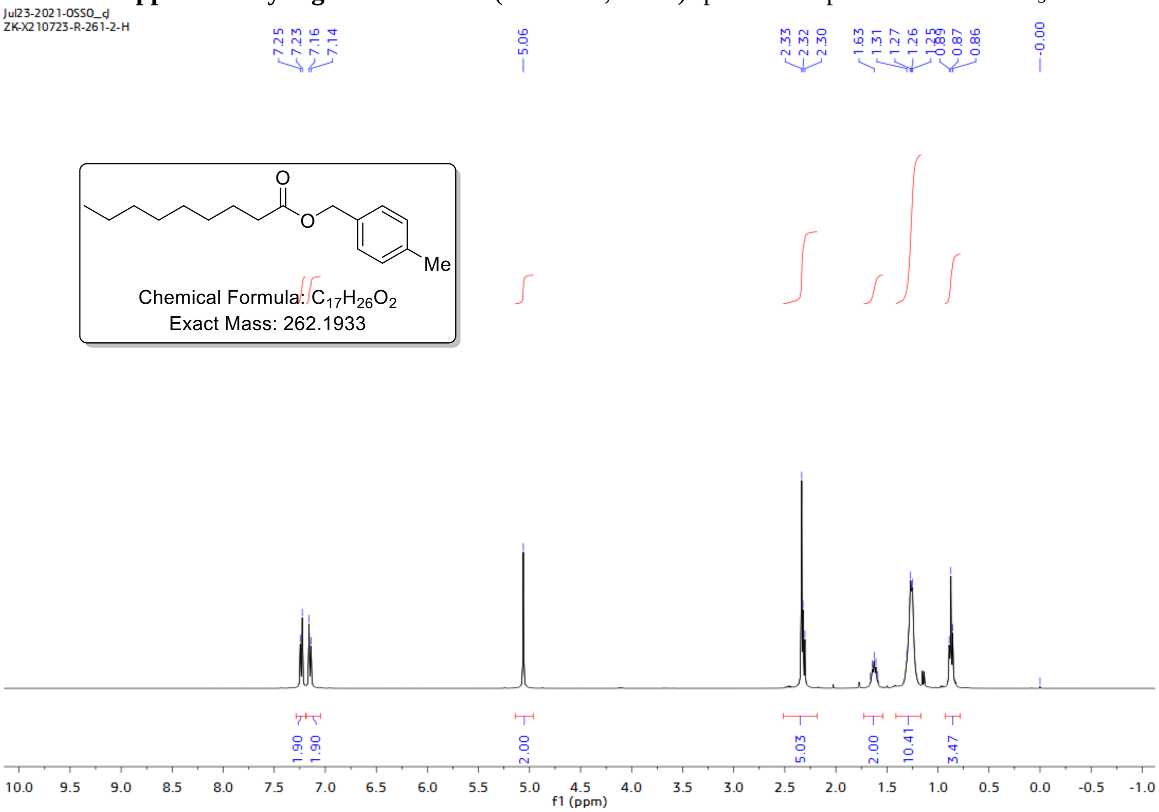

Supplementary Fig. 173 ^{1}H NMR (400 MHz, 20 ºC) spectrum of product 50 in $CDCl_3$.

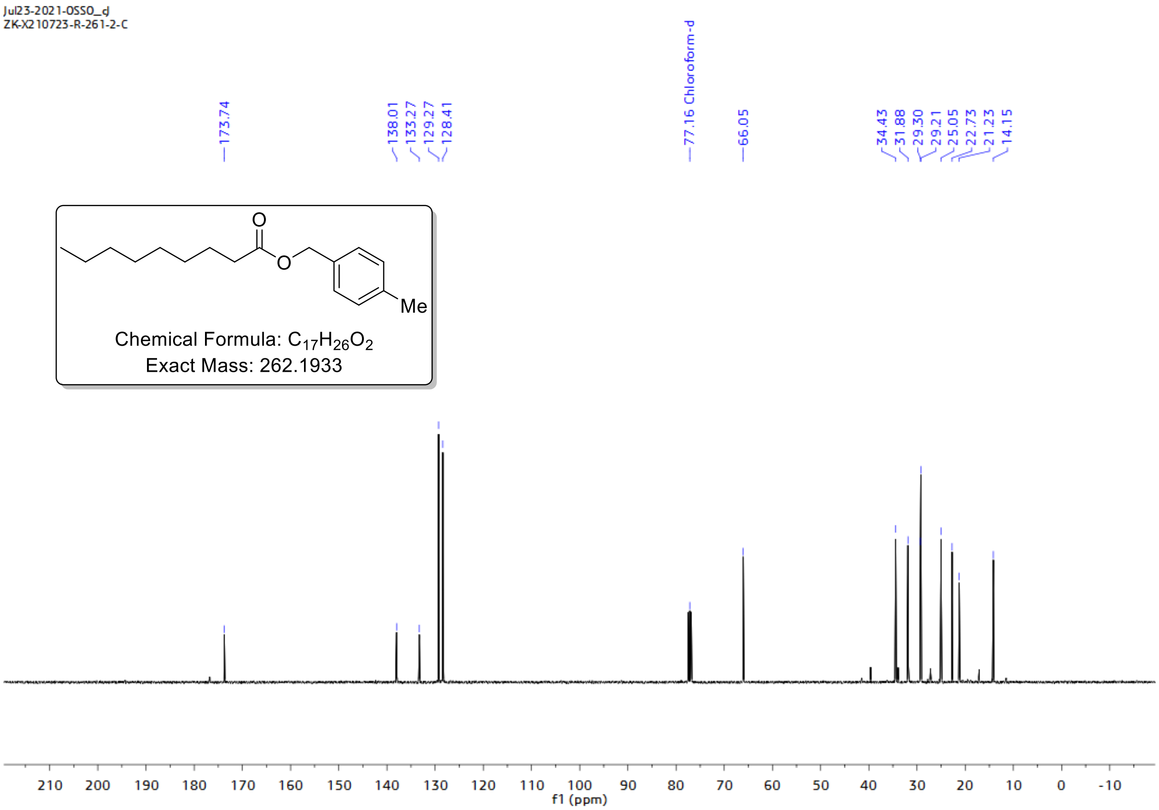

Supplementary Fig. 174 ^{13}C NMR (101 MHz, 20 ºC) spectrum of product 50 in $CDCl_3$.

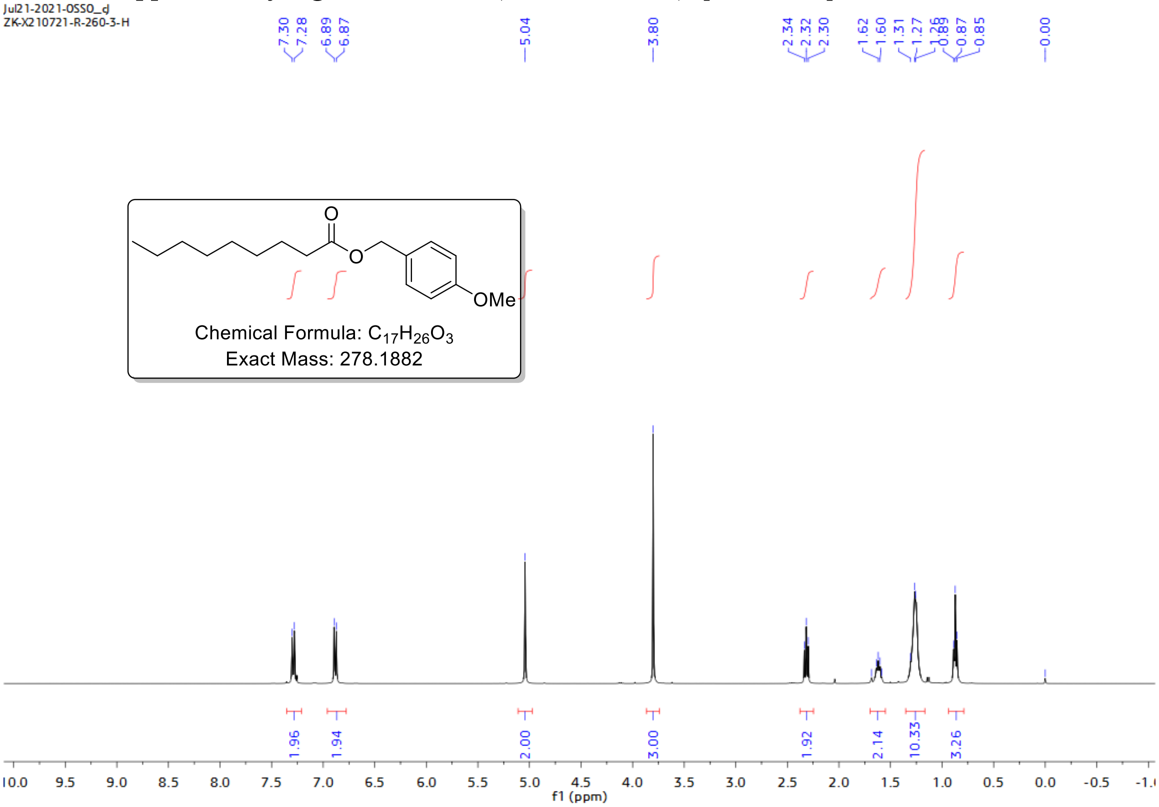

Supplementary Fig. 175 ^{1}H NMR (400 MHz, 20 ºC) spectrum of product 51 in $CDCl_3$.

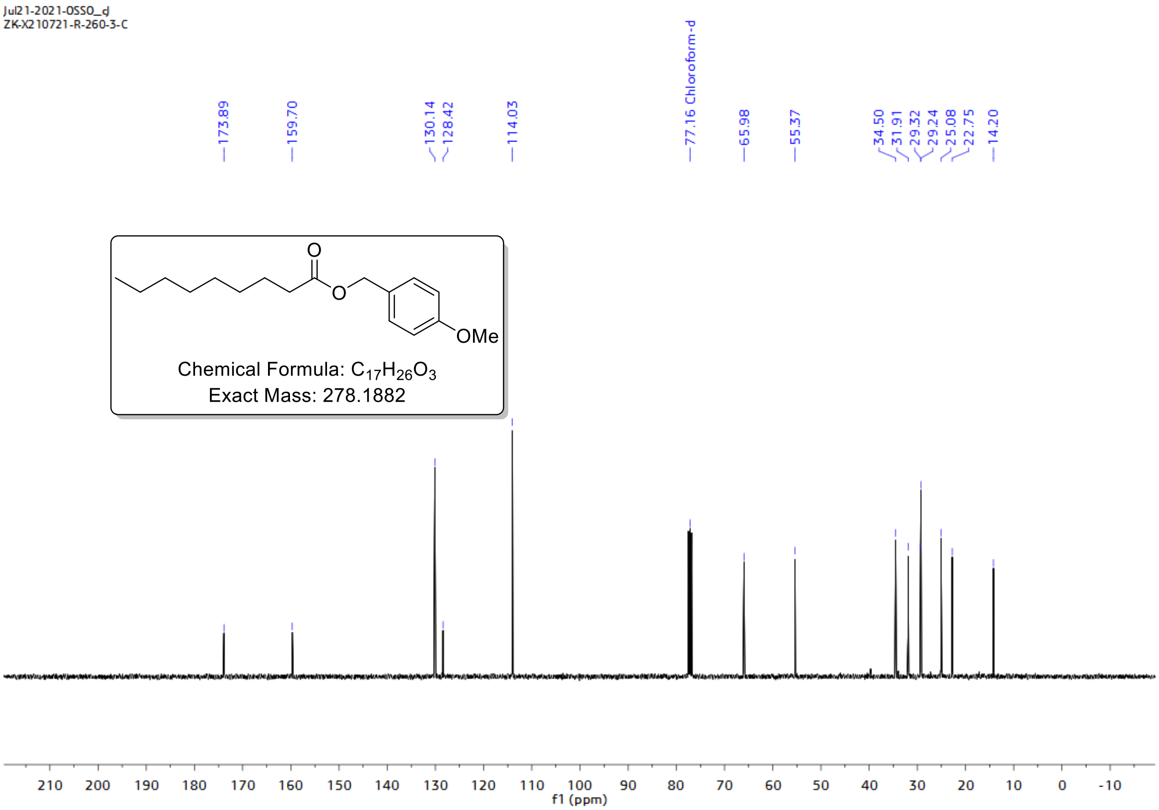

Supplementary Fig. 176 ^{13}C NMR (101 MHz, 20 ºC) spectrum of product 51 in $CDCl_3$.

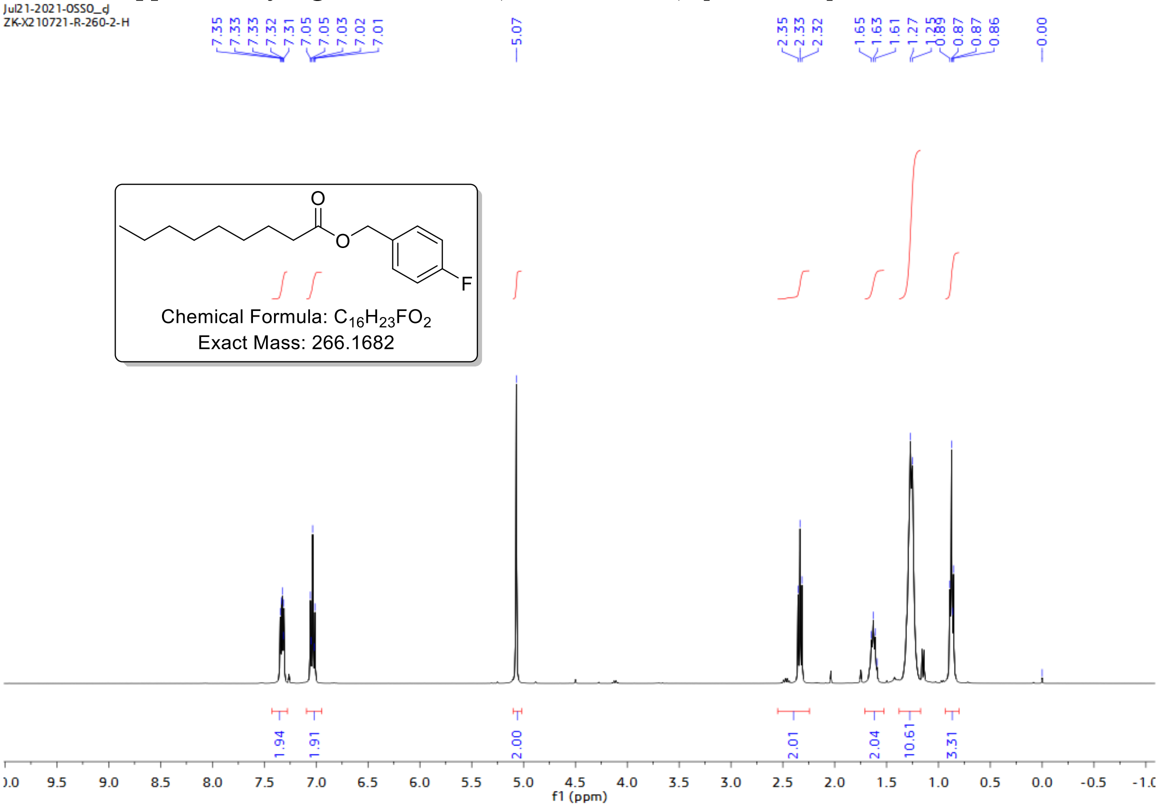

Supplementary Fig. 177 ^{1}H NMR (400 MHz, 20 ºC) spectrum of product 52 in $CDCl_3$.

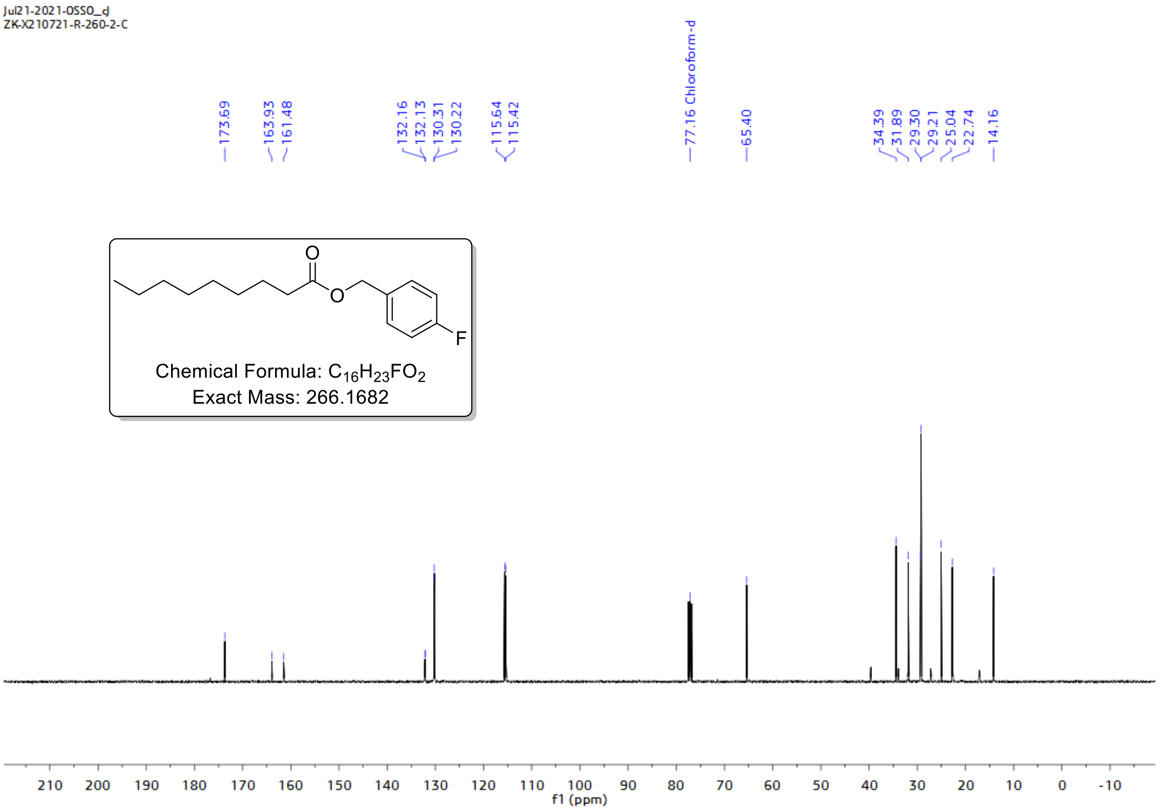

Supplementary Fig. 178 ^{13}C NMR (101 MHz, 20 ºC) spectrum of product 52 in $CDCl_3$.

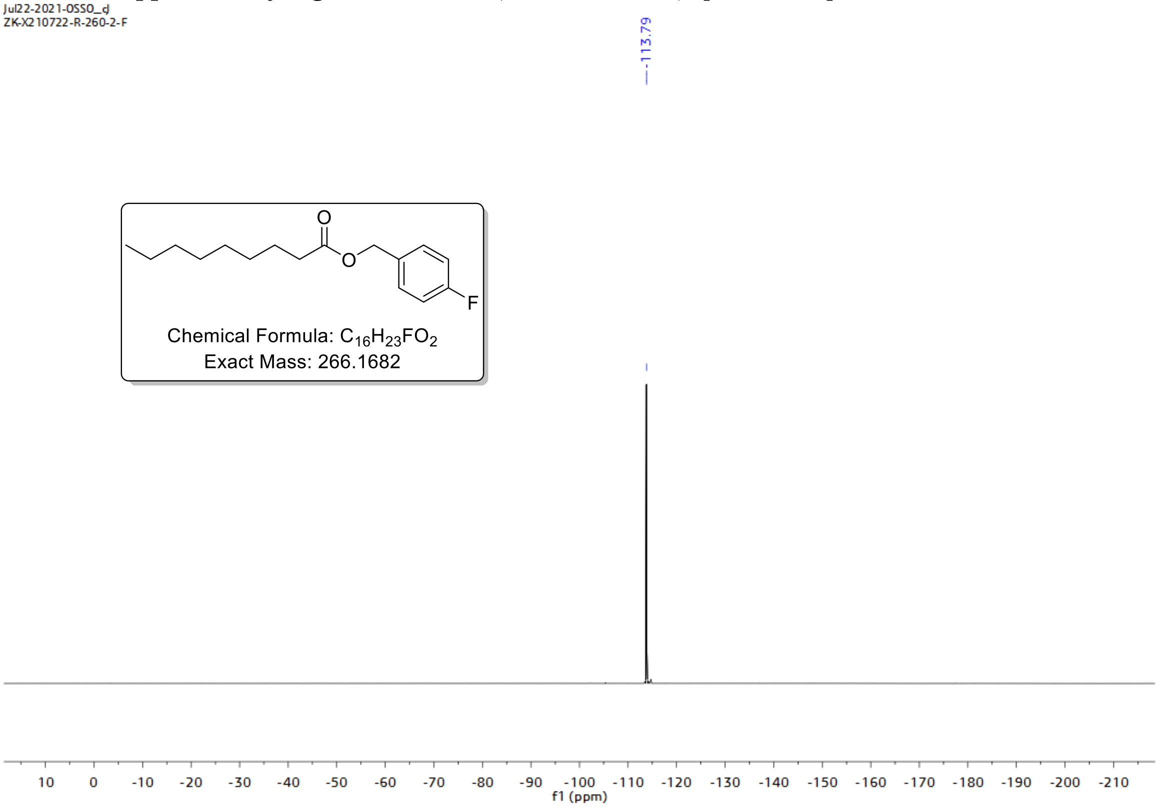

Supplementary Fig. 179 ^{19}F NMR (376 MHz, 20 ºC) spectrum of product 52 in $CDCl_3$.

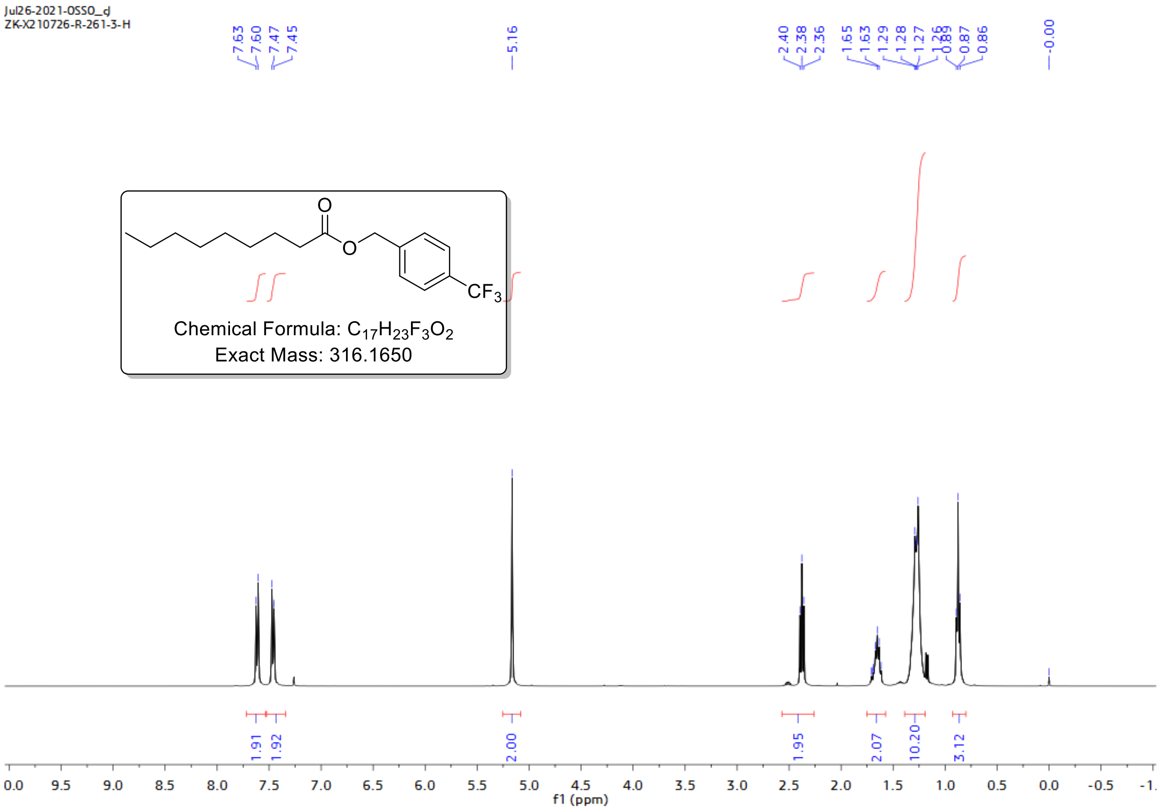

Supplementary Fig. 180 ^{1}H NMR (400 MHz, 20 ºC) spectrum of product 53 in $CDCl_3$.

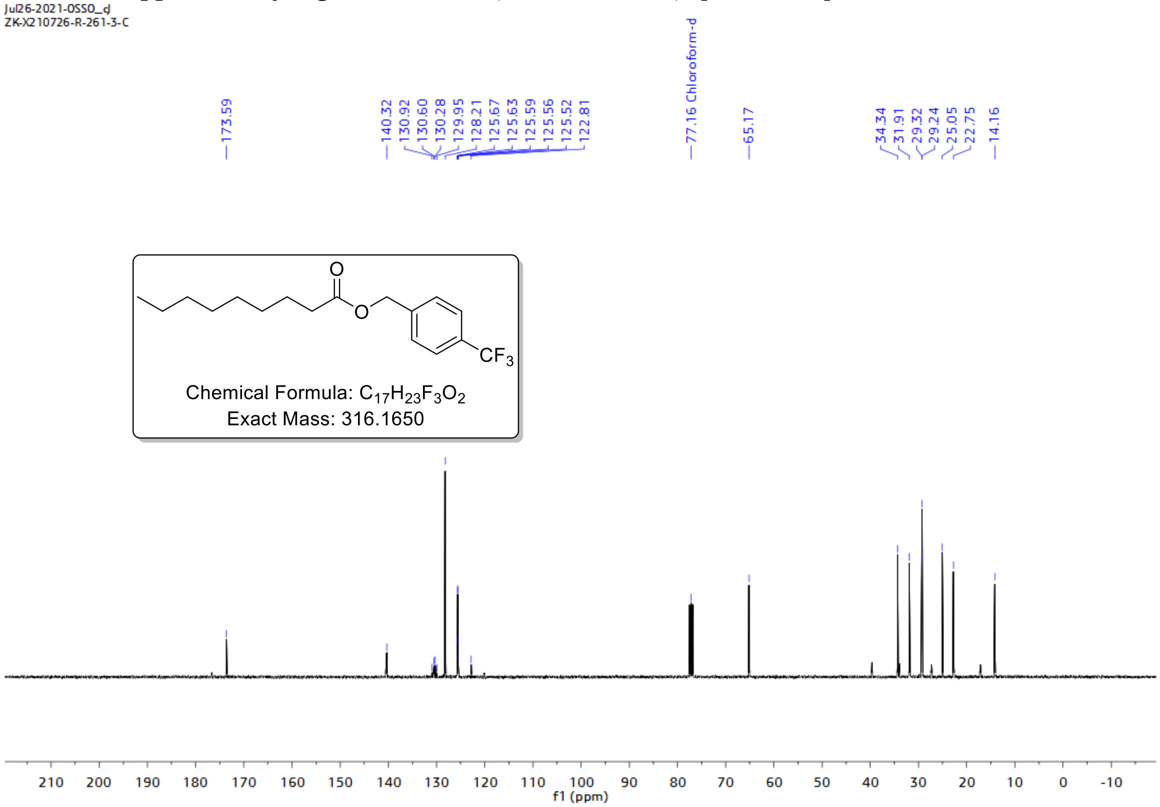

Supplementary Fig. 181 ^{13}C NMR (101 MHz, 20 ºC) spectrum of product 53 in $CDCl_3$.

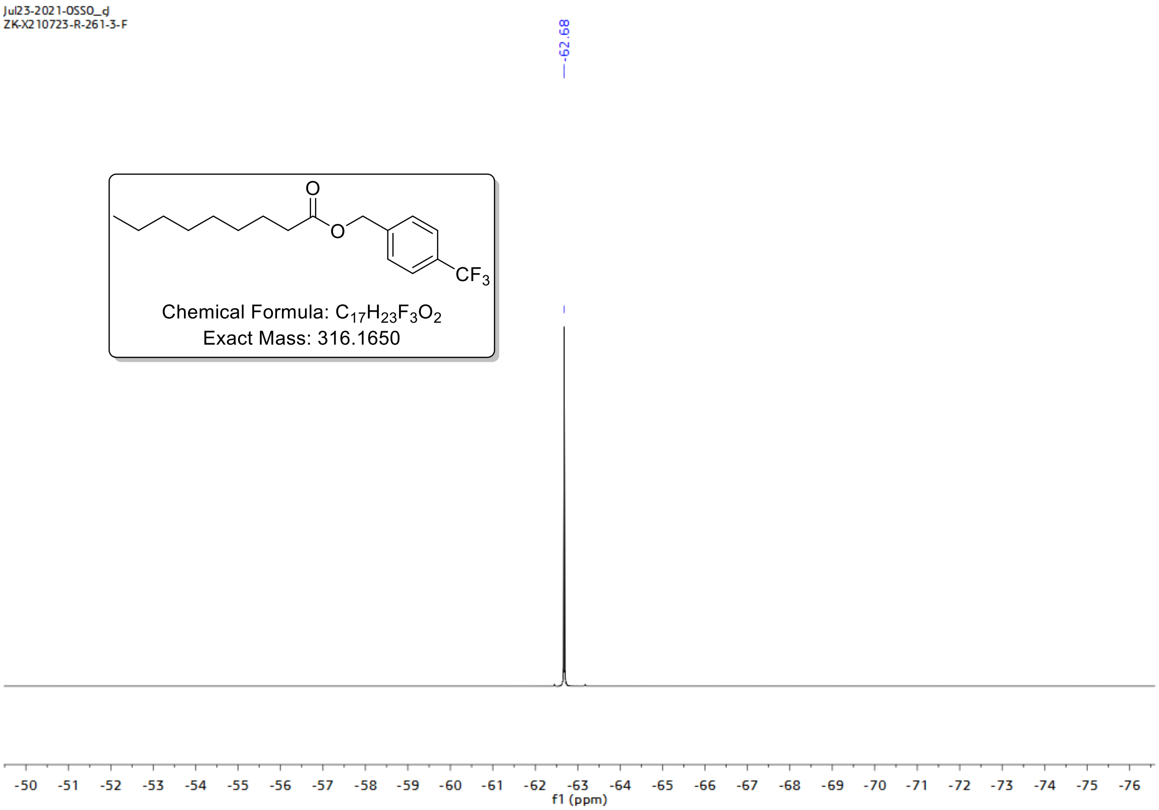

Supplementary Fig. 182 ^{19}F NMR (376 MHz, 20 ºC) spectrum of product 53 in $CDCl_3$.

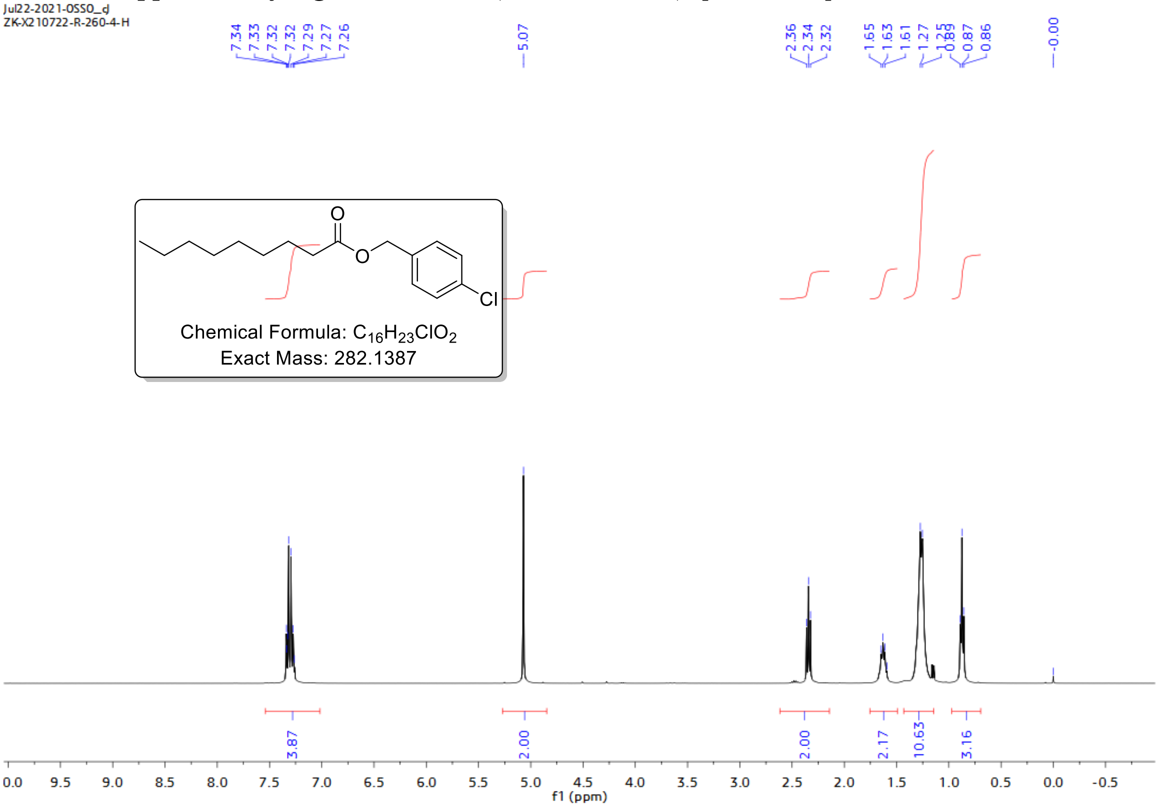

Supplementary Fig. 183 ^{1}H NMR (400 MHz, 20 ºC) spectrum of product 54 in $CDCl_3$.

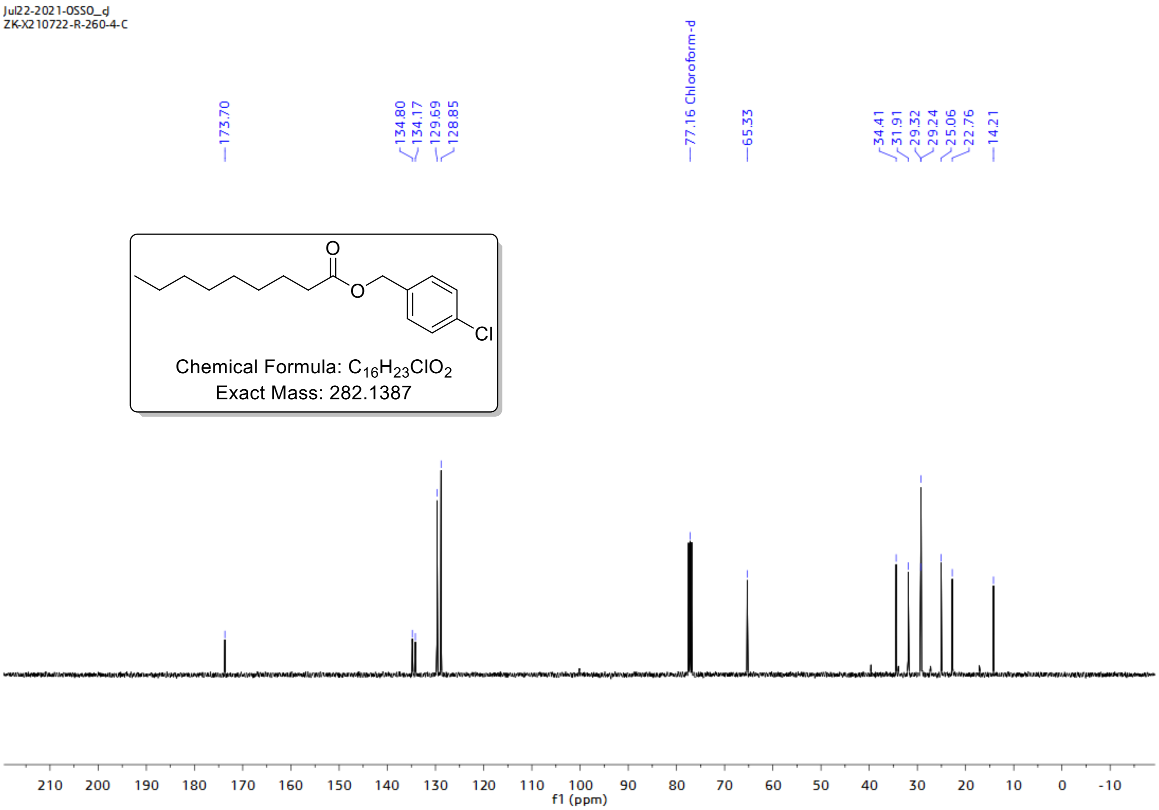

Supplementary Fig. 184 ^{13}C NMR (101 MHz, 20 °C) spectrum of product 54 in $CDCl_3$.

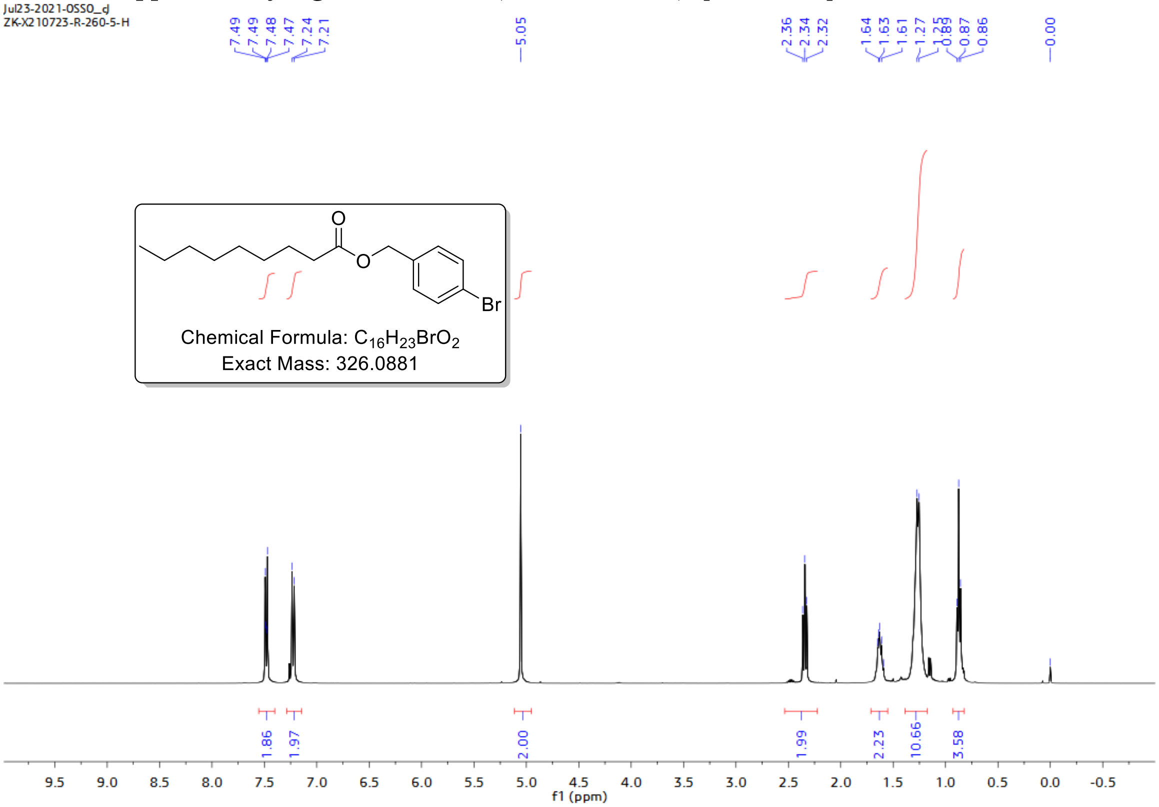

Supplementary Fig. 185 ^{1}H NMR (400 MHz, 20 °C) spectrum of product 55 in $CDCl_3$.

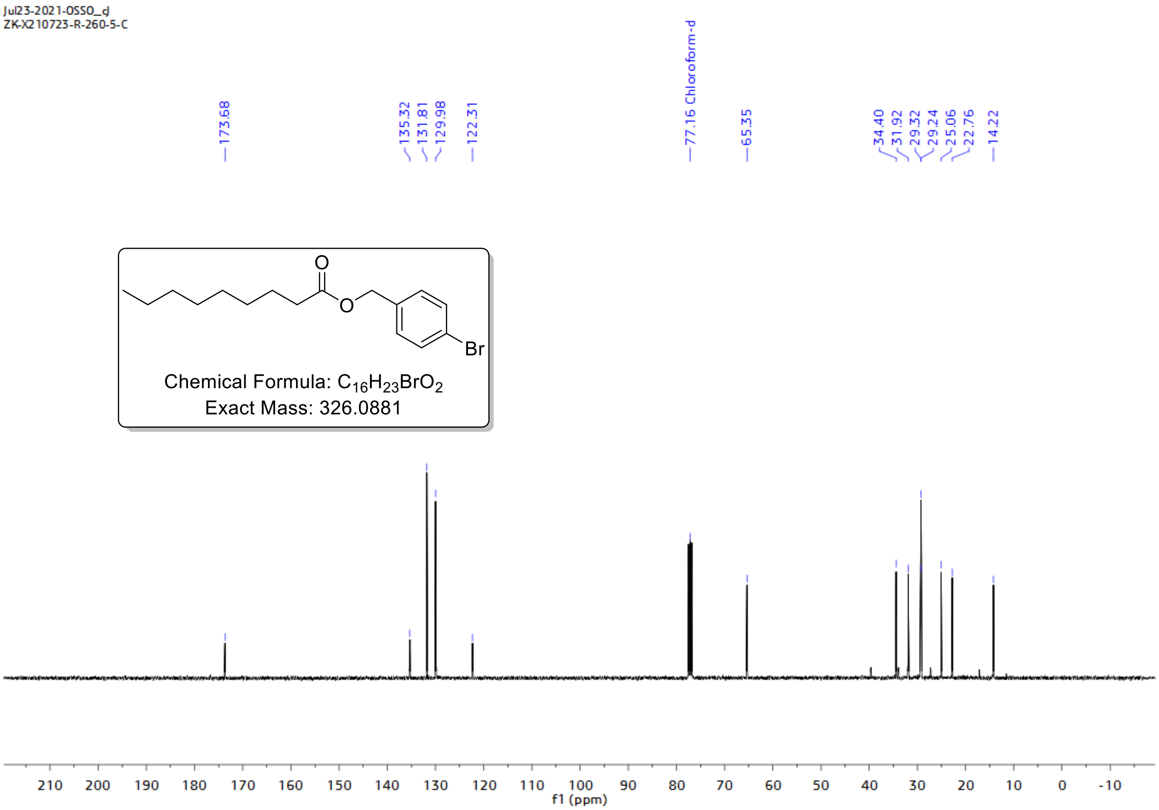

Supplementary Fig. 186 ^{13}C NMR (101 MHz, 20 °C) spectrum of product 55 in $CDCl_3$.

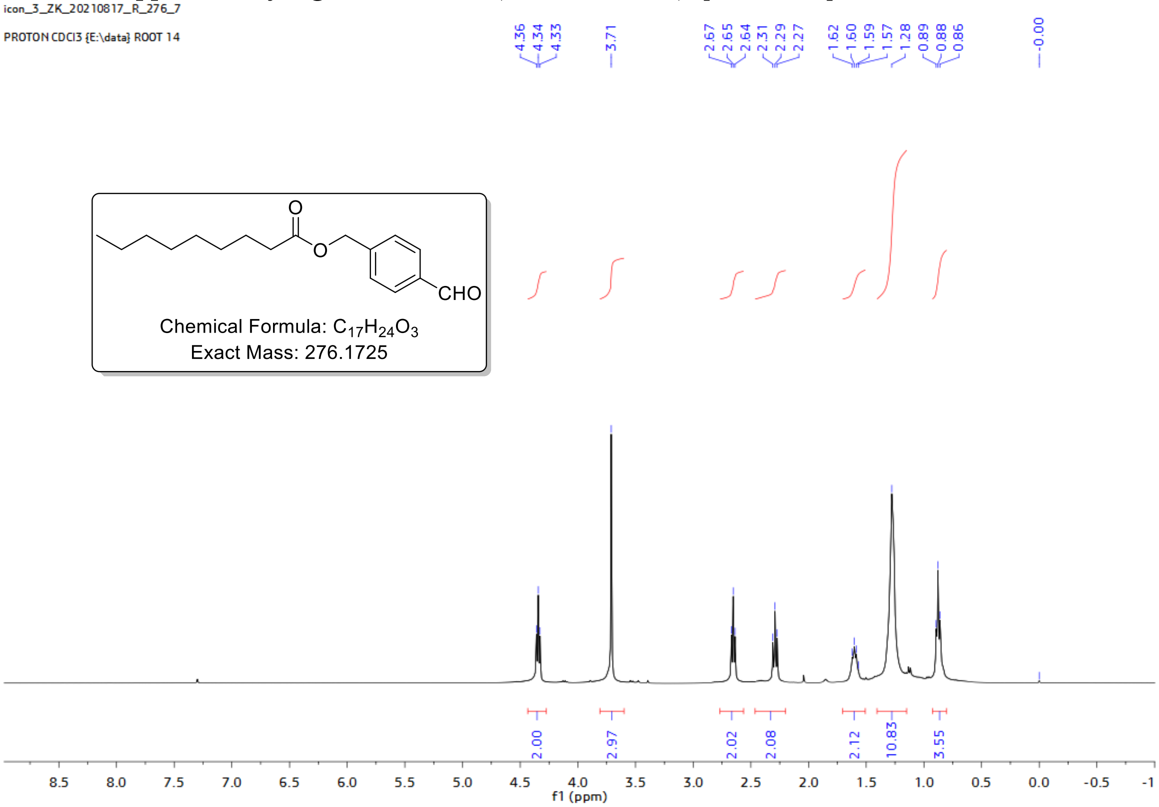

Supplementary Fig. 187 ^{1}H NMR (400 MHz, 20 °C) spectrum of product 56 in $CDCl_3$.

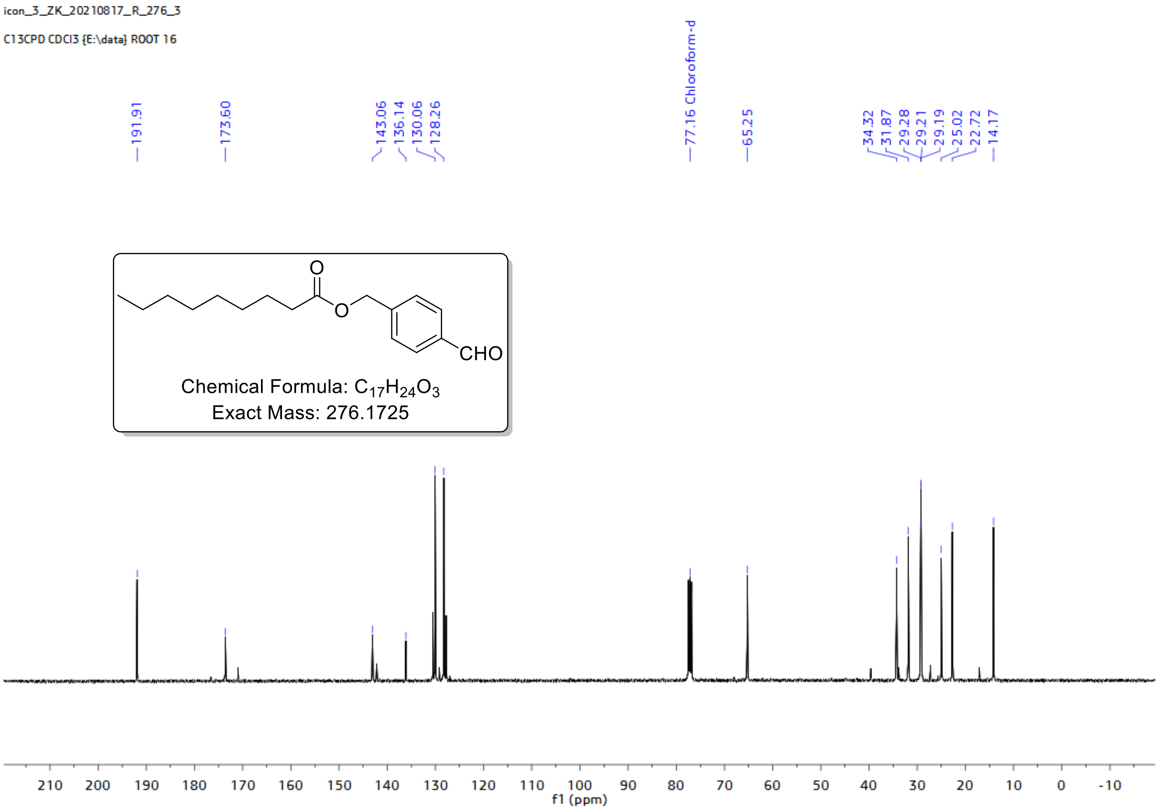

Supplementary Fig. 188 ^{13}C NMR (101 MHz, 20 ºC) spectrum of product 56 in $CDCl_3$.

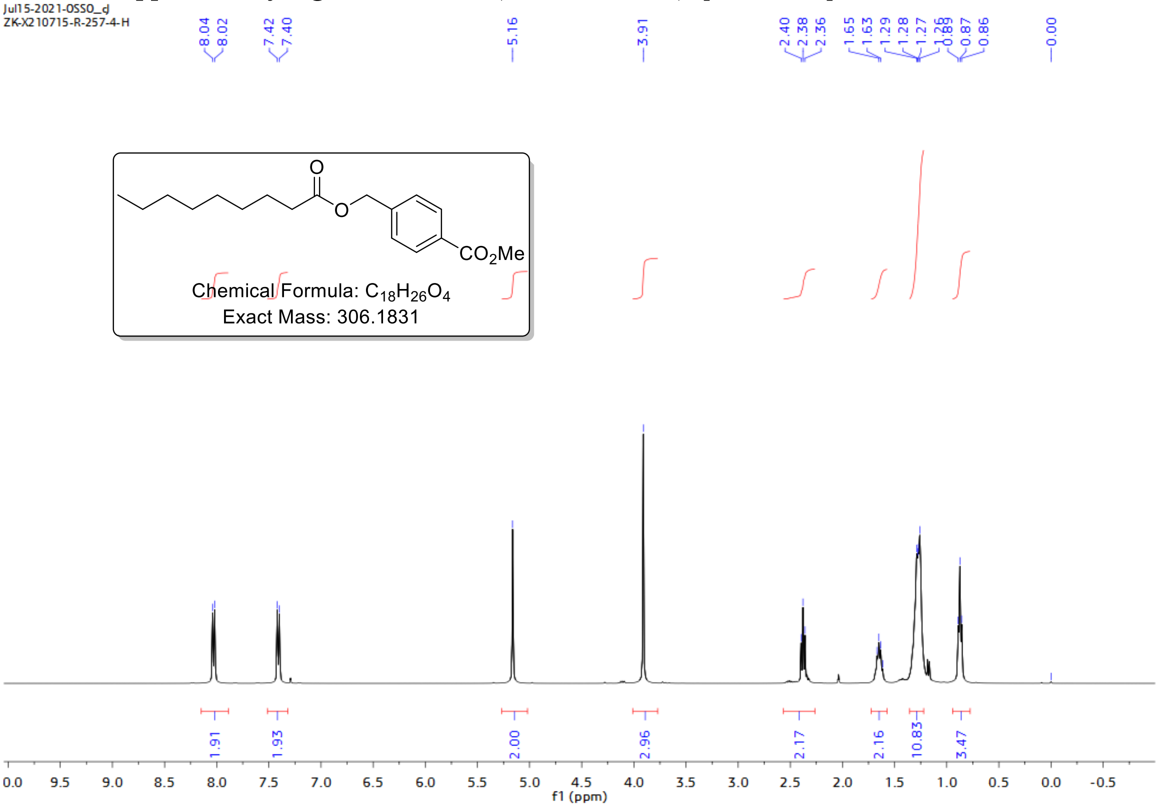

Supplementary Fig. 189 ^{1}H NMR (400 MHz, 20 ºC) spectrum of product 57 in $CDCl_3$.

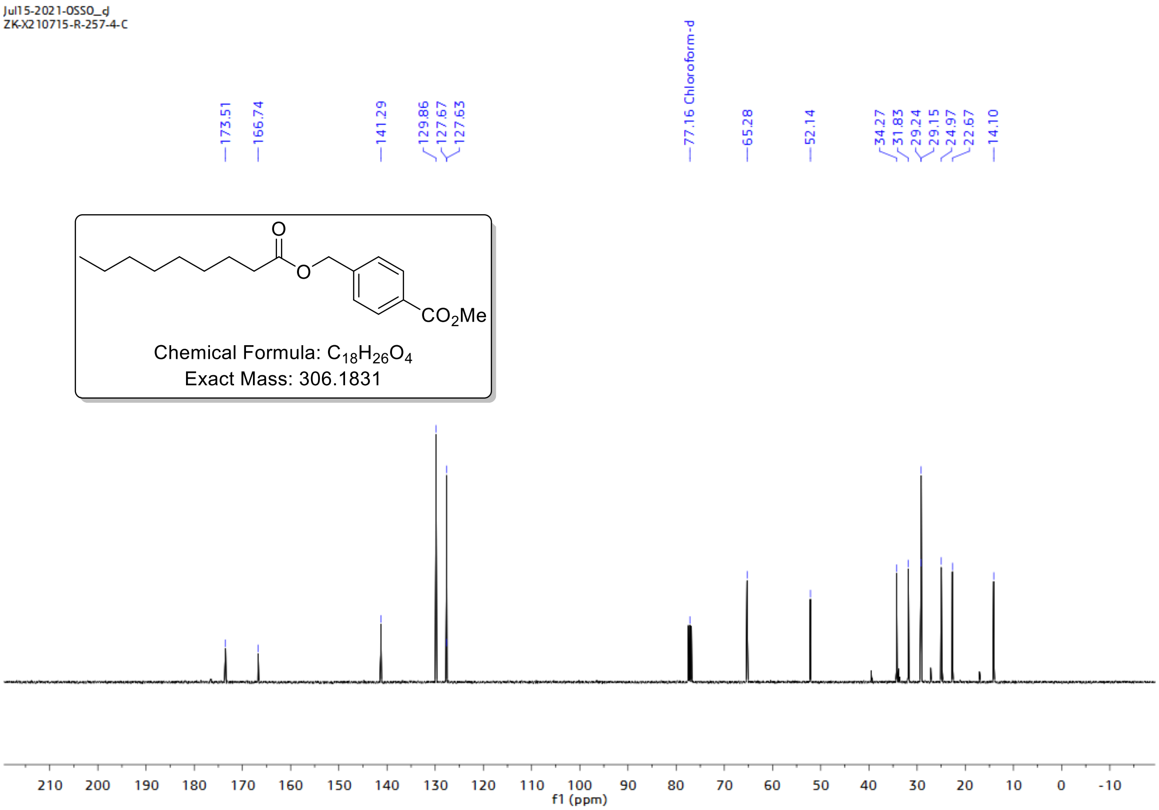

Supplementary Fig. 190 ^{13}C NMR (101 MHz, 20 ºC) spectrum of product 57 in $CDCl_3$.

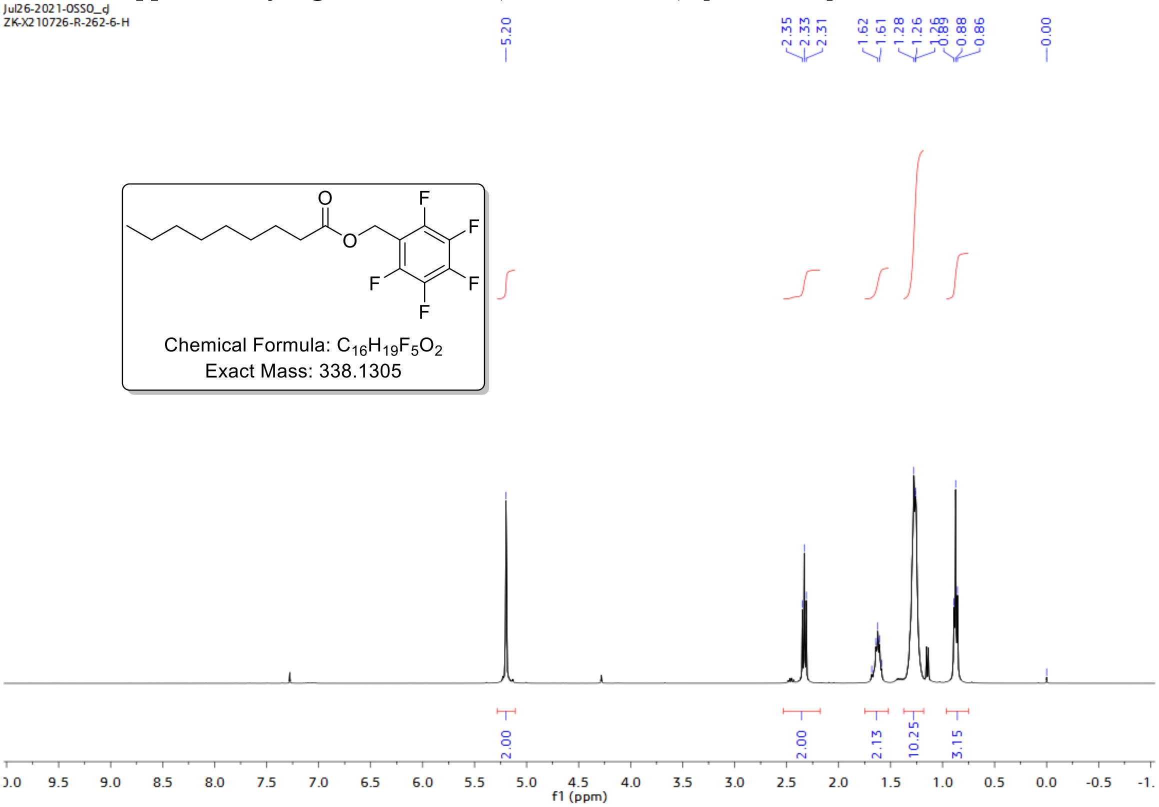

Supplementary Fig. 191 ^{1}H NMR (400 MHz, 20 ºC) spectrum of product 58 in $CDCl_3$.

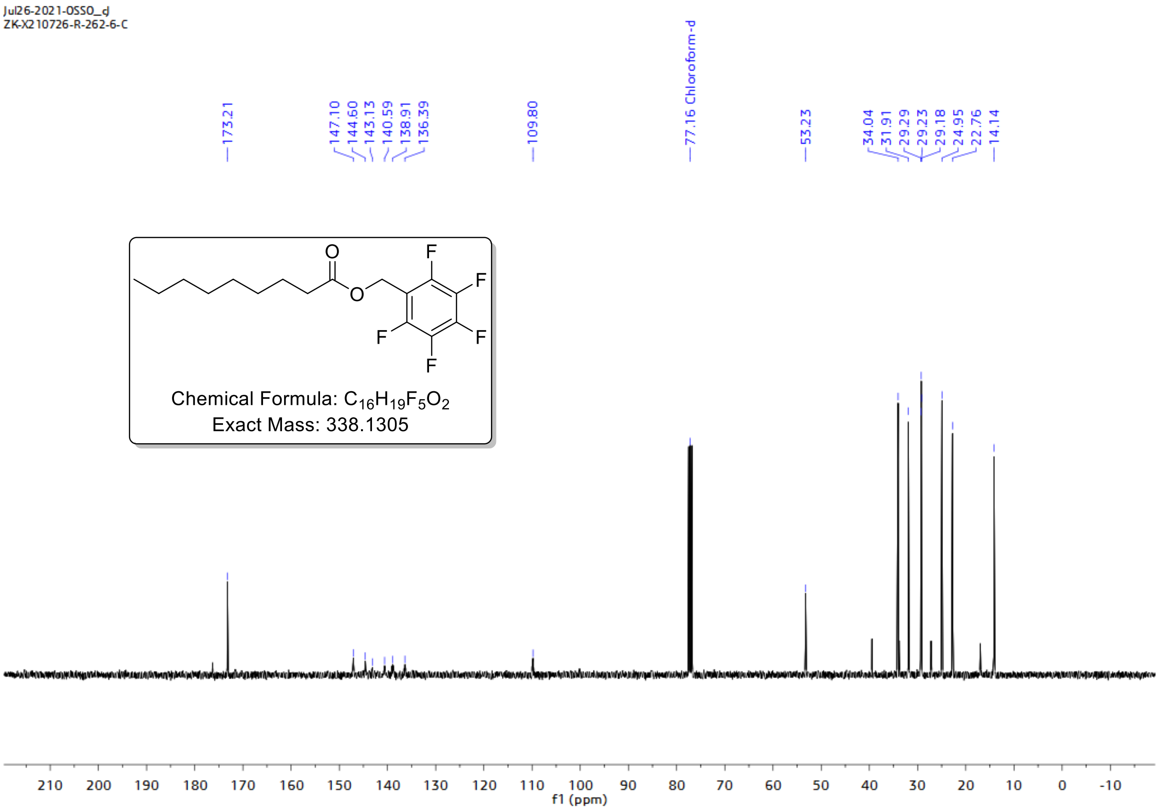

Supplementary Fig. 192 ^{13}C NMR (101 MHz, 20 ºC) spectrum of product 58 in $CDCl_3$.

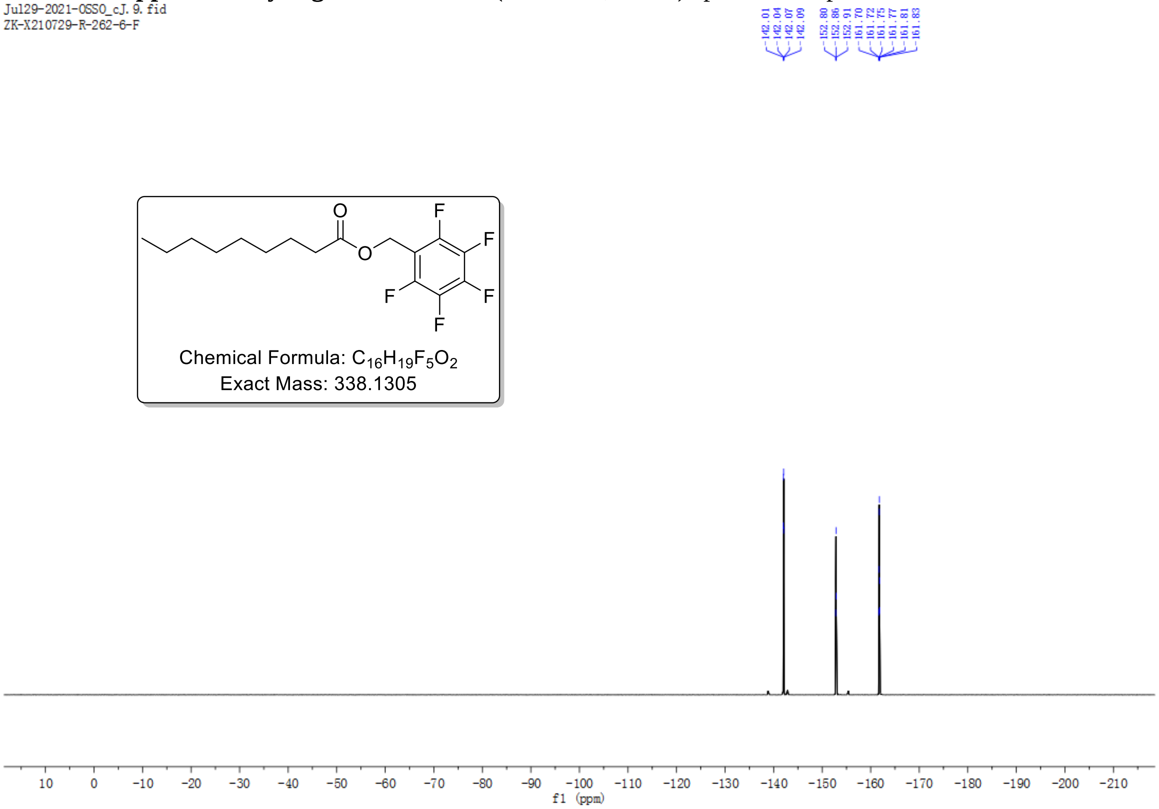

Supplementary Fig. 193 ^{19}F NMR (376 MHz, 20 ºC) spectrum of product 58 in $CDCl_3$.

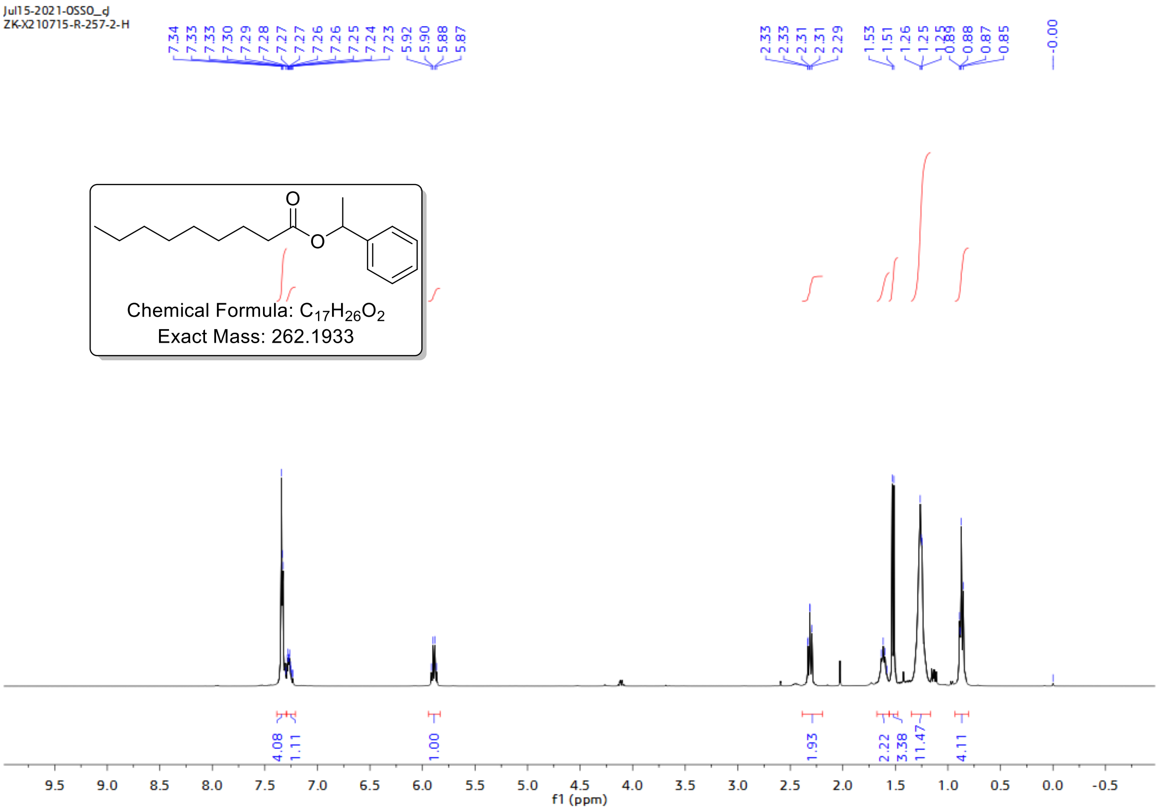

Supplementary Fig. 194 ^{1}H NMR (400 MHz, 20 ºC) spectrum of product 59 in $CDCl_3$.

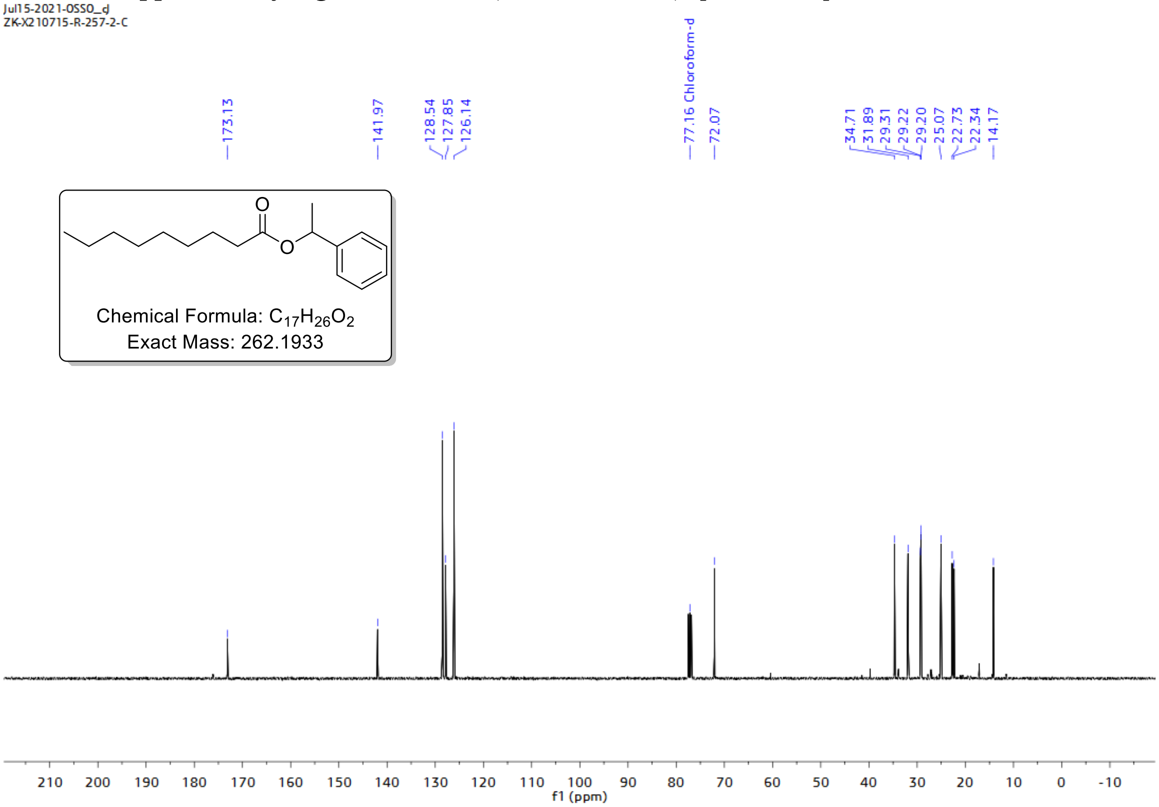

Supplementary Fig. 195 ^{13}C NMR (101 MHz, 20 ºC) spectrum of product 59 in $CDCl_3$.

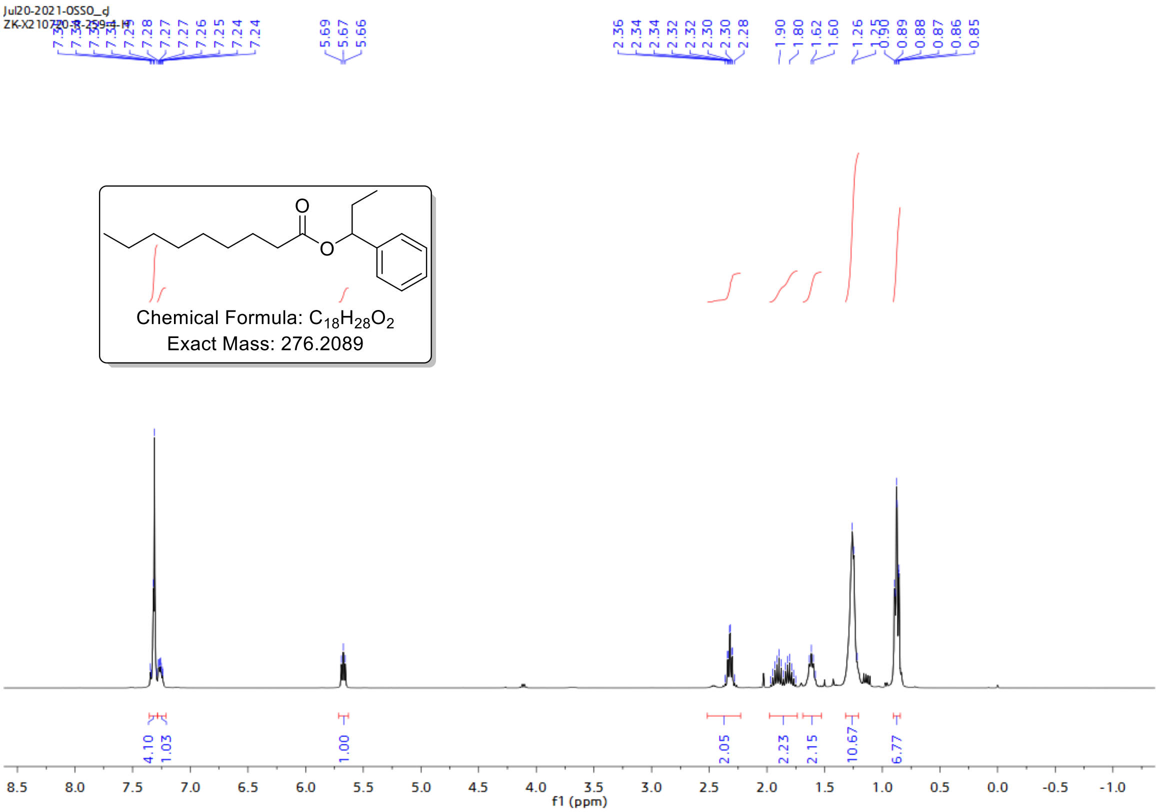

Supplementary Fig. 196 ^{1}H NMR (400 MHz, 20 ºC) spectrum of product 60 in $CDCl_3$.

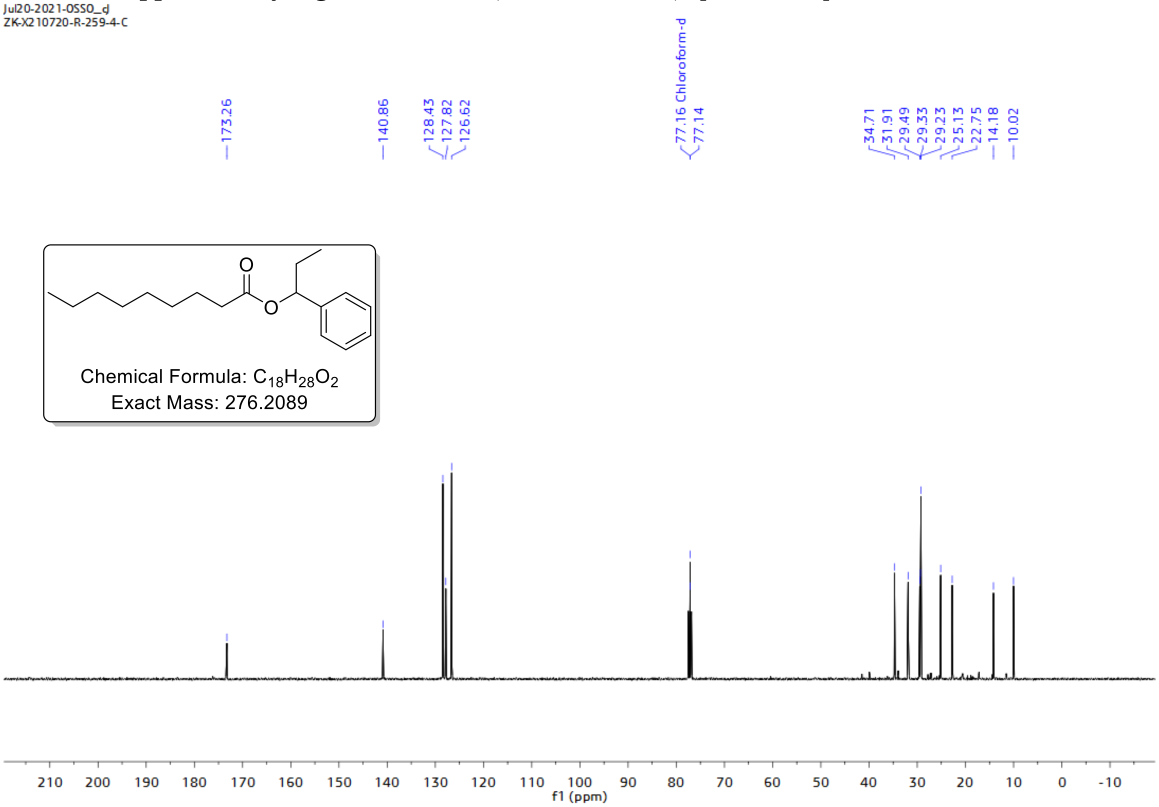

Supplementary Fig. 197 ^{13}C NMR (101 MHz, 20 ºC) spectrum of product 18 in $CDCl_3$.

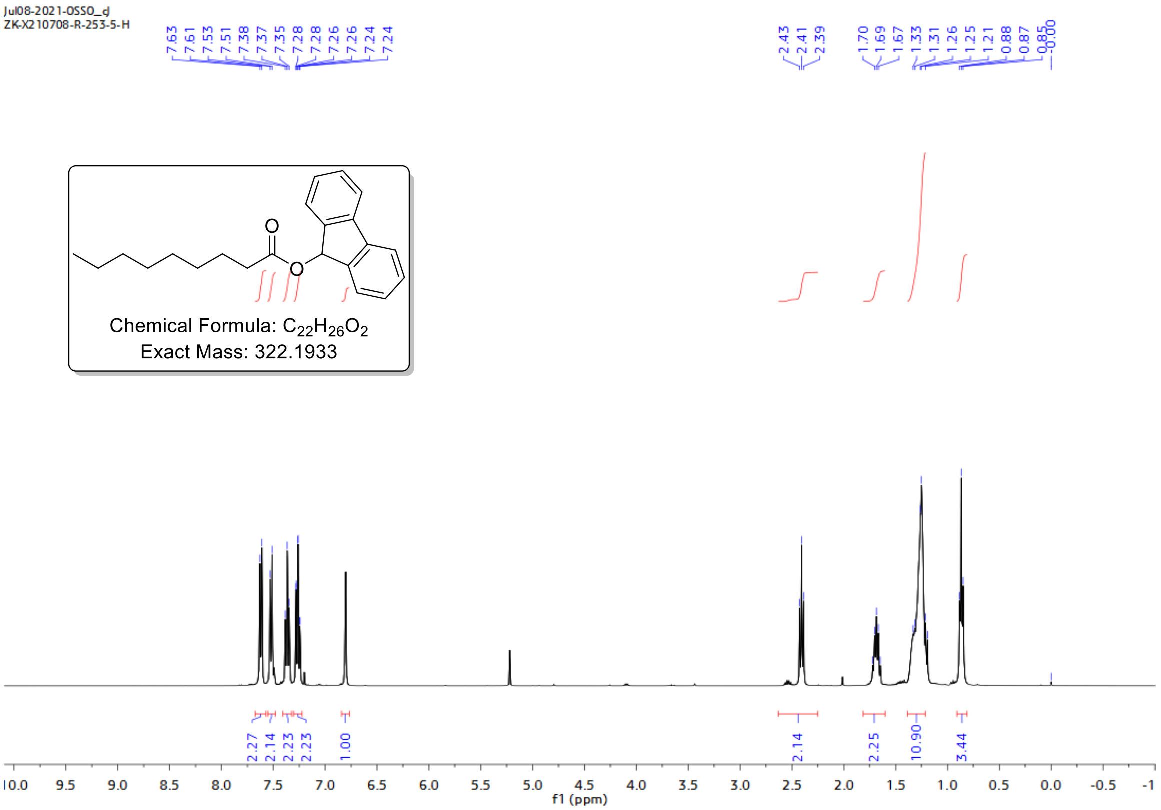

Supplementary Fig. 198 ^{1}H NMR (400 MHz, 20 ºC) spectrum of product 61 in $CDCl_3$.

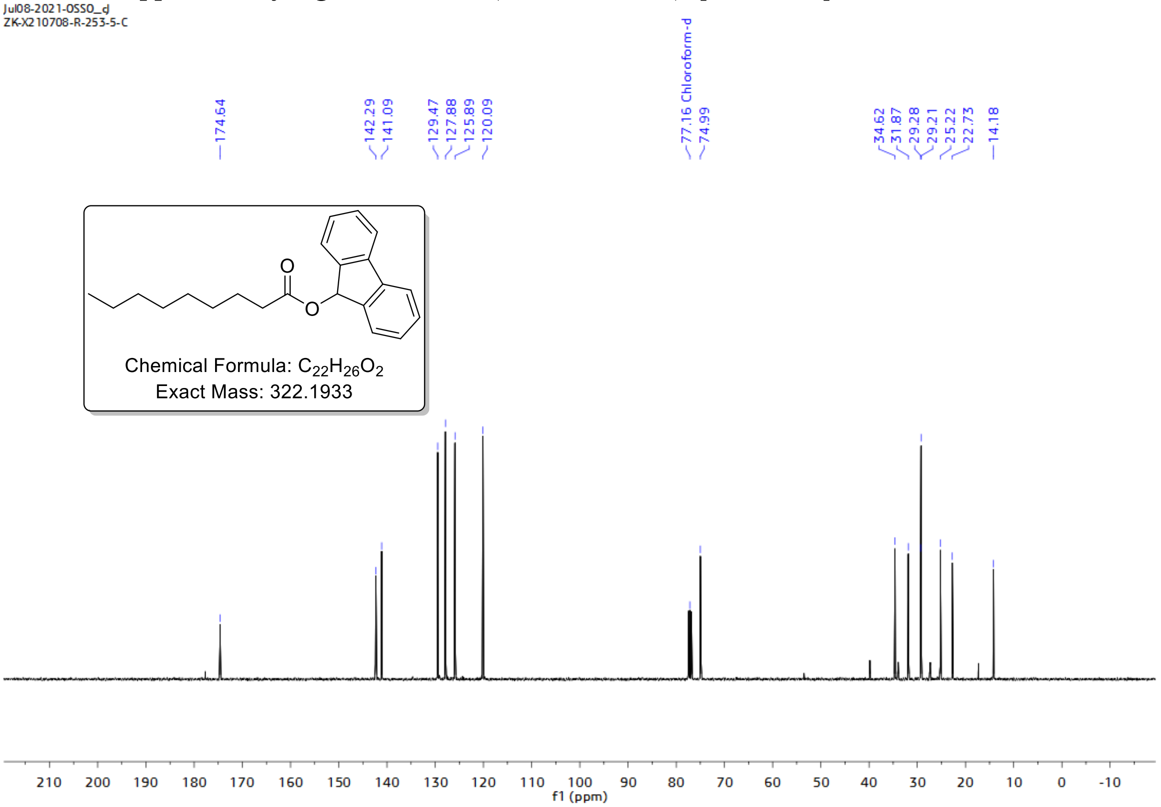

Supplementary Fig. 199 ^{13}C NMR (101 MHz, 20 ºC) spectrum of product 61 in $CDCl_3$.

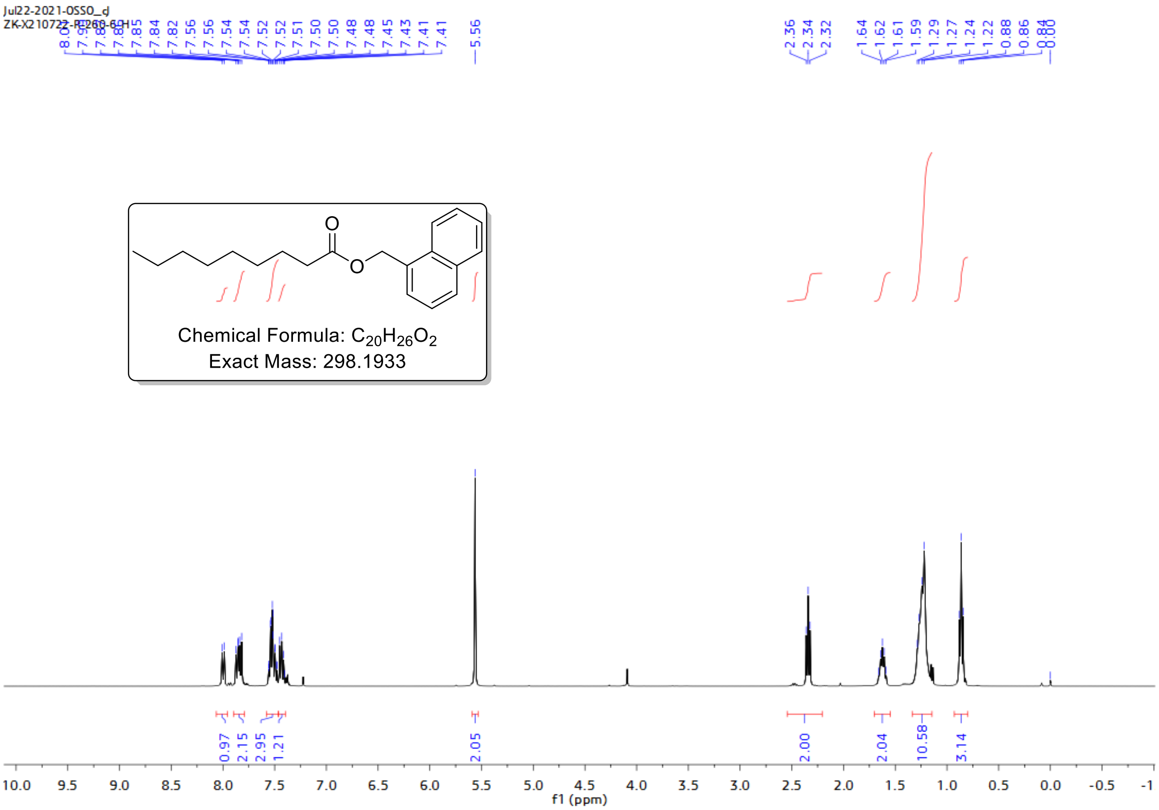

Supplementary Fig. 200 ^{1}H NMR (400 MHz, 20 ºC) spectrum of product 62 in $CDCl_3$.

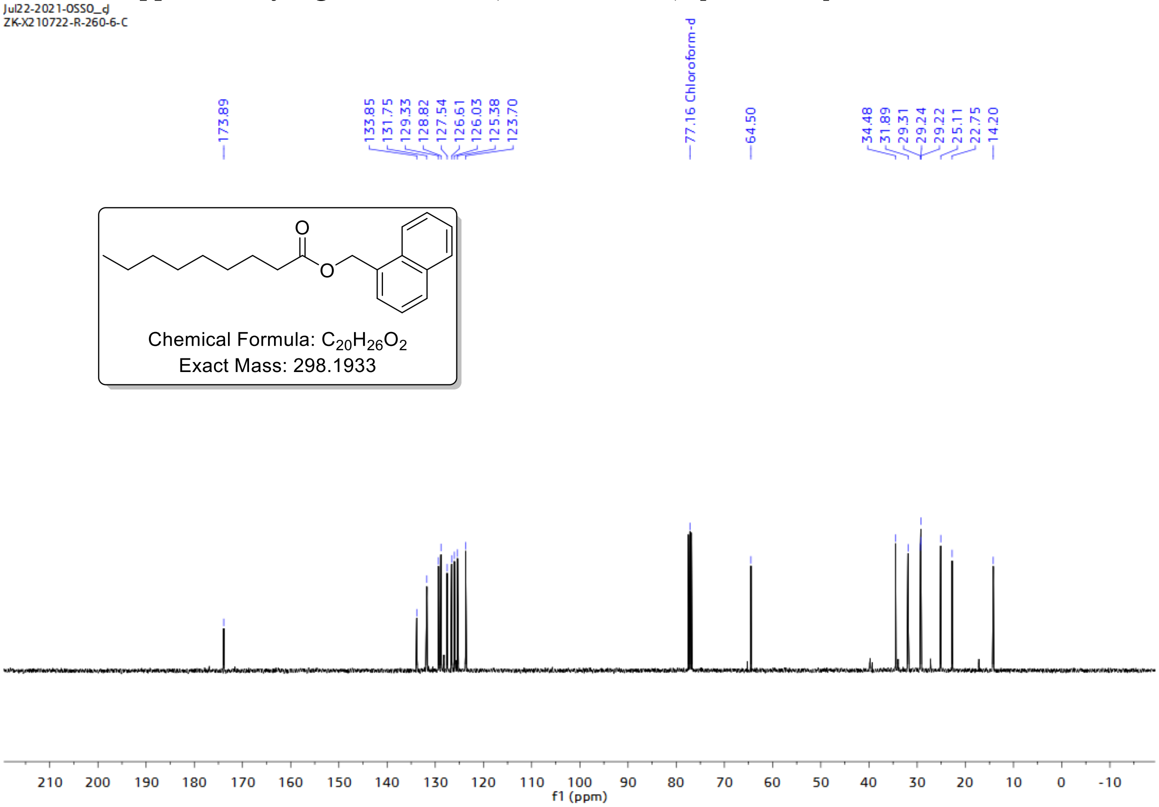

Supplementary Fig. 201 ^{13}C NMR (101 MHz, 20 ºC) spectrum of product 62 in $CDCl_3$.

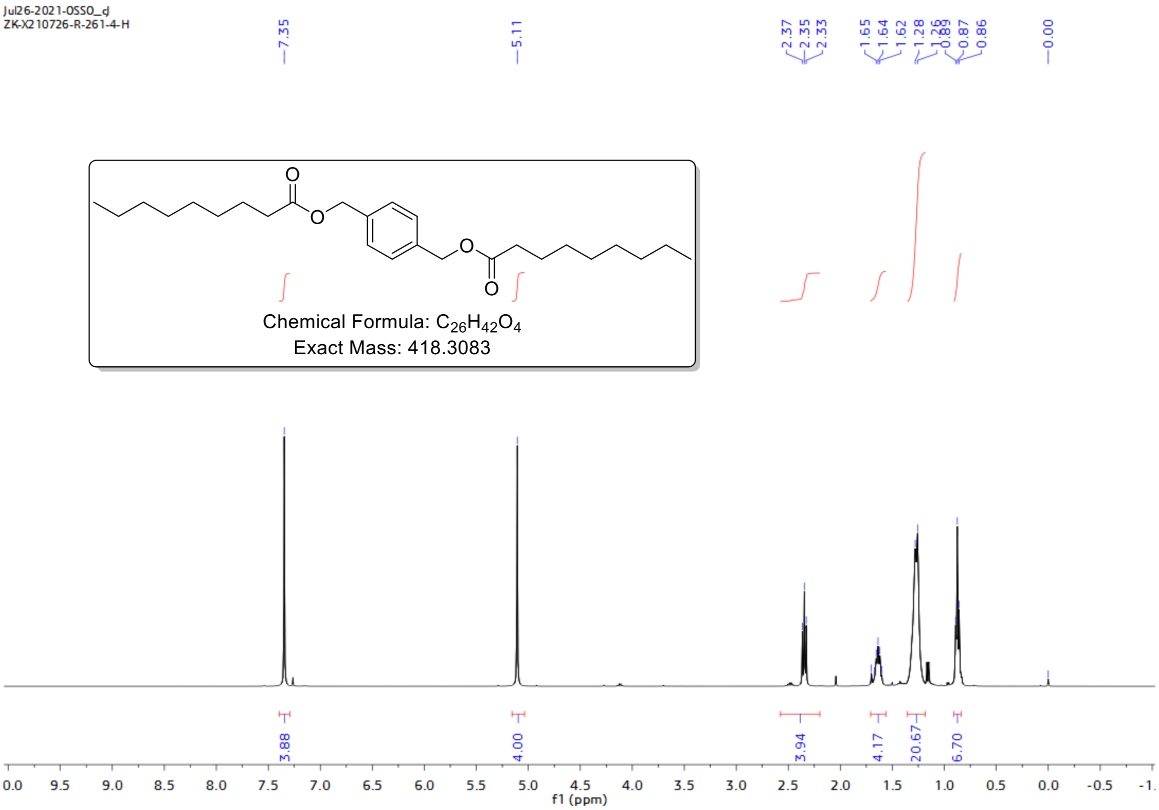

Supplementary Fig. 202 ^{1}H NMR (400 MHz, 20 ºC) spectrum of product 63 in $CDCl_3$.

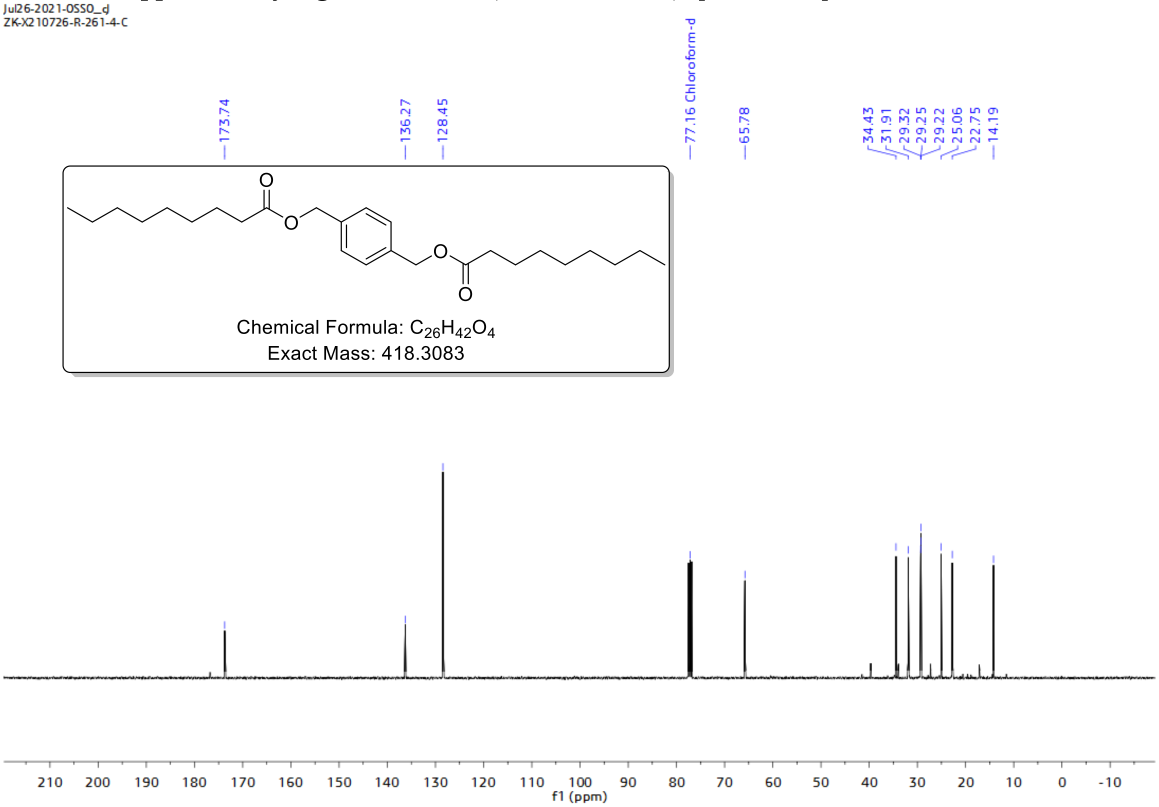

Supplementary Fig. 203 ^{13}C NMR (101 MHz, 20 ºC) spectrum of product 63 in $CDCl_3$.

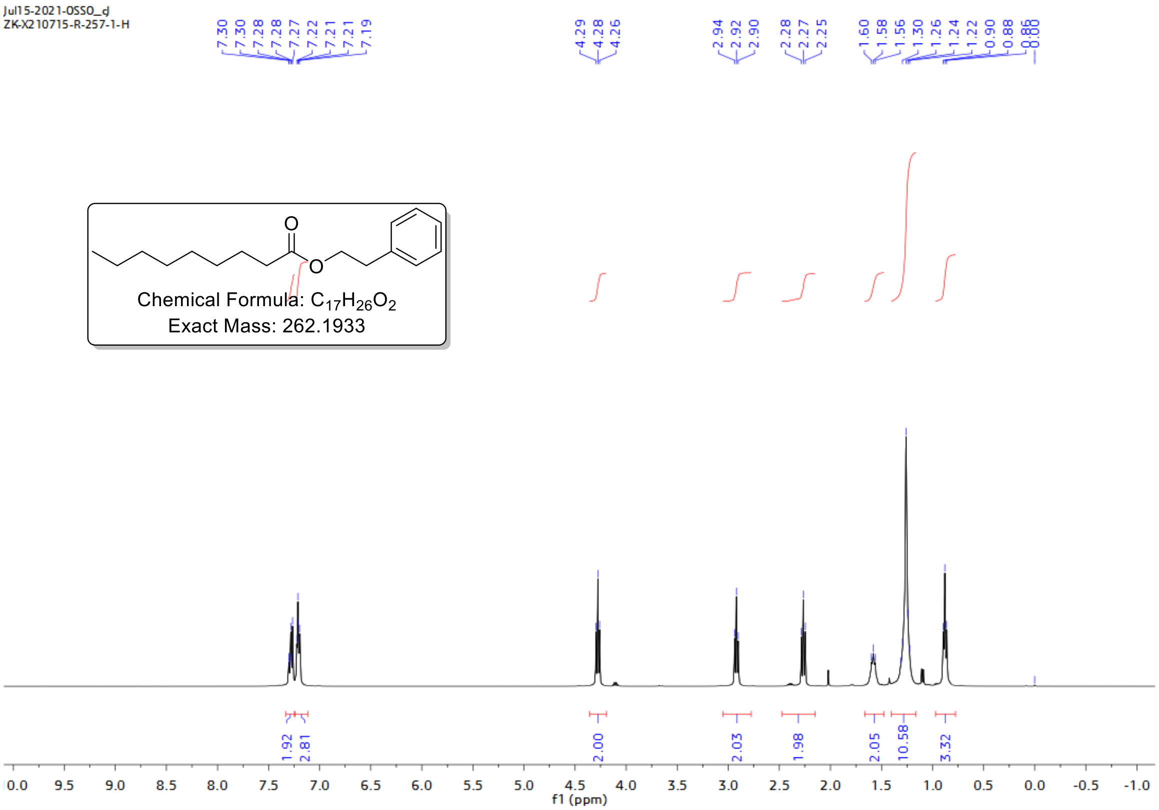

Supplementary Fig. 204 ^{1}H NMR (400 MHz, 20 ºC) spectrum of product 64 in $CDCl_3$.

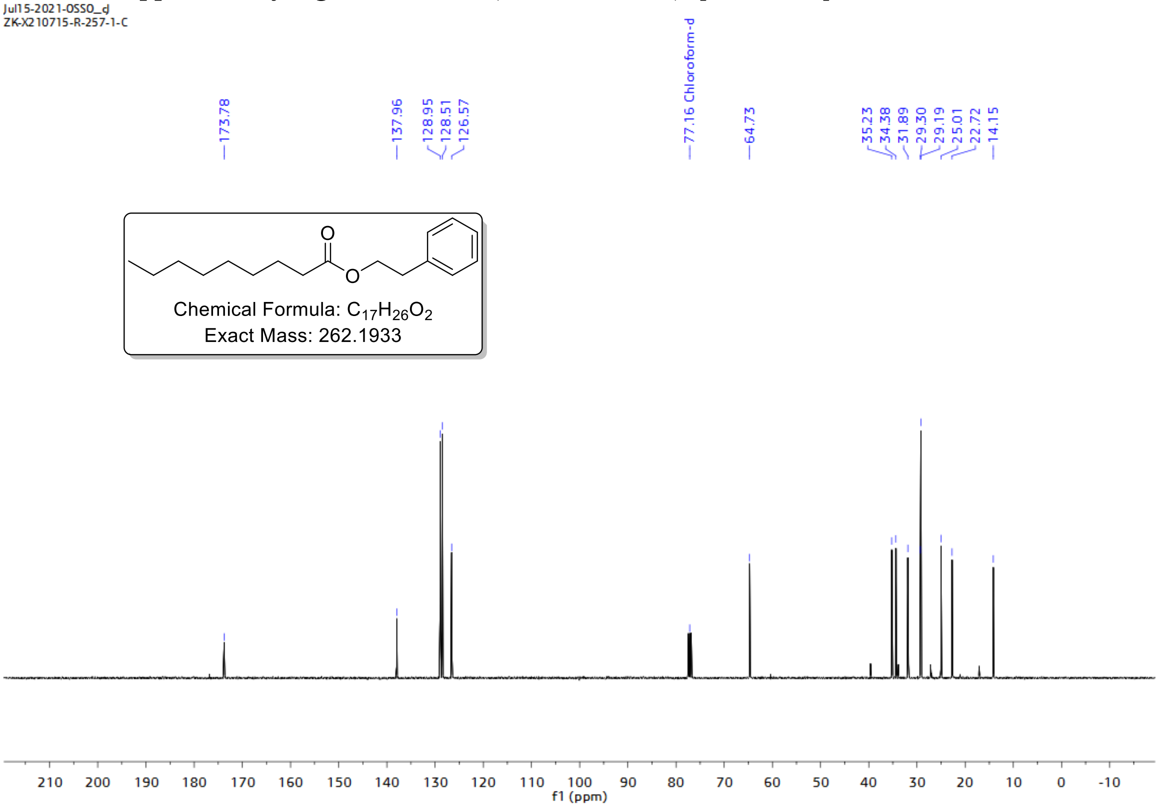

Supplementary Fig. 205 ^{13}C NMR (101 MHz, 20 ºC) spectrum of product 64 in $CDCl_3$.

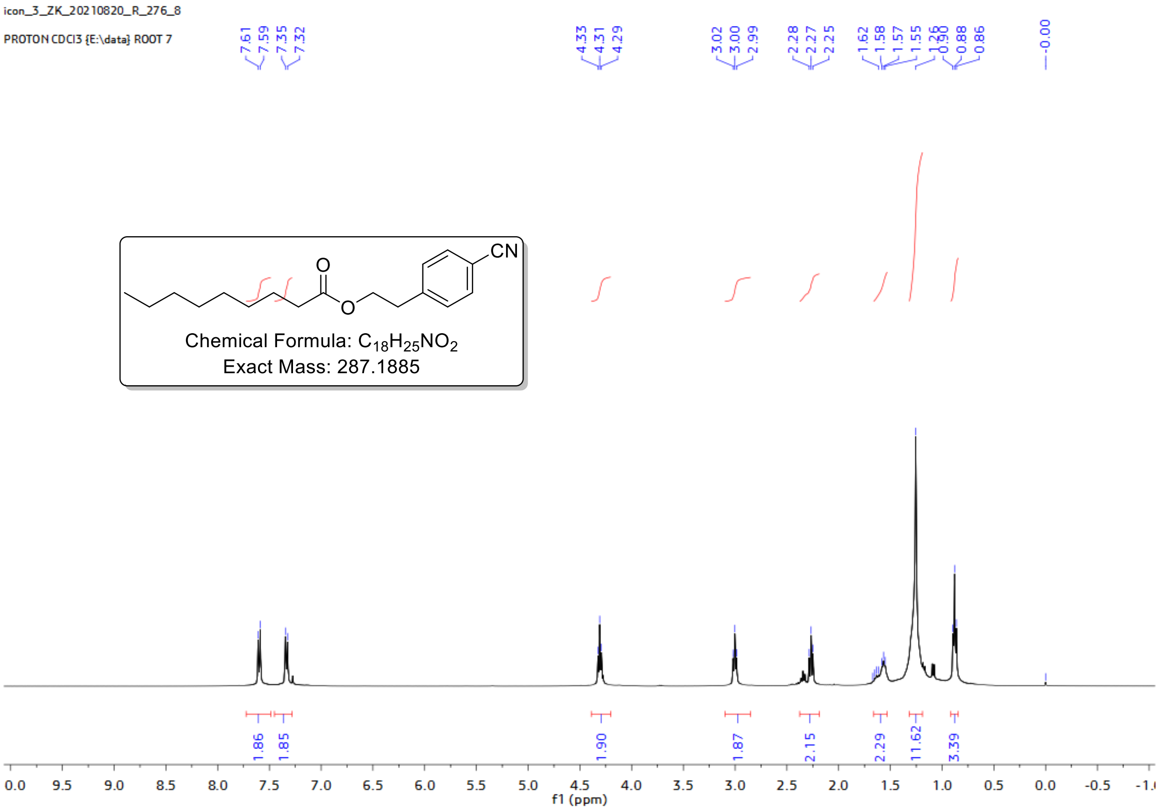

Supplementary Fig. 206 ^{1}H NMR (400 MHz, 20 ºC) spectrum of product 65 in $CDCl_3$.

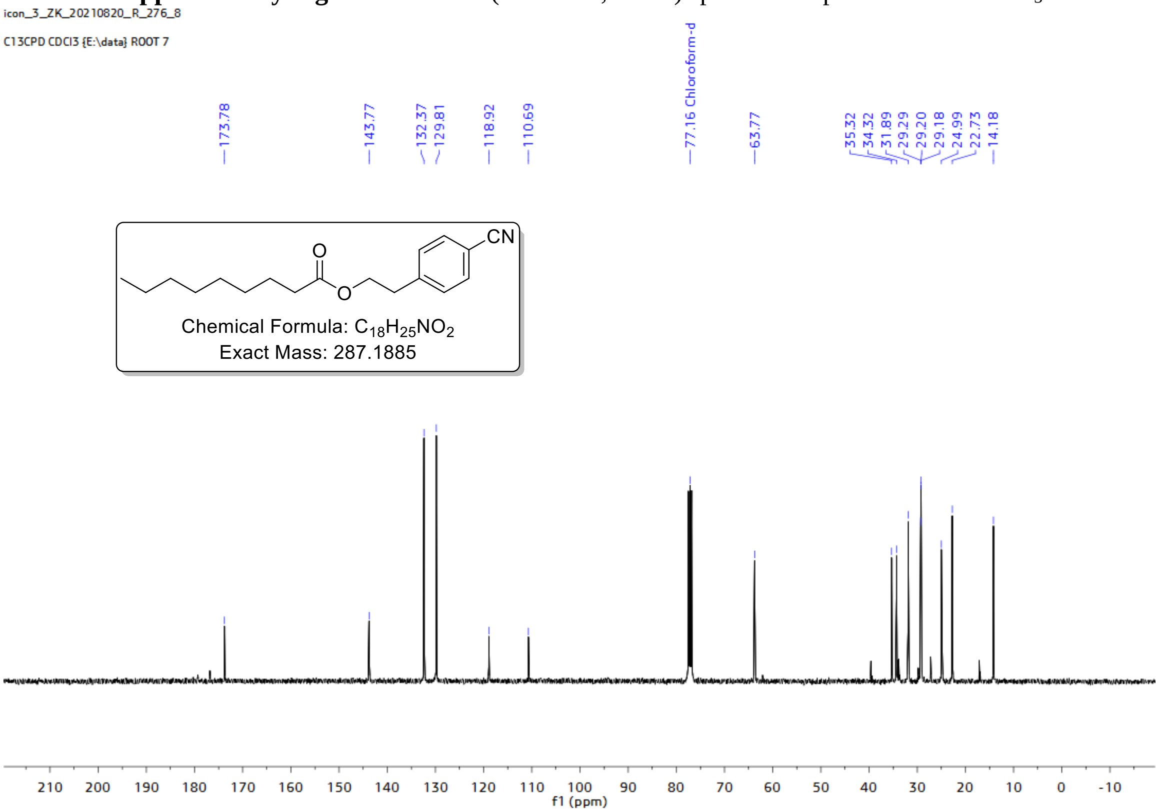

Supplementary Fig. 207 ^{13}C NMR (101 MHz, 20 ºC) spectrum of product 65 in $CDCl_3$.

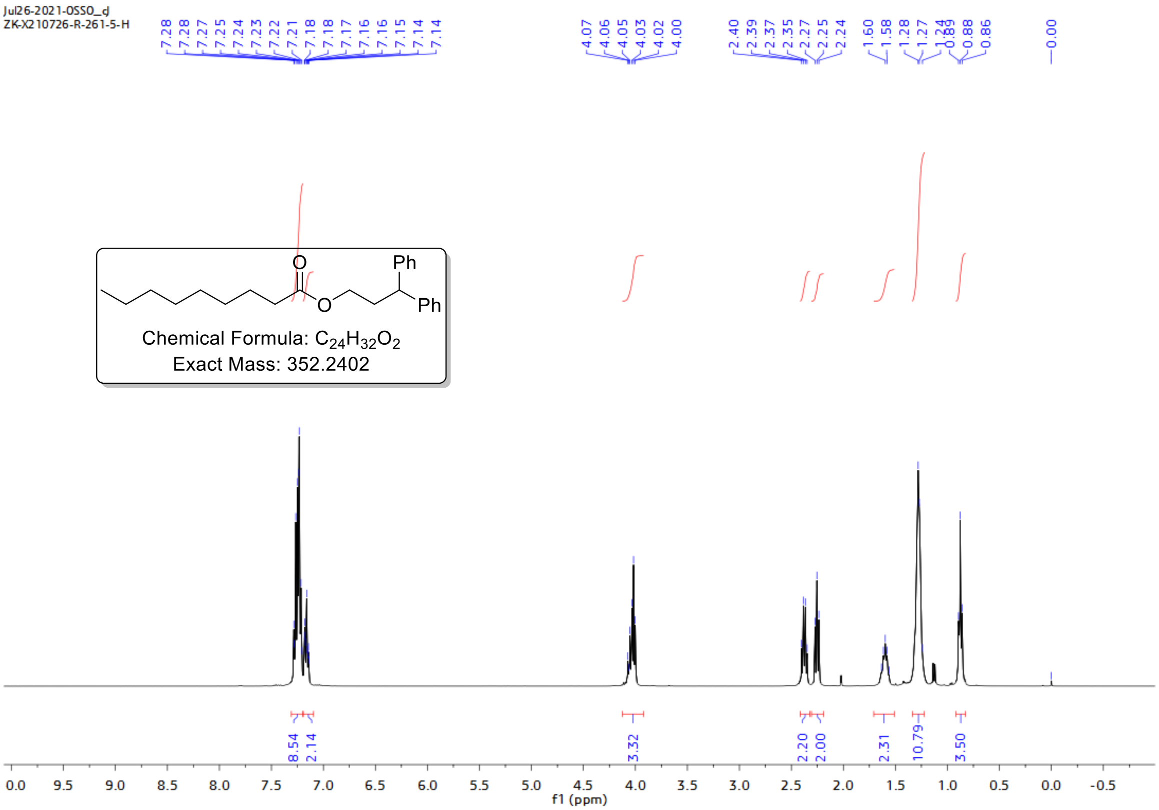

Supplementary Fig. 208 ^{1}H NMR (400 MHz, 20 ºC) spectrum of product 66 in $CDCl_3$.

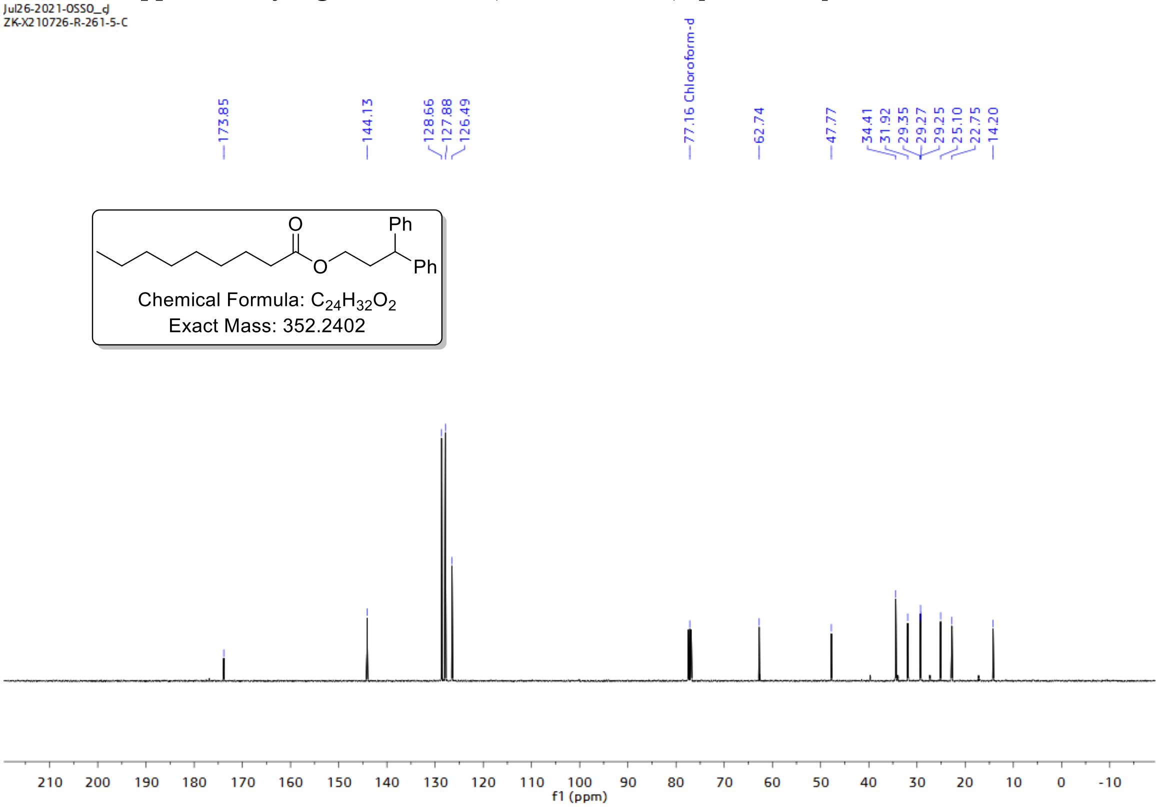

Supplementary Fig. 209 ^{13}C NMR (101 MHz, 20 ºC) spectrum of product 66 in $CDCl_3$.

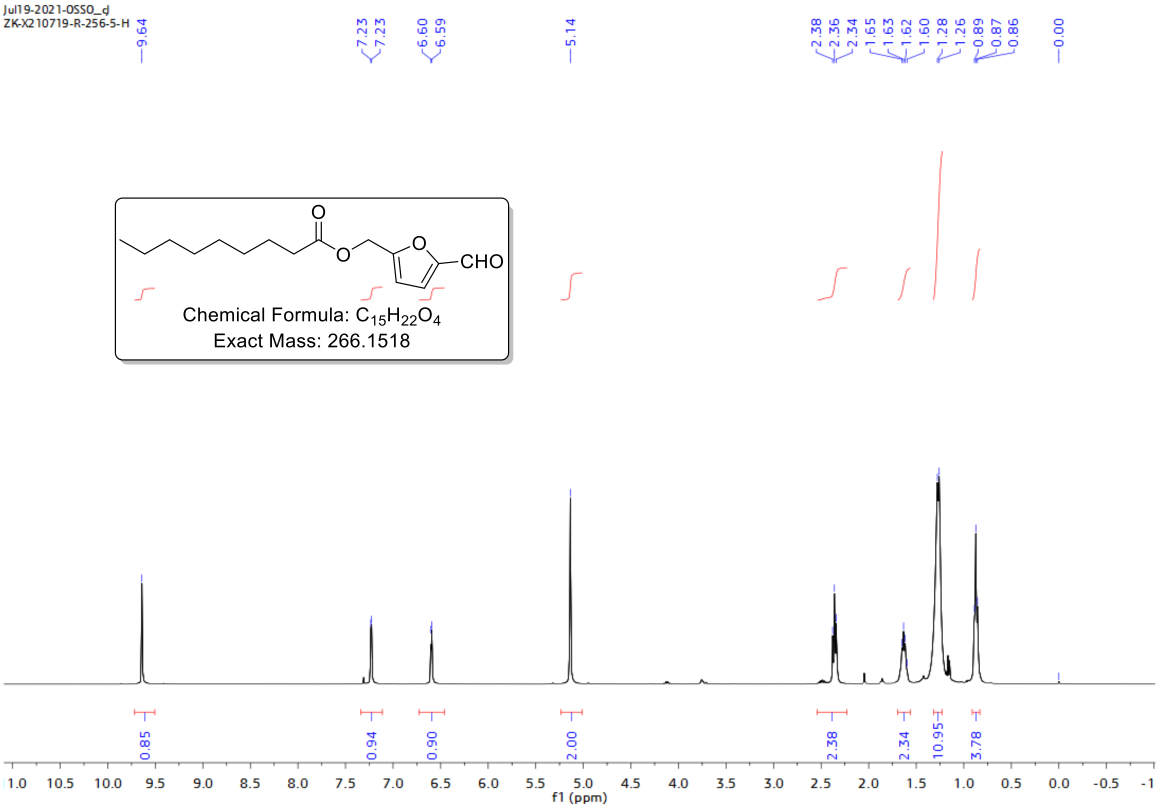

Supplementary Fig. 210 ^{1}H NMR (400 MHz, 20 ºC) spectrum of product 67 in $CDCl_3$.

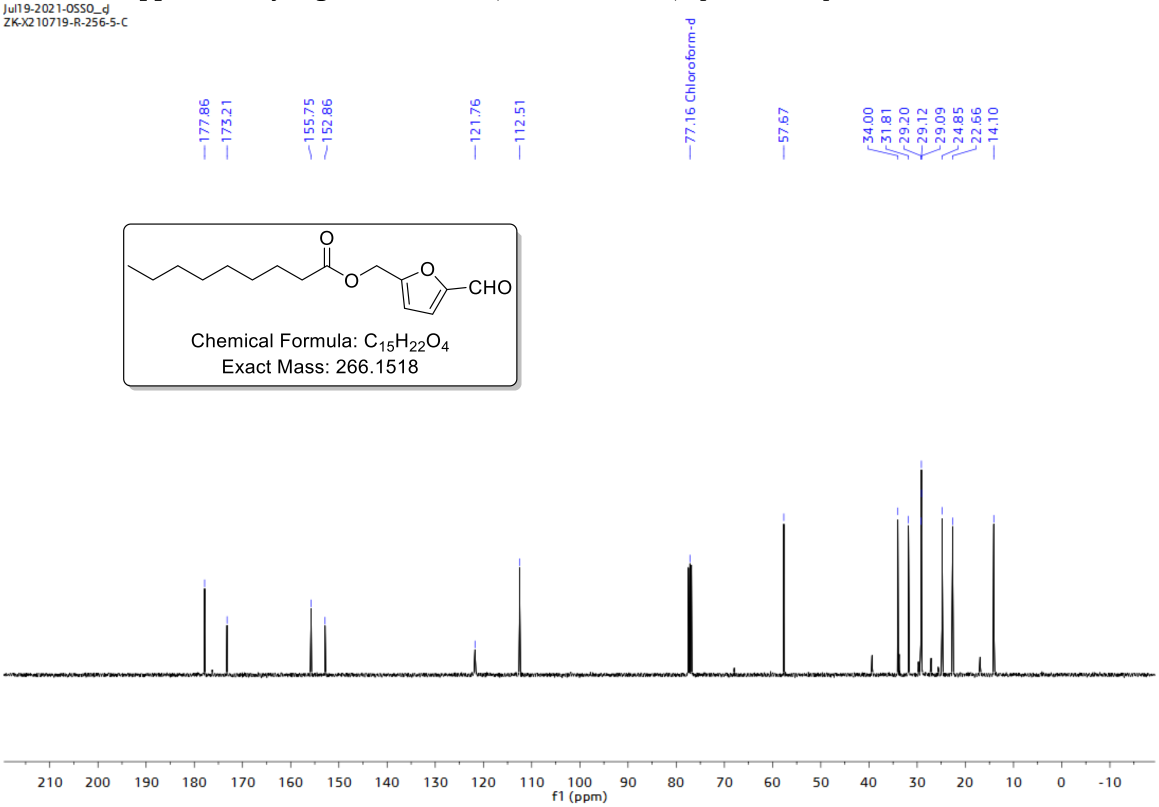

Supplementary Fig. 211 ^{13}C NMR (101 MHz, 20 ºC) spectrum of product 67 in $CDCl_3$.

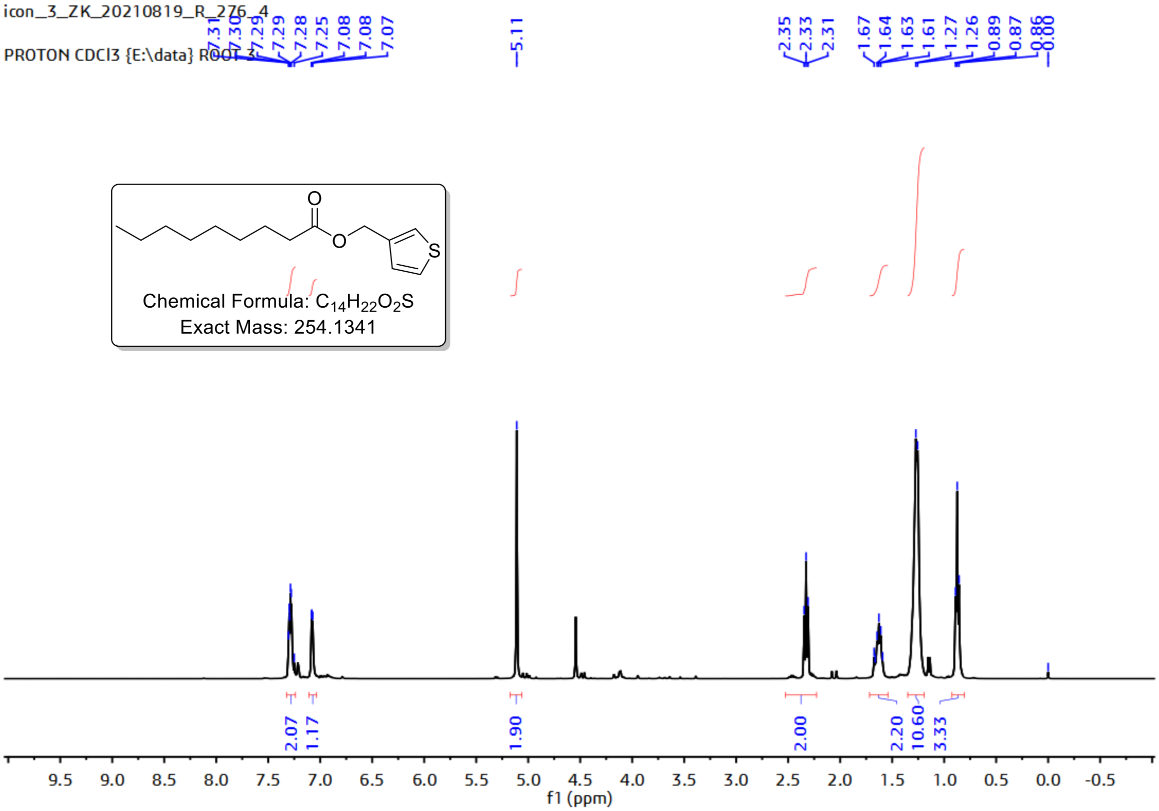

Supplementary Fig. 212 ^{1}H NMR (400 MHz, 20 ºC) spectrum of product 68 in $CDCl_3$.

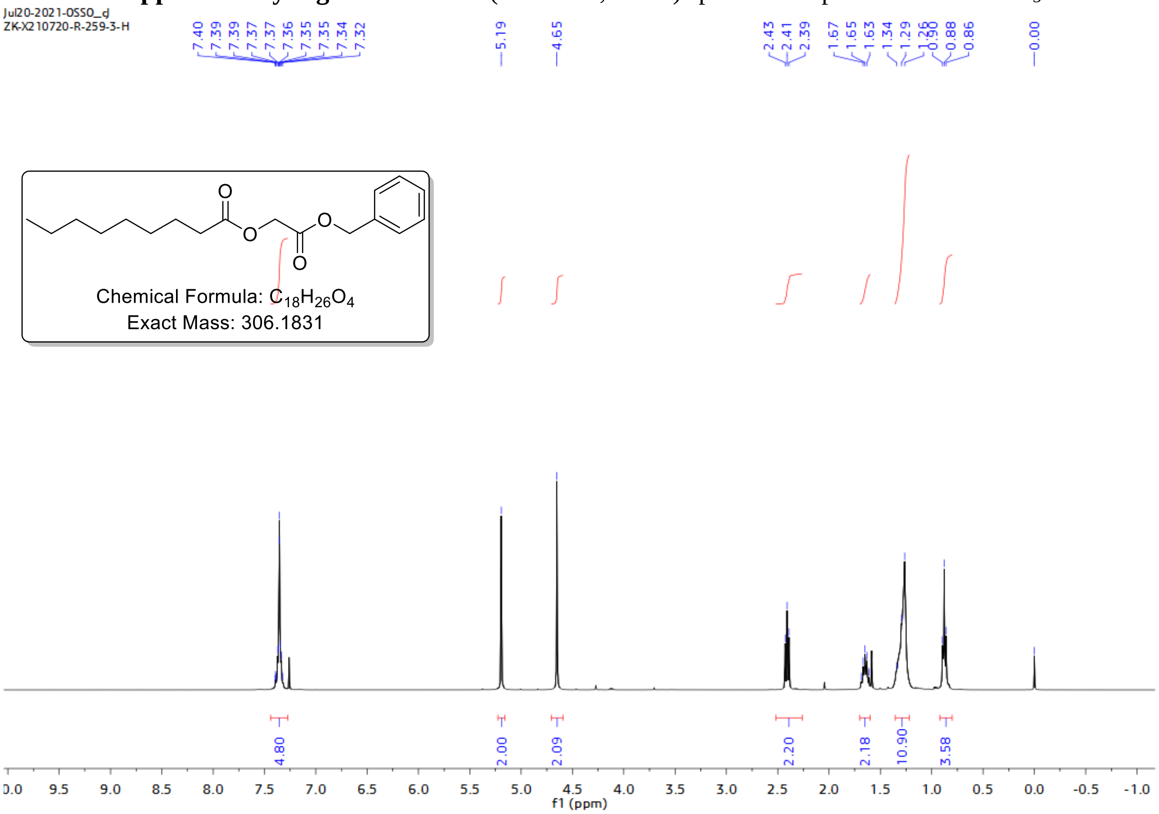

Supplementary Fig. 213 ^{1}H NMR (400 MHz, 20 ºC) spectrum of product 69 in $CDCl_3$.

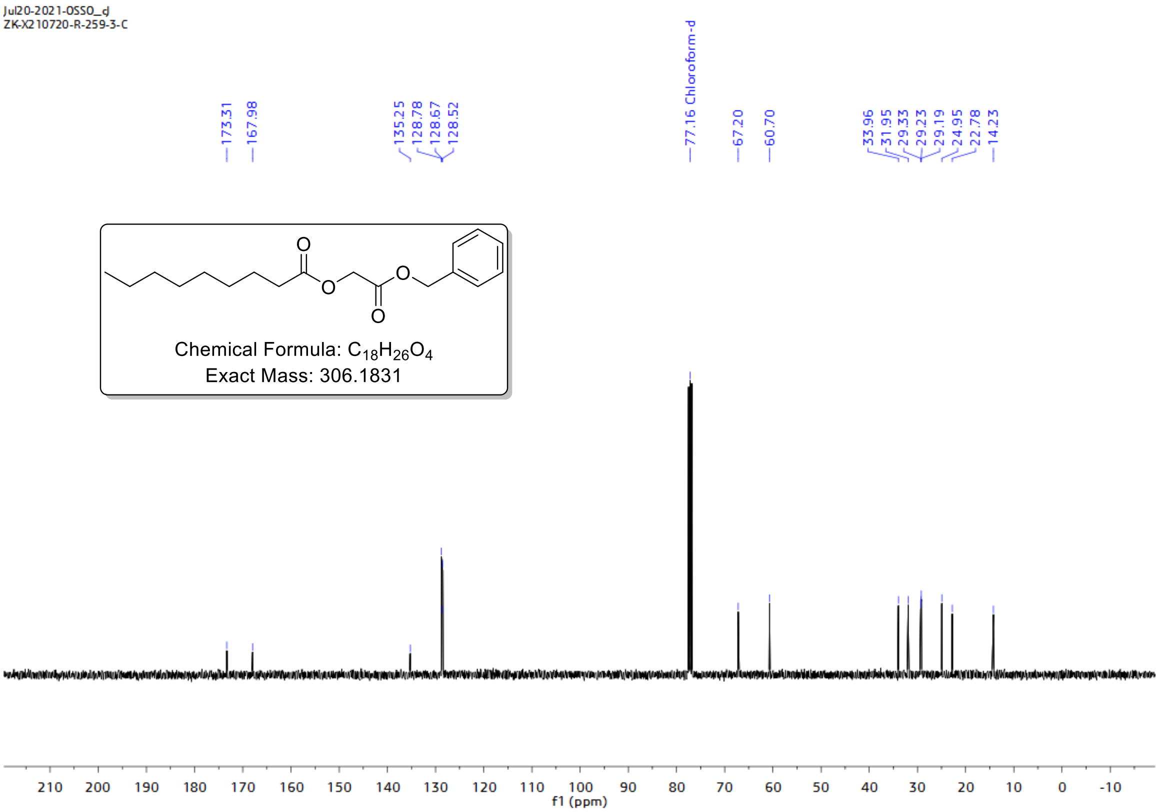

Supplementary Fig. 214 ^{13}C NMR (101 MHz, 20 ºC) spectrum of product 69 in $CDCl_3$.

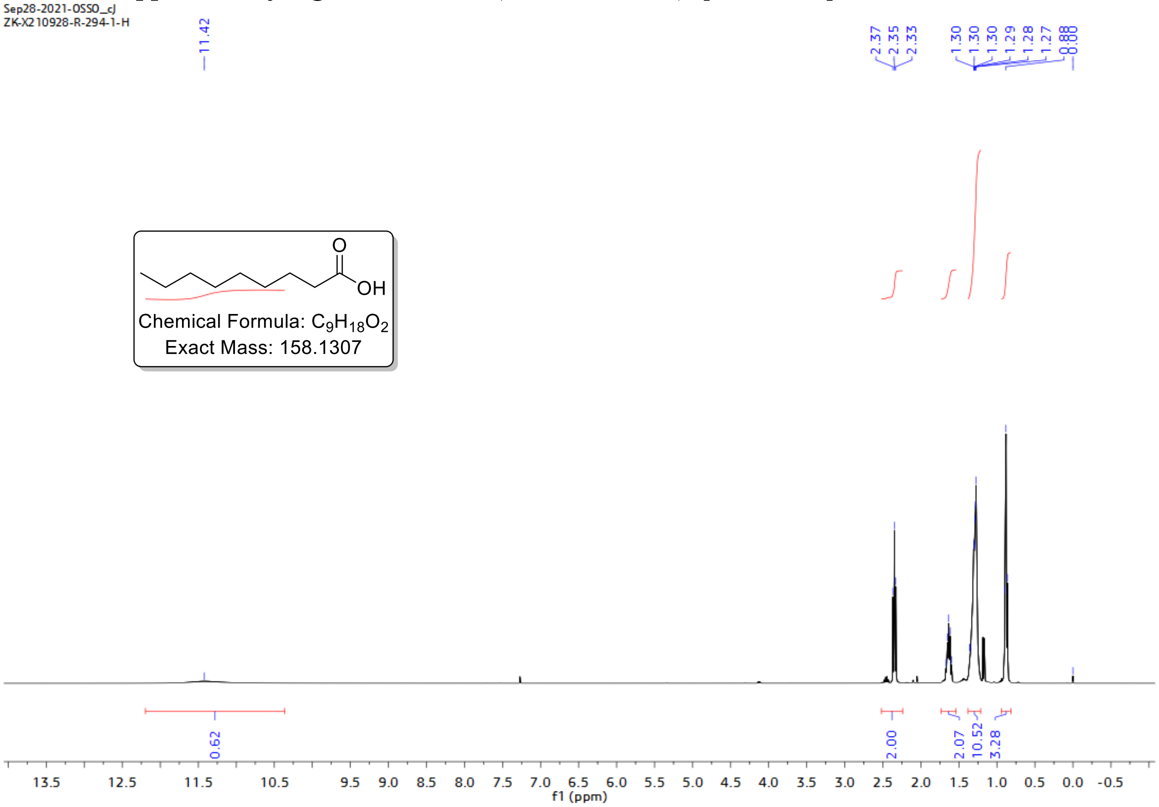

Supplementary Fig. 215 ^{1}H NMR (400 MHz, 20 ºC) spectrum of product 70 in $CDCl_3$.

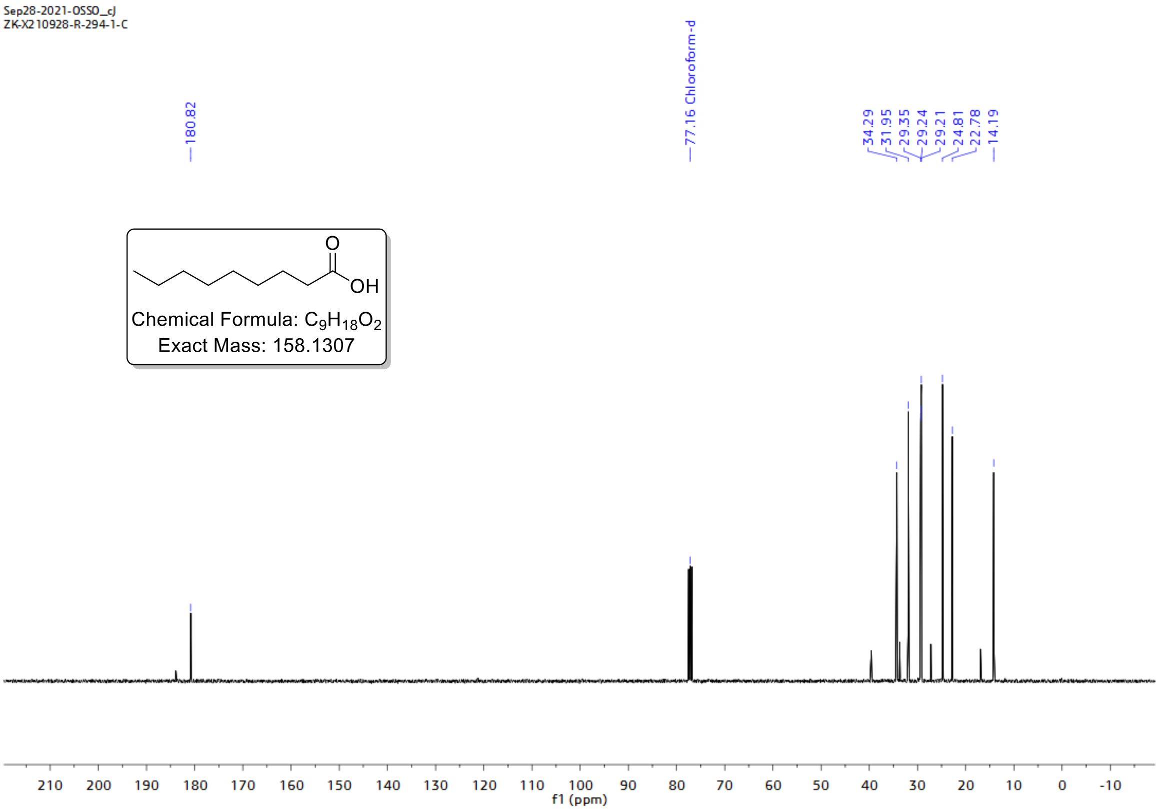

Supplementary Fig. 216 ^{13}C NMR (101 MHz, 20 ºC) spectrum of product 70 in $CDCl_3$.

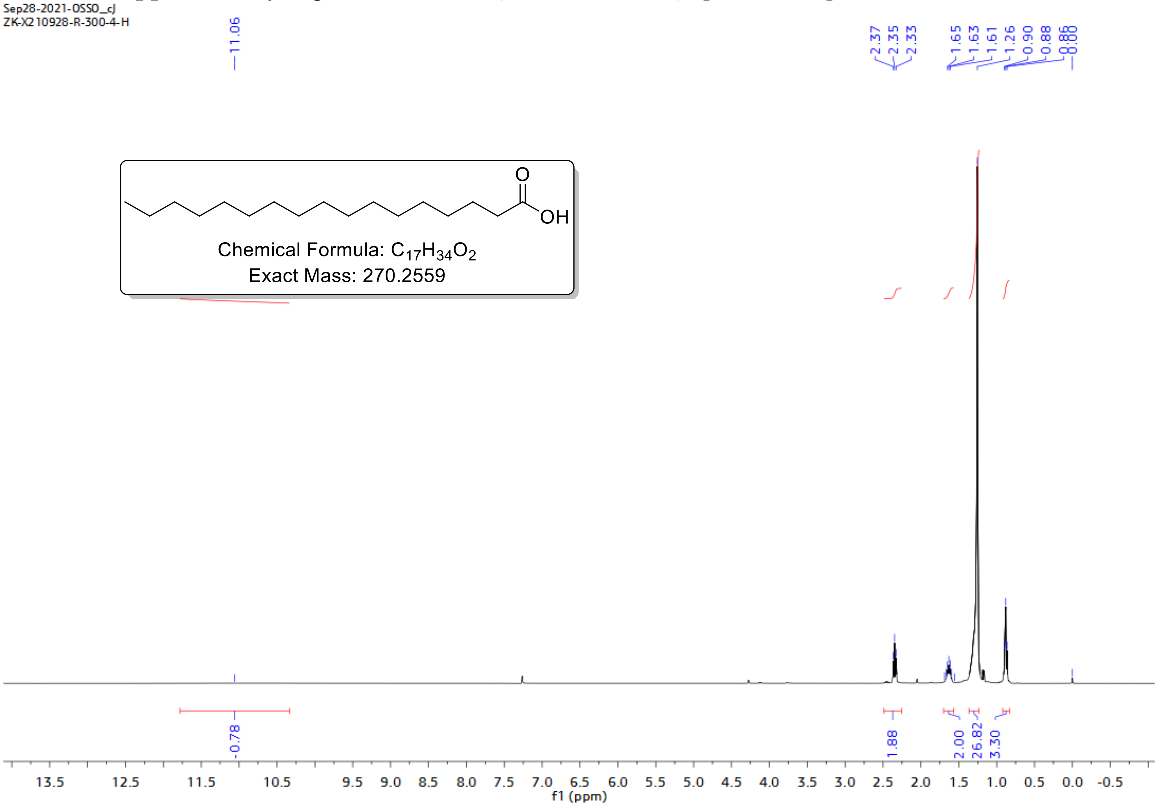

Supplementary Fig. 217 ^{1}H NMR (400 MHz, 20 ºC) spectrum of product 71 in $CDCl_3$.

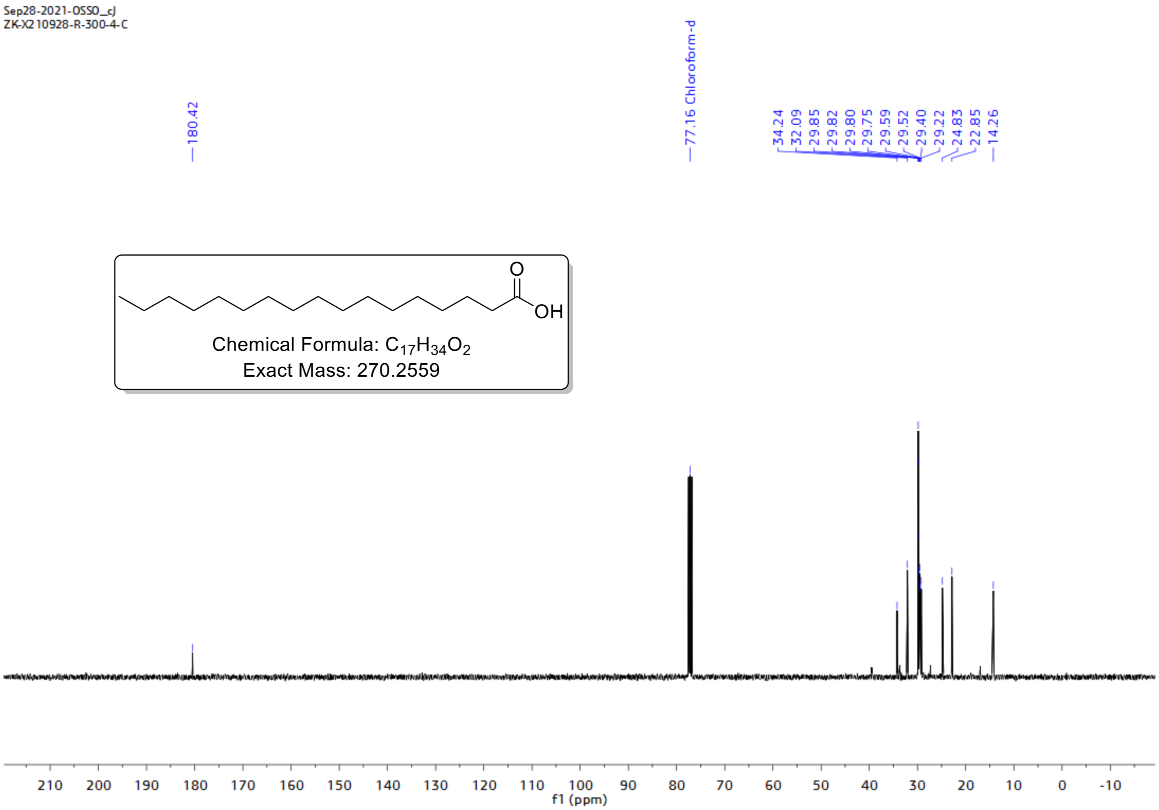

Supplementary Fig. 218 ^{13}C NMR (101 MHz, 20 ºC) spectrum of product 71 in $CDCl_3$.

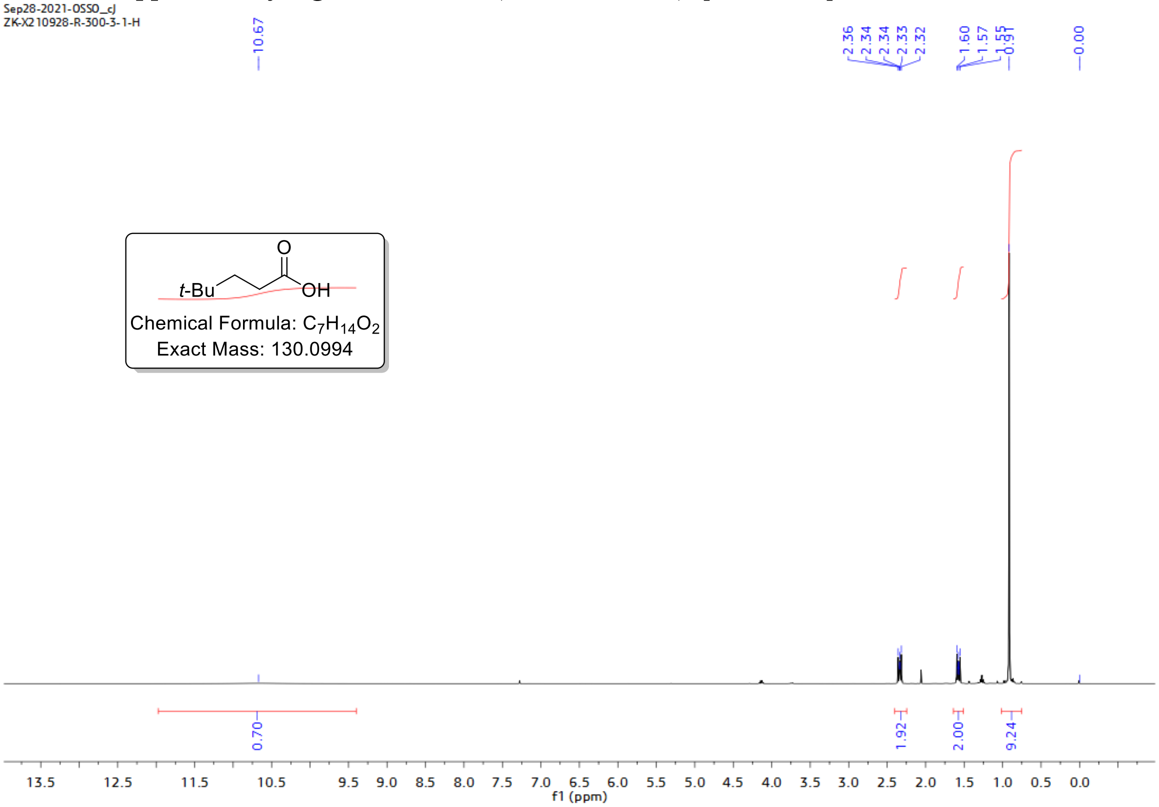

Supplementary Fig. 219 ^{1}H NMR (400 MHz, 20 ºC) spectrum of product 72 in $CDCl_3$.

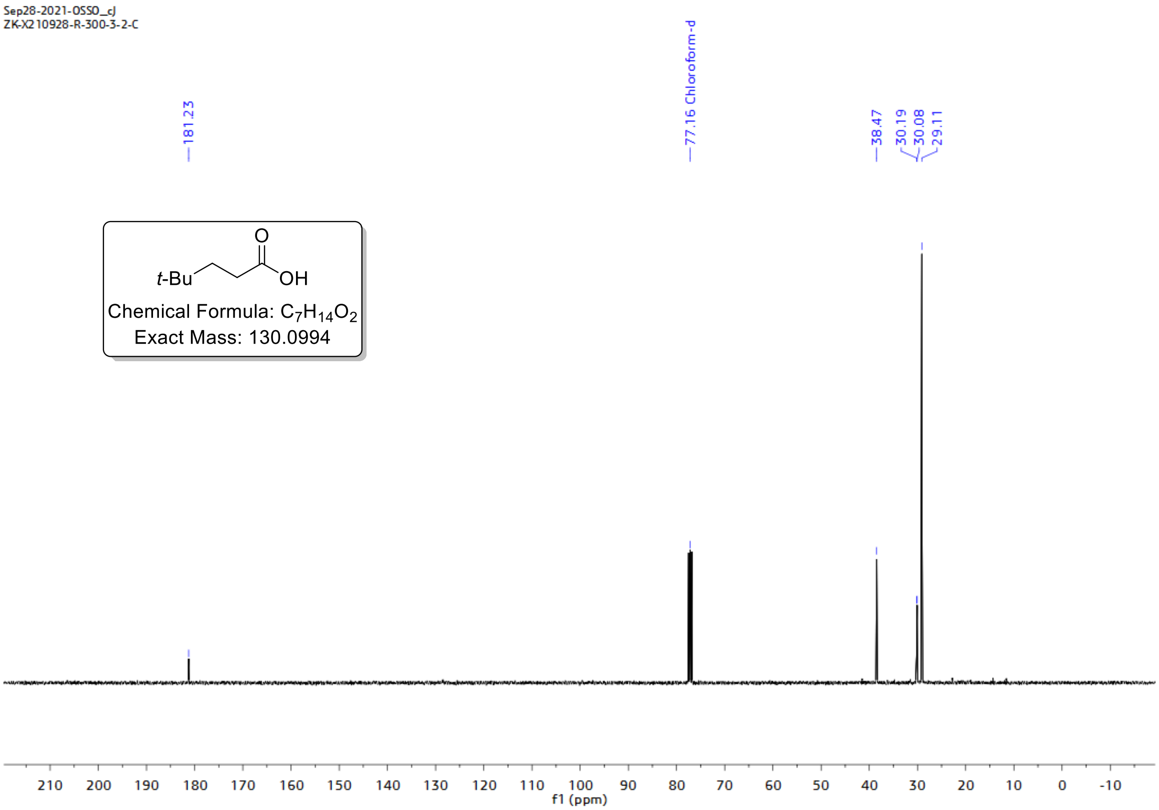

Supplementary Fig. 220 ^{13}C NMR (101 MHz, 20 ºC) spectrum of product 72 in $CDCl_3$.

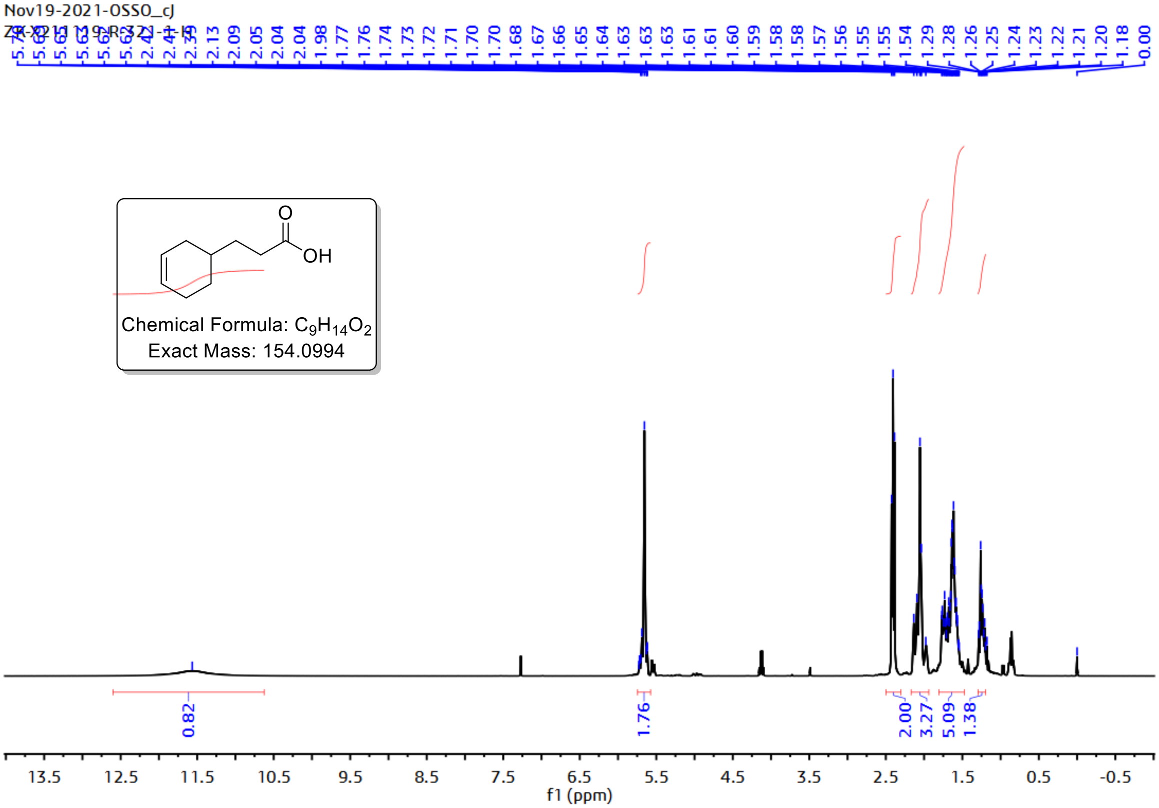

Supplementary Fig. 221 ^{1}H NMR (400 MHz, 20 ºC) spectrum of product 73 in $CDCl_3$.

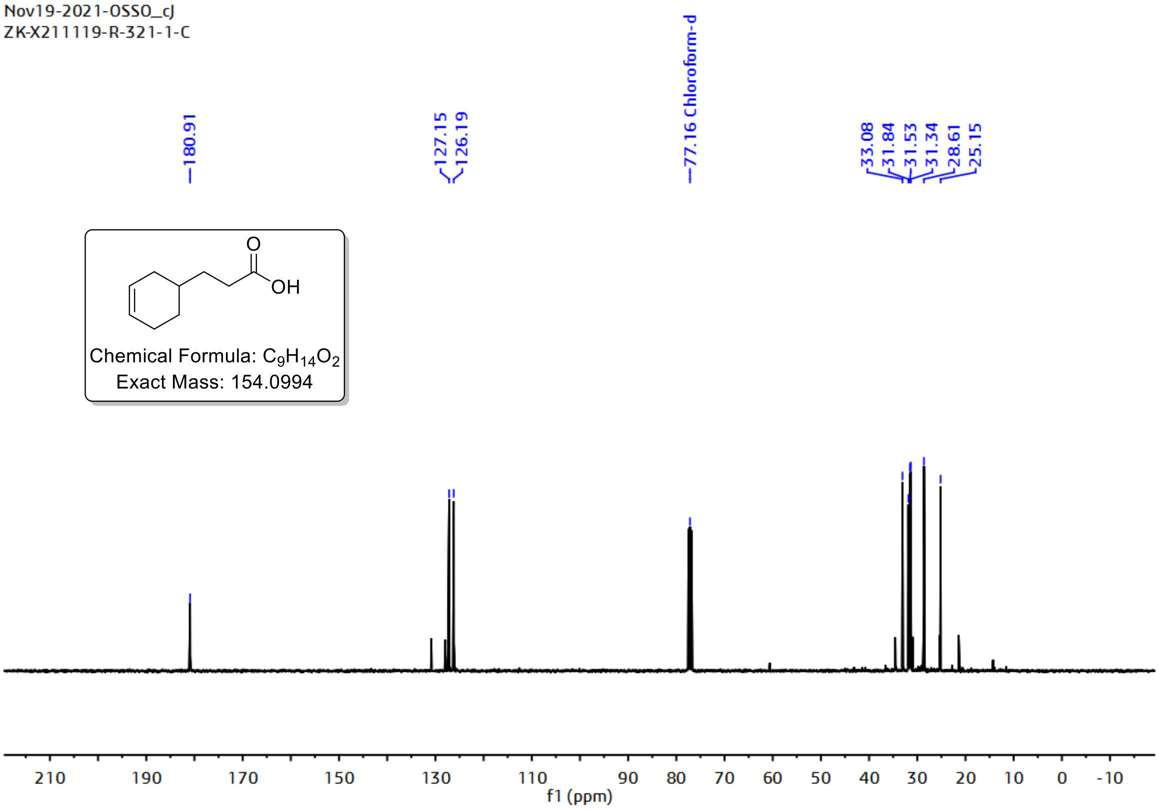

Supplementary Fig. 222 ^{13}C NMR (101 MHz, 20 ºC) spectrum of product 73 in $CDCl_3$.

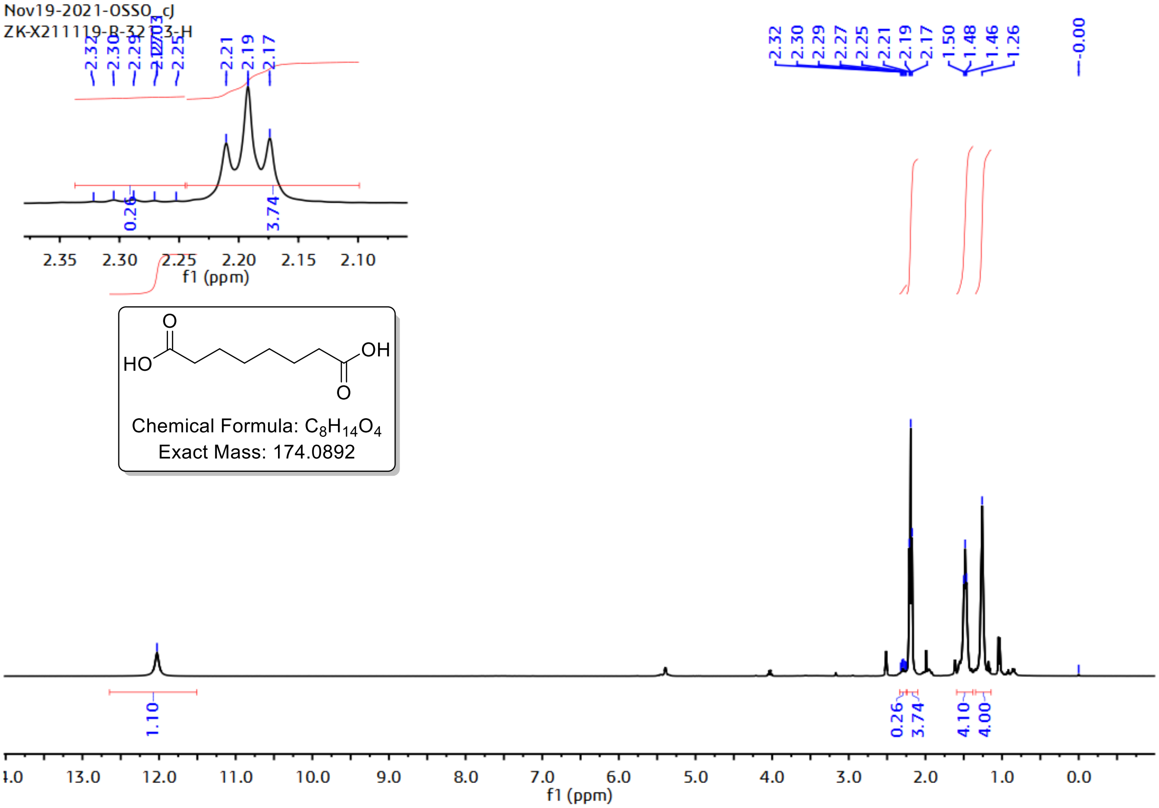

Supplementary Fig. 223 ^{1}H NMR (400 MHz, 20 ºC) spectrum of product 74 in DMSO-d6.

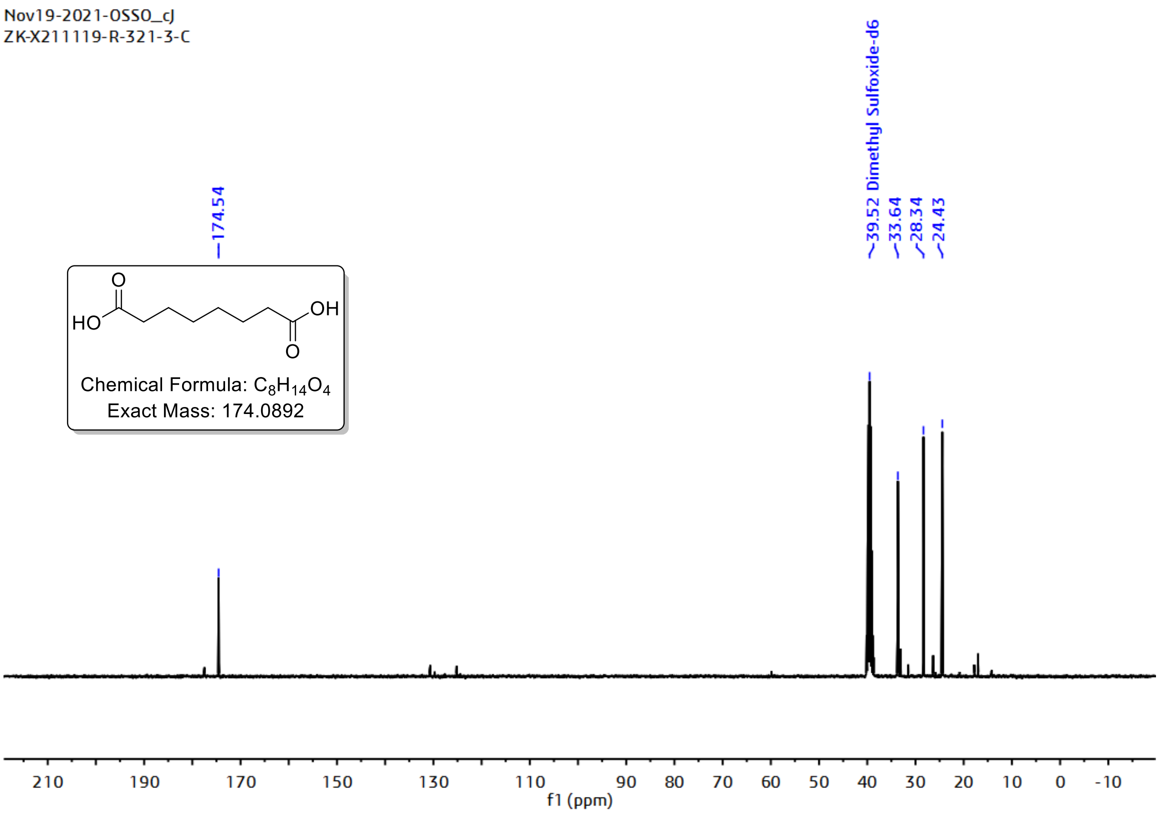

Supplementary Fig. 224 ^{13}C NMR (101 MHz, 20 °C) spectrum of product 74 in DMSO-d6.

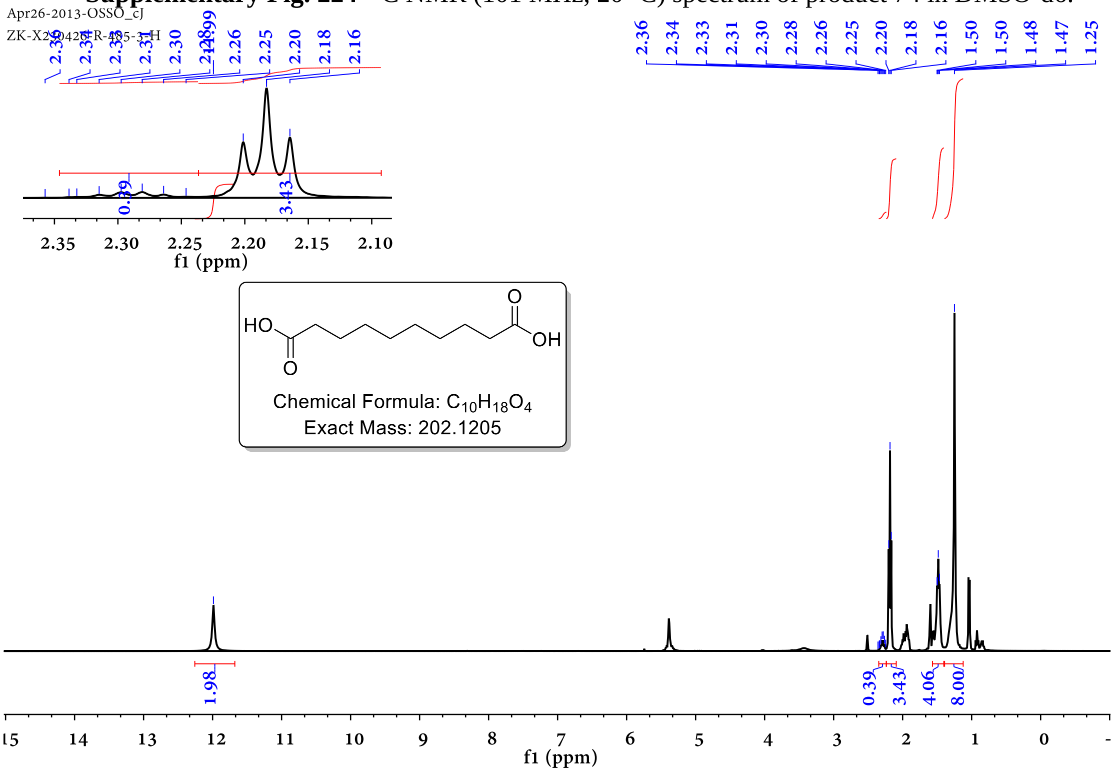

Supplementary Fig. 225 ^{1}H NMR (400 MHz, 20 °C) spectrum of product 75 in DMSO-d6.

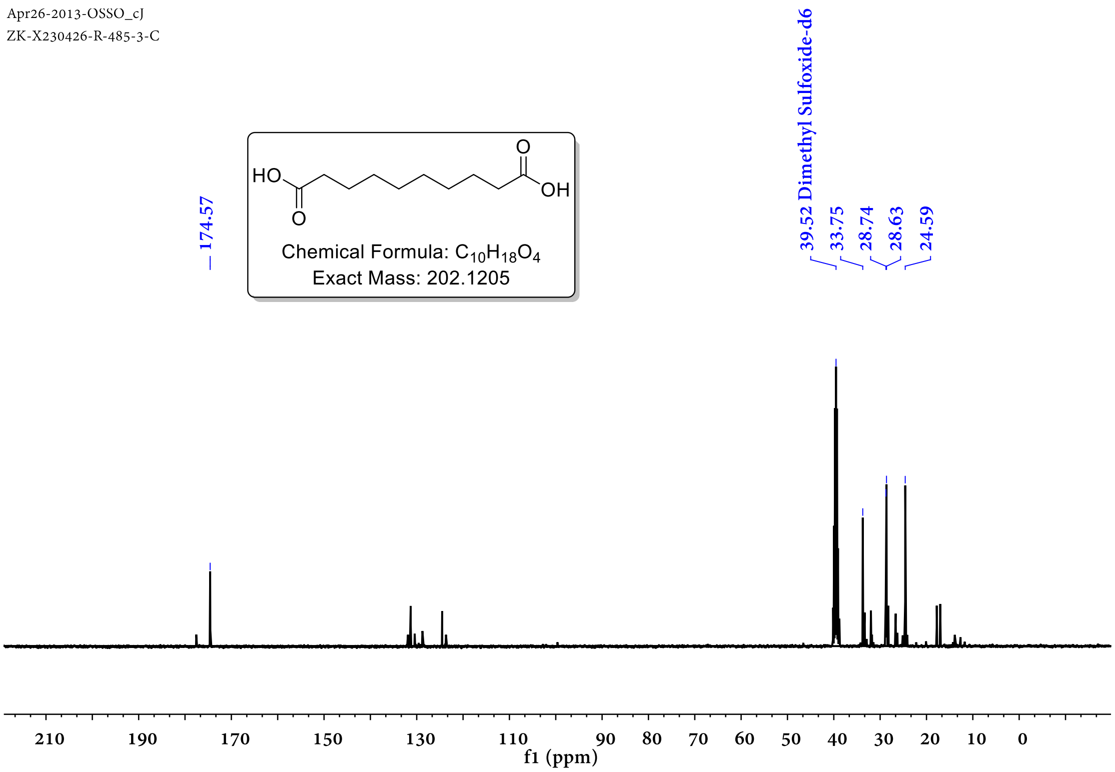

Supplementary Fig. 226 ^{13}C NMR (101 MHz, 20 °C) spectrum of product 75 in DMSO-d6.

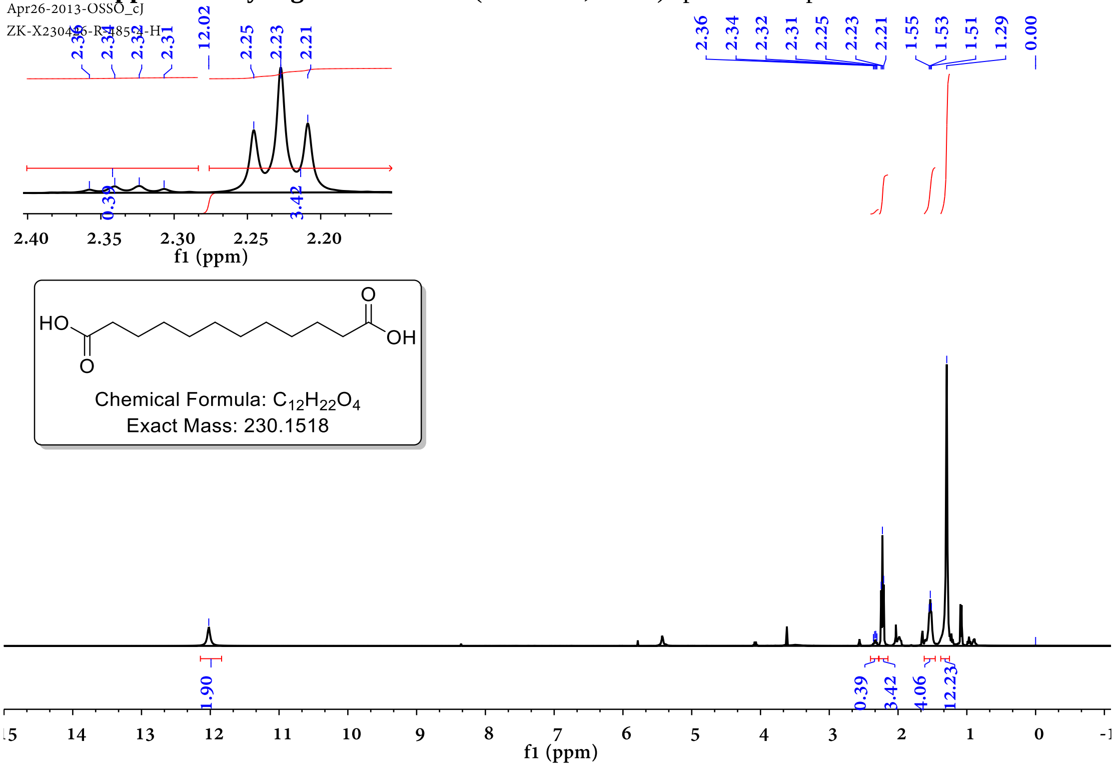

Supplementary Fig. 227 ^{1}H NMR (400 MHz, 20 °C) spectrum of product 76 in DMSO-d6.

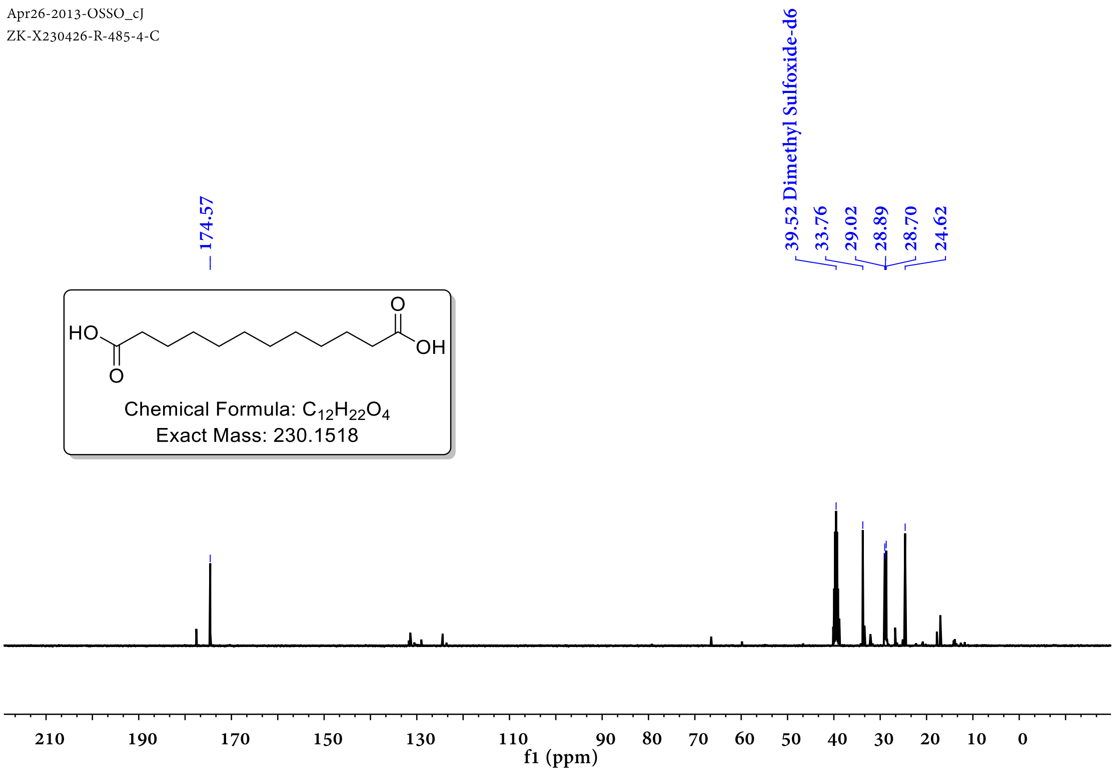

Supplementary Fig. 228 ^{13}C NMR (101 MHz, 20 ^{o}C) spectrum of product 76 in DMSO-d6.

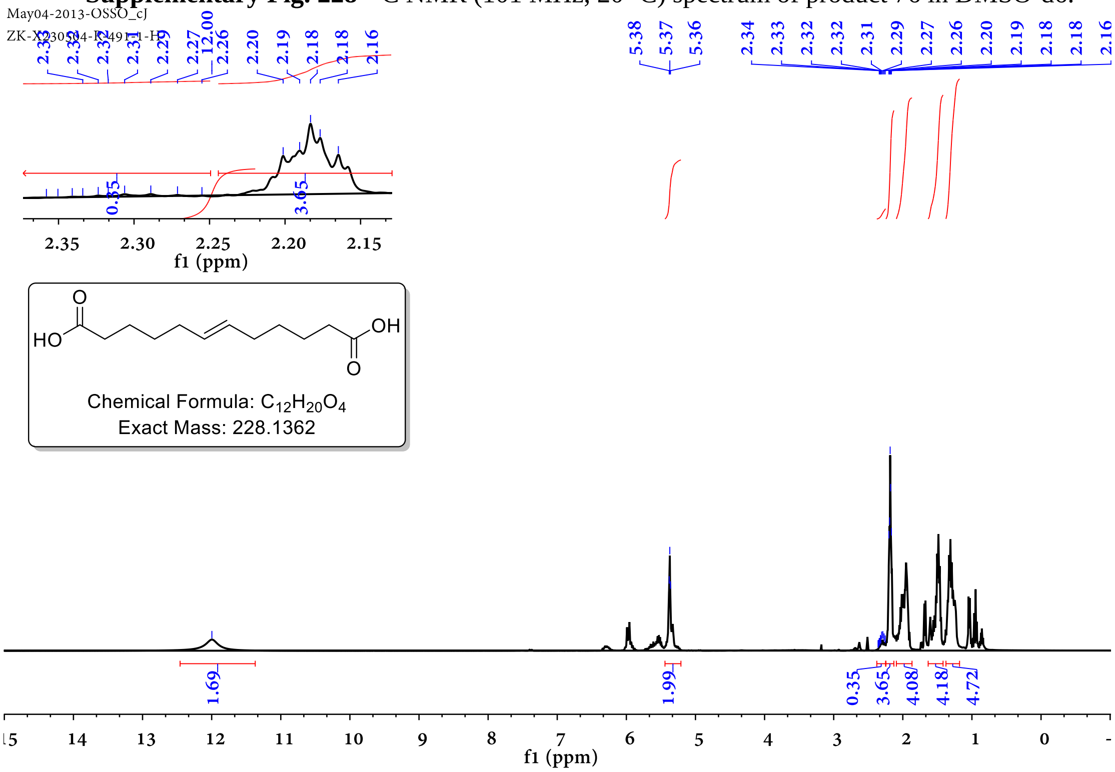

Supplementary Fig. 229 ^{1}H NMR (400 MHz, 20 ^{o}C) spectrum of product 77 in DMSO-d6.

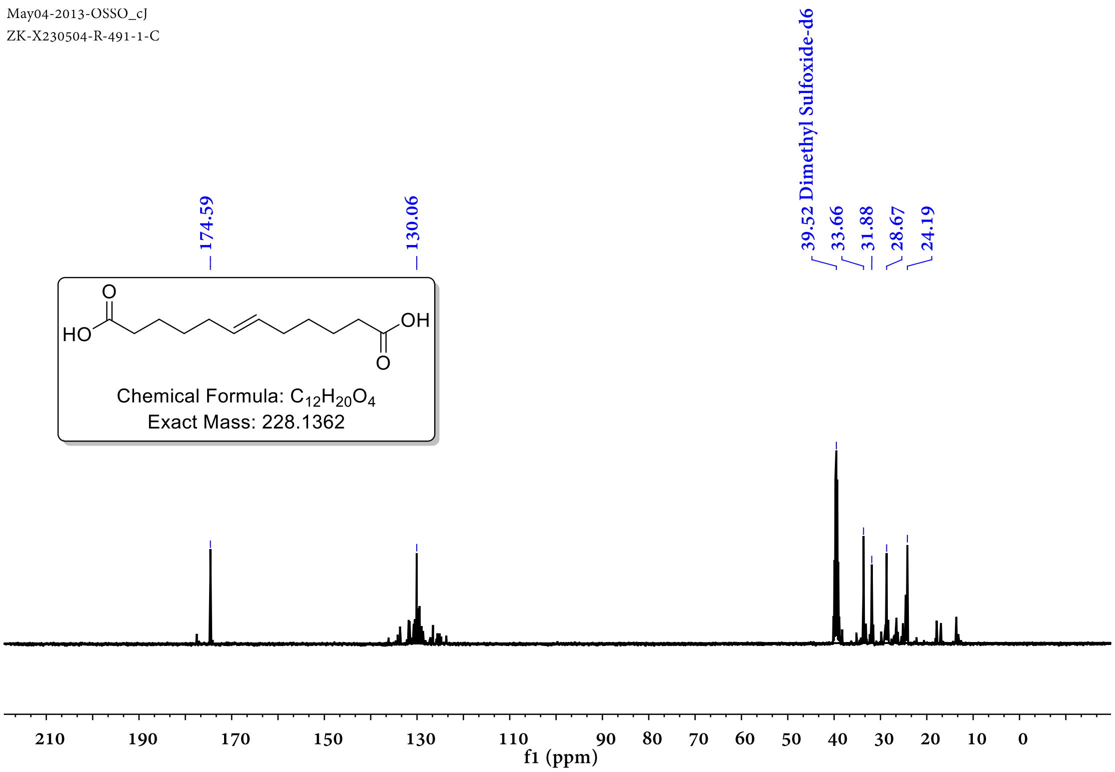

Supplementary Fig. 230 ^{13}C NMR (101 MHz, 20 °C) spectrum of product 77 in DMSO-d6.

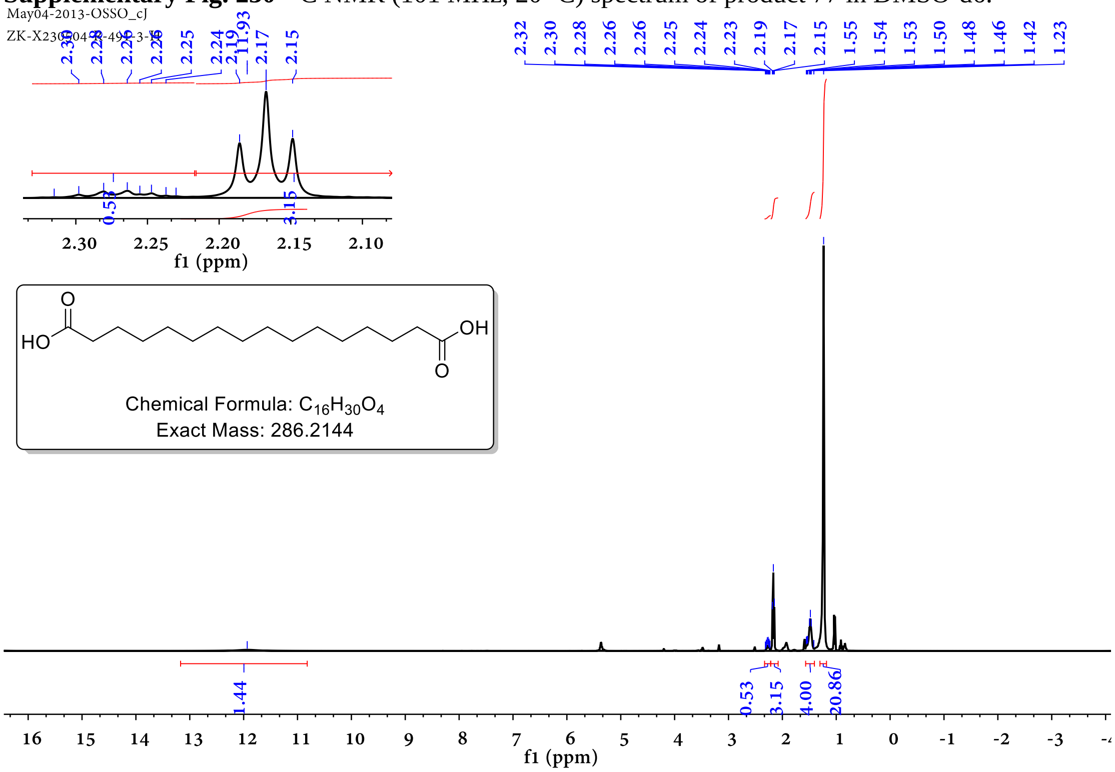

Supplementary Fig. 231 ^{1}H NMR (400 MHz, 20 °C) spectrum of product 78 in DMSO-d6.

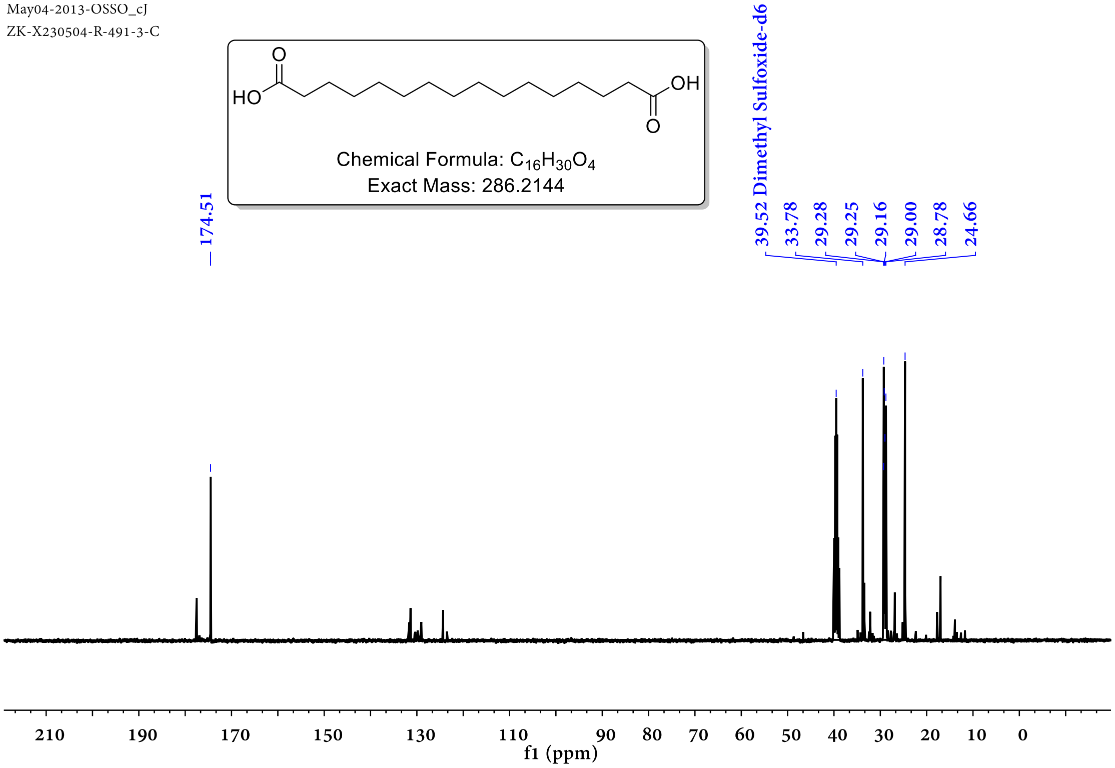

Supplementary Fig. 232 ^{13}C NMR (101 MHz, 20 °C) spectrum of product 78 in DMSO-d6.

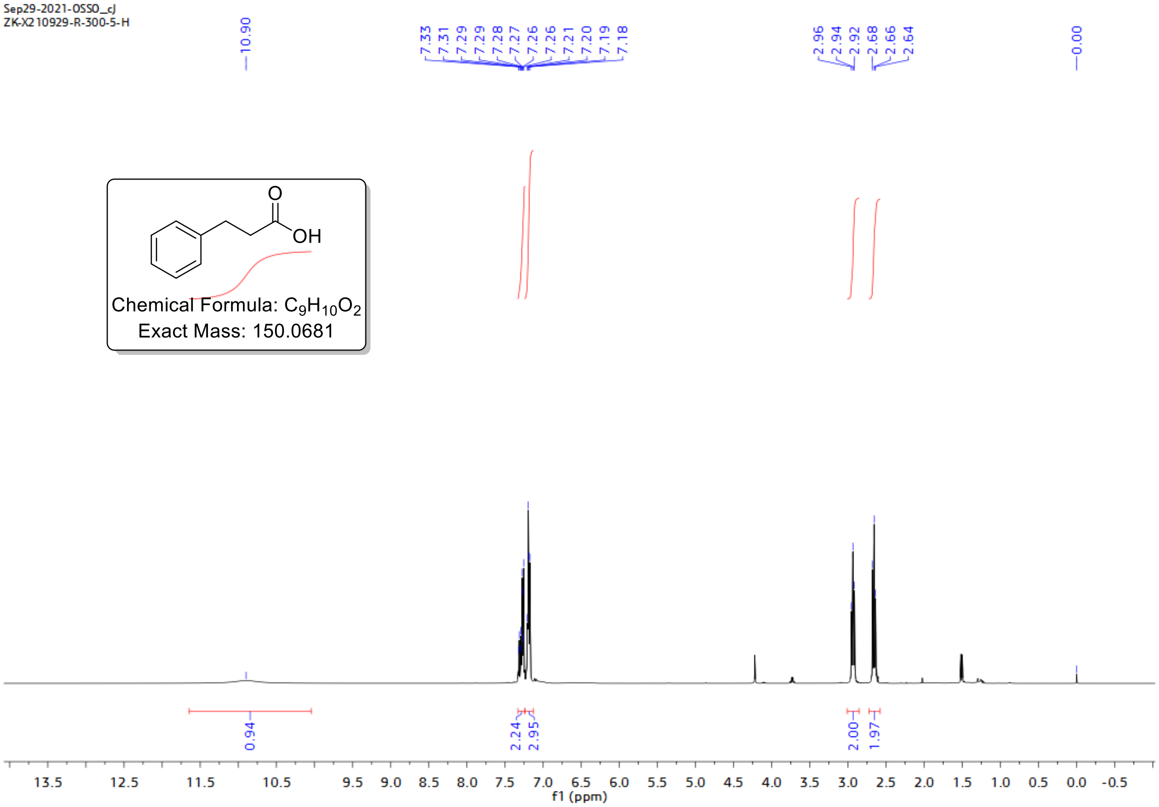

Supplementary Fig. 233 ^{1}H NMR (400 MHz, 20 °C) spectrum of product 79 in DMSO-d6.

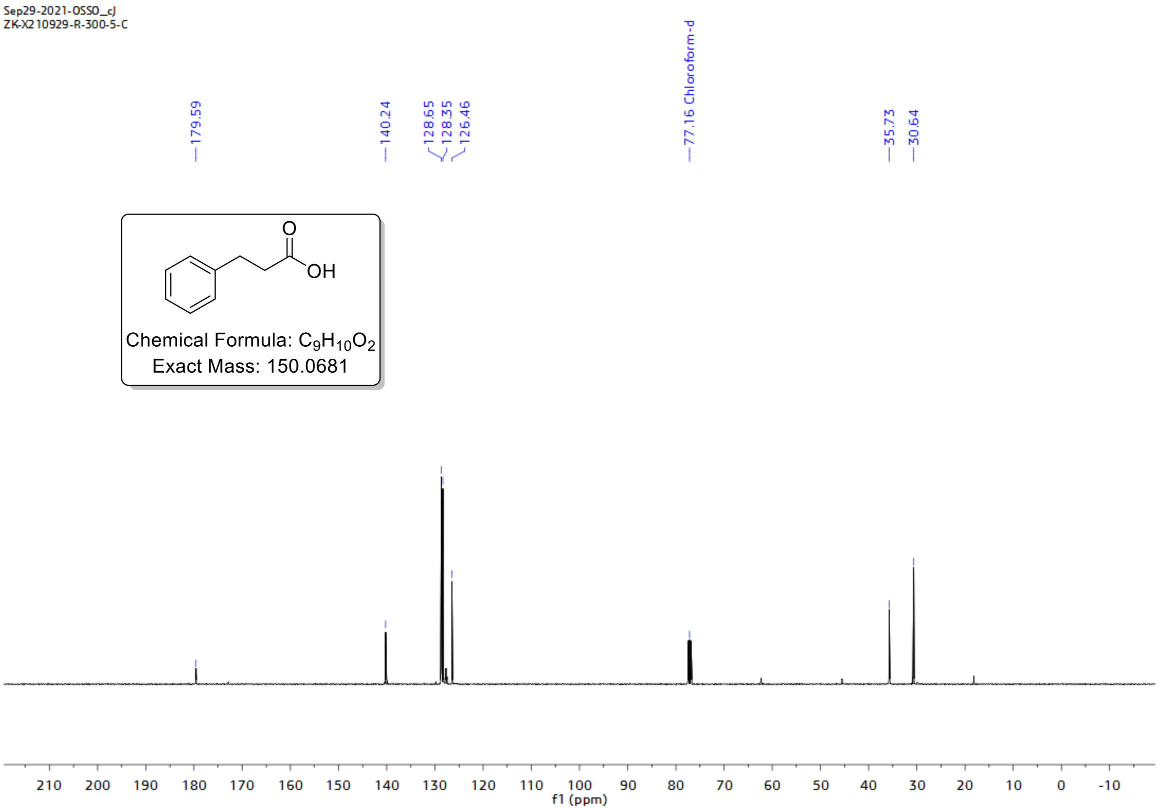

Supplementary Fig. 234 ^{13}C NMR (101 MHz, 20 ºC) spectrum of product 79 in $CDCl_3$.

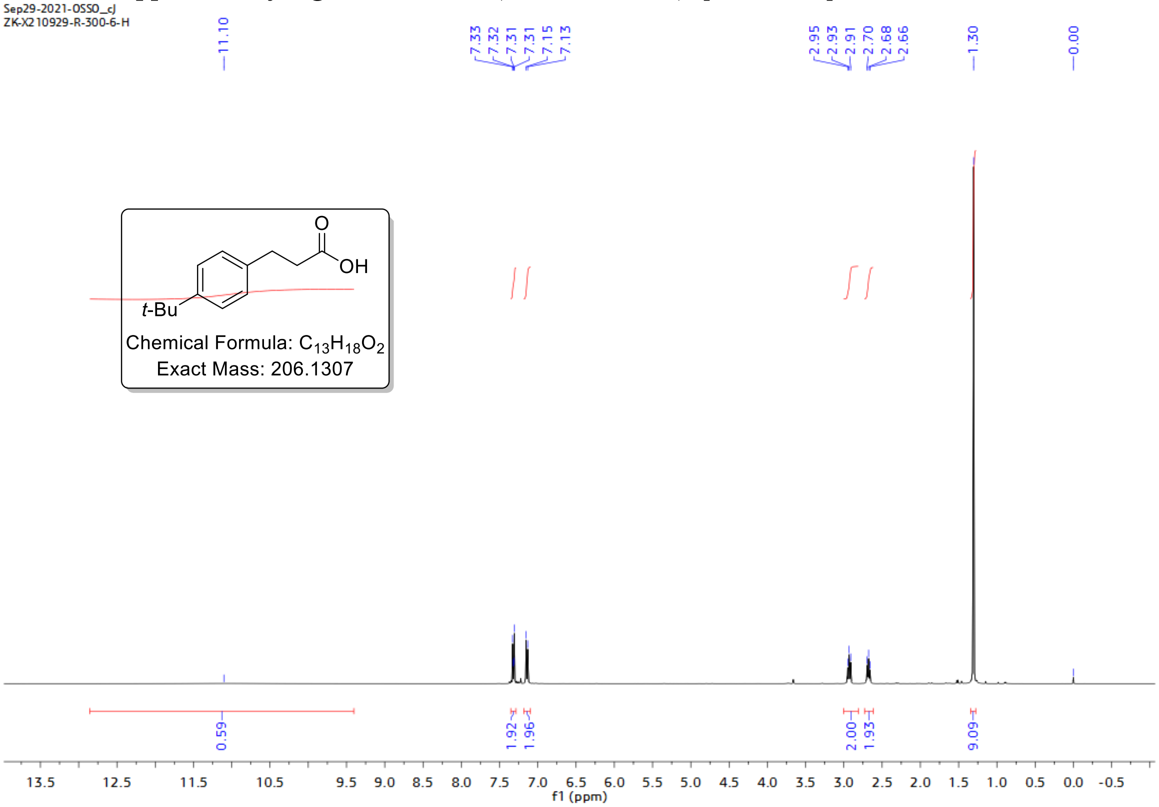

Supplementary Fig. 235 ^{1}H NMR (400 MHz, 20 ºC) spectrum of product 80 in $CDCl_3$.

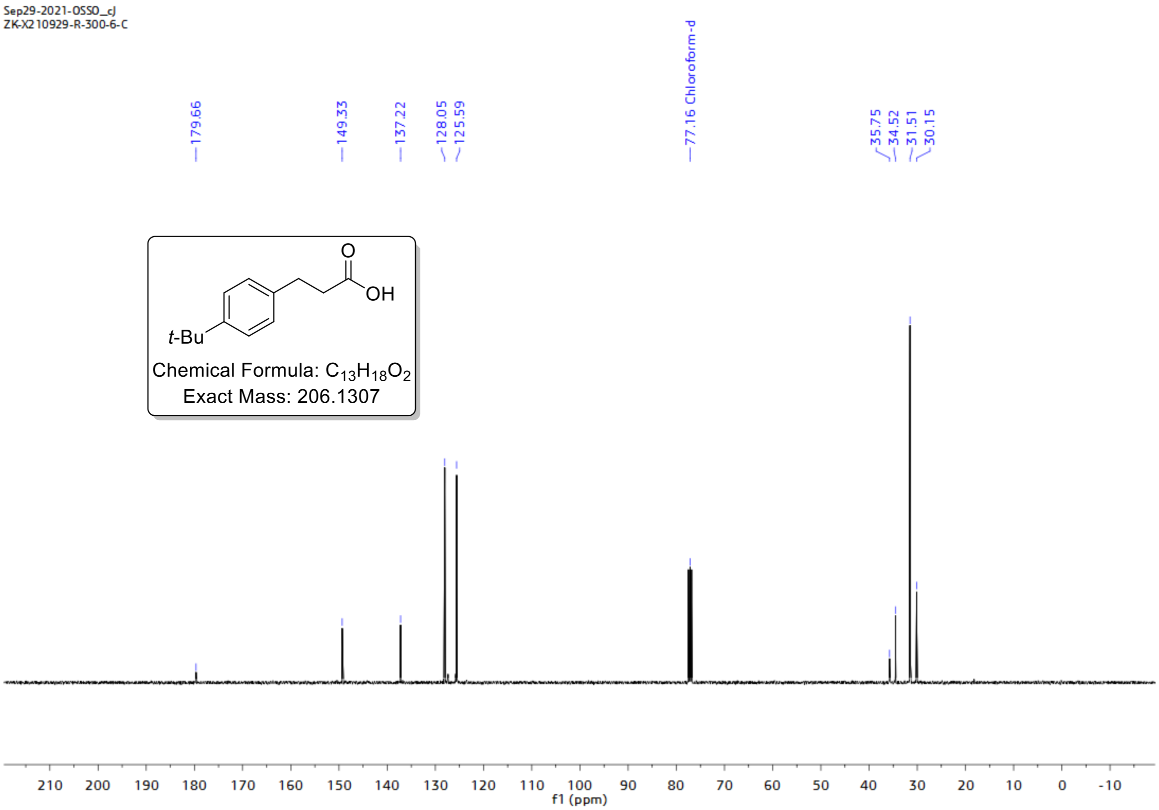

Supplementary Fig. 236 ^{13}C NMR (101 MHz, 20 ºC) spectrum of product 80 in $CDCl_3$.

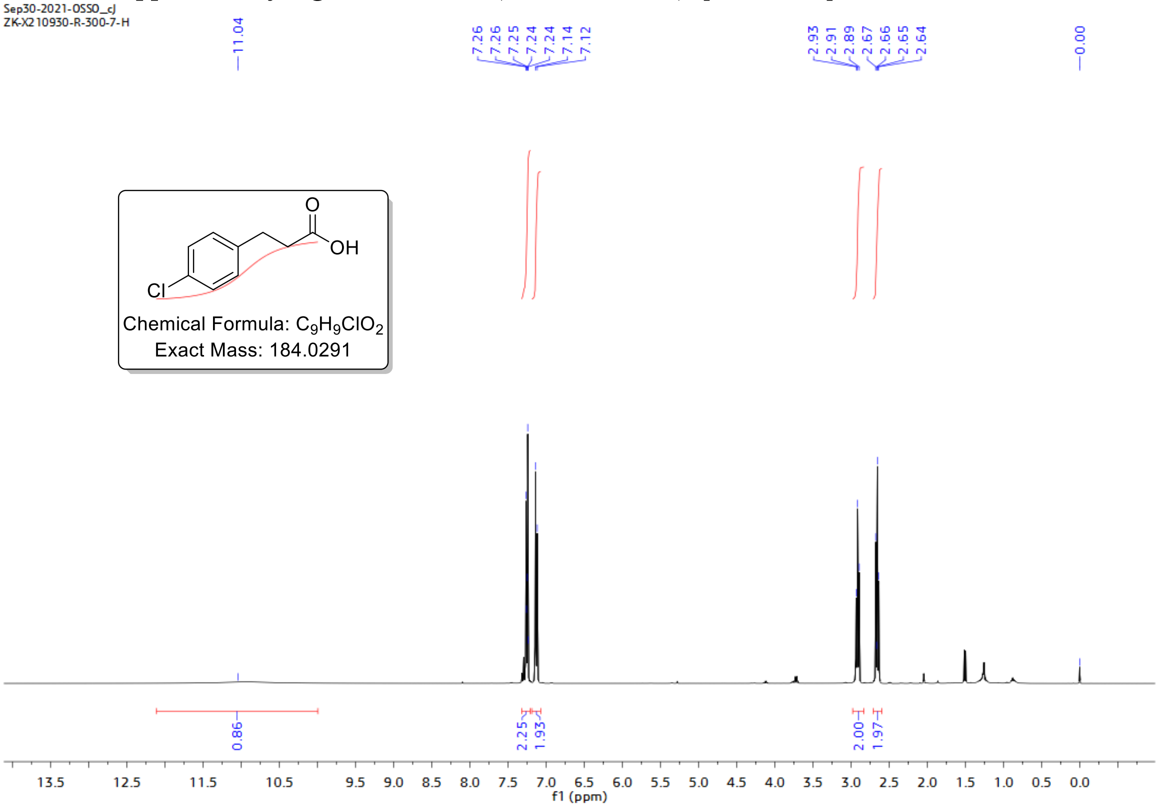

Supplementary Fig. 237 ^{1}H NMR (400 MHz, 20 ºC) spectrum of product 81 in $CDCl_3$.

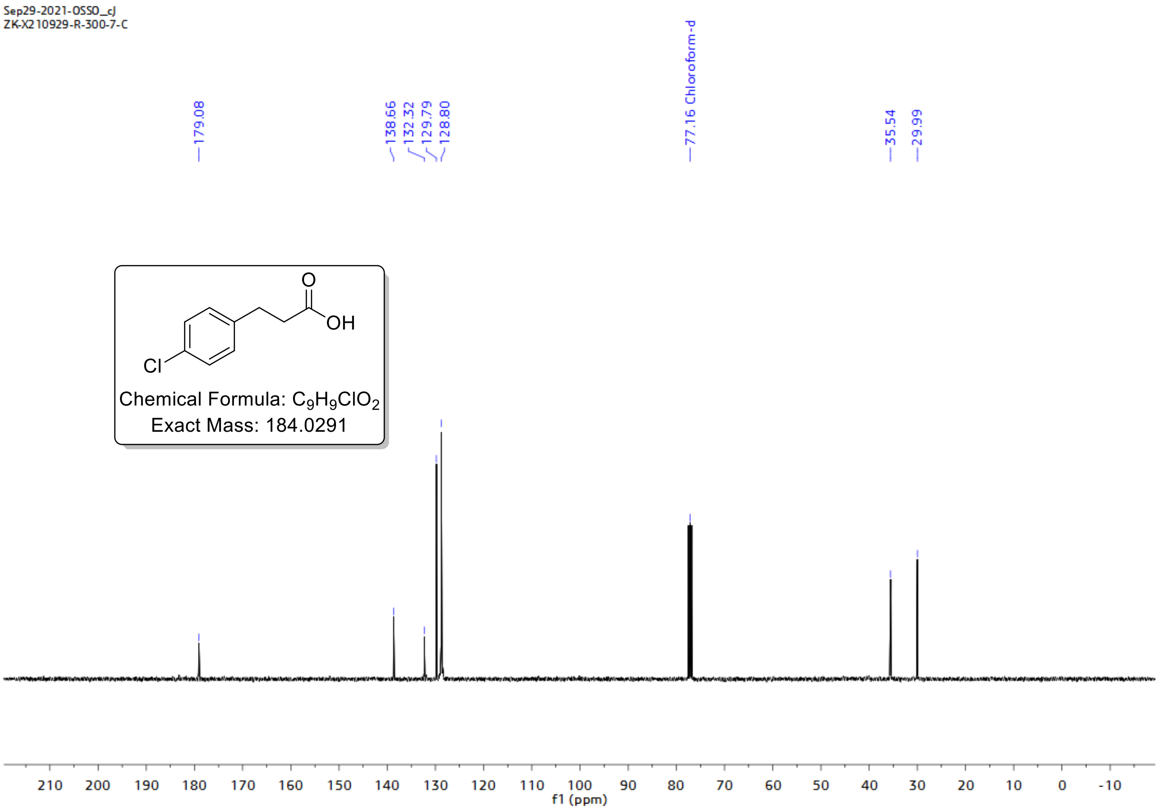

Supplementary Fig. 238 ^{13}C NMR (101 MHz, 20 ºC) spectrum of product 81 in $CDCl_3$.

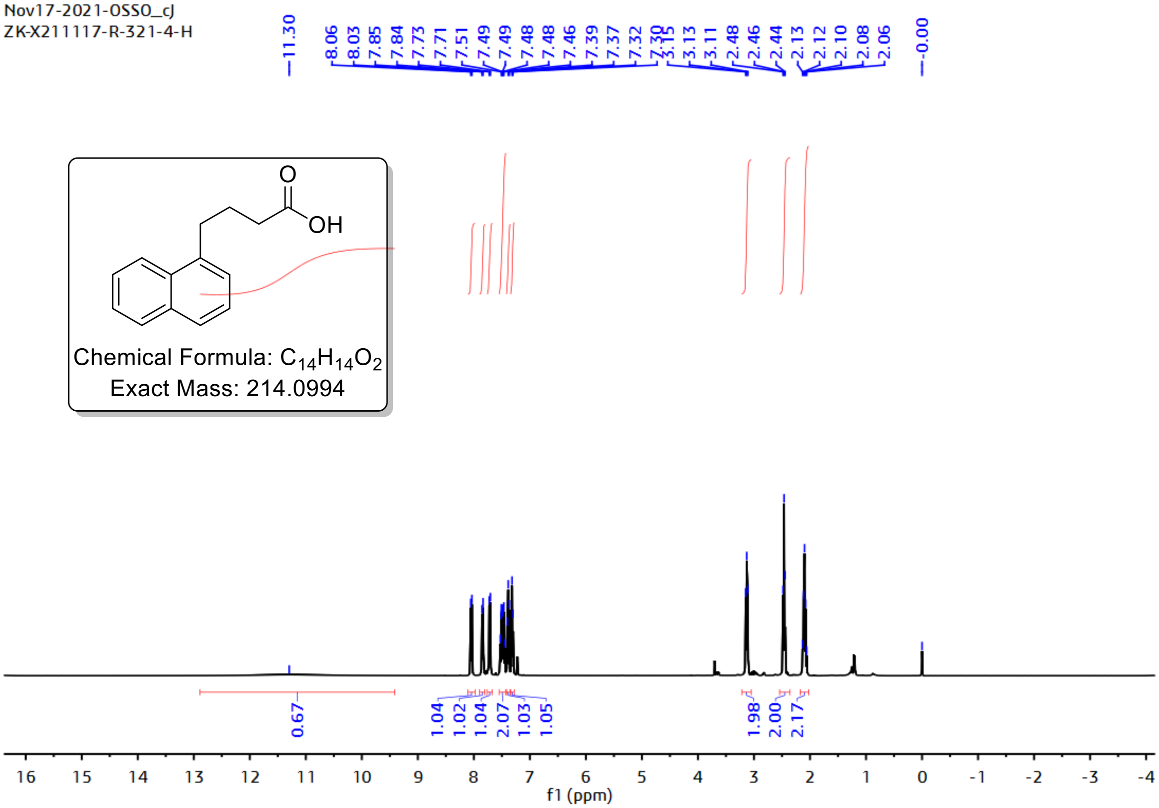

Supplementary Fig. 239 ^{1}H NMR (400 MHz, 20 ºC) spectrum of product 82 in $CDCl_3$.

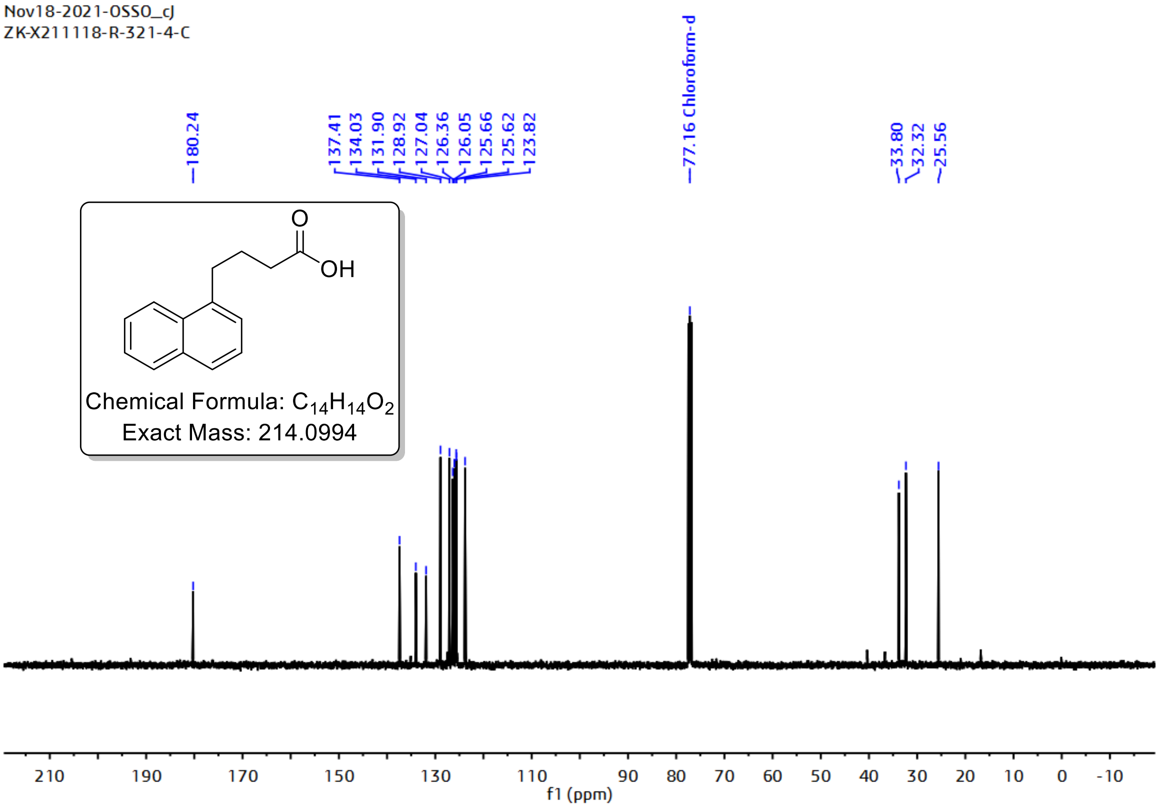

Supplementary Fig. 240 ^{13}C NMR (101 MHz, 20 ºC) spectrum of product 82 in $CDCl_3$.

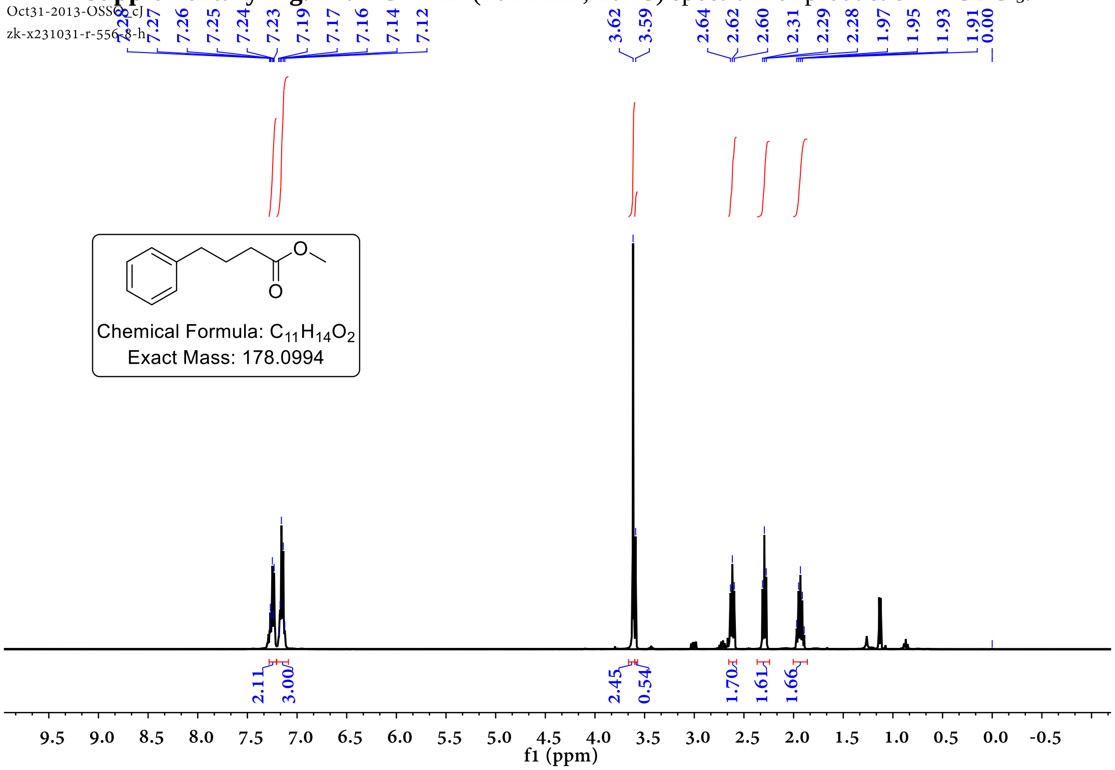

Supplementary Fig. 241 ^{1}H NMR (400 MHz, 20 ºC) spectrum of product 89 in $CDCl_3$.

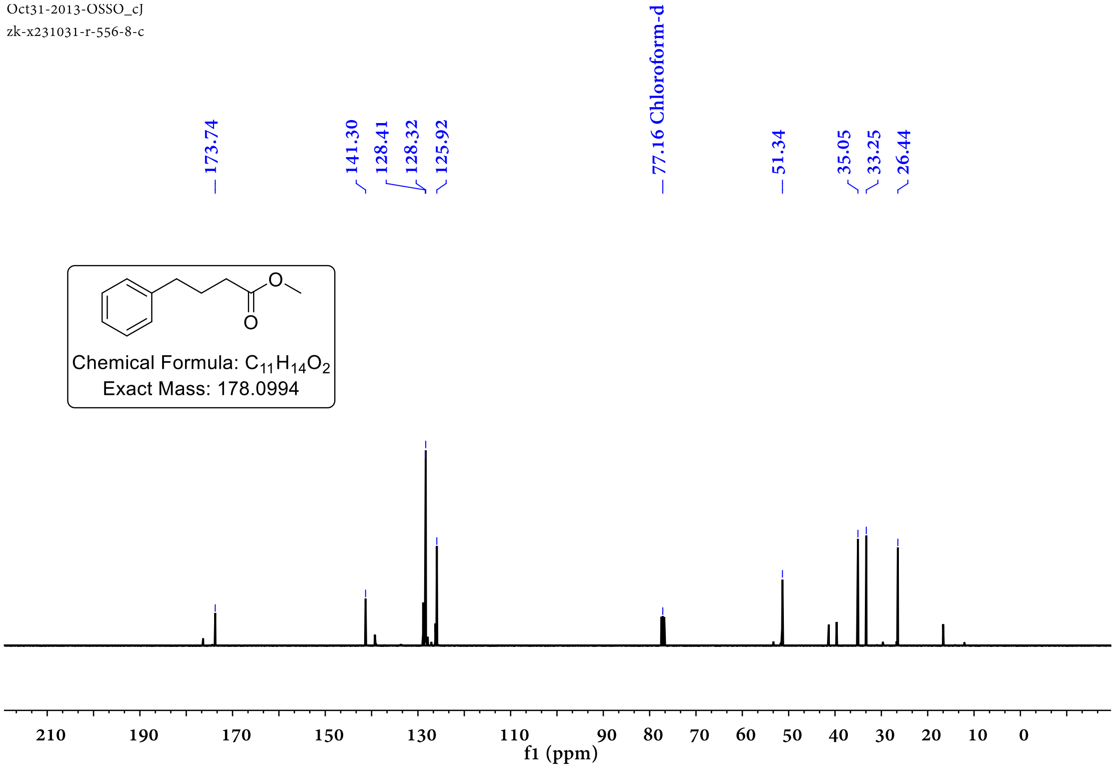

Supplementary Fig. 242 ^{13}C NMR (101 MHz, 20 °C) spectrum of product 89 in $CDCl_3$.

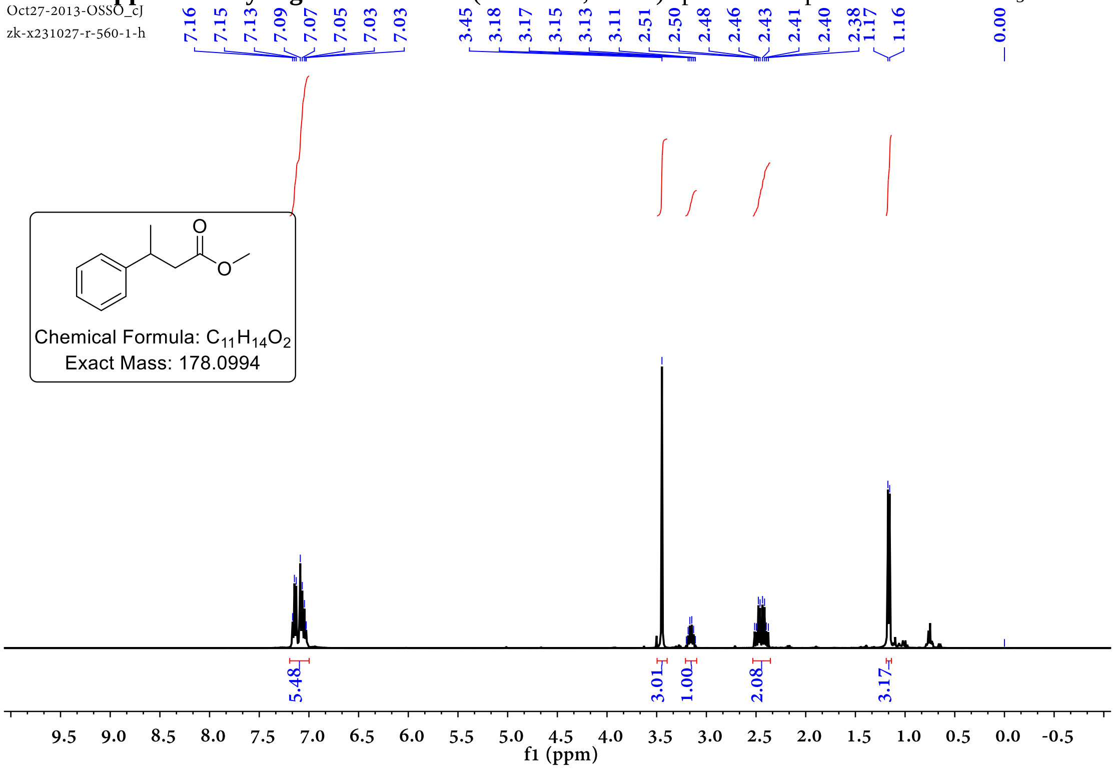

Supplementary Fig. 243 ^{1}H NMR (400 MHz, 20 °C) spectrum of product 93 in $CDCl_3$.

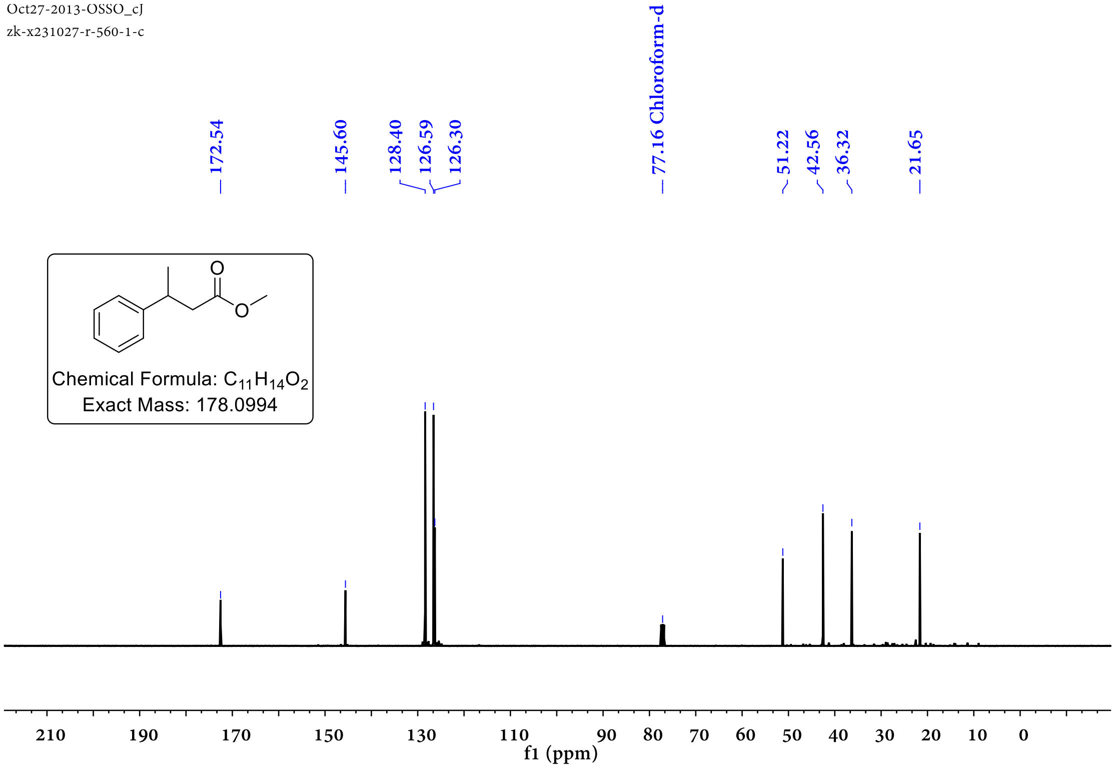

Supplementary Fig. 244 ^{13}C NMR (101 MHz, 20 °C) spectrum of product 93 in $CDCl_3$.

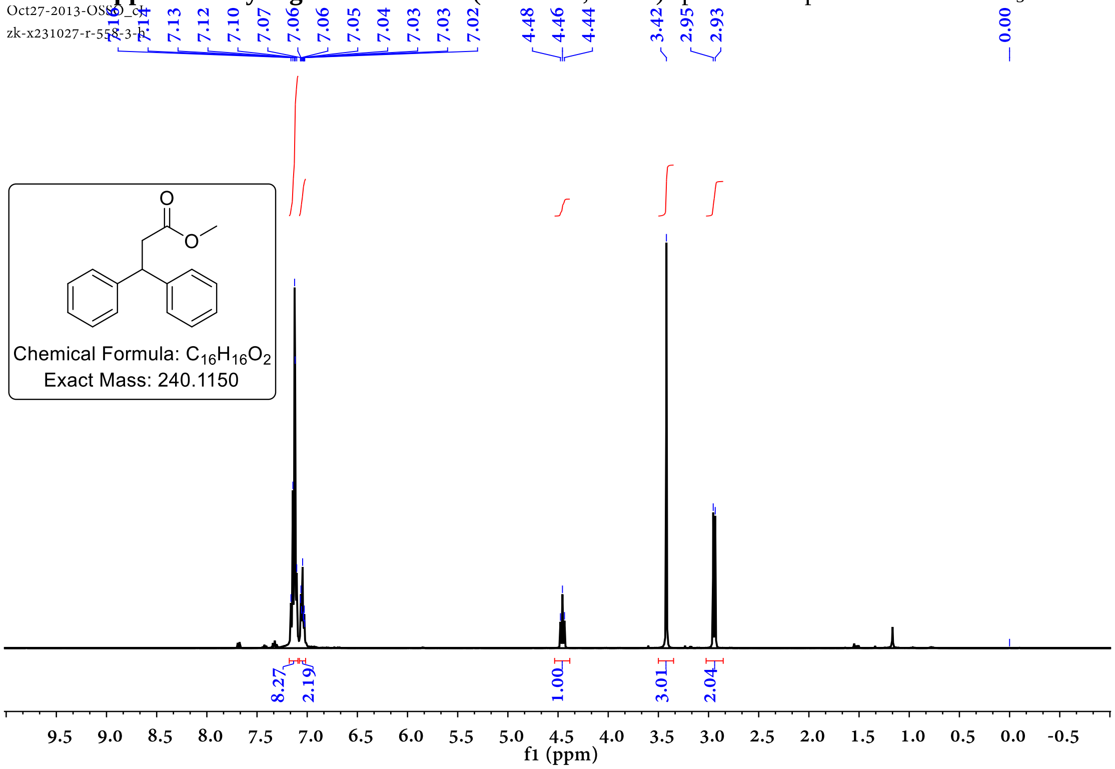

Supplementary Fig. 245 ^{1}H NMR (400 MHz, 20 °C) spectrum of product 94 in $CDCl_3$.

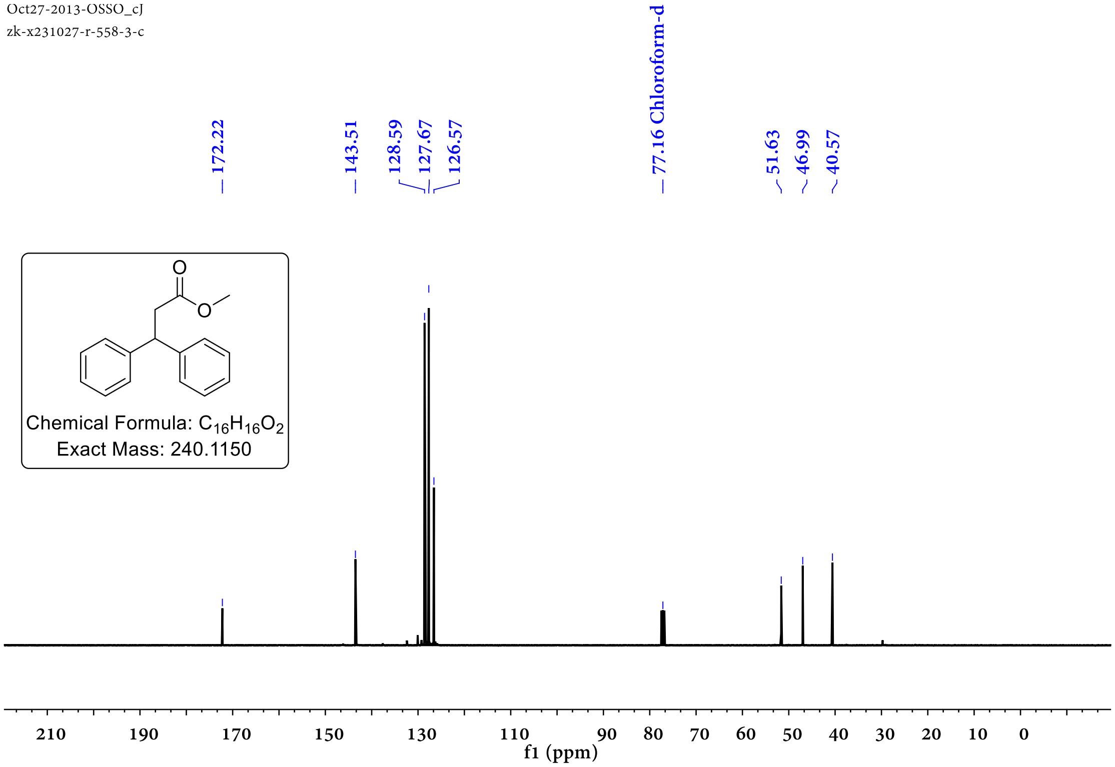

Supplementary Fig. 246 ^{13}C NMR (101 MHz, 20 °C) spectrum of product 94 in $CDCl_3$.

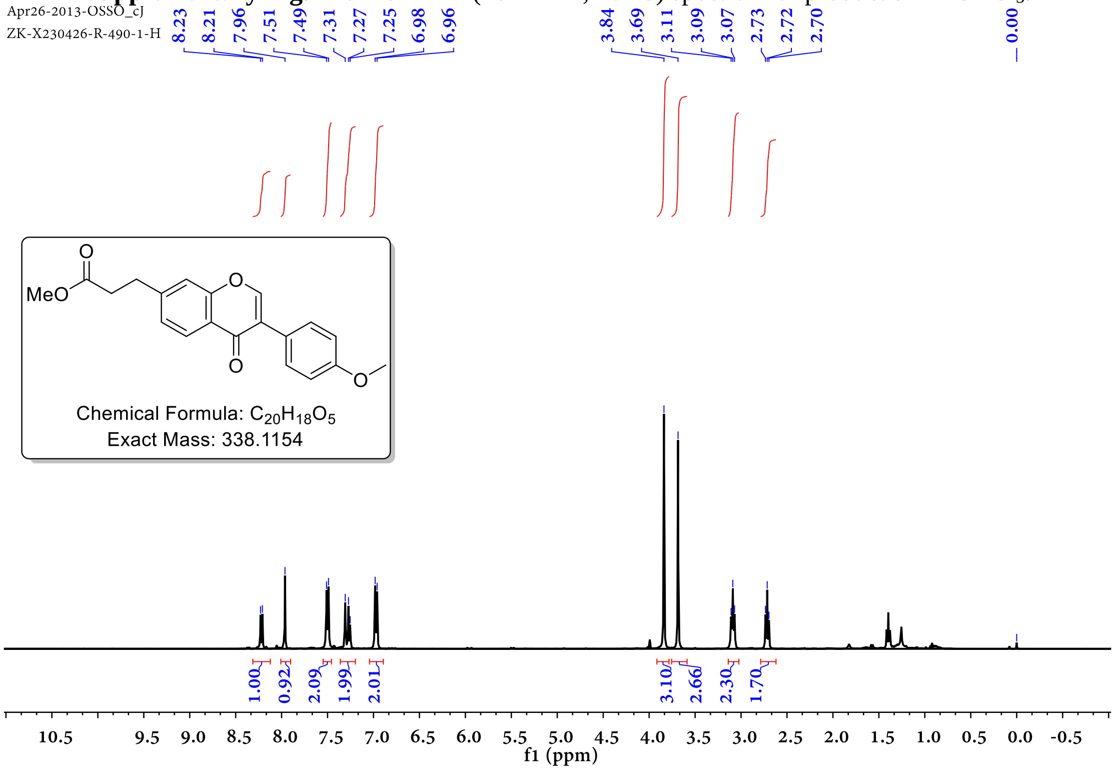

Supplementary Fig. 247 ^{1}H NMR (400 MHz, 20 °C) spectrum of product 96 in $CDCl_3$.

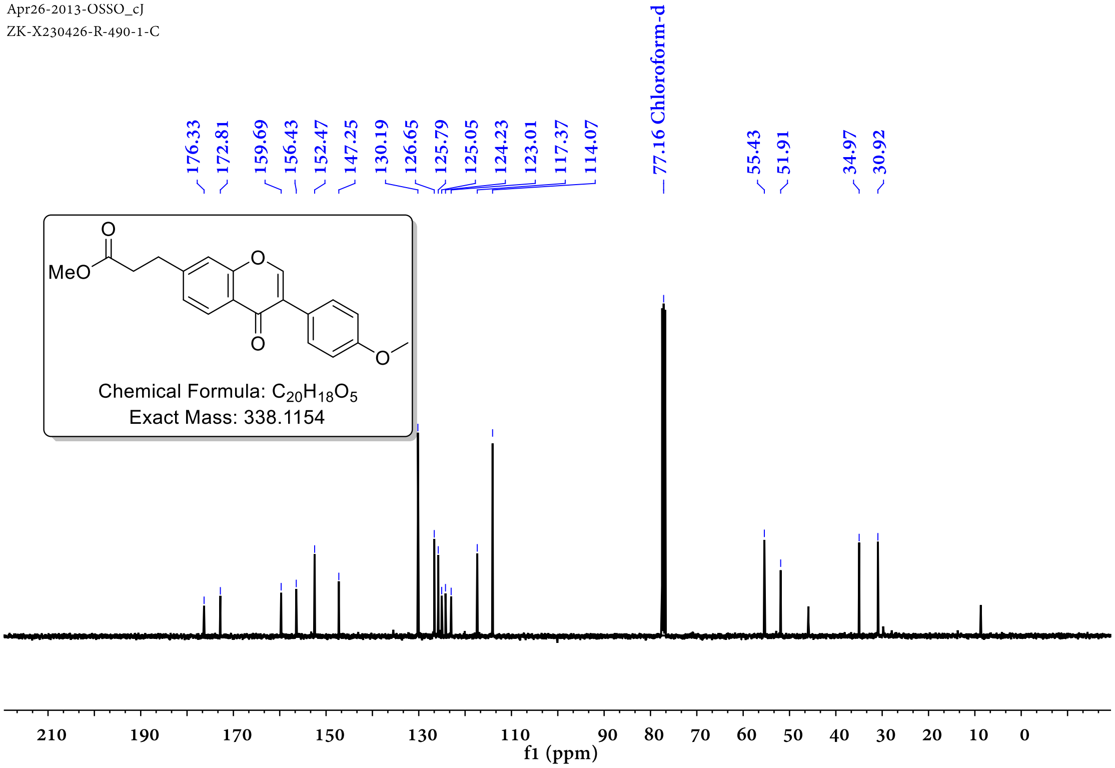

Supplementary Fig. 248 ^{13}C NMR (101 MHz, 20 ºC) spectrum of product 96 in $CDCl_3$.

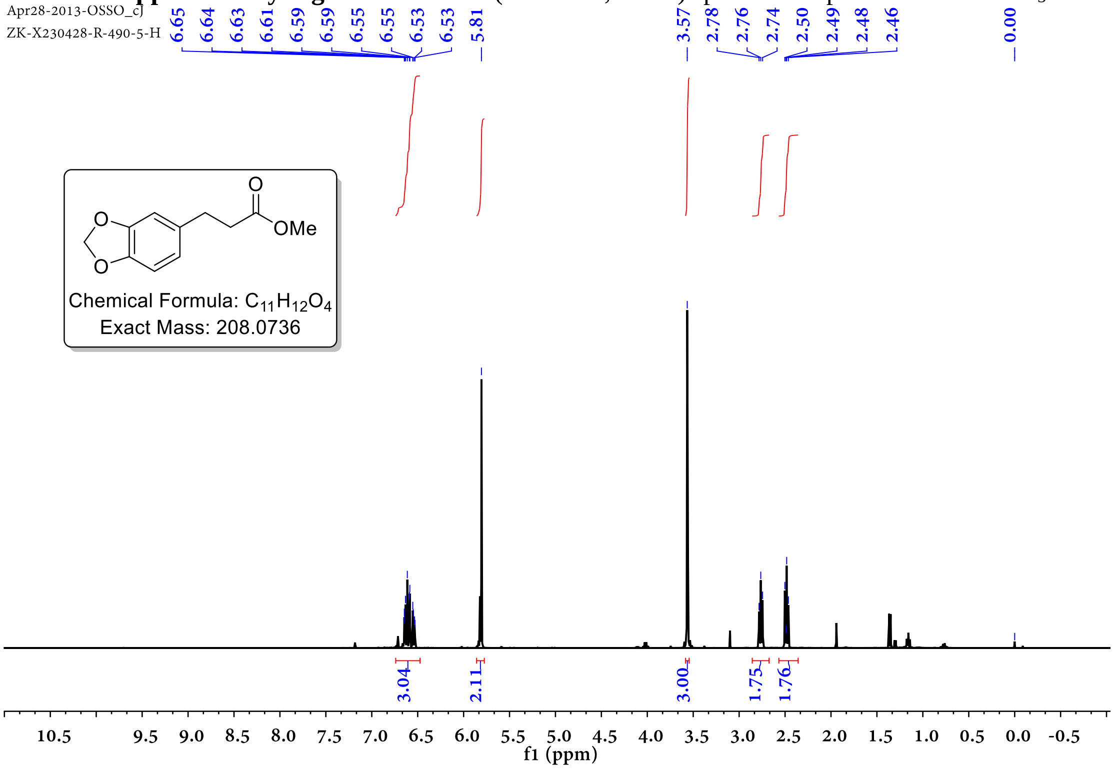

Supplementary Fig. 249 ^{1}H NMR (400 MHz, 20 ºC) spectrum of product 97 in $CDCl_3$.

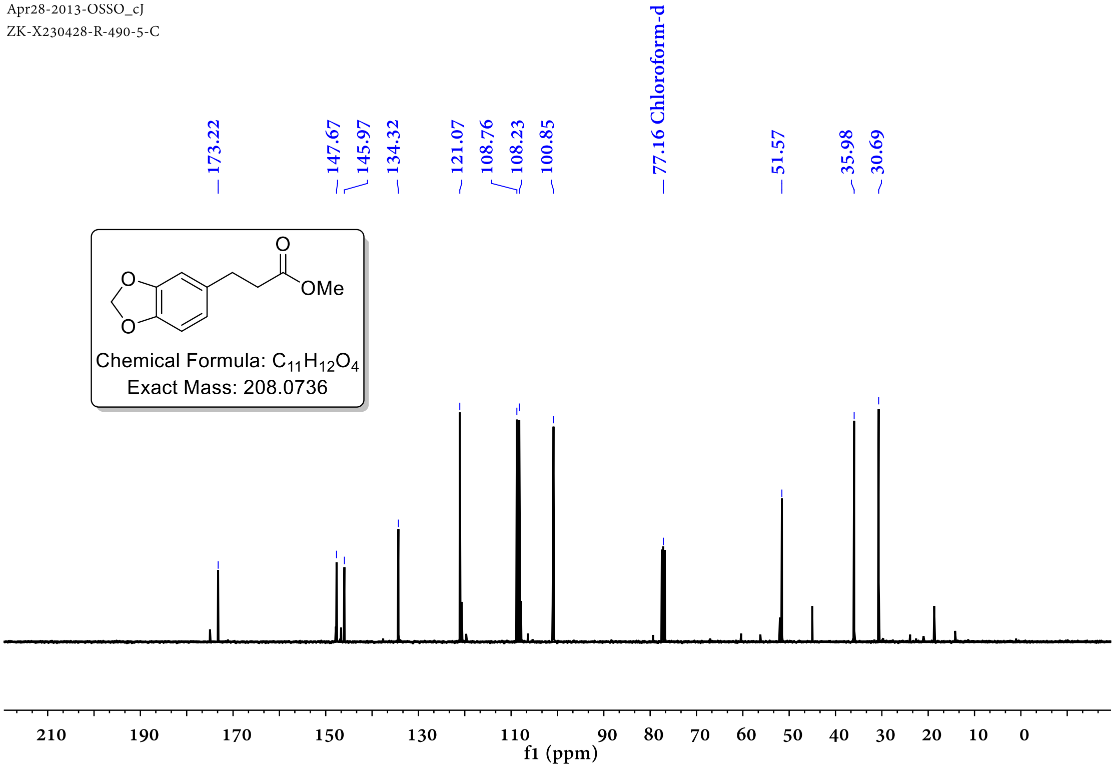

Supplementary Fig. 250 ^{13}C NMR (101 MHz, 20 °C) spectrum of product 97 in $CDCl_3$.

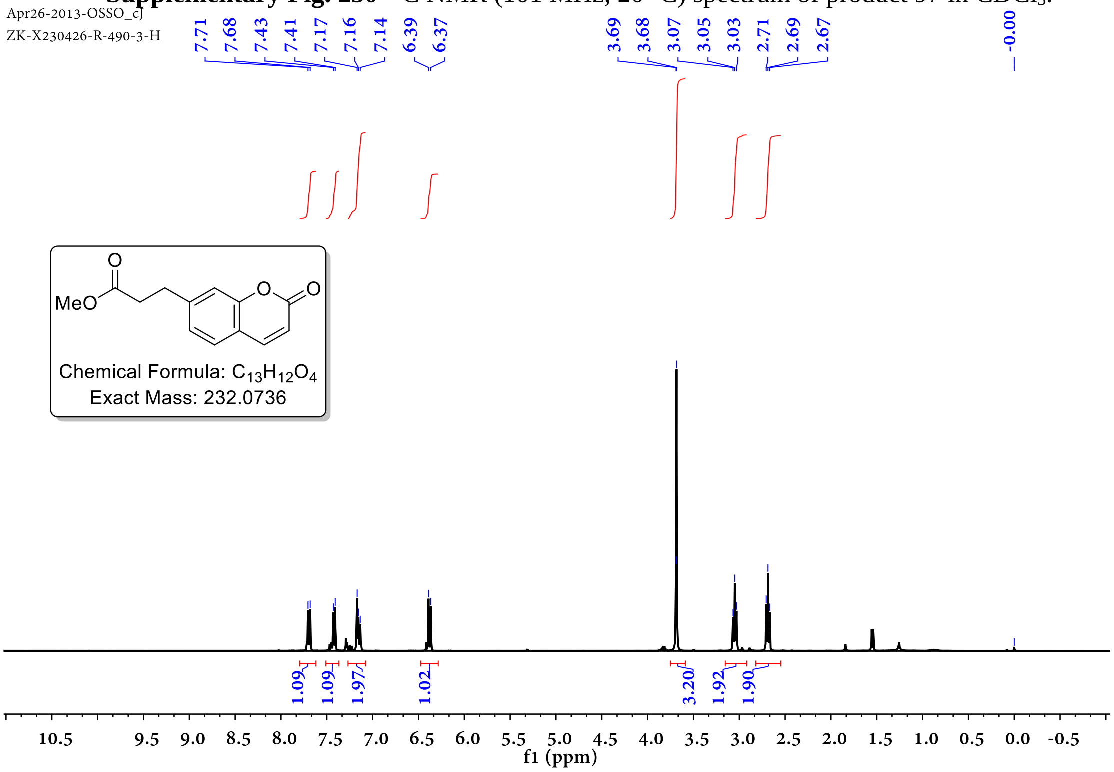

Supplementary Fig. 251 ^{1}H NMR (400 MHz, 20 °C) spectrum of product 98 in $CDCl_3$.

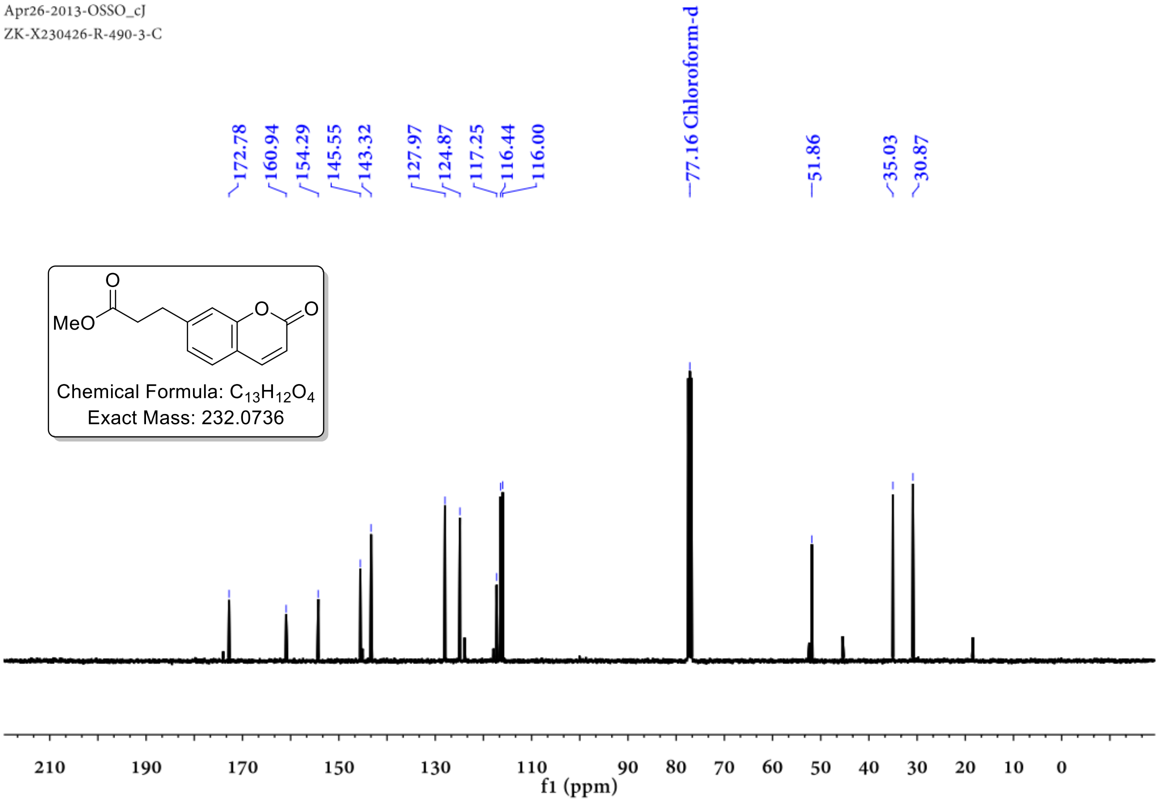

Supplementary Fig. 252 ^{13}C NMR (101 MHz, 20 ºC) spectrum of product 98 in $CDCl_3$.

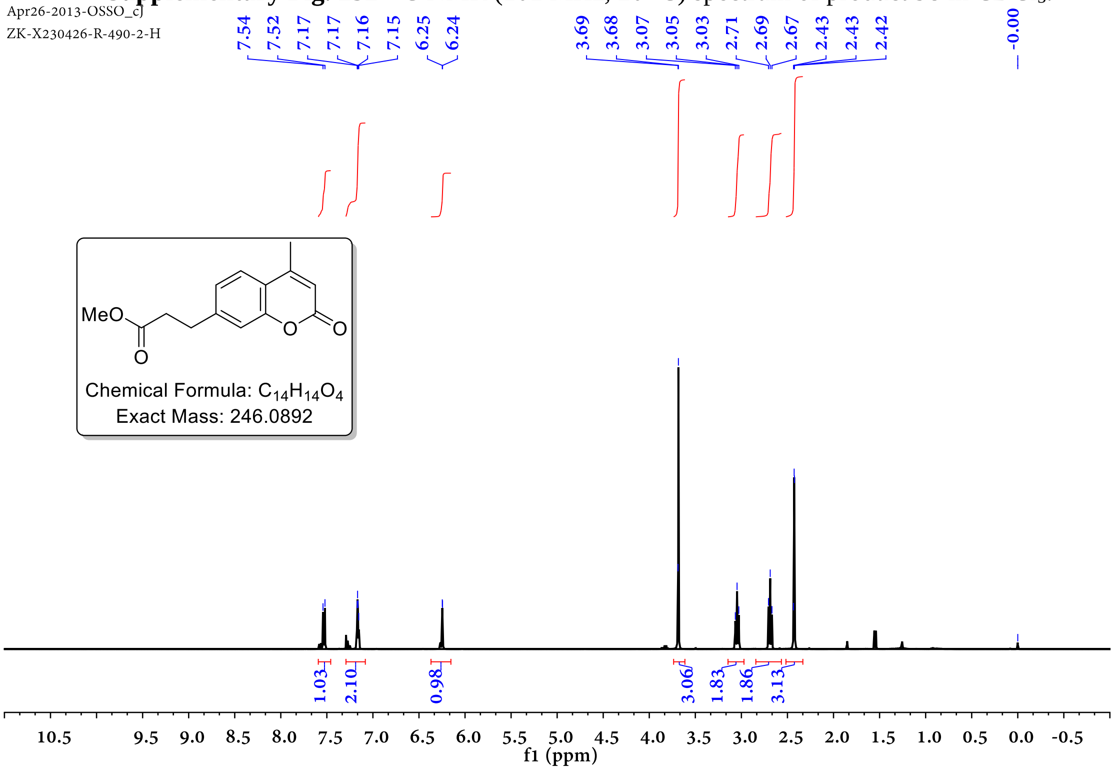

Supplementary Fig. 253 ^{1}H NMR (400 MHz, 20 ºC) spectrum of product 99 in $CDCl_3$.

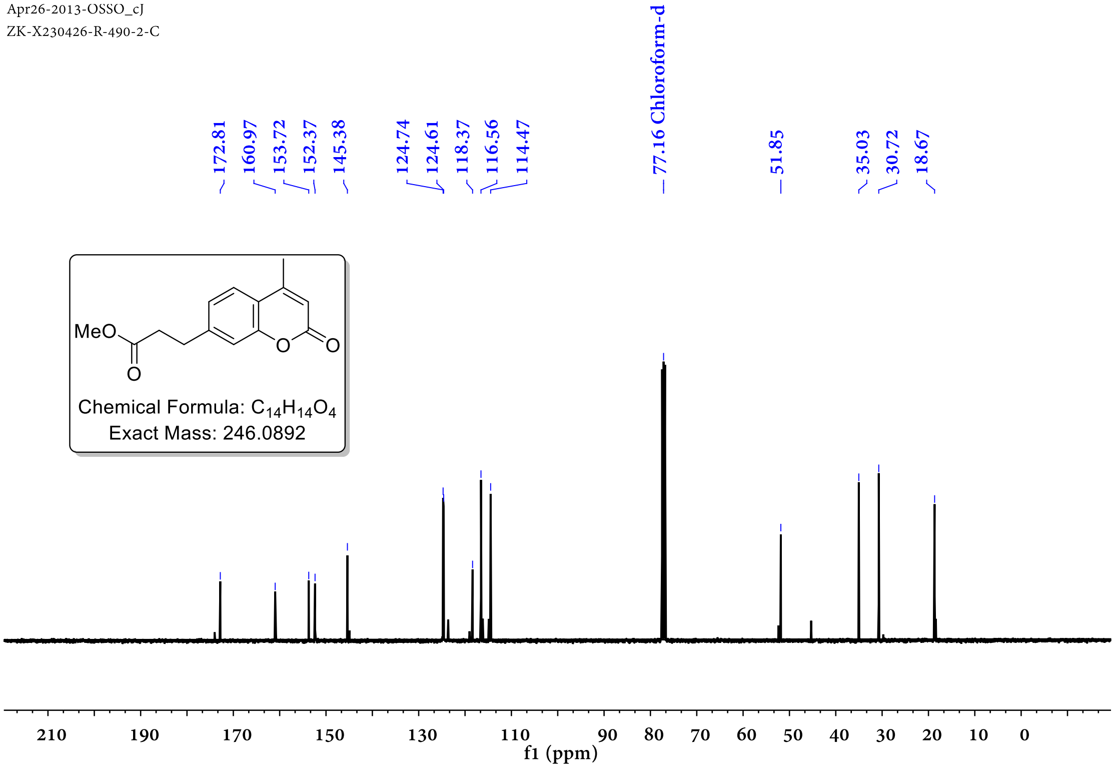

Supplementary Fig. 254 ^{13}C NMR (101 MHz, 20 °C) spectrum of product 99 in $CDCl_3$.

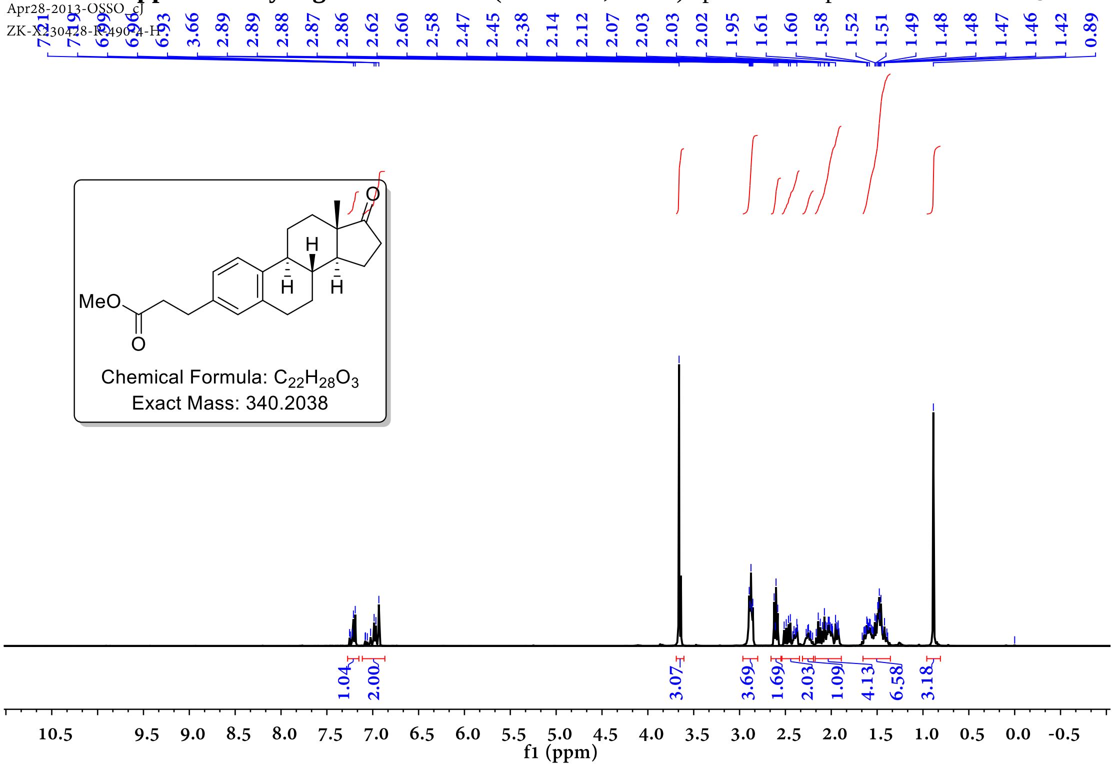

Supplementary Fig. 255 ^{1}H NMR (400 MHz, 20 °C) spectrum of product 100 in $CDCl_3$.

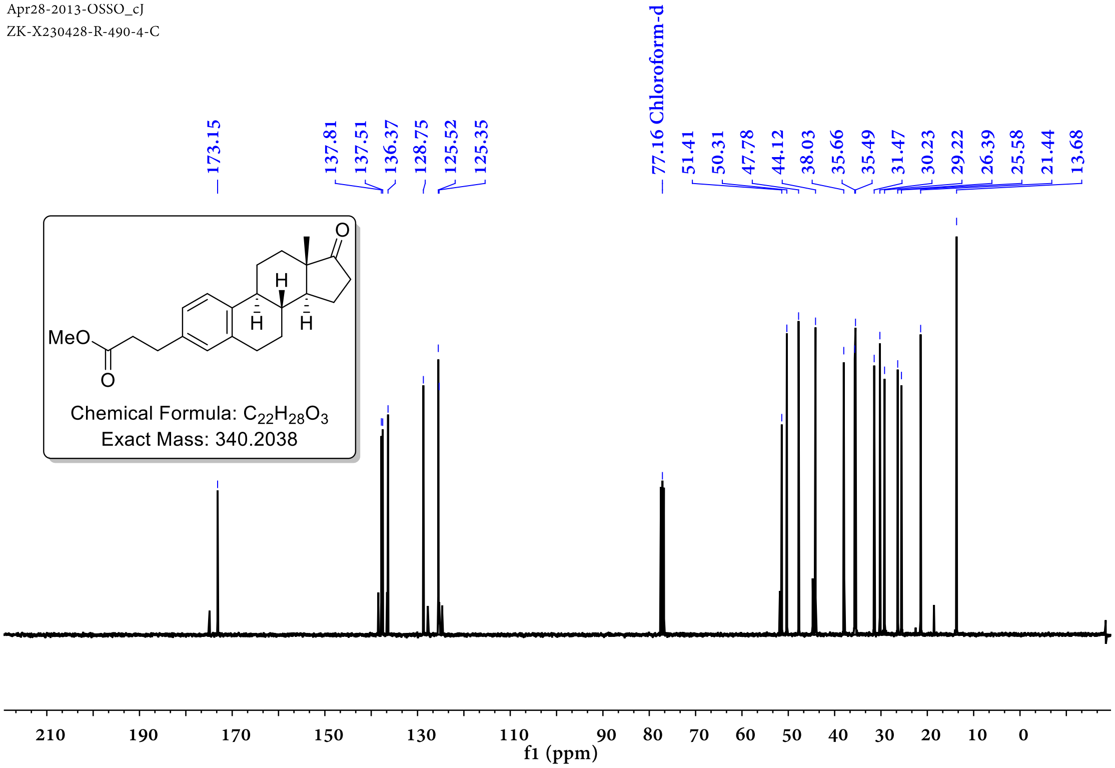

Supplementary Fig. 256 ^{13}C NMR (101 MHz, 20 ºC) spectrum of product 100 in $CDCl_3$.

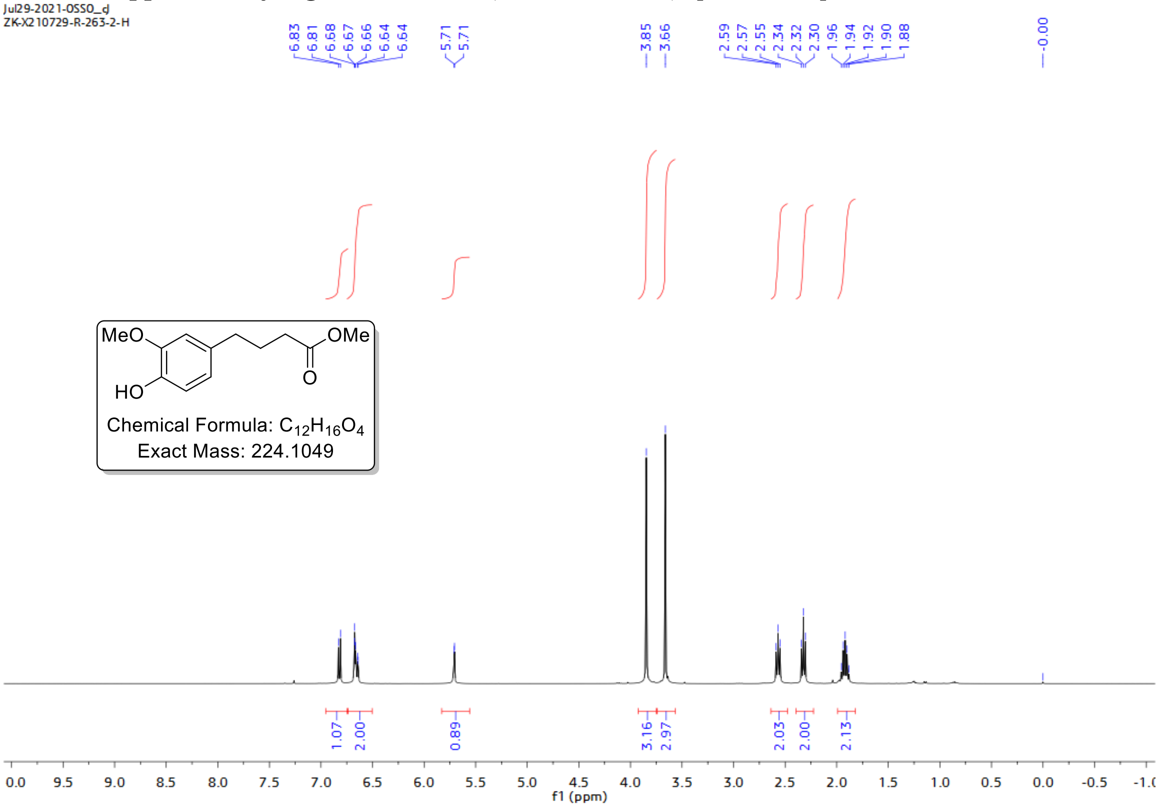

Supplementary Fig. 257 ^{1}H NMR (400 MHz, 20 ºC) spectrum of product 101 in $CDCl_3$.

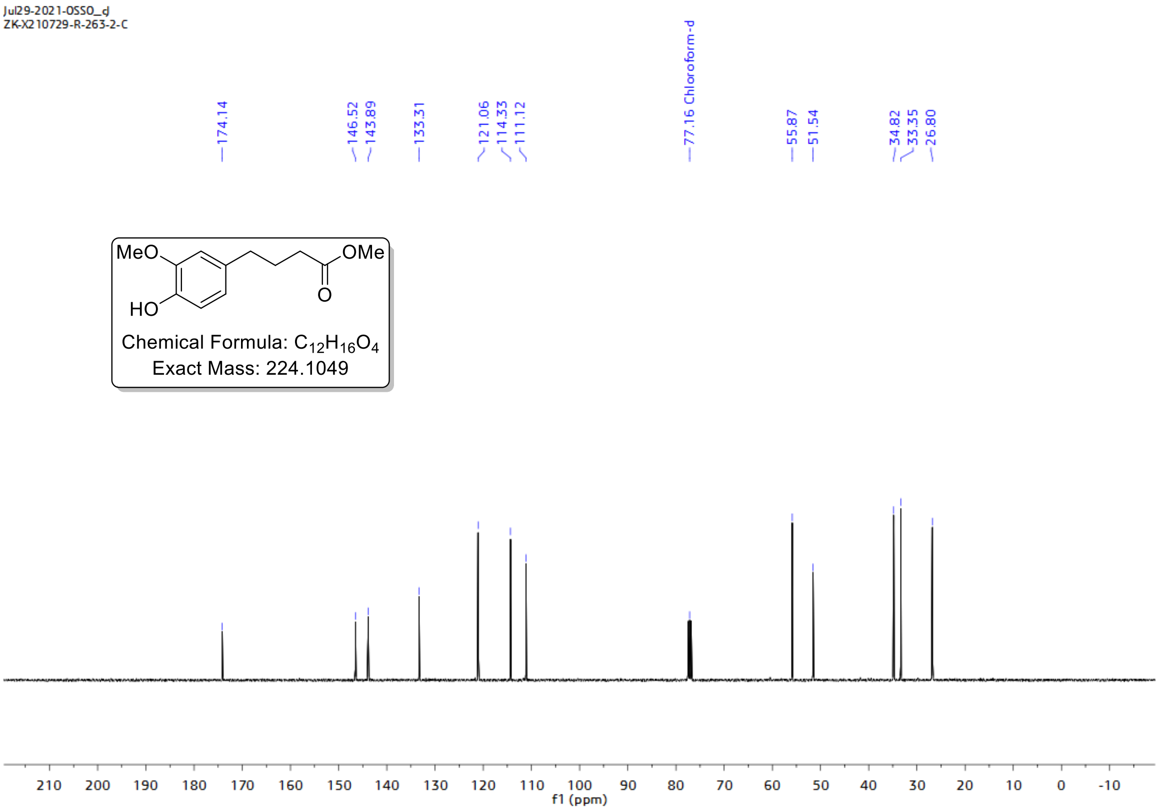

Supplementary Fig. 258 ^{13}C NMR (101 MHz, 20 °C) spectrum of product 101 in $CDCl_3$.

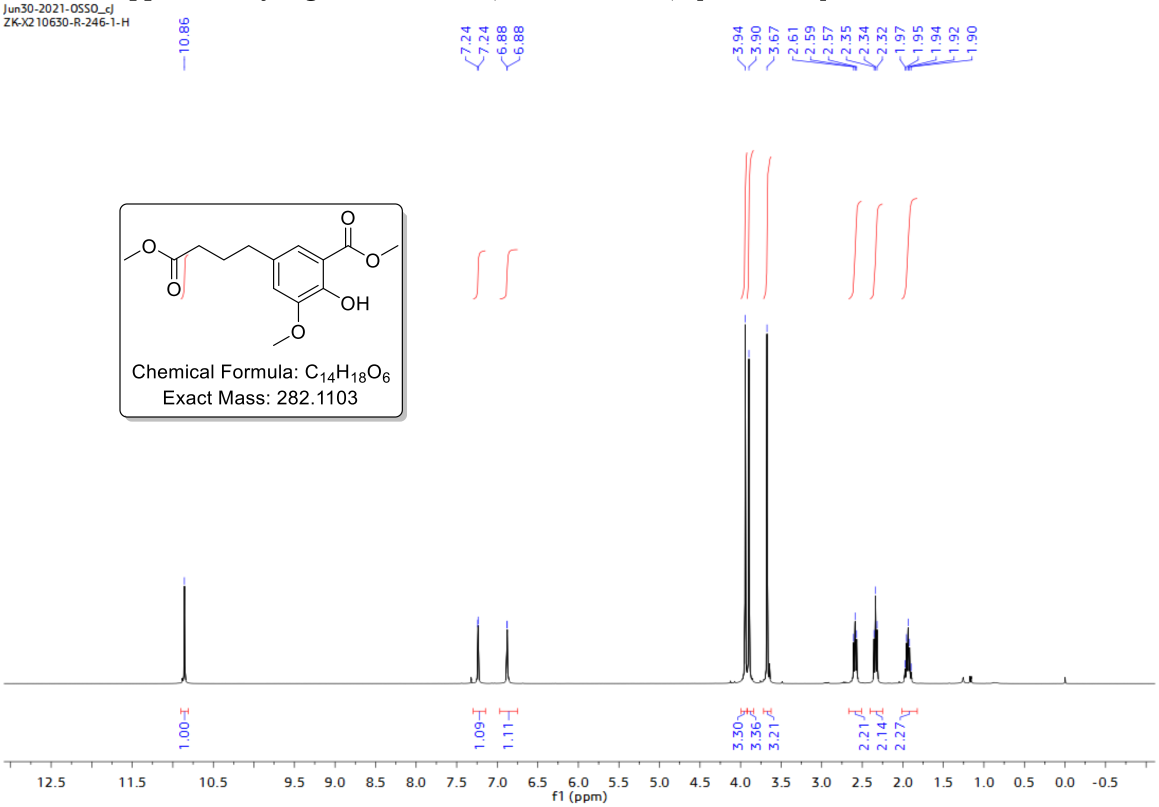

Supplementary Fig. 259 ^{1}H NMR (400 MHz, 20 °C) spectrum of product 102 in $CDCl_3$.

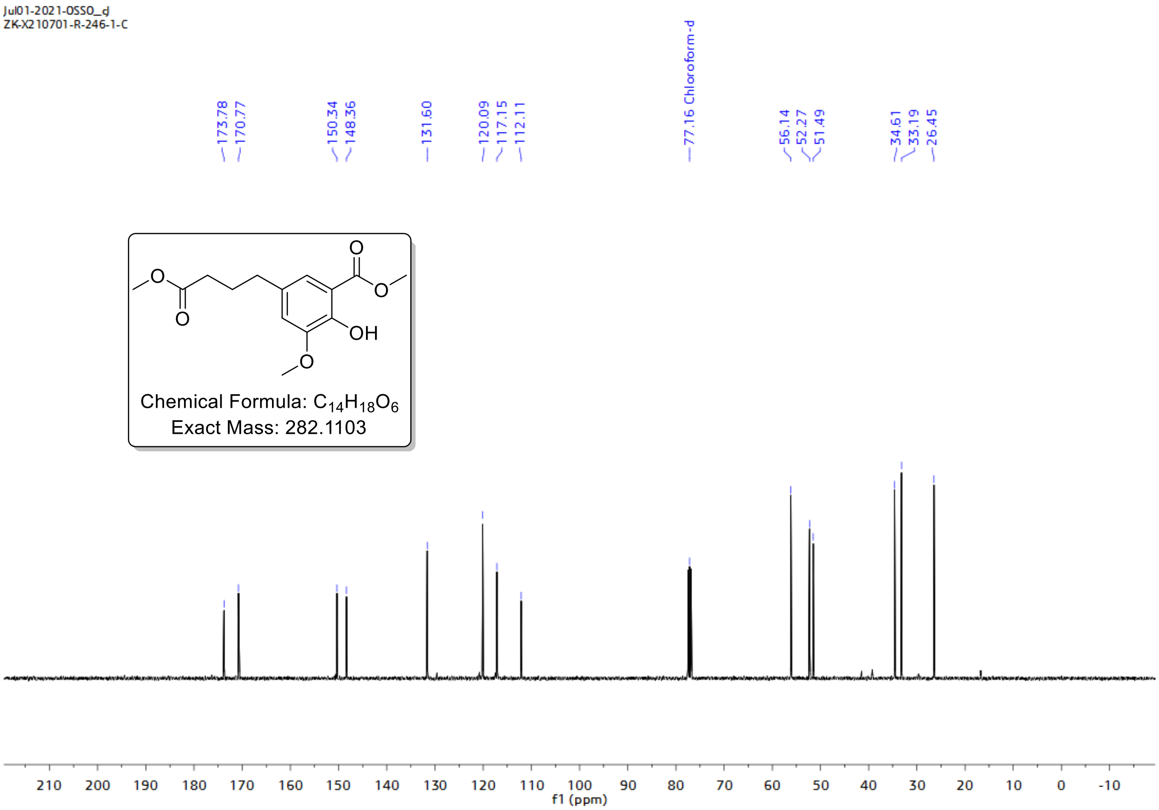

Supplementary Fig. 260 ^{13}C NMR (101 MHz, 20 °C) spectrum of product 102 in $CDCl_3$.

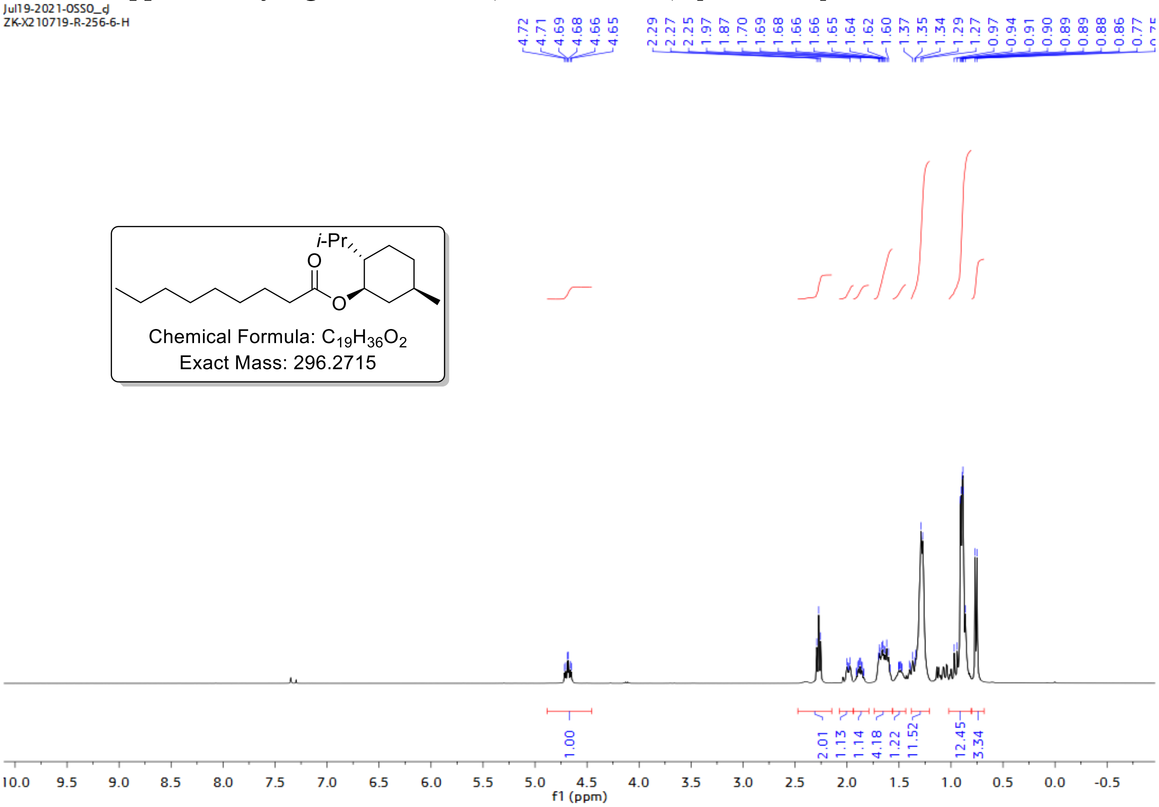

Supplementary Fig. 261 ^{1}H NMR (400 MHz, 20 °C) spectrum of product 103 in $CDCl_3$.

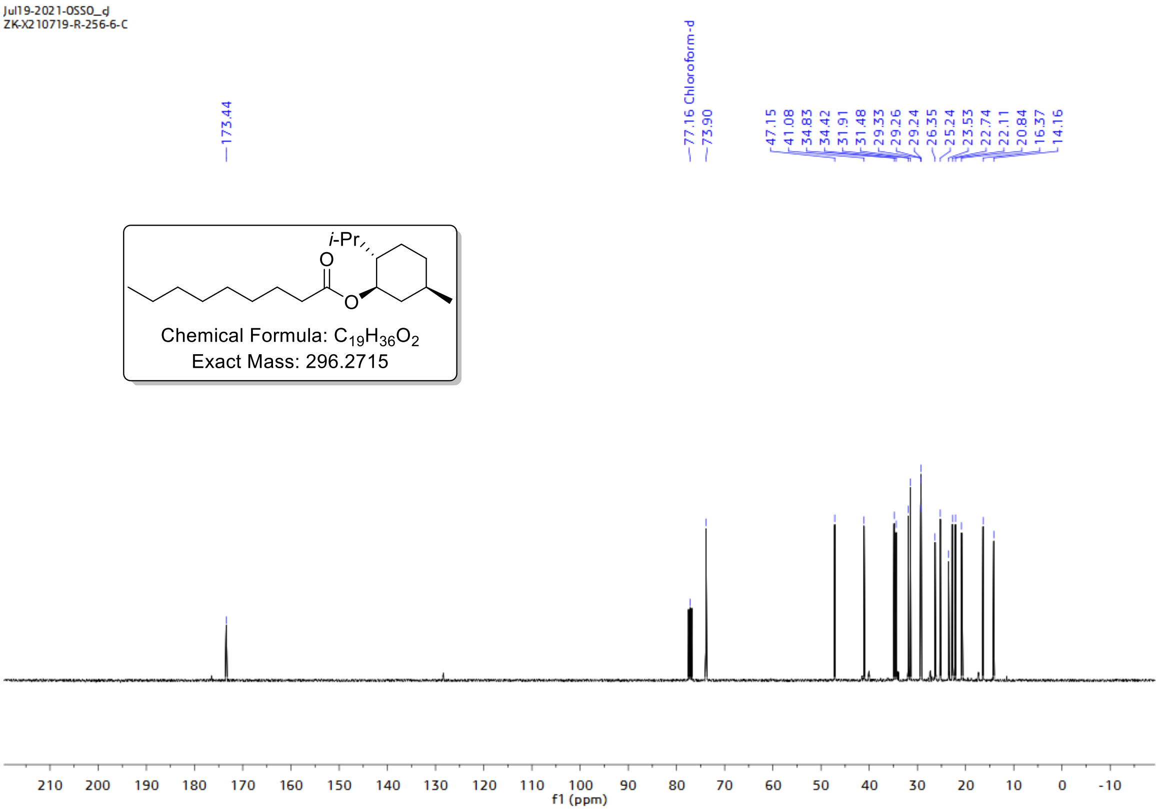

Supplementary Fig. 262 ^{13}C NMR (101 MHz, 20 ºC) spectrum of product 103 in $CDCl_3$.

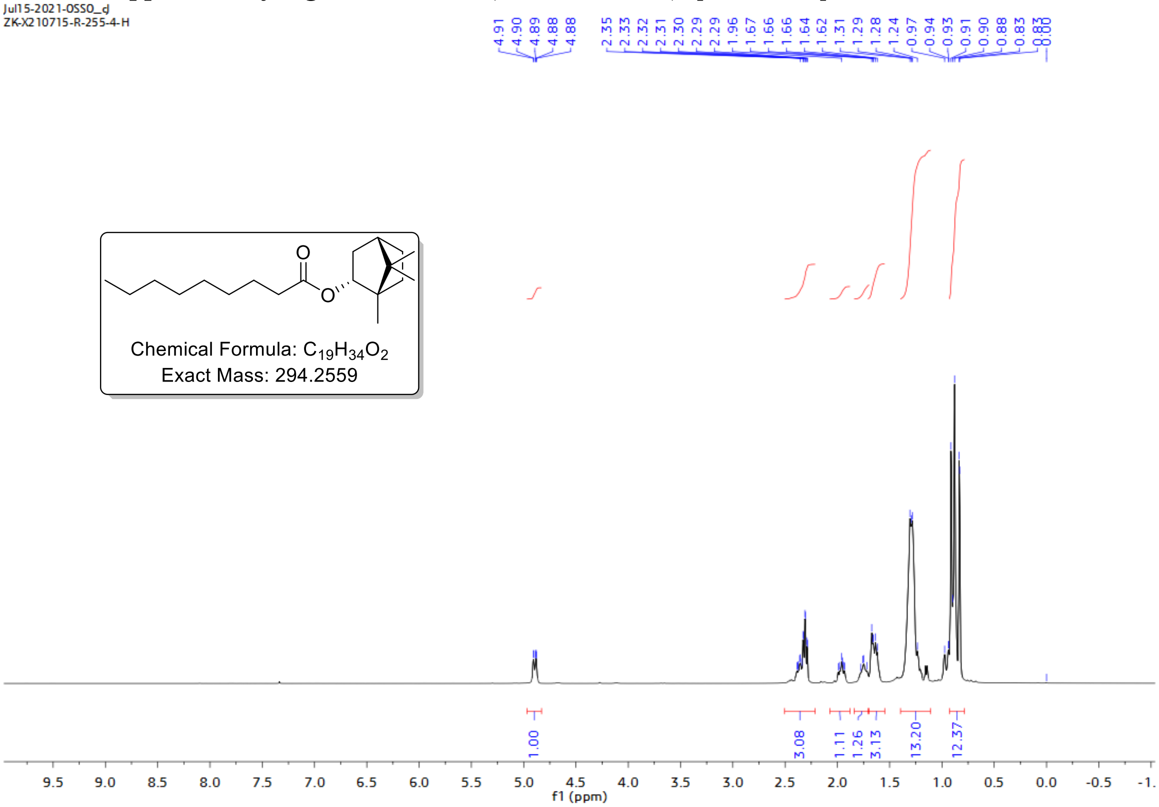

Supplementary Fig. 263 ^{1}H NMR (400 MHz, 20 ºC) spectrum of product 104 in $CDCl_3$.

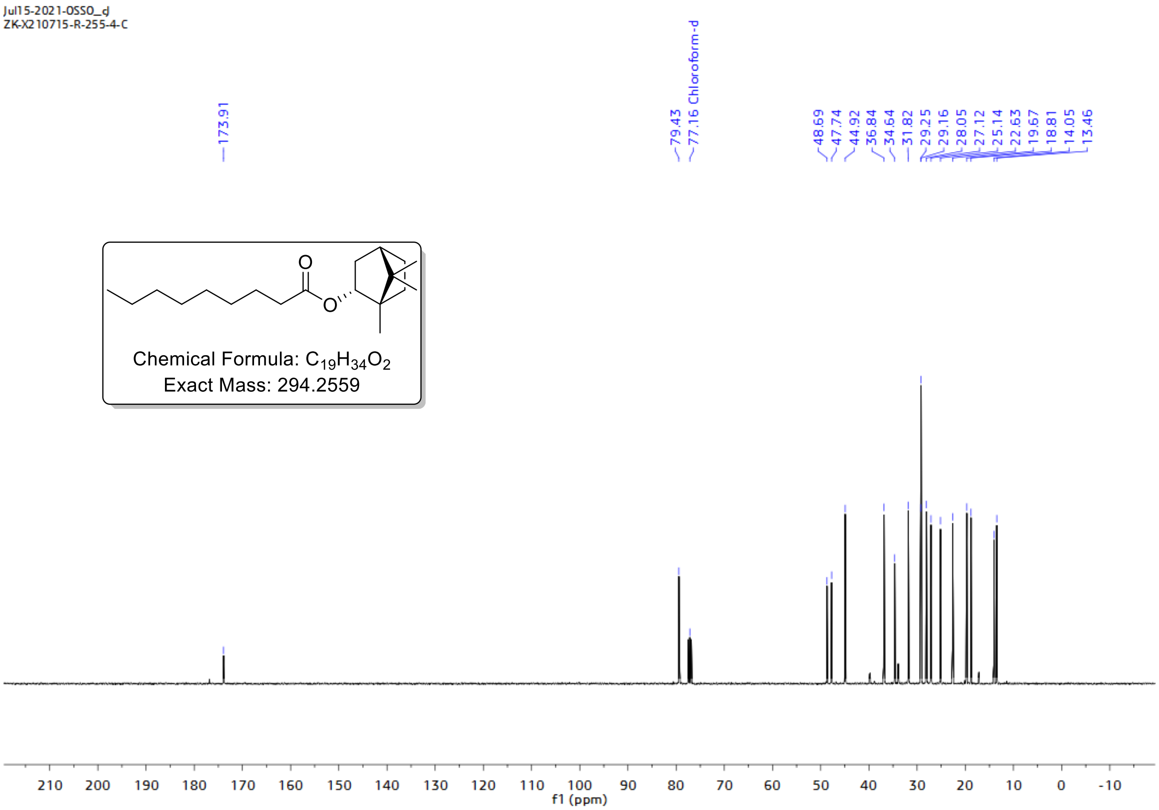

Supplementary Fig. 264 ^{13}C NMR (101 MHz, 20 °C) spectrum of product 104 in $CDCl_3$.

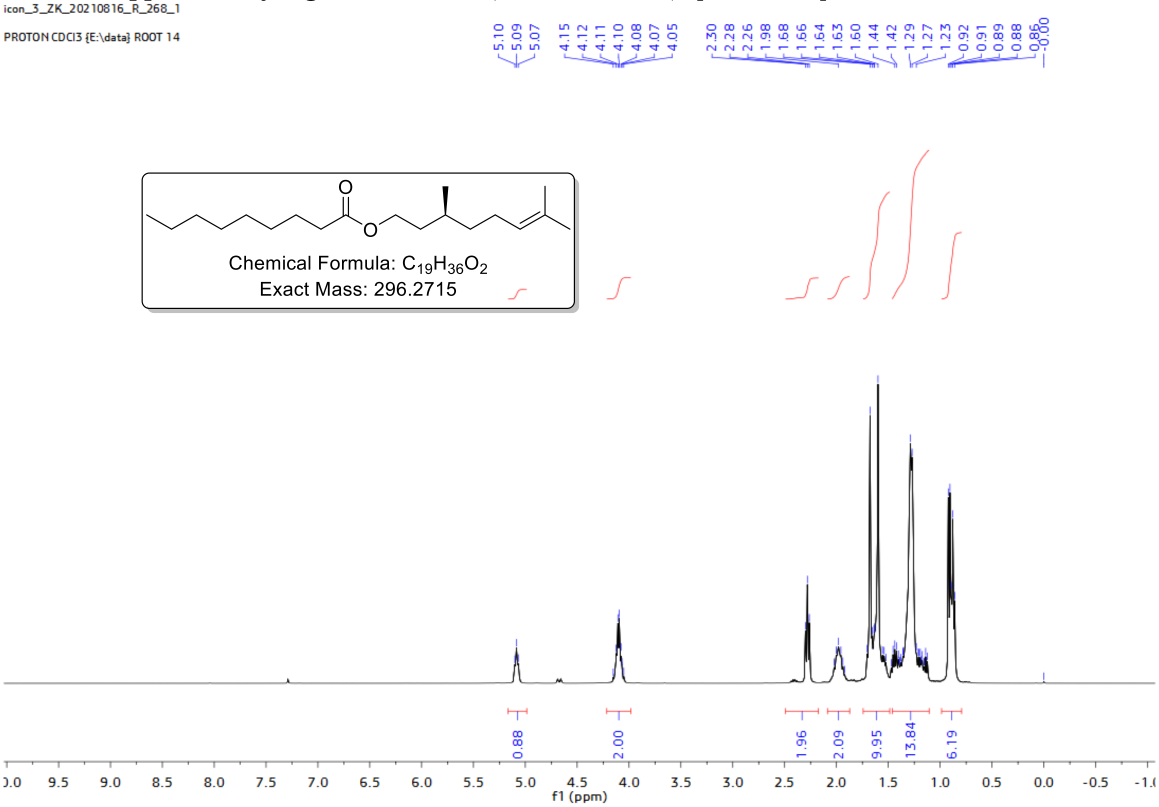

Supplementary Fig. 265 ^{1}H NMR (400 MHz, 20 °C) spectrum of product 105 in $CDCl_3$.

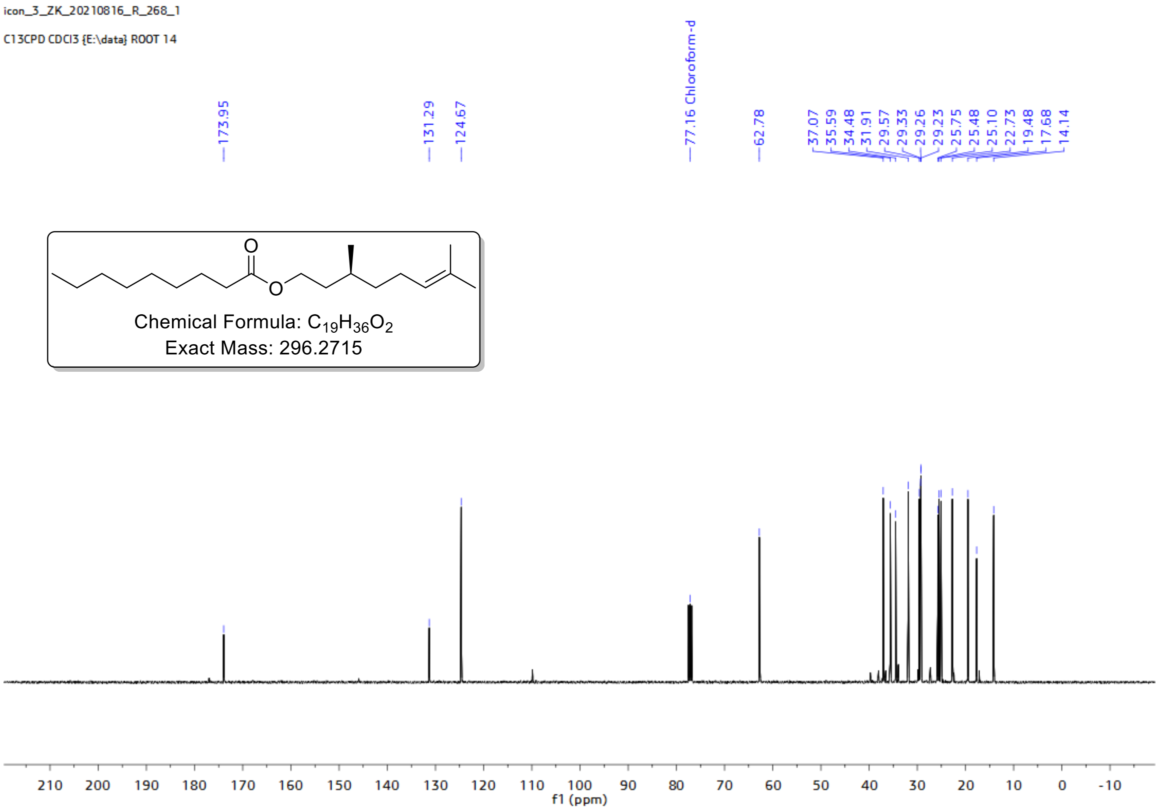

Supplementary Fig. 266 ^{13}C NMR (101 MHz, 20 °C) spectrum of product 105 in $CDCl_3$.

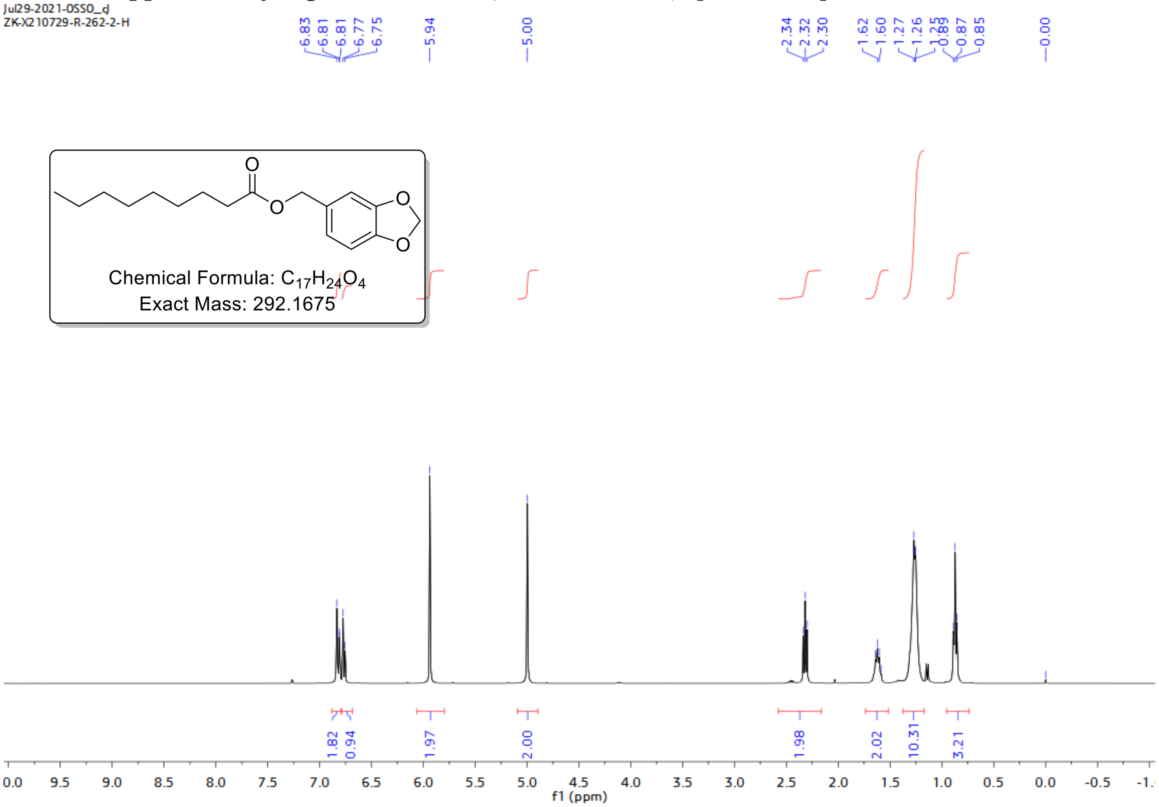

Supplementary Fig. 267 ^{1}H NMR (400 MHz, 20 °C) spectrum of product 106 in $CDCl_3$.

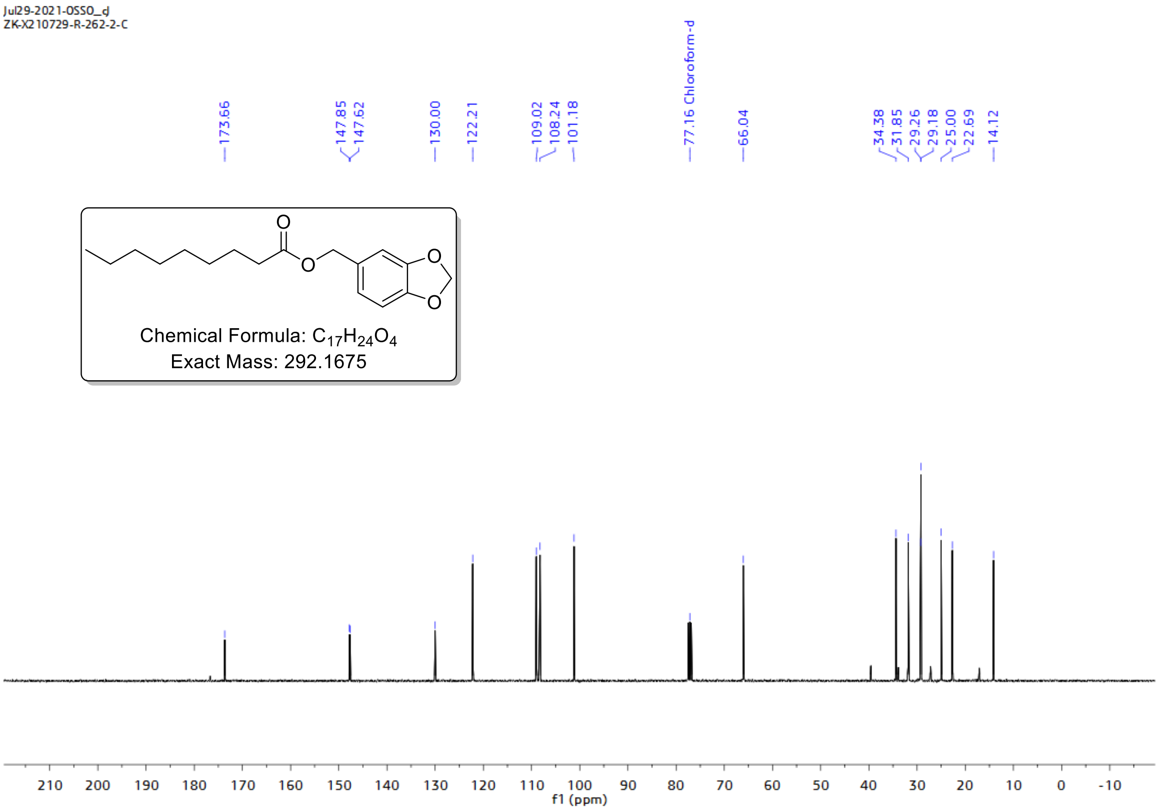

Supplementary Fig. 268 ^{13}C NMR (101 MHz, 20 °C) spectrum of product 106 in $CDCl_3$.

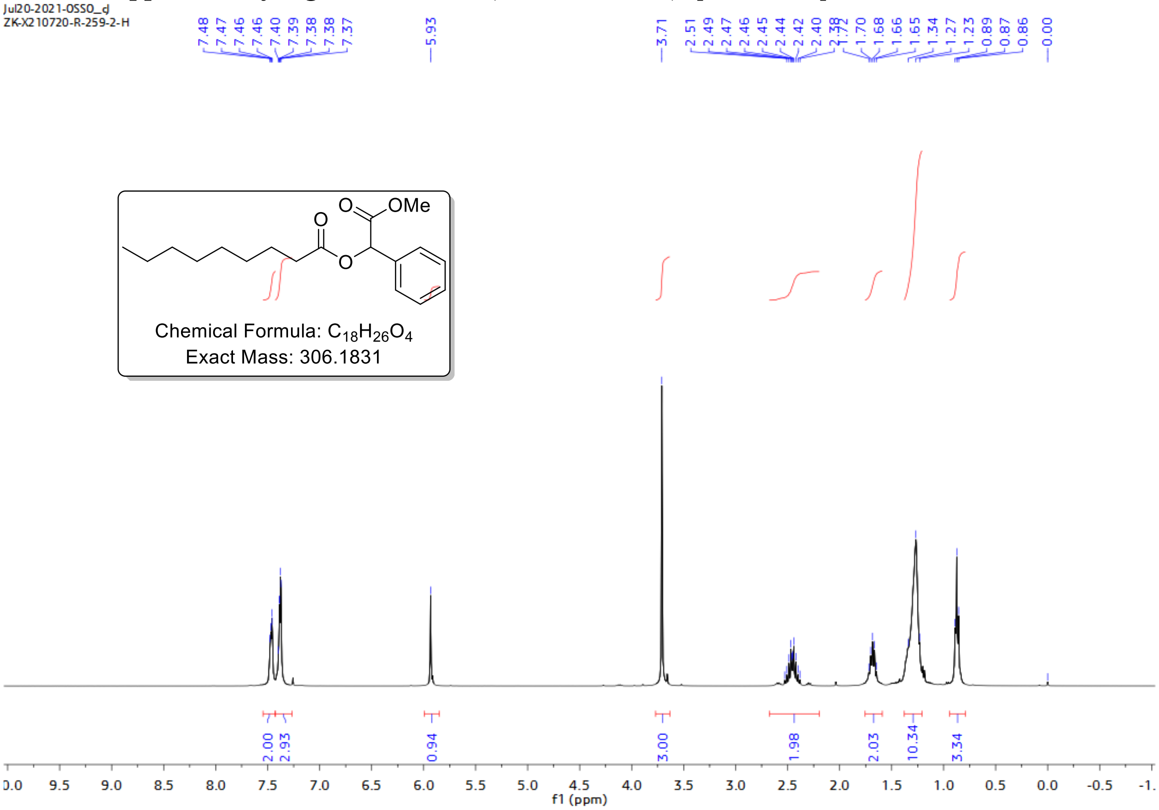

Supplementary Fig. 269 ^{1}H NMR (400 MHz, 20 °C) spectrum of product 107 in $CDCl_3$.

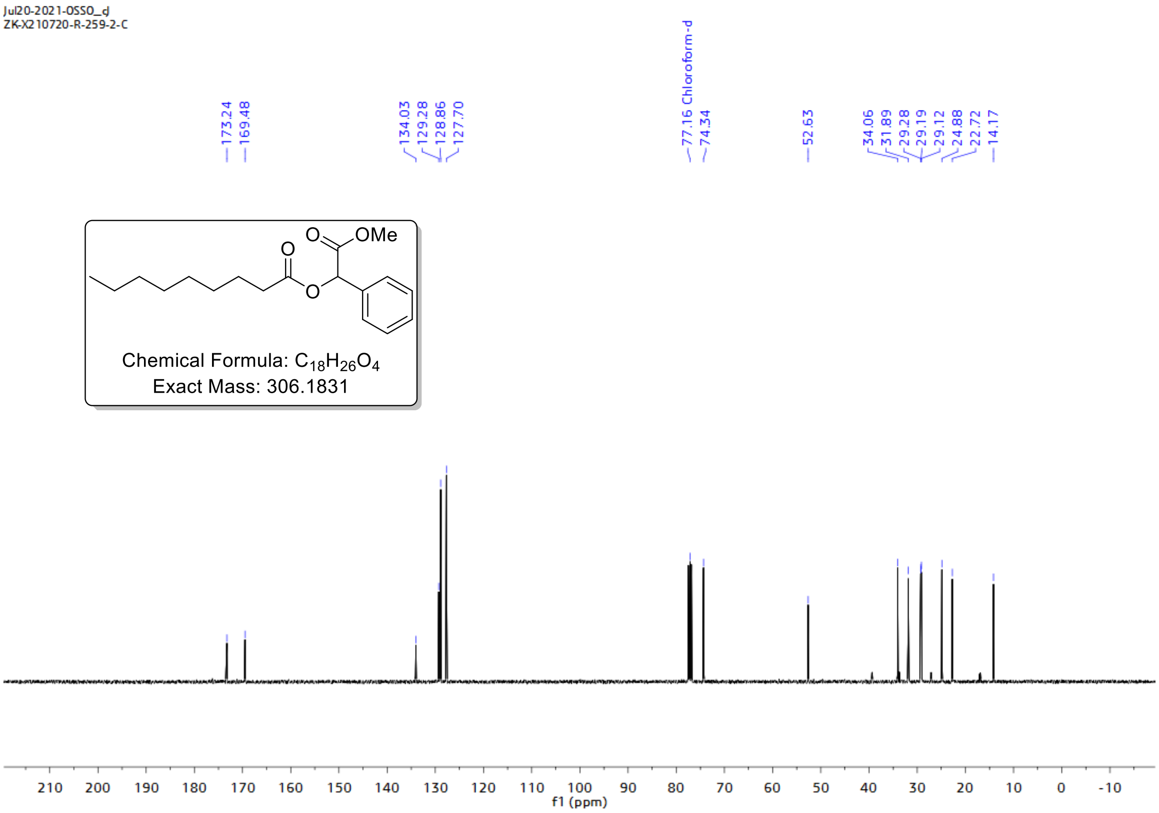

Supplementary Fig. 270 ^{13}C NMR (101 MHz, 20 °C) spectrum of product 107 in $CDCl_3$.

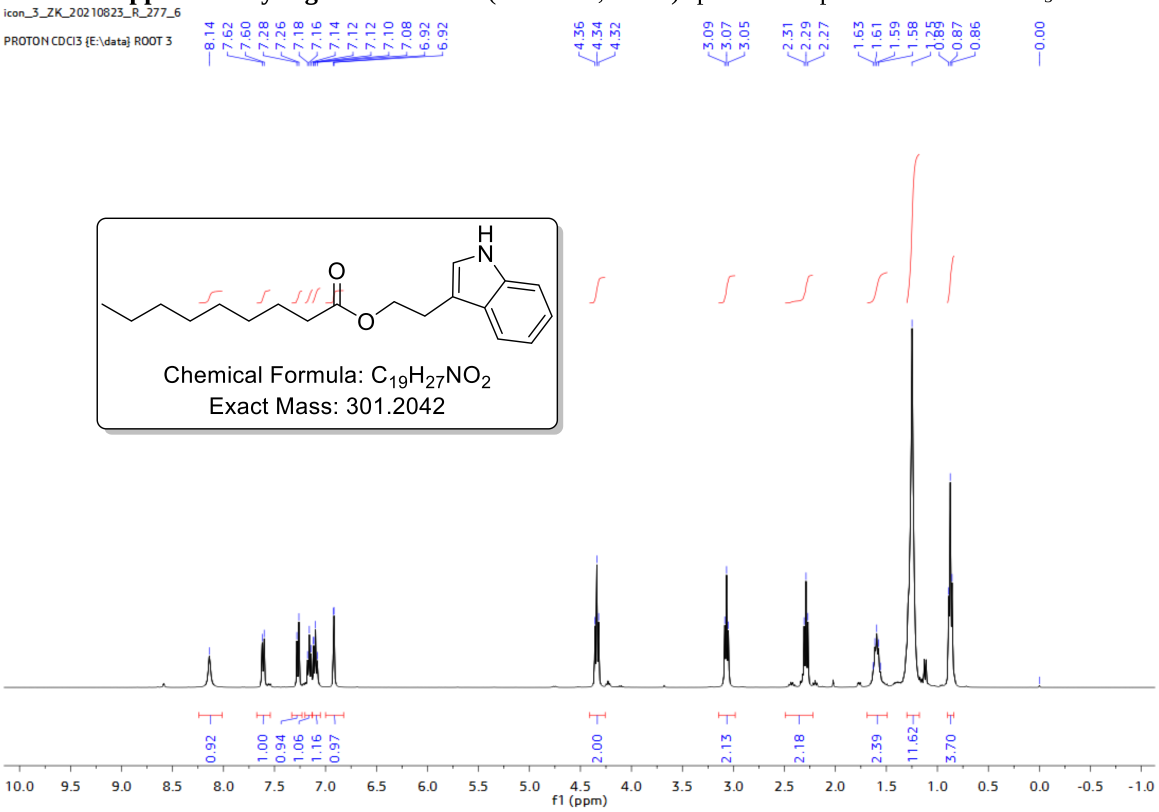

Supplementary Fig. 271 ^{1}H NMR (400 MHz, 20 °C) spectrum of product 108 in $CDCl_3$.

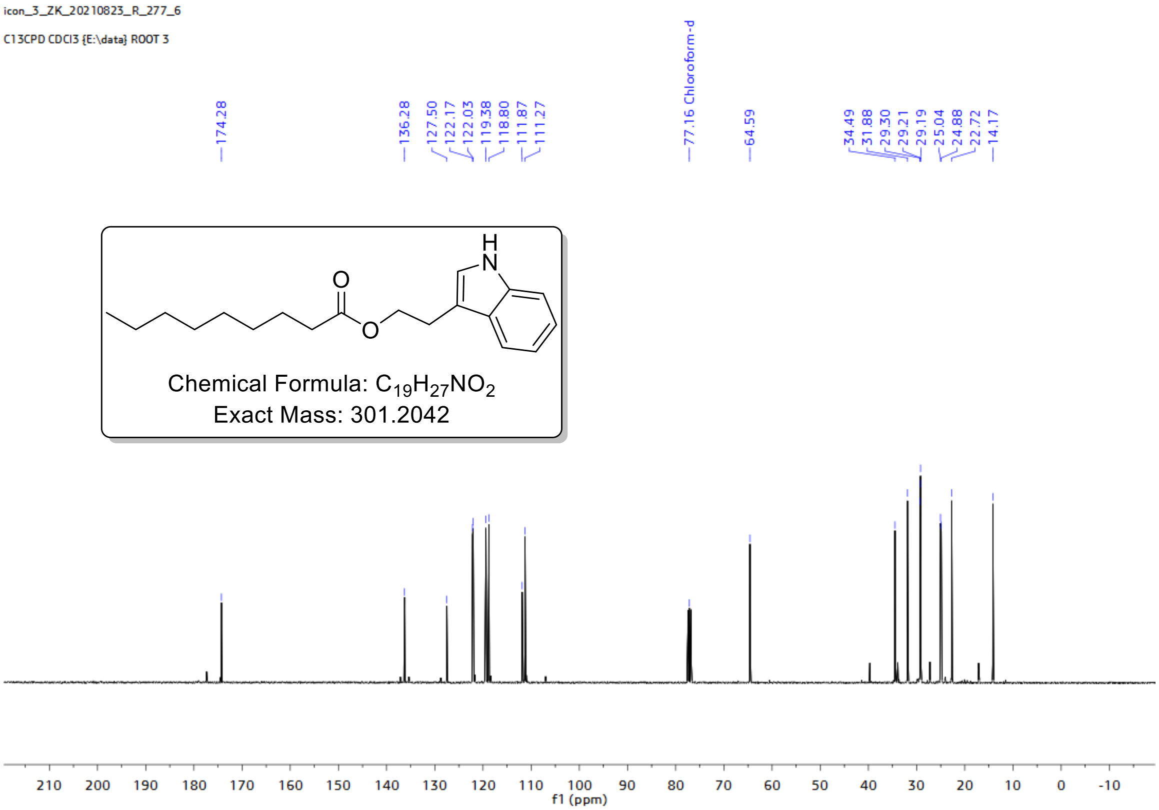

Supplementary Fig. 272 ^{13}C NMR (101 MHz, 20 °C) spectrum of product 108 in $CDCl_3$.

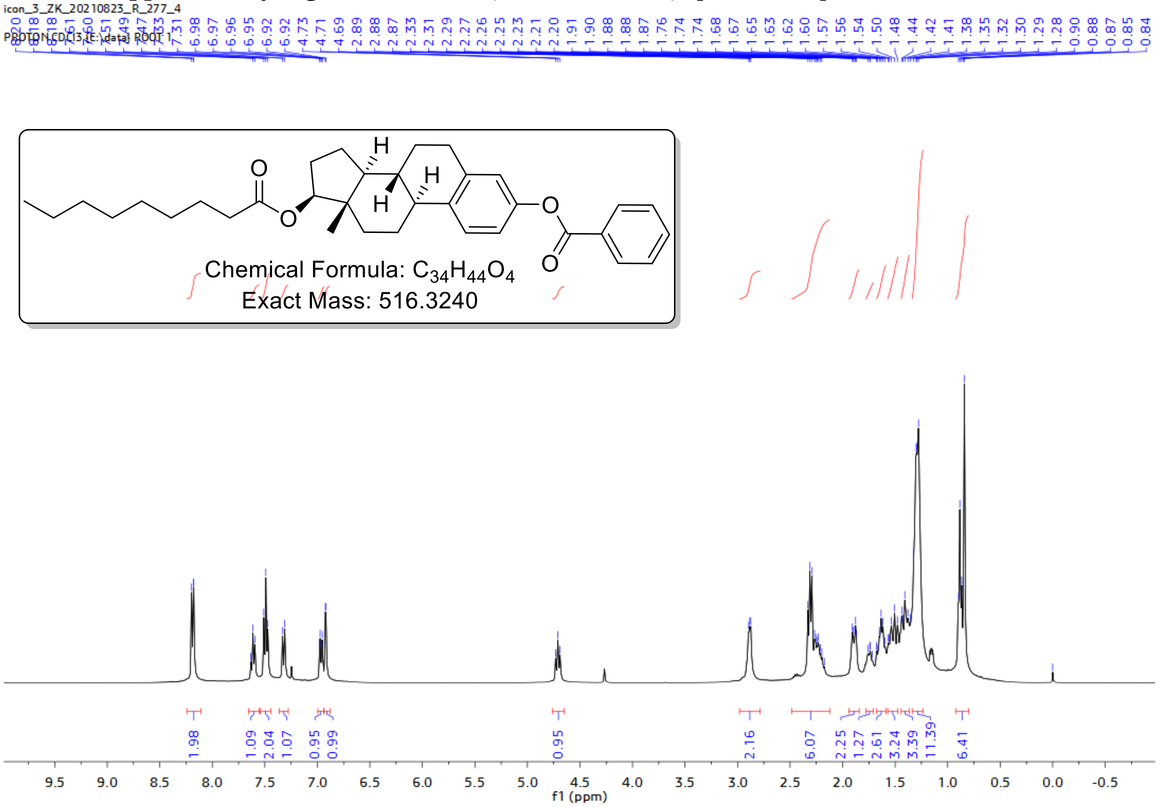

Supplementary Fig. 273 ^{1}H NMR (400 MHz, 20 °C) spectrum of product 109 in $CDCl_3$.

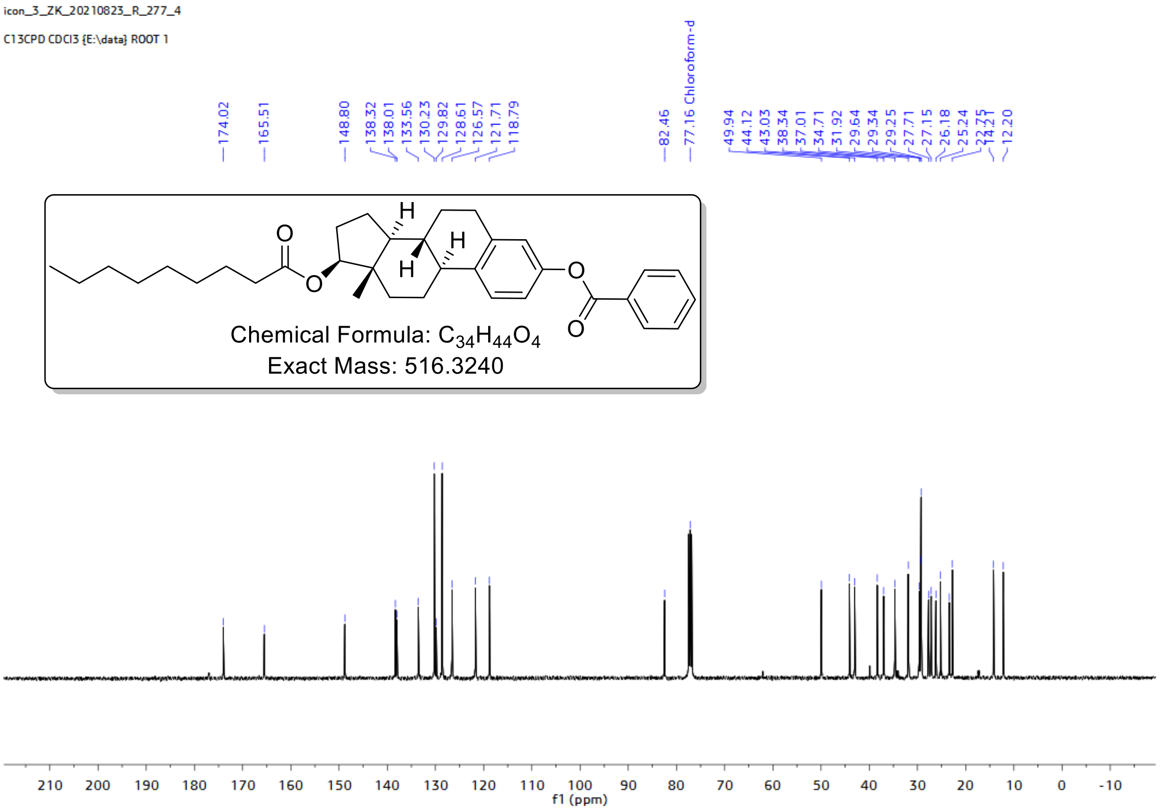

Supplementary Fig. 274 ^{13}C NMR (101 MHz, 20 °C) spectrum of product 109 in $CDCl_3$.

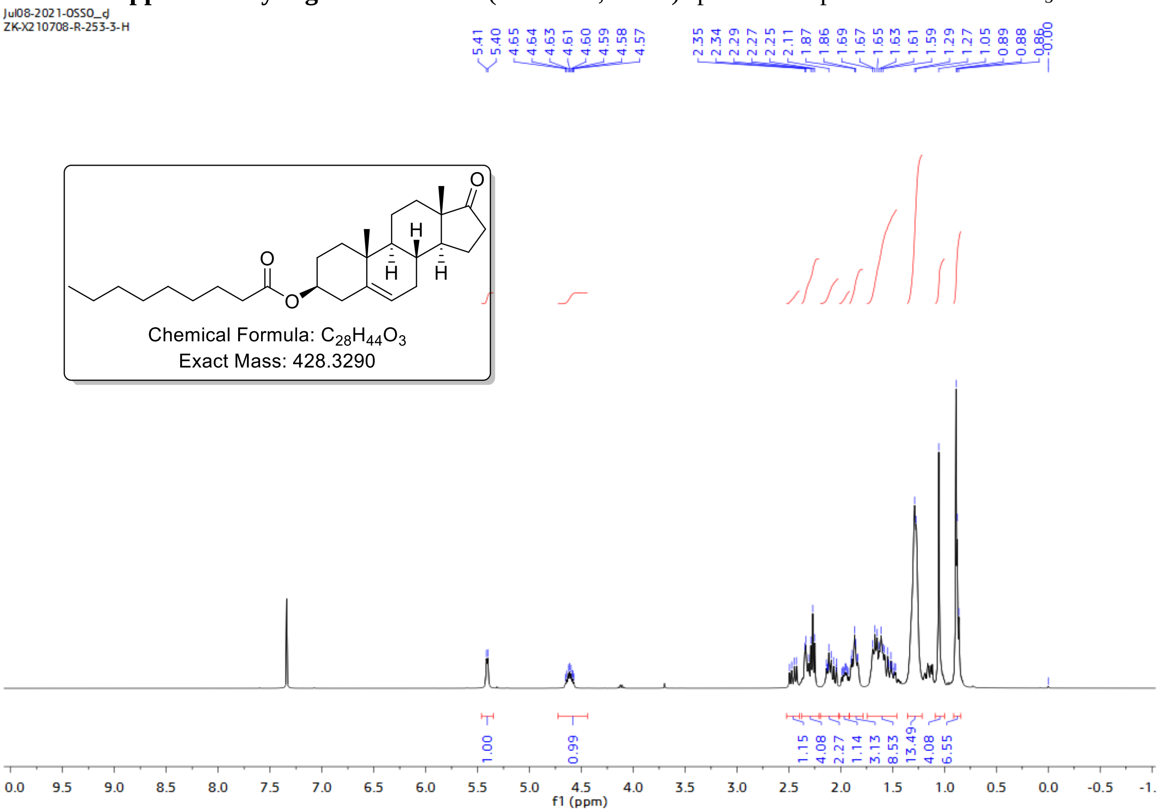

Supplementary Fig. 275 ^{1}H NMR (400 MHz, 20 °C) spectrum of product 110 in $CDCl_3$.

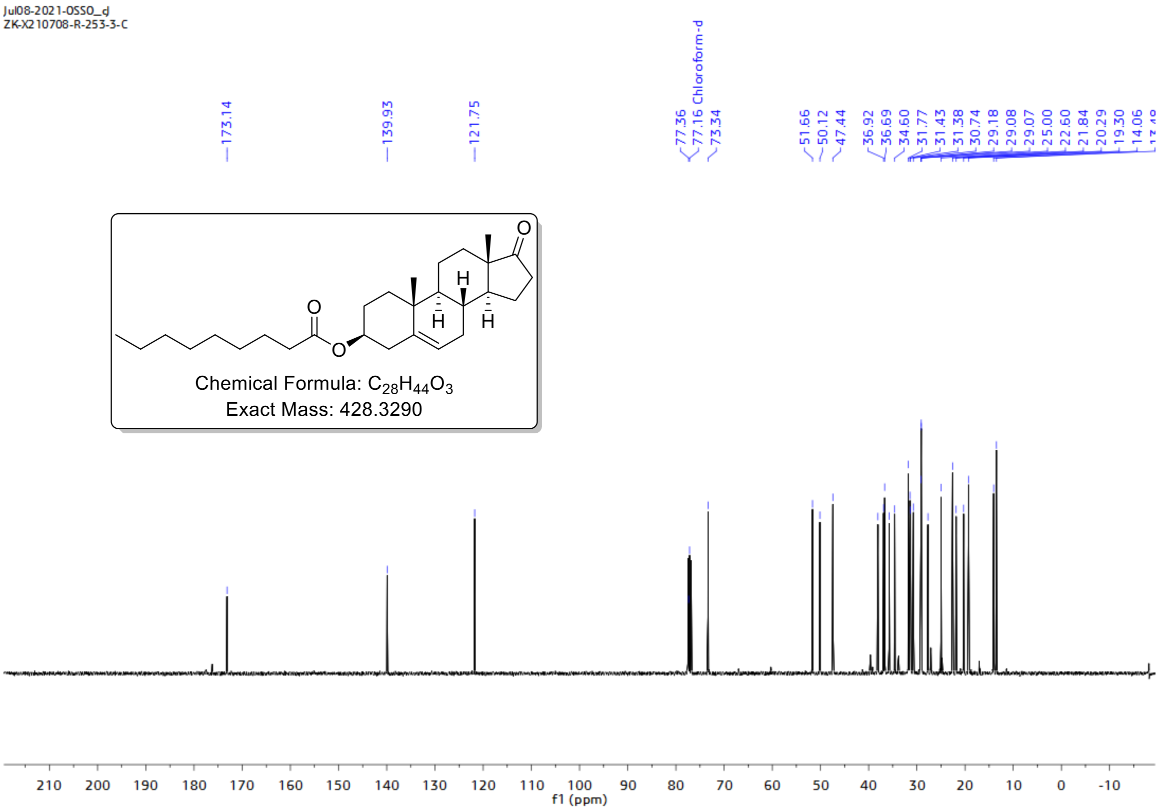

Supplementary Fig. 276 ^{1}H NMR (400 MHz, 20 °C) spectrum of product 110 in $CDCl_3$.

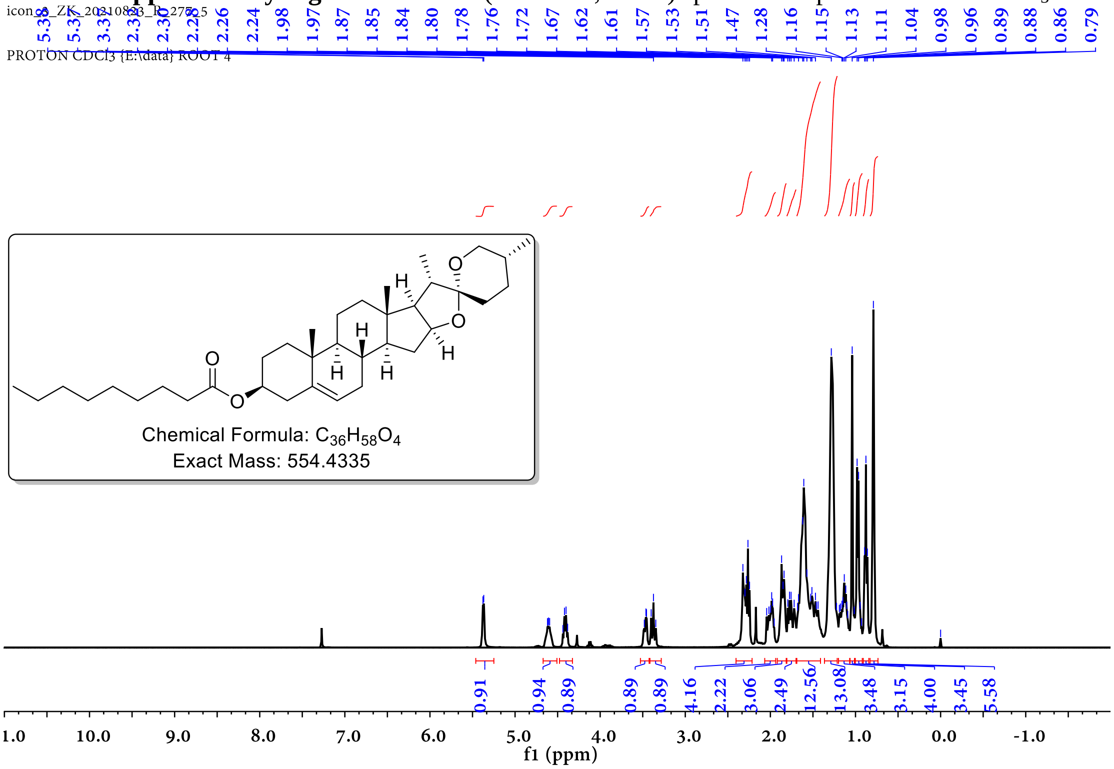

Supplementary Fig. 277 ^{1}H NMR (400 MHz, 20 °C) spectrum of product 111 in $CDCl_3$.

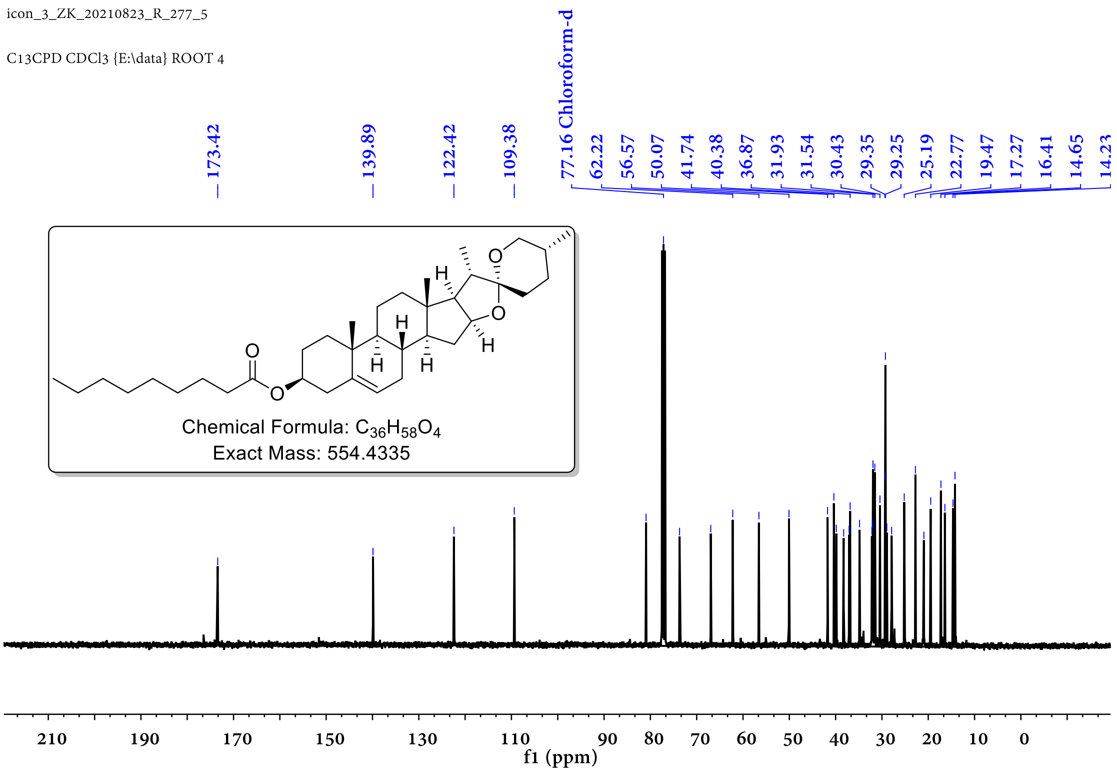

Supplementary Fig. 278 ^{13}C NMR (101 MHz, 20 °C) spectrum of product 111 in $CDCl_3$.

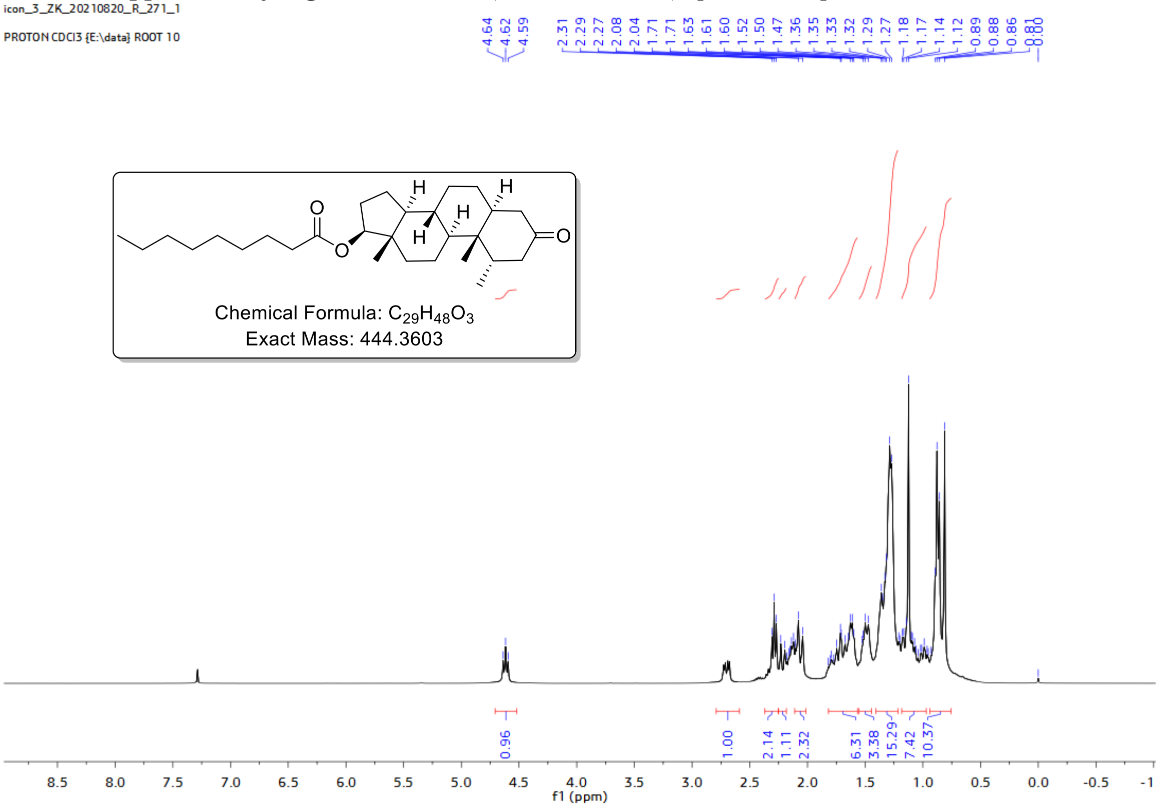

Supplementary Fig. 279 ^{1}H NMR (400 MHz, 20 °C) spectrum of product 112 in $CDCl_3$.

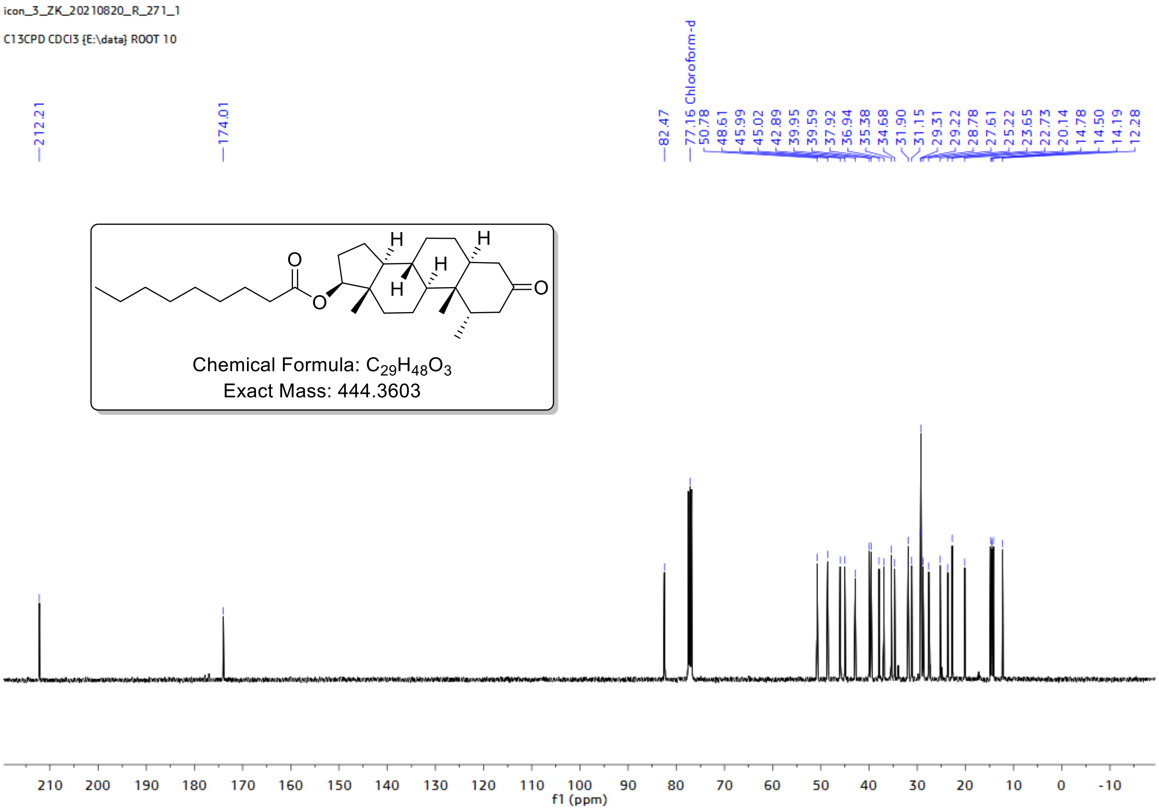

Supplementary Fig. 280 ^{13}C NMR (101 MHz, 20 °C) spectrum of product 112 in $CDCl_3$.

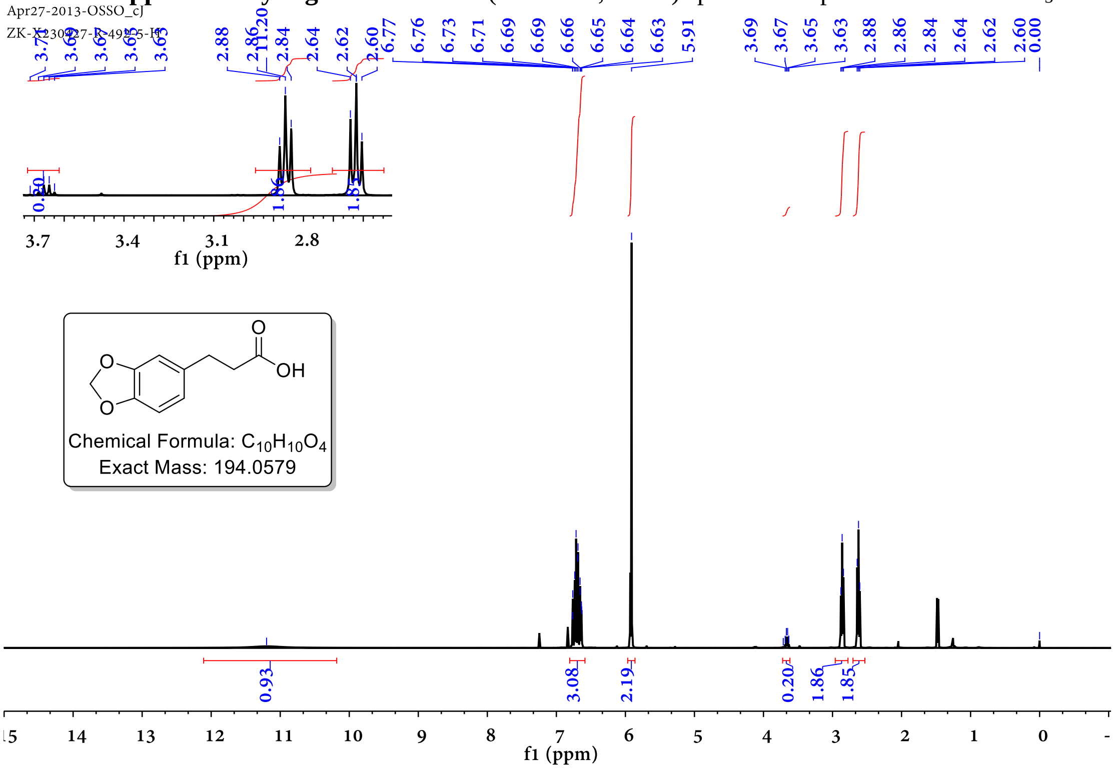

Supplementary Fig. 281 ^{1}H NMR (400 MHz, 20 °C) spectrum of product 113 in $CDCl_3$.

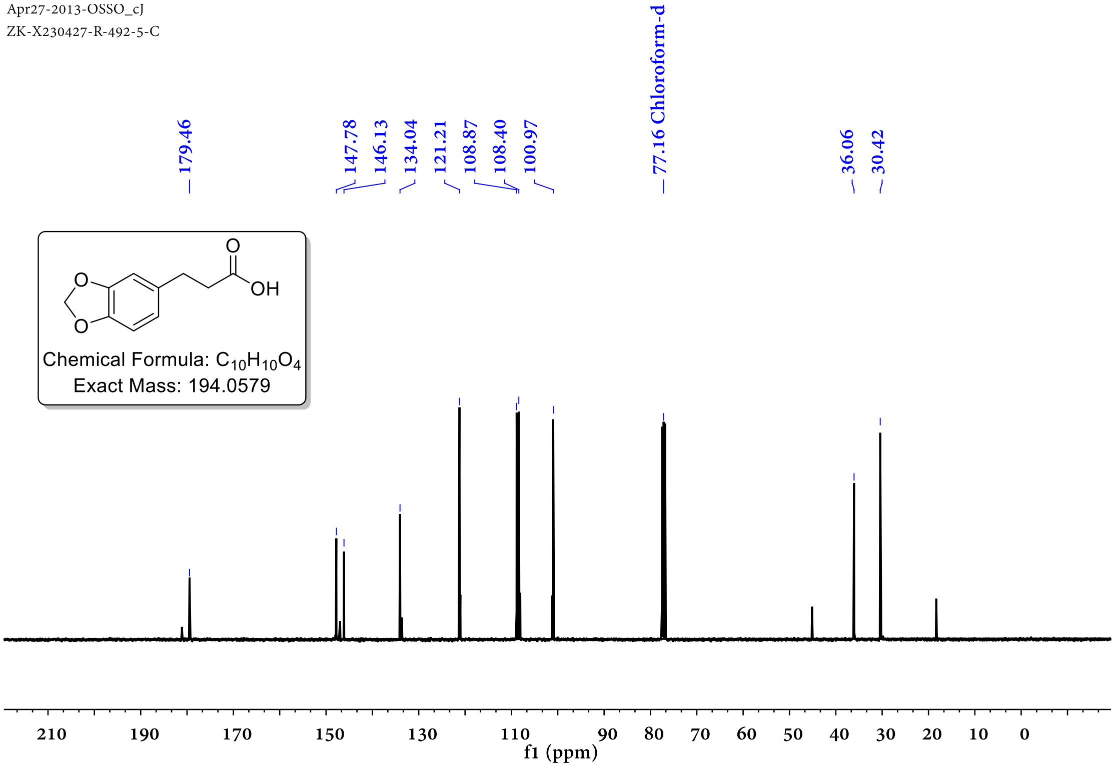

Supplementary Fig. 282 ^{13}C NMR (101 MHz, 20 °C) spectrum of product 113 in $CDCl_3$.

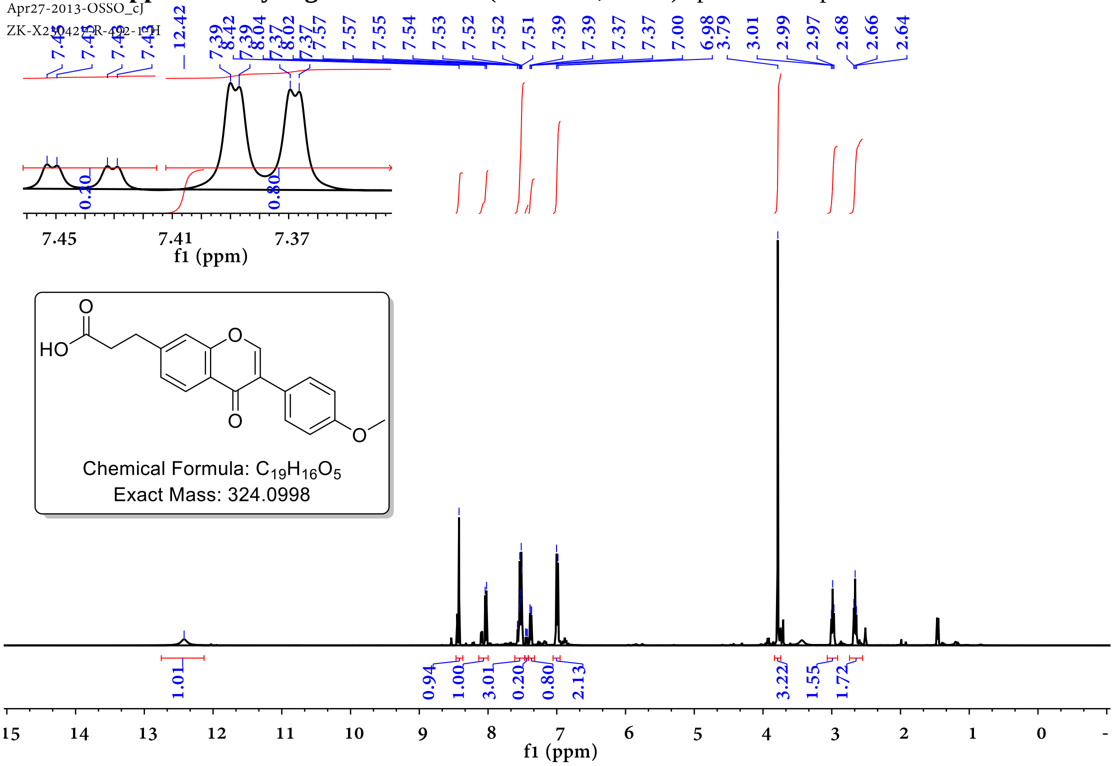

Supplementary Fig. 283 ^{1}H NMR (400 MHz, 20 °C) spectrum of product 114 in DMSO-d6.

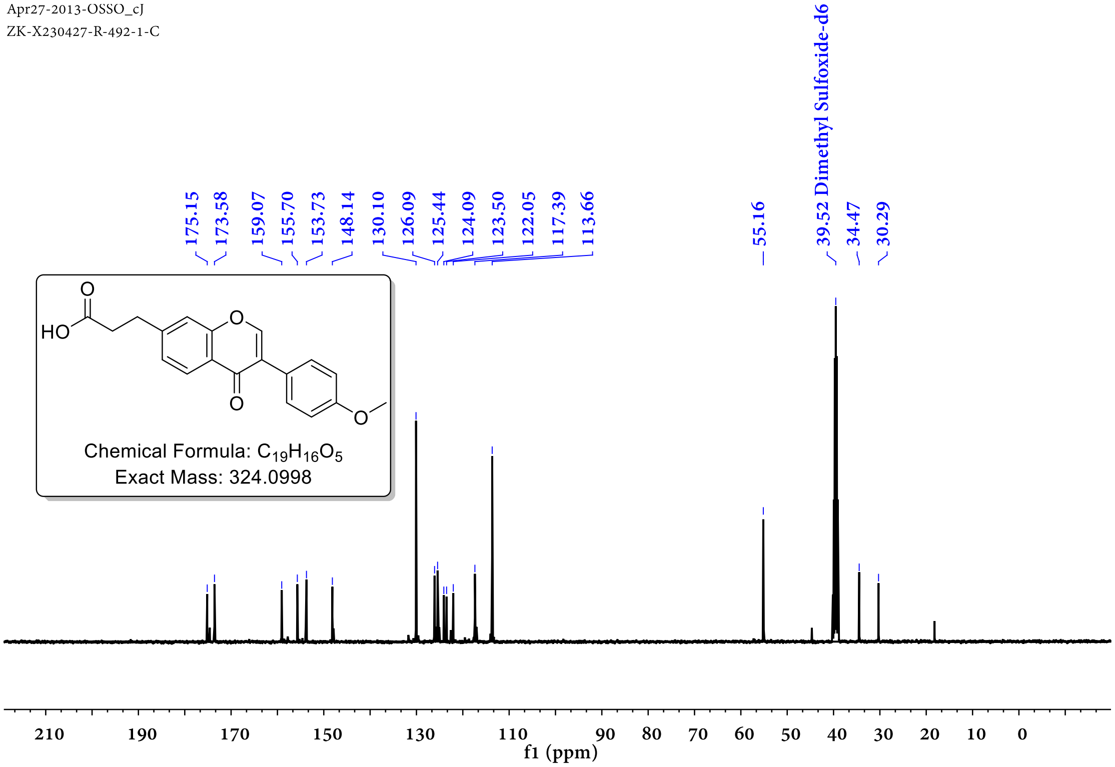

Supplementary Fig. 284 ^{13}C NMR (101 MHz, 20 °C) spectrum of product 114 in DMSO-d6.

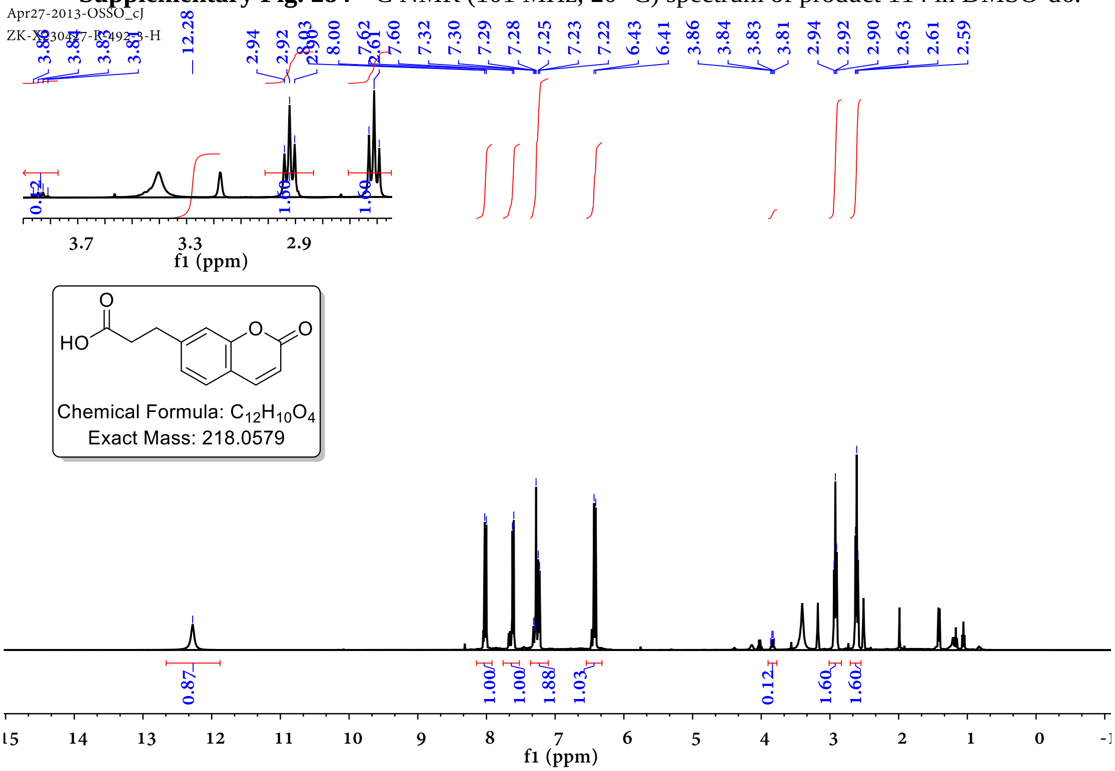

Supplementary Fig. 285 ^{1}H NMR (400 MHz, 20 °C) spectrum of product 115 in DMSO-d6.

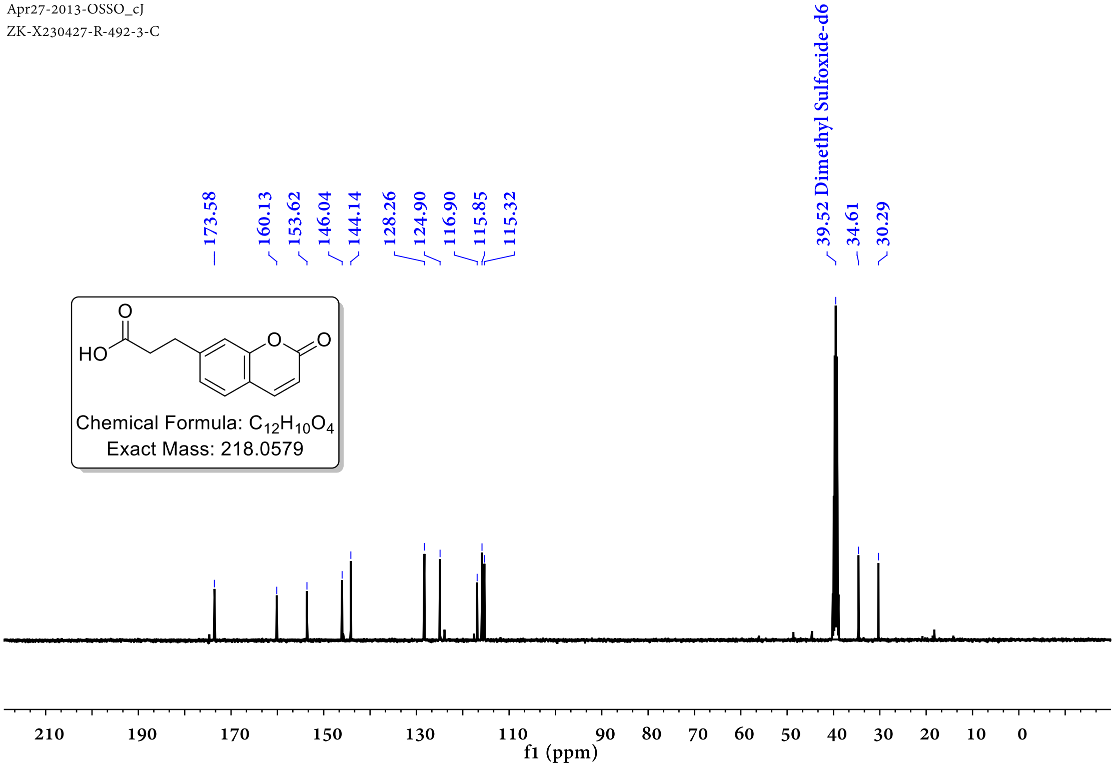

Supplementary Fig. 286 ^{13}C NMR (101 MHz, 20 °C) spectrum of product 115 in DMSO-d6.

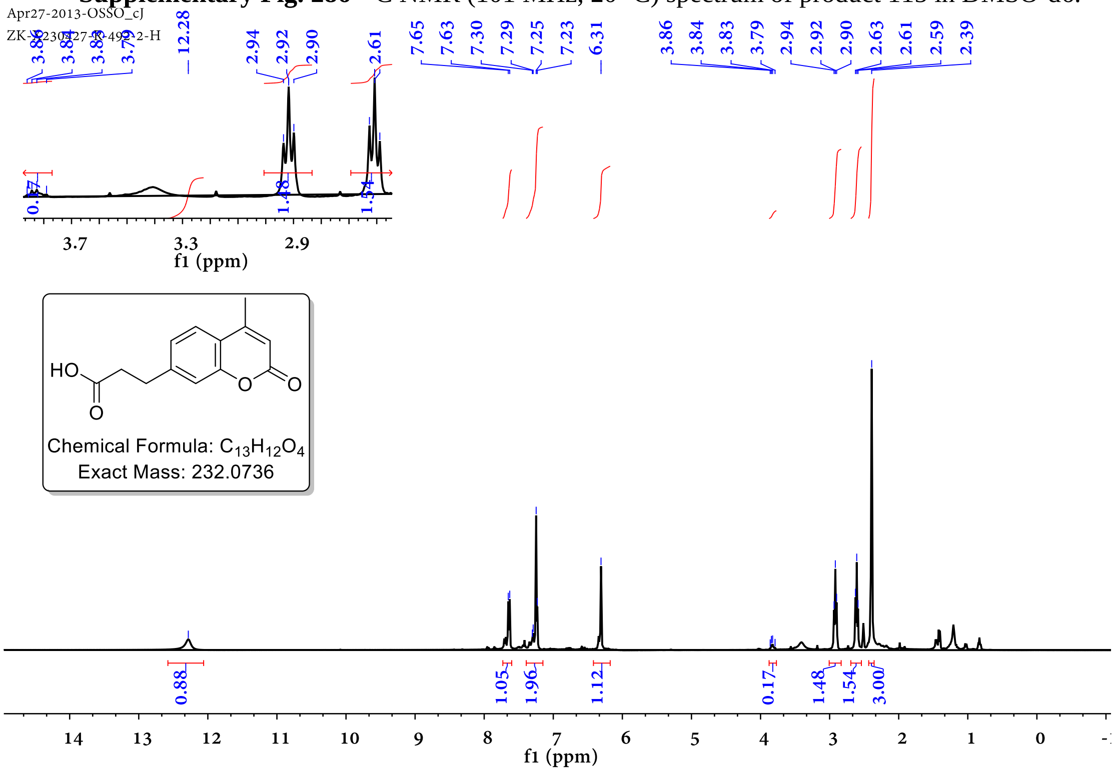

Supplementary Fig. 287 ^{1}H NMR (400 MHz, 20 °C) spectrum of product 116 in DMSO-d6.

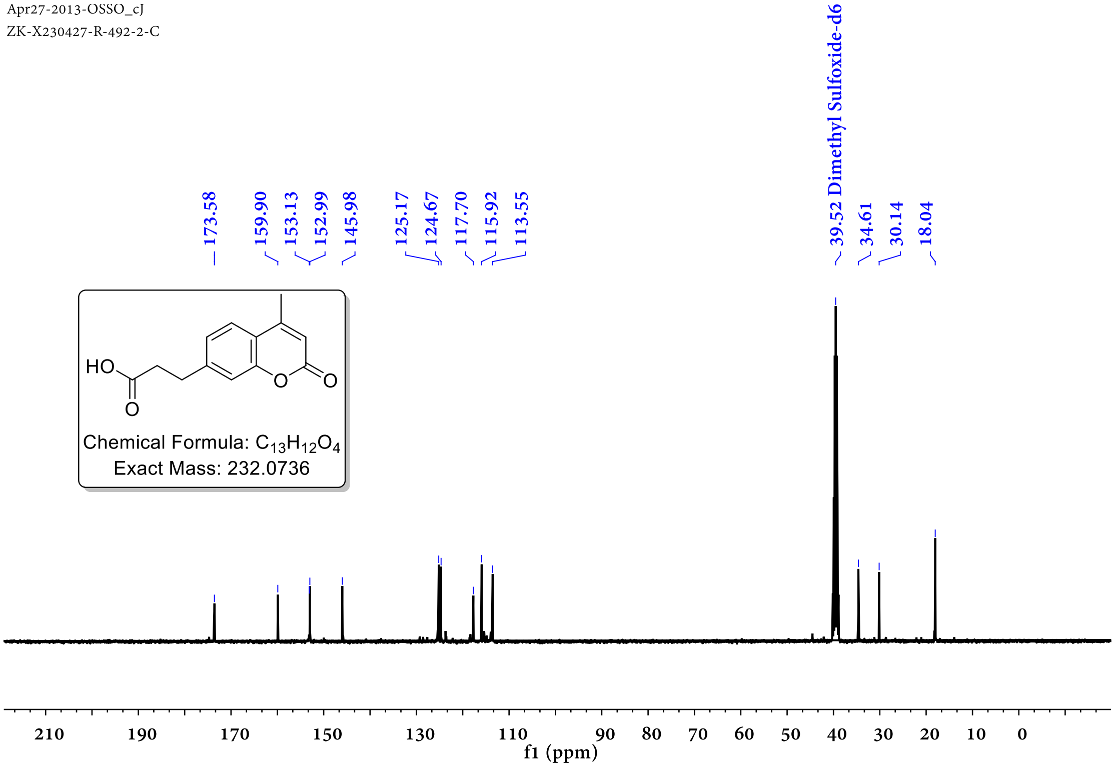

Supplementary Fig. 288 ^{13}C NMR (101 MHz, 20 °C) spectrum of product 116 in DMSO-d6.

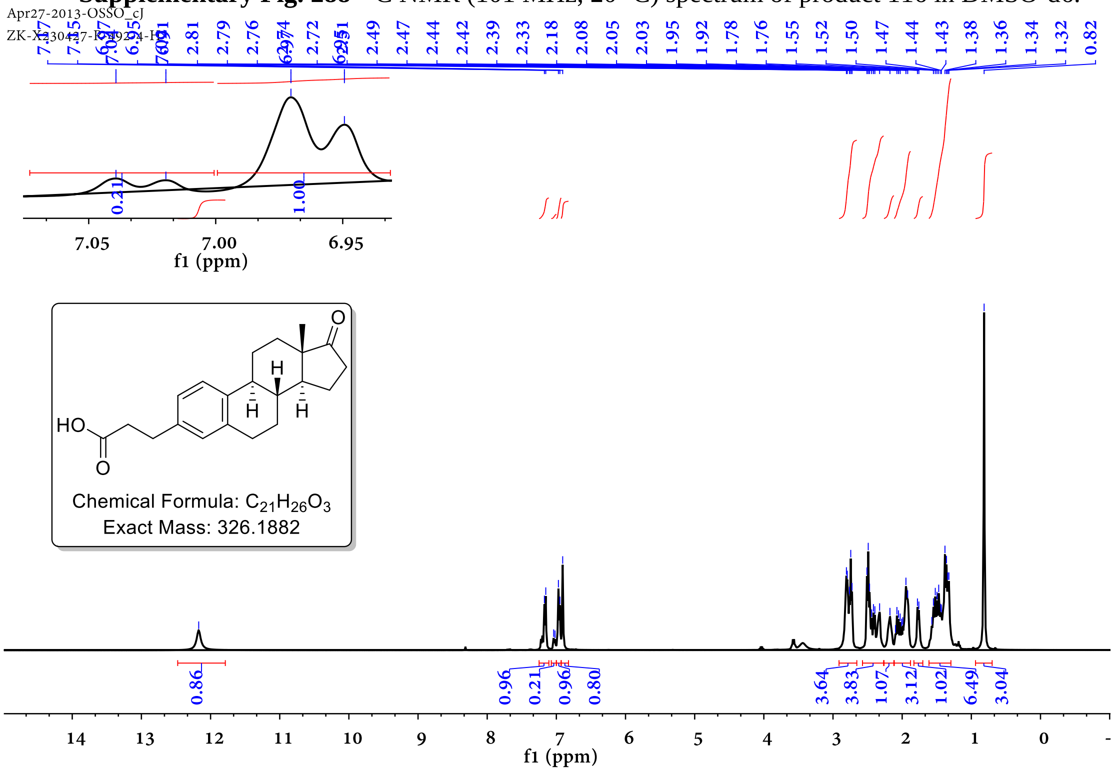

Supplementary Fig. 289 ^{1}H NMR (400 MHz, 20 °C) spectrum of product 117 in DMSO-d6.

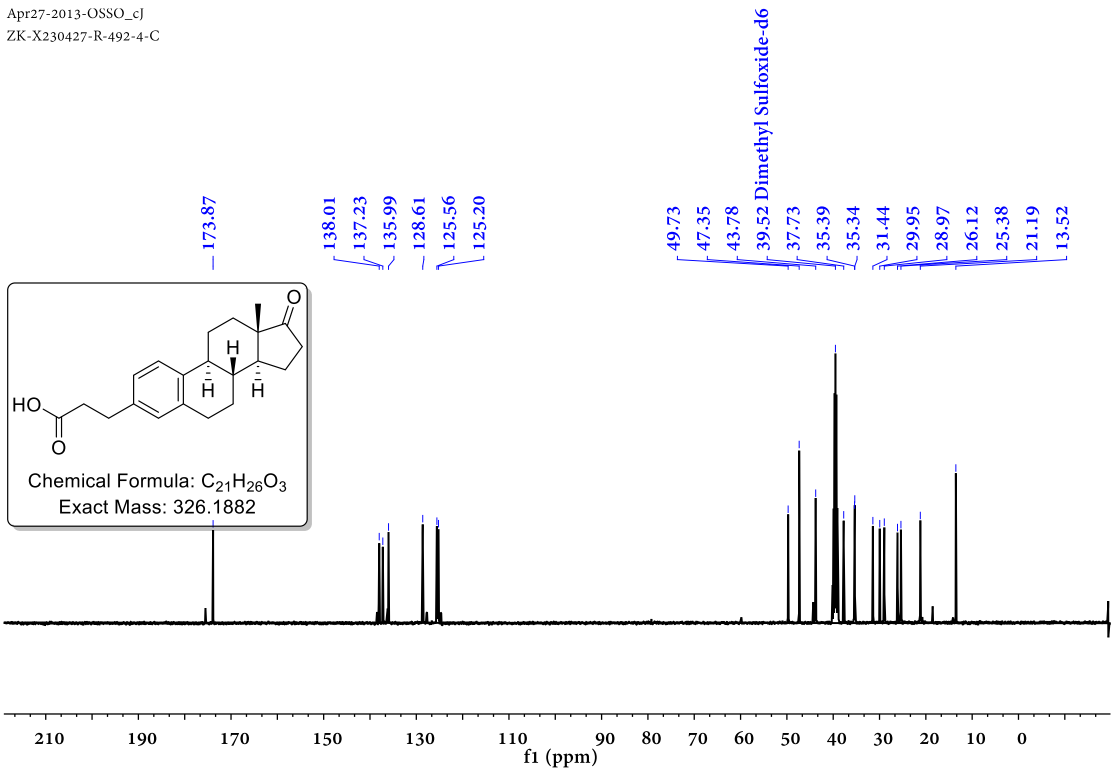

Supplementary Fig. 290 ^{13}C NMR (101 MHz, 20 °C) spectrum of product 117 in DMSO-d6.

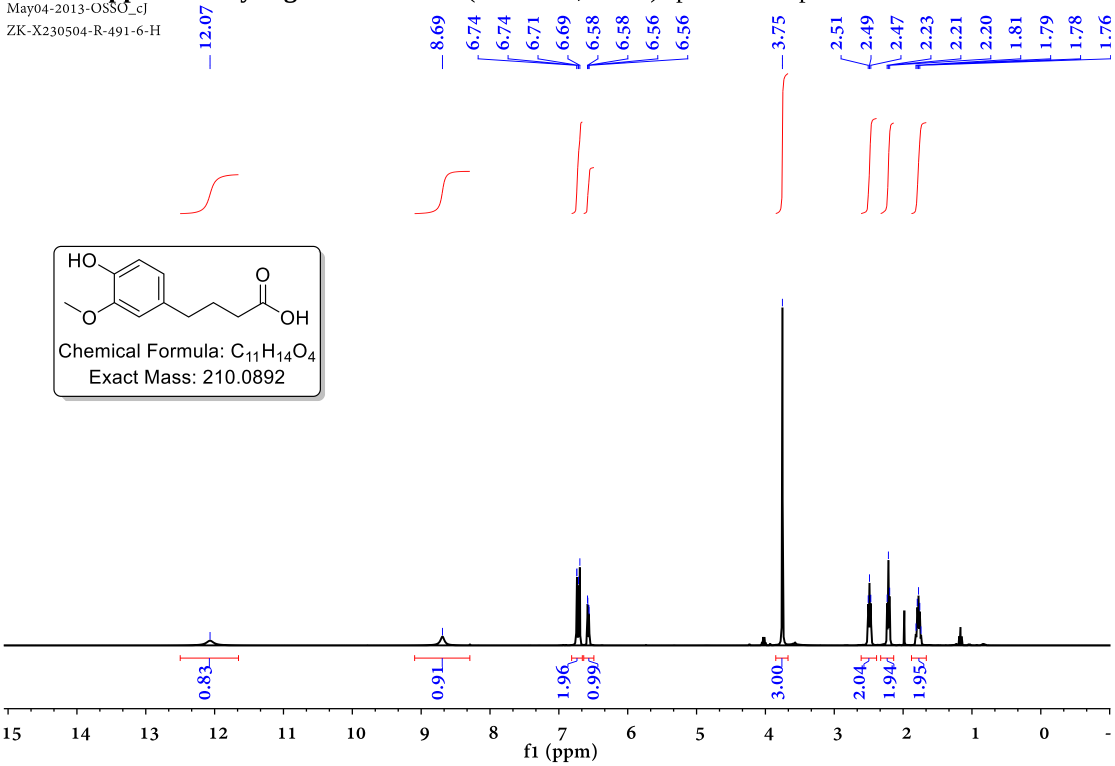

Supplementary Fig. 291 ^{1}H NMR (400 MHz, 20 °C) spectrum of product 118 in DMSO-d6.

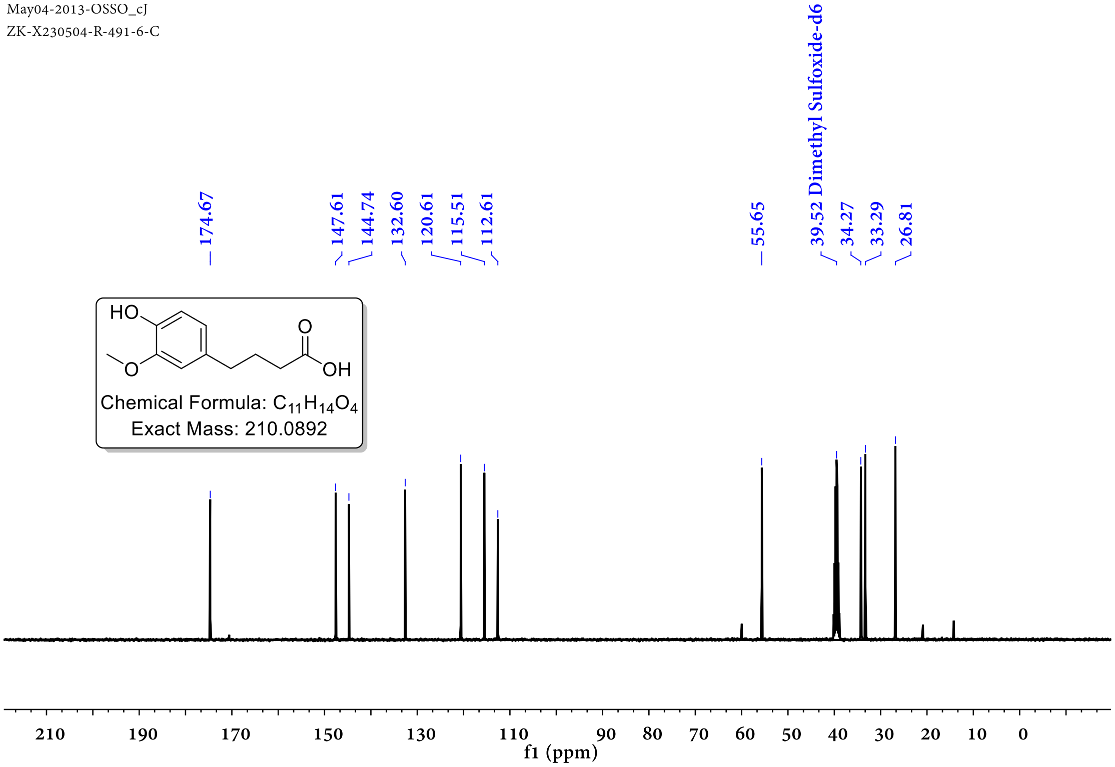

Supplementary Fig. 292 ^{13}C NMR (101 MHz, 20 °C) spectrum of product 118 in DMSO-d6.

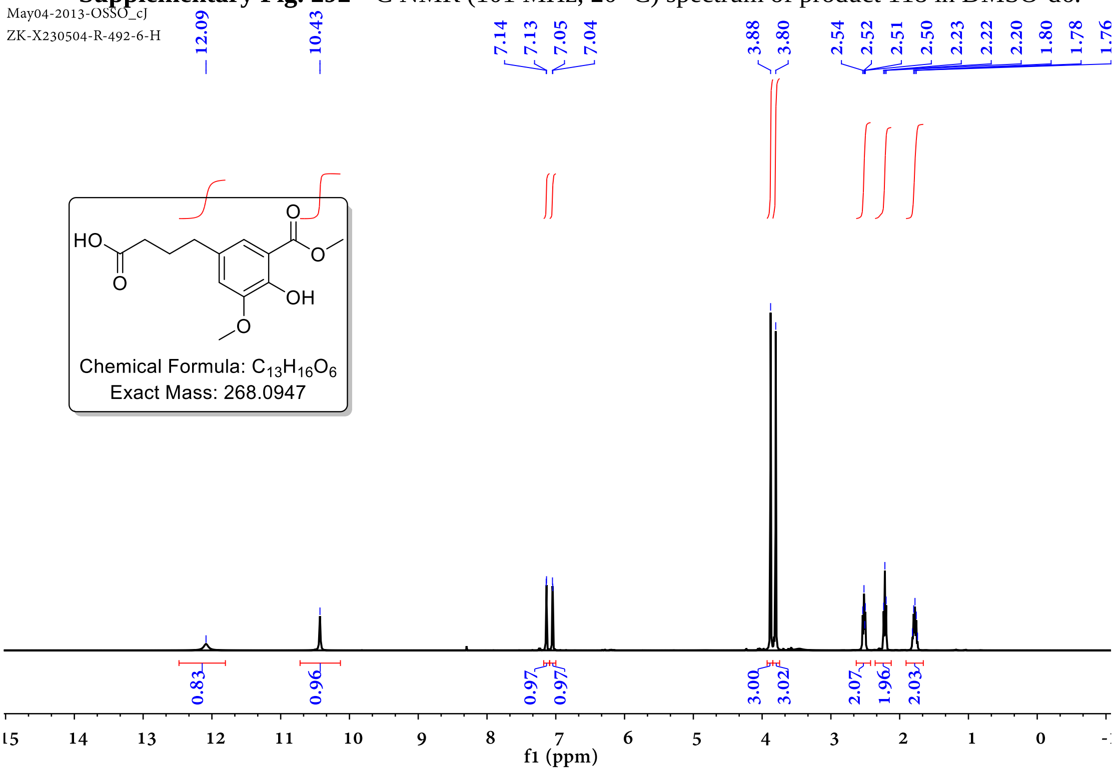

Supplementary Fig. 293 ^{1}H NMR (400 MHz, 20 °C) spectrum of product 119 in DMSO-d6.

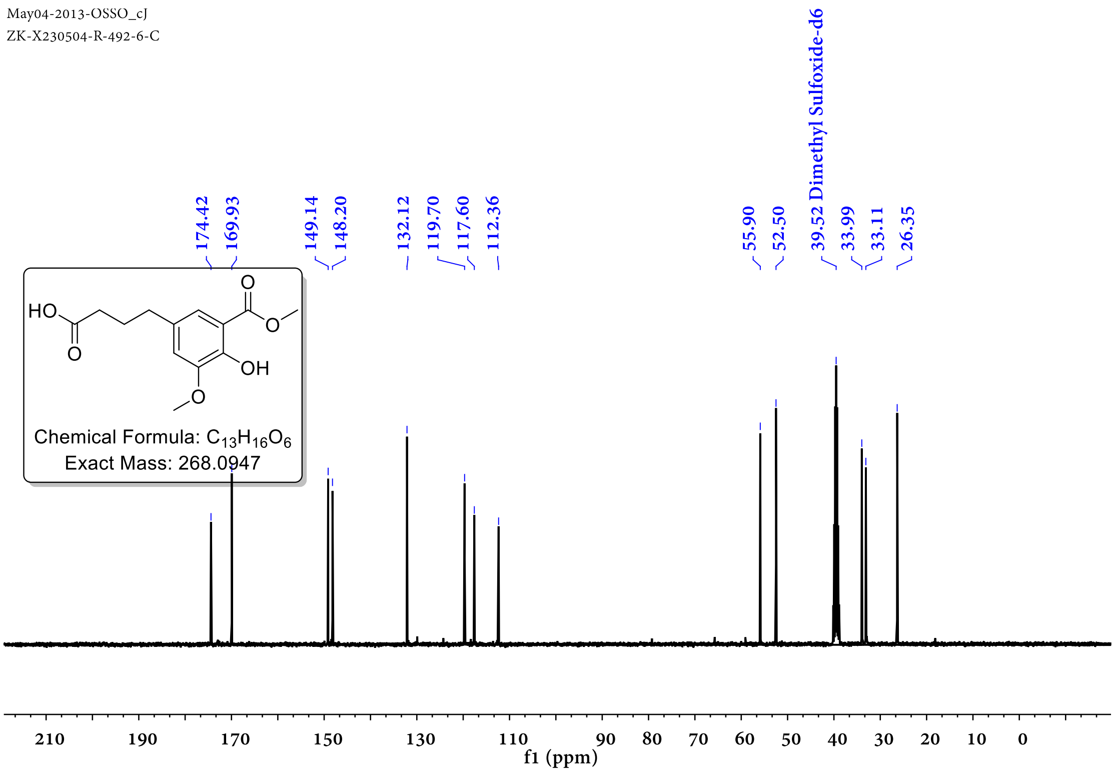

Supplementary Fig. 294 ^{13}C NMR (101 MHz, 20 °C) spectrum of product 119 in DMSO-d6.

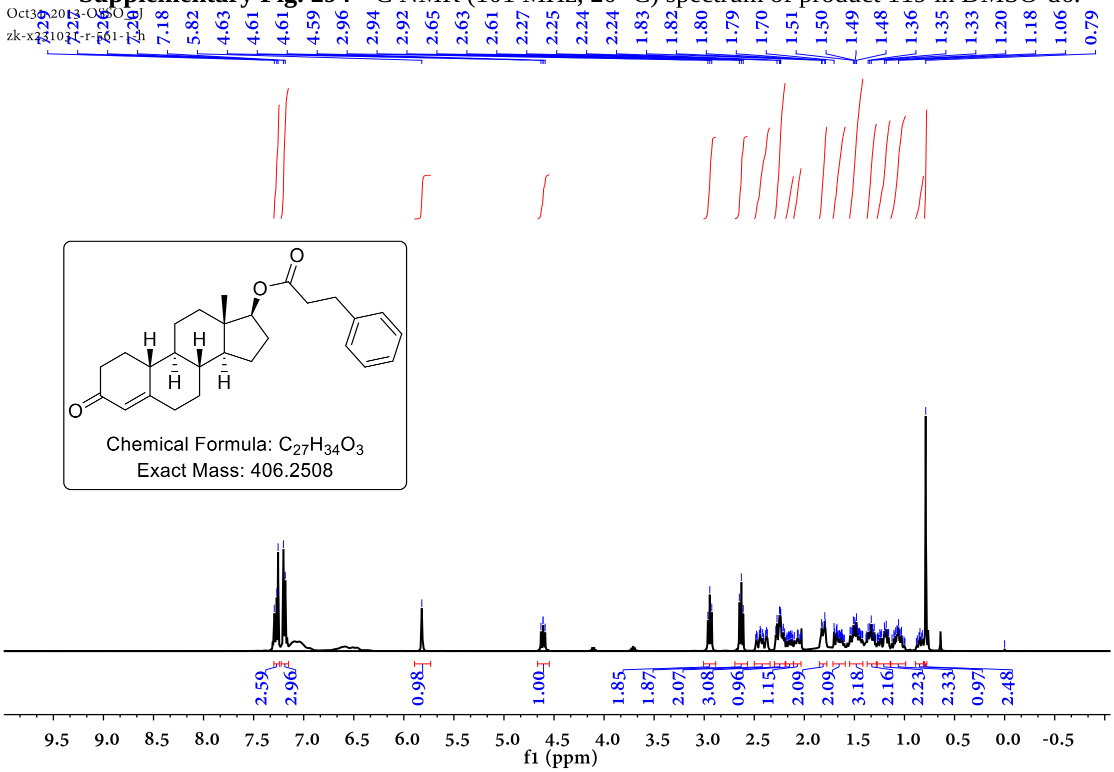

Supplementary Fig. 295 ^{1}H NMR (400 MHz, 20 °C) spectrum of product 120 in $CDCl_3$.

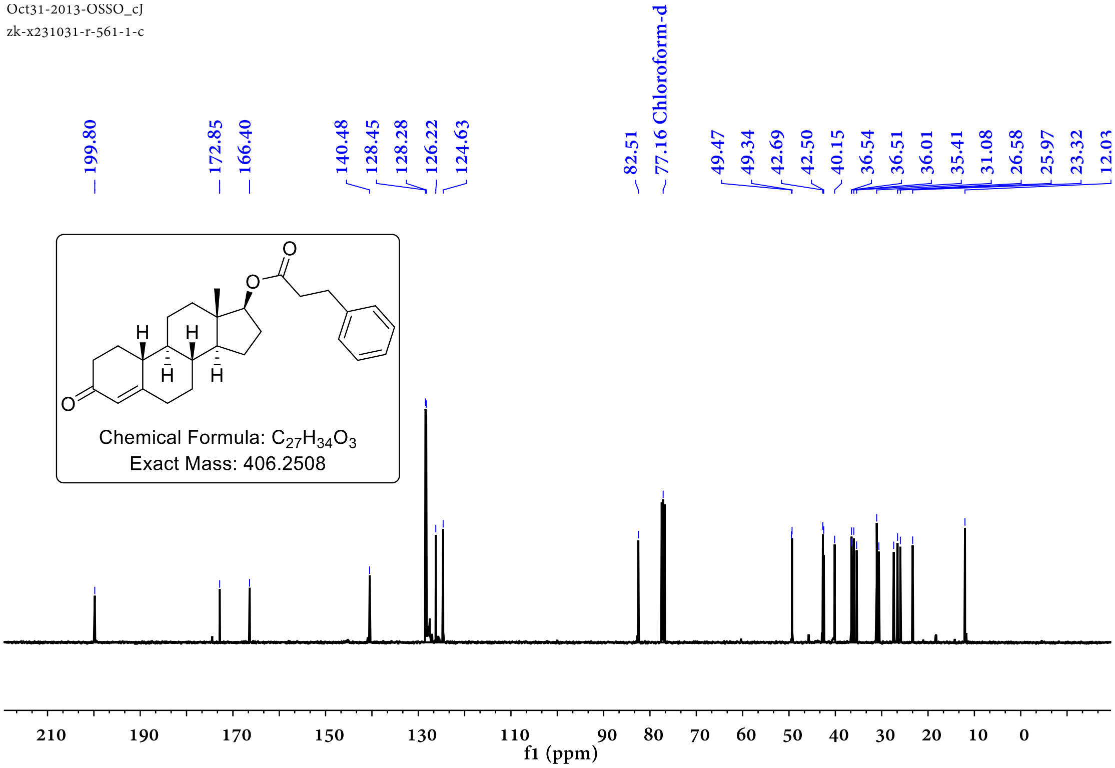

Supplementary Fig. 296 ^{13}C NMR (101 MHz, 20 °C) spectrum of product 120 in $CDCl_3$.

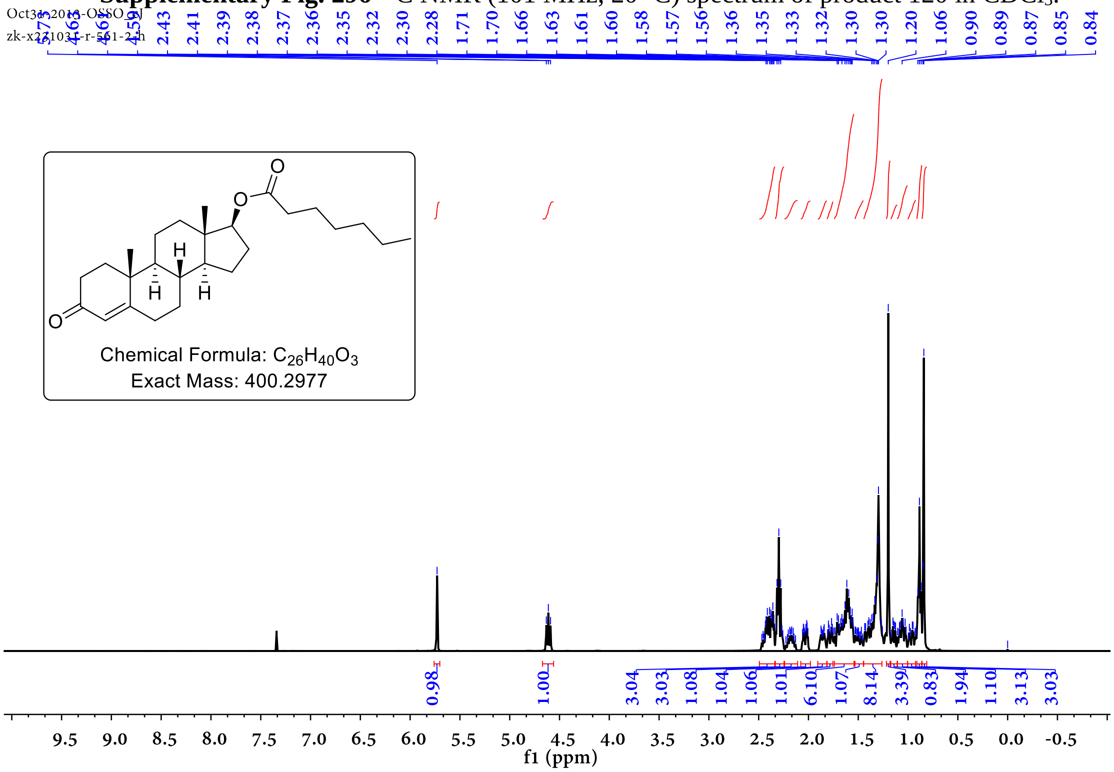

Supplementary Fig. 297 ^{1}H NMR (400 MHz, 20 °C) spectrum of product 121 in $CDCl_3$.

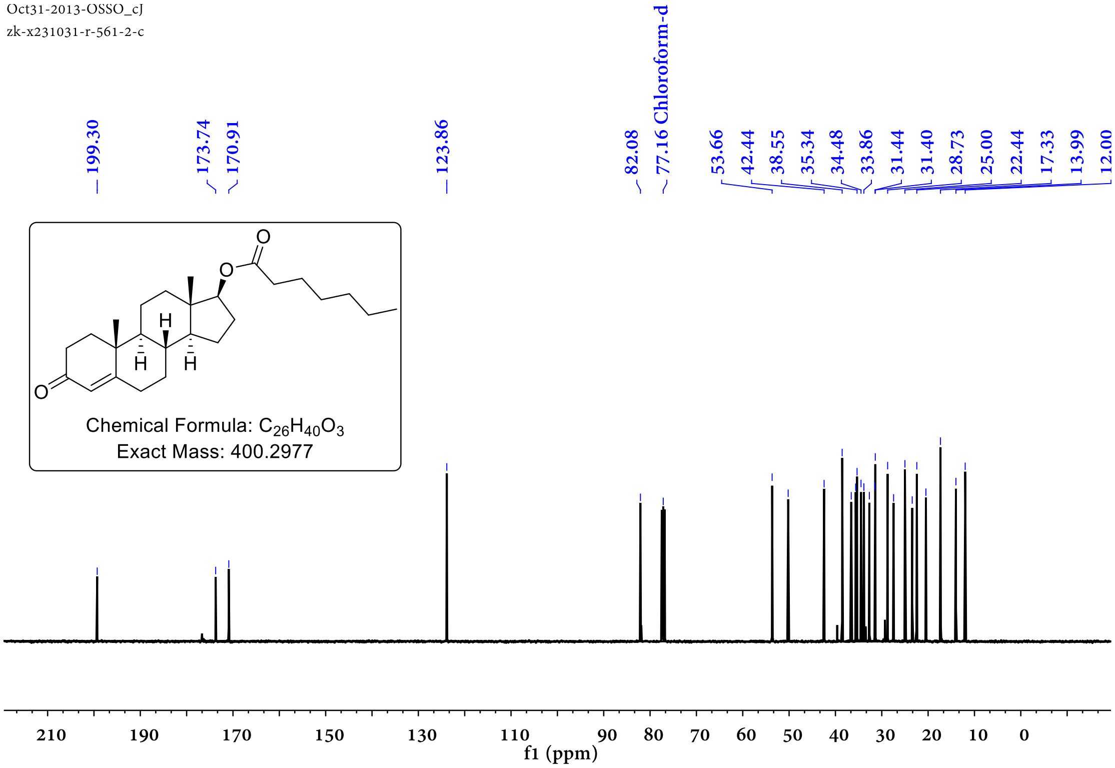

Supplementary Fig. 298 ^{13}C NMR (101 MHz, 20 °C) spectrum of product 121 in $CDCl_3$.

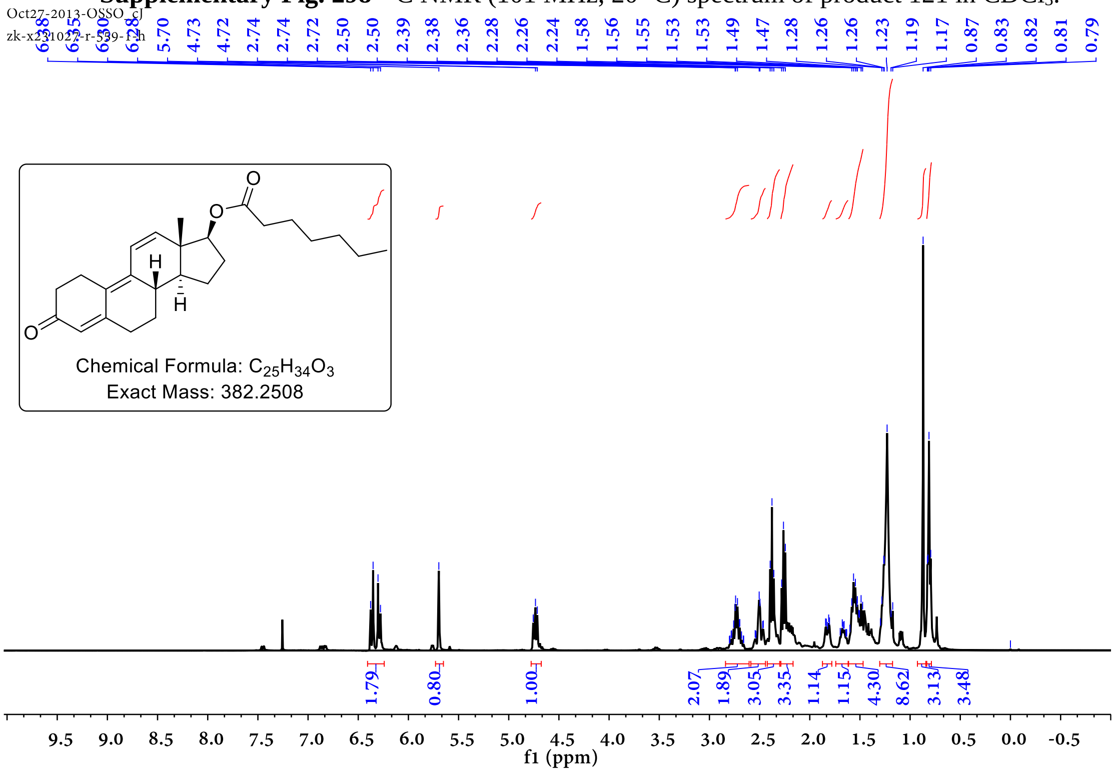

Supplementary Fig. 299 ^{1}H NMR (400 MHz, 20 °C) spectrum of product 122 in $CDCl_3$.

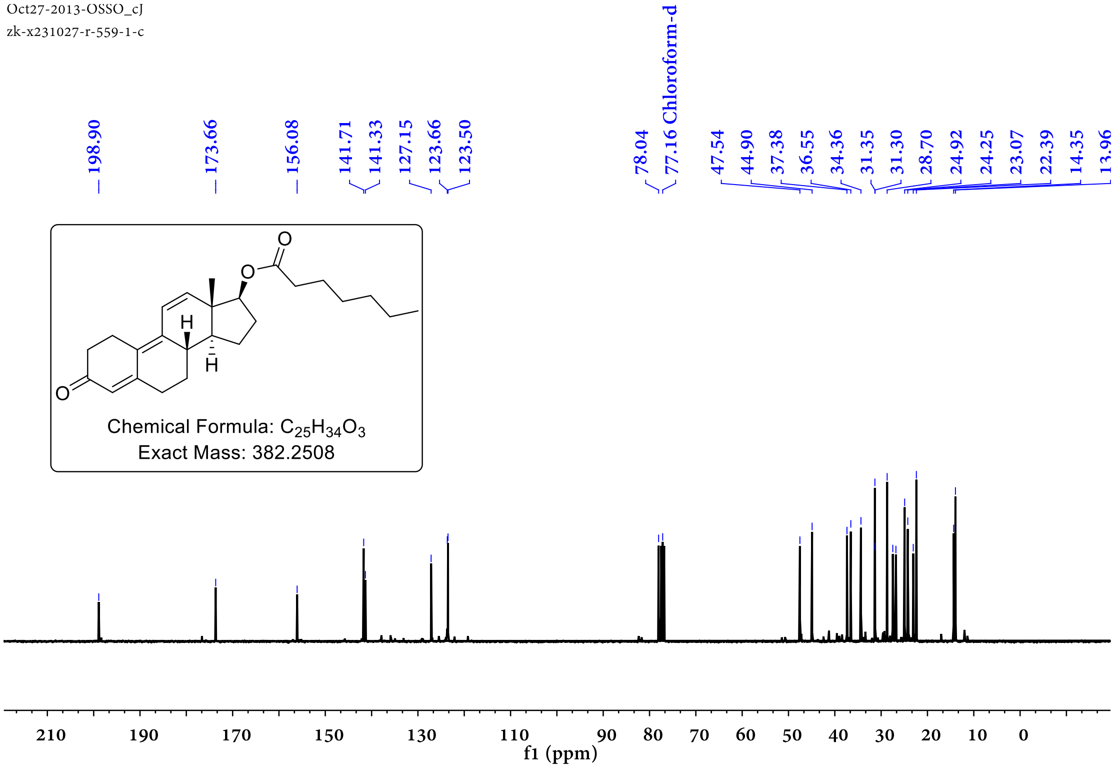

Supplementary Fig. 300 ^{13}C NMR (101 MHz, 20 °C) spectrum of product 122 in $CDCl_3$.

Supplementary Note 10: References

1. Hu, X., Wang, G., Qin, C. et al. Ligandless nickel-catalyzed transfer hydrogenation of alkenes and alkynes using water as the hydrogen donor. *Org. Chem. Front.* **6**, 2619-2623 (2019).
2. Molloy, J. J., Seath, C. P., West, M. J. et al. Interrogating pd(ii) anion metathesis using a bifunctional chemical probe: A transmetalation switch. *J. Am. Chem. Soc.* **140**, 126-130 (2018).
3. Li, Y., Jiang, X., Zhao, C. et al. Silver-catalyzed anti-markovnikov hydroxyfluorination of styrenes. *ACS Catal.* **7**, 1606-1609 (2017).
4. Mato, M., Herle, B. & Echavarren, A. M. Cyclopropanation by gold- or zinc-catalyzed retro-buchner reaction at room temperature. *Org. Lett.* **20**, 4341-4345 (2018).
5. Wood, T. K., Piers, W. E., Keay, B. A. et al. Synthesis and comparative characterization of 9-boraanthracene, 5-boranaphthacene, and 6-borapentacene stabilized by the h(2)imes carbene. *Chem.- Eur. J.* **16**, 12199-12206 (2010).
6. Tan, M.-L., Tong, S., Hou, S.-K. et al. Copper-catalyzed n,n-diarylation of amides for the construction of 9,10-dihydroacridine structure and applications in the synthesis of diverse nitrogen-embedded polyacenes. *Org. Lett.* **22**, 5417-5422 (2020).
7. Konishi, A., Hirao, Y., Nakano, M. et al. Synthesis and characterization of teranthene: A singlet biradical polycyclic aromatic hydrocarbon having kekulé structures. *J. Am. Chem. Soc.* **132**, 11021-11023 (2010).
8. Wyatt, P., Hudson, A., Charmant, J. et al. Synthesis and chemistry of enantiomerically pure 10,11-dihydrodibenzo[b,f]thiepines. *Org. Biomol. Chem.* **4**, 2218-2232 (2006).
9. Thu-Hong, D., Chardon, A., Osi, A. et al. Methylene bridging effect on the structures, lewis acidities and optical properties of semi-planar triarylboranes. *Chem.- Eur. J.* **27**, 1736-1743 (2021).
10. Jing, Q., Sandoval, C. A., Yoshiki, Y. et al. Solution structure and behavior of benzophenone-based achiral bisphosphine ligands in noyori-type ru(ii)-catalysts. *Chin. J. Chem.* **25**, 1163-1170 (2007).
11. Amézquita-Valencia, M., Achonduh, G. & Alper, H. Pd-catalyzed regioselective alkoxycarbonylation of 1-alkenes using a lewis acid [sncl2 or ti(oipr)4] and a phosphine. *J. Org. Chem.* **80**, 6419-6424 (2015).
12. Dong, K., Fang, X., Gülak, S. et al. Highly active and efficient catalysts for alkoxycarbonylation of alkenes. *Nat. Commun.* **8**, 14117-14123 (2017).
13. Paul, A., Smith, M. D. & Vannucci, A. K. Photoredox-assisted reductive cross-coupling: Mechanistic insight into catalytic aryl – alkyl cross-couplings. *J. Org. Chem.* **82**, 1996-2003 (2017).
14. Amezquita-Valencia, M., Achonduh, G. & Alper, H. Pd-catalyzed regioselective alkoxycarbonylation of 1-alkenes using a lewis acid [sncl2 or ti(oipr)4] and a phosphine. *J. Org. Chem.* **80**, 6419-6424 (2015).
15. Dannecker, P.-K., Biermann, U., von Czapiewski, M. et al. Renewable polyethers via gabr3-catalyzed reduction of polyesters. *Angew. Chem. Int. Ed.* **57**, 8775-8779 (2018).
16. Torron, S. & Johansson, M. Oxetane-terminated telechelic epoxy-functional polyesters as cationically polymerizable thermoset resins: Tuning the reactivity with structural design. *J. Polym. Sci., Part A: Polym. Chem.* **53**, 2258-2266 (2015).
17. Chen, H., Chen, D.-H. & Huang, P.-Q. Ni-catalyzed direct alcoholysis of n-acylpyrrole-type tertiary amides under mild conditions. *Sci. China: Chem.* **63**, 370-376 (2020).
18. Vieira, T. O., Green, M. J. & Alper, H. Highly regioselective anti-markovnikov palladium-borate-catalyzed methoxycarbonylation reactions: Unprecedented results for aryl olefins. *Org. Lett.* **8**, 6143-6145 (2006).
19. Lou, J., Wang, Q., He, Y. et al. A simple aliphatic diamine auxiliary for palladium-catalyzed arylation of unactivated β-c(sp^3)-h bonds. *Adv. Synth. Catal.* **360**, 4571-4584 (2018).
20. Ma, X. & Herzon, S. B. Synthesis of ketones and esters from heteroatom-functionalized alkenes by cobalt-mediated hydrogen atom transfer. *J. Org. Chem.* **81**, 8673-8695 (2016).
21. Kerim, M. D., Jia, S., Theodorakidou, C. et al. Palladium triggered diene formation from nitro allylic compounds: A versatile entry into naphthalene derivatives. *Chem. Commun.* **54**, 10917-10920 (2018).
22. Guissart, C., Barros, A., Rosa Barata, L. et al. Broadly applicable ytterbium-catalyzed esterification, hydrolysis, and amidation of imides. *Org. Lett.* **20**, 5098-5102 (2018).
23. Bobbitt, J. M., Bartelson, A. L., Bailey, W. F. et al. Oxoammonium salt oxidations of alcohols in the presence of pyridine bases. *J. Org. Chem.* **79**, 1055-1067 (2014).
24. Gehrtz, P. H., Hirschbeck, V. & Fleischer, I. A recyclable co surrogate in regioselective alkoxycarbonylation of alkenes: Indirect use of carbon dioxide. *Chem. Commun.* **51**, 12574-12577 (2015).

25. Samanta, R. C. & Studer, A. N-heterocyclic carbene catalysed oxidative esterification of aliphatic aldehydes. *Org. Chem. Front.* **1**, 936-939 (2014).
26. Nicolas, M., Guittard, F. & Géribaldi, S. Stable superhydrophobic and lipophobic conjugated polymers films. *Langmuir* **22**, 3081-3088 (2006).
27. Sang, R., Kucmierczyk, P., Duehren, R. et al. Synthesis of carboxylic acids by palladium-catalyzed hydroxycarbonylation. *Angew. Chem. Int. Ed.* **58**, 14365-14373 (2019).
28. Bonaparte, A. C., Betush, M. P., Panseri, B. M. et al. Novel aerobic oxidation of primary sulfones to carboxylic acids. *Org. Lett.* **13**, 1447-1449 (2011).
29. Schneider, C., Franke, R., Jackstell, R. et al. A direct synthesis of carboxylic acids via platinum-catalysed hydroxycarbonylation of olefins. *Catal. Sci. Technol.* **11**, 2703-2707 (2021).
30. Moorthy, J. N. & Parida, K. N. Oxidative cleavage of olefins by in situ-generated catalytic 3,4,5,6-tetramethyl-2-iodoxybenzoic acid/oxone. *J. Org. Chem.* **79**, 11431-11439 (2014).
31. Obaza, J. & Smith, F. X. A malonic ester synthesis with acid chlorides. The homologation of dioic acids. *Synth. Commun.* **12**, 19-23 (1982).
32. Lemhadri, M., Doucet, H. & Santelli, M. Direct synthesis of 3-arylpropionic acids by tetraphosphine/palladium catalysed heck reactions of aryl halides with acrolein ethylene acetal. *Tetrahedron* **60**, 11533-11540 (2004).
33. Pearson, A. J. & Lee, K. Some studies on the uses of 2-bromoethyl and 2-iodoethyl ester blocking groups in peptide synthesis: Samarium diiodide-mediated deprotection. *J. Org. Chem.* **59**, 2257-2260 (1994).
34. Li, Z.-Q., Fu, Y., Deng, R. et al. Ligand-controlled regiodivergence in nickel-catalyzed hydroarylation and hydroalkenylation of alkenyl carboxylic acids. *Angew. Chem. Int. Ed.* **59**, 23306-23312 (2020).
35. Zieliński, G. K., Majtczak, J., Gutowski, M. et al. A selective and functional group-tolerant ruthenium-catalyzed olefin metathesis/transfer hydrogenation tandem sequence using formic acid as hydrogen source. *J. Org. Chem.* **83**, 2542-2553 (2018).
36. Becke, A. D. Density-functional thermochemistry. 3. The role of exact exchange. *J. Chem. Phys.* **98**, 5648-5652 (1993).
37. Perdew, J. P., Chevary, J. A., Vosko, S. H. et al. Atoms, molecules, solids, and surfaces - applications of the generalized gradient approximation for exchange and correlation. *Phys. Rev. B* **46**, 6671-6687 (1992).
38. Perdew, J. P., Chevary, J. A., Vosko, S. H. et al. Atoms, molecules, solids, and surfaces - applications of the generalized gradient approximation for exchange and correlation. *Phys. Rev. B* **48**, 4978-4978 (1993).
39. Perdew, J. P., Burke, K. & Wang, Y. Generalized gradient approximation for the exchange-correlation hole of a many-electron system. *Phys. Rev. B* **54**, 16533-16539 (1996).
40. Grimme, S., Ehrlich, S. & Goerigk, L. Effect of the damping function in dispersion corrected density functional theory. *J. Comput. Chem.* **32**, 1456-1465 (2011).
41. Crawford, L., Cole-Hamilton, D. J., Drent, E. et al. Mechanism of alkyne alkoxycarbonylation at a pd catalyst with p,n hemilabile ligands: A density functional study. *Chem. Eur. J.* **20**, 13923-13926 (2014).
42. Crawford, L., Cole-Hamilton, D. J. & Buehl, M. Uncovering the mechanism of homogeneous methyl methacrylate formation with p,n chelating ligands and palladium: Favored reaction channels and selectivities. *Organometallics* **34**, 438-449 (2015).
43. Ahmad, S., Crawford, L. E. & Buehl, M. Palladium-catalysed methoxycarbonylation of ethene with bidentate diphosphine ligands: A density functional theory study. *Phys. Chem. Chem. Phys.* **22**, 24330-24336 (2020).
44. Ahmad, S. & Buhl, M. Computational modelling of pd-catalysed alkoxycarbonylation of alkenes and alkynes. *Phys. Chem. Chem. Phys.* **23**, 15869-15880 (2021).
45. Tomasi, J., Mennucci, B. & Cammi, R. Quantum mechanical continuum solvation models. *Chem. Rev.* **105**, 2999-3093 (2005).
46. M. D. Lechner (Ed.) Ch. Wohlfarth (Auth.), "*Static dielectric constants of pure liquids and binary liquid mixtures*". In: Landolt-Börnstein: Numerical Data and Functional Relationships in Science and Technology – New Series. Group IV: Physical Chemistry. Volume 27. Springer-Verlag Berlin Heidelberg, **2015**.
47 Gaussian 16, Revision C.01, M. J. Frisch, G. W. Trucks, H. B. Schlegel, G. E. Scuseria, M. A. Robb, J. R. Cheeseman, G. Scalmani, V. Barone, G. A. Petersson, H. Nakatsuji, X. Li, M. Caricato, A. V. Marenich, J. Bloino, B. G. Janesko, R. Gomperts, B. Mennucci, H. P. Hratchian, J. V. Ortiz, A. F. Izmaylov, J. L. Sonnenberg, D. Williams-Young, F. Ding, F. Lipparini, F. Egidi, J. Goings, B. Peng, A.

Petrone, T. Henderson, D. Ranasinghe, V. G. Zakrzewski, J. Gao, N. Rega, G. Zheng, W. Liang, M. Hada, M. Ehara, K. Toyota, R. Fukuda, J. Hasegawa, M. Ishida, T. Nakajima, Y. Honda, O. Kitao, H. Nakai, T. Vreven, K. Throssell, J. A. Montgomery, Jr., J. E. Peralta, F. Ogliaro, M. J. Bearpark, J. J. Heyd, E. N. Brothers, K. N. Kudin, V. N. Staroverov, T. A. Keith, R. Kobayashi, J. Normand, K. Raghavachari, A. P. Rendell, J. C. Burant, S. S. Iyengar, J. Tomasi, M. Cossi, J. M. Millam, M. Klene, C. Adamo, R. Cammi, J. W. Ochterski, R. L. Martin, K. Morokuma, O. Farkas, J. B. Foresman, and D. J. Fox, Gaussian, Inc., Wallingford CT, 2019. 1. Zhu, S., Qin, J., Wang, F. et al. Photoredox-catalyzed branch-selective pyridylation of alkenes for the expedient synthesis of triprolidine. Nat. Commun. 10, 749-755 (2019).
