# Supplementary material for: Identification of a potent palladium-aryldiphosphine catalytic system for high-performance carbonylation of alkenes
Source: Nat Commun. 2024 Mar 5;15:2016. doi: 10.1038/s41467-024-46286-9 (PMC10914764; doi:10.1038/s41467-024-46286-9)
Supplement: Supplementary file 2 — Peer Review File [file 41467_2024_46286_MOESM2_ESM.pdf]

nature portfolio

Peer Review File

Identification of a potent palladium-aryldiphosphine catalytic system for high-performance carbonylation of alkenes

Open Access This file is licensed under a Creative Commons Attribution 4.0 International License, which permits use, sharing, adaptation, distribution and reproduction in any medium or format, as long as you give appropriate credit to the original author(s) and the source, provide a link to the Creative Commons license, and indicate if changes were made. In the cases where the authors are anonymous, such as is the case for the reports of anonymous peer reviewers, author attribution should be to 'Anonymous Referee' followed by a clear attribution to the source work. The images or other third party material in this file are included in the article's Creative Commons license, unless indicated otherwise in a credit line to the material. If material is not included in the article's Creative Commons license and your intended use is not permitted by statutory regulation or exceeds the permitted use, you will need to obtain permission directly from the copyright holder. To view a copy of this license, visit http://creativecommons.org/licenses/by/4.0/.

Editorial Note: This manuscript has been previously reviewed at another journal that is not operating a transparent peer review scheme. This document only contains reviewer comments and rebuttal letters for versions considered at *Nature Communications*.

REVIEWERS' COMMENTS

Reviewer #1 (Remarks to the Author):

The authors have revised all the questions. I think this work can be published in Nature Communications because it has strong innovation and important findings.

Reviewer #2 (Remarks to the Author):

The revised work entitled "Identification of a potent palladium-aryldiphosphine catalytic system for high-performance carbonylation of alkenes" by Kang Zhao, Hongli Wang, Teng Li, Shujuan Liu, Enrico Benassi, Xiao Li, Yao Yao, Xiaojun Wang, Xinjiang Cui, Feng Shi submitted for publication in "Nature Commnications" is an improved version of the past submitted version, and again I want to remark the impressive performance of the carbonylation of alkenes with a TON over 2 million.
The authors have addressed not only all my concerns, but also from the other referees. Particularly the mechanistic changes in terms of format are welcome.

Reviewer #1 (Remarks to the Author):

The authors have revised all the questions. I think this work can be published in Nature Communications because it has strong innovation and important findings.

Reply: Thanks very much for your kind work and consideration on publication of our manuscript.

Reviewer #2 (Remarks to the Author):

The revised work entitled "Identification of a potent palladium-aryldiphosphine catalytic system for high-performance carbonylation of alkenes" by Kang Zhao, Hongli Vang, Teng Li, Shujuan Liu, Enrico Benassi, Xiao Li, Yao Yao, Xiaojun Wang, Xinjiang Cui, Feng Shi submitted for publication in "Nature Communications" is an improved version of the past submitted version, and again I want to remark the impressive performance of the carbonylation of alkenes with a TON over 2 million.

The authors have addressed not only all my concerns, but also from the other referees. Particularly the mechanistic changes in terns of format are welcome.

Reply: Thanks very much for your kind work and consideration on publication of our manuscript.

Reply to editorial:

(1) Thank you for submitting your manuscript "Identification of a potent palladium-aryldiphosphine catalytic system for high-performance carbonylation of alkenes" to Nature Communications. I am delighted to say that we are happy, in principle, to publish it under the open access CC BY license (Creative Commons Attribution 4.0 International License).

Reply: Thanks very much for your kind work and consideration on publication of our manuscript.

(2) First, we ask you to revise your paper one last time to address our editorial requests (in the attached author checklist) and any remaining comments from reviewers (included at the end of this email, if applicable).

Reply: The editorial requests have been addressed point-by-point according to the attached author checklist. All remaining comments from reviewers have been addressed point-by-point and presented in this cove letter.

(3) Failure to comply with our editorial requests will cause delays in accepting your manuscript. Please also see the Nature Communications formatting instructions for further information.

Reply: All editorial requests have been solved.
